# Palladium-catalyzed difluoromethylthiolation of heteroaryl bromides, iodides, triflates and aryl iodides†
†Electronic supplementary information (ESI) available. CCDC 1436785. For ESI and crystallographic data in CIF or other electronic format see DOI: 10.1039/c6sc00082g


**DOI:** 10.1039/c6sc00082g

**Published:** 2016-02-17

**Authors:** Jiang Wu, Yafei Liu, Changhui Lu, Qilong Shen

**Affiliations:** a Key Laboratory of Organofluorine Chemistry , Shanghai Institute of Organic Chemistry , Chinese Academy of Sciences , 345 Lingling Road , Shanghai 200032 , China . Email: shenql@sioc.ac.cn

## Abstract

A palladium-catalyzed difluoromethylthiolation of heteroaryl halides and triflates under mild conditions was described. A vast range of heteroaryl halides such as pyridyl, quinolinyl, benzothiazolyl, thiophenyl, carbazolyl and pyazolyl halides could be difluoromethylthiolated efficiently, thus providing medicinal chemists with new tools for their search of new lead compounds for drug discovery. Likewise, aryl iodides were difluoromethylthiolated in high yields under a modified reaction condition.

## Abstract 2

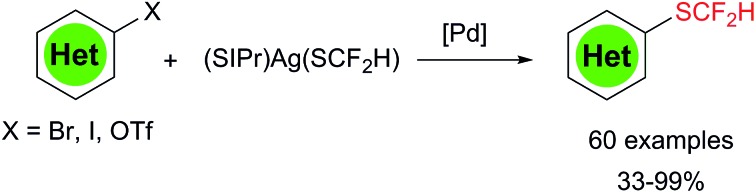
A palladium-catalyzed difluoromethylthiolation of heteroaryl halides and triflates under mild conditions was described.

## Introduction

Heteroarenes are common structural motifs and generally play key roles in pharmaceuticals and agrichemicals, as evidenced by the fact that 640 out of 1086 small molecules approved by the U.S. Food and Drug administration (FDA) are heterocycles.^1^ On the other hand, the advantageous impact of the fluorine and fluoroalkyl groups in the field of medicinal chemistry and agro-chemistry has also been well-recognized and practised.^2^ Very recently, one of the fluoroalkyl groups, the difluoromethylthio group (SCF_2_H), has attracted increasing attention.^3^ The difluoromethylthio group (Hansch parameter *π*_R_ = 0.68)^4^ is slightly more lipophilic than the methyl group (*π*_R_ = 0.56), but much less lipophilic than its analog the trifluoromethylthio group (CF_3_S, *π*_R_ = 1.44), providing medicinal chemists the opportunity to fine tune the drug molecule's lipophilicity. In addition, the electron-withdrawing nature of the difluoromethylthio group would increase the drug molecule's metabolic stability. Furthermore, the difluoromethylthio group, bearing an acidic proton that can act as a hydrogen bonding donor to interact with the enzyme, could enhance the drug molecule's binding selectivity.3*b* Thus, if the beneficial effects of the difluoromethylthio groups could be combined with the appreciated heteroarene structural motif, a synergistic effect would be achieved that would improve on the drug molecule's overall efficacy.

Despite the potential of the difluoromethylthioheteroarene unit for the drug molecule's properties, its widespread application in medicinal studies remains rather limited due to a lack of efficient synthetic methods. Limited examples for the formation of heteroaryldifluoromethylthioether *via* difluoromethylation of simple heteroaryl thiolates with a difluorocarbene precursor, a difluoromethyl radical reagent or an electrophilic difluoromethyl reagent have been reported.^5^ Nevertheless, various base-sensitive functional groups were not compatible with the strong basic conditions in these reactions. Furthermore, except for some structurally simple heteroarylthiols, few methods are available for the formation of structurally more complicated heteroarylthiols. More recently, our3*e* and Goossen's group3c,d independently reported that the heteroaryldifluoromethylthioether could be accessed from heteroaryldiazonium salts under mild conditions. Yet, the explosive nature of the heteroaryldiazonium salts requires extreme care when handling which limits their practical applications.^6^ Alternatively, we recently discovered an electrophilic difluoromethylthiolating reagent – difluoromethyl–thiophthalimide, that was able to direct difluoromethylthiolate electron-rich heteroarenes such as indoles, pyrroles, imidazo[1,2-*a*]pyridine, aminothiazole and isoxazole in the presence of a Lewis acid.3*f* However, no desired difluoromethylthiolated heteroarenes were observed when other less electron-rich or electron-poor heteroarenes such as benzothiophene, benzofuran or pyridine were subjected to the reaction conditions. Thus, new and efficient methods that allow for difluoromethylthiolation of a broad range of common heteroarenes are highly desirable.

Palladium-catalyzed cross-coupling reactions^7^ represent one of the most utilized synthetic tools for the formation of carbon–carbon and carbon–heteroatom bonds.^8^ In this respect, several palladium-catalyzed couplings of aryl halides with fluoroalkylated nucleophiles for trifluoromethylation, difluoromethylation, monofluoromethylation and trifluoromethylthiolation under significantly different reaction conditions^9^ have been developed, while the analogous difluoromethylthiolation has not been reported. One main reason behind the fluoroalkyl group's different behavior is due to the unique properties of fluoroalkyl groups. One subtle change in the structure of fluoroalkyl groups may lead to dramatic stability and reactivity differences. For instance, AgSCF_3_ is a stable solid and is known to be a good coupling partner for aryl and heteroaryl iodides and bromides, while its analog AgSCF_2_H (or another nucleophilic difluoromethylthiolating reagent) is an unknown species and many efforts to synthesize it have all failed. Since we recently discovered that a bulky *N*-heterocyclic carbene (SIPr) can stabilize AgSCF_2_H,3*e* we wondered whether this reagent [(SIPr)Ag(SCF_2_H)] can be utilized in the palladium-catalyzed difluoromethylthiolation of various heteroaryl halides. Herein, we report the realization of such a new cross-coupling reaction under mild reaction conditions. A vast range of different difluoromethylthiolated heteroarenes could then be accessed, thus providing medicinal chemists with new tools for their search of new lead compounds for drug discovery. In addition, under a modified condition, the difluoromethylthiolation of aryl iodides were also equally achieved.

## Results and discussion

**Fig. 1:**
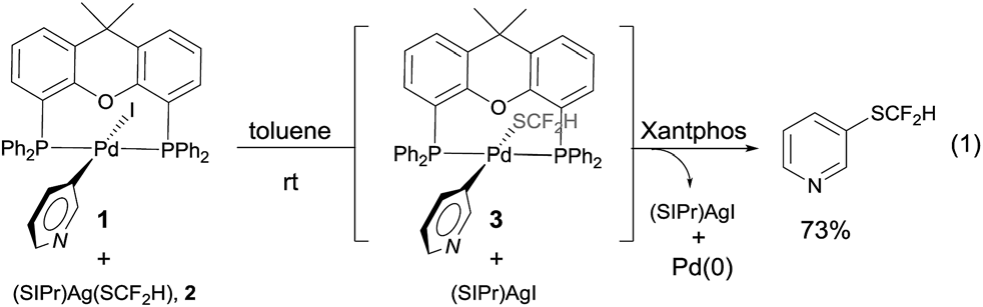
Stoichiometric reaction of complex [(Xantphos)Pd(3-Py)(I)] **1** with [(SIPr)Ag(SCF_2_H)] **2** at room temperature.

One concern for the palladium-catalyzed difluoromethylthiolation of heteroaryl halides is the reductive-elimination step of the commonly proposed catalytic cycle since it is well-known that the reductive-elimination from heteroaryl–palladium complexes proceeds with much more difficulty than those of aryl–palladium complexes.^10^ To probe whether the reductive-elimination from the putative intermediate [L_2_Pd(heteroaryl)(SCF_2_H)] **3** is problematic, we synthesized the Xantphos–ligated complex^11^ [(Xantphos)Pd(3-Py)(I)] **1** and studied its reaction with [(SIPr)Ag(SCF_2_H)] **2** with various solvents and at various temperatures. To our surprise, the reaction of [(Xantphos)Pd(3-Py)(I)] with 1.5 equivalents of [(SIPr)Ag(SCF_2_H)] in toluene occurred smoothly at room temperature after 1.0 h to afford the reductive-elimination product 3-difluoromethylthiopyridine as the only product in a 73% yield, as determined by ^19^F NMR spectroscopy of the reaction mixture (Fig. 1). These results indicate that reductive-elimination to form heteroaryldifluoromethylthioether is a fast process and would not likely be the turnover limiting step of the possible palladium-catalyzed difluoromethylthiolating reaction.

**Scheme 1:**
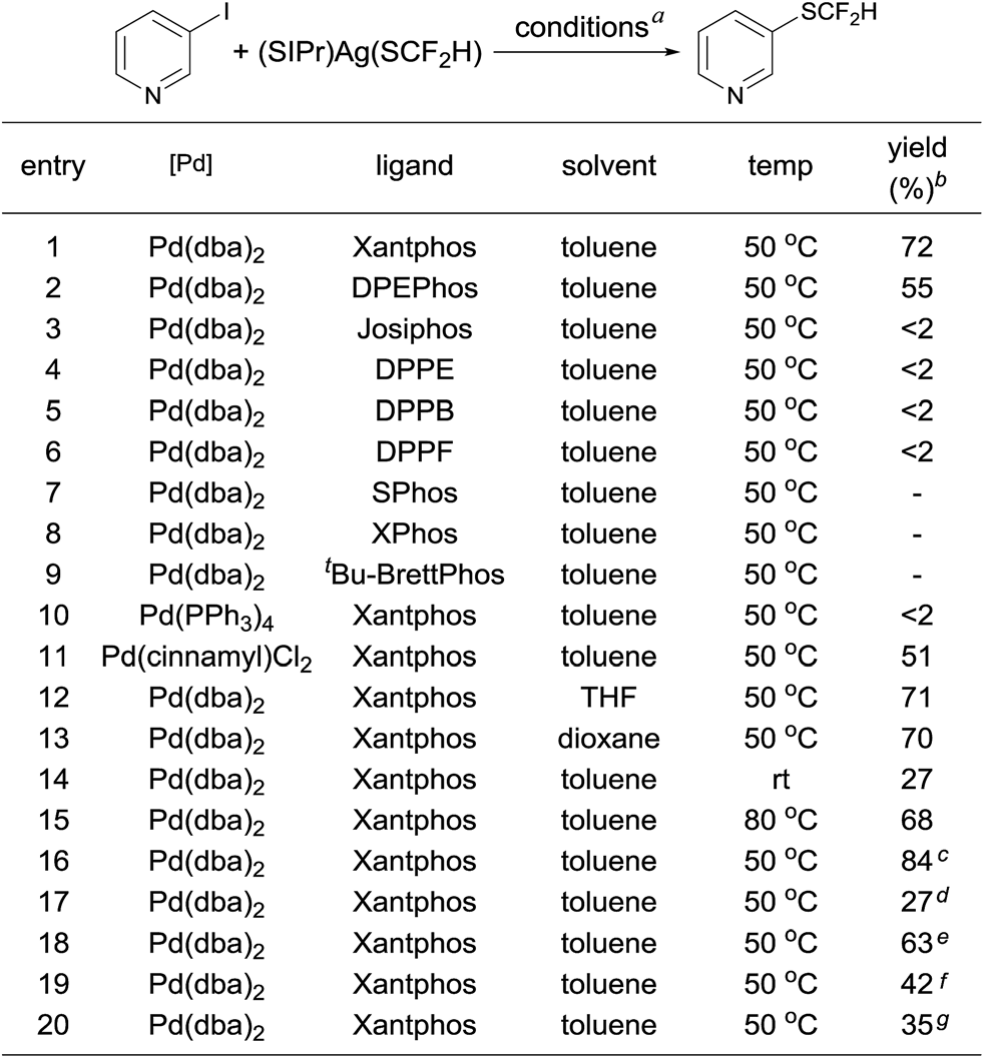
Optimization of the palladium-catalyzed difluoromethylthiolation of 3-Iodopyridine. ^*a*^Reaction conditions: 3-iodopyridine (0.05 mmol), (SIPr)Ag(SCF_2_H) **2** (0.05 mmol), palladium precursor (10 mol%), and ligand (15 mol%) in different solvents (1.0 mL) at 50 °C for 12 h; ^*b*^yields were determined by ^19^F NMR analysis of the crude reaction mixture with trifluorotoluene as an internal standard; ^*c*^1.2 equiv. of (SIPr)Ag(SCF_2_H) **2** was used; ^*d*^Pd(dba)_2_ (10 mol%) and Xantphos (10 mol%) were used as the catalyst; ^*e*^Pd(dba)_2_ (10 mol%) and Xantphos (20 mol%) were used as the catalyst; ^*f*^Pd(dba)_2_ (7.5 mol%) and Xantphos (11.3 mol%) were used as the catalyst; ^*g*^Pd(dba)_2_ (5.0 mol%) and Xantphos (7.5 mol%) were used as the catalyst.

The observed stoichiometric reaction was successfully translated into a catalytic reaction. The reaction of 3-iodopyridine with reagent **2** in the presence of 10 mol% Pd(dba)_2_ and 15 mol% Xantphos occurred smoothly after 12 h at 50 °C to give 3-difluoromethylthiopyridine in a 72% yield (Scheme 1, entry 1). The reaction using DPEPhos as the ligand generated the corresponding product in a 55% yield, while reactions using other bidentate ligands such as DPPE, DPPB, and DPPF occurred in less than 2% yields (Scheme 1, entries 3–6). Interestingly electron-rich, monodentate alkyl phosphines such as BrettPhos or XPhos that can be used efficiently in the palladium-catalyzed trifluoromethylthiolation were not effective at all9*d* (Scheme 1, entries 7–9). We then further studied the effect of solvent and temperature. It turned out that reactions in other solvents such as THF or dioxane gave comparable yields (Scheme 1, entries 12–13). Reactions conducted at room temperature or 80 °C occurred in lower conversions (Scheme 1, entries 14–15). Further studies indicated that the ratio of palladium to ligand is very important for the reaction. Using a combination of 10 mol% Pd(dba)_2_ and 10 mol% Xantphos as the catalyst was much less effective while reaction with 10 mol% Pd(dba)_2_ and 20 mol% Xantphos occurred in a slightly lower yield (Scheme 1, entries 17–18). Finally, reactions with a lower catalyst loading resulted in 35–42% yields (Scheme 1, entries 19–20).

**Scheme 2:**
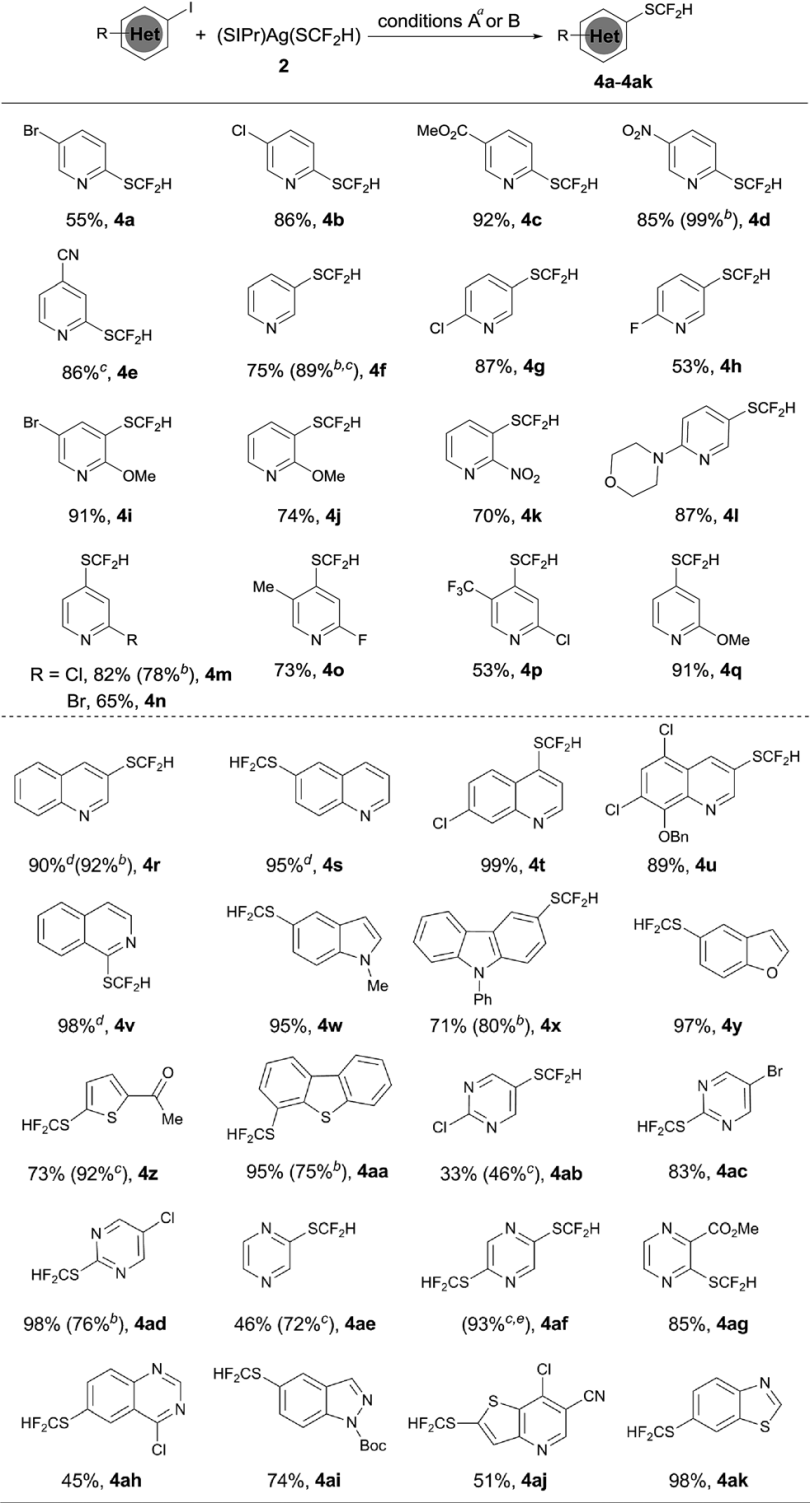
Scope of the palladium-catalyzed difluoromethylthiolation of heteroaryl iodides. ^*a*^Reaction conditions A: heteroaryl iodide (0.5 mmol), (SIPr)Ag(SCF_2_H) **2** (0.6 mmol), Pd(dba)_2_ (10 mol%), and Xantphos (15 mol%) in toluene (2.5 mL) at 50 °C for 12 h; isolated yields; ^*b*^reaction conditions B: heteroaryl iodide (0.5 mmol), (SIPr)Ag(SCF_2_H) **2** (0.6 mmol), (XantPhos)Pd(3-py)(Br) (5.0 mol%), and XantPhos (2.5 mol%) in toluene (5.0 mL) at 50 °C for 6 h; isolated yields; ^*c*^yields were determined by ^19^F NMR analysis of the crude reaction mixture with trifluorotoluene as an internal standard; ^*d*^Pd(dba)_2_ (10 mol%) and DPEPhos (10 mol%) were used; ^*e*^2-bromo-5-iodopyrazine and 2.4 equiv. of (SIPr)Ag(SCF_2_H) **2** were used.

The optimized reaction conditions for difluoromethylthiolation of 3-iodopyridine turned out to be rather effective for difluoromethylthiolation of a variety of heteroaryl iodides, as summarized in Scheme 2. In general, pyridyl iodides bearing a halogen in the 2, 3, or 4-position with electron-donating or withdrawing groups underwent difluoromethylthiolation in high yields (Scheme 2, **4a–q**). For example, the reaction of 2-chloro-5-iodopyridine with [(SIPr)Ag(SCF_2_H)] **2** occurred after 12 h at 50 °C to give 2-chloro-5-difluoromethylthiopyridine in a 87% yield (**4g**), while the same reaction with 4-iodo-2-methoxypyridine occurred in a 91% yield (**4q**). As well as pyridyl iodides, the reactions of iodine-substituted quinoline, isoquinoline, indole, carbazole, benzofuran, thiophene, or dibenzothiophene also occurred in good to excellent yields (Scheme 1, **4r–4aa**). In addition, iodo-substituted heteroarenes with two hetero-atoms such as pyrimidine, pyrazine, quinazoline, indazole, thieno[3,2-*b*]pyridine, and benzothiazole can all be difluoromethylthiolated in high yields (Scheme 1, **4ab–ak**). Interestingly, in most cases, when a heteroarene containing both an iodo group and another halogen such as fluoride, chloride or bromide was subjected to the reaction conditions, the iodo group was preferentially difluoromethylthiolated (Scheme 2, **4a–b**, **4g–i**, **4m–p**, **4t–u**, **4ab–ad**, **4ah**, **4aj**). However, the bisdifluoromethylthiolated pyrazine was obtained as the only product when 2-bromo-5-iodopyrazine was used (**4af**). Notably, a variety of functional groups other than halogens such as nitro, cyano, ester, and enolizable ketone or protected phenolic hydroxyl groups were compatible with the reaction conditions (Scheme 2, **4c–e**, **4k–l**, **4u**, **4z**, **4ag**, **4aj**). These compounds are of great interest since they allow further functional group transformation or derivatization. Interestingly, the isolated complex [(Xantphos)Pd(3-py)(Br)] could also serve as the catalyst for the same transformation. In these cases, the catalyst loading could be decreased to 5.0 mol% (Scheme 2, **4d**, **4f**, **4m**, **4r**, **4x**, **4aa** and **4ad**).

**Scheme 3:**
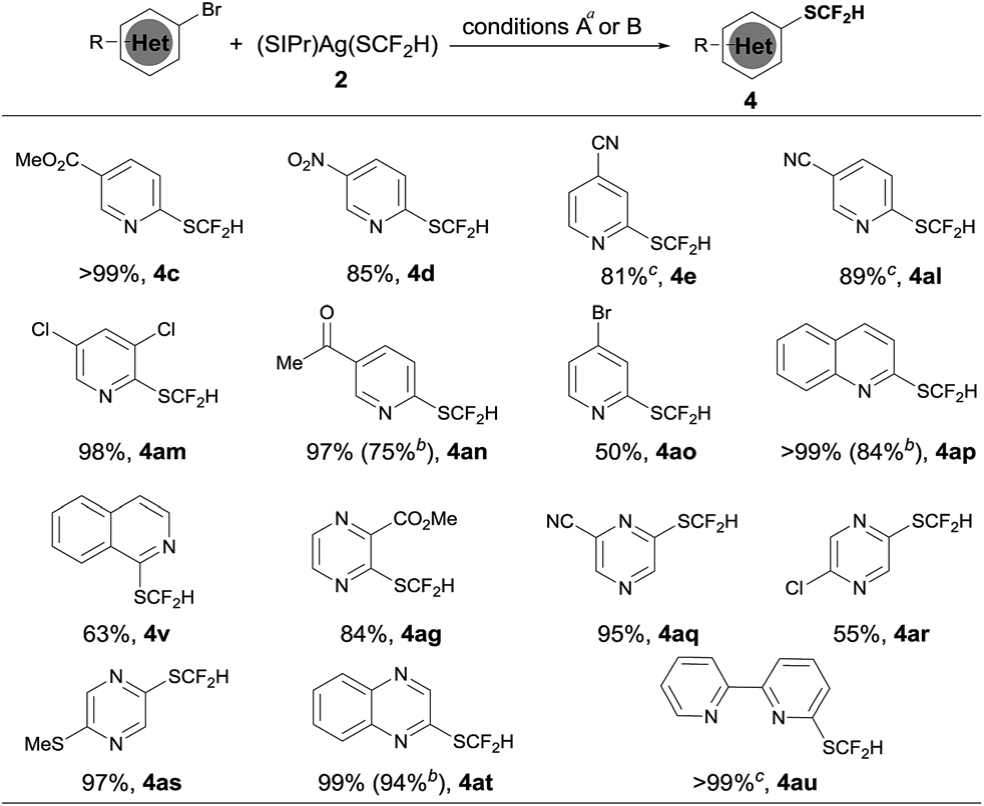
Scope of the palladium-catalyzed difluoromethylthiolation of heteroaryl bromides. ^*a*^Reaction conditions A: heteroaryl bromides (0.5 mmol), (SIPr)Ag(SCF_2_H) **2** (0.6 mmol), Pd(dba)_2_ (10 mol%), and Xantphos (15 mol%) in toluene (2.5 mL) at 50 °C for 6 h; isolated yields; ^*b*^reaction conditions B: heteroaryl bromides (0.5 mmol), (SIPr)Ag(SCF_2_H) **2** (0.6 mmol), (XantPhos)Pd(3-py)(Br) (5.0 mol%), and XantPhos (2.5 mol%) in toluene (5.0 mL) at 50 °C for 6 h; isolated yields; ^*c*^yields were determined by ^19^F NMR spectroscopy using trifluorotoluene as the internal standard.

Since we observed that heteroaryl bromide can also be difluoromethylthiolated in the case of **4af**, we studied the reaction of heteroaryl bromides with [(SIPr)Ag(SCF_2_H)] **2** in detail, as summarized in Scheme 3. Various activated heteroaryl bromides could be difluoromethylthiolated in high yields, while reactions of the electron-rich heteroaryl bromides occurred in less than 5% conversion. Heteroaryl bromides bearing two bromo groups at different positions reacted preferentially at the activated carbon–bromide bond. For example, the reaction of 2,4-dibromopyridine gave 4-bromo-2-difluoromethylthiopyridine exclusively in a 50% yield (Scheme 3, **4ao**). Nevertheless, various heteroaryl bromides such as bromo–pyridine, quinoline, isoquinoline, pyrazine, and bipyridine were successfully difluoromethylthiolated in good to excellent yields. Likewise, when the isolated palladium complex [(Xantphos)Pd(3-py)(Br)] (5.0 mol%) was used as the catalyst, the reactions also occurred in comparable yields, as demonstrated in the cases of **4an**, **4ap**, **4at** in Scheme 3.

**Scheme 4:**
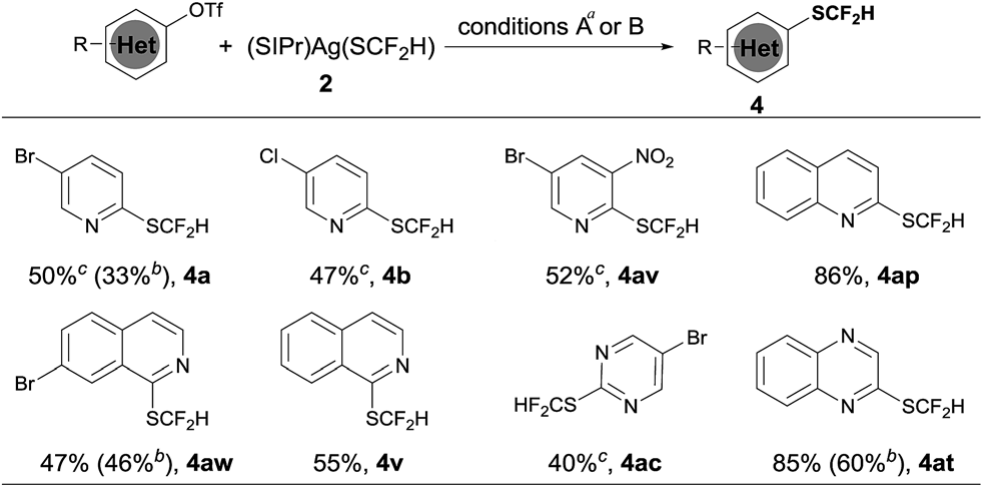
Scope of the palladium-catalyzed difluoromethylthiolation of heteroaryl triflates. ^*a*^Reaction conditions A: heteroaryl triflates (0.5 mmol), (SIPr)Ag(SCF_2_H) **2** (0.6 mmol), Pd(dba)_2_ (10 mol%), Xantphos (15 mol%) and NaBr (2.0 equiv.) in toluene (2.5 mL) at 50 °C for 6 h; isolated yields; ^*b*^reaction conditions B: heteroaryl triflates (0.5 mmol), (SIPr)Ag(SCF_2_H) **2** (0.6 mmol), (XantPhos)Pd(3-py)(Br) (6.0 mol%), XantPhos (3.0 mol%) and NaBr (2.0 equiv.) in toluene (5.0 mL) at 50 °C for 6 h; isolated yields; ^*c*^Pd(dba)_2_ (20 mol%) and XantPhos (30 mol%) were used.

Heteroaryl phenols are common in naturally occurring organic molecules and the corresponding triflic esters are valuable coupling partners for transition metal-catalyzed cross-coupling reactions.^12^ Having establishing a general and excellent protocol for the formation of heteroaryldifluoromethylthioether, we next tried to extend this method to the easily available heteroaryl triflates. However, a less than 10% conversion was observed when 5-bromo-2-pyridyl triflates were subjected to the standard conditions for heteroaryl bromides and iodides. After a quick screening of the reaction conditions, we found that the addition of 2.0 equivalents of NaBr dramatically improved the yields of the reaction. It is likely that the bromide anion could stabilize [(XantPhos)Pd(heteroaryl)(OTf)] by forming the stable palladium intermediate [(XantPhos)Pd(heteroaryl)(Br)]. Under these conditions, a variety of heteroaryl triflates could be successfully converted into their corresponding difluoromethylthiolated compounds in moderate to good yields, as summarized in Scheme 4.

**Scheme 5:**
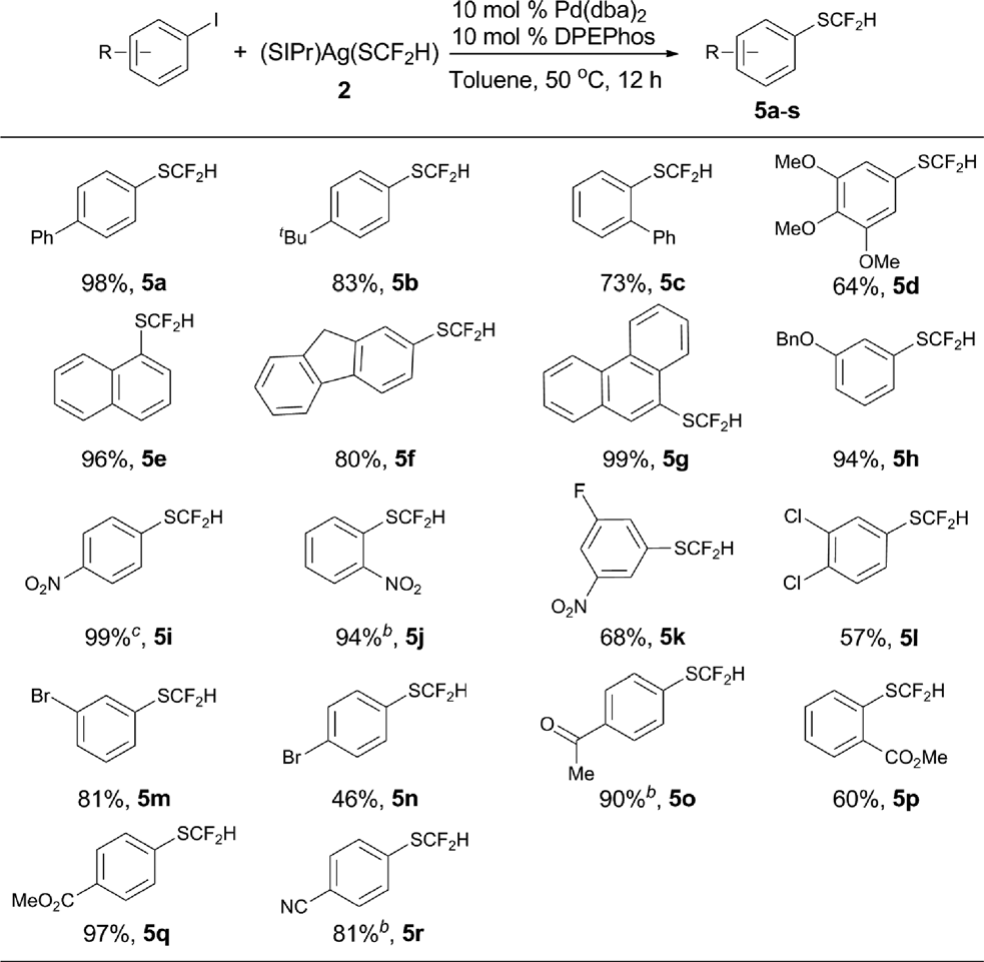
Scope of the palladium-catalyzed difluoromethylthiolation of aryl iodides. ^*a*^Reaction conditions: aryl iodides (0.5 mmol), (SIPr)Ag(SCF_2_H) **2** (0.6 mmol), Pd(dba)_2_ (10 mol%), and DPEPhos (10 mol%) in toluene (2.5 mL) at 50 °C for 12 h; isolated yields; ^*b*^Pd(dba)_2_ (7.5 mol%) and DPEPhos (7.5 mol%) were used; ^*c*^Pd(dba)_2_ (5.0 mol%) and DPEPhos (5.0 mol%) were used.

Encouraged by the broad scope of the palladium-catalyzed difluoromethylthiolation of heteroaryl substrates, we further extended this protocol to difluoromethylthiolation of aryl iodides. However, only moderate yields were observed when a combination of Pd(dba)_2_/Xantphos was used as the catalyst. Instead, high yields were obtained when DPEPhos was used as the ligand, as summarized in Scheme 5. In the presence of 10 mol% Pd(dba)_2_ and 10 mol% DPEPhos, a variety of aryl iodides were able to be difluoromethylthiolated in excellent yields. The catalyst loading can be decreased to 5–7.5 mol% when activated aryl iodides with electron-withdrawing groups are subjected to the reaction conditions (Scheme 5, **5i**, **5j**, **5p**, **5s**). It is noteworthy that common functional groups such as halides, esters, ketones, and nitro and cyano groups were compatible (Scheme 5, **5i–s**). Nevertheless, the reactions of aryl bromides occurred in low yields under the current reaction conditions and further optimization of the conditions are needed.

**Scheme 6:**
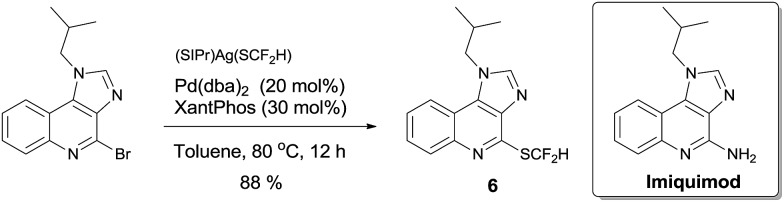
Preparation of the difluoromethylthiolated analog of Imiquimod

**Scheme 7:**
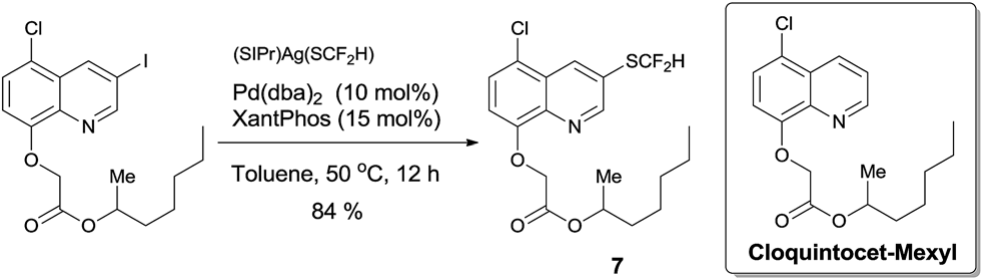
Preparation of the difluoromethylthiolated derivative of the herbicide safener Cloquintocet-mexyl

To demonstrate the applicability of this difluoromethylthiolating protocol, we studied the difluoromethylthiolation of two medicinally important compounds. Compound **6**, a difluoromethylthiolated analog of Imiquimod,^13^ a medication that acts as an immune response modifier to treat genital warts, was generated in an 88% yield (Scheme 6). Likewise, compound **7**, a difluoromethylthiolated derivative of the herbicide safener Cloquintocet-mexyl,^14^ was formed in an 84% yield under standard reaction conditions (Scheme 7).

## Conclusions

In summary, we have developed the first palladium-catalyzed direct difluoromethylthiolation of heteroaryl bromides, iodides, triflates and aryl iodides. The reactions were conducted under mild reaction conditions and many common functional groups were tolerant. Thus, the current method provides an alternative and attractive strategy for the preparation of the difluoromethylthiolated heteroarenes. Detailed mechanistic studies, including on the isolation of the difluoromethylthiolated palladium intermediate, investigation of the reaction kinetics and expansion of the reaction scope to other common aryl halides/triflates are undergoing currently in our laboratory.

## Supplementary Material

Supplementary information

Crystal structure data
