# Supplementary material for: Palladium-catalyzed difluoromethylthiolation of heteroaryl bromides, iodides, triflates and aryl iodides
Source: Chem Sci. 2016 Feb 17;7(6):3757–62. doi: 10.1039/c6sc00082g (PMC6008600; doi:10.1039/c6sc00082g)
Supplement: Supplementary file 1 [file SC-007-C6SC00082G-s001.pdf]

Electronic Supplementary Material (ESI) for Chemical Science.
This journal is © The Royal Society of Chemistry 2016

Supporting Information

Table of Contents

Supporting Information **1**

General information **2**

Preparation of [(SIPr)Ag(CF_2H)][1] **3**

Preparation of [(SIPr)Ag(SCF_2H)][2] **3**

Synthesis of Starting Materials **4**

Stoichiometric reaction of complex [(Xantphos)Pd(3-Py)(I)] with [(SIPr)Ag(SCF_2H)]. **15**

General Procedure for Difluoromethylthiolation of Heteroaryl iodide **16**

General Procedure for Difluoromethylthiolation of Heteroaryl bromide. **36**

General Procedure for Difluoromethylthiolation of Heteroaryl triflates **44**

General Procedure for Difluoromethylthiolation of aryl iodide **49**

Procedure for Difluoromethylthiolation of 4-bromo-1-*iso*butyl-1*H*-imidazo[4,5-*c*] quinolone 6 .... **58**

Procedure for Difluoromethylthiolation of heptan-2-yl 2-((5-chloro-3-iodo quinolin-8-yl)oxy)acetate 7 .... **59**

Reference **60**

Spectrum of the Starting Material **61**

Spectrum of the Products **82**

X-ray structure of [(Xantphos)Pd(3-py)(Br)] **186**

General information

All solvents were purified by standard methods. ^{1}H NMR spectra were recorded on a 500 MHz, 400 MHz or 300 MHz spectrometer. ^{19}F NMR spectra were recorded on a 376 MHz or 282 MHz spectrometer. ^{13}C NMR spectra were recorded on a 400 MHz or 500 MHz spectrometer. ^{1}H NMR and ^{13}C NMR chemical shifts were determined relative to internal standard TMS at δ 0.0 and ^{19}F NMR chemical shifts were determined relative to $CFCl_3$ as inter standard. Chemical shifts (δ) are reported in ppm, and coupling constants (*J*) are in Hertz (Hz). The following abbreviations were used to explain the multiplicities: s = singlet, d = doublet, t = triplet, q = quartet, m = multiplet, br = broad. Flash column chromatograph was carried out using 300-400 mesh silica gel at medium pressure. Detection of melting point was conducted on the SGW X-4 microscopic melting point meter. Elemental analysis was conducted on the VARIO EL III. X-ray structure was obtained on BRUKER SMART APEX CCD.

All reagents were received from commercial sources (Adamas-beta®, Alfa, TCI). Solvents were freshly dried and degassed according to the purification handbook *Purification of Laboratory Chemicals* before using.

Preparation of [(SIPr)Ag(CF_2H)][1]

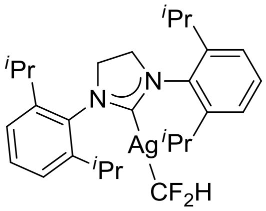

To a solution of [(SIPr)AgCl] (831 mg, 1.50 mmol) and NaO^{t}Bu (285 mg, 3.00 mmol) in THF (30 mL) was added TMSCF$_2$H (375 uL, 3.00 mmol). The resulting mixture was stirred for 1.5 h at ambient temperature. The mixture was filtered through a short plug with Celite and the solvent was evaporated under vacuum to give an off-white solid. The solid was recrystallized from CH_2Cl_2/pentane to give (1,3-bis(2,6-diisopropylphenyl)imidazolidin-2-yl) (difluoromethyl)silver [(SIPr)Ag(CF_2H)] as a white solid (698 mg, 82%). ^{1}H NMR (400 MHz, THF-d$_8$) δ 7.36 (t, J = 8.0 Hz, 2 H), 7.26 (d, J = 8.0 Hz, 4 H), 5.90 (td, J = 43.6, 14.0 Hz, 1 H), 4.04 (s, 4 H), 3.15 (hept, J = 6.8 Hz, 4 H), 1.34 (d, J = 7.2 Hz, 12 H), 1.32 (d, J = 7.2 Hz, 12 H); ^{19}F NMR (376 MHz,THF-d$_8$) δ -113.66 (dd, $J^{109}{}_{Ag\text{-}F}$ = 62.4 Hz, $J^{107}{}_{Ag\text{-}F}$ = 54.5 Hz, $J_{H\text{-}F}$ = 43.6 Hz); ^{13}C NMR (101 MHz, CDCl$_3$) δ 24.11, 25.76, 28.96, 124.55, 129.78, 134.80, 146.76, 153.67 (dt, $J^{109}{}_{Ag\text{-}C}$ = 260.6Hz, $J^{107}{}_{Ag\text{-}C}$ = 225.2 Hz, $J_{C\text{-}F}$ = 280.8 Hz), 211.41 (d, $J^{109}{}_{Ag\text{-}C}$ = 151.5 Hz, $J^{109}{}_{Ag\text{-}C}$ = 131.3 Hz) ppm. Anal. Calcd for $C_{28}H_{39}AgF_2N_2$: C, 61.20; H, 7.15; N, 5.10; Found: C, 60.91; H, 6.87; N, 4.78.

Preparation of [(SIPr)Ag(SCF_2H)][2]

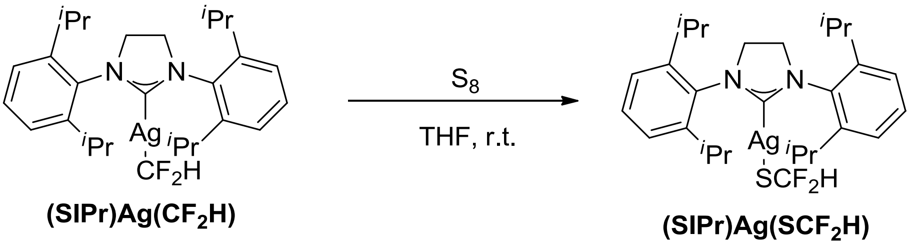

In a 250 mL round-bottom flask, a solution of [(SIPr)Ag(CF_2H)] (5.6 g, 10 mmol, 1.0 equiv) and S_8 (1.92 g, 60 mmol, 6.0 equiv) in THF (200 mL) was stirred for 50 min under an argon atmosphere at ambient temperature. The mixture was filtered through a short plug with Celite and the solvent was evaporated under vacuum to give an greyish-green solid. The solid was recrystallized twice from THF/pentane to give

(1,3-bis(2,6-diisopropylphenyl)imidazolidin-2-yl)(difluoromethylthio)silver [(SIPr)Ag(SCF_2H)] as a off-white solid (4.74 g, 82%).

(1,3-Bis(2,6-diisopropylphenyl)imidazolidin-2-yl) (difluoromethylthio) silver 1

^{1}H NMR (400 MHz, d^8-THF) δ 7.56 (dd, *J* = 8.3, 7.1 Hz, 2 H), 7.50 – 7.42 (m, 4 H), 6.74 (t, *J* = 65.2 Hz, 1 H), 4.31 (s, 4 H), 3.36 (hept, *J* = 6.9 Hz, 4 H), 1.52 (t, *J* = 6.6 Hz, 24 H); ^{19}F NMR (376 MHz, d^8-THF) δ -59.40 (d, *J* = 65.2 Hz, 2 F); ^{13}C NMR (101 MHz, $CDCl_3$) δ 210.49 – 207.67 (m), 146.65, 134.47, 129.92, 124.60, 124.20 (t, *J* = 269.4 Hz), 53.89, 53.81, 28.89, 25.31, 24.09 ppm. Anal. Calcd. for $C_{28}H_{39}AgF_2N_2S$: C, 57.83; H, 6.76; N, 4.82; Found: C, 57.43; H, 6.94; N, 4.54.

Synthesis of Starting Materials

Preparation of (Xantphos)Pd(3-Py)(I) 3

$Pd(dba)_2$ (574 mg, 1.0 mmol) and Xantphos (578 mg, 1.0 mmol) were placed in a 100 mL flask. Toluene (40 mL) and 3-iodopyridine (2.04 g, 10.0 mmol) were added, and the mixture was stirred at room temperature for 4 h. The solution was filtered through Celite. The filtrate was evaporated in vacuum to a volume of 5.0 mL. Addition of pentane (150 mL) gave a yellow precipitate. The precipitate was filtered, and the solid was recrystallized from THF/pentane (510.0 mg, 57%) to give (Xantphos)Pd(3-py)(I) **3**.

(Xantphos)Pd(3-Py)(I). ^{1}H NMR (400 MHz, $CDCl_3$) δ 7.80 (s, 1 H), 7.61 (d, *J* = 7.6 Hz, 2 H), 7.45 – 7.13 (m, 25 H), 6.56 (s, 1 H), 5.93 (s, 1 H), 1.78 (s, 6 H); ^{31}P NMR (162 MHz, $CDCl_3$) δ 10.64 (s, 2 P) ppm. Anal. Calcd. for $C_{44}H_{36}INOP_2Pd$: C, 59.38; H, 4.08; N, 1.57; Found: C, 59.44; H, 4.23; N, 1.67.

Preparation of (Xantphos)Pd(3-Py)(Br)

$Pd(dba)_2$ (574 mg, 1.0 mmol) and Xantphos (578 mg, 1.0 mmol) were placed in a 100 mL flask. Toluene (40 mL) and 3-bromopyridine (1.56 g, 10.0 mmol) were added, and the mixture was stirred at 60 ^oC for 4 h. The solution was filtered through Celite. The filtrate was evaporated in vacuum to a volume of 5.0 mL. Addition of pentane (150 mL) gave a pale yellow precipitate (592 mg, 70%).

(Xantphos)Pd(3-Py)(Br). ^{1}H NMR (400 MHz, $CDCl_3$) δ 7.88 (s, 1 H), 7.60 (d, *J* = 5.2 Hz, 3 H), 7.44 – 7.12 (m, 24 H), 6.58 (s, 1 H), 5.88 (s, 1 H), 1.76 (s, 6 H); ^{31}P NMR (162 MHz, $CDCl_3$) δ 9.20 (s, 2 P) ppm. Anal. Calcd. for $C_{44}H_{36}BrNOP_2Pd$: C, 62.69; H, 4.30; N, 1.66; Found: C, 62.47; H, 4.56; N, 1.75.

Preparation of 6-Iodobenzo[d]thiazole[3] 3ak

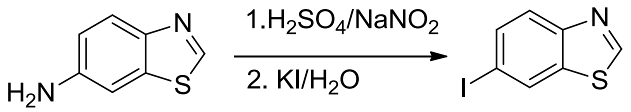

A mixture of concentrated H_2SO_4 (20 mL) and $NaNO_2$ (1.3 g, 18.5 mmol) was stirred at 70 °C for 15 min. After cooling to 40 °C, a solution of 6-aminobenzothiazole (6) (2.5 g, 16.6 mmol) in acetic acid (35.0 mL) was added dropwise. The reaction mixture was stirred at room temperature for 30 min. The mixture was then added to the stirred solution of KI (3.32 g, 20 mmol) in water (35 mL). The resulting mixture was stirred at 70 °C for 30 min and then poured onto ice. The precipitate was collected by filtration, washed with water, and dissolved in $CHCl_3$ (50 mL). The organic solution was washed with 10% aqueous solution of $Na_2S_2O_3$ (2 × 30 mL), dried over Na_2SO_4. The solvent was evaporated, and the crude product was purified by column chromatography on silica gel to yield 6-iodobenzo[*d*]thiazole as a white solid (1.6 g, 37 %).

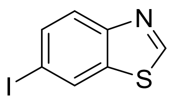

6-Iodobenzo[d]thiazole 3ak. ^{1}H NMR (400 MHz, $CDCl_3$) δ 8.90 (s, 1 H), 8.28 (d, *J* = 0.9 Hz, 1 H), 7.86 (d, *J* = 8.6 Hz, 1 H), 7.78 (dd, *J* = 8.6, 1.6 Hz, 1 H); ^{13}C NMR (101 MHz, $CDCl_3$) δ 154.32, 152.61, 135.87, 135.29, 130.40, 125.07, 90.21 ppm.

Preparation of 5-Iodobenzofuran[3] 3y

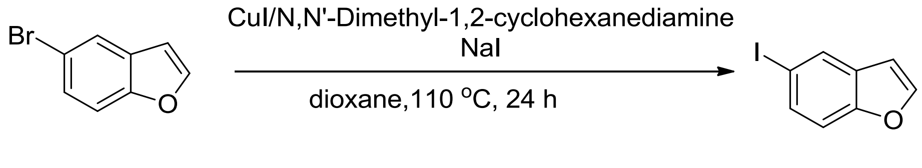

A Schlenk tube was charged with CuI (48.0 mg, 0.25 mmol, 5.0 mol%), NaI (1.5 g,

10.0 mmol), briefly evacuated and backfilled with argon. Racemic *trans*-N,N'-dimethyl-1,2-cyclohexanediamine (80.0 μL, 1.00 mmol, 10 mol%), 5-bromobenzofuran (0.98 g, 5.00 mmol), and dioxane (5.0 mL) were added under argon. The Schlenk tube was sealed with a Teflon valve and the reaction mixture was stirred at 110 ℃ for 24 h. The resulting suspension was allowed to reach room temperature, diluted with 30% aq ammonia (25 mL), poured into water (100 mL), and extracted with dichloromethane (3×15 mL). The combined organic phases were dried ($MgSO_4$ or Na_2SO_4), concentrated, and the residue was purified by flash chromatography on silica gel (eluent: hexane / EtOAc = 10:1) to provide the desired product as a colorless liquid (1.17 g, 96 %).

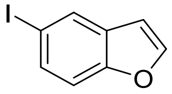

5-Iodobenzofuran 3y. ^{1}H NMR (400 MHz, $CDCl_3$) δ 7.92 (s, 1 H), 7.55 (d, *J* = 12.5 Hz, 2 H), 7.27 (d, *J* = 8.6 Hz, 1 H), 6.69 (s, 1 H); ^{13}C NMR (101 MHz, $CDCl_3$) δ 154.33, 145.78, 132.85, 130.16, 130.06, 113.43, 105.83, 86.26 ppm.

Preparation of *tert*-butyl 5-iodo-1*H*-indazole-1-carboxylate[3] 3ai

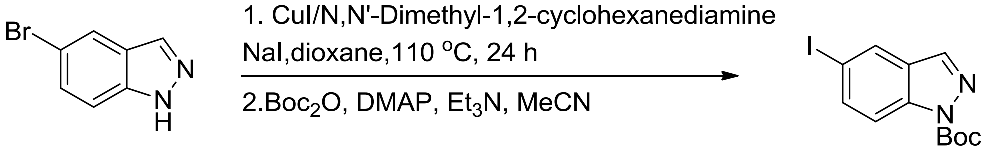

A Schlenk tube was charged with CuI (48.0 mg, 0.25 mmol, 5.0 mol%), NaI (1.5 g, 10.0 mmol), briefly evacuated and backfilled with argon. Racemic trans-N,N'-dimethyl-1,2-cyclohexanediamine (80.0 μL, 1.00 mmol, 10 mol%), 5-bromo-1*H*-indazole (0.98 g, 5.00 mmol), and dioxane (5.0 mL) were added under argon. The Schlenk tube was sealed with a Teflon valve and the reaction mixture was stirred at 110 ℃ for 24 h. The resulting suspension was allowed to reach room temperature, diluted with 30% aq ammonia (25 mL), poured into water (100 mL), and extracted with dichloromethane (3×15 mL). The combined organic phases were dried ($MgSO_4$ or Na_2SO_4), concentrated, and the residue was used directly without further

purification.

In a 100 mL round-bottom flask, Boc_2O (1.72 g, 7.6 mmol), DMAP (0.12 g, 1 mmol) was dissolved in MeCN (15 mL). Et_3N (0.70 mL, 5 mmol) was added and the mixture was stirred at room temperature for 3 h. The mixture was then concentrated in vacuo. The resulting residue was purified by flash chromatography (eluent: hexane / EtOAc = 30:1) to give the *tert*-butyl 5-iodo-1*H*-indazole-1-carboxylate as colorless oil (1.3 g, 76 %).

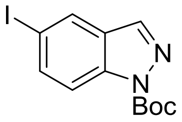

***tert*-Butyl 5-iodo-1*H*-indazole-1-carboxylate 3ai.** ^{1}H NMR (400 MHz, $CDCl_3$) δ 8.07 (s, 2 H), 7.96-7.94 (m, 1 H), 7.75 (dd, *J* = 8.8, 1.7 Hz, 1 H), 1.71 (s, 9 H); ^{13}C NMR (101 MHz, $CDCl_3$) δ 148.90, 138.99, 138.15, 137.30, 129.96, 128.05, 116.33, 87.34, 85.36, 28.15 ppm.

Preparation of 8-(benzyloxy)-5,7-dichloroquinoline[5,6] 3u

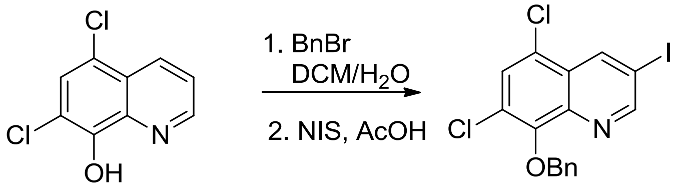

In a 500 mL round-bottom flask, the 5,7-dichloroquinolin-8-ol (8.5 g, 40 mmol), sodium hydroxide (2.4 g, 60 mmol) and *tetra*-butylammonium bromide (332 mg, 1.0 mmol) were dissolved in a mixture of dichloromethane (100 mL) and water (100 mL). Benzyl bromide (13.6 g, 80 mmol) was added dropwise to the solution. The mixture was stirred at room temperature for 4 h. The organic layer was separated and the aqueous layer extracted with dichloromethane (2 × 100 mL). The organic phase was combined and dried over anhydrous Na_2SO_4, and then concentrated in vacuo. The resulting residue was purified by flash chromatography (eluent: hexane / EtOAc = 20:1) to give the 8-(benzyloxy)-5,7- dichloroquinoline as a white solid (9.5 g, 78 %).

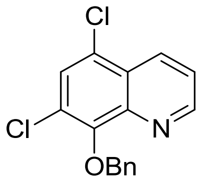

8-(Benzyloxy)-5,7-dichloroquinoline 2u. ^{1}H NMR (400 MHz, $CDCl_3$) δ 9.02 (d, *J* = 4.1 Hz, 1 H), 8.52 (d, *J* = 8.6 Hz, 1 H), 7.64 (s, 1 H), 7.60 (d, *J* = 7.3 Hz, 2 H), 7.54 (dd, *J* = 8.5, 4.1 Hz, 1 H), 7.39-7.31 (m, 3 H), 5.47 (s, 2 H); ^{13}C NMR (101 MHz, $CDCl_3$) δ 150.77, 150.34, 143.90, 137.03, 133.33, 128.65, 128.35, 128.21, 127.88, 127.02, 126.28, 126.21, 122.02, 76.67 ppm. MS (EI): 91.1 (100), 303. HRMS (EI) for $C_{16}H_{11}Cl_2NO$ Calcd: 303.0218; Found: 303.0223. IR (KBr): ν = 3085, 3064, 3030, 2956, 1600, 1580, 1496, 1483, 1456, 1446, 1369, 1349, 1284, 1238, 1215, 1138, 1095, 1041, 990, 945, 883, 877, 807 cm^{-1}. Mp: 87.9 – 89.2 ^{o}C.

N-Iodo-succinimide (2.25 g, 10 mmol) was added in portions to a stirred solution of 8-(benzyloxy)-5,7-dichloroquinoline (3.0 g, 10.0 mmol) in acetic acid (10 mL) at 70 ℃ under argon. The mixture was heated to 70 ℃ for 18 h. After cooling to room temperature, the mixture was concentrated in vacuo. The residue was redissolved in dichloromethane (50 mL) and the solution was washed successively with 10 % aqueous sodium thiosulfate solution (2 × 30 mL) and 10 % aqueous sodium hydrogen carbonate solution (2 × 30 mL), dried ($NaSO_4$) and then concentrated in vacuo. The resulting residue was purified by flash chromatography (eluent: hexane / EtOAc = 20:1) to give the 8-(benzyloxy)-5,7-dichloro-3- iodoquinoline as a white solid (1.4 g, 33 %).

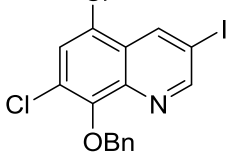

8-(Benzyloxy)-5,7-dichloro-3-iodoquinoline 3u. ^{1}H NMR (400 MHz, $CDCl_3$) δ 9.13 (d, *J* = 1.9 Hz, 1 H), 8.88 (d, *J* = 2.0 Hz, 1 H), 7.63 (s, 1 H), 7.57 (d, *J* = 6.6 Hz, 2 H), 7.39-7.33 (m, 3 H), 5.44 (s, 2 H); ^{13}C NMR (101 MHz, $CDCl_3$) δ 156.06, 150.53,

142.00, 141.08, 136.75, 128.84, 128.62, 128.39, 128.32, 127.68, 127.55, 124.95, 91.59, 77.82 ppm. MS (EI): 91.1 (100), 429. HRMS (EI) for $C_{16}H_{10}Cl_2INO$ Calcd: 428.9184, Found: 428.9181. IR (KBr): ν = 3072, 3028, 2949, 1569, 1455, 1438, 1384, 1350, 1280, 1242, 1206, 1101, 1072, 940, 890, 762, 729, 693, 623 cm^{-1}. Mp: 130.8 – 132.4 °C.

Preparation of 2-((5-chloro-3-iodoquinolin-8-yl)oxy)acetate[6] 3ay.

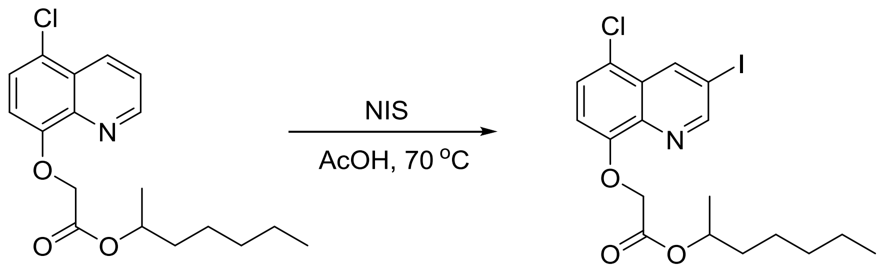

N-Iodo-succinimide (4.5 g, 20 mmol) was added in portions to a stirred solution of heptan-2-yl 2-((5-chloroquinolin-8-yl)oxy)acetate (6.7 g, 20 mmol) in acetic acid (20 mL) at 70 °C under argon. The mixture was heated to 70 °C for 18 h. After cooling to room temperature, the mixture was concentrated in vacuo. The residue was redissolved in dichloromethane (100 mL) and the solution was washed successively with 10 % aqueous sodium thiosulfate solution (2 × 60 mL) and 10 % aqueous sodium hydrogen carbonate solution (2 × 60 mL), dried ($NaSO_4$) and then concentrated in vacuo. The resulting residue was purified by flash chromatography (eluent: hexane / EtOAc = 20:1) to give the heptan-2-yl 2-((5-chloro-3-iodoquinolin-8-yl)oxy)acetate as a white solid (1.02 g, 11 %).

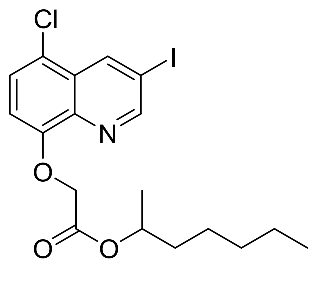

2-((5-Chloro-3-iodoquinolin-8-yl)oxy)acetate 3ay. ^{1}H NMR (400 MHz, $CDCl_3$) δ 9.09 (s, 1 H), 8.89 (s, 1 H), 7.49 (d, *J* = 8.4 Hz, 1 H), 6.89 (d, *J* = 8.4 Hz, 1 H), 5.07-4.96 (m, 1 H), 4.92 (s, 2 H), 1.56-1.42 (m, 2 H), 1.28-1.18 (m, 9 H), 0.87-0.82 (m, 3

H); ^{13}C NMR (101 MHz, $CDCl_3$) δ 167.91, 155.19, 153.11, 140.85, 138.76, 128.60, 127.06, 122.17, 109.95, 92.24, 72.83, 66.39, 35.65, 31.46, 24.88, 22.47, 19.86, 13.93 ppm. MS (EI): 318 (100), 461. HRMS (EI) for $C_{18}H_{21}ClINO_3$ Calcd: 461.0255; Found: 461.0253. IR (KBr): ν = 2930, 1743, 1605, 1557, 1446, 1364, 1338, 1314, 1217, 1158, 1119, 947, 892, 834, 720 cm^{-1}. Mp: 66.8 – 68.2 ^{o}C.

Preparation of 4-bromo-1-*iso*butyl-1*H*-imidazo[4,5-c]quinolone[7] 3ax

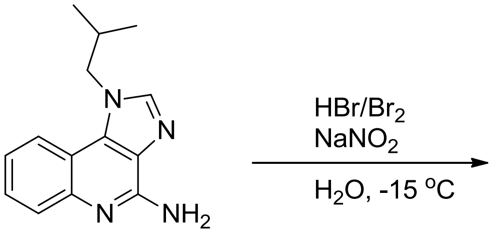

1-*iso*Butyl-1*H*-imidazo[4,5-c]quinolin-4-amine (932 mg, 3.88 mmol) was suspended in hydrobromic acid (5.0 mL, 48% in water), and the mixture was cooled to −15 ℃. Bromine (1.2 mL, 23.5 mmol) was added dropwise to the mixture followed by addition of sodium nitrite (1.5 g, 21.7 mmol) in water (2 mL). The reaction mixture was warmed to room temperature and was further stirred for 3 h. The reaction mixture was cooled to −15 ℃ and quenched with aqueous solution of potassium hydroxiode. The mixture was extracted with ethyl acetate (3 × 30 mL). The combined extracts were washed with water (2 × 20 mL), saturated aqueous sodium bicarbonate (20 mL), dried with sodium sulfate, and concentrated in vacuo. The resulting residue was purified by flash chromatography (eluent: hexane / EtOAc = 10:1) to give the 4-bromo-1-*iso*butyl-1*H*-imidazo[4,5-c]quinoline as a white solid (732 mg, 66 %).

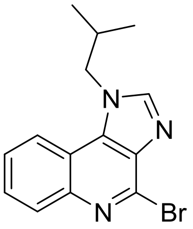

4-Bromo-1-*iso*butyl-1*H*-imidazo[4,5-c]quinolone 3ax. ^{1}H NMR (400 MHz, $CDCl_3$) δ 8.22 (d, *J* = 7.8 Hz, 1 H), 8.08 (d, *J* = 7.7 Hz, 1 H), 7.94 (s, 1 H), 7.71-7.64 (m, 2 H), 4.35 (d, *J* = 7.4 Hz, 2 H), 2.40-2.29 (m, 1 H), 1.04 (d, *J* = 6.6 Hz, 6 H). ^{13}C NMR (126

MHz, $CDCl_3$) δ 144.84, 143.94, 136.48, 132.93, 130.32, 127.97, 127.00, 120.16, 117.81, 109.99, 55.41, 28.83, 19.78 ppm. MS (EI): 303 (100), 305 (100). HRMS (EI) for $C_{14}H_{14}BrN_3$ Calcd: 303.0371; Found: 303.0377. IR (KBr): ν = 3092, 2962, 1740, 1561, 1390, 1354, 1218, 1137, 1077, 1014, 915, 773 cm^{-1}. Mp: 176.2 – 177.8 ^{o}C.

Preparation of heteroaryl triflates[8]

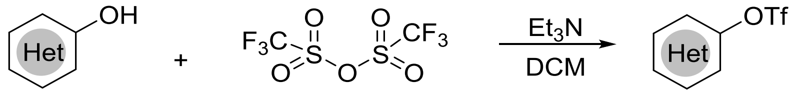

To a stirred solution of starting material (10.0 mmol) and Et_3N (1.8 mL, 13.0 mmol) in CH_2Cl_2 (20.0 mL) at ^{o}C was added Tf_2O (2.0 mL, 12.0 mmol) dropwise over 3 min. The mixture was stirred for 2 h then concentrated *in vacuo* and the residue purified by flash chromatography to provide heteroaryl triflates.

5-Bromopyridin-2-yl trifluoromethanesulfonate 3a

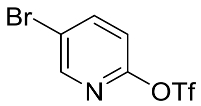

Prepared from 5-bromopyridin-2-ol (1.73 g, 10.0 mmol) according to general procedure. The crude residue was purified by flash column chromatography on silica gel (eluent: hexane / EtOAc = 10:1) to yield **5-bromopyridin-2-yl trifluoromethanesulfonate** (1.5 g, 49 %) as a colorless oil. ^{1}H NMR (400 MHz, $CDCl_3$) δ 8.45 (d, *J* = 2.4 Hz, 1 H), 8.01-7.98 (m, 1 H), 7.10 (dd, *J* = 8.6, 0.6 Hz, 1 H); ^{13}C NMR (101 MHz, $CDCl_3$) δ 154.50, 149.72, 143.49, 120.51, 118.56 (q, *J* = 320.6 Hz), 116.75 ppm.

5-Chloropyridin-2-yl trifluoromethanesulfonate 3b

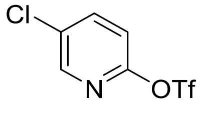

Prepared from 5-chloropyridin-2-ol (1.29 g, 10.0 mmol) according to general procedure. The crude residue was purified by flash column chromatography on silica gel (eluent: hexane / EtOAc = 10:1) to yield **5-chloropyridin-2-yl trifluoromethanesulfonate** (1.3 g, 50 %) as a pale yellow oil. ^{1}H NMR (400 MHz,

$CDCl_3$) δ 8.35 (d, *J* = 2.6 Hz, 1 H), 7.86 (dd, *J* = 8.6, 2.7 Hz, 1 H), 7.16 (d, *J* = 8.6 Hz, 1 H); ^{19}F NMR (376 MHz, $CDCl_3$) δ -72.97 (s, 3 F); ^{13}C NMR (101 MHz, $CDCl_3$) δ 153.89, 147.42, 140.60, 132.44, 118.58 (q, *J* = 320.6 Hz), 116.29 ppm.

Isoquinolin-1-yl trifluoromethanesulfonate 3v

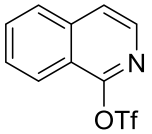

Prepared from isoquinolin-1-ol (1.45 g, 10.0 mmol) according to general procedure. The crude residue was purified by flash column chromatography on silica gel (eluent: hexane / EtOAc = 30:1) to yield **isoquinolin-1-yl trifluoromethanesulfonate** (1.36 g, 49 %) as a colorless oil. ^{1}H NMR (400 MHz, $CDCl_3$) δ 8.18 (dd, *J* = 5.7, 2.4 Hz, 1 H), 8.10 (d, *J* = 8.4 Hz, 1 H), 7.89 (d, *J* = 8.3 Hz, 1 H), 7.81-7.77 (m, 1 H), 7.73-7.67 (m, 2 H); ^{19}F NMR (376 MHz, $CDCl_3$) δ -73.09 (s, 3 F); ^{13}C NMR (101 MHz, $CDCl_3$) δ 152.94, 139.43, 139.42, 131.97, 129.18, 126.85, 122.81, 122.03, 119.91, 118.68 (q, *J* = 320.5 Hz) ppm.

5-Bromopyrimidin-2-yl trifluoromethanesulfonate 3ac

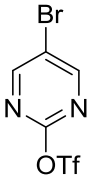

Prepared from 5-bromopyrimidin-2-ol (1.74 g, 10.0 mmol) according to general procedure. The crude residue was purified by flash column chromatography on silica gel (eluent: hexane / EtOAc = 20:1) to yield **5-bromopyrimidin-2-yl trifluoromethanesulfonate** (0.5 g, 16 %) as a pale yellow oil. ^{1}H NMR (400 MHz, $CDCl_3$) δ 8.80 (s, 2 H); ^{19}F NMR (376 MHz, $CDCl_3$) δ -73.01 – -73.07 (m, 3 F); ^{13}C NMR (101 MHz, $CDCl_3$) δ 161.26, 157.02, 119.50, 118.45(q, *J* = 320.8 Hz) ppm. MS (EI): 163 (100), 306, 308. HRMS (EI) for $C_5H_2BrF_3N_2O_3S$ Calcd: 305.8922; Found: 305.8917. IR (KBr): ν = 1561, 1429, 1405, 1216, 1135, 1021, 908, 807, 763, 610 cm^{-1}.

Quinolin-2-yl trifluoromethanesulfonate 3ap

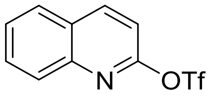

Prepared from quinolin-2-ol (1.45 g, 10.0 mmol) according to general procedure. The crude residue was purified by flash column chromatography on silica gel (eluent: hexane / EtOAc = 20:1) to yield **quinolin-2-yl trifluoromethanesulfonate** (1.1 g, 40 %) as a pink oil. ^{1}H NMR (400 MHz, $CDCl_3$) δ 8.29 (dd, *J* = 8.7, 3.4 Hz, 1 H), 8.06-7.93 (m, 1 H), 7.85 (d, *J* = 8.1 Hz, 1 H), 7.78-7.74 (m, 1 H), 7.62-7.57 (m, 1 H), 7.20 (dd, *J* = 8.7, 2.4 Hz, 1 H); ^{19}F NMR (376 MHz, $CDCl_3$) δ -73.07 (s, 3 F); ^{13}C NMR (101 MHz, $CDCl_3$) δ 153.70, 145.88, 141.89, 131.23, 128.89, 127.79, 127.73, 127.64, 118.73 (q, *J* = 320.5 Hz), 113.13 ppm.

Quinoxalin-2-yl trifluoromethanesulfonate 3at

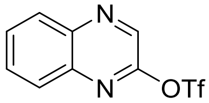

Prepared from quinoxalin-2-ol (1.46 g, 10.0 mmol) according to general procedure. The crude residue was purified by flash column chromatography on silica gel (eluent: hexane / EtOAc = 20:1) to yield **quinoxalin-2-yl trifluoromethanesulfonate** (2.3 g, 83 %) as a pale yellow oil. ^{1}H NMR (400 MHz, $CDCl_3$) δ 8.78 (s, 1 H), 8.21-8.17 (m, 1 H), 8.07-8.03 (m, 1 H), 7.88-7.83 (m, 2 H); ^{19}F NMR (376 MHz, $CDCl_3$) δ -72.74 (s, 3 F); ^{13}C NMR (101 MHz, $CDCl_3$) δ 149.54, 142.20, 139.62, 137.86, 131.99, 131.02, 129.35, 128.86, 118.63 (q, *J* = 320.8 Hz) ppm.

5-Bromo-3-nitropyridin-2-yl trifluoromethanesulfonate 3av

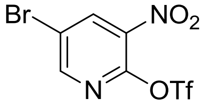

Prepared from 5-bromo-3-nitropyridin-2-ol (2.18 g, 10.0 mmol) according to general procedure. The crude residue was purified by flash column chromatography on silica gel (eluent: hexane / EtOAc = 10:1) to yield **5-bromo-3-nitropyridin-2-yl trifluoromethanesulfonate** (1.1 g, 31 %) as a yellow oil. ^{1}H NMR (400 MHz, $CDCl_3$)

δ 8.70 (d, *J* = 2.3 Hz, 1 H), 8.69 (d, *J* = 2.3 Hz, 1 H); ^{19}F NMR (376 MHz, $CDCl_3$) δ -72.81 (s, 3 F); ^{13}C NMR (101 MHz, $CDCl_3$) δ 153.45, 145.87, 138.93, 136.26, 120.33, 118.35 (q, *J* = 321.1 Hz) ppm. MS (EI): 174 (100), 350, 352. HRMS (EI) for $C_6H_2BrF_3N_2O_5S$ Calcd: 349.8820; Found: 349.8816. IR (KBr): ν = 3082, 1363, 1587, 1541, 1435, 1342, 1219, 1133, 910, 855, 792, 760, 610 cm^{-1}.

7-Bromoisoquinolin-1-yl trifluoromethanesulfonate 3aw

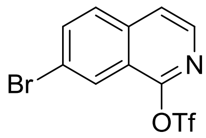

Prepared from 7-bromoisoquinolin-1-ol (2.23 g, 10.0 mmol) according to general procedure. The crude residue was purified by flash column chromatography on silica gel (eluent: hexane / EtOAc = 10:1) to yield **7-bromoisoquinolin-1-yl trifluoromethanesulfonate** (2.0 g, 56 %) as a white solid. ^{1}H NMR (400 MHz, $CDCl_3$) δ 8.27-8.21 (m, 2 H), 7.91 – 7.86 (m, 1 H), 7.81-7.77 (m, 1 H), 7.68 (t, *J* = 5.1 Hz, 1 H); ^{19}F NMR (376 MHz, $CDCl_3$) δ -72.72 (s, 3 F); ^{13}C NMR (101 MHz, $CDCl_3$) δ 151.75, 139.89, 137.79, 135.64, 128.44, 125.11, 123.24, 121.71, 120.70, 118.64 (q, *J* = 320.7 Hz) ppm. MS (EI): 196 (100), 355, 357. HRMS (EI) for $C_{10}H_5BrF_3NO_3S$ Calcd: 354.9126; Found: 354.9124. IR (KBr): ν = 3074, 1633, 1584, 1409, 1311, 1182, 1153, 1077, 1038, 895, 853, 802, 763, 666 cm^{-1}. Mp: 107.2 – 109.4 ^{o}C.

Stoichiometric reaction of complex [(Xantphos)Pd(3-Py)(I)] with [(SIPr)Ag(SCF$_2$H)].

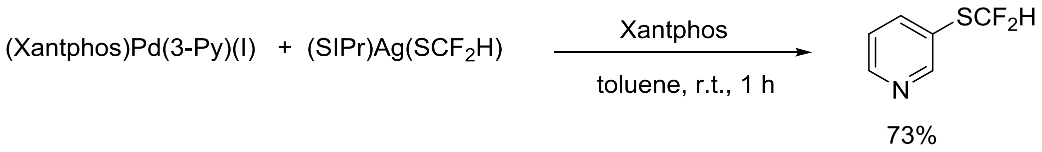

[(Xantphos)Pd(3-Py)(I)] (17.8 mg, 0.02 mmol, 1.0 equiv), Xantphos (11.6 mg, 0.02 mmol, 1.0 equiv) and [(SIPr)Ag(SCF$_2$H)] (17.4 mg, 0.03 mmol, 1.5 equiv) were added in a 20 mL schlenk tube under argon. To the tube was added 1.0 mL of anhydrous toluene and the mixture was stirred at room temperature for 1 h. Yields were determined by ^{19}F NMR analysis of the crude reaction mixture with trifluorotoluene as an internal standard.

General Procedure for Difluoromethylthiolation of Heteroaryl iodide

Method A

Heteroaryl iodide (0.5 mmol, 1.0 equiv), $Pd(dba)_2$ (30.0 mg, 10.0 mol%), XantPhos (44.0 mg, 15.0 mol%), and [(SIPr)Ag(SCF_2H)] (350.0 mg, 0.6 mmol, 1.2 equiv) were added in a 20 mL schlenk tube under argon. To the tube was added 2.5 mL of anhydrous toluene and the mixture was stirred at 50 ^oC for 12 h. The dark solution was diluted with Et_2O (15.0 mL). The mixture was filtered through a short plug of silica gel, washed with Et_2O (100 mL). The organic layer was combined, and concentrated under vacuum. The crude product was purified by column chromatography on silica gel with a mixed solvent of pentane/Et_2O or pentane/EtOAc as the eluent to give the product.

Method B

Hetary iodide (0.5 mmol, 1.0 equiv), XantphosPd(3-py)(Br) (21.0 mg, 5.0 mol%), XantPhos (7.5 mg, 2.5 mol%), and [(SIPr)Ag(SCF_2H)] (350.0 mg, 0.6 mmol, 1.2 equiv) were added in a 20 mL schlenk tube under argon. To the tube was added 5.0 mL of anhydrous toluene and the mixture was stirred at 50 ^oC for 6 h. The dark solution was diluted with Et_2O (15.0 mL). The mixture was filtered through a short plug of silica gel, washed with Et_2O (100 mL). The organic layer was combined, and concentrated under vacuum. The crude product was purified by column chromatography on silica gel with a mixed solvent of pentane/Et_2O or pentane/EtOAc as the eluent to give the product.

5-Bromo-2-((difluoromethyl)thio)pyridine 4a

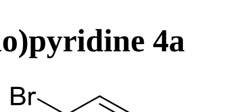

Prepared from 5-bromo-2-iodopyridine (141.5 mg, 0.5 mmol) according to general procedure **A**. The crude residue was purified by flash column chromatography on silica gel to yield **5-bromo-2-((difluoromethyl)thio)pyridine 4a** (66 mg, 55 %) as a colorless oil. ^1H NMR (400 MHz, $CDCl_3$) δ 8.56 (d, J = 2.1 Hz, 1 H), 7.73 (dd, J =

8.4, 2.4 Hz, 1 H), 7.63 (t, J = 56.2 Hz, 1 H), 7.17 (dd, J = 8.4, 0.5 Hz, 1 H); ^{19}F NMR (376 MHz, $CDCl_3$) δ -96.31 (d, J = 56.1 Hz, 2 F); ^{13}C NMR (101 MHz, $CDCl_3$) δ 151.81 (t, J = 3.7 Hz), 151.09, 139.73, 125.34 (t, J = 2.3 Hz), 120.83 (t, J = 272.0 Hz), 118.80 ppm. MS (EI): 191 (100), 239, 241. HRMS (EI) for $C_6H_4BrF_2NS$ Calcd: 238.9216; Found: 238.9214. IR (KBr): ν = 2926, 1560, 1545.7, 1452, 1352, 1285, 1119, 1069, 1004, 823, 791, 750, 627, 483 cm^{-1}.

5-Chloro-2-((difluoromethyl)thio)pyridine 4b

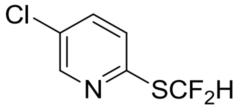

Prepared from 5-chloro-2-iodopyridine (119.5 mg, 0.5 mmol) according to general procedure **A**. The crude residue was purified by flash column chromatography on silica gel to yield **5-chloro-2-((difluoromethyl)thio)pyridine 4b** (84 mg, 86 %) as a colorless oil. ^{1}H NMR (400 MHz, $CDCl_3$) δ 8.46 (d, J = 2.3 Hz, 1 H), 7.62 (t, J = 56.2 Hz, 1 H), 7.60 (dd, J = 8.5, 2.6 Hz, 1 H), 7.23 (dd, J = 8.5, 0.5 Hz, 1 H); ^{19}F NMR (376 MHz, $CDCl_3$) δ -96.23 (d, J = 56.2 Hz, 2 F); ^{13}C NMR (101 MHz, $CDCl_3$) δ 151.17 (t, J = 4.0 Hz), 148.93, 136.97, 130.51, 124.97 (t, J = 2.3 Hz), 120.94 (t, J = 272.0 Hz) ppm. MS (EI): 145 (100), 195. HRMS (EI) for $C_6H_4ClF_2NS$ Calcd: 194.9721; Found: 194.9720. IR (KBr): ν = 3051, 2927, 2855, 1567, 1551, 1447, 1358, 1285, 1122, 1068, 1010, 825, 789, 765 cm^{-1}.

Methyl 6-((difluoromethyl)thio)nicotinate 4c

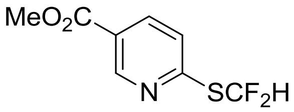

Prepared from methyl 6-iodonicotinate (131.5 mg, 0.5 mmol) according to general procedure **A**. The crude residue was purified by flash column chromatography on silica gel to yield **methyl 6-((difluoromethyl)thio)nicotinate 4c** (101 mg, 92 %) as a white solid. ^{1}H NMR (400 MHz, $CDCl_3$) δ 9.05 (d, J = 1.7 Hz, 1 H), 8.18 (dd, J = 8.3, 2.2 Hz, 1 H), 7.83 (t, J = 55.9 Hz, 1 H), 7.29 (d, J = 8.3 Hz, 1 H), 3.95 (s, 3 H); ^{19}F NMR (376 MHz, $CDCl_3$) δ -97.45 (d, J = 55.9 Hz, 2 F); ^{13}C NMR (101 MHz, $CDCl_3$)

δ 165.21, 158.87 (t, *J* = 3.7 Hz), 151.00, 137.66, 123.23, 122.60 (t, *J* = 2.4 Hz), 120.71 (t, *J* = 297.9 Hz), 52.48 ppm. MS (EI): 137 (100), 219. HRMS (EI) for $C_8H_7F_2NO_2S$ Calcd: 219.0166; Found: 219.0160. IR (KBr): ν = 3074, 2965, 1731, 1556, 1463, 1439, 1367, 1306, 1281, 1248, 847, 832, 785, 762, 582 cm^{-1}. Mp: 40.2 – 42.3 °C.

2-((Difluoromethyl)thio)-5-nitropyridine 4d

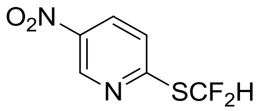

Prepared from 2-iodo-5-nitropyridine (125.0 mg, 0.5 mmol) according to general procedure **A**. The crude residue was purified by flash column chromatography on silica gel to yield **2-((difluoromethyl)thio)-5-nitropyridine 4d** (88 mg, 85 %) as a white solid.

Prepared from 2-iodo-5-nitropyridine (125.0 mg, 0.5 mmol) according to general procedure **B**. The crude residue was purified by flash column chromatography on silica gel to yield **2-((difluoromethyl)thio)-5-nitropyridine 4d** (102 mg, 99 %) as a white solid.

^{1}H NMR (400 MHz, $CDCl_3$) δ 9.28 (d, *J* = 2.6 Hz, 1 H), 8.38 (dd, *J* = 8.8, 2.7 Hz, 1 H), 7.83 (t, *J* = 55.6 Hz, 1 H), 7.39 (dd, *J* = 8.8, 0.5 Hz, 1 H); ^{19}F NMR (376 MHz, $CDCl_3$) δ -97.80 (d, *J* = 55.5 Hz, 2 F); ^{13}C NMR (101 MHz, $CDCl_3$) δ 161.67 (t, *J* = 3.9 Hz), 145.26, 142.36, 142.36, 131.75, 122.63 (t, *J* = 3.1 Hz), 119.95 (t, *J* = 272.1 Hz) ppm. MS (EI): 124.1 (100), 206. HRMS (EI) for $C_6H_4F_2NO_2S$ Calcd: 205.9962; Found: 205.9958. IR (KBr): ν = 3099, 2844, 1593, 1568, 1514, 1455, 1346, 1277, 1139, 1061, 1011, 857, 843, 788, 748 580, 526 cm^{-1}. Mp: 44.3 – 46.8 °C.

2-((Difluoromethyl)thio)isonicotinonitrile 4e

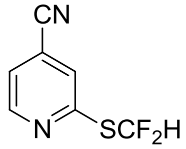

Prepared from 2-iodoisonicotinonitrile (115.0 mg, 0.5 mmol) according to general

procedure **A**. The crude residue was purified by flash column chromatography on silica gel to yield **2-((difluoromethyl)thio)isonicotinonitrile 4e** (86%, determined by ^{19}F NMR analysis) as a colorless oil. The compound was further purified by prep-HPLC with a C18 column (Waters, Prep Nova-pak® HR C18, 19×300 mm，6 μ m) using a water-acetonitrile mixed solvent as the eluent (CH_3CN : H_2O = 80 : 20, flow = 10.0 mL/min, 254 nm). ^{1}H NMR (400 MHz, $CDCl_3$) δ 8.65 (dd, *J* = 5.0, 0.7 Hz, 1 H), 7.71 (t, *J* = 55.8 Hz, 1 H), 7.47 (d, *J* = 1.0 Hz, 1 H), 7.36 (dd, *J* = 5.0, 1.3 Hz, 1 H); ^{19}F NMR (376 MHz, $CDCl_3$) δ -97.00 (d, *J* = 55.8 Hz, 2 F); ^{13}C NMR (101 MHz, $CDCl_3$) δ 155.83 (t, *J* = 4.0 Hz), 150.79, 125.06 (t, *J* = 2.5 Hz), 122.62, 121.56, 120.21 (t, *J* = 272.6 Hz), 115.54 ppm. MS (EI): 104.1 (100), 186. HRMS (EI) for $C_7H_4F_2N_2S$ Calcd: 186.006; Found: 186.0069. IR (KBr): ν = 3128, 3062, 3023.2, 2947, 2242, 1587, 1538, 1460, 1287, 1120, 1073, 850, 788, 608 cm^{-1}.

3-((Difluoromethyl)thio)pyridine[9] 4f

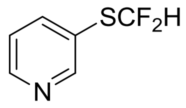

Prepared from 3-iodopyridine (102.5 mg, 0.5 mmol) according to general procedure **A**. The crude residue was purified by flash column chromatography on silica gel to yield **3-((difluoromethyl)thio)pyridine 4f** (60 mg, 75 %) as a pale yellow oil. ^{1}H NMR (400 MHz, $CDCl_3$) δ 8.77 (s, 1 H), 8.65 (d, *J* = 4.7 Hz, 1 H), 7.91 (d, *J* = 7.9 Hz, 1 H), 7.33 (dd, *J* = 7.7, 4.9 Hz, 1 H), 6.83 (t, *J* = 56.3 Hz, 1 H); ^{19}F NMR (376 MHz, $CDCl_3$) δ -91.49 (d, *J* = 56.3 Hz, 2 F); ^{13}C NMR (101 MHz, $CDCl_3$) δ 155.25, 150.79, 143.09, 124.10, 123.11 (t, *J* = 2.9 Hz), 119.68 (t, *J* = 276.7 Hz) ppm.

2-Chloro-5-((difluoromethyl)thio)pyridine 4g

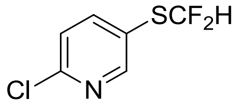

Prepared from 2-chloro-5-iodopyridine (119.5 mg, 0.5 mmol) according to general procedure **A**. The crude residue was purified by flash column chromatography on silica gel to yield **2-chloro-5-((difluoromethyl)thio)pyridine 4g** (85 mg, 87 %) as a

colorless oil. ^{1}H NMR (400 MHz, $CDCl_3$) δ 8.54 (d, *J* = 2.1 Hz, 1 H), 7.86 (dd, *J* = 8.3, 2.4 Hz, 1 H), 7.37 (d, *J* = 8.2 Hz, 1 H), 6.82 (t, *J* = 56.0 Hz, 1 H); ^{19}F NMR (376 MHz, $CDCl_3$) δ -91.64 (d, *J* = 56.0 Hz, 2 F); ^{13}C NMR (101 MHz, $CDCl_3$) δ 155.18, 153.42, 145.52, 124.97, 121.70 (t, *J* = 2.8 Hz), 119.13 (t, *J* = 277.3 Hz) ppm. MS (EI): 195 (100). HRMS (EI) for $C_6H_4ClF_2NS$ Calcd: 197.9721; Found: 194.9722. IR (KBr): ν = 2960, 2867, 1559, 1450, 1356, 1318, 1298, 1138, 1117, 1070, 1044, 1014, 833, 773, 753, 668, 509 cm^{-1}.

5-((Difluoromethyl)thio)-2-fluoropyridine 4h

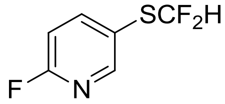

Prepared from 2-fluoro-5-iodopyridine (111.5 mg, 0.5 mmol) according to general procedure **A**. The crude residue was purified by flash column chromatography on silica gel to yield **5-((difluoromethyl)thio)-2-fluoropyridine 4h** (47 mg, 53 %) as a pale yellow oil. ^{1}H NMR (400 MHz, $CDCl_3$) δ 8.41 (s, 1 H), 8.01 (t, *J* = 7.9 Hz, 1 H), 7.01-6.98 (m, 1 H), 6.83 (dt, *J* = 57.3, 28.6 Hz, 1 H); ^{19}F NMR (376 MHz, $CDCl_3$) δ -65.41 (s, 1 F), -92.02 (d, *J* = 56.1 Hz, 2 F); ^{13}C NMR (101 MHz, $CDCl_3$) δ 164.56 (d, *J* = 244.1 Hz), 154.23 (d, *J* = 15.6 Hz), 148.49 (d, *J* = 8.8 Hz), 119.73, 119.19 (t, *J* = 277.3 Hz), 110.53 (d, *J* = 38.0 Hz) ppm. MS (EI): 179 (100). HRMS (EI) for $C_6H_4F_3NS$ Calcd: 179.0017; Found: 197.0021. IR (KBr): ν = 1583, 1507, 1471, 1365, 1256, 1073, 1018, 835, 791, 761, 636, 533 cm^{-1}.

5-Bromo-3-((difluoromethyl)thio)-2-methoxypyridine 4i

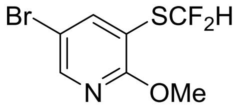

Prepared from 5-bromo-3-iodo-2-methoxypyridine (156.5 mg, 0.5 mmol) according to general procedure **A**. The crude residue was purified by flash column chromatography on silica gel to yield **5-bromo-3-((difluoromethyl)thio)-2-methoxypyridine 4i** (100 mg, 74 %) as a pale yellow oil. ^{1}H NMR (400 MHz, $CDCl_3$) δ 8.22 (d, *J* = 2.3 Hz, 1 H), 7.90 (d, *J* = 2.3

Hz, 1 H), 6.98 (t, J = 57.4 Hz, 1 H), 4.00 (s, 3 H); ^{19}F NMR (376 MHz, $CDCl_3$) δ -92.89 (d, J = 57.3 Hz, 2 F); ^{13}C NMR (101 MHz, $CDCl_3$) δ 161.80, 148.68, 146.18, 119.31 (t, J = 276.6 Hz), 112.20, 111.50, 54.72 ppm. MS (EI): 219.9 (100), 269, 271. HRMS (EI) for $C_7H_6BrF_2NOS$ Calcd: 268.9322; Found: 268.9325. IR (KBr): ν = 2991, 2953, 2925, 2853 1563, 1466, 1404.2, 1372, 1297, 1237, 1065, 1010, 905, 792, 770, 716, 642, 551 cm^{-1}.

3-((Difluoromethyl)thio)-2-methoxypyridine 4j

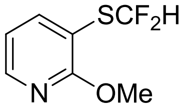

Prepared from 3-iodo-2-methoxypyridine (117.5 mg, 0.5 mmol) according to general procedure **A**. The crude residue was purified by flash column chromatography on silica gel to yield **3-((difluoromethyl)thio)-2-methoxypyridine 4j** (87 mg, 91 %) as a pale yellow oil. ^{1}H NMR (400 MHz, $CDCl_3$) δ 8.19 (dd, J = 5.0, 1.8 Hz, 1 H), 7.80 (dd, J = 7.4, 1.8 Hz, 1 H), 6.97 (t, J = 57.7 Hz, 1 H), 6.90 (dd, J = 7.4, 5.0 Hz, 1 H), 4.02 (s, 3 H); ^{19}F NMR (376 MHz, $CDCl_3$) δ -93.02 (d, J = 57.6 Hz, 2 F); ^{13}C NMR (101 MHz, $CDCl_3$) δ 163.06, 148.28, 144.94, 119.76 (t, J = 275.6 Hz), 117.43, 110.32 (t, J = 3.4 Hz), 54.26 ppm. MS (EI): 140(100), 191. HRMS (EI) for $C_7H_7F_2NOS$ Calcd: 191.0216; Found: 191.0222. IR (KBr): ν = 3057, 2955, 1577, 1466, 1401, 1300, 12560, 1070, 1044, 1014, 833, 801, 763, 697 cm^{-1}.

3-((Difluoromethyl)thio)-2-nitropyridine[2] 4k

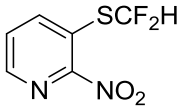

Prepared from 3-iodo-2-nitropyridine (125.0 mg, 0.5 mmol) according to general procedure **A**. The crude residue was purified by flash column chromatography on silica gel to yield **3-((difluoromethyl)thio)-2-nitropyridine 4k** (72 mg, 70 %) as a yellow oil. ^{1}H NMR (400 MHz, $CDCl_3$) δ 8.55-8.54 (m, 1 H), 8.24 (d, J = 8.0 Hz, 1 H), 7.64 (dd, J = 8.0, 4.6 Hz, 1 H), 6.97 (t, J = 55.5 Hz, 1 H); ^{19}F NMR (376 MHz, $CDCl_3$) δ -92.73 (d, J = 55.6 Hz, 2 F); ^{13}C NMR (101 MHz, $CDCl_3$) δ 157.99, 148.10,

143.52, 127.97, 120.41, 119.03 (t, J = 278.1 Hz) ppm.

4-(5-((Difluoromethyl)thio)pyridin-2-yl)morpholine 4l

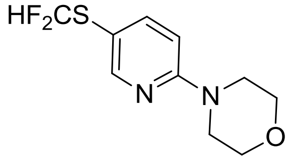

Prepared from 4-(5-iodopyridin-2-yl)morpholine (145.0 mg, 0.5 mmol) according to general procedure **A**. The crude residue was purified by flash column chromatography on silica gel to yield **4-(5-((difluoromethyl)thio)pyridin-2-yl)morpholine 4l** (107 mg, 87 %) as a pale yellow oil. ^{1}H NMR (400 MHz, $CDCl_3$) δ 8.30 (d, J = 2.2 Hz, 1 H), 7.63 (dd, J = 8.9, 2.4 Hz, 1 H), 6.67 (t, J = 57.0 Hz, 1 H), 6.59 (d, J = 8.9 Hz, 1 H), 3.80-3.77 (m, 4 H), 3.57 – 3.54 (m, 4 H); ^{19}F NMR (376 MHz, $CDCl_3$) δ -92.74 (d, J = 56.9 Hz, 2 F); ^{13}C NMR (101 MHz, $CDCl_3$) δ 159.66, 155.02, 144.99, 120.22 (t, J = 275.8 Hz), 108.78 (t, J = 2.9 Hz), 106.73, 66.58, 45.03 ppm. MS (EI): 246.1 (100). HRMS (EI) for $C_{10}H_{12}F_2N_2OS$ Calcd: 246.0638; Found: 246.0634. IR (KBr): ν = 2960, 2925, 2856, 1731, 1586, 1486, 1448, 1398, 1270, 1249.5, 1114, 1069, 1032, 945, 812, 771, 756, 741 cm^{-1}.

2-Chloro-4-((difluoromethyl)thio)pyridine 4m

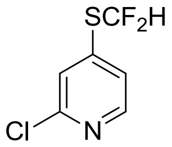

Prepared from 2-chloro-4-iodopyridine (119.5 mg, 0.5 mmol) according to general procedure **A**. The crude residue was purified by flash column chromatography on silica gel to yield **2-chloro-4-((difluoromethyl)thio)pyridine 4m** (80 mg, 82 %) as a pale yellow oil.

Prepared from 2-chloro-4-iodopyridine (119.5 mg, 0.5 mmol) according to general procedure **B**. The crude residue was purified by flash column chromatography on silica gel to yield **2-chloro-4-((difluoromethyl)thio)pyridine 4m** (76 mg, 78 %) as a pale yellow oil.

^{1}H NMR (400 MHz, $CDCl_3$) δ 8.35 (d, J = 5.2 Hz, 1 H), 7.43 (s, 1 H), 7.30 (d, J = 5.2

Hz, 1 H), 7.00 (t, J = 55.5 Hz, 1 H); ^{19}F NMR (376 MHz, $CDCl_3$) δ -91.29 (d, J = 55.5 Hz, 2 F); ^{13}C NMR (101 MHz, $CDCl_3$) δ 152.17, 149.85, 141.43 (t, J = 2.9 Hz), 126.02, 124.41, 119.21 (t, J = 277.1 Hz) ppm. MS (EI): 195.1 (100). HRMS (EI) for $C_6H_4ClF_2NS$ Calcd: 194.9721; Found: 194.9718. IR (KBr): ν = 3052, 2927, 2857, 1568, 1532, 1503, 1456, 1364, 1300, 1149, 1078, 1048, 832, 795, 751 cm^{-1}.

2-Bromo-4-((difluoromethyl)thio)pyridine 4n

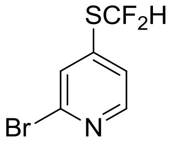

Prepared from 2-bromo-4-iodopyridine (141.5 mg, 0.5 mmol) according to general procedure **A**. The crude residue was purified by flash column chromatography on silica gel to yield **2-bromo-4-((difluoromethyl)thio)pyridine 4n** (78 mg, 65 %) as a pale yellow oil. ^{1}H NMR (400 MHz, $CDCl_3$) δ 8.33 (d, J = 5.2 Hz, 1 H), 7.59 (d, J = 1.3 Hz, 1 H), 7.34 (dd, J = 5.2, 1.5 Hz, 1 H), 6.99 (t, J = 55.5 Hz, 1 H); ^{19}F NMR (376 MHz, $CDCl_3$) δ -91.19 (d, J = 55.5 Hz, 2 F); ^{13}C NMR (101 MHz, $CDCl_3$) δ 150.23, 142.66, 141.14 (t, J = 2.9 Hz), 129.69 , 124.81, 119.17 (t, J = 277.2 Hz) ppm. MS (EI): 239 (100), 241 (100). HRMS (EI) for $C_6H_4BrF_2NS$ Calcd: 238.9216; Found: 238.9218. IR (KBr): ν = 3048, 2964, 1564, 1523, 1455, 1359, 1321, 1300, 1135, 1077, 984, 831, 793, 769, 746, 552 cm^{-1}.

4-((Difluoromethyl)thio)-2-fluoro-5-methylpyridine 4o

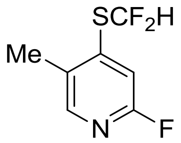

Prepared from 2-fluoro-4-iodo-5-methylpyridine (118.5 mg, 0.5 mmol) according to general procedure **A**. The crude residue was purified by flash column chromatography on silica gel to yield **4-((difluoromethyl)thio)-2-fluoro-5-methylpyridine 4o** (70 mg, 73 %) as a pale yellow oil. ^{1}H NMR (400 MHz, $CDCl_3$) δ 8.04 (s, 1 H), 7.04 (d, J = 2.3 Hz, 1 H), 7.01 (t, J = 55.6 Hz, 1 H), 2.31 (s, 3 H); ^{19}F NMR (376 MHz, $CDCl_3$) δ -71.21 (s, 1 F), -91.99 (d, J = 55.6 Hz, 2 F); ^{13}C NMR (101 MHz, $CDCl_3$) δ 162.16 (d,

J = 239.1 Hz), 147.80 (d, J = 15.1 Hz), 143.16 (dt, J = 8.3, 2.8 Hz), 131.35 (d, J = 4.8 Hz), 119.27 (t, J = 276.5 Hz), 110.46 (d, J = 40.0 Hz), 16.57 ppm. MS (EI): 142 (100), 193. HRMS (EI) for $C_7H_6F_3NS$ Calcd: 193.0173; Found: 193.0177. IR (KBr): ν = 3064, 2962, 2867, 1590, 1557, 1475, 1451, 1387, 1352, 1299.6, 1262, 1240, 1185, 1082, 1046, 917, 853, 794, 764, 746, 709 cm^{-1}.

2-Chloro-4-((difluoromethyl)thio)-5-(trifluoromethyl)pyridine 4p

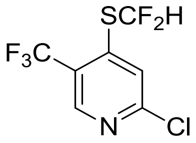

Prepared from 2-chloro-4-iodo-5-(trifluoromethyl)pyridine (153.5 mg, 0.5 mmol) according to general procedure **A**. The crude residue was purified by flash column chromatography on silica gel to yield **2-chloro-4-((difluoromethyl)thio) -5-(trifluoromethyl)pyridine 4p** (70 mg, 53 %) as a pale yellow oil. ^{1}H NMR (400 MHz, $CDCl_3$) δ 8.68 (s, 1 H), 7.64 (s, 1 H), 7.03 (t, J = 55.2 Hz, 1 H); ^{19}F NMR (376 MHz, $CDCl_3$) δ -60.62 (s, 3 F), -91.48 (ddd, J = 55.2, 3.4, 1.8 Hz, 2 F); ^{13}C NMR (101 MHz, $CDCl_3$) δ 155.52, 147.78 (q, J = 6.1 Hz), 141.10, 126.88, 125.32 (q, J = 30.3 Hz), 122.48 (q, J = 274.1 Hz), 118.65 (tq, J = 278.6, 1.6 Hz) ppm. MS (EI): 193 (100), 263. HRMS (EI) for $C_7H_3ClF_5NS$ Calcd: 262.9589; Found: 262.9595. IR (KBr): ν = 3110, 2929, 1571, 1538, 1462, 1315, 1285, 1222, 1114, 1025, 941, 871, 835, 789, 741 cm^{-1}.

4-((difluoromethyl)thio)-2-methoxypyridine 4q

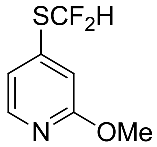

Prepared from 4-iodo-2-methoxypyridine (117.5 mg, 0.5 mmol) according to general procedure **A**. The crude residue was purified by flash column chromatography on silica gel to yield **4-((difluoromethyl)thio)-2-methoxypyridine 4q** (87 mg, 91 %) as a colorless oil. ^{1}H NMR (400 MHz, $CDCl_3$) δ 8.11 (d, J = 5.4 Hz, 1 H), 6.93 (dd, J = 5.4, 1.5 Hz, 1 H), 6.96 (t, J = 56.1 Hz, 1 H), 6.85 (d, J = 1.3 Hz, 1 H), 3.93 (s, 3 H);

^{19}F NMR (376 MHz, $CDCl_3$) δ -91.11 (d, *J* = 56.0 Hz, 2 F); ^{13}C NMR (101 MHz, $CDCl_3$) δ 164.48, 147.34, 140.27 (t, *J* = 3.0 Hz), 119.94 (t, *J* = 275.6 Hz), 119.00, 113.37, 53.71 ppm. MS (EI): 191 (100). HRMS (EI) for $C_7H_7F_2NOS$ Calcd: 191.0216; Found: 191.0222. IR (KBr): ν = 3062, 2984, 2951, 2862, 1589, 1548, 1473, 1385, 1309, 1281, 1224, 1073, 1034, 985, 863, 816, 790, 757, 709, 619 cm^{-1}.

3-((Difluoromethyl)thio)quinolone[9] 4r

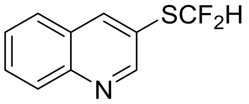

Prepared from 3-iodoquinoline (127.5 mg, 0.5 mmol) according to general procedure **A**. The crude residue was purified by flash column chromatography on silica gel to yield **3-((difluoromethyl)thio)quinolone 4r** (95 mg, 90 %) as a pale yellow solid. Prepared from 3-iodoquinoline (127.5`mg, 0.5 mmol) according to general procedure **B**. The crude residue was purified by flash column chromatography on silica gel to yield **3-((difluoromethyl)thio)quinolone 4r** (97 mg, 92 %) as a pale yellow solid.

^{1}H NMR (400 MHz, $CDCl_3$) δ 8.97 (d, *J* = 1.9 Hz, 1 H), 8.42 (d, *J* = 1.2 Hz, 1 H), 8.12 (d, *J* = 8.5 Hz, 1 H), 7.82 – 7.75 (m, 2 H), 7.61 – 7.57 (m, 1 H), 6.88 (t, *J* = 56.3 Hz, 1 H); ^{19}F NMR (376 MHz, $CDCl_3$) δ -91.33 (d, *J* = 56.3 Hz, 2 F); ^{13}C NMR (101 MHz, $CDCl_3$) δ 154.47, 147.96, 143.63, 131.06, 129.41, 127.83, 127.57, 119.80 (t, *J* = 276.8 Hz), 119.39 (t, *J* = 2.7 Hz) ppm.

6-((Difluoromethyl)thio)quinolone[9] 4s

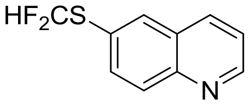

Prepared from 6-iodoquinoline (127.5 mg, 0.5 mmol) according to general procedure **A**. The crude residue was purified by flash column chromatography on silica gel to yield **6-((difluoromethyl)thio)quinolone 4s** (100 mg, 95 %) as a pale yellow oil. ^{1}H NMR (400 MHz, $CDCl_3$) δ 8.92 (dd, *J* = 4.2, 1.7 Hz, 1 H), 8.08 (t, *J* = 8.7 Hz, 2 H), 8.03 (d, *J* = 1.8 Hz, 1 H), 7.79 (dd, *J* = 8.8, 2.0 Hz, 1 H), 7.40 (dd, *J* = 8.3, 4.2 Hz, 1 H), 6.91 (t, *J* = 56.6 Hz, 1 H); ^{19}F NMR (376 MHz, $CDCl_3$) δ -91.30 (d, *J* = 56.6 Hz,

2 F); ^{13}C NMR (101 MHz, $CDCl_3$) δ 151.69, 148.08, 135.91, 135.00, 134.91, 130.53, 128.31, 124.38 (t, J = 3.0 Hz), 121.91, 120.58 (t, J = 275.7 Hz) ppm.

7-Chloro-4-((difluoromethyl)thio)quinolone 4t

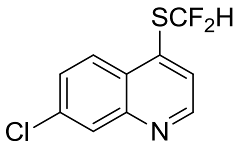

Prepared from 7-chloro-4-iodoquinoline (144.5 mg, 0.5 mmol) according to general procedure **A**. The crude residue was purified by flash column chromatography on silica gel to yield **7-chloro-4-((difluoromethyl)thio)quinolone 4t** (121 mg, 99 %) as a white solid. ^{1}H NMR (400 MHz, $CDCl_3$) δ 8.83 (d, J = 4.5 Hz, 1 H), 8.17 (d, J = 9.0 Hz, 1 H), 8.09 (d, J = 2.0 Hz, 1 H), 7.60 (d, J = 4.5 Hz, 1 H), 7.54 (dd, J = 9.0, 2.1 Hz, 1 H), 6.99 (t, J = 56.0 Hz, 1 H); ^{19}F NMR (376 MHz, $CDCl_3$) δ -90.49 (d, J = 56.0 Hz, 2 F); ^{13}C NMR (101 MHz, $CDCl_3$) δ 150.58, 148.89, 136.32, 136.08 (t, J = 2.8 Hz), 129.03, 128.65, 127.22, 126.31, 126.14, 119.81 (t, J = 277.3 Hz). MS (EI): 245 (100). HRMS (EI) for $C_{10}H_6ClF_2NS$ Calcd: 244.9878; Found: 244.9577. IR (KBr): ν = 3000, 1603, 1560, 1487, 1415, 1329, 1291 1192, 1070, 1024, 975, 874, 827, 813, 787, 627 cm^{-1}. Mp: 75.2 – 76.9 ^{o}C.

8-(Benzyloxy)-5,7-dichloro-3-((difluoromethyl)thio)quinolone 4u

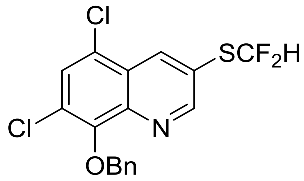

Prepared from 8-(benzyloxy)-5,7-dichloro-3-iodoquinoline (214.5 mg, 0.5 mmol) according to general procedure **A**. The crude residue was purified by flash column chromatography on silica gel to yield **8-(Benzyloxy)-5,7-dichloro-3-((difluoromethyl)thio)quinolone 4u** (171 mg, 89 %) as a white solid. ^{1}H NMR (400 MHz, $CDCl_3$) δ 9.08 (d, J = 2.1 Hz, 1 H), 8.75 (d, J = 2.1 Hz, 1 H), 7.67 (s, 1 H), 7.61 – 7.59 (m, 2 H), 7.41 – 7.34 (m, 3 H), 6.95 (t, J = 56.0 Hz, 1 H), 5.47 (s, 2 H); ^{19}F NMR (376 MHz, $CDCl_3$) δ -90.89 (d, J = 56.0 Hz, 2 F); ^{13}C NMR (101 MHz, $CDCl_3$)

δ 154.61, 150.44, 143.50, 140.34, 136.76, 129.03, 128.68, 128.63, 128.42, 128.35, 126.02, 126.00, 121.43 (t, J = 2.6 Hz), 119.48 (t, J = 277.5 Hz), 76.87 ppm. MS (EI): 91 (100), 385. HRMS (EI) for $C_{17}H_{11}Cl_2F_2NOS$ Calcd: 384.9906; Found: 384.9904. IR (KBr): ν = 3070, 2888, 1591, 1575, 1462, 1439, 1347, 1323, 1284, 1215, 1057, 1025, 954, 907, 874, 846, 754, 696, 633 cm^{-1}. Mp: 67.1 – 69.2 ^{o}C.

1-((Difluoromethyl)thio)isoquinoline 4v

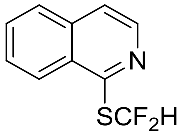

Prepared from 1-iodoisoquinoline (127.5 mg, 0.5 mmol) according to general procedure **A**. The crude residue was purified by flash column chromatography on silica gel to yield **1-((difluoromethyl)thio)isoquinoline 4v** (103 mg, 98 %) as a pale green yellow oil. ^{1}H NMR (400 MHz, $CDCl_3$) δ 8.33 (d, J = 5.7 Hz, 1 H), 8.04 (t, J = 56.2 Hz, 1 H), 7.98 (d, J = 8.4 Hz, 1 H), 7.79 (d, J = 8.2 Hz, 1 H), 7.70 (t, J = 7.5 Hz, 1 H), 7.60 (t, J = 7.7 Hz, 1 H), 7.46 (d, J = 5.7 Hz, 1 H); ^{19}F NMR (376 MHz, $CDCl_3$) δ -97.60 (d, J = 56.1 Hz, 2 F); ^{13}C NMR (101 MHz, $CDCl_3$) δ 154.63 (t, J = 3.6 Hz), 141.89, 136.00, 130.95, 127.78, 127.37, 126.95 (t, J = 2.0 Hz), 124.18, 121.70 (t, J = 270.0 Hz), 119.17 ppm. MS (EI): 129 (100), 211. HRMS (EI) for $C_{10}H_7F_2NS$ Calcd: 211.0267; Found: 211.0272. IR (KBr): ν = 3057, 1621, 1585, 1554, 1494, 1317, 1277, 1261, 1144, 1071, 985, 866, 839, 787, 744, 674, 646 cm^{-1}.

5-((Difluoromethyl)thio)-1-methyl-1*H*-indole 4w

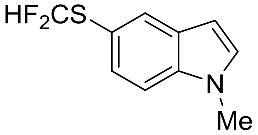

Prepared from 5-iodo-1-methyl-1*H*-indole (128.5 mg, 0.5 mmol) according to general procedure **A**. The crude residue was purified by flash column chromatography on silica gel to yield **5-((difluoromethyl)thio)-1-methyl-1*H*-indole 4w** (101 mg, 95 %) as a pale yellow oil. ^{1}H NMR (400 MHz, $CDCl_3$) δ 7.91 (d, J = 1.5 Hz, 1 H), 7.43 (dd, J = 8.5, 1.6 Hz, 1 H), 7.33 (d, J = 8.5 Hz, 1 H), 7.11 (d, J = 3.1 Hz, 1 H), 6.81 (t, J =

57.6 Hz, 1 H), 6.52 (dd, *J* = 3.1, 0.6 Hz, 1 H), 3.80 (s, 3 H); ^{19}F NMR (376 MHz, $CDCl_3$) δ -91.97 (d, *J* = 57.6 Hz, 2 F); ^{13}C NMR (101 MHz, $CDCl_3$) δ 137.29, 130.19, 129.48, 129.32, 128.87, 121.85 (t, *J* = 274.7 Hz), 115.00, 110.05, 101.38, 32.99 ppm. MS (EI): 162 (100), 213. HRMS (EI) for $C_{10}H_9F_2NS$ Calcd: 213.0424; Found: 213.0422. IR (KBr): ν = 3102, 2947, 2919, 2883, 2819, 1607, 1512, 1476, 1422, 1329, 1294, 1278, 1244, 1061, 1030, 887, 806, 757, 723, 611 cm^{-1}.

3-((Difluoromethyl)thio)-9-phenyl-9*H*-carbazole 4x

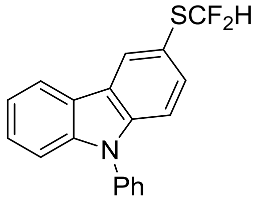

Prepared from 3-iodo-9-phenyl-9*H*-carbazole (134.5 mg, 0.5 mmol) according to general procedure **A**. The crude residue was purified by flash column chromatography on silica gel to yield **3-((difluoromethyl)thio)-9-phenyl-9*H*-carbazole 4x** (80 mg, 71 %) as a colorless oil.

Prepared from 3-iodo-9-phenyl-9*H*-carbazole (134.5 mg, 0.5 mmol) according to general procedure **B**. The crude residue was purified by flash column chromatography on silica gel to yield **3-((difluoromethyl)thio)-9-phenyl-9*H*-carbazole 4x** (130 mg, 80 %) as a colorless oil.

^{1}H NMR (400 MHz, $CDCl_3$) δ 8.42 (d, *J* = 1.6 Hz, 1 H), 8.17 (d, *J* = 7.8 Hz, 1 H), 7.66-7.33 (m, 10 H), 6.87 (t, *J* = 57.3 Hz, 1 H); ^{19}F NMR (376 MHz, $CDCl_3$) δ -91.98 (d, *J* = 57.4 Hz, 2 F); ^{13}C NMR (101 MHz, $CDCl_3$) δ 141.69, 141.43, 137.10, 133.56, 130.07, 128.76, 128.01, 127.15, 126.80, 124.38, 122.58, 121.49 (t, *J* = 275.2 Hz), 120.68, 120.58, 115.36 (t, *J* = 2.8 Hz), 110.61, 110.13 ppm. MS (EI): 274.1 (100), 325. HRMS (EI) for $C_{19}H_{13}F_2NS$ Calcd: 325.0737; Found: 325.0731. IR (KBr): ν = 3063, 2962, 2927, 2854, 1595, 1487, 1469, 1361, 1329, 1294, 1064, 1028, 889, 819, 748, 728, 699, 667, 619, 600, 567 cm^{-1}.

5-((Difluoromethyl)thio)benzofuran 4y

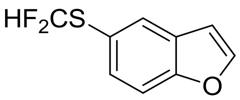

Prepared from 5-iodobenzofuran (122 mg, 0.5 mmol) according to general procedure **A**. The crude residue was purified by flash column chromatography on silica gel to yield **5-((difluoromethyl)thio)benzofuran 4y** (97 mg, 97 %) as a pale yellow oil. ^{1}H NMR (400 MHz, $CDCl_3$) δ 7.88 (s, 1 H), 7.68 (d, *J* = 2.2 Hz, 1 H), 7.52 (s, 2 H), 6.83 (t, *J* = 57.1 Hz, 1 H), 6.79 (d, *J* = 2.1 Hz, 1 H); ^{19}F NMR (376 MHz, $CDCl_3$) δ -91.99 (d, *J* = 57.1 Hz, 2 F); ^{13}C NMR (101 MHz, $CDCl_3$) δ 155.71, 146.26, 131.85, 129.40, 128.68, 121.15 (t, *J* = 275.1 Hz), 119.32 (t, *J* = 3.1 Hz), 112.31, 106.49 ppm. MS (EI): 200.1 (100). HRMS (EI) for $C_9H_6F_2OS$ Calcd: 200.0107; Found: 200.0114. IR (KBr): ν = 3125, 2965, 1535, 1449, 1326, 1296, 1261, 1249, 1174, 1112, 1073, 1062, 1031, 875, 815, 757, 737, 613 cm^{-1}.

1-(5-((Difluoromethyl)thio)thiophen-2-yl)ethanone 4z

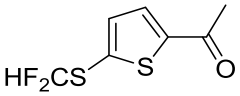

Prepared from 1-(5-iodothiophen-2-yl)ethanone (126 mg, 0.5 mmol) according to general procedure **A**. The crude residue was purified by flash column chromatography on silica gel to yield **1-(5-((difluoromethyl)thio)thiophen-2-yl)ethanone 4z** (76 mg, 73 %) as a pale yellow oil. ^{1}H NMR (400 MHz, $CDCl_3$) δ 7.61 (d, *J* = 3.9 Hz, 1 H), 7.30 (d, *J* = 3.9 Hz, 1 H), 6.78 (t, *J* = 56.5 Hz, 1 H), 2.54 (s, 3 H); ^{19}F NMR (376 MHz, $CDCl_3$) δ -92.46 (d, *J* = 56.5 Hz, 2 F); ^{13}C NMR (101 MHz, $CDCl_3$) δ 189.99, 149.79, 138.02, 132.26, 131.67 (t, *J* = 3.6 Hz), 119.40 (t, *J* = 278.9 Hz), 26.70 ppm. MS (EI): 208 (100). HRMS (EI) for $C_7H_6F_2OS_2$ Calcd: 207.9828; Found: 207.9824. IR (KBr): ν = 3095, 3005, 1667, 1516, 1420, 1361, 1316, 1267, 1072, 1000, 929, 812, 784, 746, 608 cm^{-1}.

4-((Difluoromethyl)thio)dibenzo[b,d]thiophene 4aa

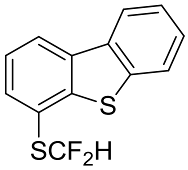

Prepared from 4-iododibenzo[*b,d*]thiophene (155 mg, 0.5 mmol) according to general procedure **A**. The crude residue was purified by flash column chromatography on silica gel to yield **4-((difluoromethyl)thio)dibenzo[*b,d*]thiophene 4aa** (126 mg, 95 %) as a white solid.

Prepared from 4-iododibenzo[*b,d*]thiophene (155 mg, 0.5 mmol) according to general procedure **B**. The crude residue was purified by flash column chromatography on silica gel to yield **4-((difluoromethyl)thio)dibenzo[*b,d*]thiophene 4aa** (100 mg, 75 %) as a white solid.

^{1}H NMR (400 MHz, $CDCl_3$) δ 8.18 (d, *J* = 7.9 Hz, 1 H), 8.12 (dd, *J* = 5.9, 2.9 Hz, 1 H), 7.88 (dd, *J* = 6.1, 2.7 Hz, 1 H), 7.71 (d, *J* = 7.4 Hz, 1 H), 7.51 – 7.46 (m, 3 H), 6.95 (t, *J* = 57.0 Hz, 1 H); ^{19}F NMR (376 MHz, $CDCl_3$) δ -90.52 (d, *J* = 57.0 Hz, 2 F); ^{13}C NMR (101 MHz, $CDCl_3$) δ 147.23, 139.36, 136.57, 135.78, 134.89, 127.36, 125.42, 124.78, 123.36, 122.87, 122.03, 120.55 (t, *J* = 277.3 Hz), 119.63 (t, *J* = 3.2 Hz) ppm. MS (EI): 266 (100). HRMS (EI) for $C_{13}H_8F_2S_2$ Calcd: 266.0035; Found: 266.0038. IR (KBr): ν = 3058, 2989, 1451, 1386, 1291, 1249, 1198, 1085, 1013, 798, 748, 704, 494 cm^{-1}. Mp: 68.7 – 73.2 ^oC.

2-Chloro-5-((difluoromethyl)thio)pyrimidine 4ab

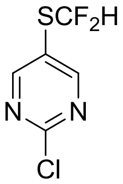

Prepared from 2-chloro-5-iodopyrimidine (120 mg, 0.5 mmol) according to general procedure **A**. The crude residue was purified by flash column chromatography on silica gel to yield **2-chloro-5-((difluoromethyl)thio)pyrimidine 4ab** (32 mg, 33 %) as a pale yellow solid. ^{1}H NMR (400 MHz, $CDCl_3$) δ 8.77 (s, 2 H), 6.86 (t, *J* = 55.4 Hz, 1 H); ^{19}F NMR (376 MHz, $CDCl_3$) δ -91.13 (d, *J* = 55.4 Hz, 2 F); ^{13}C NMR (101

MHz, $CDCl_3$) δ 164.69, 162.93, 120.65 (t, J = 2.6 Hz) 118.12 (t, J = 278.8 Hz) ppm. MS (EI): 196 (100). HRMS (EI) for $C_5H_3ClF_2N_2S$ Calcd: 195.9674; Found: 195.9675. IR (KBr): ν 3045, 2927, 1592, 1552, 1531, 1390.1, 1348, 1315, 1174, 1072, 1043, 794, 770, 746, 636 cm^{-1}.

5-Bromo-2-((difluoromethyl)thio)pyrimidine 4ac

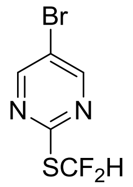

Prepared from 5-bromo-2-iodopyrimidine (142 mg, 0.5 mmol) according to general procedure **A**. The crude residue was purified by flash column chromatography on silica gel to yield **5-bromo-2-((difluoromethyl)thio)pyrimidine 4ac** (100 mg, 83 %) as a pale yellow oil. ^{1}H NMR (400 MHz, $CDCl_3$) δ 8.63 (s, 2 H), 7.70 (t, J = 55.8 Hz, 1 H); ^{19}F NMR (376 MHz, $CDCl_3$) δ -98.97 (d, J = 55.8 Hz, 2 F); ^{13}C NMR (101 MHz, $CDCl_3$) δ 165.84 (t, J = 5.8 Hz), 158.38, 120.33 (t, J = 271.3 Hz), 116.95 ppm. MS (EI): 160 (100), 240, 242. HRMS (EI) for $C_5H_3BrF_2N_2S$ Calcd: 239.9168; Found: 239.9171. IR (KBr): ν = 3033, 2927, 1546, 1527, 1389, 1362, 1289, 1191, 1111, 1072, 1006, 929, 791, 763, 631 cm^{-1}.

5-chloro-2-((difluoromethyl)thio)pyrimidine 4ad

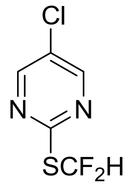

Prepared from 5-chloro-2-iodopyrimidine (120 mg, 0.5 mmol) according to general procedure **A**. The crude residue was purified by flash column chromatography on silica gel to yield **5-chloro-2-((difluoromethyl)thio)pyrimidine 4ad** (96 mg, 98 %) as a pale yellow oil.

Prepared from 5-chloro-2-iodopyrimidine (120 mg, 0.5 mmol) according to general procedure **B**. The crude residue was purified by flash column chromatography on

silica gel to yield **5-chloro-2-((difluoromethyl)thio)pyrimidine 4ad** (75 mg, 76 %) as a pale yellow oil.

^{1}H NMR (400 MHz, $CDCl_3$) δ 8.55 (s, 2 H), 7.70 (t, *J* = 55.8 Hz, 1 H); ^{19}F NMR (376 MHz, $CDCl_3$) δ -99.01 (d, *J* = 55.8 Hz, 2 F); ^{13}C NMR (101 MHz, $CDCl_3$) δ 165.35 (t, *J* = 5.9 Hz), 156.28, 128.56, 120.42 (t, *J* = 271.2 Hz) ppm. MS (EI): 114 (100), 196. HRMS (EI) for $C_5H_3ClF_2N_2S$ Calcd: 195.9674; Found: 195.9680. IR (KBr): ν = 3038, 2928, 1555, 1530, 1387, 1290, 1194, 1073, 930, 792, 781, 763, 632, 586, 552 cm^{-1}. Mp: 47.5 – 49.3 ^oC.

2-((Difluoromethyl)thio)pyrazine 4ae

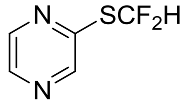

Prepared from 2-iodopyrazine (103 mg, 0.5 mmol) according to general procedure **A**. The crude residue was purified by flash column chromatography on silica gel to yield **2-((difluoromethyl)thio)pyrazine 4ae** (35 mg, 43 %) as a pale yellow oil. ^{1}H NMR (400 MHz, $CDCl_3$) δ 8.55 (d, *J* = 1.4 Hz, 1 H), 8.47 – 8.46 (m, 1 H), 8.42 (d, *J* = 2.5 Hz, 1 H), 7.61 (t, *J* = 56.0 Hz, 1 H); ^{19}F NMR (376 MHz, $CDCl_3$) δ -95.68 (d, *J* = 56.0 Hz, 2 F); ^{13}C NMR (101 MHz, $CDCl_3$) δ 151.13 (t, *J* = 3.5 Hz), 145.08 (t, *J* = 2.3 Hz), 144.48, 142.26, 120.34 (t, *J* = 273.3 Hz) ppm. MS (EI): 80 (100), 162. HRMS (EI) for $C_5H_4F_2N_2S$ Calcd: 162.0063; Found: 162.0070. IR (KBr): ν = 3065, 2960, 1559, 1513, 1458, 1388, 1288, 1133, 1073, 1010, 840, 789, 768, 755 cm^{-1}.

2,5-Bis((difluoromethyl)thio)pyrazine 4af

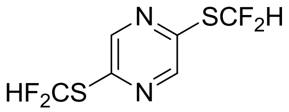

Prepared from 2-bromo-5-iodopyrazine (142 mg, 0.5 mmol) according to general procedure **A**. The crude residue was purified by flash column chromatography on silica gel to yield **2,5-bis((difluoromethyl)thio)pyrazine 4af** (15 mg, 12 %) as a pale yellow oil. When 2.4 eqiuv (SIPr)Ag(SCF_2H) was used, the yield determined by ^{19}F NMR analysis was 93 %。 ^{1}H NMR (400 MHz, $CDCl_3$) δ 8.46 (s, 2 H), 7.52 (t, *J* =

56.0 Hz, 2 H); ^{19}F NMR (376 MHz, $CDCl_3$) δ -95.22 (d, *J* = 55.9 Hz, 2 F); ^{13}C NMR (101 MHz, $CDCl_3$) δ 148.44 (t, *J* = 3.6 Hz), 144.79 (t, *J* = 2.4 Hz), 120.06 (t, *J* = 274.6 Hz) ppm. MS (EI): 244 (100). HRMS (EI) for $C_6H_4F_4N_2S_2$ Calcd: 243.9752; Found: 243.9751. IR (KBr): ν = 2928, 1497, 1450, 1289, 1161, 1070, 1015, 899, 781, 743, 587 cm^{-1}.

Methyl 3-((difluoromethyl)thio)pyrazine-2-carboxylate 4ag

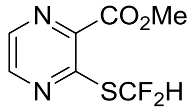

Prepared from methyl 3-iodopyrazine-2-carboxylate (132 mg, 0.5 mmol) according to general procedure **A**. The crude residue was purified by flash column chromatography on silica gel to yield **methyl 3-((difluoromethyl)thio)pyrazine-2-carboxylate 4ag** (92 mg, 84 %) as a white solid. ^{1}H NMR (400 MHz, $CDCl_3$) δ 8.57 (d, *J* = 2.3 Hz, 1 H), 8.49 (d, *J* = 2.3 Hz, 1 H), 7.74 (t, *J* = 55.8 Hz, 1 H), 4.02 (s, 3 H); ^{19}F NMR (376 MHz, $CDCl_3$) δ -101.63 (d, *J* = 55.8 Hz, 2 F); ^{13}C NMR (101 MHz, $CDCl_3$) δ 164.45, 156.03 (t, *J* = 4.3 Hz), 145.97, 140.10, 138.91, 119.48 (t, *J* = 270.2 Hz), 53.47 ppm. MS (EI): 220 (100). HRMS (EI) for $C_7H_6F_2N_2O_2S$ Calcd: 220.0118; Found: 220.0117. IR (KBr): ν = 3023, 2964, 1710, 1526, 1455, 1380, 1300, 1278, 1228, 1158, 1069, 1048, 949, 871, 854, 807, 785, 762, 524, 440 cm^{-1}. Mp: 94.6 – 96.2 ^{o}C.

4-Chloro-6-((difluoromethyl)thio)quinazoline 4ah

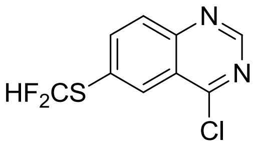

Prepared from 4-chloro-6-iodoquinazoline (145 mg, 0.5 mmol) according to general procedure **A**. The crude residue was purified by flash column chromatography on silica gel to yield **4-chloro-6-((difluoromethyl)thio)quinazoline 4ah** (55 mg, 45 %) as a white solid. ^{1}H NMR (400 MHz, $CDCl_3$) δ 9.09 (s, 1 H), 8.51 (t, *J* = 1.2 Hz, 1 H), 8.09 (d, *J* = 1.2 Hz, 2 H), 6.97 (t, *J* = 56.0 Hz, 1 H); ^{19}F NMR (376 MHz, $CDCl_3$) δ -91.25 (d, *J* = 56.1 Hz, 2 F); ^{13}C NMR (101 MHz, $CDCl_3$) δ 162.31, 154.73, 151.13,

140.23, 132.33, 129.94, 128.98 (t, J = 3.0 Hz), 124.24, 119.80 (t, J = 277.0 Hz) ppm. MS (EI): 246 (100). HRMS (EI) for $C_9H_5ClF_2N_2S$ Calcd: 245.9830; Found: 245.9824. IR (KBr): ν = 3054, 3007, 2676, 2603, 1712, 1645, 1559, 1474, 1321, 1143, 1065, 1025, 983, 869, 846, 785, 755, 695, 501 cm^{-1}. Mp: 138.6 – 140.2 ^oC.

***tert*-Butyl 5-((difluoromethyl)thio)-1*H*-indazole-1-carboxylate 4ai**

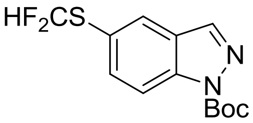

Prepared from *tert*-butyl 5-iodo-1*H*-indazole-1-carboxylate (172 mg, 0.5 mmol) according to general procedure **A**. The crude residue was purified by flash column chromatography on silica gel to yield ***tert*-butyl 5-((difluoromethyl)thio)-1*H*-indazole-1-carboxylate 4ai** (111 mg, 74 %) as a white solid. ^1H NMR (400 MHz, $CDCl_3$) δ 8.19 (d, J = 8.7 Hz, 1 H), 8.17 (s, 1 H), 7.99 (d, J = 0.7 Hz, 1 H), 7.70 (dd, J = 8.7, 1.4 Hz, 1 H), 6.83 (t, J = 56.7 Hz, 1 H), 1.71 (s, 9 H); ^{19}F NMR (376 MHz, $CDCl_3$) δ -91.99 (d, J = 56.7 Hz, 2 F); ^{13}C NMR (101 MHz, $CDCl_3$) δ 148.83, 140.19, 139.01, 135.76, 129.21, 126.64, 120.51 (t, J = 275.7 Hz), 120.46 (t, J = 2.9 Hz), 115.36, 85.51, 28.11 ppm. MS (EI): 200 (100), 300. HRMS (EI) for $C_{13}H_{14}F_2N_2O_2S$ Calcd: 300.0744; Found: 300.0745. IR (KBr): ν = 3004, 2990, 1756, 1434, 1364, 1295, 1322, 1295, 1238, 1076, 1060, 1020, 904, 852, 821, 762, 590 cm^{-1}. Mp: 104.4 – 105.8 ^oC.

7-Chloro-2-((difluoromethyl)thio)thieno[3,2-b]pyridine-6-carbonitrile 4aj

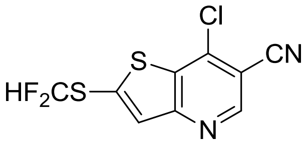

Prepared from 7-chloro-2-iodothieno[3,2-*b*]pyridine-6-carbonitrile (160 mg, 0.5 mmol) according to general procedure **A**. The crude residue was purified by flash column chromatography on silica gel to yield **7-chloro-2-((difluoromethyl)thio)thieno[3,2-b]pyridine-6-carbonitrile 4aj** (70 mg, 51 %) as a white solid. ^1H NMR (400 MHz, $CDCl_3$) δ 8.87 (s, 1 H), 7.85 (s, 1 H), 6.96

(t, J = 55.8 Hz, 1 H); ^{19}F NMR (376 MHz, $CDCl_3$) δ -91.31 (d, J = 55.8 Hz, 2 F); ^{13}C NMR (101 MHz, $CDCl_3$) δ 157.66, 150.62, 140.39, 137.02, 136.22, 134.66, 118.79 (t, J = 280.4 Hz), 114.17, 106.01 ppm. MS (EI): 226 (100), 276. HRMS (EI) for $C_9H_3ClF_2N_2S_2$ Calcd: 275.9394; Found: 275.9388. IR (KBr): ν = 3059, 2233, 1567, 1514, 1517, 1467, 1446, 1346, 1310, 1126, 1066, 1042, 997, 859, 746, 730, 677, 529 cm^{-1}. Mp: 146.3 – 148.0 ^{o}C.

6-((Difluoromethyl)thio)benzo[*d*]thiazole 4ak

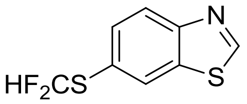

Prepared from 6-iodobenzo[*d*]thiazole (130.5 mg, 0.5 mmol) according to general procedure **A**. The crude residue was purified by flash column chromatography on silica gel to yield **6-((Difluoromethyl)thio)benzo[*d*]thiazole 4ak** (106 mg, 98 %) as a pale yellow oil. ^{1}H NMR (400 MHz, $CDCl_3$) δ 9.06 (s, 1 H), 8.20 (d, J = 1.1 Hz, 1 H), 8.12 (d, J = 8.5 Hz, 1 H), 7.70 (dd, J = 8.5, 1.4 Hz, 1 H), 6.87 (t, J = 56.7 Hz, 1 H); ^{13}C NMR (101 MHz, $CDCl_3$) δ 155.90, 154.08, 134.68, 133.28, 129.27, 124.19, 123.07 (t, J = 3.0 Hz), 120.57 (t, J = 275.9 Hz) ppm. MS (EI): 217 (100). HRMS (EI) for $C_8H_5F_2NS_2$ Calcd: 216.9831; Found: 216.9836. IR (KBr): ν = 3064, 2964, 1724, 1462, 1430, 1387, 1293, 1067, 1032, 884, 843, 812, 764, 517 cm^{-1}.

General Procedure for Difluoromethylthiolation of Heteroaryl Bromide.

Method C

Heteroaryl bromide (0.5 mmol, 1.0 equiv), Pd(dba)$_2$ (30 mg, 10 mol%), XantPhos (44 mg, 15 mol%), and [(SIPr)Ag(SCF$_2$H)] (350 mg, 0.6 mmol, 1.2 equiv) were added in a 20 mL schlenk tube under argon. To the tube was added 2.5 mL of anhydrous toluene and the mixture was stirred at 50 ^{o}C for 12 h. The dark solution was diluted with Et$_2$O (15.0 mL). The mixture was filtered through a short plug of silica gel, washed with Et$_2$O (100 mL). The organic layer was combined, and concentrated under vacuum. The crude product was purified by column chromatography on silica gel with a mixed solvent of pentane/Et$_2$O or pentane/EtOAc as the eluent to give the product.

Method D

Heteroaryl bromide (0.5 mmol, 1.0 equiv), XantphosPd(3-py)(Br) (21 mg, 5 mol%), XantPhos (7.5 mg, 2.5 mol%), and [(SIPr)Ag(SCF$_2$H)] (350 mg, 0.6 mmol, 1.2 equiv) were added in a 20 mL schlenk tube under argon. To the tube was added 5.0 mL of anhydrous toluene and the mixture was stirred at 50 ^{o}C for 6 h. The dark solution was diluted with Et$_2$O (15.0 mL). The mixture was filtered through a short plug of silica gel, washed with Et$_2$O (100 mL). The organic layer was combined, and concentrated under vacuum. The crude product was purified by column chromatography on silica gel with a mixed solvent of pentane/Et$_2$O or pentane/EtOAc as the eluent to give the product.

Methyl 6-((difluoromethyl)thio)nicotinate 4c

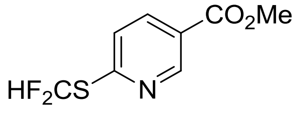

Prepared from methyl 6-bromonicotinate (107.5 mg, 0.5 mmol) according to general procedure **C**. The crude residue was purified by flash column chromatography on silica gel to yield **methyl 6-((difluoromethyl)thio)nicotinate 4c** (109 mg, >99 %) as a white solid. (^{1}H, ^{19}F and ^{13}C NMR spectra are the same as those for the product in

Scheme 2, 4c).

2-((Difluoromethyl)thio)-5-nitropyridine 4d

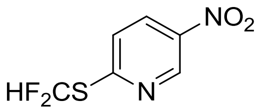

Prepared from 2-bromo-5-nitropyridine (101 mg, 0.5 mmol) according to general procedure **C**. The crude residue was purified by flash column chromatography on silica gel to yield **2-((difluoromethyl)thio)-5-nitropyridine 4d** (88 mg, 83 %) as a white solid. (^{1}H, ^{19}F and ^{13}C NMR spectra are the same as those for the product in **Scheme 2, 4d**).

2-((Difluoromethyl)thio)isonicotinonitrile 4e

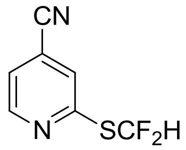

Prepared from 2-bromoisonicotinonitrile (91 mg, 0.5 mmol) according to general procedure **C**. The crude residue was purified by flash column chromatography on silica gel to yield **2-((difluoromethyl)thio)isonicotinonitrile 4e** (86%, determined by ^{19}F NMR analysis) as a colorless oil. The compound was further purified by prep-HPLC with a C18 column (Waters, Prep Nova-pak® HR C18, 19×300 mm，6 μ m) using a water-acetonitrile mixed solvent as the eluent (CH_3CN : H_2O = 80 : 20, flow = 10.0 mL/min, 254 nm). (^{1}H, ^{19}F and ^{13}C NMR spectra are the same as those for the product in **Scheme 2, 4e**).

6-((Difluoromethyl)thio)nicotinonitrile 4al

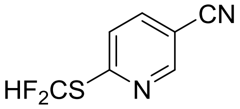

Prepared from 6-bromonicotinonitrile (91 mg, 0.5 mmol) according to general procedure **C**. The crude residue was purified by flash column chromatography on silica gel to yield **6-((difluoromethyl)thio)nicotinonitrile 4al** (89%, determined by ^{19}F NMR analysis) as a white solid. The compound was further purified by

prep-HPLC with a C18 column (Waters, Prep Nova-pak® HR C18, 19×300 mm，6 μ m) using a water-acetonitrile mixed solvent as the eluent (CH_3CN : H_2O = 70 : 30, flow = 10.0 mL/min, 254 nm). ^{1}H NMR (400 MHz, $CDCl_3$) δ 8.72 (d, *J* = 1.4 Hz, 1 H), 7.83 (dd, *J* = 8.4, 2.2 Hz, 1 H), 7.78 (t, *J* = 55.7 Hz, 1 H), 7.33 (d, *J* = 8.4 Hz, 1 H); ^{19}F NMR (376 MHz, $CDCl_3$) δ -97.83 (d, *J* = 55.7 Hz, 1 H); ^{13}C NMR (101 MHz, $CDCl_3$) δ 159.51, 152.39, 139.36, 122.83 (t, *J* = 2.4 Hz), 119.90 (t, *J* = 272.5 Hz), 116.14, 107.22 ppm. MS (EI): 136.1 (100), 186. HRMS (EI) for $C_7H_4F_2N_2S$ Calcd: 186.0063; Found: 186.0064. IR (KBr): ν = 3089, 2923, 2238, 1585, 1463, 1367, 1292, 1117, 1063, 1023, 850, 777, 598, 554 cm^{-1}. Mp: 54.8 – 56.4 ^oC.

3,5-Dichloro-2-((difluoromethyl)thio)pyridine 4am

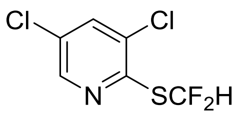

Prepared from 2-bromo-3,5-dichloropyridine (112.5 mg, 0.5 mmol) according to general procedure **C**. The crude residue was purified by flash column chromatography on silica gel to yield **3,5-dichloro-2-((difluoromethyl)thio)pyridine 4am** (102 mg, 89 %) as a colorless oil. ^{1}H NMR (400 MHz, $CDCl_3$) δ 8.35 (d, *J* = 2.2 Hz, 1 H), 7.73 (t, *J* = 55.9 Hz, 1 H), 7.68 (d, *J* = 2.2 Hz, 1 H); ^{19}F NMR (376 MHz, $CDCl_3$) δ -98.85 (d, *J* = 55.9 Hz, 2 F); ^{13}C NMR (101 MHz, $CDCl_3$) δ 151.29 (t, *J* = 4.4 Hz), 146.27, 136.45, 129.50, 129.34 (t, *J* = 2.3 Hz), 120.60 (t, *J* = 271.0 Hz) ppm. MS (EI): 179 (100), 229. HRMS (EI) for $C_6H_3Cl_2F_2NS$ Calcd: 228.9331; Found: 228.9330. IR (KBr): ν = 3071, 2928, 1558, 1408, 1363, 1286, 1212, 1072, 1041, 893, 840, 783, 719, 588 cm^{-1}.

1-(6-((Difluoromethyl)thio)pyridin-3-yl)ethanone 4an

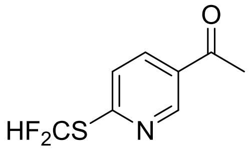

Prepared from 1-(6-bromopyridin-3-yl)ethanone (99.5`mg, 0.5 mmol) according to general procedure **C**. The crude residue was purified by flash column chromatography

on silica gel to yield **1-(6-((difluoromethyl)thio)pyridin-3-yl)ethanone 4an** (98 mg, 97 %) as a pale yellow oil.

Prepared from 1-(6-bromopyridin-3-yl)ethanone (99.5 mg, 0.5 mmol) according to general procedure **D**. The crude residue was purified by flash column chromatography on silica gel to yield **1-(6-((difluoromethyl)thio)pyridin-3-yl)ethanone 4an** (76 mg, 75 %) as a pale yellow oil.

^{1}H NMR (400 MHz, $CDCl_3$) δ 8.96 (d, *J* = 1.5 Hz, 1 H), 8.10 (dd, *J* = 8.4, 2.1 Hz, 1 H), 7.80 (t, *J* = 55.9 Hz, 1 H), 7.28 (d, *J* = 8.4 Hz, 1 H), 2.58 (s, 3 H); ^{19}F NMR (376 MHz, $CDCl_3$) δ -97.55 (d, *J* = 55.9 Hz, 1 H); ^{13}C NMR (101 MHz, $CDCl_3$) δ 195.69, 159.06 (t, *J* = 3.7 Hz), 150.08, 136.18, 130.05, 122.87 (t, *J* = 2.4 Hz), 120.45 (t, *J* = 271.5 Hz), 26.55 ppm. MS (EI): 138 (100), 203. HRMS (EI) for $C_8H_7F_2NOS$ Calcd: 203.0216; Found: 203.0218. IR (KBr): ν = 3057, 2963, 1723, 1690, 1583, 1551, 1459, 1367, 1121, 1066, 1016, 834, 792, 775 cm^{-1}.

4-Bromo-2-((difluoromethyl)thio)pyridine 4ao

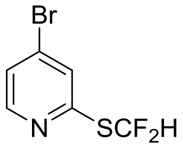

Prepared from 2,4-dibromopyridine (117.5 mg, 0.5 mmol) according to general procedure **C**. The crude residue was purified by flash column chromatography on silica gel to yield **4-bromo-2-((difluoromethyl)thio)pyridine 4ao** (60 mg, 50 %) as a colorless oil. ^{1}H NMR (400 MHz, $CDCl_3$) δ 8.30 (d, *J* = 5.3 Hz, 1 H), 7.69 (t, *J* = 56.1 Hz, 1 H), 7.45 (s, 1 H), 7.31 (dd, *J* = 5.3, 1.5 Hz, 1 H); ^{19}F NMR (376 MHz, $CDCl_3$) δ -96.51 (d, *J* = 56.2 Hz, 2 F); ^{13}C NMR (101 MHz, $CDCl_3$) δ 154.90 (t, *J* = 3.9 Hz), 150.42, 133.47, 126.74 (t, *J* = 2.2 Hz), 125.09, 120.75 (t, *J* = 271.9 Hz) ppm. MS (EI): 157 (100), 239, 241. HRMS (EI) for $C_6H_4BrF_2NS$ Calcd: 238.9216; Found: 238.9214. IR (KBr): ν = 3048, 2927, 1558, 1455, 1358, 1285, 1145, 1070, 823, 790, 768, 676 cm^{-1}.

2-((Difluoromethyl)thio)quinolone 4ap

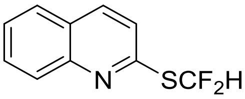

Prepared from 2-bromoquinoline (103.5 mg, 0.5 mmol) according to general procedure **C**. The crude residue was purified by flash column chromatography on silica gel to yield **2-((difluoromethyl)thio)quinolone 4ap** (105 mg, >99 %) as a pale yellow oil.

Prepared from 2-bromoquinoline (103.5`mg, 0.5 mmol) according to general procedure **D**. The crude residue was purified by flash column chromatography on silica gel to yield **2-((difluoromethyl)thio)quinolone 4ap** (88.5 mg, 84 %) as a pale yellow oil.

^{1}H NMR (400 MHz, $CDCl_3$) δ 8.05 (t, *J* = 56.0 Hz, 1 H), 8.01-7.97 (m, 2 H), 7.76-7.69 (m, 2 H), 7.53-7.49 (m, 1 H), 7.21 (d, *J* = 8.5 Hz, 1 H); ^{19}F NMR (376 MHz, $CDCl_3$) δ -97.49 (d, *J* = 56.0 Hz, 2 F); ^{13}C NMR (101 MHz, $CDCl_3$) δ 153.75 (t, *J* = 3.6 Hz), 148.17, 136.92, 130.34, 128.50, 127.75, 126.53, 126.49, 121.07 (t, *J* = 2.3 Hz), 121.30 (t, *J* = 270.1 Hz) ppm. MS (EI): 129.1 (100), 211. HRMS (EI) for $C_{10}H_7F_2NS$ Calcd: 211.0267; Found: 211.0272. IR (KBr): ν = 3062, 2959, 1616, 1591, 1559, 1498, 1422, 1139, 1097, 1064, 945, 816, 782, 749, 635, 474 cm^{-1}.

1-((Difluoromethyl)thio)isoquinoline 4v

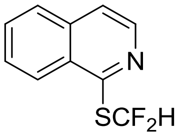

Prepared from 1-bromoisoquinoline (103.5 mg, 0.5 mmol) according to general procedure **C**. The crude residue was purified by flash column chromatography on silica gel to yield **1-((difluoromethyl)thio)isoquinoline 4v** (66 mg, 63 %) as a pale green yellow oil. (^{1}H, ^{19}F and ^{13}C NMR spectra are the same as those for the product in **Scheme 2, 4v**).

Methyl 3-((difluoromethyl)thio)pyrazine-2-carboxylate 4ag

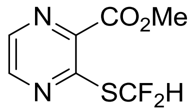

Prepared from methyl 3-bromopyrazine-2-carboxylate (108 mg, 0.5 mmol) according to general procedure **C**. The crude residue was purified by flash column chromatography on silica gel to yield **Methyl 3-((difluoromethyl)thio)pyrazine-2-carboxylate 4ag** (92 mg, 84 %) as a white solid. (^{1}H, ^{19}F and ^{13}C NMR spectra are the same as those for the product in **Scheme 2, 4ag**).

6-((Difluoromethyl)thio)pyrazine-2-carbonitrile 4aq

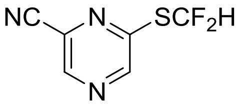

Prepared from 6-bromopyrazine-2-carbonitrile (91.5 mg, 0.5 mmol) according to general procedure **C**. The crude residue was purified by flash column chromatography on silica gel to yield **6-((difluoromethyl)thio)pyrazine-2-carbonitrile 4aq** (89 mg, 95 %) as a pale yellow oil. ^{1}H NMR (400 MHz, $CDCl_3$) δ 8.70 (d, J = 8.8 Hz, 2 H), 7.65 (t, J = 55.4 Hz, 1 H); ^{19}F NMR (376 MHz, $CDCl_3$) δ -96.54 (d, J = 55.4 Hz, 2 F); ^{13}C NMR (101 MHz, $CDCl_3$) δ 153.45 (t, J = 3.6 Hz), 146.91 (t, J = 2.2 Hz), 144.86, 129.87, 119.25 (t, J = 275.3 Hz), 114.61 ppm. MS (EI): 137.1 (100), 187. HRMS (EI) for $C_6H_3F_2N_3S$ Calcd: 187.0016; Found: 187.0018. IR (KBr): ν = 3050, 2963, 2242, 1723, 1516, 1395, 1295, 1243, 1191, 1133, 1074, 1007, 889, 779, 755, 453 cm^{-1}.

2-Chloro-5-((difluoromethyl)thio)pyrazine 4ar

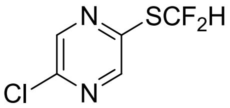

Prepared from 2-bromo-5-chloropyrazine (96 mg, 0.5 mmol) according to general procedure **C**. The crude residue was purified by flash column chromatography on silica gel to yield **2-chloro-5-((difluoromethyl)thio)pyrazine 4ar** (54 mg, 55 %) as a pale yellow oil. ^{1}H NMR (400 MHz, $CDCl_3$) δ 8.50 (s, 1 H), 8.36 (s, 1 H), 7.52 (t, J = 55.9 Hz, 1 H); ^{13}C NMR (101 MHz, $CDCl_3$) δ 148.55 (t, J = 3.8 Hz), 147.70, 144.43,

144.09 (t, J = 2.4 Hz), 120.05 (t, J = 274.7 Hz) ppm. MS (EI): 119 (100), 196. HRMS (EI) for $C_5H_3ClF_2N_2S$ Calcd: 195.9674; Found: 195.9677. IR (KBr): ν = 3068, 2927, 2854, 1446, 1292, 1155, 1070, 1016, 897, 843, 781, 660 cm^{-1}.

2-((Difluoromethyl)thio)-5-(methylthio)pyrazine 4as

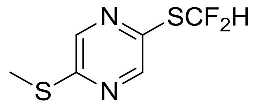

Prepared from 2-bromo-5-(methylthio)pyrazine (102 mg, 0.5 mmol) according to general procedure **C**. The crude residue was purified by flash column chromatography on silica gel to yield **2-((difluoromethyl)thio)-5-(methylthio)pyrazine 4as** (101 mg, 97 %) as a pale yellow oil. ^1H NMR (400 MHz, $CDCl_3$) δ 8.41 (d, J = 1.4 Hz, 1 H), 8.38 (d, J = 1.5 Hz, 1 H), 7.34 (t, J = 56.4 Hz, 1 H), 2.56 (s, 3 H); ^{19}F NMR (376 MHz, $CDCl_3$) δ -93.93 (d, J = 56.5 Hz, 2 F); ^{13}C NMR (101 MHz, $CDCl_3$) δ 157.01, 145.76 (t, J = 2.1 Hz), 143.26, 142.43 (t, J = 3.6 Hz), 120.62 (t, J = 274.8 Hz), 12.79 ppm. MS (EI): 208 (100). HRMS (EI) for $C_6H_6F_2N_2S_2$ Calcd: 207.9940; Found: 207.9945. IR (KBr): ν = 2930, 1538, 1493, 1448, 1280, 1162, 1068, 1015, 996, 895, 798, 782, 407 cm^{-1}.

2-((Difluoromethyl)thio)quinoxaline 4at

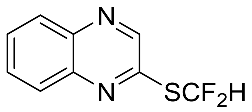

Prepared from 2-bromoquinoxaline (104 mg, 0.5 mmol) according to general procedure **C**. The crude residue was purified by flash column chromatography on silica gel to yield **2-((difluoromethyl)thio)quinoxaline 4at** (105 mg, 99 %) as a white solid.

Prepared from 2-bromoquinoxaline (104 mg, 0.5 mmol) according to general procedure **D**. The crude residue was purified by flash column chromatography on silica gel to yield **2-((difluoromethyl)thio)quinoxaline 4at** (100 mg, 94 %) as a white solid.

^1H NMR (400 MHz, $CDCl_3$) δ 8.62 (s, 1 H), 8.06-8.04 (m, 1 H), 7.97-7.95 (m, 1 H),

7.90 (t, J = 56.0 Hz, 1 H), 7.77-7.69 (m, 2 H); ^{19}F NMR (376 MHz, $CDCl_3$) δ -96.76 (d, J = 55.7 Hz, 2 F); ^{13}C NMR (101 MHz, $CDCl_3$) δ 150.52 (t, J = 3.4 Hz), 144.13 (t, J = 2.3 Hz), 142.34, 140.70, 130.93, 129.66, 129.44, 128.35, 120.41 (t, J = 272.6 Hz) ppm. MS (EI): 212.1 (100). HRMS (EI) for $C_9H_6F_2N_2S$ Calcd: 212.0220; Found: 212.0226. IR (KBr): ν = 3065, 1543, 1487, 1367, 1287, 1246, 1153, 1126, 1064, 963, 911, 781, 761, 597 cm^{-1}.

6-((Difluoromethyl)thio)-2,2'-bipyridine 4au

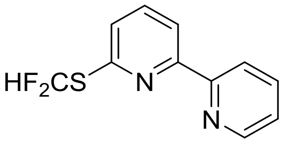

Prepared from 6-bromo-2,2'-bipyridine (117 mg, 0.5 mmol) according to general procedure **D**. The crude residue was purified by flash column chromatography on silica gel to yield **6-((difluoromethyl)thio)-2,2'-bipyridine 4au** (>99 %, determined by ^{19}F NMR analysis) as a white solid. The compound was further purified by prep-HPLC with a C18 column (Waters, Prep Nova-pak® HR C18, 19×300 mm，6 μ m) using a water-acetonitrile mixed solvent as the eluent (CH_3CN : H_2O = 60 : 40, flow = 10.0 mL/min, 254 nm). ^{1}H NMR (400 MHz, $CDCl_3$) δ 8.64 (d, J = 4.2 Hz, 1 H), 8.33 (d, J = 8.0 Hz, 1 H), 8.25 (dd, J = 7.8, 0.6 Hz, 1 H), 7.83 (t, J = 54.4 Hz, 1 H), 7.79 (td, J = 7.8, 1.8 Hz, 1 H), 7.69 (dd, J = 9.8, 5.9 Hz, 1 H), 7.29 (ddd, J = 7.5, 4.8, 1.1 Hz, 1 H), 7.22 (dd, J = 7.8, 0.6 Hz, 1 H); ^{19}F NMR (376 MHz, $CDCl_3$) δ -96.37 (d, J = 56.3 Hz, 2 F); ^{13}C NMR (101 MHz, $CDCl_3$) δ 156.49, 154.90, 152.60 (t, J = 3.7 Hz), 149.22, 138.06, 137.00, 124.21, 123.75 (t, J = 2.1 Hz), 121.37 (t, J = 270.8 Hz), 121.14, 118.79 ppm. MS (EI): 156 (100), 238. HRMS (EI) for $C_{11}H_8F_2N_2S$ Calcd: 238.0376; Found: 238.0367. IR (KBr): ν = 3060, 3012, 1578, 1556, 1419, 1279, 1146, 1070, 1036, 772, 740, 692 cm^{-1}. Mp: 72.3 – 73.7 ^{o}C.

General Procedure for Difluoromethylthiolation of Heteroaryl triflates

Method E

Heteroaryl triflates (0.5 mmol, 1.0 equiv), $Pd(dba)_2$ (30 mg, 10 mol%), XantPhos (44 mg, 15 mol%) (or $Pd(dba)_2$ (60mg, 20 mol%), XantPhos (88 mg, 30 mol%)), NaBr (103 mg, 2.0 equiv) and [(SIPr)Ag(SCF_2H)] (350 mg, 0.6 mmol, 1.2 equiv) were added in a 20 mL schlenk tube under argon. To the tube was added 2.5 mL of anhydrous toluene and the mixture was stirred at 50 ^oC for 12 h. The dark solution was diluted with Et_2O (15.0 mL). The mixture was filtered through a short plug of silica gel, washed with Et_2O (100 mL). The organic layer was combined, and concentrated under vacuum. The crude product was purified by column chromatography on silica gel with a mixed solvent of pentane/Et_2O or pentane/EtOAc as the eluent to give the product.

Method F

Heteroaryl triflates (0.5 mmol, 1.0 equiv), XantphosPd(3-py)Br (25 mg, 6.0 mol%), XantPhos (9.0 mg, 3.0 mol%), NaBr (103 mg, 2.0 equiv) and [(SIPr)Ag(SCF_2H)] (350 mg, 0.6 mmol, 1.2 equiv) were added in a 20 mL schlenk tube under argon. To the tube was added 5.0 mL of anhydrous toluene and the mixture was stirred at 50 ^oC for 6 h. The dark solution was diluted with Et_2O (15.0 mL). The mixture was filtered through a short plug of silica gel, washed with Et_2O (100 mL). The organic layer was combined, and concentrated under vacuum. The crude product was purified by column chromatography on silica gel with a mixed solvent of pentane/Et_2O or pentane/EtOAc as the eluent to give the product.

5-Bromo-2-((difluoromethyl)thio)pyridine 4a

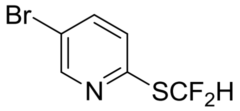

Prepared from 5-bromopyridin-2-yl trifluoromethanesulfonate (152.5 g, 0.5 mmol) according to general procedure **E** ($Pd(dba)_2$ (60 mg, 20 mol%) and XantPhos (88 mg, 30 mol%) was used). The crude residue was purified by flash column chromatography

on silica gel to yield **5-bromo-2-((difluoromethyl)thio)pyridine 4a** (60 mg, 50 %) as a colorless oil.

Prepared from 5-bromopyridin-2-yl trifluoromethanesulfonate (152.5 mg, 0.5 mmol) according to general procedure **F**. The crude residue was purified by flash column chromatography on silica gel to yield **5-bromo-2-((difluoromethyl)thio)pyridine 4a** (39 mg, 33 %) as a colorless oil.(^{1}H, ^{19}F and ^{13}C NMR spectra are the same as those for the product in **Scheme 2, 4a**).

5-Chloro-2-((difluoromethyl)thio)pyridine 4b

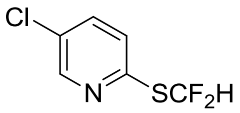

Prepared from 5-chloropyridin-2-yl trifluoromethanesulfonate (130.5 mg, 0.5 mmol) according to general procedure **E** ($Pd(dba)_2$ (60 mg, 20 mol%) and XantPhos (88 mg, 30 mol%) was used). The crude residue was purified by flash column chromatography on silica gel to yield **5-chloro-2-((difluoromethyl)thio)pyridine 4b** (46 mg, 47 %) as a colorless oil. (^{1}H, ^{19}F and ^{13}C NMR spectra are the same as those for the product in **Scheme 2, 4b**).

5-Bromo-2-((difluoromethyl)thio)-3-nitropyridine 4av

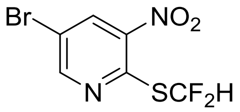

Prepared from 5-bromo-3-nitropyridin-2-yl trifluoromethanesulfonate (175 mg, 0.5 mmol) according to general procedure **E** ($Pd(dba)_2$ (60 mg, 20 mol%) and XantPhos (88 mg, 30 mol%) was used). The crude residue was purified by flash column chromatography on silica gel to yield **5-bromo-2-((difluoromethyl)thio)-3-nitropyridine 4av** (74 mg, 52 %) as a yellow oil. ^{1}H NMR (400 MHz, $CDCl_3$) δ 8.79 (d, *J* = 2.2 Hz, 1 H), 8.69 (d, *J* = 2.1 Hz, 1 H), 7.78 (t, *J* = 55.6 Hz, 1 H); ^{19}F NMR (376 MHz, $CDCl_3$) δ -101.98 (d, *J* = 55.7 Hz, 2 F); ^{13}C NMR (101 MHz, $CDCl_3$) δ 154.52, 152.06 (t, *J* = 5.1 Hz), 141.27, 136.24, 119.28 (t, *J* = 271.2 Hz), 116.73. MS (EI): 187 (100), 284, 286. HRMS (EI) for

$C_6H_3BrF_2N_2O_2S$ Calcd: 283.9067; Found: 283.9061. IR (KBr): ν = 3072, 1580, 1545, 1518, 1420, 1337, 1282, 1062, 912, 887, 765 cm^{-1}.

2-((Difluoromethyl)thio)quinoline 4ap

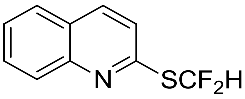

Prepared from quinoxalin-2-yl trifluoromethanesulfonate (139 mg, 0.5 mmol) according to general procedure **E** ($Pd(dba)_2$ (30 mg, 10 mol%) and XantPhos (44 mg, 15 mol%) was used). The crude residue was purified by flash column chromatography on silica gel to yield **2-((difluoromethyl)thio)quinoline 4ap** (91 mg, 86 %) as a pale yellow oil. (^{1}H, ^{19}F and ^{13}C NMR spectra are the same as those for the product in **Scheme 3, 4ap**).

7-Bromo-1-((difluoromethyl)thio)isoquinoline 4aw

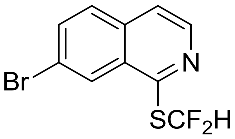

Prepared from 7-bromoisoquinolin-1-yl trifluoromethanesulfonate (177.5 mg, 0.5 mmol) according to general procedure **E** ($Pd(dba)_2$ (30 mg, 10 mol%) and XantPhos (44 mg, 15 mol%) was used). The crude residue was purified by flash column chromatography on silica gel to yield **7-bromo-1-((difluoromethyl)thio)isoquinoline 4aw** (68 mg, 47 %) as a white solid.

Prepared from 7-bromoisoquinolin-1-yl trifluoromethanesulfonate (177.5 mg, 0.5 mmol) according to general procedure **F**. The crude residue was purified by flash column chromatography on silica gel to yield **7-bromo-1-((difluoromethyl)thio)isoquinoline 4aw** (66 mg, 46 %) as a white solid.

^{1}H NMR (400 MHz, $CDCl_3$) δ 8.36 (d, *J* = 5.7 Hz, 1 H), 8.19-8.13 (m, 1 H), 7.97 (t, *J* = 56.1 Hz, 1 H), 7.77 (dd, *J* = 8.7, 1.8 Hz, 1 H), 7.67 (d, *J* = 8.7 Hz, 1 H), 7.44 (dd, *J* = 5.7, 0.5 Hz, 1 H); ^{19}F NMR (376 MHz, $CDCl_3$) δ -97.45 (d, *J* = 56.0 Hz, 2 F); ^{13}C NMR (101 MHz, $CDCl_3$) δ 153.67 (t, *J* = 3.7 Hz), 142.35, 134.51, 128.99, 127.88 (t, *J*

= 2.0 Hz), 126.68, 121.63, 118.83, 99.99 ppm. MS (EI): 207.1 (100), 289, 291. HRMS (EI) for $C_{10}H_6BrF_2NS$ Calcd: 288.9372; Found: 288.9373. IR (KBr): ν = 3053, 1577, 1545, 1487, 1274, 1072, 1045, 989, 845, 828, 785, 680 cm^{-1}. Mp: 96.7- 98.2 ^{o}C.

1-((Difluoromethyl)thio)isoquinoline 4v

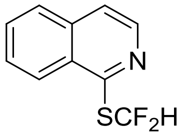

Prepared from isoquinolin-1-yl trifluoromethanesulfonate (138.5 mg, 0.5 mmol) according to general procedure **E** ($Pd(dba)_2$ (30 mg, 10 mol%) and XantPhos (44 mg, 15 mol%) was used). The crude residue was purified by flash column chromatography on silica gel to yield **1-((difluoromethyl)thio)isoquinoline 4v** (58 mg, 55 %) as a pale green yellow oil. (^{1}H, ^{19}F and ^{13}C NMR spectra are the same as those for the product in **Scheme 2, 4v**).

5-Bromo-2-((difluoromethyl)thio)pyrimidine 4ac

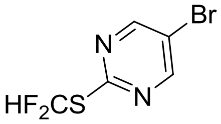

Prepared from 5-bromopyrimidin-2-yl trifluoromethanesulfonate (153 mg, 0.5 mmol) according to general procedure **E** ($Pd(dba)_2$ (60 mg, 20 mol%) and XantPhos (88 mg, 30 mol%) was used). The crude residue was purified by flash column chromatography on silica gel to yield **5-bromo-2-((difluoromethyl)thio)pyrimidine 4ac** (48 mg, 40 %) as a pale yellow oil. (^{1}H, ^{19}F and ^{13}C NMR spectra are the same as those for the product in **Scheme 2, 4ac**).

2-((Difluoromethyl)thio)quinoxaline 4at

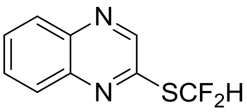

Prepared from quinoxalin-2-yl trifluoromethanesulfonate (139 mg, 0.5 mmol) according to general procedure **E** ($Pd(dba)_2$ (30 mg, 10 mol%) and XantPhos (44 mg, 15 mol%) was used). The crude residue was purified by flash column chromatography

on silica gel to yield **2-((difluoromethyl)thio)quinoxaline 4at** (90 mg, 85 %) as a white solid.

Prepared from quinoxalin-2-yl trifluoromethanesulfonate (139 mg, 0.5 mmol) according to general procedure **F**. The crude residue was purified by flash column chromatography on silica gel to yield **2-((difluoromethyl)thio)quinoxaline 4at** (64 mg, 60 %) as a white solid. (^{1}H, ^{19}F and ^{13}C NMR spectra are the same as those for the product in **Scheme 3, 4at**).

General Procedure for Difluoromethylthiolation of Aryl Iodide

Method G

Aryl iodide (0.5 mmol, 1.0 equiv), $Pd(dba)_2$, DPEPhos, and $[(SIPr)Ag(SCF_2H)]$ (350.0 mg, 0.6 mmol, 1.2 equiv) were added in a 20.0 mL schlenk tube under argon. To the tube was added 2.5 mL of anhydrous toluene and the mixture was stirred at 50 ^{o}C for 12 h. The dark solution was diluted with Et_2O (15.0 mL). The mixture was filtered through a short plug of silica gel, washed with Et_2O (100.0 mL). The organic layer was combined, and concentrated under vacuum. The crude product was purified by column chromatography on silica gel with a mixed solvent of pentane/Et_2O or pentane/EtOAc as the eluent to give the product.

[1,1'-Biphenyl]-4-yl(difluoromethyl)sulfane 5a[2]

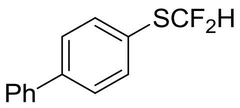

Prepared from 4-iodo-1,1'-biphenyl (140.0 mg, 0.5 mmol) according to general procedure **G** ($Pd(dba)_2$ (30.0 mg, 10.0 mol%) and DPEPhos (27.0 mg, 10.0 mol%) was used). The crude residue was purified by flash column chromatography on silica gel to yield **[1,1'-biphenyl]-4-yl(difluoromethyl)sulfane 5a** (116.0 mg, 98 %) as a white solid. ^{1}H NMR (400 MHz, $CDCl_3$) δ 7.70-7.61 (m, 6 H), 7.50 (t, *J* = 7.5 Hz, 2 H), 7.42 (t, *J* = 7.3 Hz, 1 H), 6.89 (t, *J* = 56.9 Hz, 1 H). ^{19}F NMR (376 MHz, $CDCl_3$) δ -91.24 (d, *J* = 56.9 Hz, 2 F); ^{13}C NMR (101 MHz, $CDCl_3$) δ 142.85, 139.90, 135.79, 129.00, 128.09, 128.03, 127.21, 124.84 (t, *J* = 2.9 Hz), 121.02 (t, *J* = 275.3 Hz) ppm.

(4-(*tert*-Butyl)phenyl)(difluoromethyl)sulfane 5b[2]

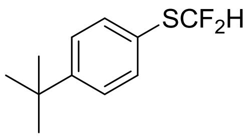

Prepared from 1-(*tert*-butyl)-4-iodobenzene (130.0 mg, 0.5 mmol) according to general procedure **G** ($Pd(dba)_2$ (30.0 mg, 10.0 mol%) and DPEPhos (27.0 mg, 10.0 mol%) was used). The crude residue was purified by flash column chromatography on

silica gel to yield **(4-(*tert*-butyl)phenyl)(difluoromethyl)sulfane 5b** (90.0 mg, 83 %) as a colorless oil. ^{1}H NMR (400 MHz, $CDCl_3$) δ 7.52-7.50 (m, 2 H), 7.43-7.40 (m, 2 H), 6.81 (t, *J* = 57.2 Hz, 1 H), 1.33 (s, 9 H); ^{19}F NMR (376 MHz, $CDCl_3$) δ -91.43 (d, *J* = 57.2 Hz, 2 F); ^{13}C NMR (101 MHz, $CDCl_3$) δ 153.26, 135.23, 126.49, 122.55 (t, *J* = 3.0 Hz), 121.22 (t, *J* = 274.9 Hz), 34.77, 31.18 ppm.

[1,1'-Biphenyl]-2-yl(difluoromethyl)sulfane 5c[2]

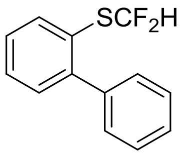

Prepared from 2-iodo-1,1'-biphenyl (140.0 mg, 0.5 mmol) according to general procedure **G** ($Pd(dba)_2$ (30.0 mg, 10.0 mol%) and DPEPhos (27.0 mg, 10.0 mol%) was used). The crude residue was purified by flash column chromatography on silica gel to yield **[1,1'-Biphenyl]-2-yl(difluoromethyl)sulfane 5c** (86.0 mg, 73 %) as a colorless oil. ^{1}H NMR (400 MHz, $CDCl_3$) δ 7.75-7.73 (m, 1 H), 7.50-7.37 (m, 8 H), 6.68 (td, *J* = 57.0, 2.2 Hz, 1 H); ^{19}F NMR (376 MHz, $CDCl_3$) δ -91.39 (dd, *J* = 56.9, 1.9 Hz, 2 F); ^{13}C NMR (101 MHz, $CDCl_3$) δ 146.51, 140.41, 135.47, 131.08, 129.66, 129.53, 128.26, 128.05, 127.69, 125.43 (t, *J* = 3.0 Hz), 121.00 (t, *J* = 275.0 Hz) ppm.

(Difluoromethyl)(3,4,5-trimethoxyphenyl)sulfane 5d

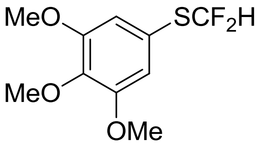

Prepared from 5-iodo-1,2,3-trimethoxybenzene (147.0 mg, 0.5 mmol) according to general procedure **G** ($Pd(dba)_2$ (30.0 mg, 10.0 mol%) and DPEPhos (27.0 mg, 10.0 mol%) was used). The crude residue was purified by flash column chromatography on silica gel to yield **(difluoromethyl)(3,4,5-trimethoxyphenyl)sulfane 5d** (80.0 mg, 64 %) as a pale yellow oil. ^{1}H NMR (400 MHz, $CDCl_3$) δ 6.82 (t, *J* = 57.1 Hz, 1 H), 6.80 (s, 2 H), 3.86 (s, 6 H), 3.86 (s, 3 H); ^{19}F NMR (376 MHz, $CDCl_3$) δ -91.50 (d, *J* = 57.1 Hz, 2 F); ^{13}C NMR (101 MHz, $CDCl_3$) δ 153.42, 139.70, 121.14 (t, *J* = 275.3 Hz), 120.25 (t, *J* = 3.2 Hz), 112.77, 60.88, 56.28 ppm. MS (EI): 250 (100). HRMS (EI)

for $C_{10}H_{12}F_2O_3S$ Calcd: 250.0475, Found: 250.0477. IR (KBr): ν = 3158, 3095, 2940, 2227, 1582, 1432, 1407, 1311, 1234, 1177, 1129, 1067, 925, 879, 833 cm^{-1}.

(Difluoromethyl)(naphthalen-1-yl)sulfane 5e[2]

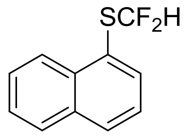

Prepared from 1-iodonaphthalene (127.0 mg, 0.5 mmol) according to general procedure **G** ($Pd(dba)_2$ (30.0 mg, 10.0 mol%) and DPEPhos (27.0 mg, 10.0 mol%) was used). The crude residue was purified by flash column chromatography on silica gel to yield **(difluoromethyl)(naphthalen-1-yl)sulfane 5e** (101.0 mg, 96 %) as a colorless oil. ^{1}H NMR (400 MHz, $CDCl_3$) δ 8.57 (d, *J* = 8.5 Hz, 1 H), 7.98 (d, *J* = 8.3 Hz, 1 H), 7.94 – 7.90 (m, 2 H), 7.66 (ddd, *J* = 8.4, 6.9, 1.4 Hz, 1 H), 7.59 (ddd, *J* = 8.1, 6.9, 1.2 Hz, 1 H), 7.50 (dd, *J* = 8.2, 7.2 Hz, 1 H), 6.86 (t, *J* = 57.1 Hz, 1 H); ^{19}F NMR (376 MHz, $CDCl_3$) δ -90.65 (d, *J* = 57.1 Hz, 2 F); ^{13}C NMR (101 MHz, $CDCl_3$) δ 136.36, 135.23, 134.26, 131.29, 128.59, 127.47, 126.66, 125.81, 125.62, 123.43 (t, *J* = 3.0 Hz), 121.36 (t, *J* = 275.9 Hz) ppm.

(Difluoromethyl)(9*H*-fluoren-2-yl)sulfane 5f

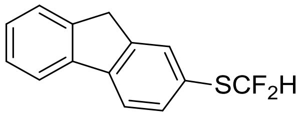

Prepared from 2-iodo-9*H*-fluorene (146.0 mg, 0.5 mmol) according to general procedure **G** ($Pd(dba)_2$ (30.0 mg, 10.0 mol%) and DPEPhos (27.0 mg, 10.0 mol%) was used). The crude residue was purified by flash column chromatography on silica gel to yield **(difluoromethyl)(9*H*-fluoren-2-yl)sulfane 5f** (99.0 mg, 80 %) as a white solid. ^{1}H NMR (400 MHz, $CDCl_3$) δ 7.79 (t, *J* = 8.2 Hz, 2 H), 7.76 (s, 1 H), 7.61 (d, *J* = 7.5 Hz, 1 H), 7.57 (d, *J* = 7.5 Hz, 1 H), 7.42 (t, *J* = 7.3 Hz, 1 H), 7.39 (d, *J* = 7.3 Hz, 1 H), 6.88 (t, *J* = 57.1 Hz, 1 H), 3.90 (s, 2 H); ^{19}F NMR (376 MHz, $CDCl_3$) δ -91.40 (d, *J* = 57.0 Hz, 2 F); ^{13}C NMR (101 MHz, $CDCl_3$) δ 144.31, 143.54, 143.51, 140.51,

134.28, 132.23, 127.68, 127.03, 125.19, 123.49 (t, *J* = 3.0 Hz), 121.24 (t, *J* = 275.2 Hz), 120.50, 120.43, 36.78 ppm. MS (EI): 165.1 (100), 248. HRMS (EI) for $C_{14}H_{10}F_2S$ Calcd: 248.0471, Found: 248.0469. IR (KBr): ν = 3065, 2966, 2896, 2788, 1951, 1901, 1804, 1603, 1466, 1450, 1410, 1319, 1295, 1067, 1029, 955, 875, 831, 792 cm^{-1}. Mp: 42.0 - 43.1 ^oC.

(Difluoromethyl)(phenanthren-9-yl)sulfane 5g

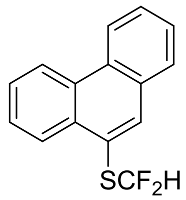

Prepared from 9-iodophenanthrene (152.0 mg, 0.5 mmol) according to general procedure **G** ($Pd(dba)_2$ (30.0 mg, 10.0 mol%) and DPEPhos (27.0 mg, 10.0 mol%) was used). The crude residue was purified by flash column chromatography on silica gel to yield **(difluoromethyl)(phenanthren-9-yl)sulfane 5g** (130.0 mg, >99 %) as a white solid. ^1H NMR (400 MHz, $CDCl_3$) δ 8.72-8.63 (m, 3 H), 8.23 (s, 1 H), 7.89 (d, *J* = 7.2 Hz, 1 H), 7.76-7.70 (m, 3 H), 7.66-7.62 (m, 1 H), 6.91 (t, *J* = 57.1 Hz, 1 H); ^{19}F NMR (376 MHz, $CDCl_3$) δ -90.65 (d, *J* = 57.1 Hz, 2 F); ^{13}C NMR (101 MHz, $CDCl_3$) δ 138.24, 132.62, 131.34, 131.22, 131.14, 128.99, 128.38, 127.45, 127.36, 127.24, 126.73, 123.07, 122.71, 122.36 (t, *J* = 2.9 Hz), 121.46 (t, *J* = 276.0 Hz) ppm. MS (EI): 165.1 (100), 260. HRMS (EI) for $C_{15}H_{10}F_2S$ Calcd: 260.0471, Found: 260.0467. IR (KBr): ν = 3075, 3060, 2968, 1588, 1507, 1450, 1296.9, 1244, 1066, 1033, 942, 899, 856, 795 cm^{-1}. Mp: 47.6 – 49.1 ^oC.

(3-(Benzyloxy)phenyl)(difluoromethyl)sulfane 5h

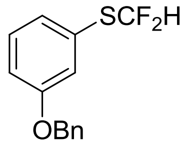

Prepared from 1-(benzyloxy)-3-iodobenzene (155.0 mg, 0.5 mmol) according to general procedure **G** ($Pd(dba)_2$ (30.0 mg, 10.0 mol%) and DPEPhos (27.0 mg, 10.0 mol%) was used). The crude residue was purified by flash column chromatography on

silica gel to yield **(3-(benzyloxy)phenyl)(difluoromethyl)sulfane 5h** (125.0 mg, 94 %) as a pale yellow oil. ^{1}H NMR (400 MHz, $CDCl_3$) δ 7.48-7.31 (m, 6 H), 7.24-7.20 (m, 2 H), 7.06 (ddd, *J* = 8.3, 2.5, 0.9 Hz, 1 H), 6.86 (t, *J* = 57.0 Hz, 1 H), 5.09 (s, 2 H); ^{19}F NMR (376 MHz, $CDCl_3$) δ -91.04 (d, *J* = 57.1 Hz, 2 F); ^{13}C NMR (101 MHz, $CDCl_3$) δ 159.14, 136.43, 130.24, 128.72, 128.24, 127.62, 127.53, 127.29 (t, *J* = 3.0 Hz), 121.19 (t, *J* = 276.7 Hz), 116.59, 70.24 ppm. MS (EI): 91.1 (100), 266. HRMS (EI) for $C_{14}H_{12}F_2OS$ Calcd: 266.0577, Found: 266.0576. IR (KBr): ν = 3066.0, 3034, 2931, 2872, 1590, 1497, 1455, 1419, 1381, 1318, 1286, 1231, 1067, 892, 854, 796 cm^{-1}.

(Difluoromethyl)(4-nitrophenyl)sulfane 5i[2]

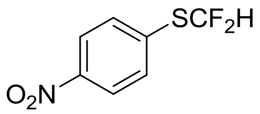

Prepared from 1-iodo-4-nitrobenzene (124.5 mg, 0.5 mmol) according to general procedure **G** ($Pd(dba)_2$ (15.0 mg, 5.0 mol%) and DPEPhos (13.5 mg, 5.0 mol%) was used). The crude residue was purified by flash column chromatography on silica gel to yield **(difluoromethyl)(4-nitrophenyl)sulfane 5i** (103 mg, >99 %) as a pale yellow oil. ^{1}H NMR (400 MHz, $CDCl_3$) δ 8.24-8.20 (m, 1 H), 7.73-7.70 (m, 1 H), 6.95 (t, *J* = 55.9 Hz, 1 H); ^{19}F NMR (376 MHz, $CDCl_3$) δ -91.25 (d, *J* = 55.9 Hz, 2 F); ^{13}C NMR (101 MHz, $CDCl_3$) δ 148.30, 135.02 (t, *J* = 2.8 Hz), 134.34, 124.18, 119.66 (t, *J* = 276.7 Hz) ppm.

(Difluoromethyl)(2-nitrophenyl)sulfane 5j[2]

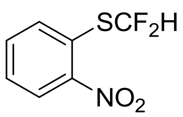

Prepared from 1-iodo-2-nitrobenzene (124.5 mg, 0.5 mmol) according to general procedure **G** ($Pd(dba)_2$ (22.5 mg, 7.5 mol%) and DPEPhos (20.3 mg, 7.5 mol%) was used). The crude residue was purified by flash column chromatography on silica gel to yield **(difluoromethyl)(2-nitrophenyl)sulfane 5j** (96.0 mg, 94 %) as a pale yellow oil. ^{1}H NMR (400 MHz, $CDCl_3$) δ 8.09 (dd, *J* = 8.2, 1.3 Hz, 1 H), 7.73 (dd, *J* = 8.0,

1.1 Hz, 1 H), 7.64 (td, J = 7.8, 1.4 Hz, 1 H), 7.52-7.48 (m, 1 H), 6.99 (t, J = 55.9 Hz, 1 H); ^{19}F NMR (376 MHz, $CDCl_3$) δ -92.95 (d, J = 55.9 Hz, 2 F); ^{13}C NMR (101 MHz, $CDCl_3$) δ 149.33, 133.65, 131.97, 128.69, 126.08 (t, J = 3.0 Hz), 125.72, 119.97 (t, J = 275.2 Hz) ppm.

(Difluoromethyl)(3-fluoro-5-nitrophenyl)sulfane 5k

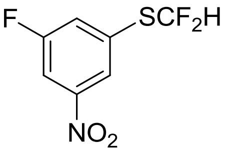

Prepared from 1-fluoro-3-iodo-5-nitrobenzene (133.5 mg, 0.5 mmol) according to general procedure **G** ($Pd(dba)_2$ (30 mg, 10.0 mol%) and DPEPhos (27.0 mg, 10.0 mol%) was used). The crude residue was purified by flash column chromatography on silica gel to yield **(difluoromethyl)(3-fluoro-5-nitrophenyl)sulfane 5k** (76.0 mg, 68 %) as a pale yellow oil. ^{1}H NMR (400 MHz, $CDCl_3$) δ 8.25 (s, 1 H), 8.01 – 7.98 (m, 1 H), 7.66 (dd, J = 7.5, 1.5 Hz, 1 H), 6.94 (t, J = 55.6 Hz, 1 H); ^{19}F NMR (376 MHz, $CDCl_3$) δ -91.56 (d, J = 55.6 Hz, 2 F), -106.90 (t, J = 7.8 Hz, 1 F); ^{13}C NMR (101 MHz, $CDCl_3$) δ 161.93 (d, J = 255.6 Hz), 149.14 – 148.98 (m), 129.94 (dt, J = 8.3, 3.1 Hz), 127.72 (d, J = 22.7 Hz), 125.28 (dd, J = 2.6, 1.0 Hz), 119.17 (t, J = 277.7 Hz), 112.64 (d, J = 26.3 Hz) ppm. MS (EI): 223 (100). HRMS (EI) for $C_7H_4F_3NO_2S$ Calcd: 222.9915, Found: 222.9918. IR (KBr): ν = 3097, 2880, 1614, 1587, 1539, 1425, 1354, 1321, 1248, 1074, 1043, 956, 885, 789 cm^{-1}.

(3,4-Dichlorophenyl)(difluoromethyl)sulfane 5l

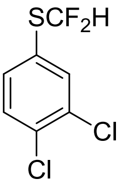

Prepared from 1,2-dichloro-4-iodobenzene (136.0 mg, 0.5 mmol) according to general procedure **G** ($Pd(dba)_2$ (30 mg, 10.0 mol%) and DPEPhos (27.0 mg, 10.0 mol%) was used). The crude residue was purified by flash column chromatography on silica gel to yield **(3,4-Dichlorophenyl)(difluoromethyl)sulfane 5l** (65.0 mg, 57 %) as a pale

yellow oil. ^{1}H NMR (400 MHz, $CDCl_3$) δ 7.69 (d, *J* = 2.0 Hz, 1 H), 7.47 (d, *J* = 8.3 Hz, 1 H), 7.42 (dd, *J* = 8.3, 2.0 Hz, 1 H), 6.83 (t, *J* = 56.4 Hz, 1 H); ^{19}F NMR (376 MHz, $CDCl_3$) δ = -91.57 (d, *J*=56.4, 2 F); ^{13}C NMR (101 MHz, $CDCl_3$) δ 136.76, 134.85, 134.46, 133.35, 131.08, 125.57 (t, *J* = 3.1 Hz), 119.92 (t, *J* = 276.5 Hz) ppm. MS (EI): 178(100), 228. HRMS (EI) for $C_7H_4Cl_2F_2S$ Calcd: 227.9379, Found: 227.9386. IR (KBr): ν 2955, 2925, 2854, 1581, 1459, 1367, 1318, 1297, 1130, 1077, 1035, 882, 815, 752, 696, 555 cm^{-1}.

(3-Bromophenyl)(difluoromethyl)sulfane 5m

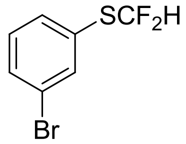

Prepared from 1-bromo-3-iodobenzene (141.0 mg, 0.5 mmol) according to general procedure **G** (Pd(dba)$_2$ (30 mg, 10.0 mol%) and DPEPhos (27.0 mg, 10.0 mol%) was used). The crude residue was purified by flash column chromatography on silica gel to yield **(3-Bromophenyl)(difluoromethyl)sulfane 5m** (96.0 mg, 81 %) as a colorless oil. ^{1}H NMR (400 MHz, $CDCl_3$) δ 7.75 (t, *J* = 1.8 Hz, 1 H), 7.56 (ddd, *J* = 8.0, 1.9, 1.0 Hz, 1H), 7.52 (d, *J* = 7.8 Hz, 1H), 7.27 (t, *J* = 7.9 Hz, 1 H), 6.84 (t, *J* = 56.6 Hz, 1 H); ^{19}F NMR (376 MHz, $CDCl_3$) δ -91.37 (d, *J* = 56.6 Hz, 2 F); ^{13}C NMR (101 MHz, $CDCl_3$) δ 137.64, 133.74, 132.92, 130.60, 127.94 (t, *J* = 3.0 Hz), 122.84, 120.38 (t, *J* = 276.0 Hz) ppm. MS (EI): 238(100). HRMS (EI) for $C_7H_5BrF_2S$ Calcd: 237.9263, Found: 237.9265.

(4-Bromophenyl)(difluoromethyl)sulfane 5n[2]

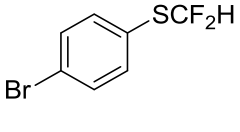

Prepared from 1-bromo-4-iodobenzene (141.0 mg, 0.5 mmol) according to general procedure **G** (Pd(dba)$_2$ (30 mg, 10.0 mol%) and DPEPhos (27.0 mg, 10.0 mol%) was used). The crude residue was purified by flash column chromatography on silica gel to yield **(4-Bromophenyl)(difluoromethyl)sulfane 5n** (55.0 mg, 46 %) as a colorless

oil. ^{1}H NMR (400 MHz, $CDCl_3$) δ 7.55-7.51 (m, 2 H), 7.46-7.43 (m, 2 H), 6.81 (t, J = 56.6 Hz, 1 H); ^{19}F NMR (376 MHz, $CDCl_3$) δ -91.60 (d, J = 56.6 Hz, 2 F); ^{13}C NMR (101 MHz, $CDCl_3$) δ 136.93, 132.59, 124.88 (t, J = 3.0 Hz), 124.75, 120.25 (t, J = 275.8 Hz) ppm.

1-(4-((Difluoromethyl)thio)phenyl)ethanone 5o[2]

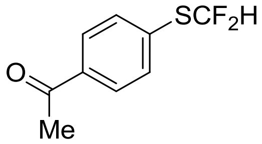

Prepared from 1-(4-iodophenyl)ethanone (123.0 mg, 0.5 mmol) according to general procedure **G** (Pd(dba)$_2$ (22.5 mg, 7.5 mol%) and DPEPhos (20.3 mg, 7.5 mol%) was used). The crude residue was purified by flash column chromatography on silica gel to yield **1-(4-((difluoromethyl)thio)phenyl)ethanone 5o** (91.0 mg, 90 %) as a pale yellow oil. ^{1}H NMR (400 MHz, $CDCl_3$) δ 7.94-7.91 (m, 2 H), 7.63-7.60 (m, 2 H), 6.89 (t, J = 56.4 Hz, 1 H); ^{19}F NMR (376 MHz, $cdcl_3$) δ -91.19 (d, J = 56.4 Hz, 2 F); ^{13}C NMR (101 MHz, $CDCl_3$) δ 197.14, 137.49, 134.13, 132.37 (t, J = 2.7 Hz), 128.98, 120.30 (t, J = 275.8 Hz), 26.63 ppm.

Methyl 2-((difluoromethyl)thio)benzoate 5p[2]

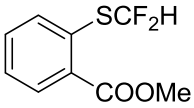

Prepared from methyl 2-iodobenzoate (131.0 mg, 0.5 mmol) according to general procedure **G** (Pd(dba)$_2$ (30 mg, 10.0 mol%) and DPEPhos (27.0 mg, 10.0 mol%) was used). The crude residue was purified by flash column chromatography on silica gel to yield **Methyl 2-((difluoromethyl)thio)benzoate 5p** (65.0 mg, 60 %) as a pale yellow oil. ^{1}H NMR (400 MHz, $CDCl_3$) δ 7.92 (dd, J = 7.8, 1.3 Hz, 1 H), 7.62 (d, J = 7.9 Hz, 1 H), 7.51 (td, J = 7.7, 1.4 Hz, 1 H), 7.40 (t, J = 7.6 Hz, 1 H), 7.00 (t, J = 56.7 Hz, 1 H), 3.94 (s, 3 H); ^{19}F NMR (376 MHz, $CDCl_3$) δ -92.72 (d, J = 56.7 Hz, 2 F); ^{13}C NMR (101 MHz, $CDCl_3$) δ 166.82, 132.42, 132.35, 132.17, 130.93, 130.40 (t, J = 3.4 Hz), 127.94, 120.98 (t, J = 273.5 Hz), 52.53 ppm.

Methyl 4-((difluoromethyl)thio)benzoate 5q[2]

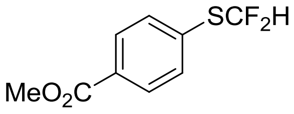

Prepared from methyl 4-iodobenzoate (131.0 mg, 0.5 mmol) according to general procedure **G** ($Pd(dba)_2$ (30 mg, 10.0 mol%) and DPEPhos (27.0 mg, 10.0 mol%) was used). The crude residue was purified by flash column chromatography on silica gel to yield **Methyl 4-((difluoromethyl)thio)benzoate 5q** (106.0 mg, 97 %) as a pale yellow oil. ^{1}H NMR (400 MHz, $CDCl_3$) δ 8.03-8.01 (m, 2 H), 7.62-7.59 (m, 2 H), 6.89 (t, J = 56.4 Hz, 1 H), 3.92 (s, 3 H); ^{19}F NMR (376 MHz, $CDCl_3$) δ -91.12 (d, J = 56.4 Hz, 2 F); ^{13}C NMR (101 MHz, $CDCl_3$) δ 166.24, 134.00, 132.19 (t, J = 3.0 Hz), 130.99, 130.30, 120.39 (t, J = 275.7 Hz), 52.39 ppm.

4-((Difluoromethyl)thio)benzonitrile 5r[2]

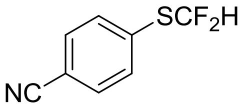

Prepared from 4-iodobenzonitrile (114.5 mg, 0.5 mmol) according to general procedure **G** ($Pd(dba)_2$ (22.5 mg, 7.5 mol%) and DPEPhos (20.3 mg, 7.5 mol%) was used). The crude residue was purified by flash column chromatography on silica gel to yield **4-((Difluoromethyl)thio)benzonitrile 5r** (75.0 mg, 81 %) as a pale yellow oil. ^{1}H NMR (400 MHz, $CDCl_3$) δ 7.66 (s, 4 H), 6.91 (t, J = 56.0 Hz, 1 H); ^{19}F NMR (376 MHz, $CDCl_3$) δ -91.19 (d, J = 56.0 Hz, 2 F); ^{13}C NMR (101 MHz, $CDCl_3$) δ 134.54, 132.78 (d, J = 2.9 Hz), 132.73, 119.77 (t, J = 276.6 Hz), 117.94, 113.29 ppm.

Procedure for Difluoromethylthiolation of 4-bromo-1-*iso*butyl-1*H*-imidazo[4,5-*c*] quinolone 6

4-Bromo-1-*iso*butyl-1*H*-imidazo[4,5-*c*] quinoline (152 mg, 0.5 mmol, 1.0 equiv), $Pd(dba)_2$ (60 mg, 20 mol%), XantPhos (88 mg, 30 mol%), and [(SIPr)Ag(SCF_2H)] (350 mg, 0.6 mmol, 1.2 equiv) were added in a 20 mL schlenk tube under argon. To the tube was added 2.5 mL of anhydrous toluene and the mixture was stirred at 80 ^{o}C for 12 h. The dark solution was diluted with Et_2O (15.0 mL). The mixture was filtered through a short plug of silica gel, washed with Et_2O (100 mL). The organic layer was combined, and concentrated under vacuum. The crude product was purified by column chromatography on silica gel with a mixed solvent of pentane/EtOAc as the eluent to give the product.

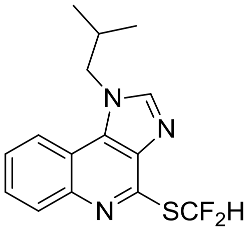

4-((Difluoromethyl)thio)-1-isobutyl-1H-imidazo[4,5-c]quinolone 6. ^{1}H NMR (400 MHz, $CDCl_3$) δ 8.31 (t, *J* = 56.0 Hz, 1 H), 8.04 (d, *J* = 8.3 Hz, 1 H), 7.91 (d, *J* = 8.1 Hz, 1 H), 7.77 (s, 1 H), 7.58 (t, *J* = 7.6 Hz, 1 H), 7.51 (t, *J* = 7.6 Hz, 1 H), 4.24 (d, *J* = 7.4 Hz, 2 H), 2.31 – 2.20 (m, 1 H), 0.99 (d, *J* = 6.6 Hz, 6 H); ^{19}F NMR (376 MHz, $CDCl_3$) δ -98.10 (d, *J* = 56.1 Hz, 2 F); ^{13}C NMR (101 MHz, $CDCl_3$) δ 148.42 (t, *J* = 3.7 Hz), 144.50, 143.51, 135.47, 131.90, 129.82, 127.59, 126.17, 120.97 (t, *J* = 269.1 Hz), 120.08, 116.83, 55.13, 28.79, 19.72 ppm. MS (EI): 225.1 (100), 307. HRMS (EI) for $C_{15}H_{15}F_2N_3S$ Calcd: 307.0955; Found: 307.0958. IR (KBr): ν 2962, 2886, 1569, 1513, 1444, 1356, 1214, 1143, 1061, 1041, 1017, 927, 777, 755, 642 cm^{-1}. Mp: 143.4 – 145.6 ^{o}C.

Procedure for Difluoromethylthiolation of heptan-2-yl 2-((5-chloro-3-iodo quinolin-8-yl)oxy)acetate 7

Heptan-2-yl 2-((5-chloro-3-iodo quinolin-8-yl)oxy)acetate (230 mg, 0.5 mmol, 1.0 equiv), $Pd(dba)_2$ (30 mg, 15 mol%), XantPhos (44 mg, 15 mol%), and [(SIPr)Ag(SCF_2H)] (350 mg, 0.6 mmol, 1.2 equiv) were added in a 20 mL schlenk tube under argon. To the tube was added 2.5 mL of anhydrous toluene and the mixture was stirred at 50 ^oC for 12 h. The dark solution was diluted with Et_2O (15.0 mL). The mixture was filtered through a short plug of silica gel, washed with Et_2O (100 mL). The organic layer was combined, and concentrated under vacuum. The crude product was purified by column chromatography on silica gel with a mixed solvent of pentane/EtOAc as the eluent to give the product.

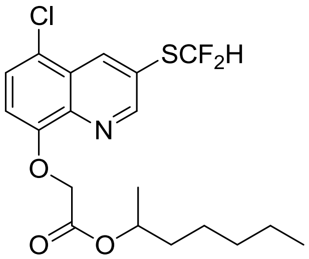

Heptan-2-yl 2-((5-chloro-3-((difluoromethyl)thio)quinolin-8-yl)oxy)acetate 7. ^1H NMR (400 MHz, $CDCl_3$) δ 8.98 (d, *J* = 2.1 Hz, 1 H), 8.70 (d, *J* = 2.1 Hz, 1 H), 7.48 (d, *J* = 8.4 Hz, 1 H), 6.91 (s, 1 H), 6.89 (t, *J* = 48.5 Hz, 1H), 5.01 – 4.93 (m, 1 H), 4.89 (s, 2 H), 1.54 – 1.36 (m, 2 H), 1.29 (t, *J* = 6.5 Hz, 1 H), 1.20 – 1.14 (m, 8 H), 0.80 – 0.77 (m, 3 H); ^{19}F NMR (376 MHz, $CDCl_3$) δ -91.11 (d, *J* = 56.2 Hz, 2 F); ^{13}C NMR (101 MHz, $CDCl_3$) δ 167.87, 153.71, 152.93, 140.26, 139.94, 127.25, 126.89, 123.11, 121.98 (t, *J* = 2.7 Hz), 119.60 (t, *J* = 277.3 Hz), 110.90, 72.82, 66.39, 35.64, 31.44, 24.88, 22.45, 19.85, 13.91 ppm. MS (EI): 274 (100), 417. HRMS (EI) for $C_{19}H_{22}ClF_2NO_3S$ Calcd: 417.0977; Found: 417.0972. IR (KBr): ν 2958, 2933, 2861, 1753, 1606, 1578, 1481, 1366, 1314, 1212, 1159, 1120, 1070, 963, 851, 793, 753, 643 cm^{-1}.

Reference

[1] Y. Gu, Q. Shen, *Nat. Commun*. **2014**, *5*, 5405, doi: 10.1038/ncomms6405.

[2] J. Wu, Y. Gu, X. Leng, and Q. Shen, *Angew. Chem. Int. Ed.* **2015**, *54*, 7648.

[3] L. Racane, H. Cicak, Z. Mihalic, G. Karminski-Zamola, V. Tralic-Kulenovic, *Tetrahedron* **2011**, 67, 2760.

[4] A. Klapars, S. L. Buchwald, *J. Am. Chem. Soc.* **2002**, *124*, 14844.

[5] C. -H. Wang, X. -T. Liu, X. -H. Chao, *Synthesis*, 1982 , *10*, 858.

[6] C. N. Johnson, S. F. Moss, D. R. Witty, WO2005030724A1, **2005**.

[7] E. Kiselev, K. Agama, Y. Pommier, M. Cushman, *J. Med. Chem.* **2012** , *55*,1682.

[8] B. Bradshaw, A. Dinsmore, D. Collison, C. D. Garner, J. A. Joule, *J. Chem. Soc*., *Perkin Trans. 1*, **2001**, *24*, 3232.

[9] B. Bayarmagnai, C. Matheis, K. Jouvin, L. J. Goossen, *Angew. Chem. Int. Ed.* **2015**, *54*, 5753.

Spectrum of the Starting Material

^{1}H NMR (400 MHz, $CDCl_3$) (Xantphos)Pd(3-Py)(I) 2

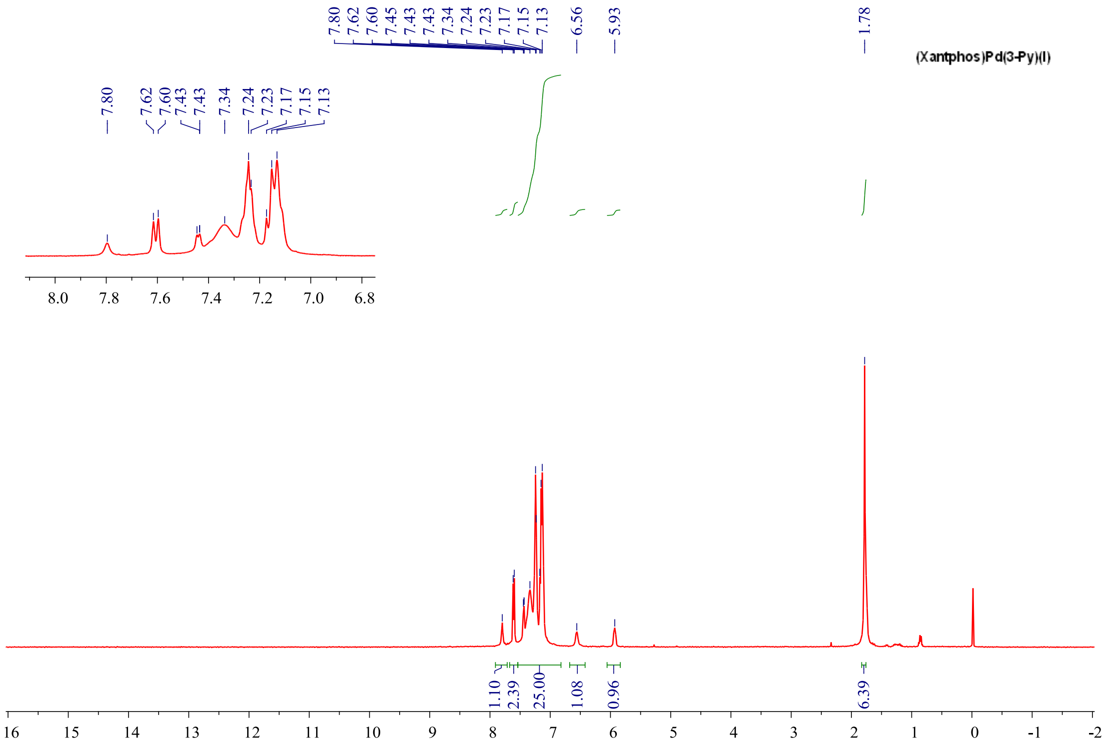

^{31}P NMR (162 MHz, $CDCl_3$) (Xantphos)Pd(3-Py)(I) 2

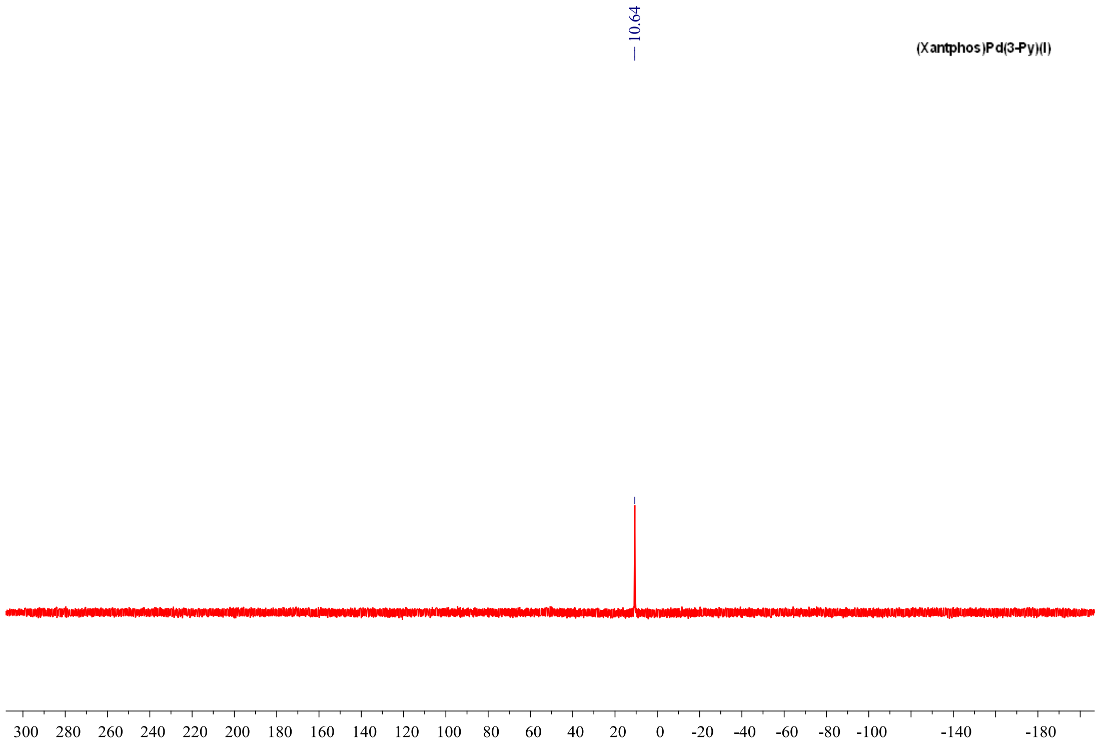

^{1}H NMR (400 MHz, $CDCl_3$) (Xantphos)Pd(3-Py)(Br)

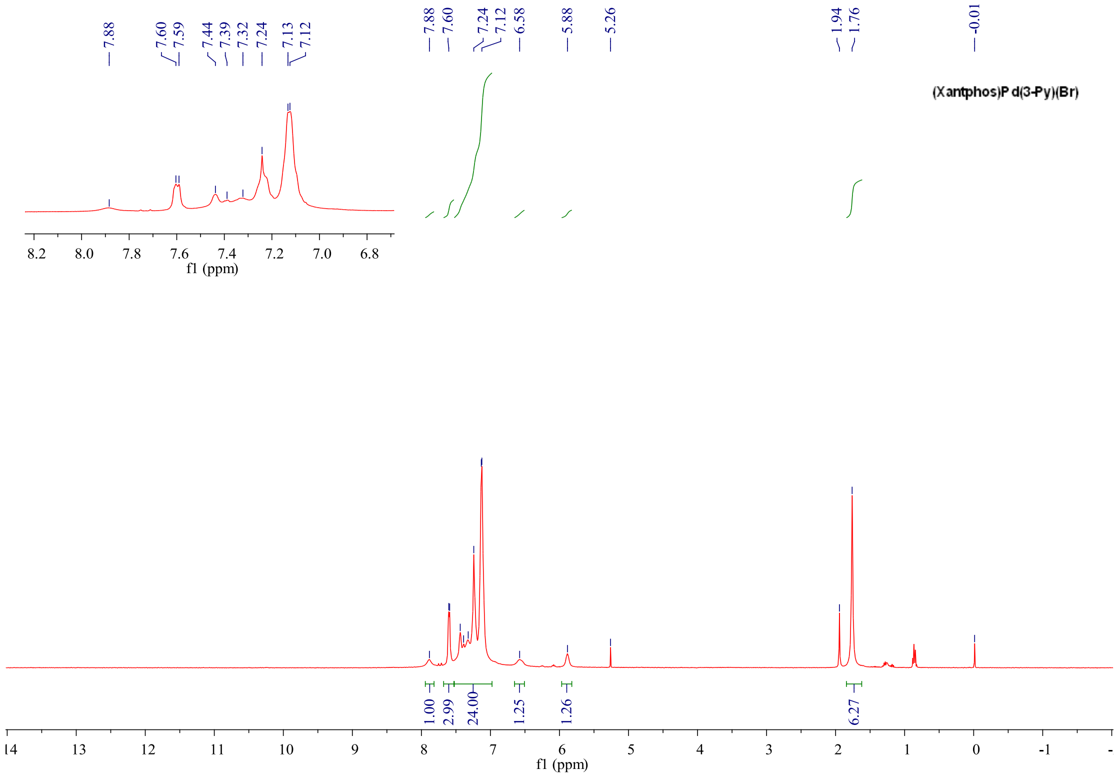

^{31}P NMR (162 MHz, $CDCl_3$) (Xantphos)Pd(3-Py)(Br)

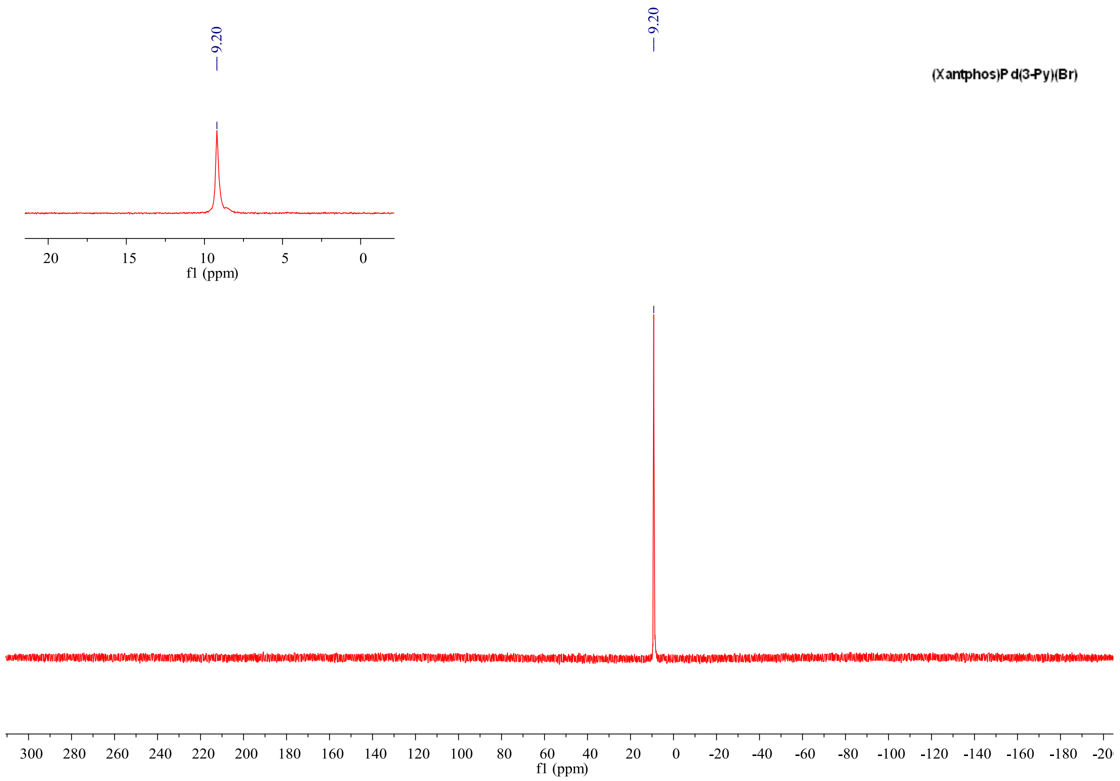

^{1}H NMR (400 MHz, $CDCl_3$) 6-iodobenzo[d]thiazole 3ak

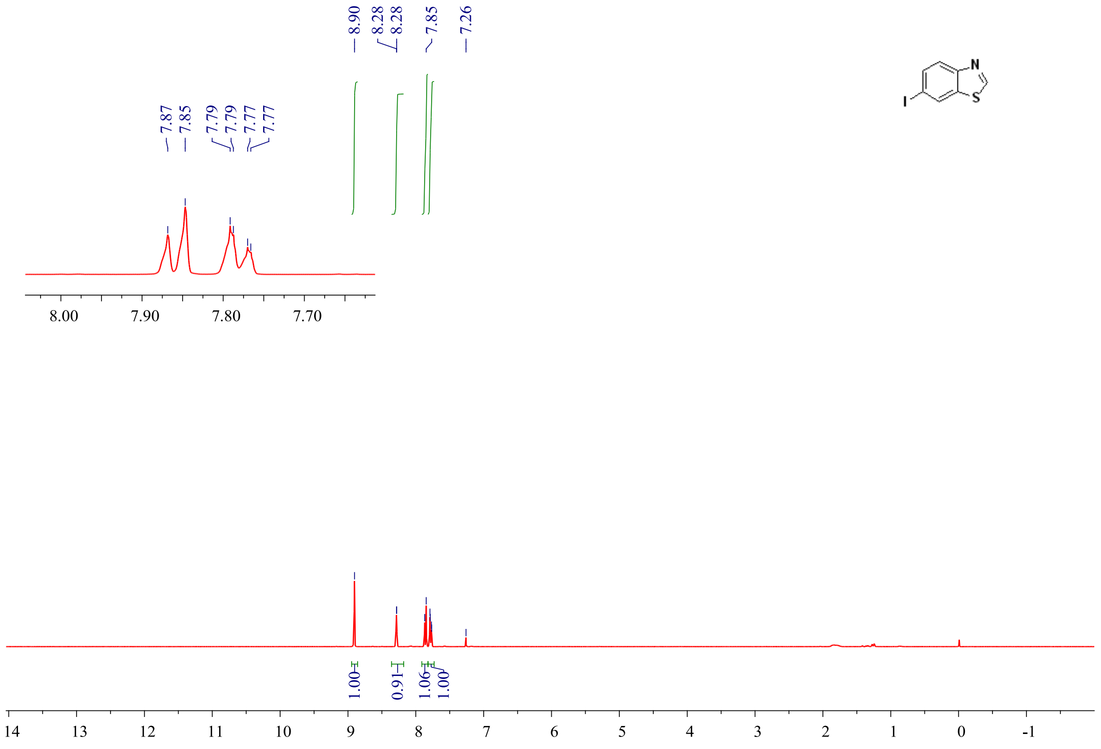

^{13}C NMR (101 MHz, $CDCl_3$) 6-iodobenzo[d]thiazole 3ak

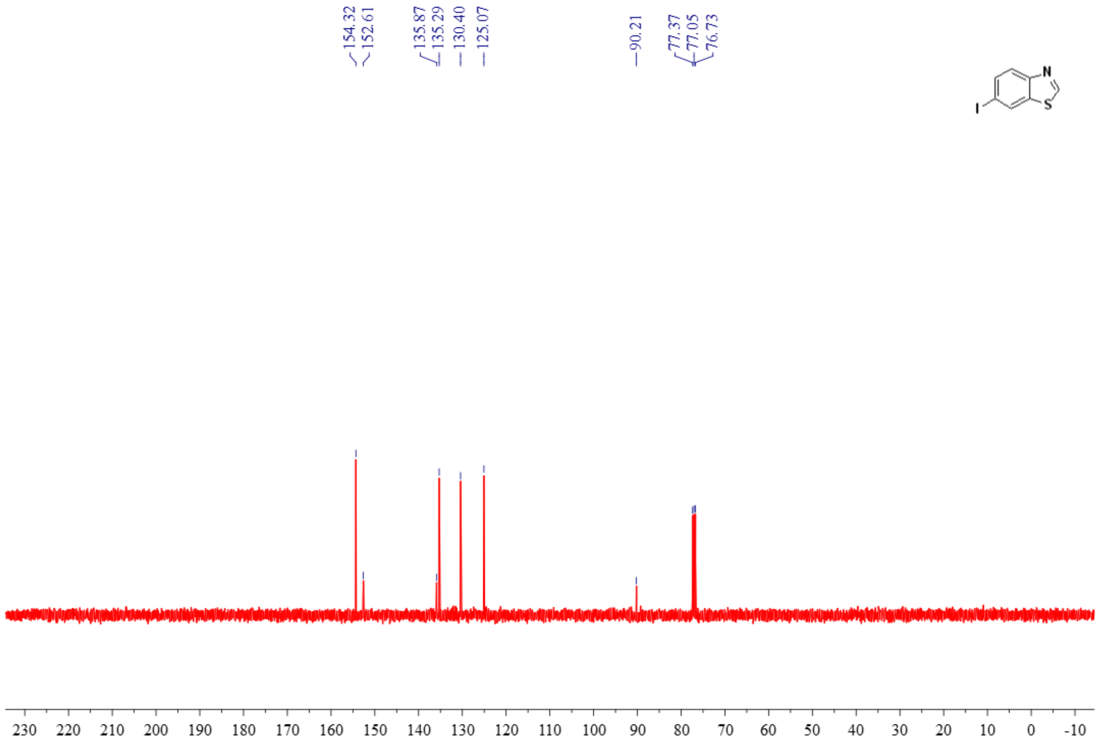

^{1}H NMR (400 MHz, $CDCl_3$) 5-iodobenzofuran 3y

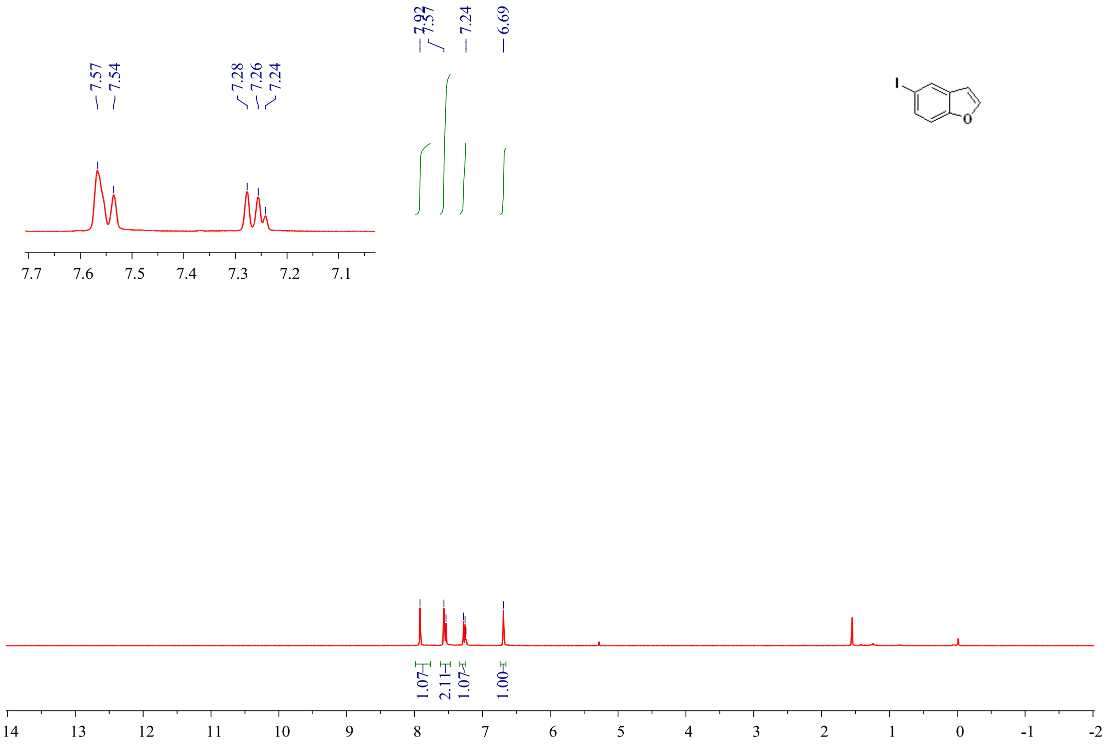

^{13}C NMR (101 MHz, $CDCl_3$) 5-iodobenzofuran 3y

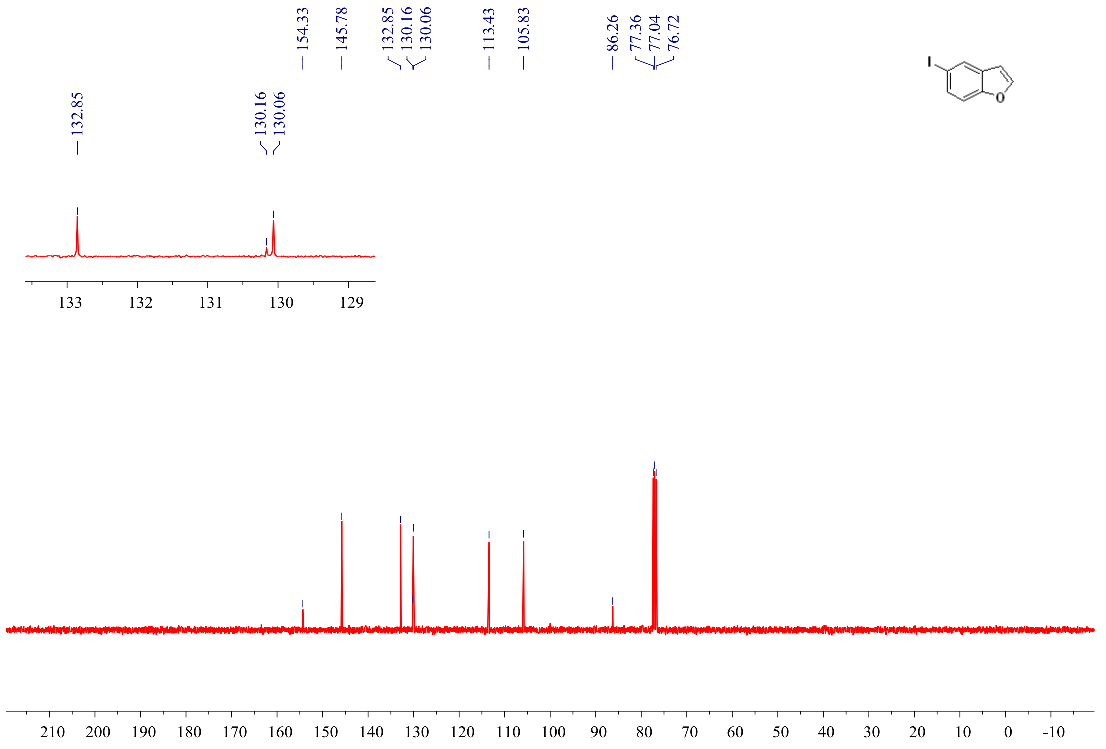

^{1}H NMR (400 MHz, $CDCl_3$) *tert*-butyl 5-iodo-1*H*-indazole-1-carboxylate 3ai

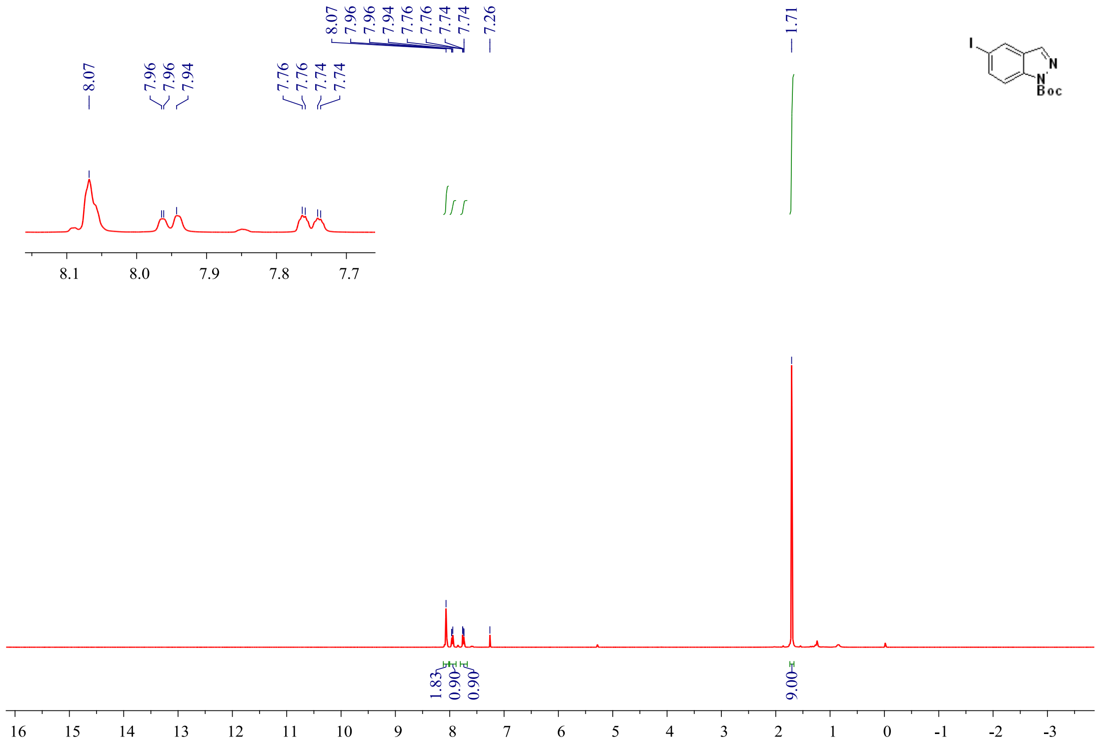

^{13}C NMR (101 MHz, $CDCl_3$) *tert*-butyl 5-iodo-1*H*-indazole-1-carboxylate 3ai

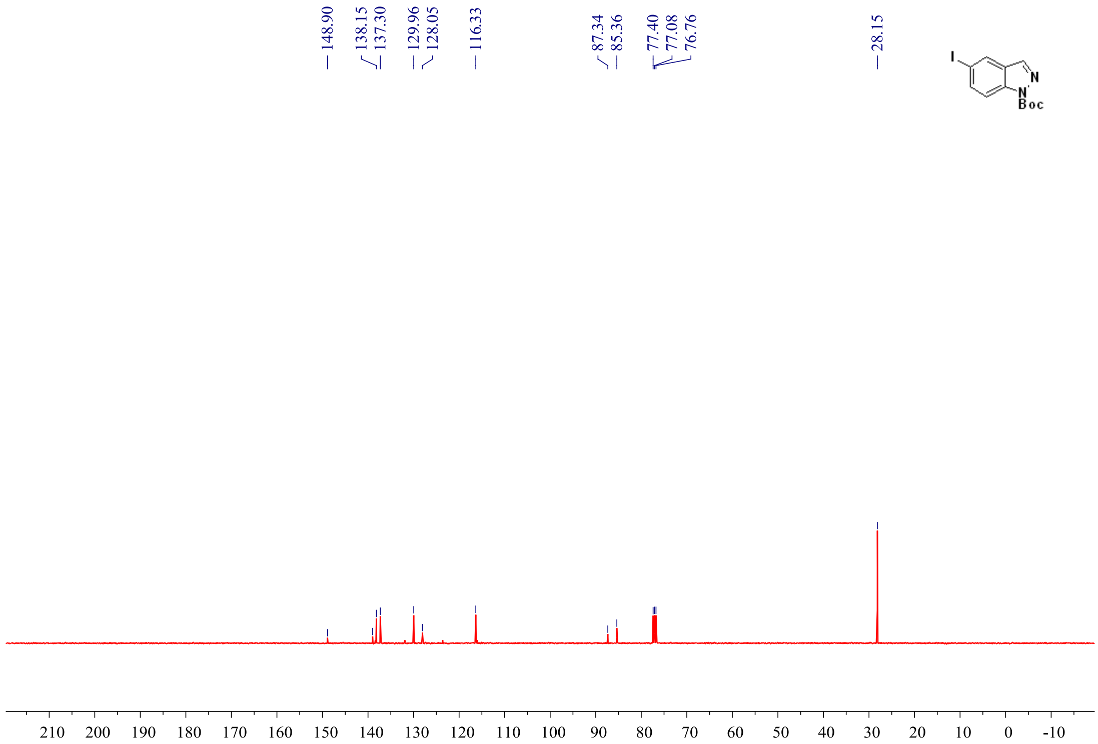

^{1}H NMR (400 MHz, $CDCl_3$) 8-(benzyloxy)-5,7-dichloroquinoline 2u

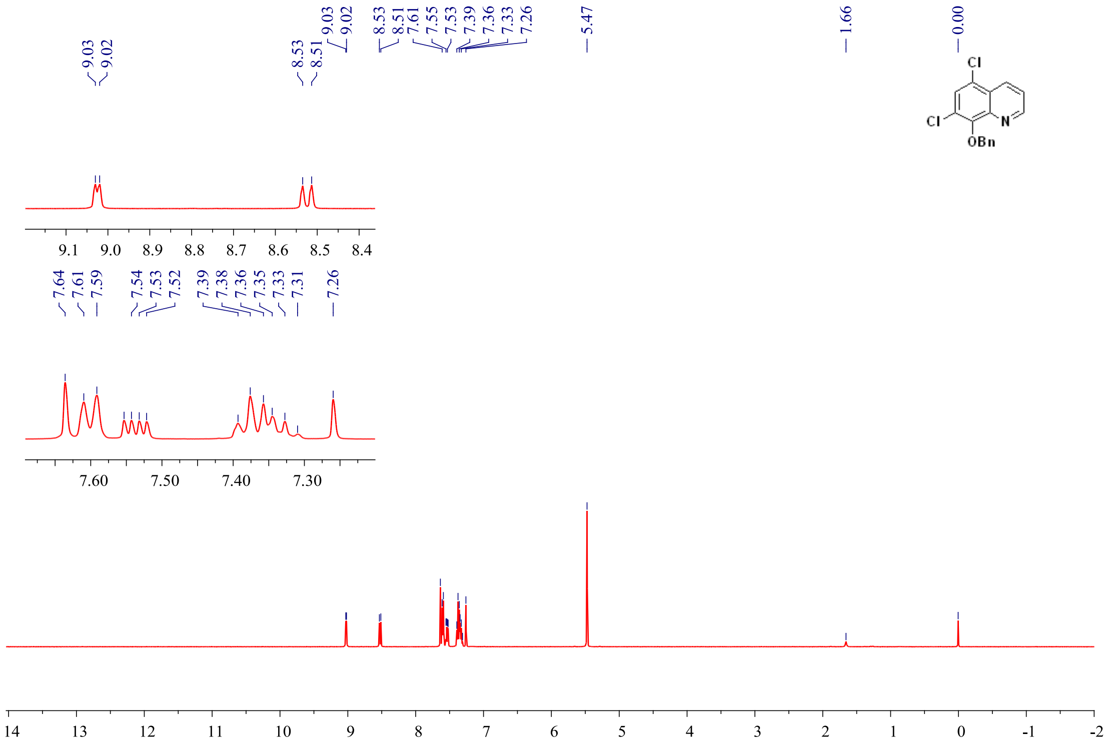

^{13}C NMR (101 MHz, $CDCl_3$) 8-(benzyloxy)-5,7-dichloroquinoline 2u

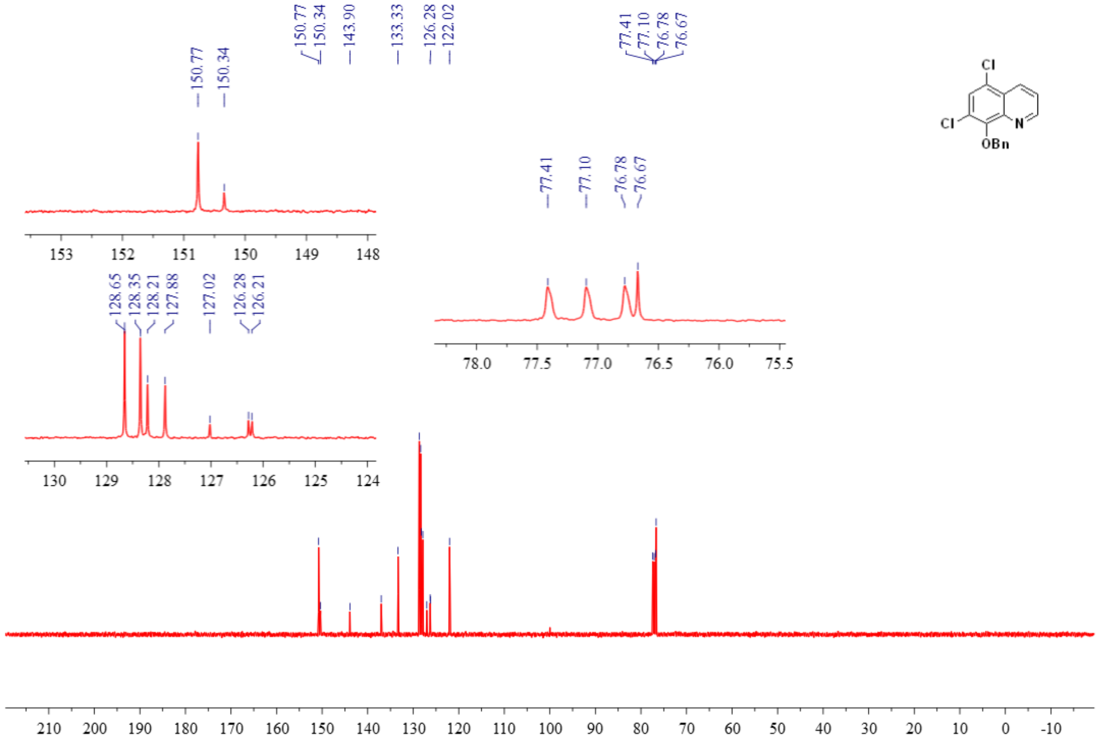

^{1}H NMR (400 MHz, $CDCl_3$) 8-(benzyloxy)-5,7-dichloro-3-iodoquinoline 3u

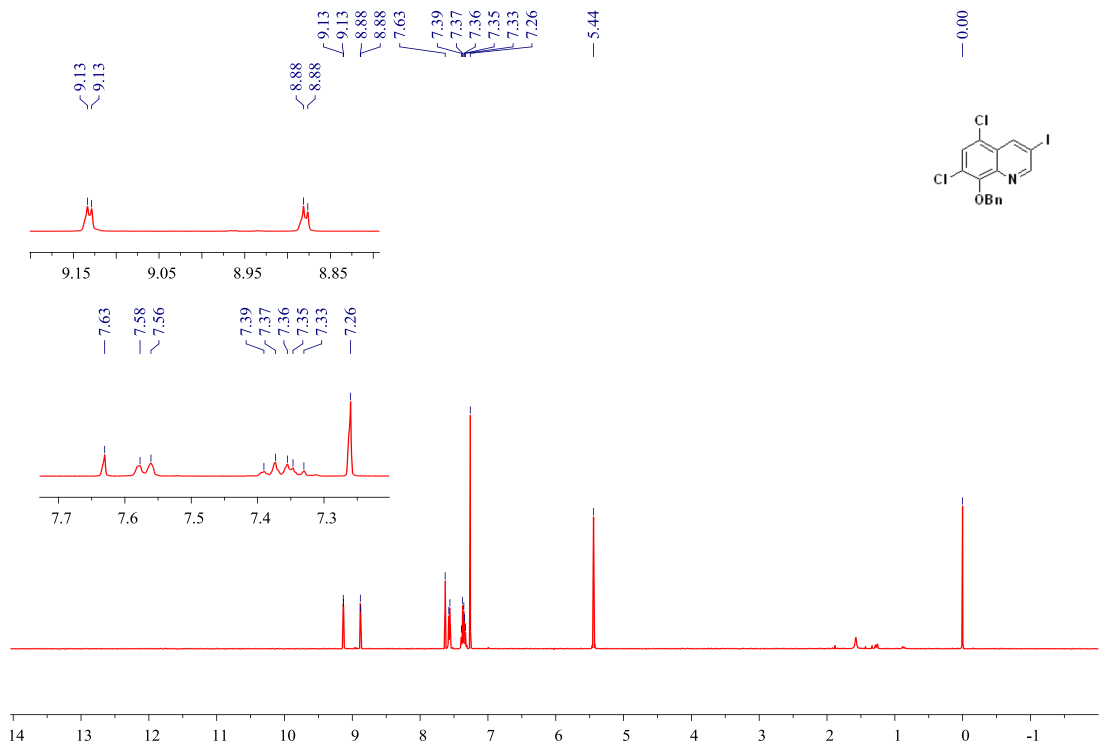

^{13}C NMR (101 MHz, $CDCl_3$) 8-(benzyloxy)-5,7-dichloro-3-iodoquinoline 3u

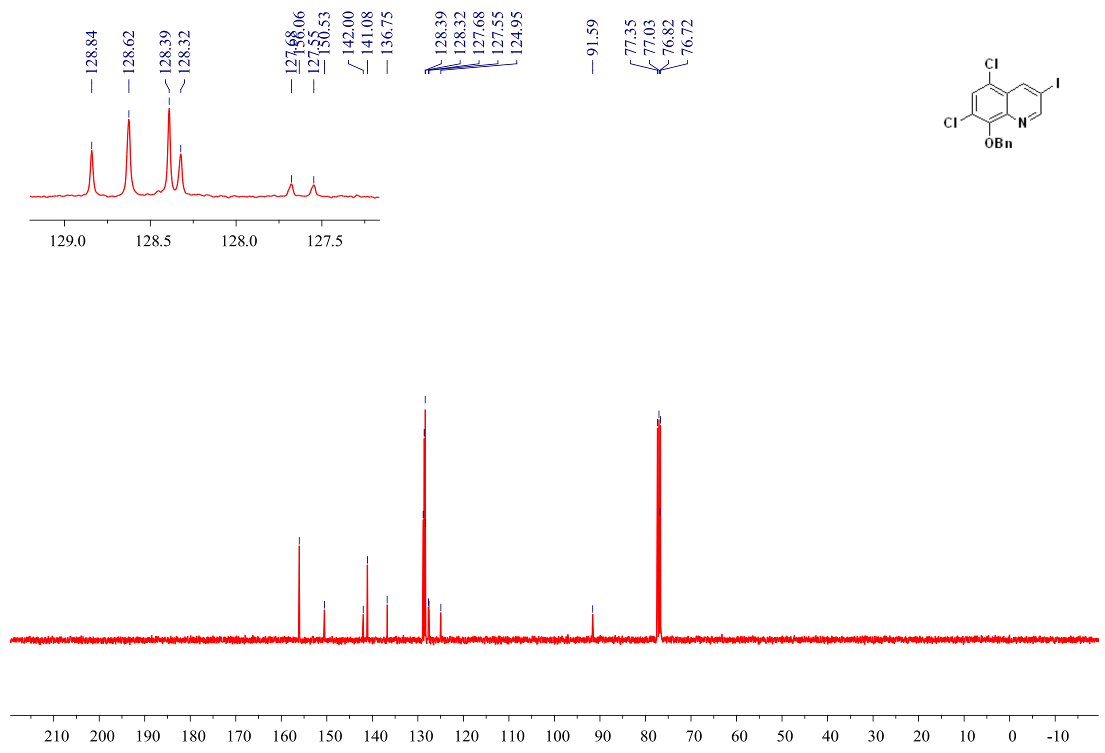

^{1}H NMR (400 MHz, $CDCl_3$) 2-((5-chloro-3-iodoquinolin-8-yl)oxy)acetate 3ay

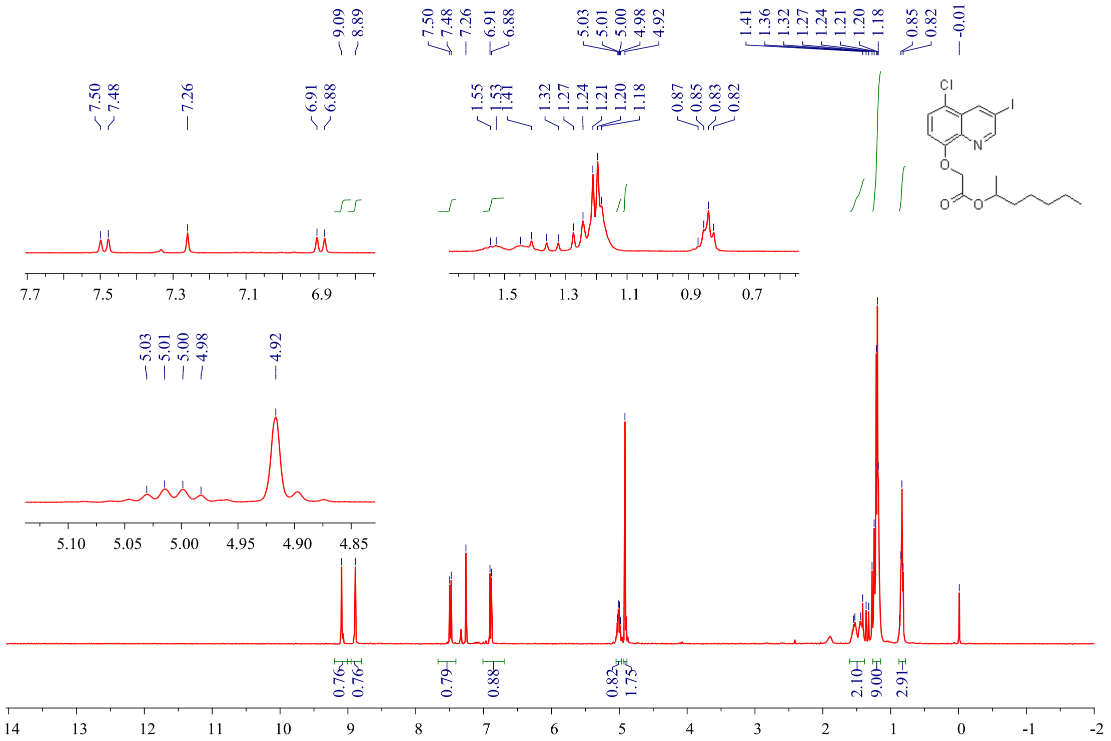

^{13}C NMR (101 MHz, $CDCl_3$) 2-((5-chloro-3-iodoquinolin-8-yl)oxy)acetate 3ay

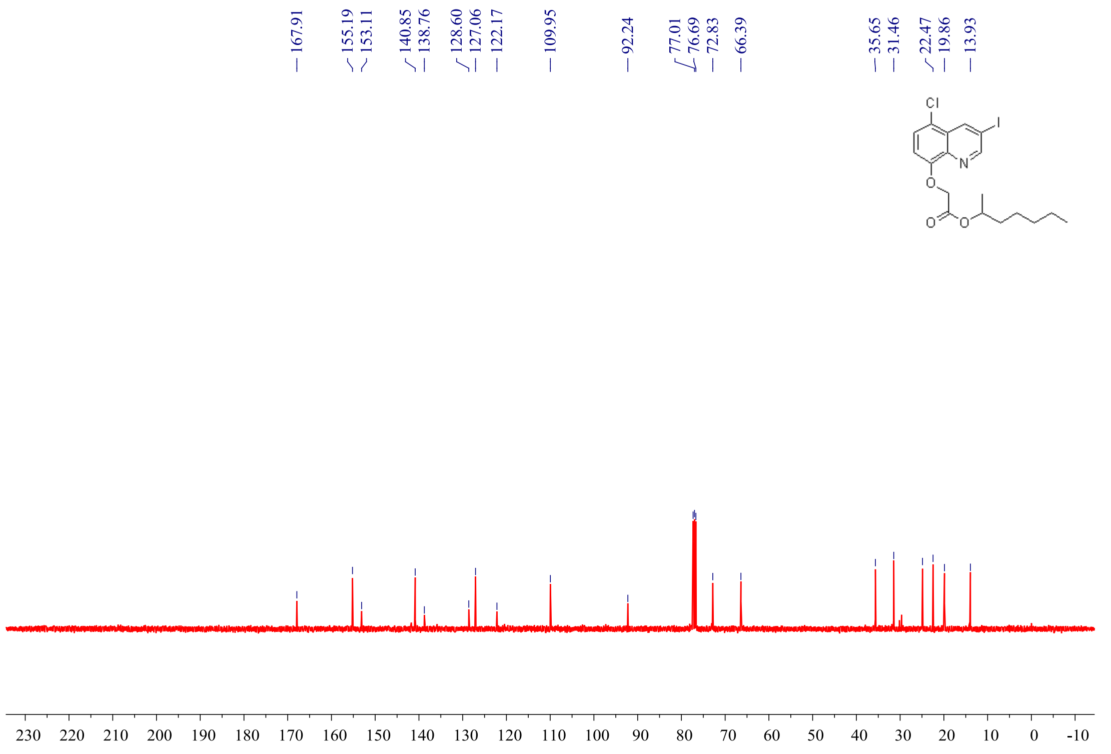

^{1}H NMR (400 MHz, $CDCl_3$) 4-bromo-1-isobutyl-1*H*-imidazo[4,5-c]quinolone 3ax

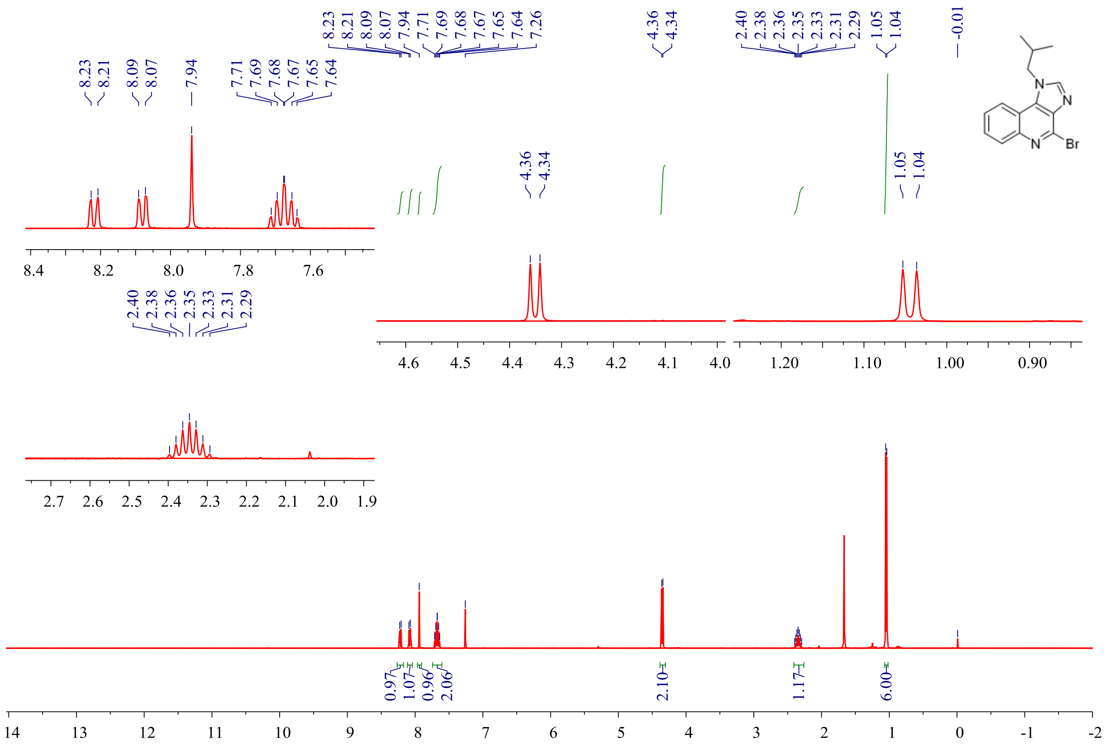

^{13}C NMR (126 MHz, $CDCl_3$) 4-bromo-1-*iso*butyl-1*H*-imidazo[4,5-c]quinolone 3ax

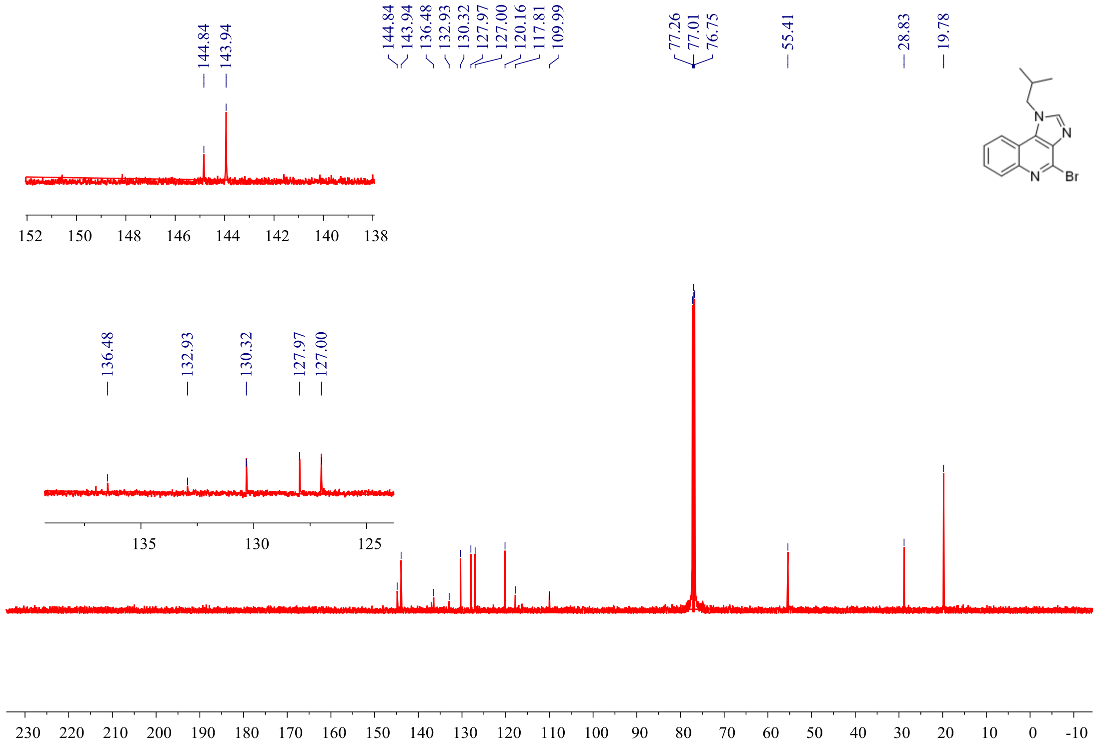

^{1}H NMR (400 MHz, $CDCl_3$) 5-bromopyridin-2-yl trifluoromethanesulfonate 3a

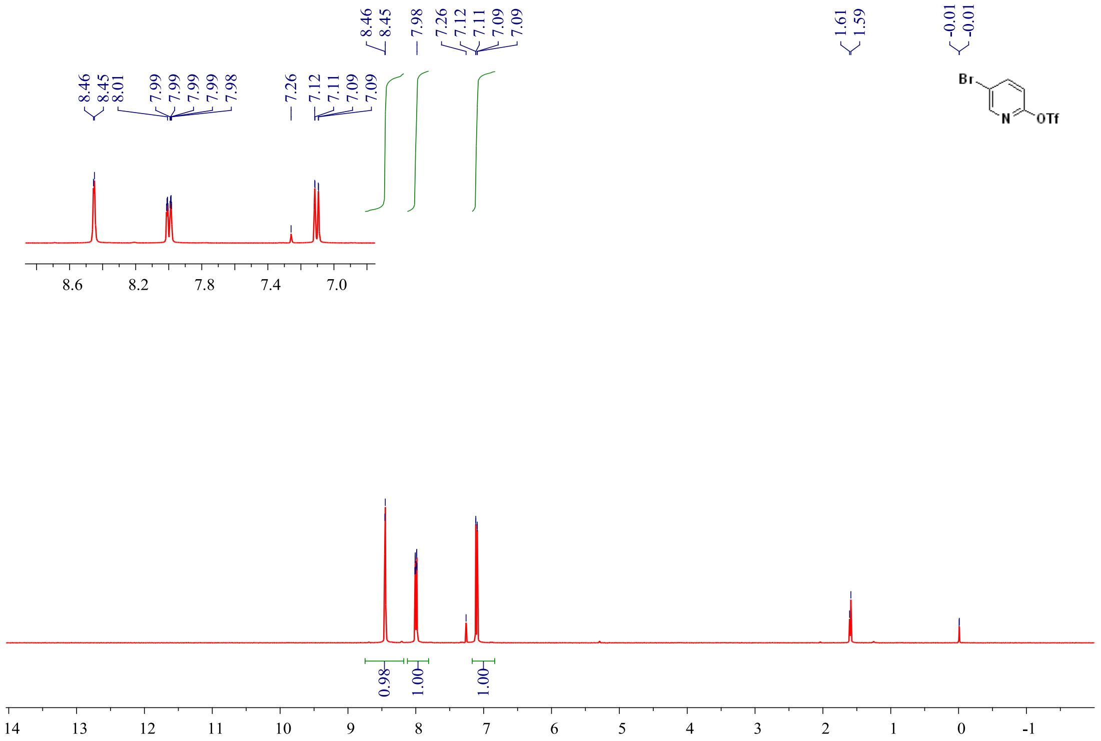

^{19}F NMR (376 MHz, $CDCl_3$) 5-bromopyridin-2-yl trifluoromethanesulfonate 3a

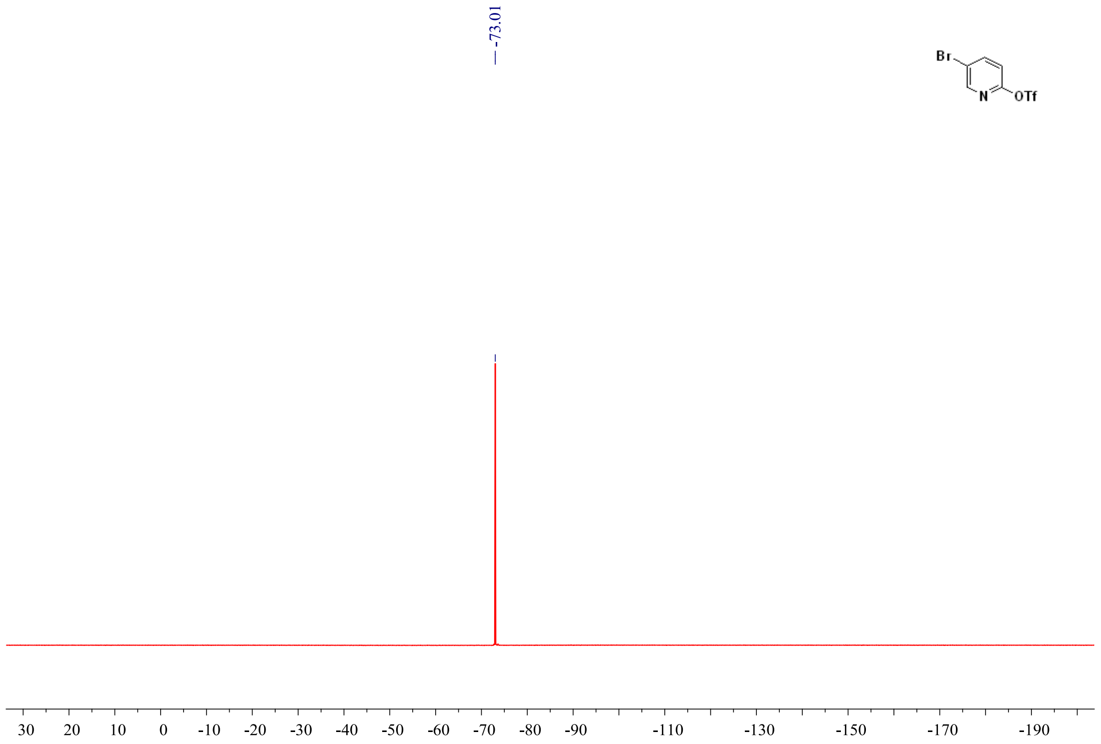

^{13}C NMR (101 MHz, CDCl$_3$) 5-bromopyridin-2-yl trifluoromethanesulfonate 3a

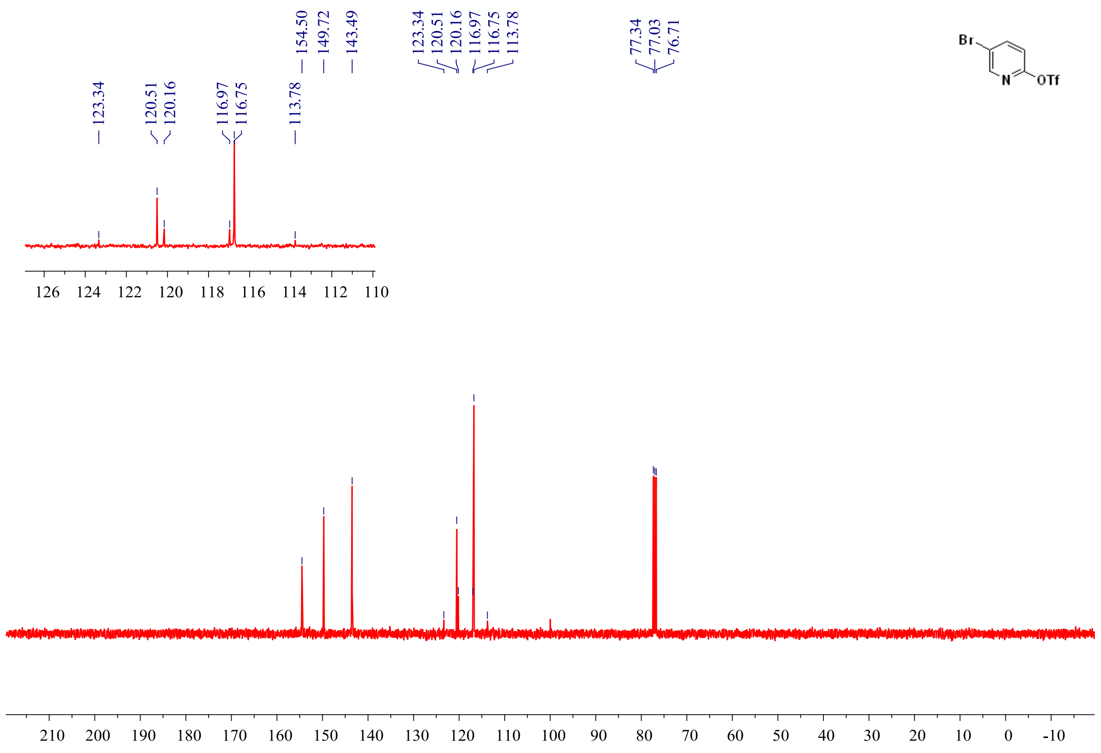

^{1}H NMR (400 MHz, CDCl$_3$) 5-chloropyridin-2-yl trifluoromethanesulfonate 3b

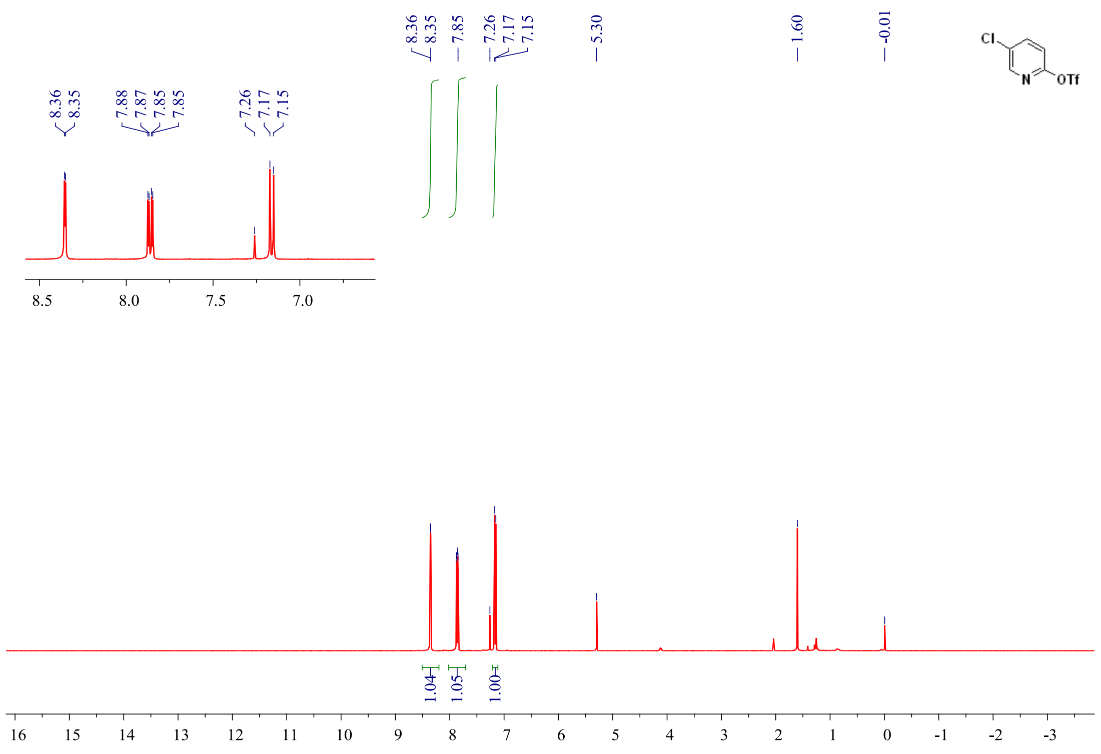

^{19}F NMR (1376 MHz, $CDCl_3$) 5-chloropyridin-2-yl trifluoromethanesulfonate 3b

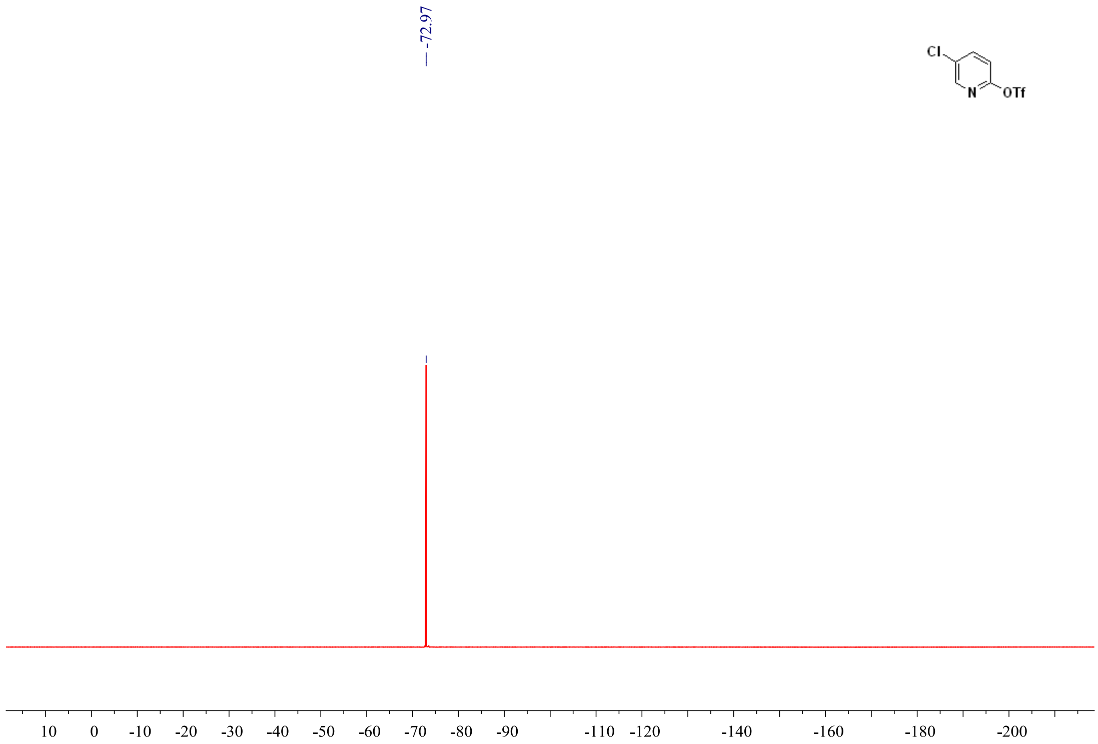

^{13}C NMR (101 MHz, $CDCl_3$) 5-chloropyridin-2-yl trifluoromethanesulfonate 3b

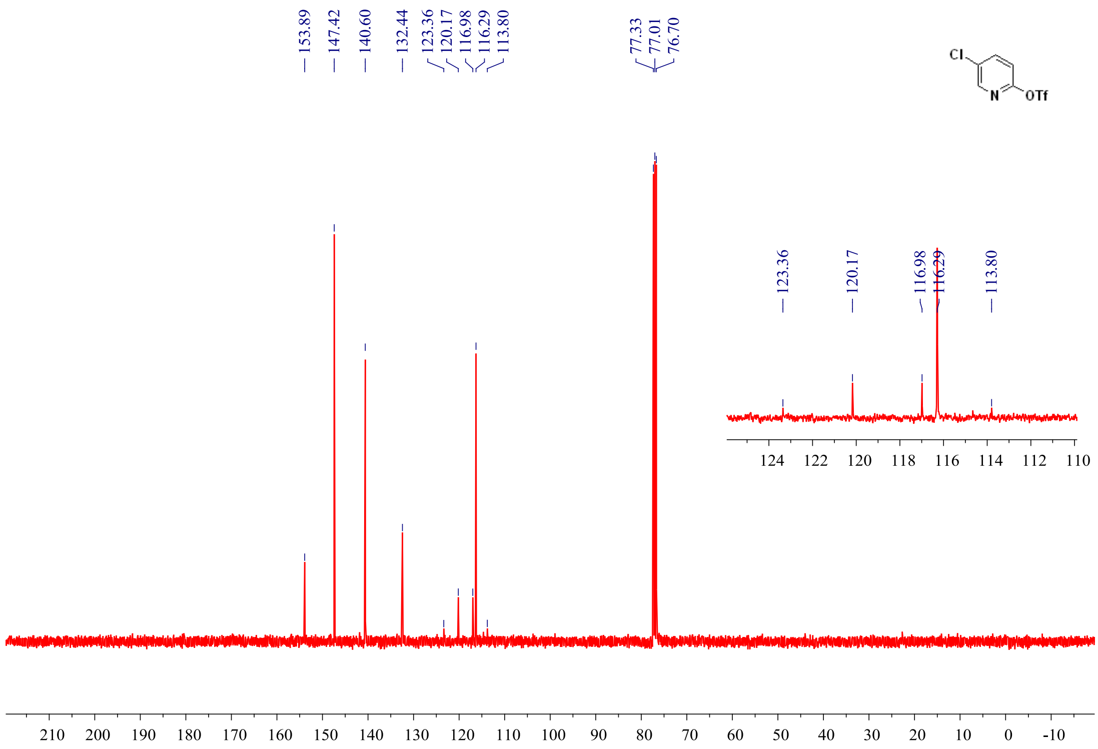

^{1}H NMR (400 MHz, $CDCl_3$) isoquinolin-1-yl trifluoromethanesulfonate 3v

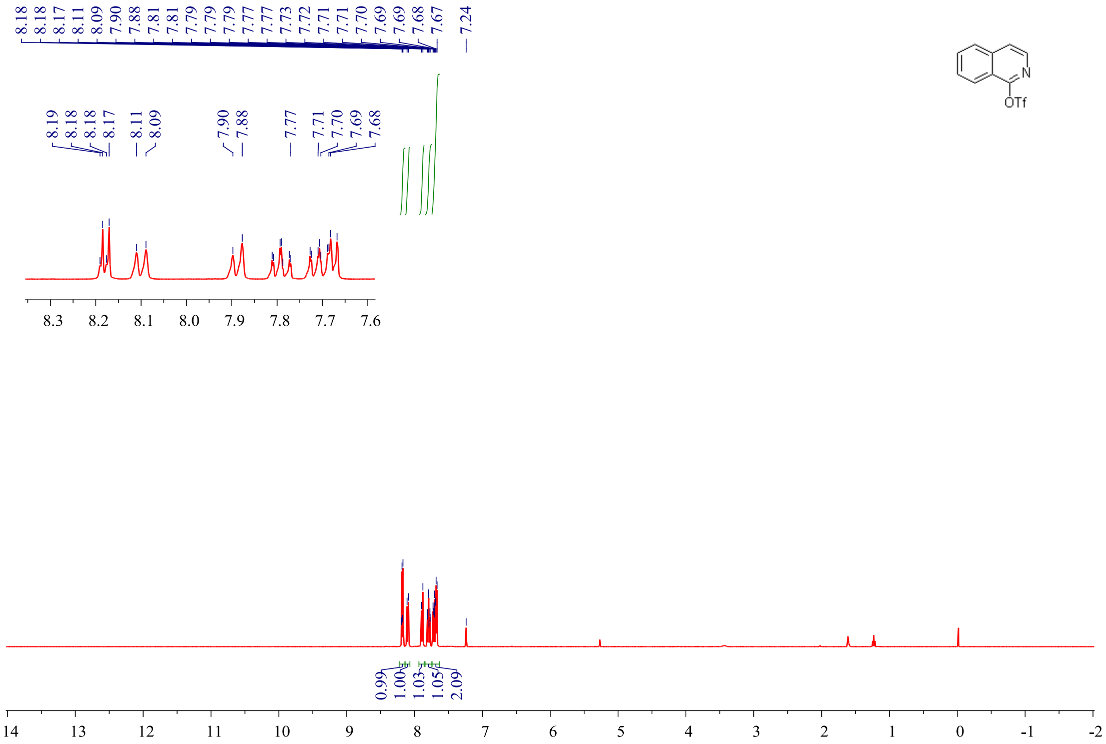

^{19}F NMR (376 MHz, $CDCl_3$) isoquinolin-1-yl trifluoromethanesulfonate 3v

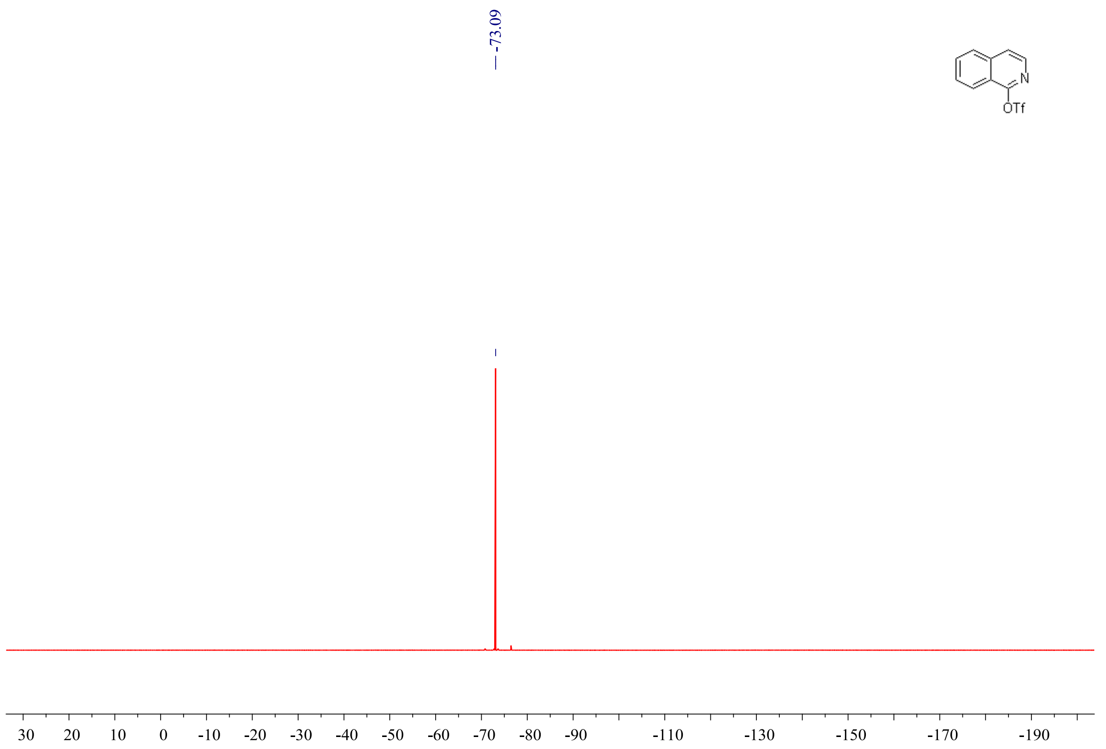

^{13}C NMR (101 MHz, $CDCl_3$) isoquinolin-1-yl trifluoromethanesulfonate 3v

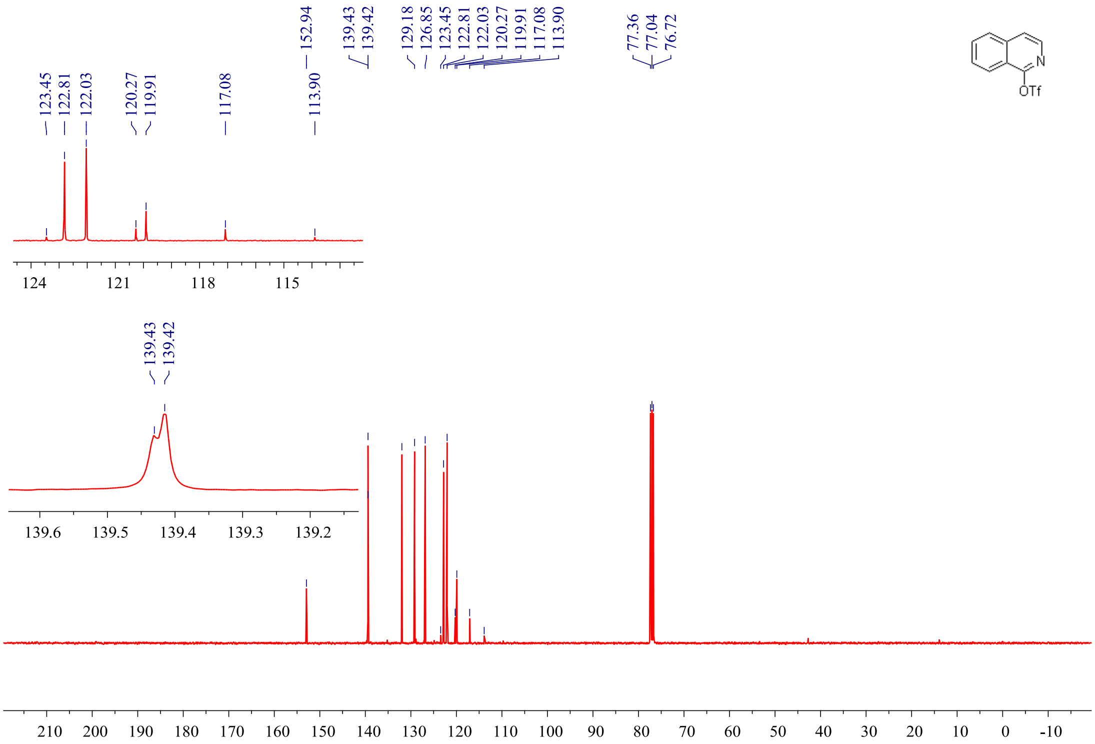

^{1}H NMR (400 MHz, $CDCl_3$) 5-bromopyrimidin-2-yl trifluoromethanesulfonate 3ac

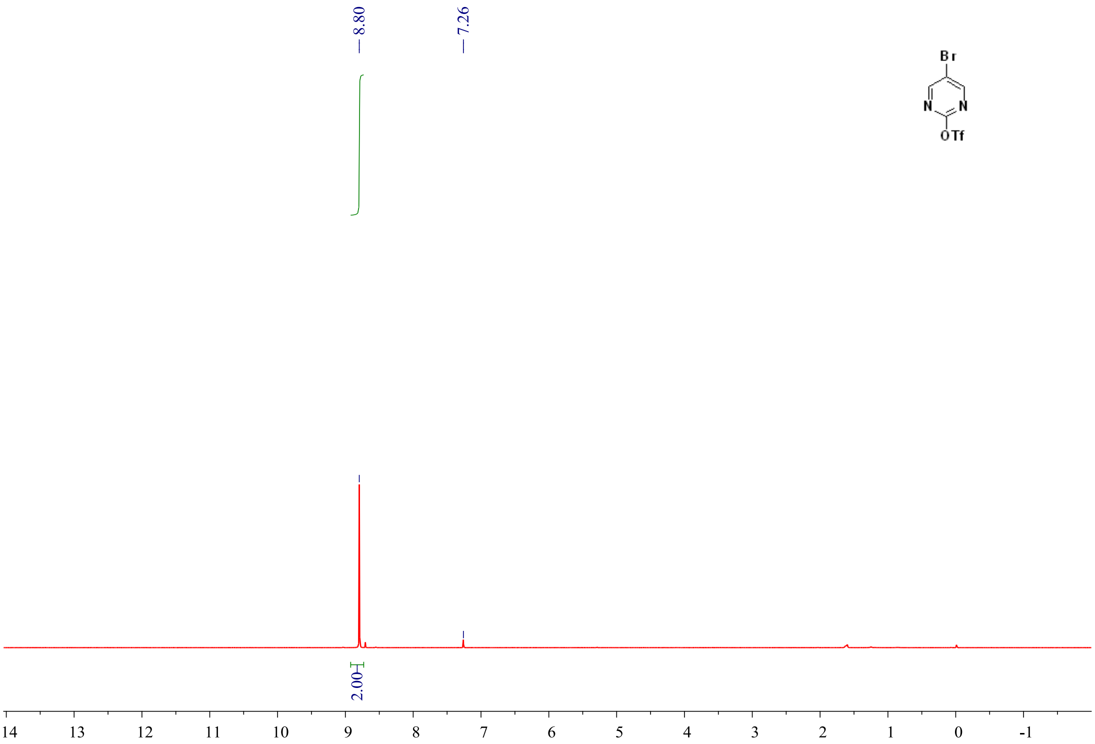

^{19}F NMR (376 MHz, $CDCl_3$) 5-bromopyrimidin-2-yl trifluoromethanesulfonate 3ac

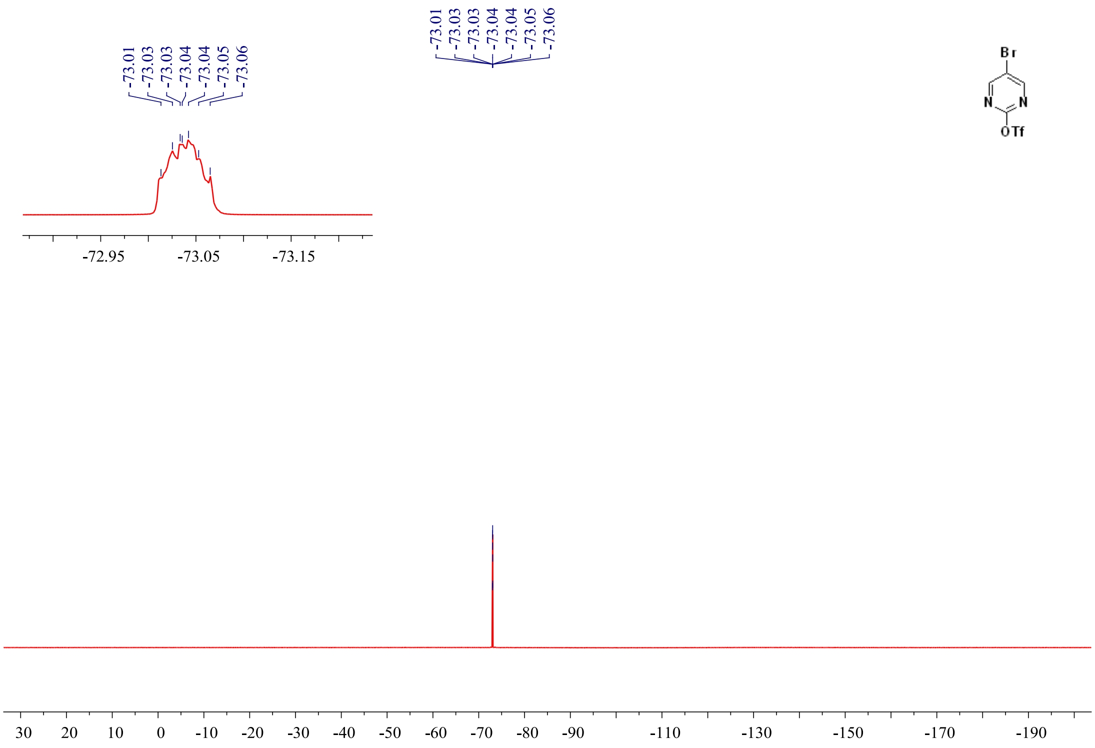

^{13}C NMR (101 MHz, $CDCl_3$) 5-bromopyrimidin-2-yl trifluoromethanesulfonate 3ac

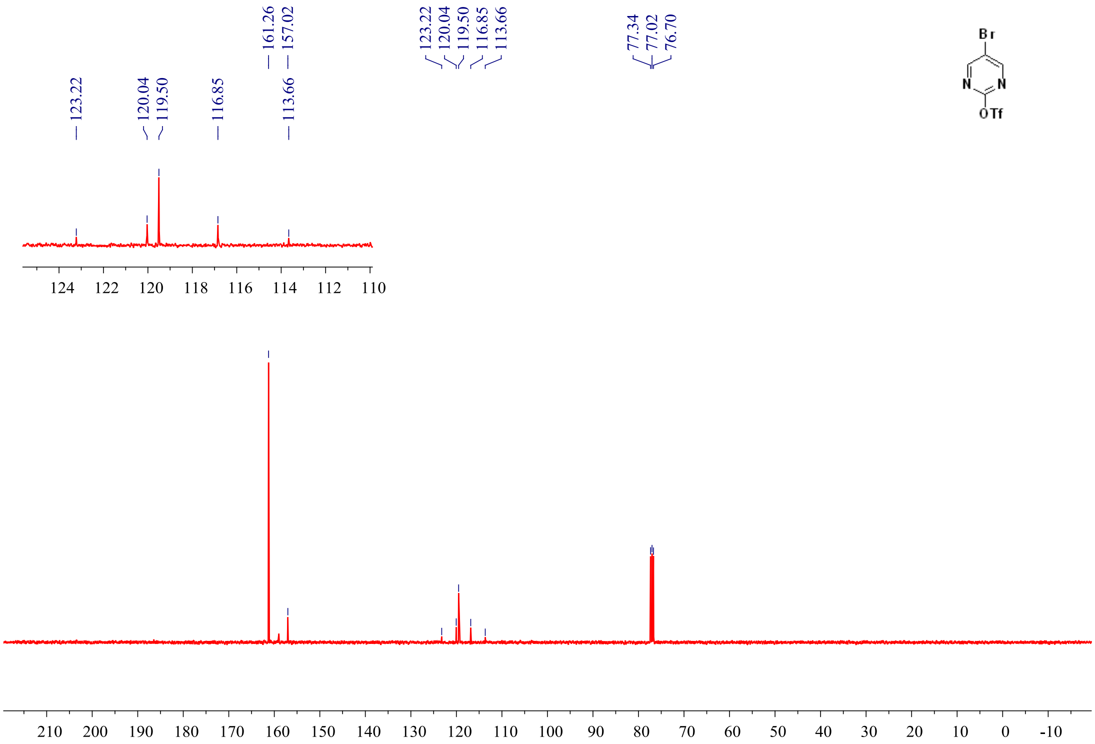

^{1}H NMR (400 MHz, $CDCl_3$) quinolin-2-yl trifluoromethanesulfonate 3ap

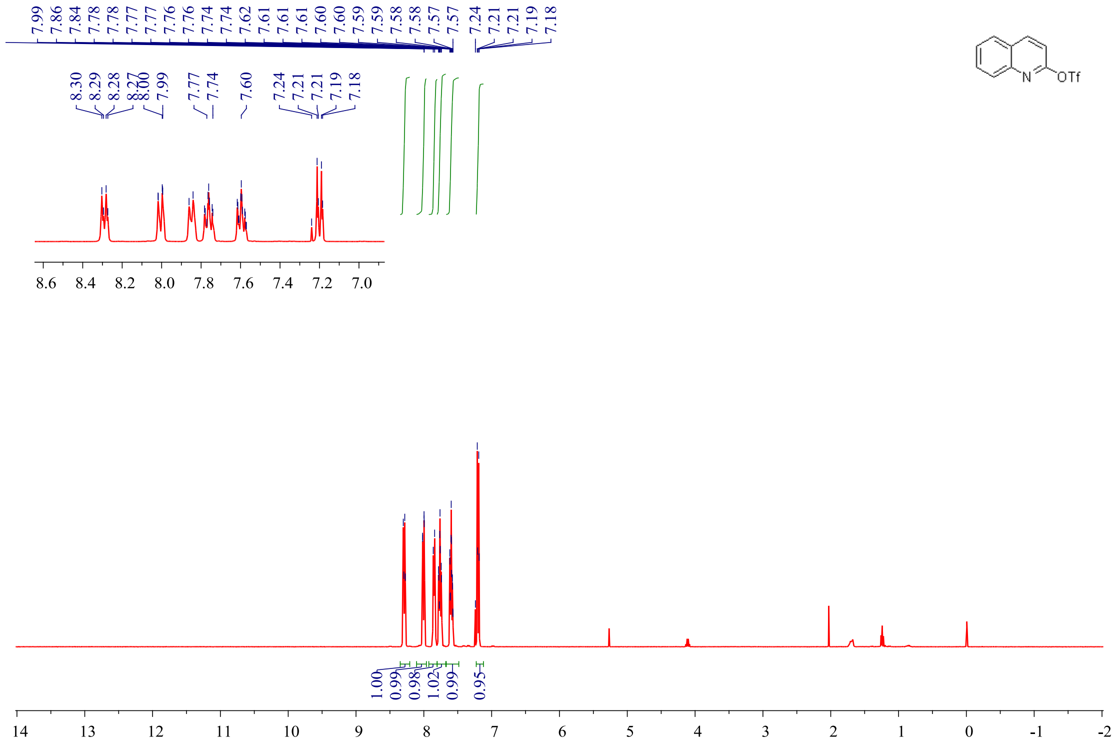

^{19}F NMR (376 MHz, $CDCl_3$) quinolin-2-yl trifluoromethanesulfonate 3ap

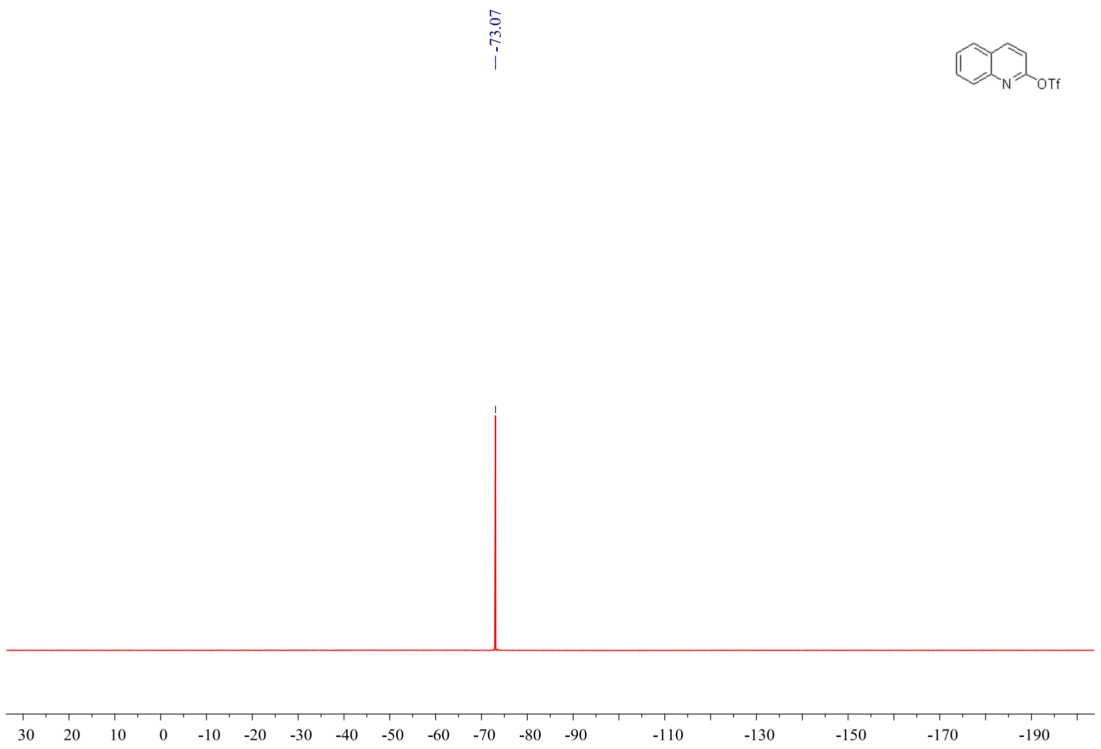

^{13}C NMR (101 MHz, $CDCl_3$) quinolin-2-yl trifluoromethanesulfonate 3ap

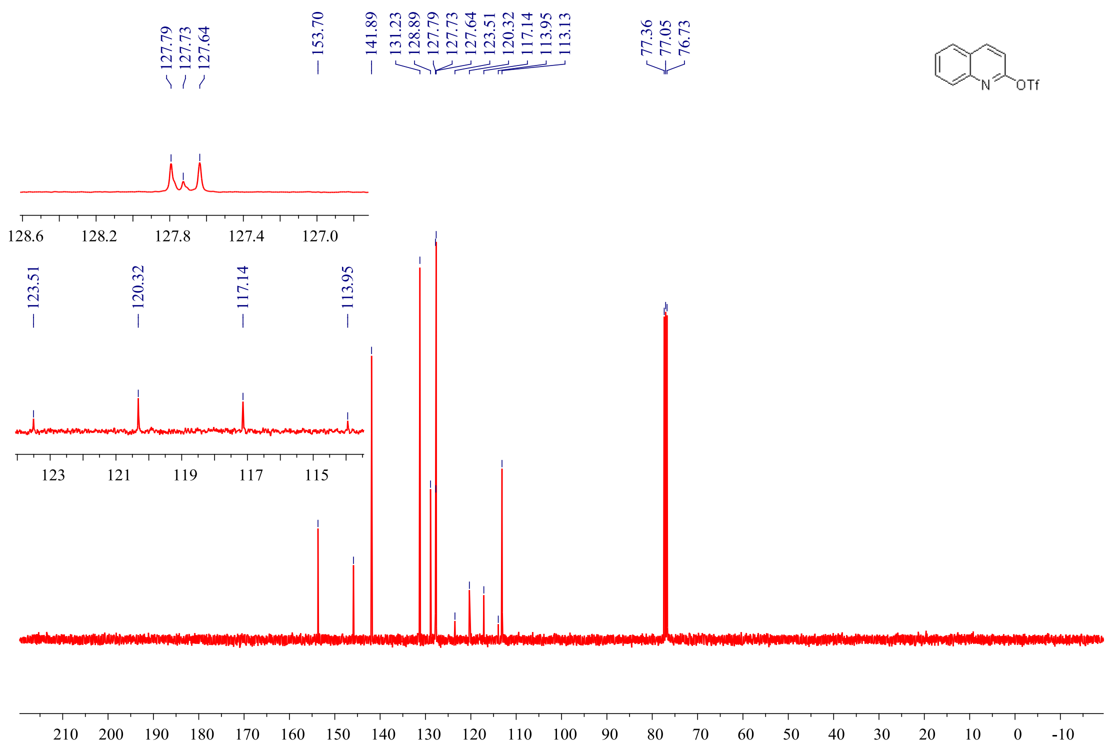

^{1}H NMR (400 MHz, $CDCl_3$) quinoxalin-2-yl trifluoromethanesulfonate 3at

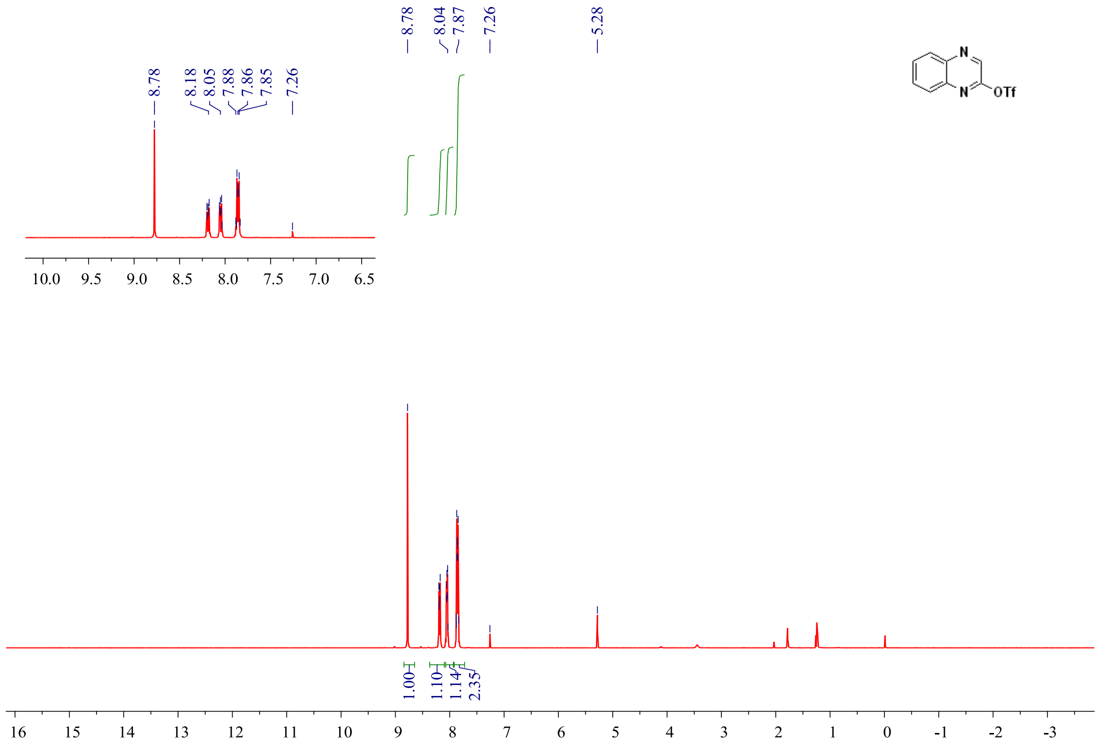

^{19}F NMR (376 MHz, $CDCl_3$) quinoxalin-2-yl trifluoromethanesulfonate 3at

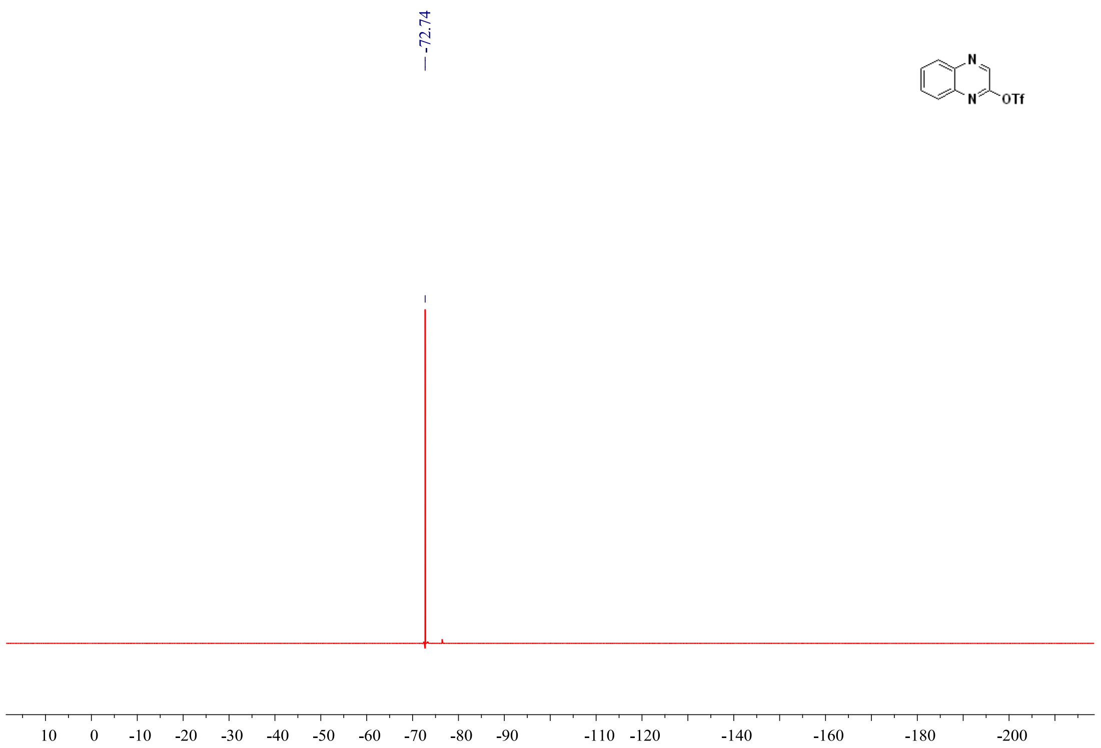

^{13}C NMR (101 MHz, $CDCl_3$) quinoxalin-2-yl trifluoromethanesulfonate 3at

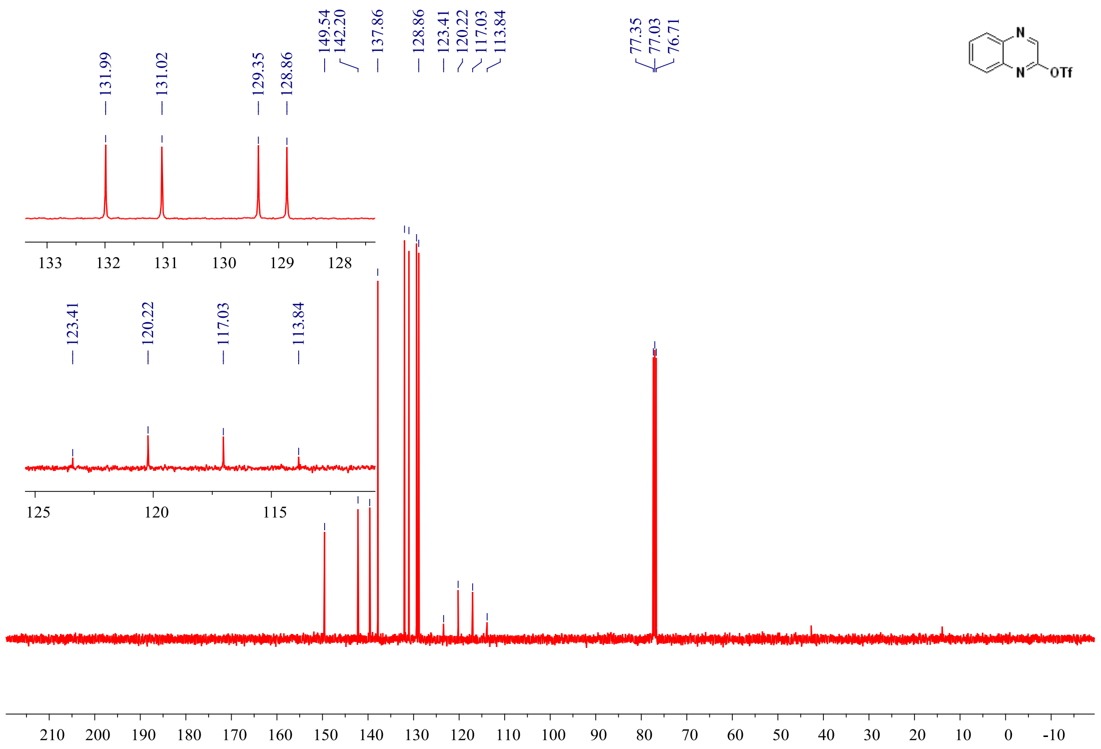

^{1}H NMR (400 MHz, $CDCl_3$) 7-bromoisoquinolin-1-yl trifluoromethanesulfonate

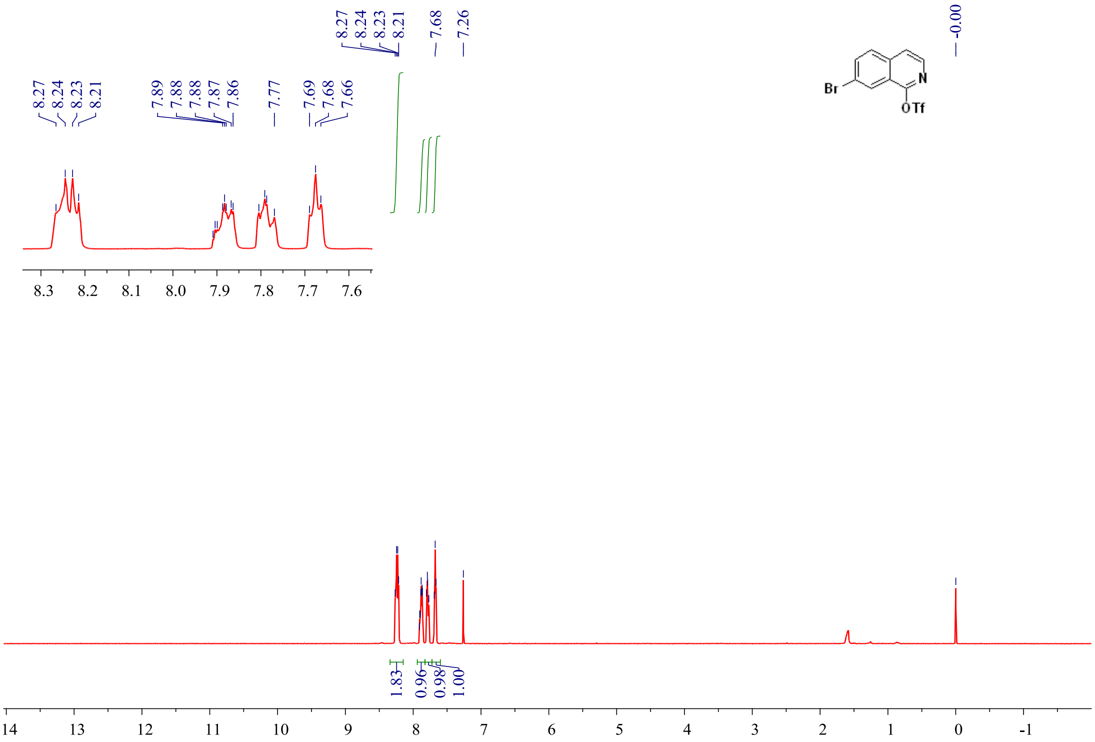

^{1}H NMR (400 MHz, $CDCl_3$) 5-bromo-3-nitropyridin-2-yl trifluoromethanesulfonate 3av

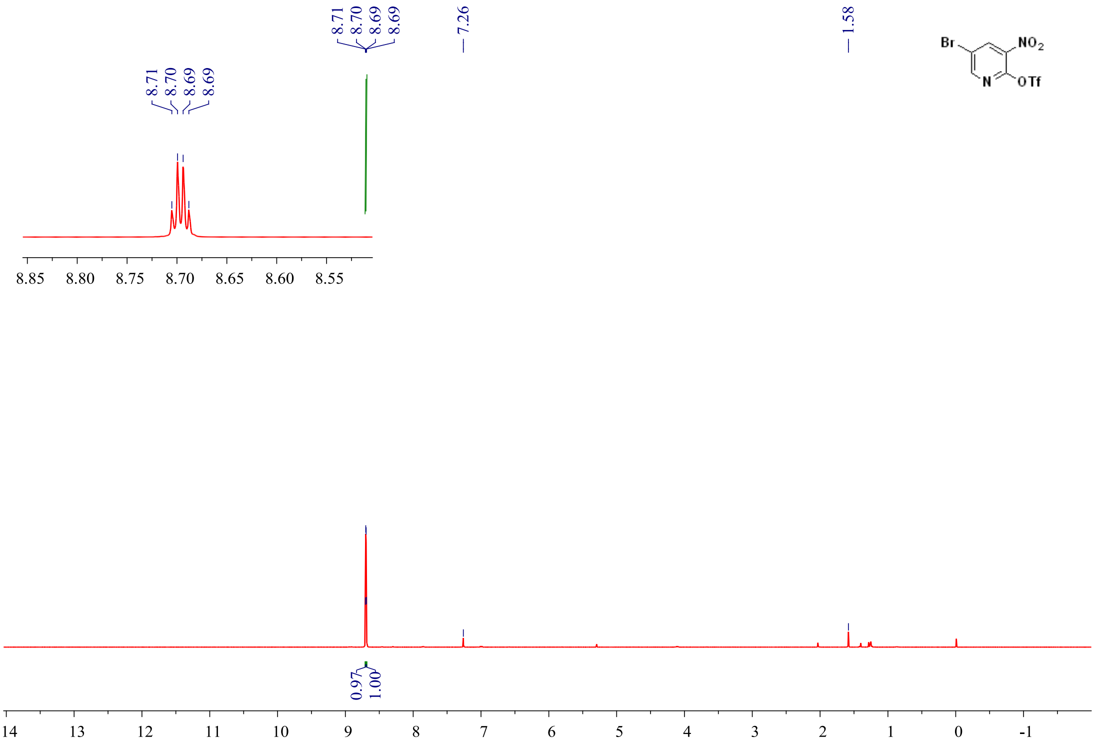

^{19}F NMR (376 MHz, $CDCl_3$) 5-bromo-3-nitropyridin-2-yl trifluoromethanesulfonate 3av

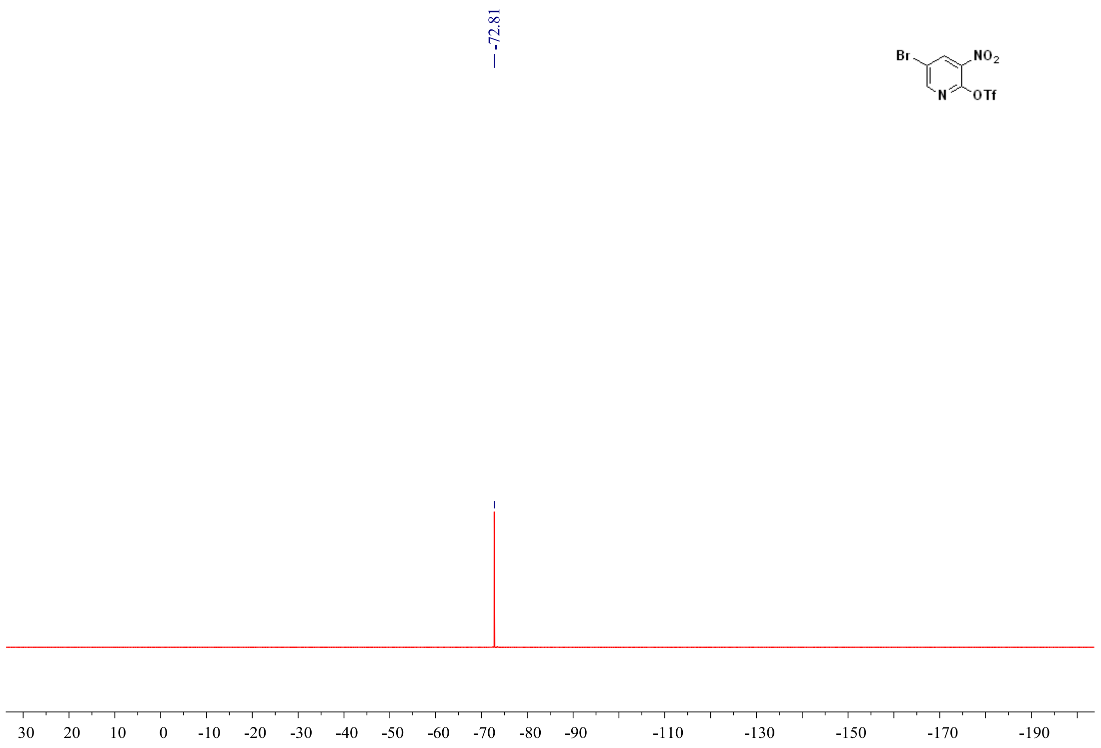

^{13}C NMR (101 MHz, $CDCl_3$) 5-bromo-3-nitropyridin-2-yl trifluoromethanesulfonate 3av

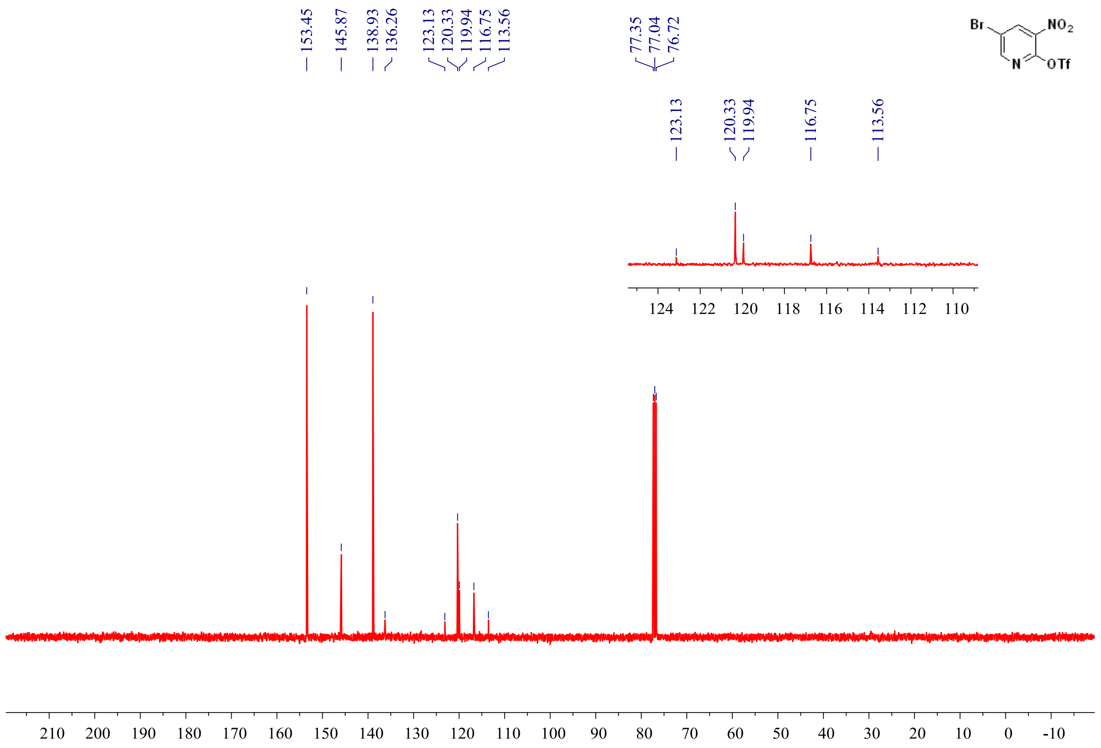

^{19}F NMR (376 MHz, $CDCl_3$) 7-bromoisoquinolin-1-yl trifluoromethanesulfonate 3aw

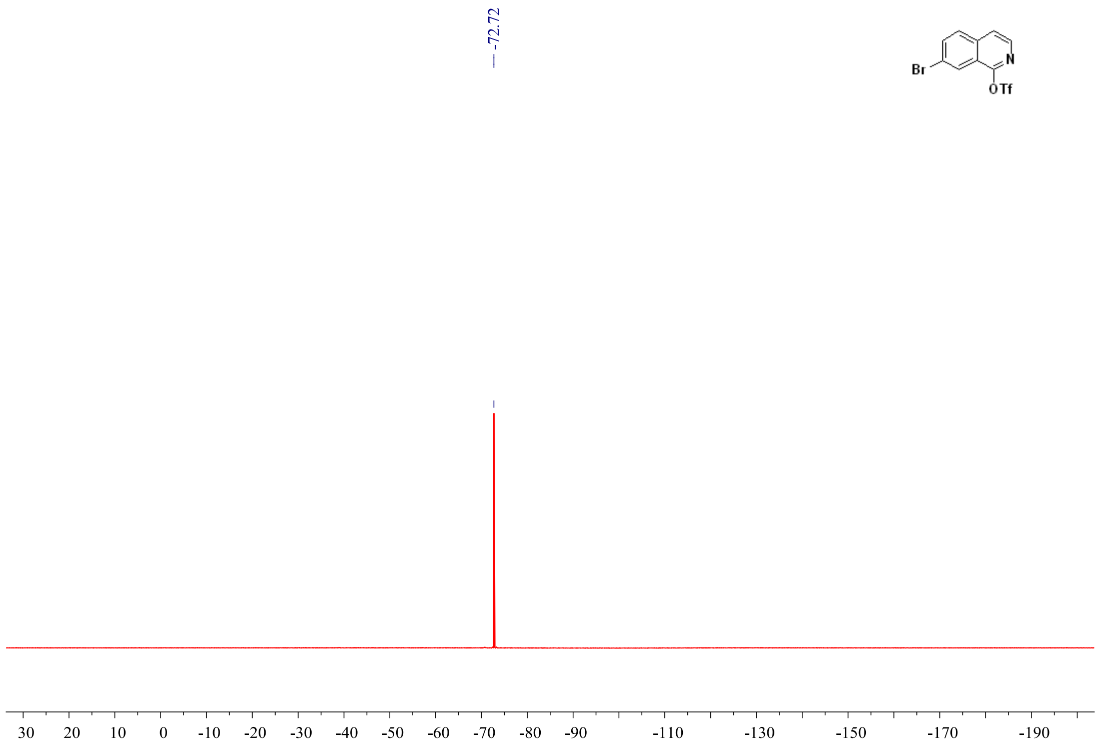

^{13}C NMR (101 MHz, $CDCl_3$) 7-bromoisoquinolin-1-yl trifluoromethanesulfonate 3aw

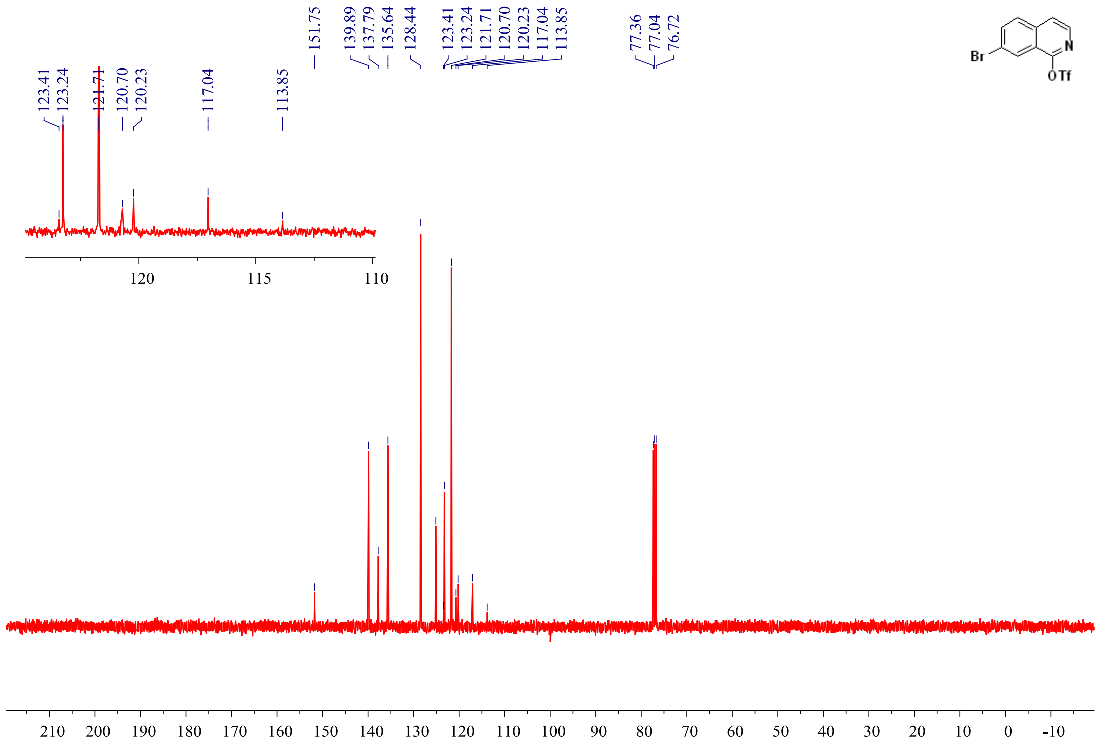

Spectrum of the Products

^{1}H NMR (400 MHz, $CDCl_3$) 5-bromo-2-((difluoromethyl)thio)pyridine 4a

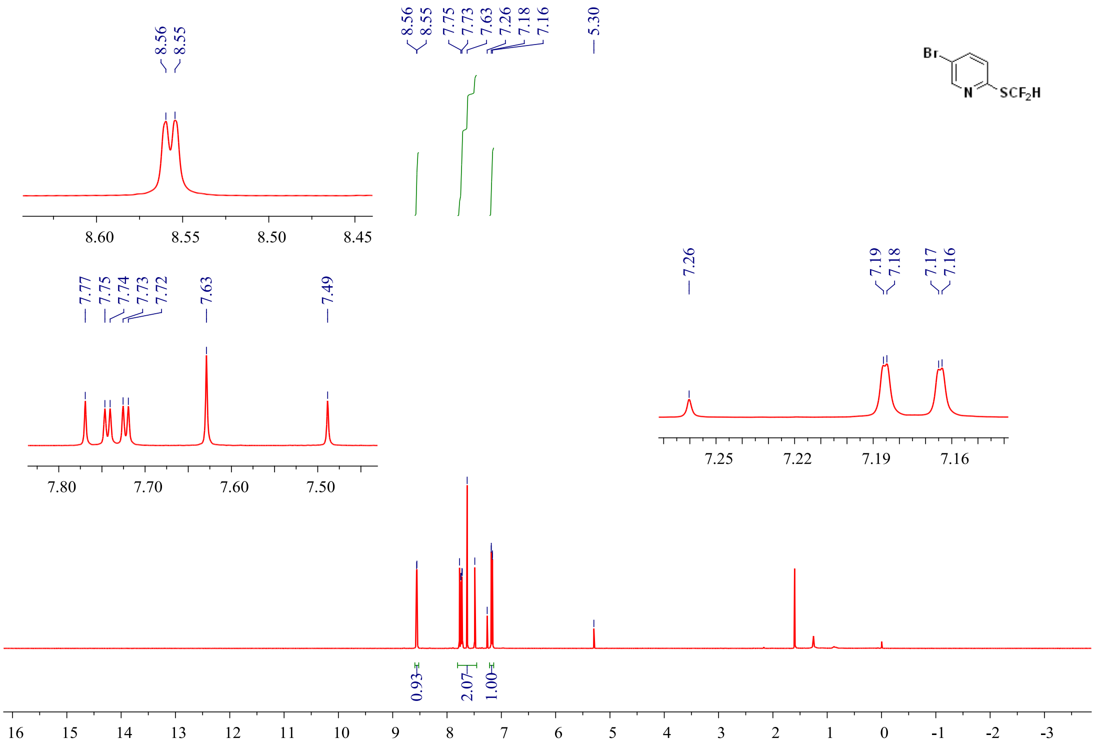

^{19}F NMR (376 MHz, $CDCl_3$) 5-bromo-2-((difluoromethyl)thio)pyridine 4a

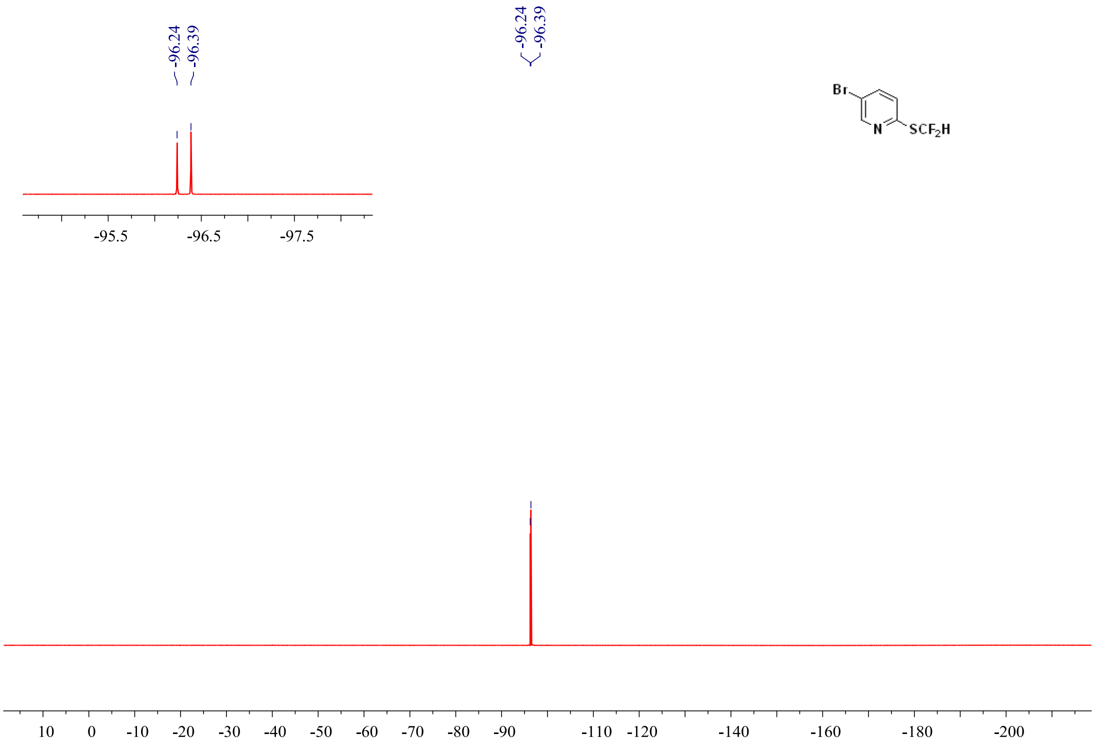

^{13}C NMR (101 MHz, $CDCl_3$) 5-bromo-2-((difluoromethyl)thio)pyridine 4a

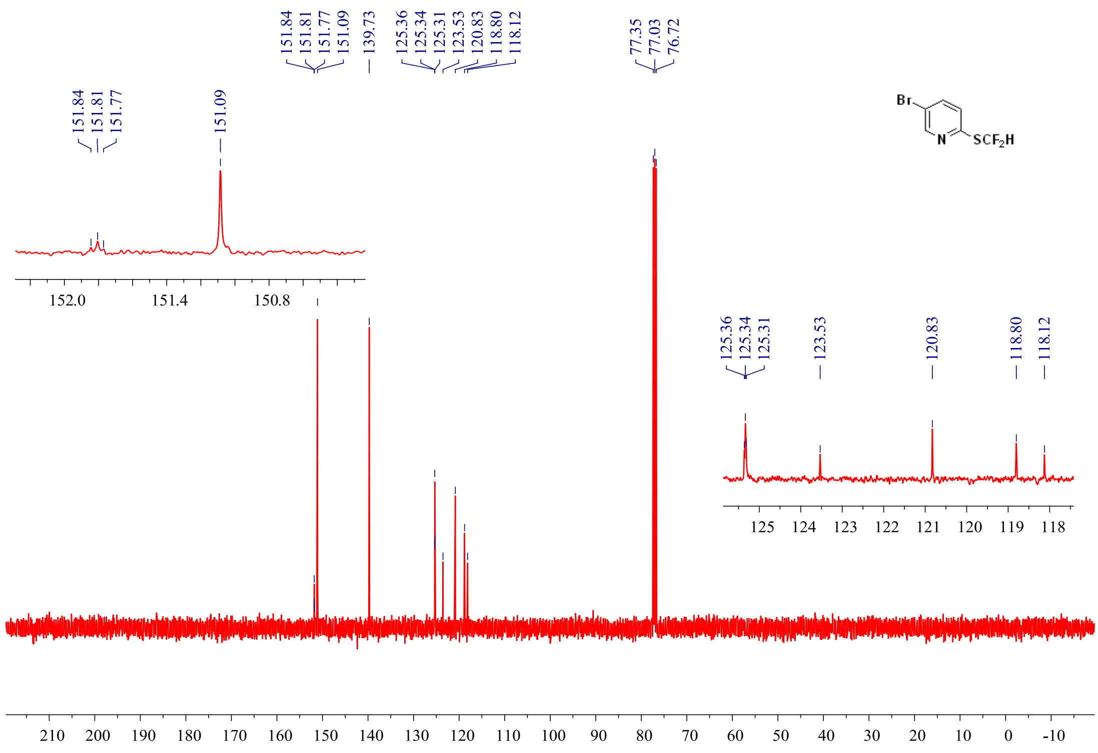

^{1}H NMR (400 MHz, $CDCl_3$) 5-chloro-2-((difluoromethyl)thio)pyridine 4b

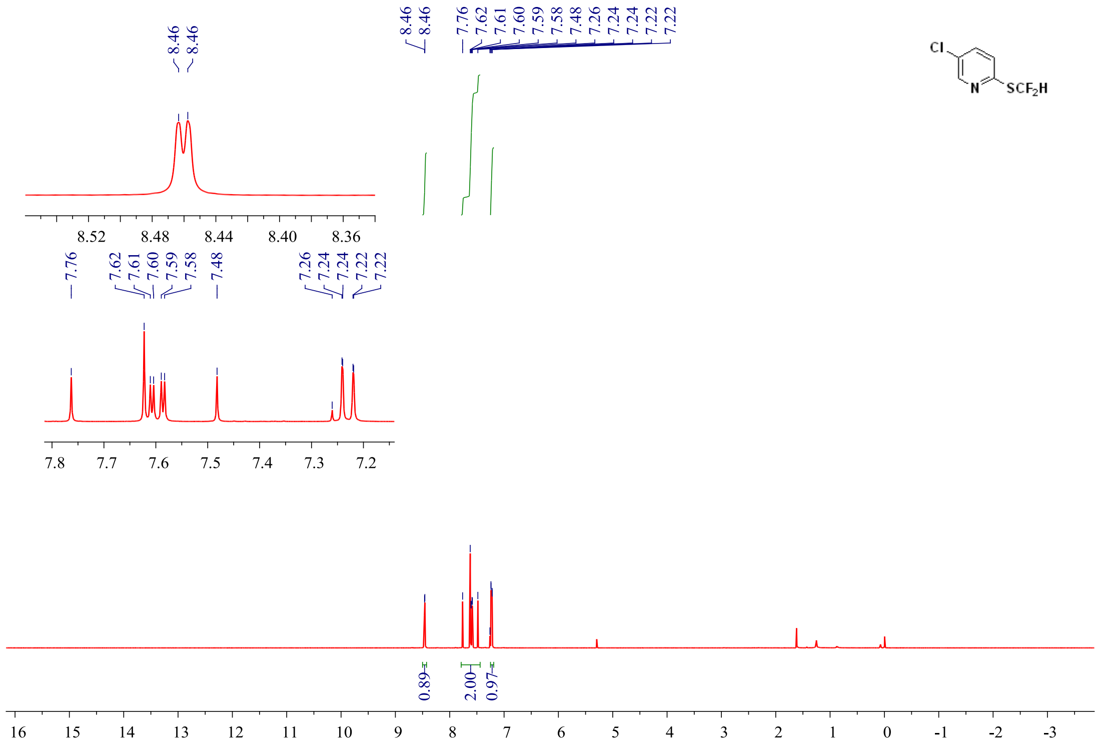

^{19}F NMR (376 MHz, $CDCl_3$) 5-chloro-2-((difluoromethyl)thio)pyridine 4b

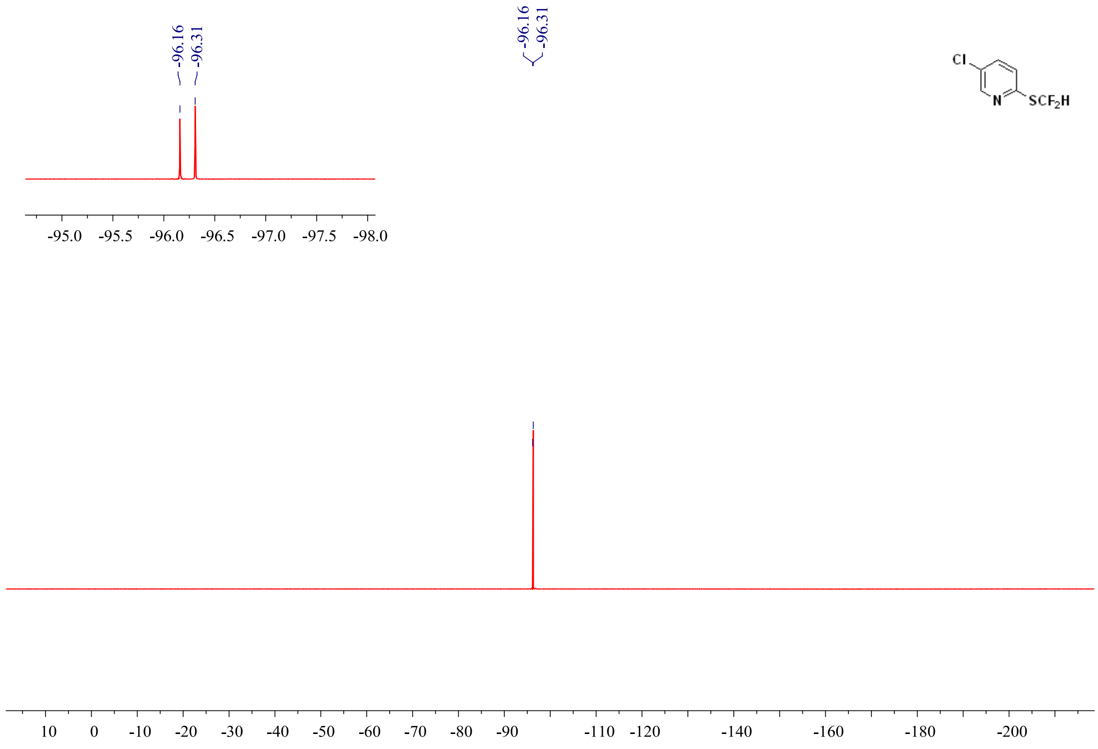

^{13}C NMR (101 MHz, $CDCl_3$) 5-chloro-2-((difluoromethyl)thio)pyridine 4b

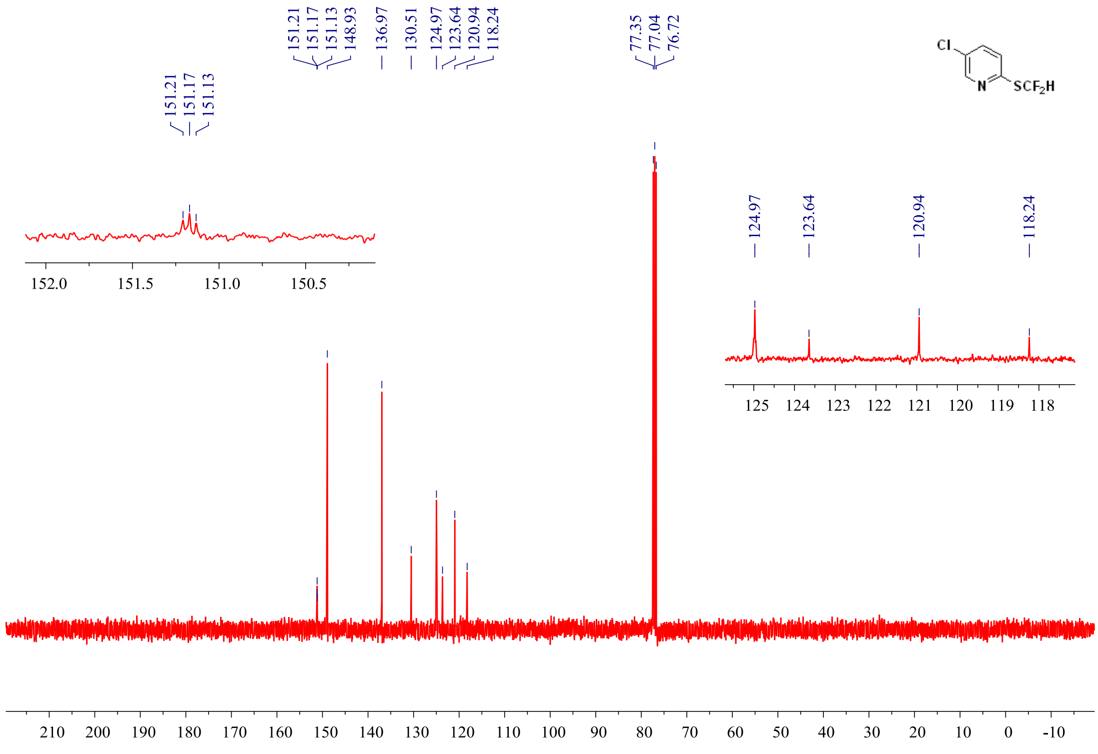

^{1}H NMR (400 MHz, $CDCl_3$) methyl 6-((difluoromethyl)thio)nicotinate 4c

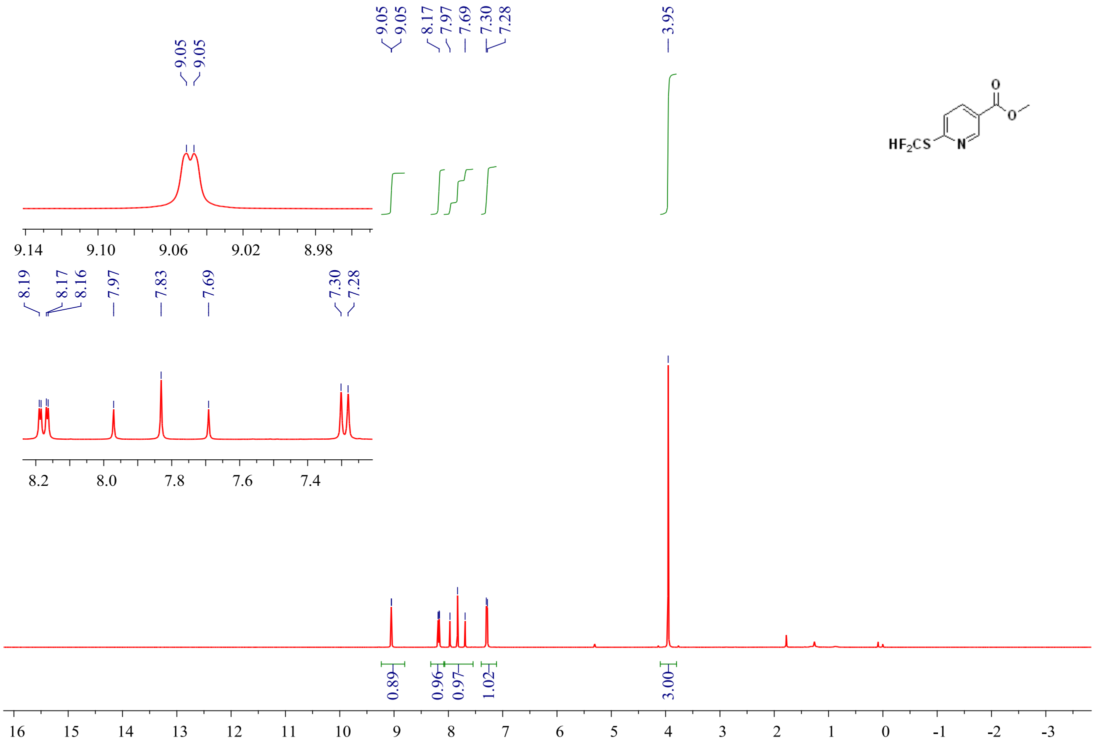

^{19}F NMR (376 MHz, $CDCl_3$) methyl 6-((difluoromethyl)thio) nicotinate 4c

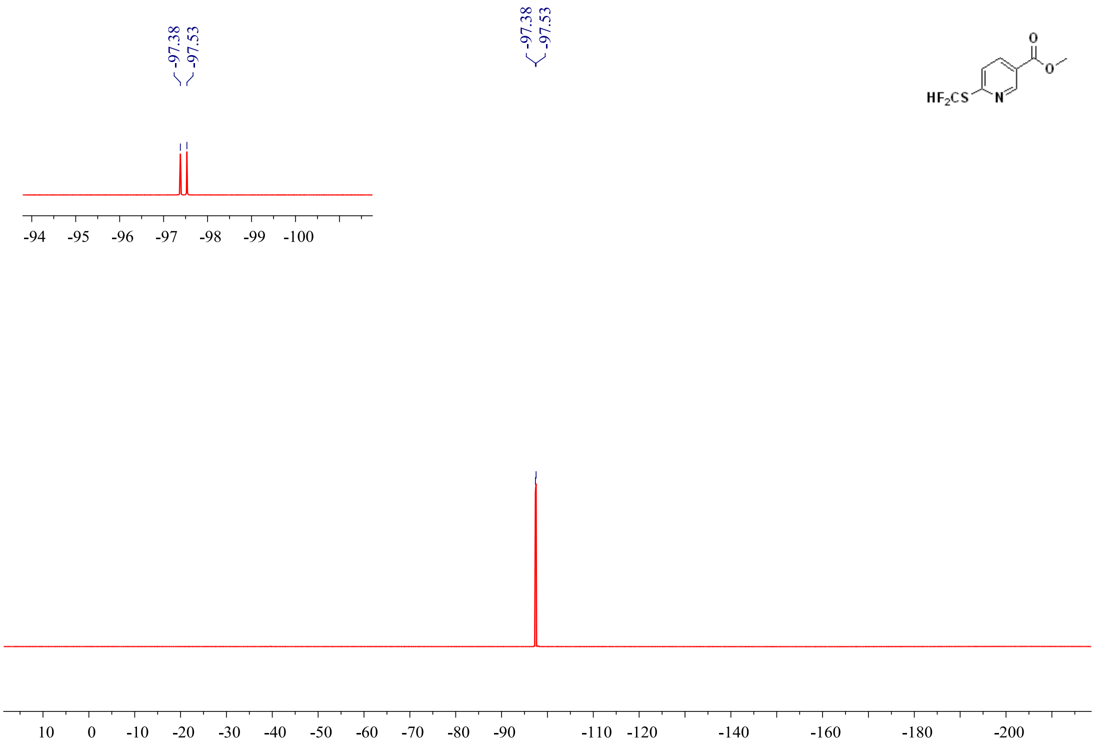

^{13}C NMR (101 MHz, $CDCl_3$) methyl 6-((difluoromethyl)thio) nicotinate 4c

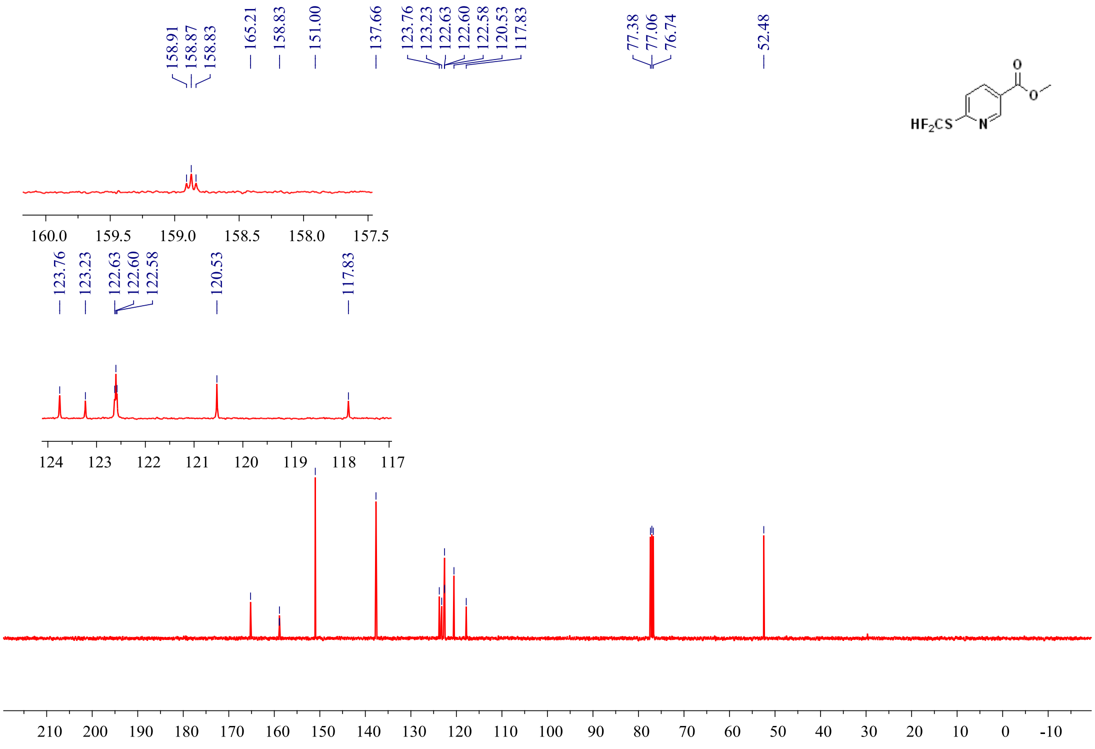

^{1}H NMR (400 MHz, $CDCl_3$) 2-((difluoromethyl)thio)isonicotinonitrile 4d

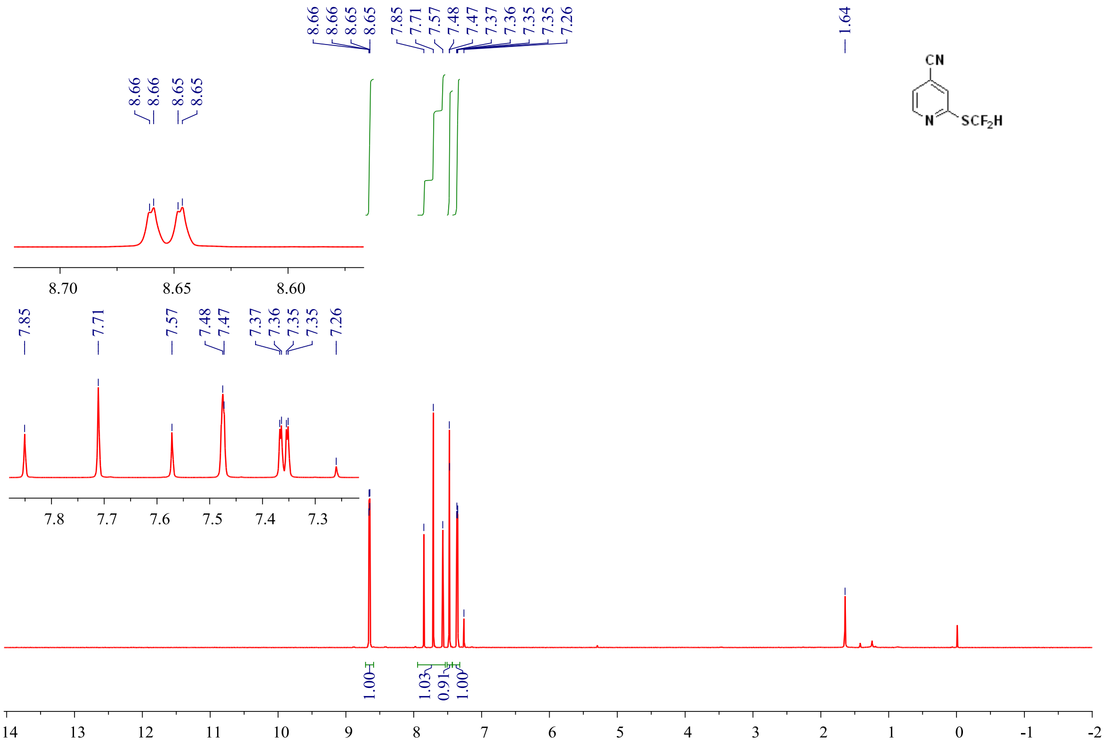

^{19}F NMR (376 MHz, $CDCl_3$) 2-((difluoromethyl)thio)isonicotinonitrile 4d

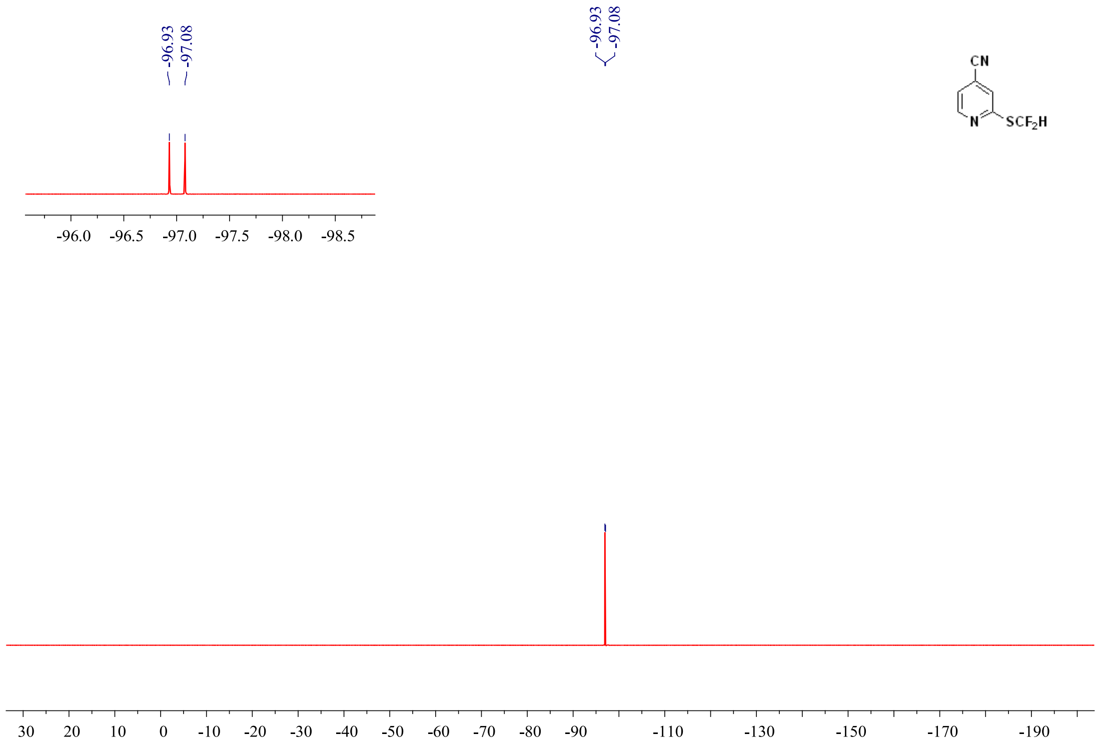

^{13}C NMR (101 MHz, $CDCl_3$) 2-((difluoromethyl)thio)isonicotinonitrile 4d

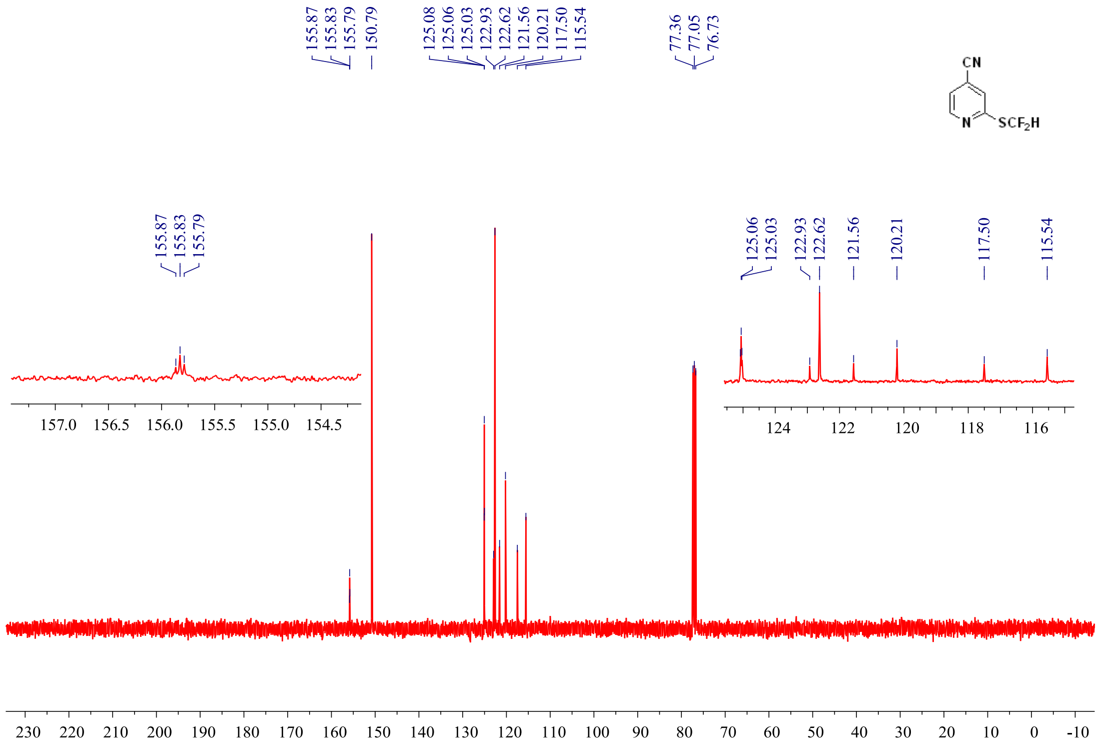

^{1}H NMR (400 MHz, $CDCl_3$) 2-((difluoromethyl)thio)-5-nitropyridine 4e

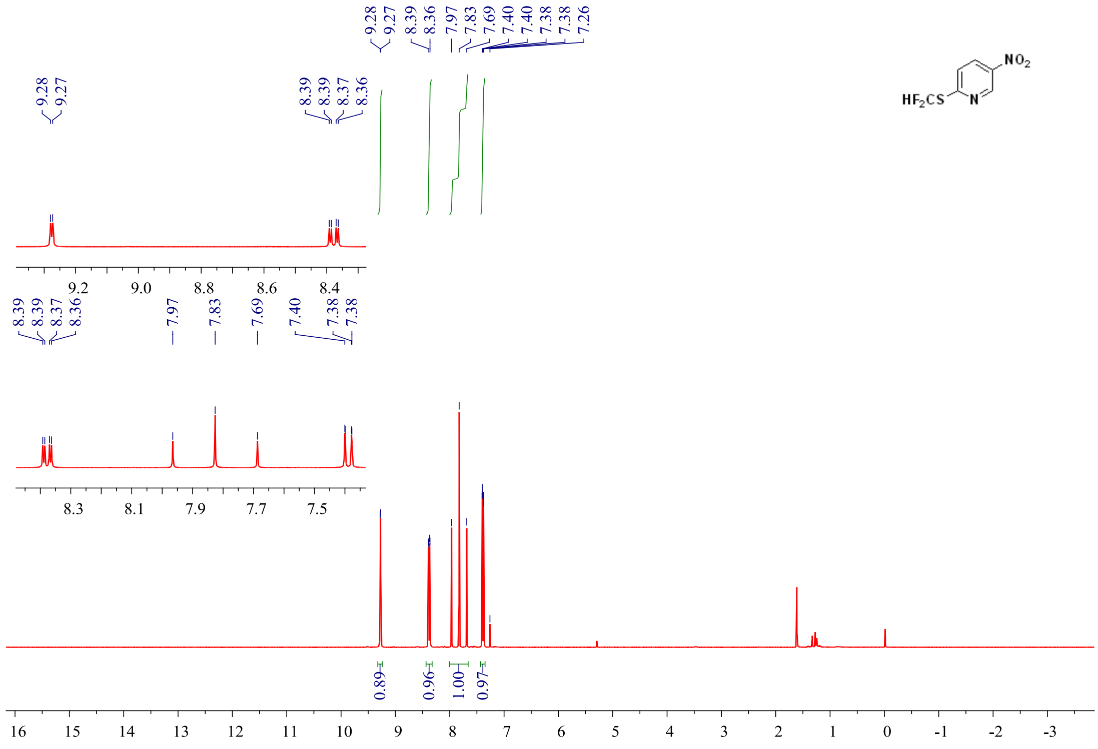

^{19}F NMR (376 MHz, $CDCl_3$) 2-((difluoromethyl)thio)-5-nitropyridine 4e

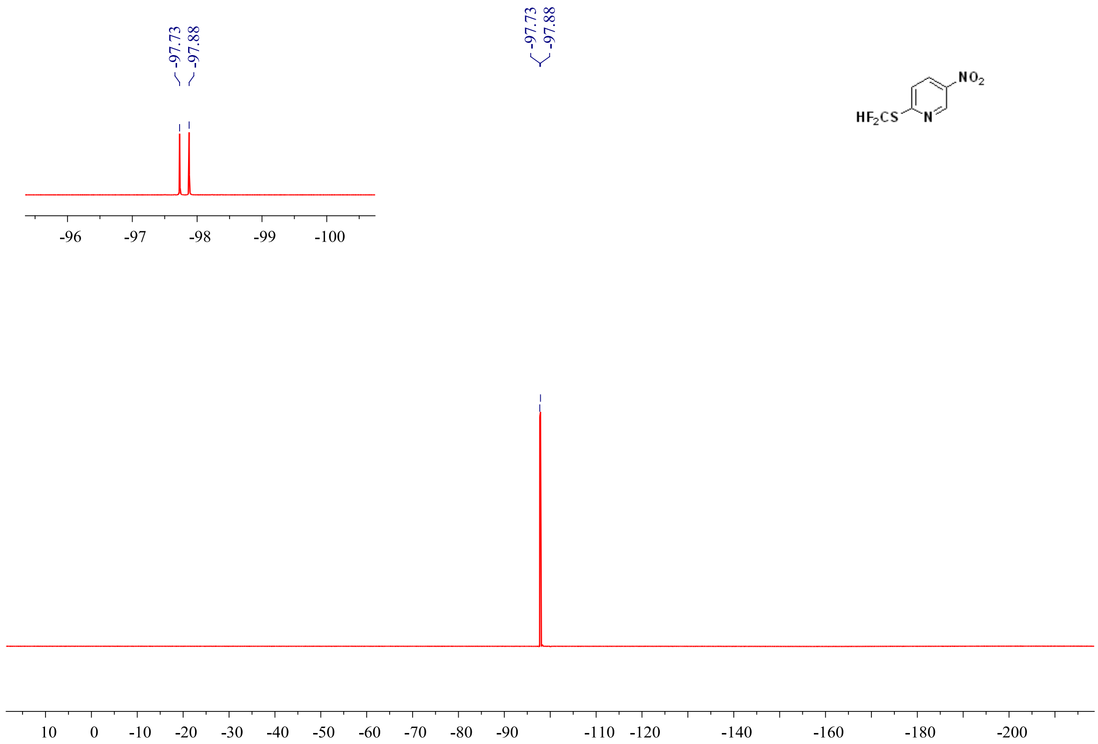

^{13}C NMR (101 MHz, $CDCl_3$) 2-((difluoromethyl)thio)-5-nitropyridine 4e

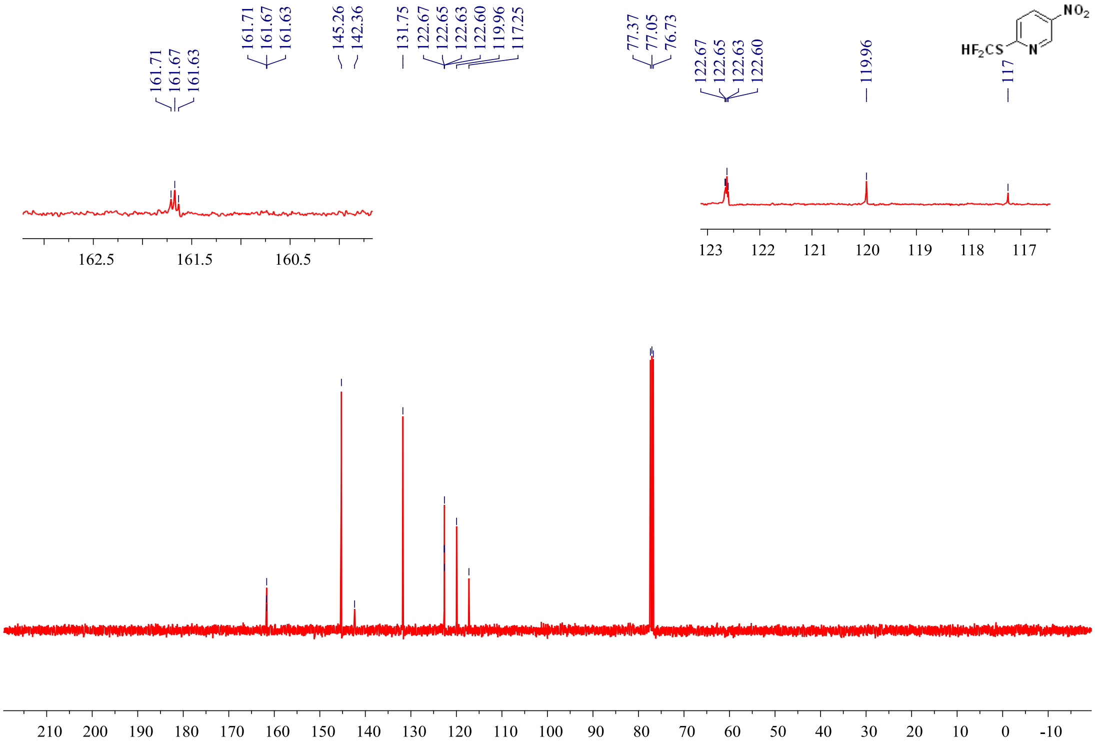

^{1}H NMR (400 MHz, $CDCl_3$) 3-((difluoromethyl)thio)pyridine 4f

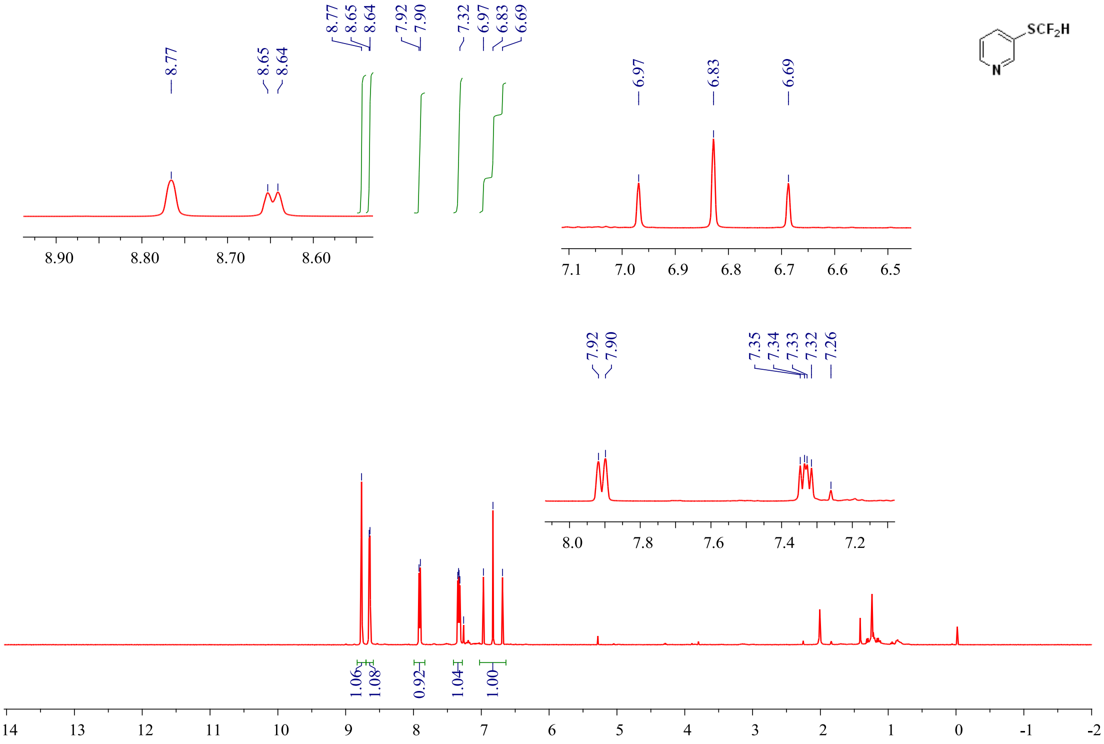

^{19}F NMR (376 MHz, $CDCl_3$) 3-((difluoromethyl)thio)pyridine 4f

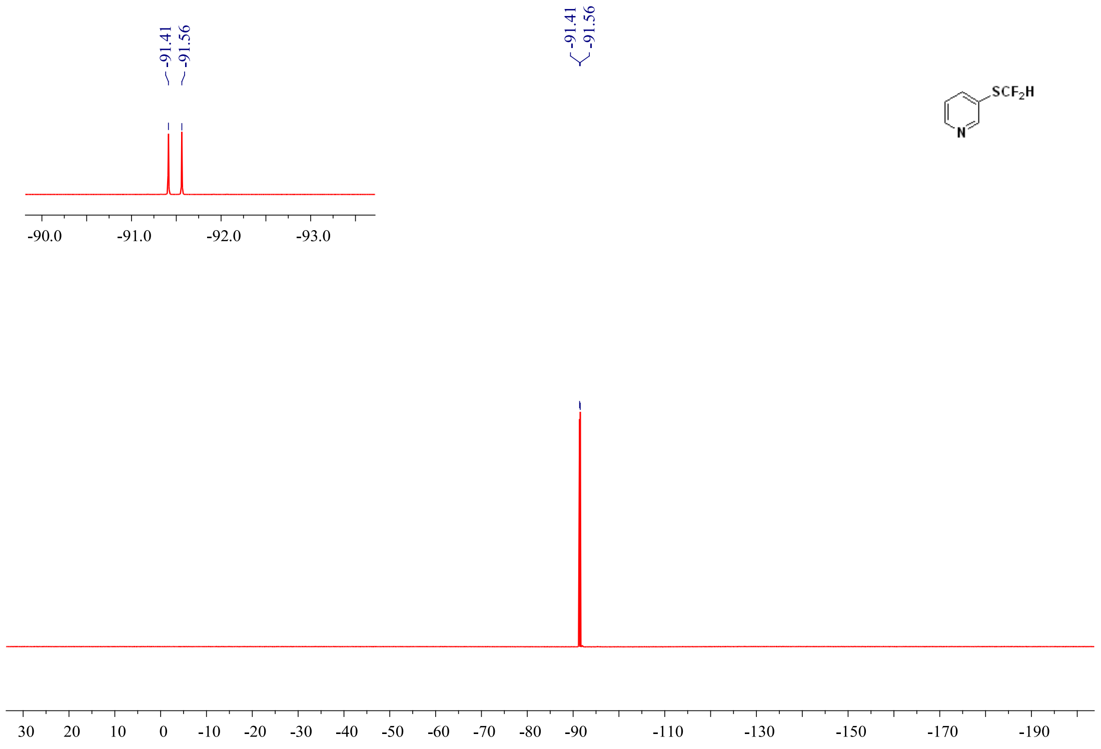

^{13}C NMR (101 MHz, $CDCl_3$) 3-((difluoromethyl)thio)pyridine 4f

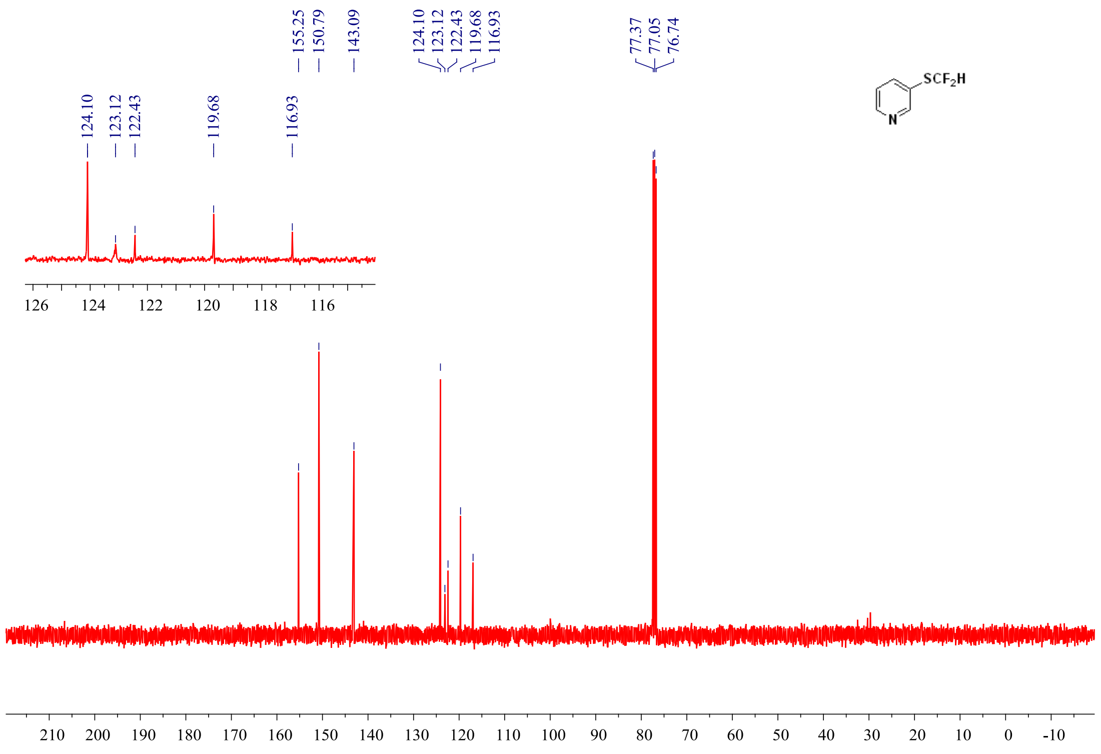

^{1}H NMR (400 MHz, $CDCl_3$) 2-chloro-5-((difluoromethyl)thio)pyridine 4g

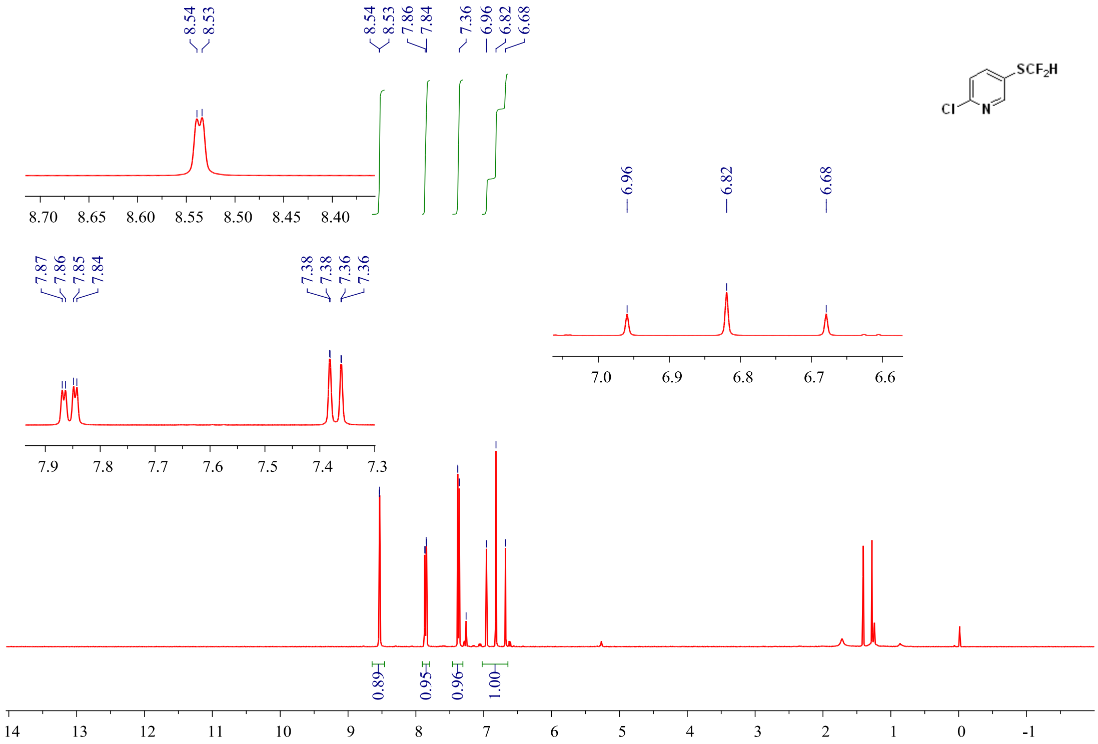

^{19}F NMR (376 MHz, $CDCl_3$) 2-chloro-5-((difluoromethyl)thio)pyridine 4g

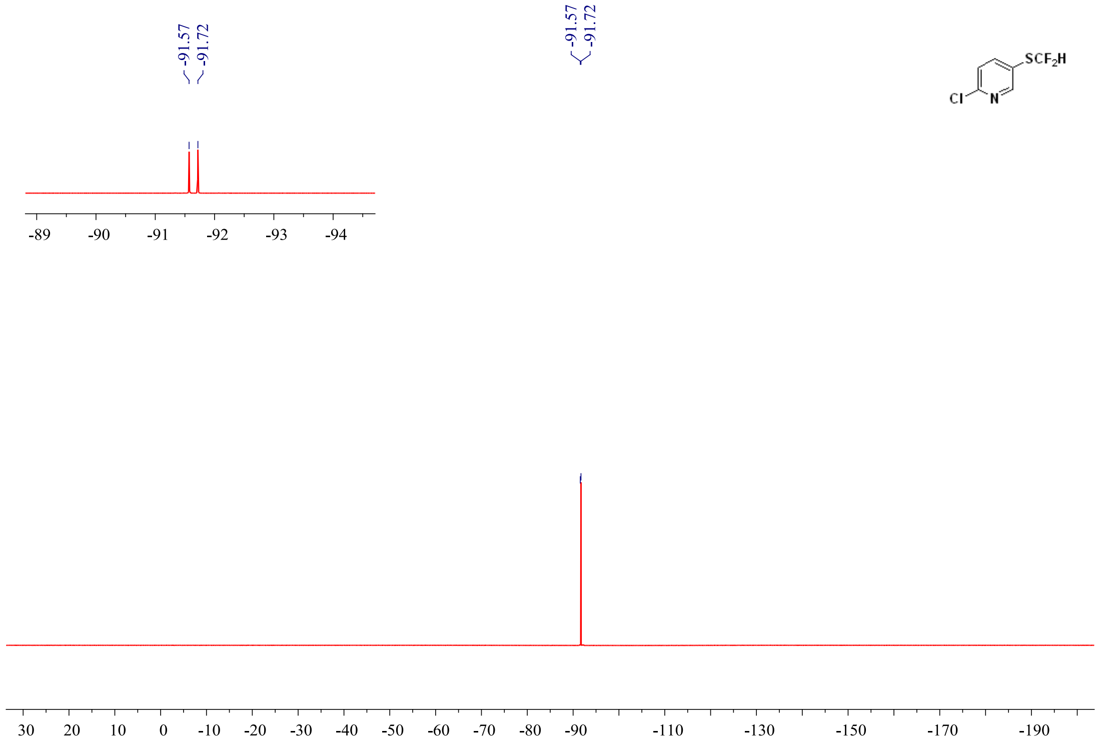

^{13}C NMR (101 MHz, $CDCl_3$) 2-chloro-5-((difluoromethyl)thio)pyridine 4g

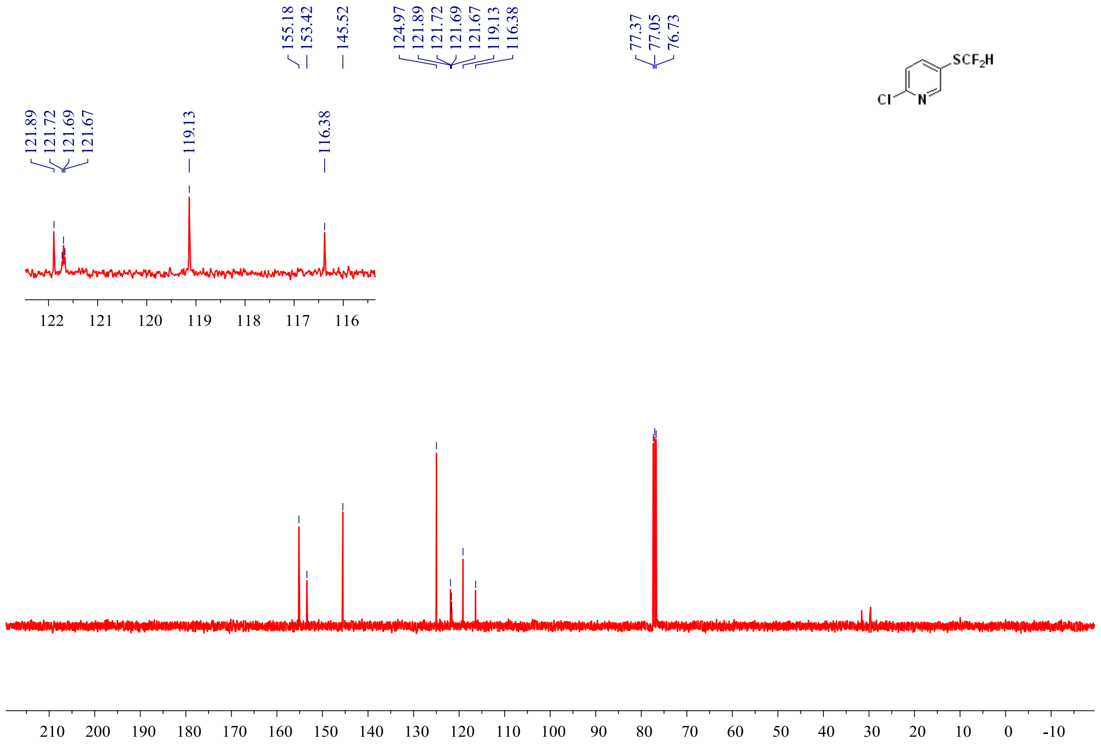

^{1}H NMR (400 MHz, $CDCl_3$) 5-((difluoromethyl)thio)-2-fluoropyridine 4h

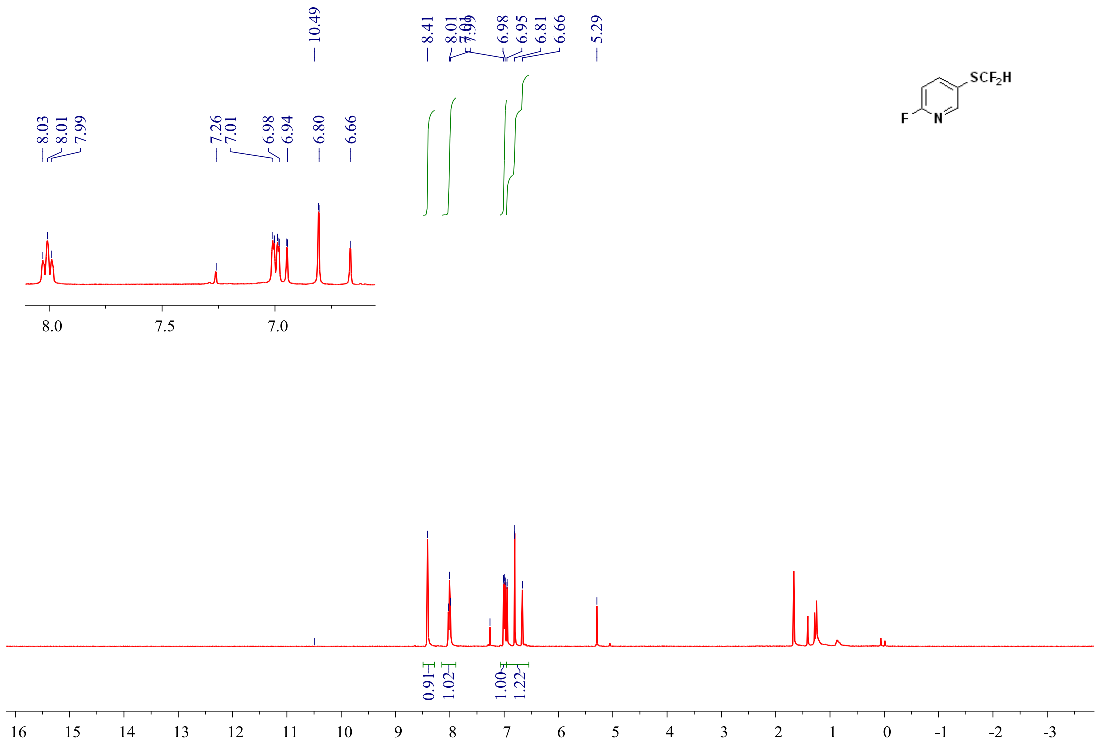

^{19}F NMR (376 MHz, $CDCl_3$) 5-((difluoromethyl)thio)-2-fluoropyridine 4h

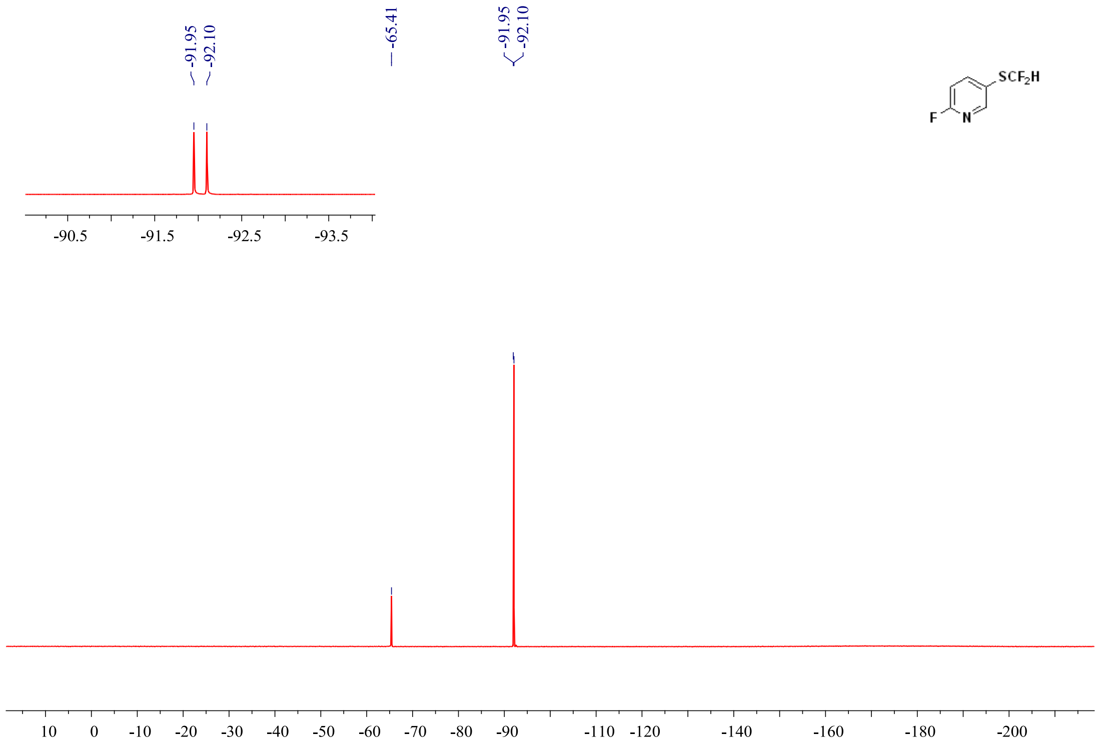

^{13}C NMR (101 MHz, $CDCl_3$) 5-((difluoromethyl)thio)-2-fluoropyridine 4h

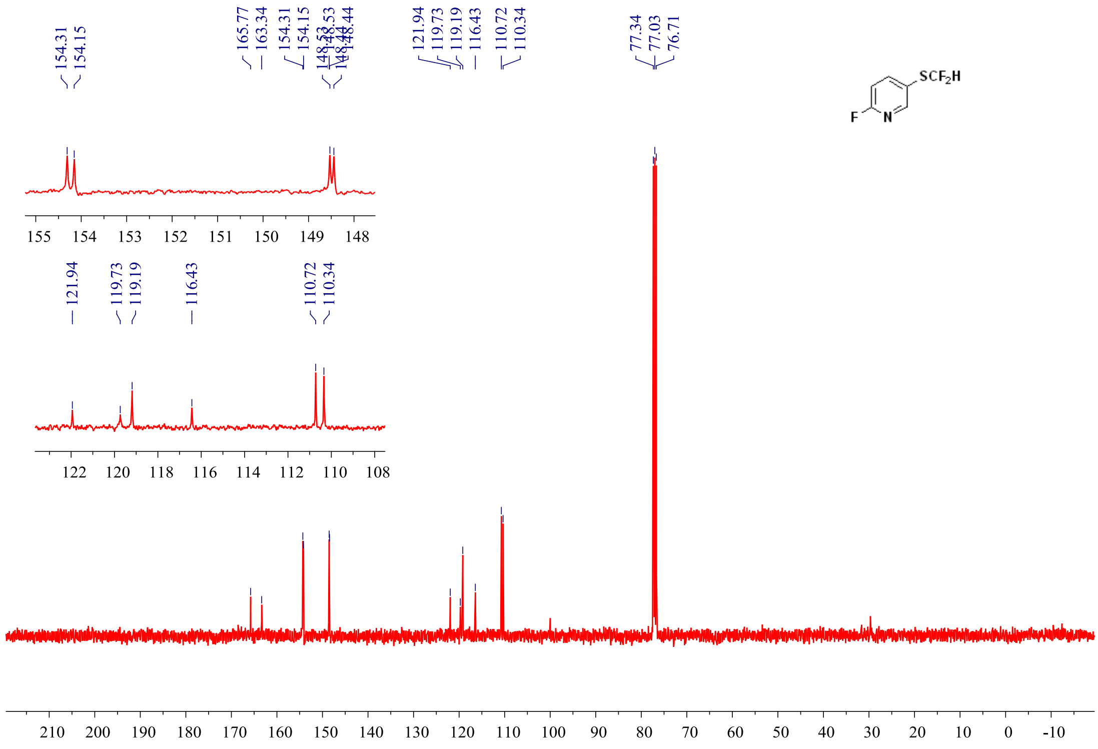

^{1}H NMR (400 MHz, $CDCl_3$) 5-bromo-3-((difluoromethyl)thio)-2-methoxy pyridine 4i

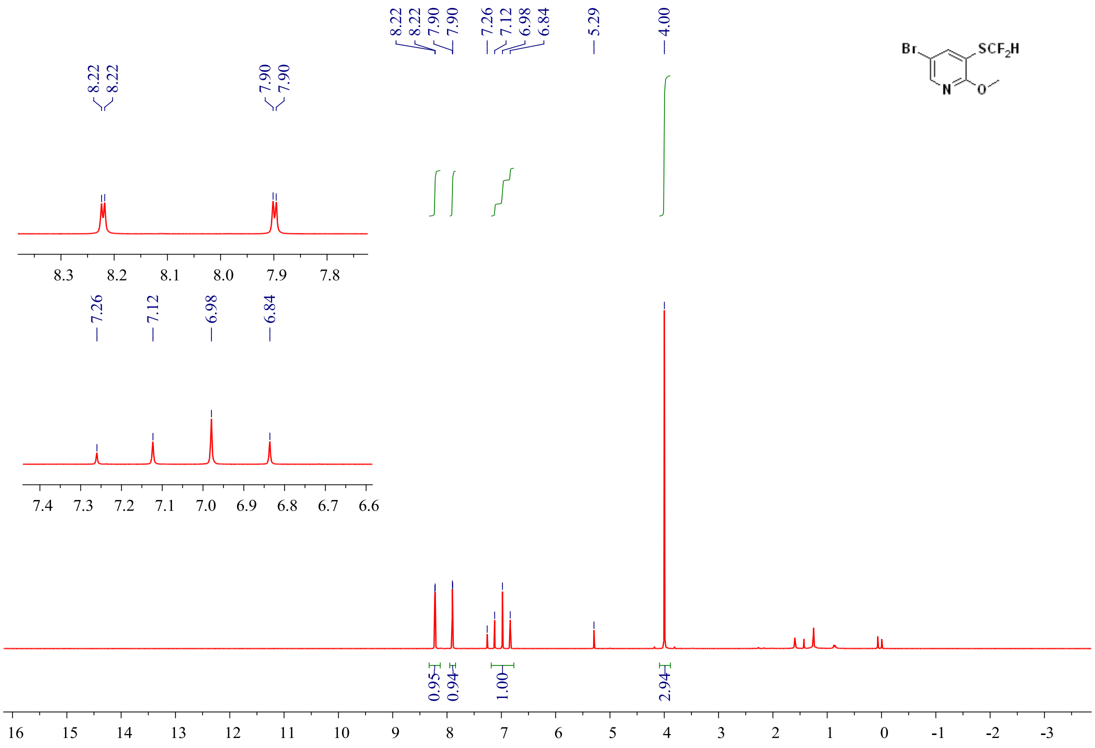

^{19}F NMR (376 MHz, $CDCl_3$) 5-bromo-3-((difluoromethyl)thio)-2-methoxy pyridine 4i

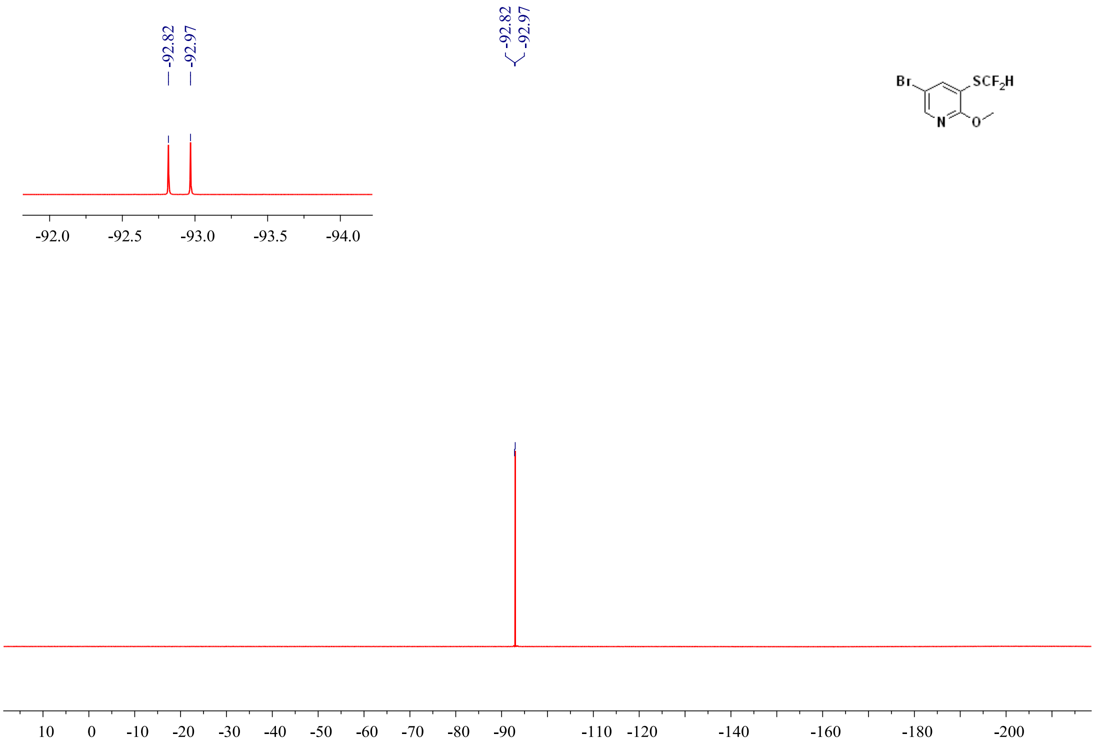

^{13}C NMR (101 MHz, $CDCl_3$) 5-bromo-3-((difluoromethyl)thio)-2-methoxy pyridine 4i

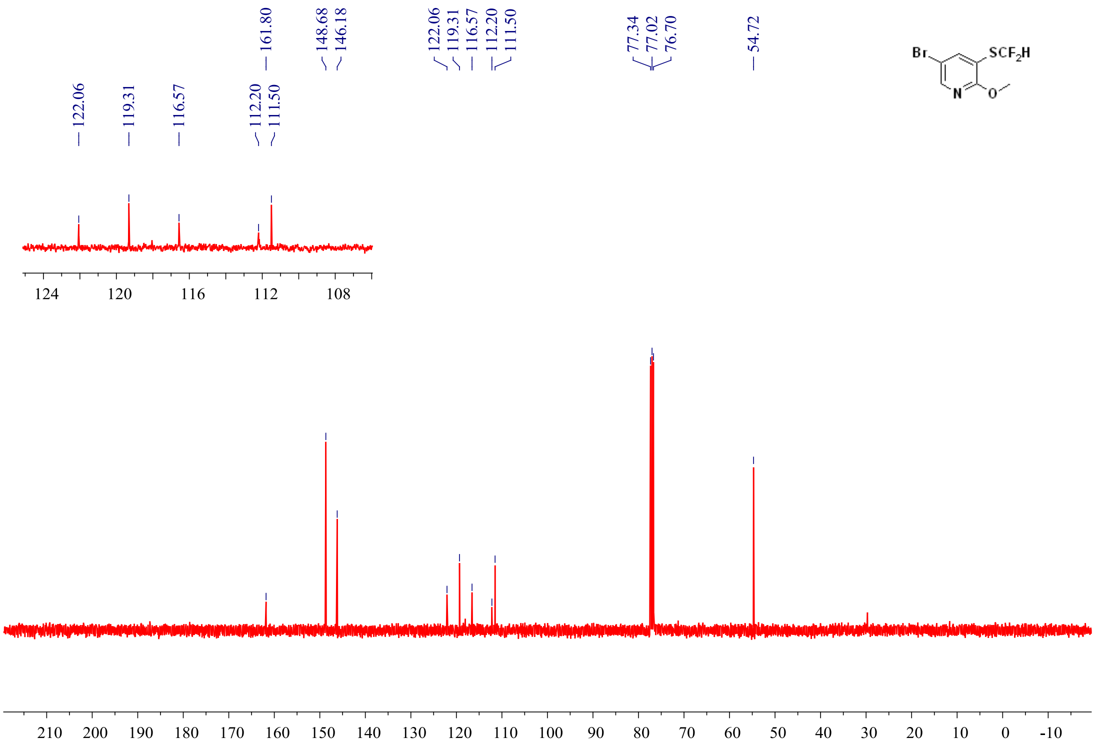

^{1}H NMR (400 MHz, $CDCl_3$) 3-((difluoromethyl)thio)-2-methoxypyridine 4j

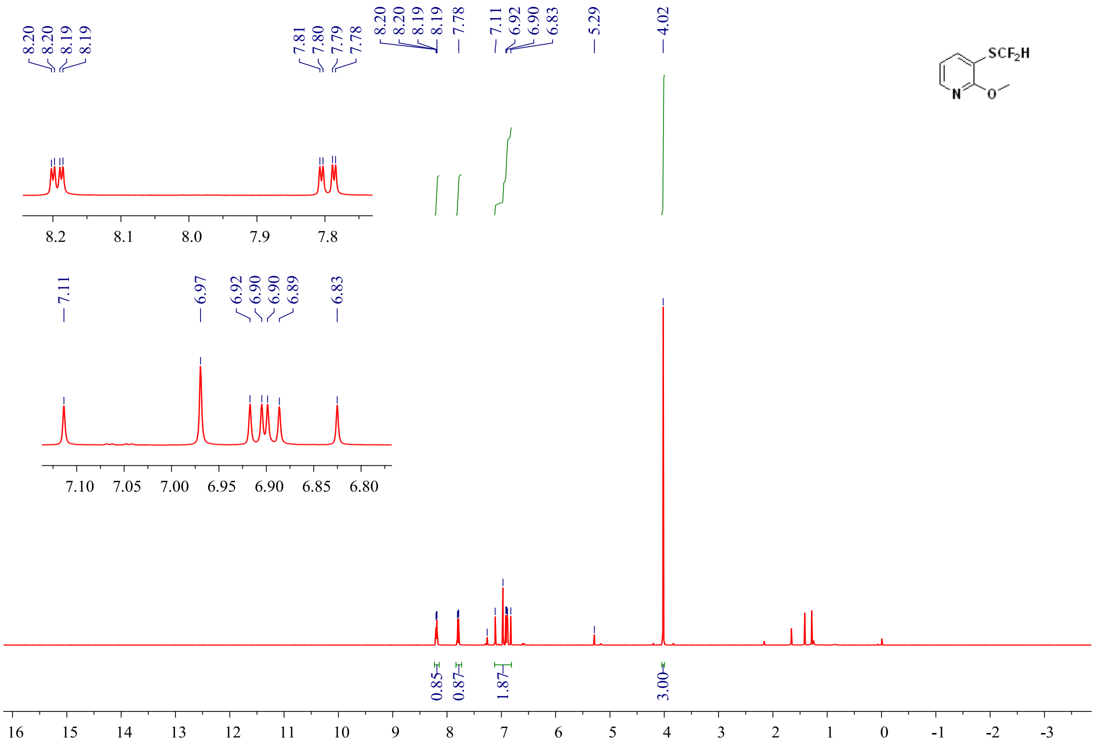

^{19}F NMR (376 MHz, $CDCl_3$) 3-((difluoromethyl)thio)-2-methoxypyridine 4j

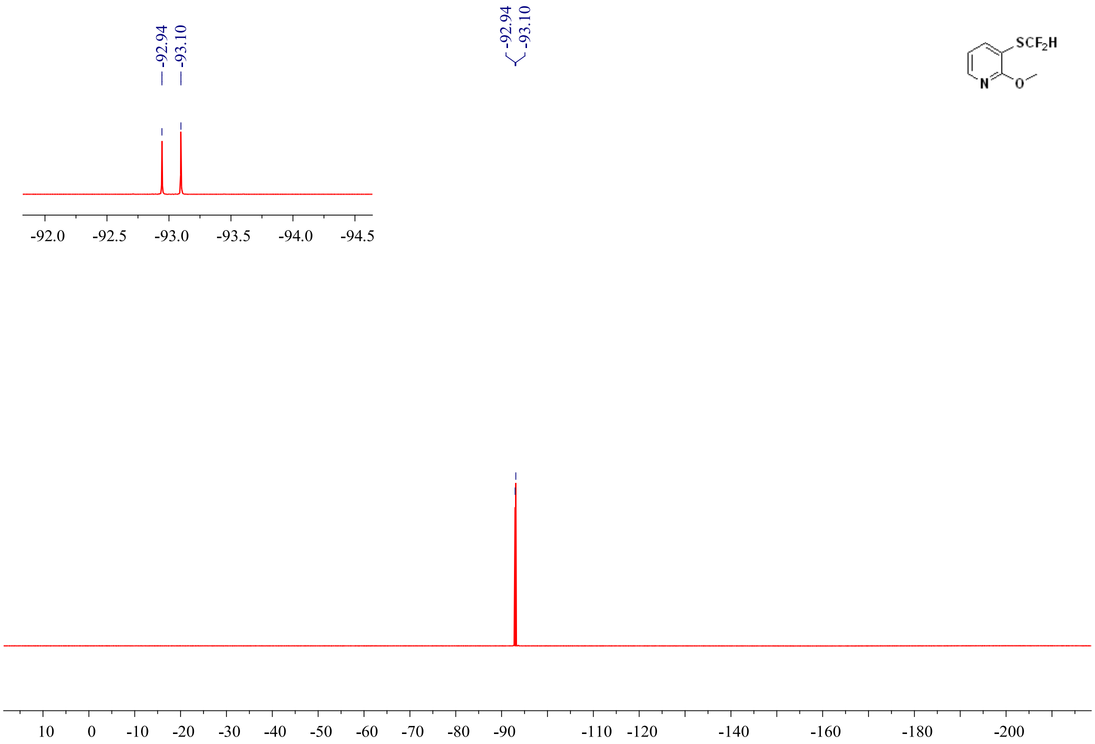

^{13}C NMR (101 MHz, $CDCl_3$) 3-((difluoromethyl)thio)-2-methoxypyridine 4j

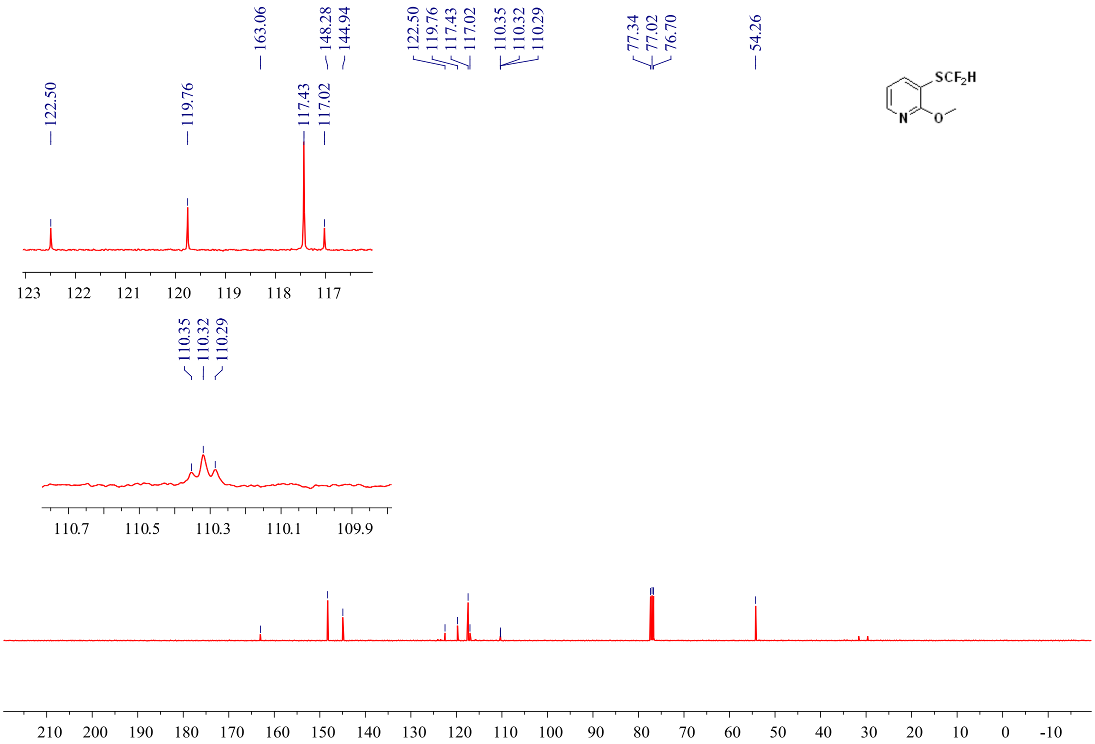

^{1}H NMR (400 MHz, $CDCl_3$) 3-((difluoromethyl)thio)-2-nitro pyridine 4k

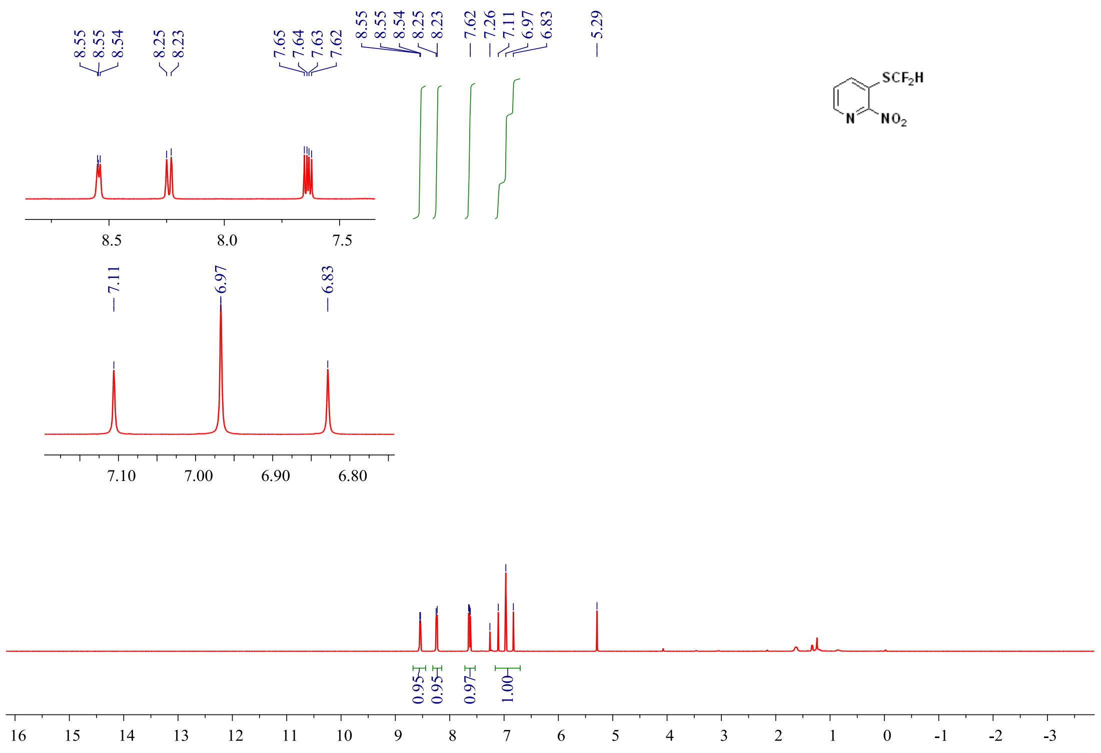

^{19}F NMR (376 MHz, $CDCl_3$) 3-((difluoromethyl)thio)-2-nitro pyridine 4k

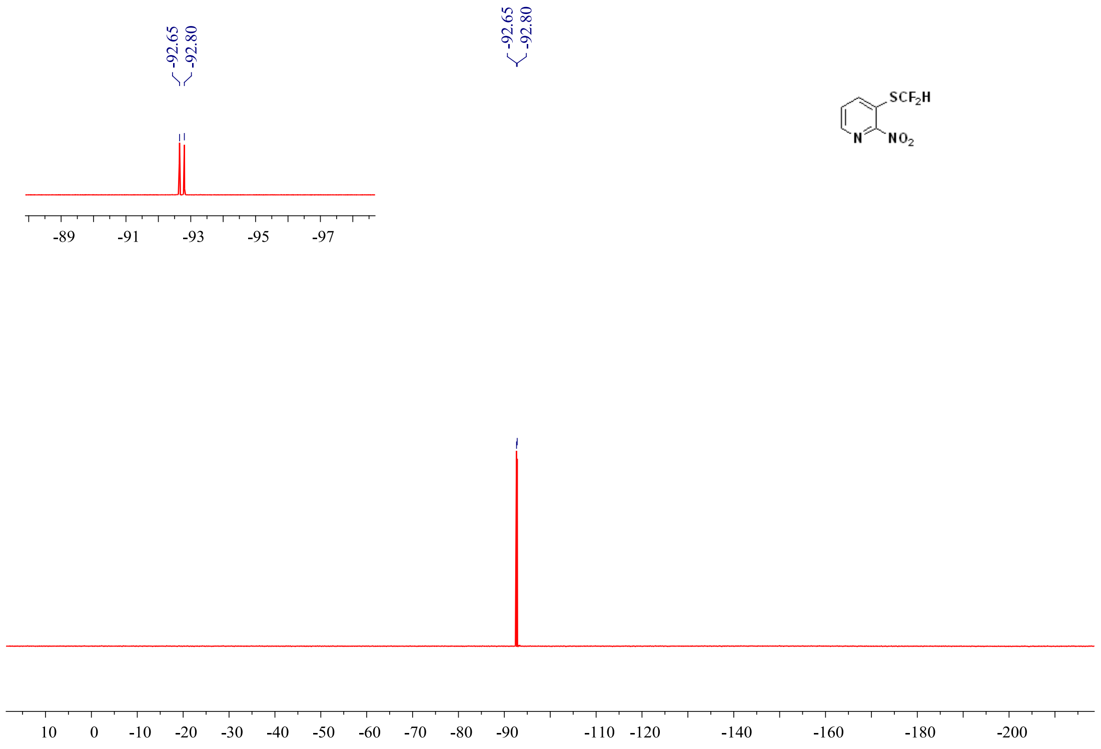

^{13}C NMR (101 MHz, $CDCl_3$) 3-((difluoromethyl)thio)-2-nitro pyridine 4k

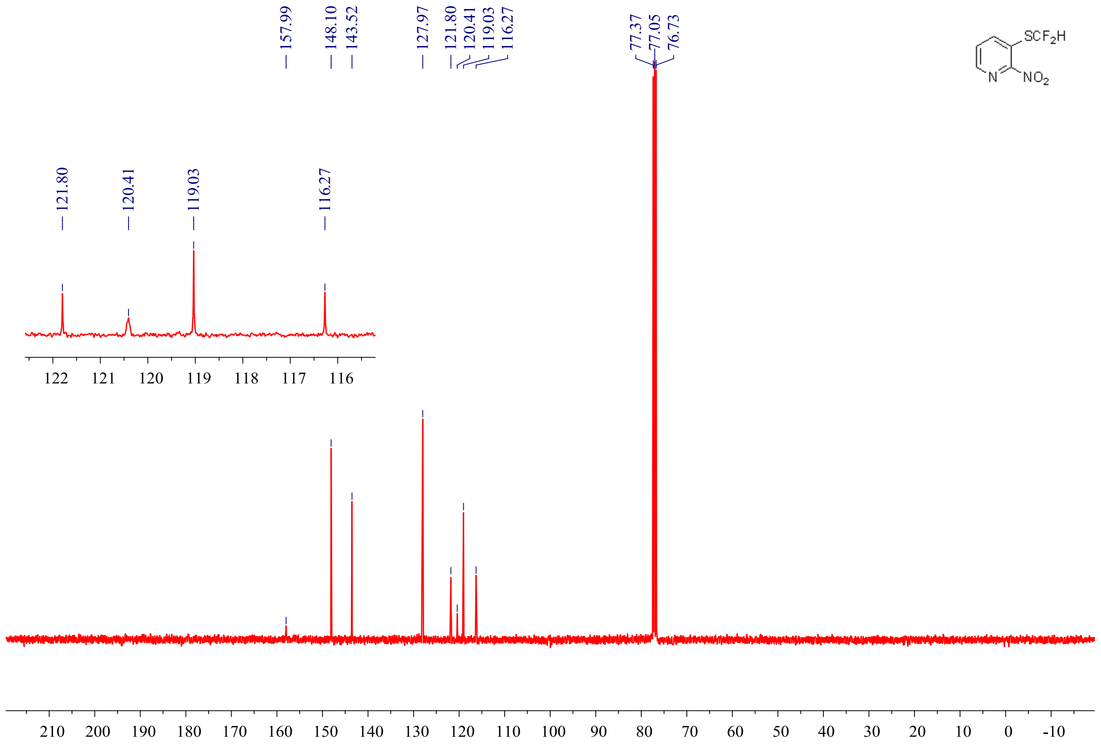

^{1}H NMR (400 MHz, $CDCl_3$) 4-(5-((difluoromethyl)thio)pyridin-2-yl)morpholine 4l

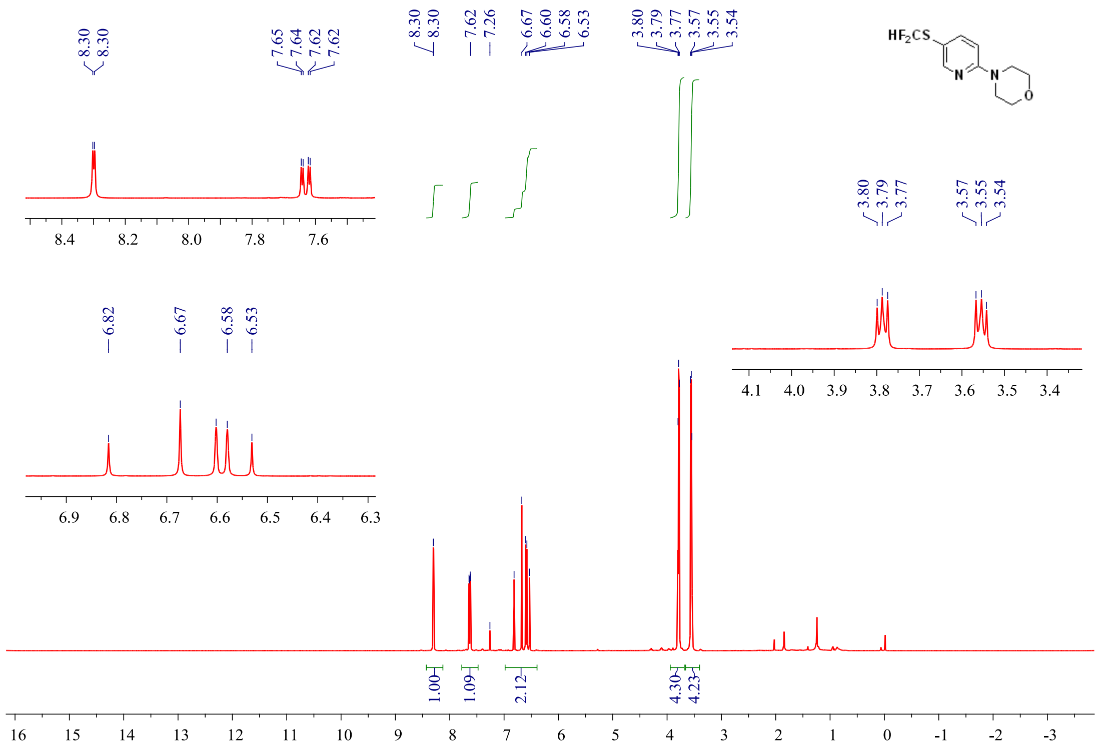

^{19}F NMR (376 MHz, $CDCl_3$) 4-(5-((difluoromethyl)thio)pyridin-2-yl)morpholine 4l

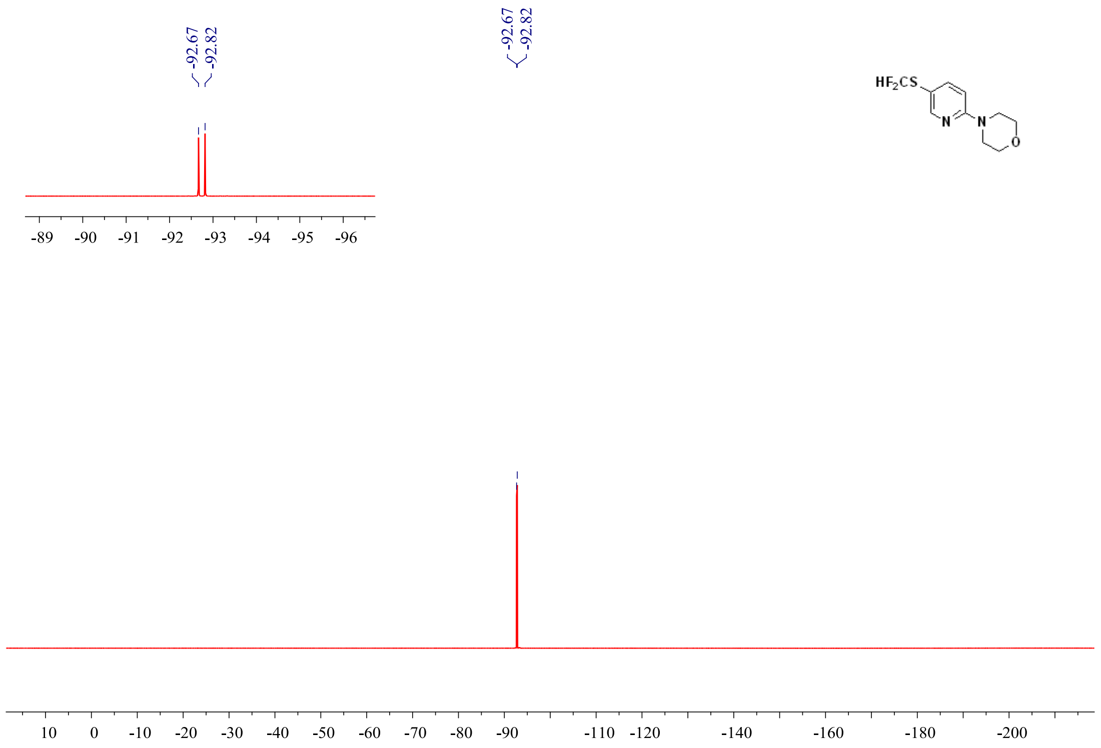

^{13}C NMR (101 MHz, $CDCl_3$) 4-(5-((difluoromethyl)thio)pyridin-2-yl)morpholine 4l

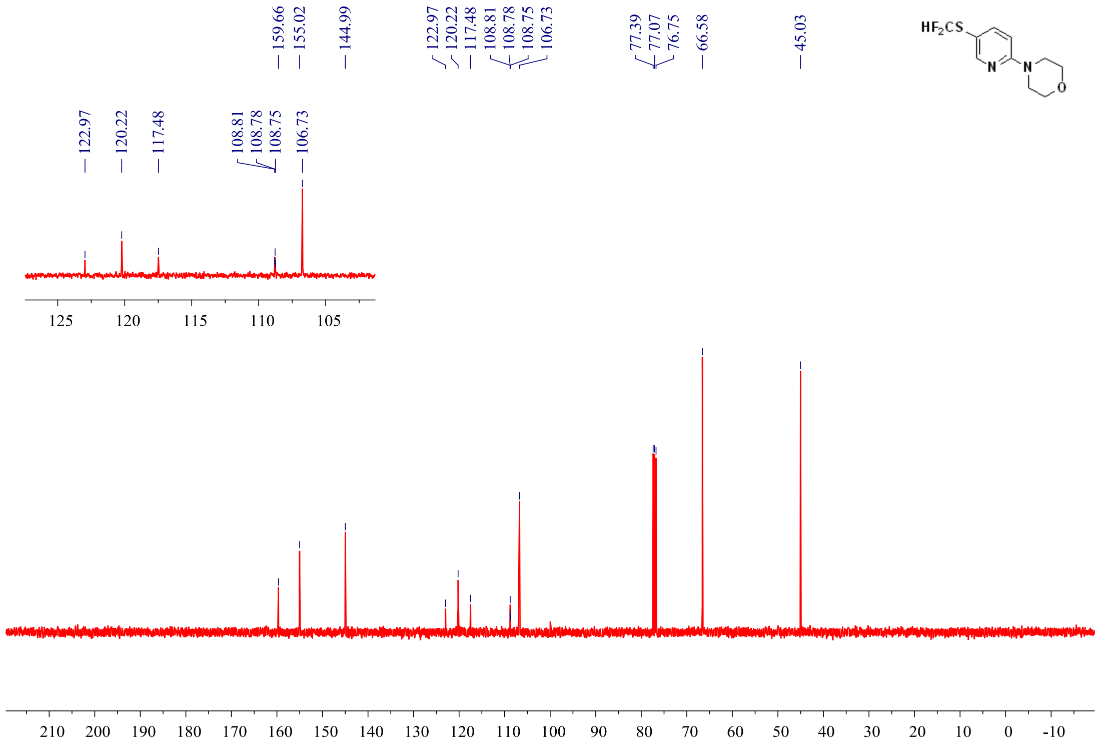

^{1}H NMR (400 MHz, $CDCl_3$) 2-chloro-4-((difluoromethyl)thio)pyridine 4m

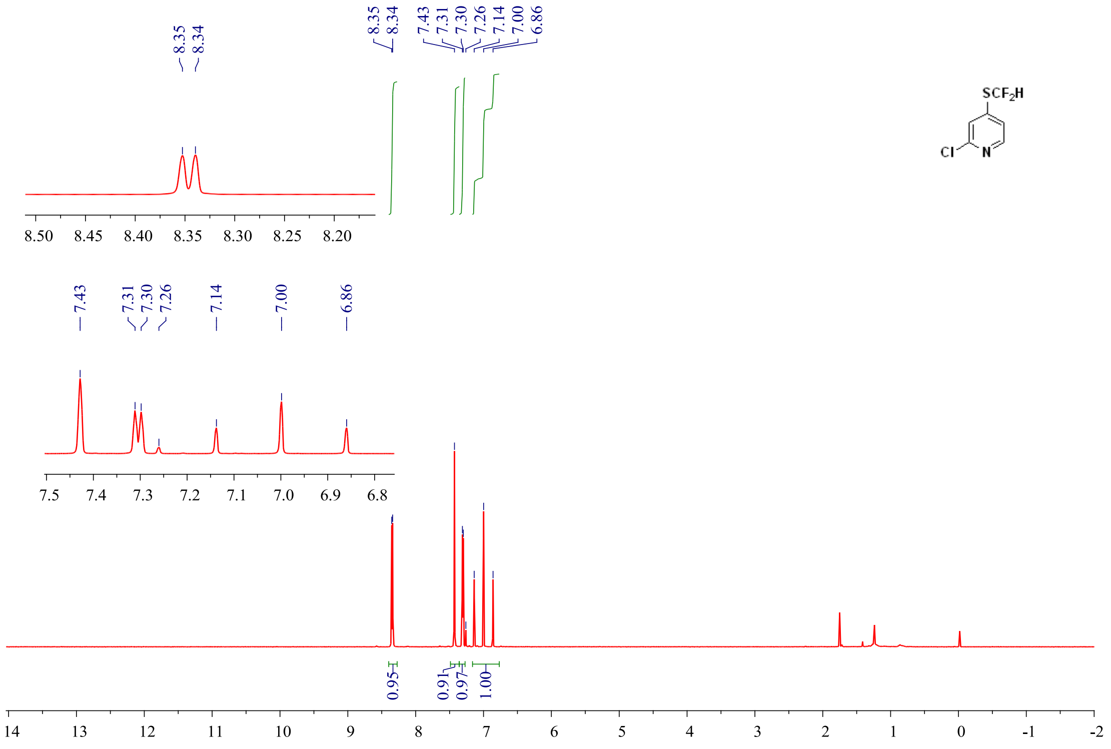

^{19}F NMR (376 MHz, $CDCl_3$) 2-chloro-4-((difluoromethyl)thio)pyridine 4m

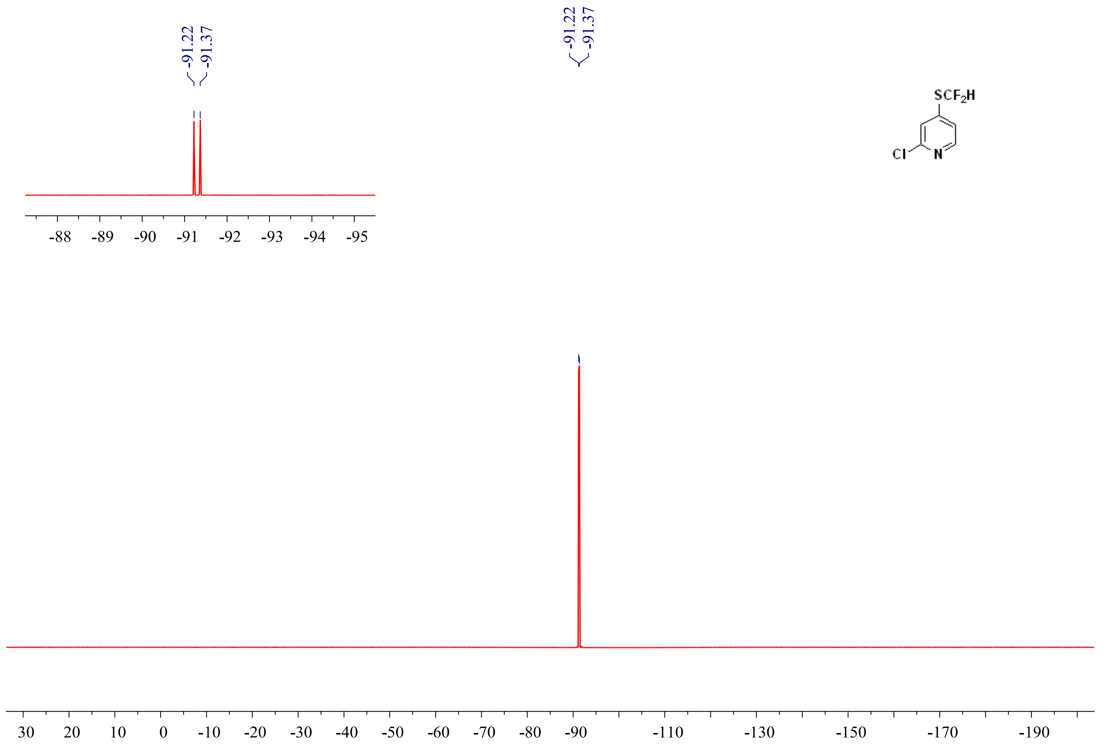

^{13}C NMR (101 MHz, $CDCl_3$) 2-chloro-4-((difluoromethyl)thio)pyridine 4m

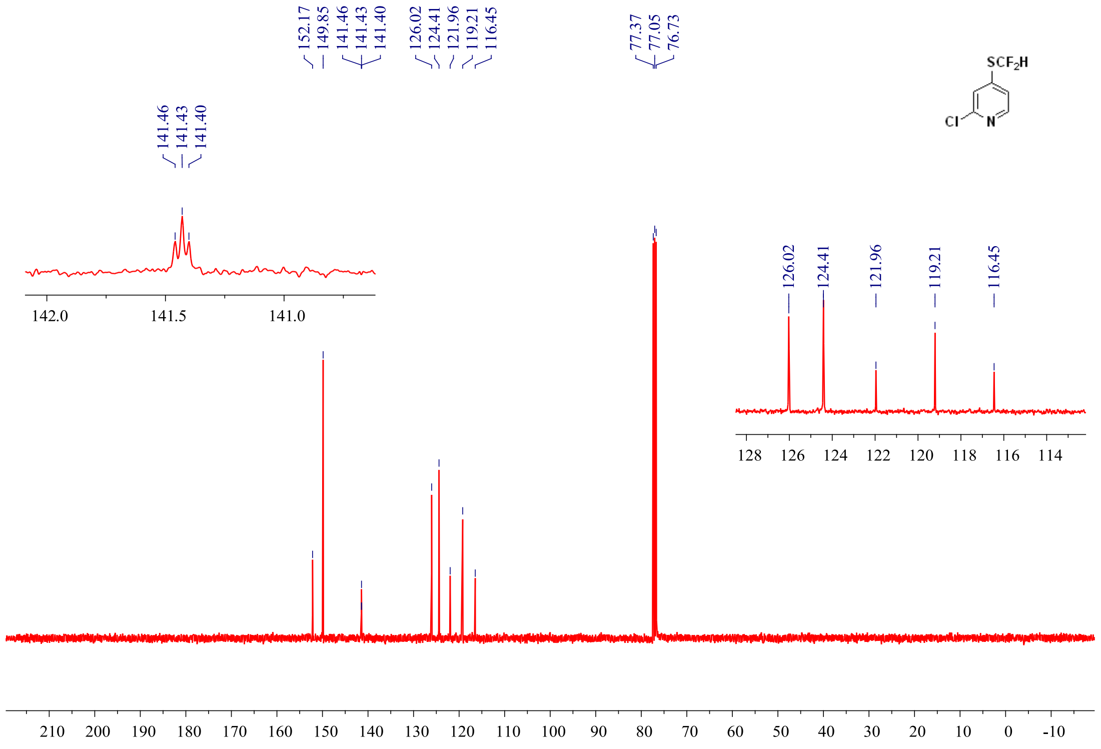

^{1}H NMR (400 MHz, $CDCl_3$) 2-bromo-4-((difluoromethyl)thio)pyridine 4n

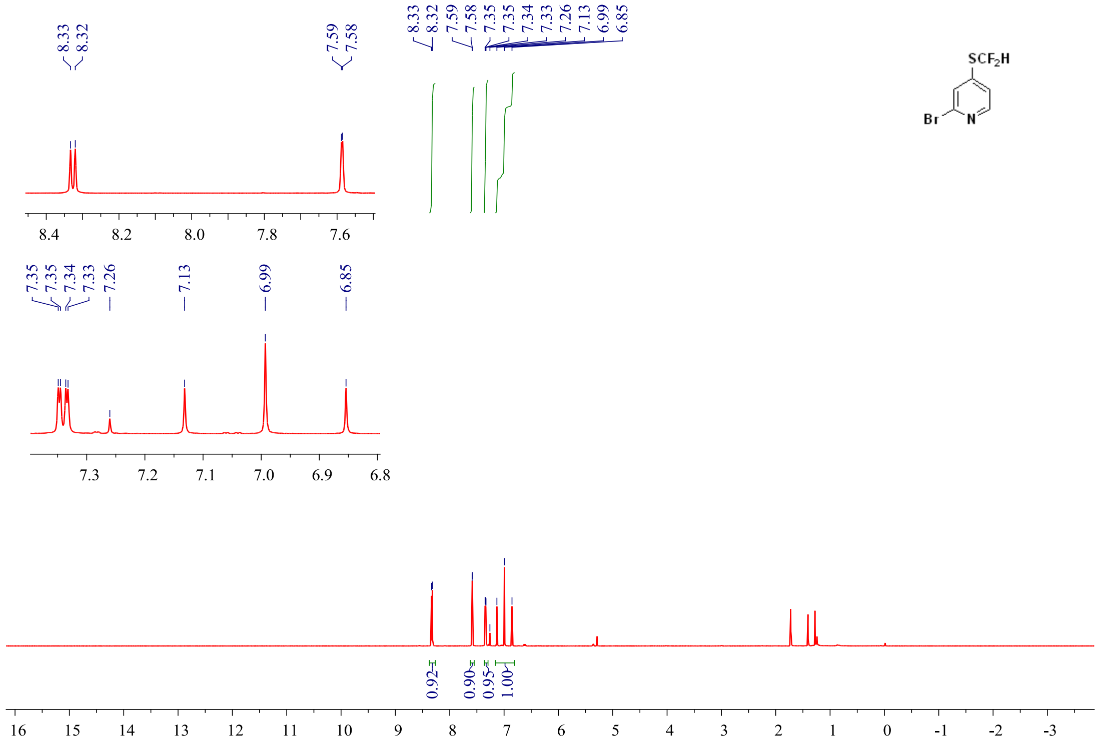

^{19}F NMR (376 MHz, CDCl$_3$) 2-bromo-4-((difluoromethyl)thio)pyridine 4n

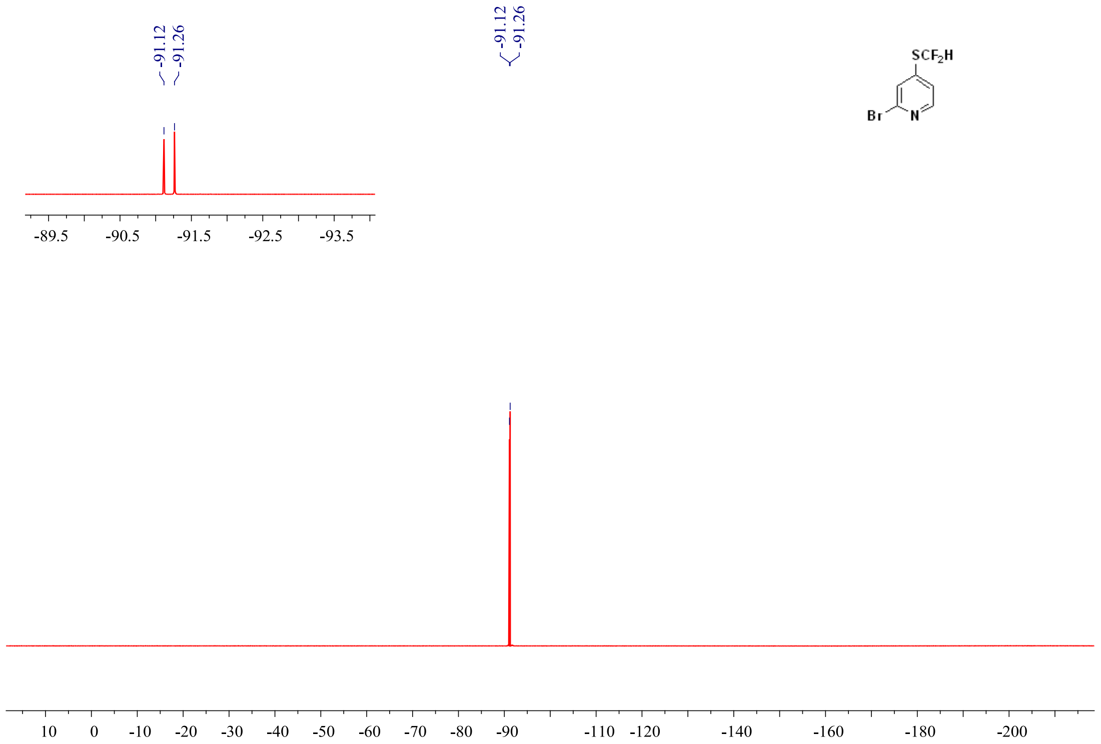

^{13}C NMR (101 MHz, CDCl$_3$) 2-bromo-4-((difluoromethyl)thio)pyridine 4n

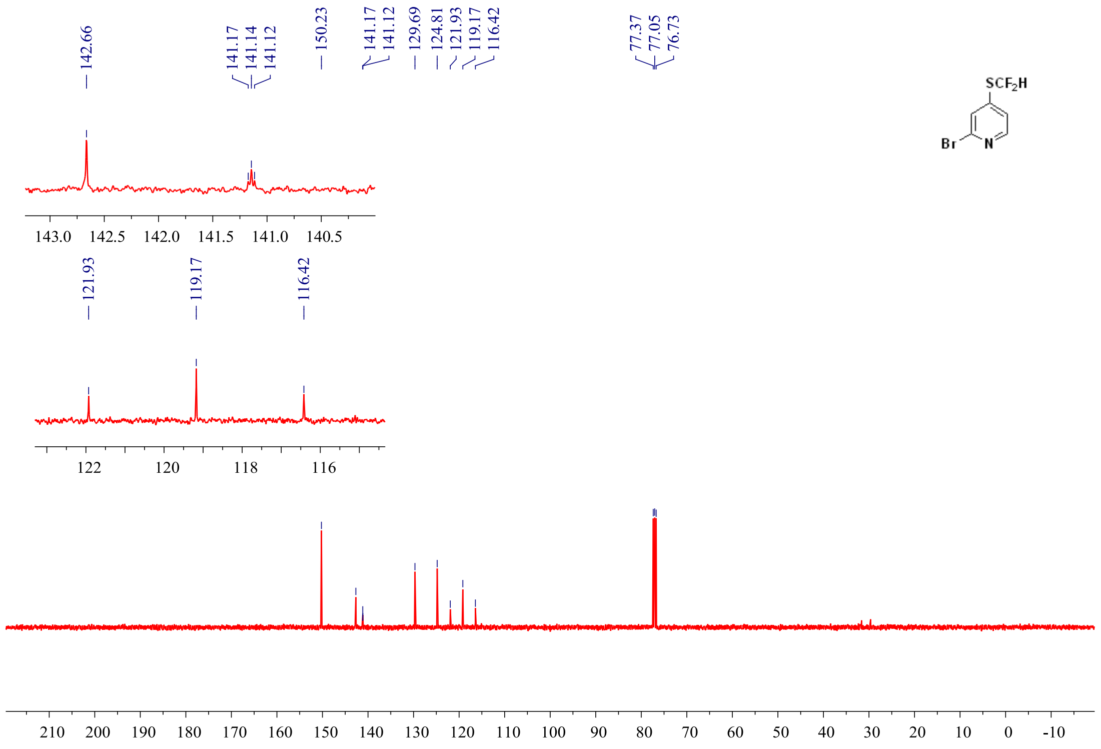

^{1}H NMR (400 MHz, $CDCl_3$) 4-((difluoromethyl)thio)-2-fluoro-5-methylpyridine 4o

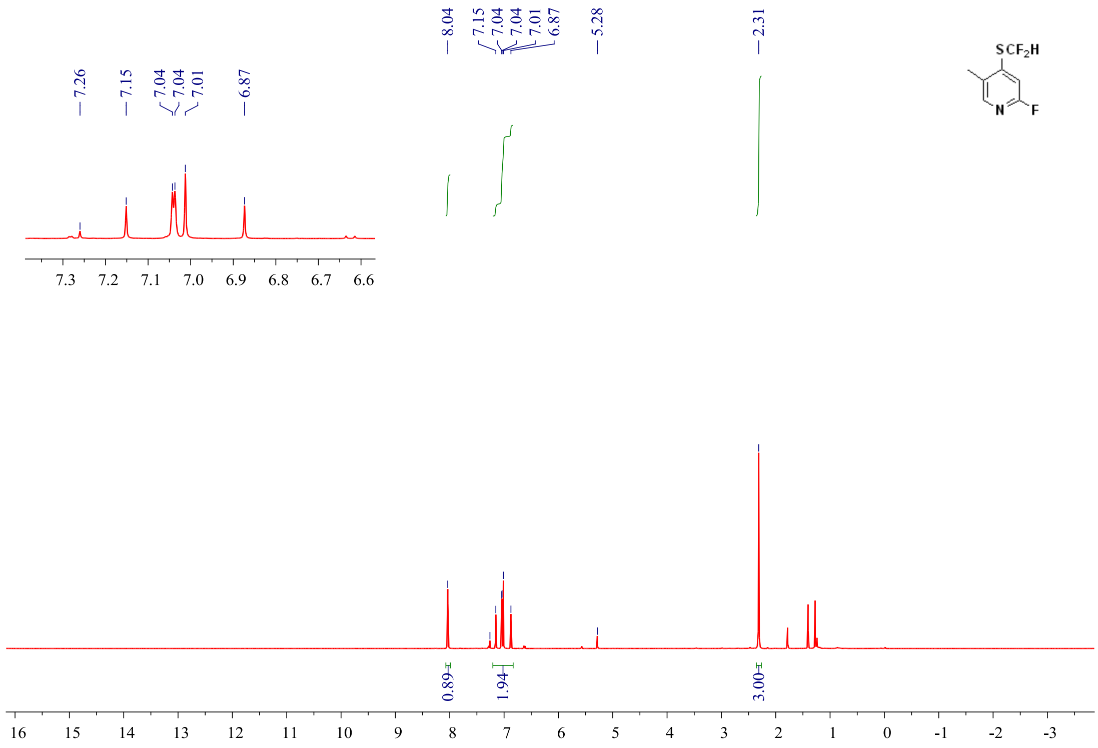

^{19}F NMR (376 MHz, $CDCl_3$) 4-((difluoromethyl)thio)-2-fluoro-5-methylpyridine 4o

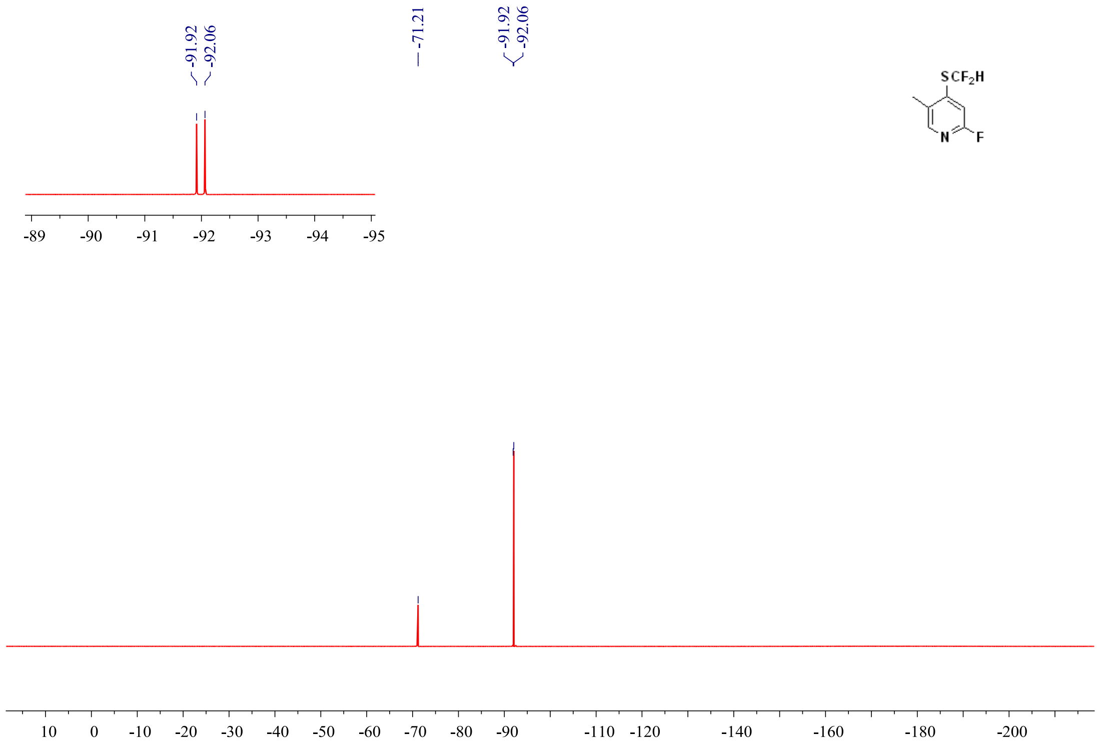

^{13}C NMR (101 MHz, $CDCl_3$) 4-((difluoromethyl)thio)-2-fluoro-5-methylpyridine 4o

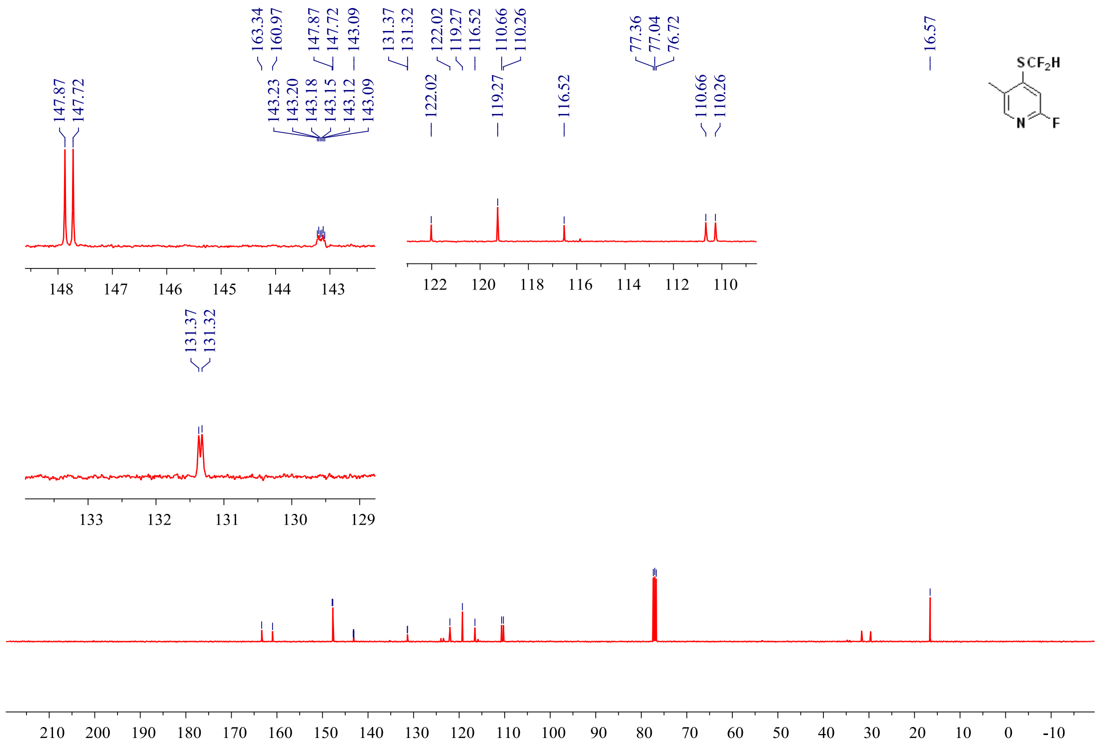

^{1}H NMR (400 MHz, $CDCl_3$) 2-chloro-4-((difluoromethyl)thio)-5- (trifluoromethyl) pyridine 4p

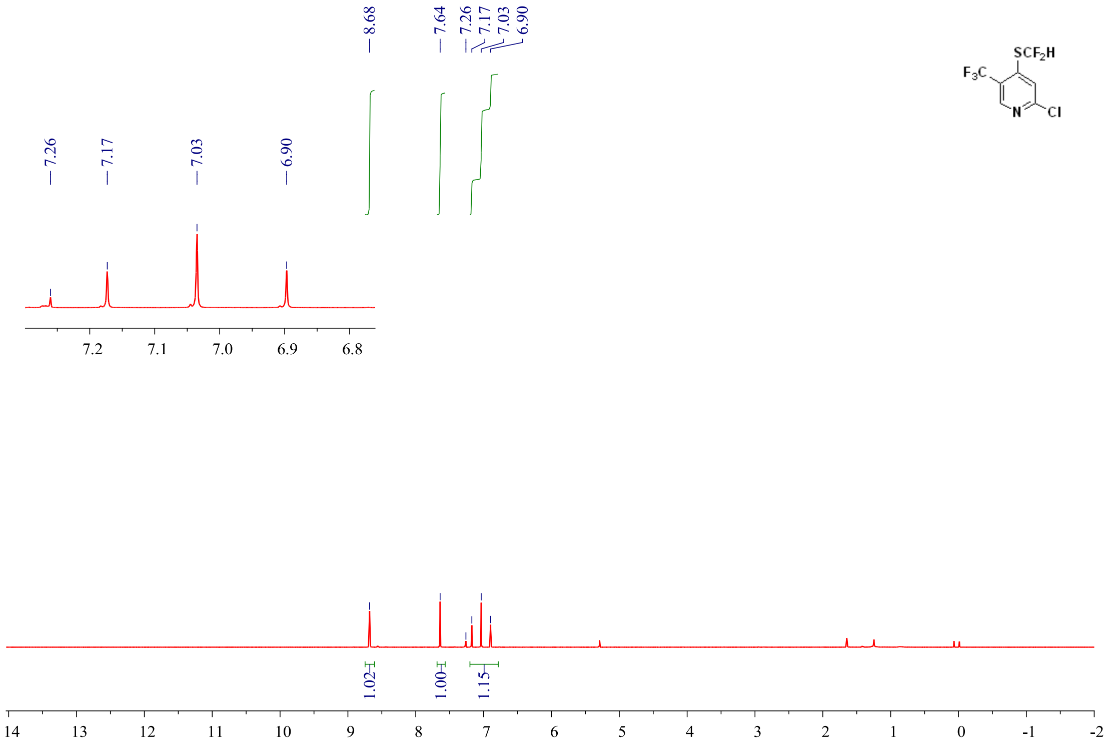

^{19}F NMR (376 MHz, $CDCl_3$) 2-chloro-4-((difluoromethyl)thio)-5-(trifluoromethyl) pyridine 4p

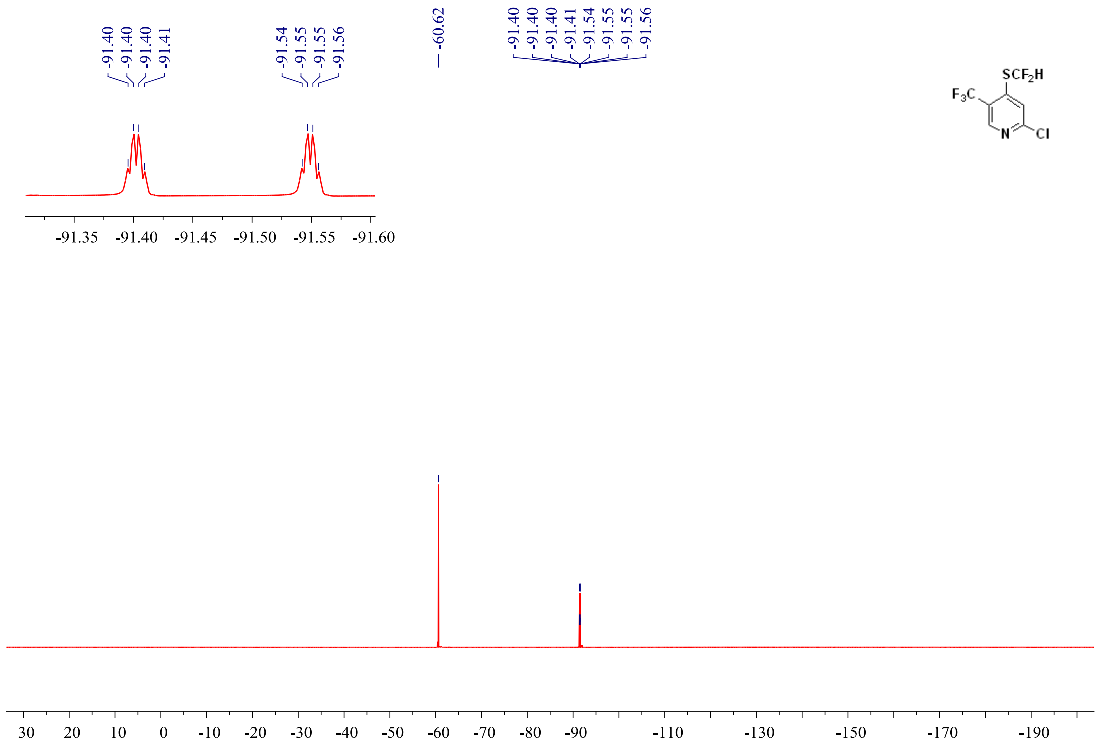

^{13}C NMR (101 MHz, $CDCl_3$) 2-chloro-4-((difluoromethyl)thio)-5-(trifluoromethyl) pyridine 4p

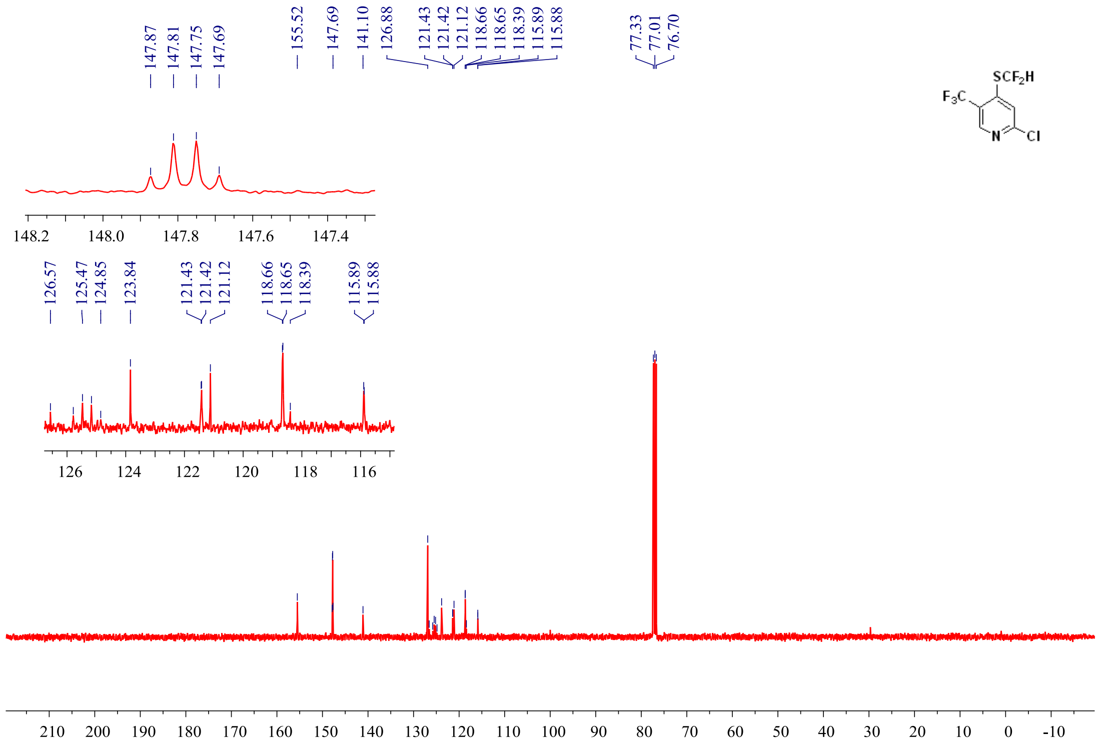

^{1}H NMR (400 MHz, $CDCl_3$) 4-((difluoromethyl)thio)-2-methoxypyridine 4q

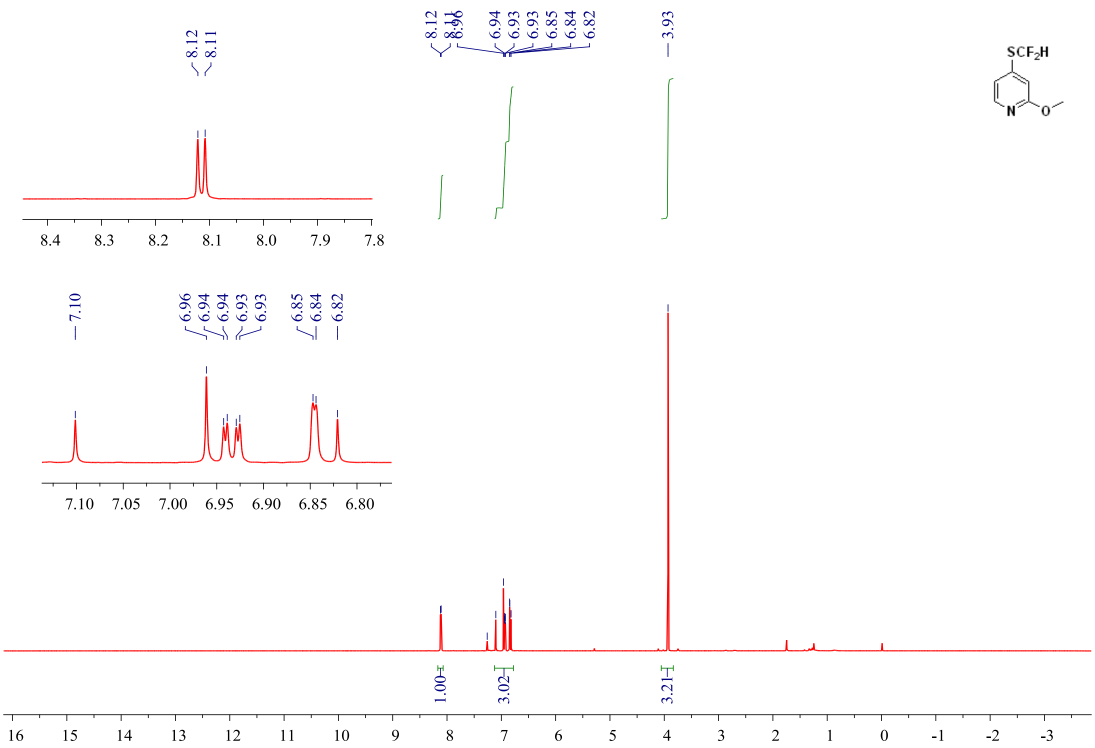

^{19}F NMR (376 MHz, $CDCl_3$) 4-((difluoromethyl)thio)-2-methoxypyridine 4q

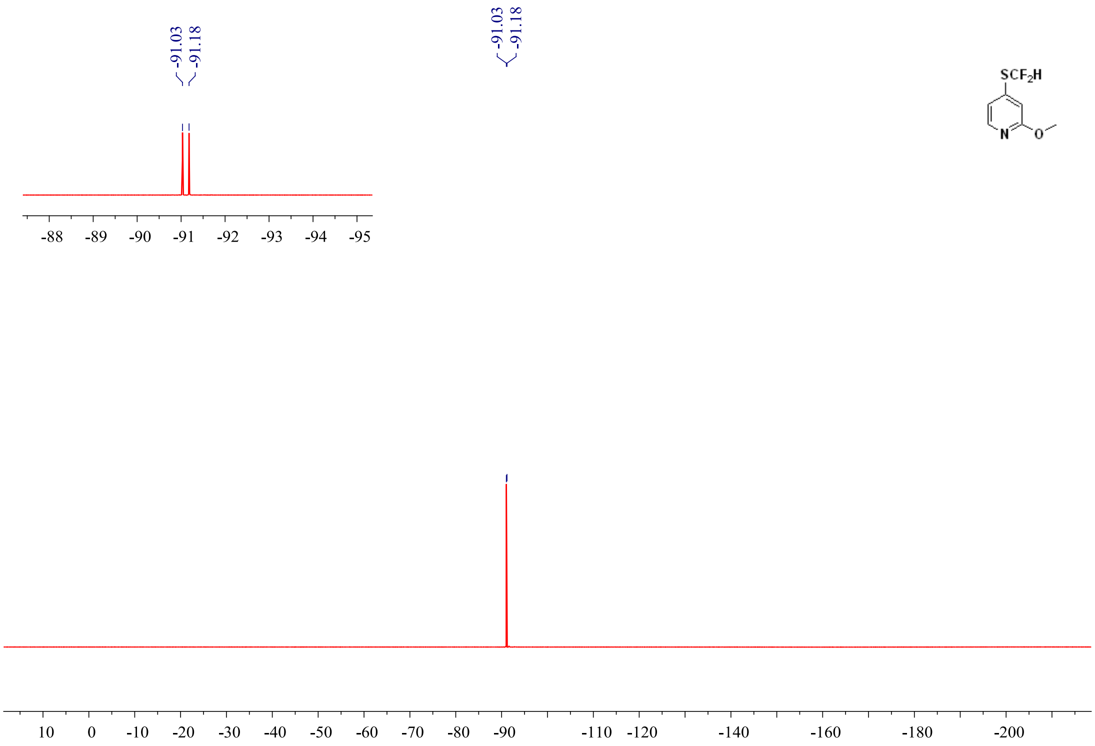

^{13}C NMR (101 MHz, $CDCl_3$) 4-((difluoromethyl)thio)-2-methoxypyridine 4q

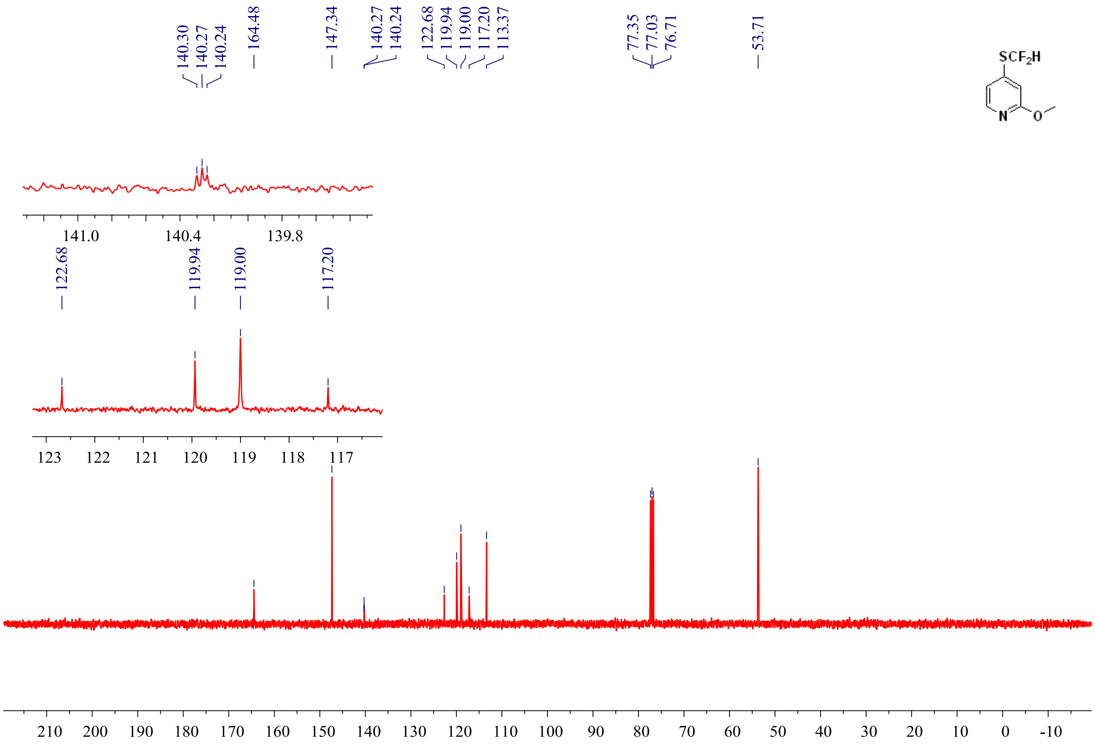

^{1}H NMR (400 MHz, $CDCl_3$) 3-((difluoromethyl)thio)quinolone 4r

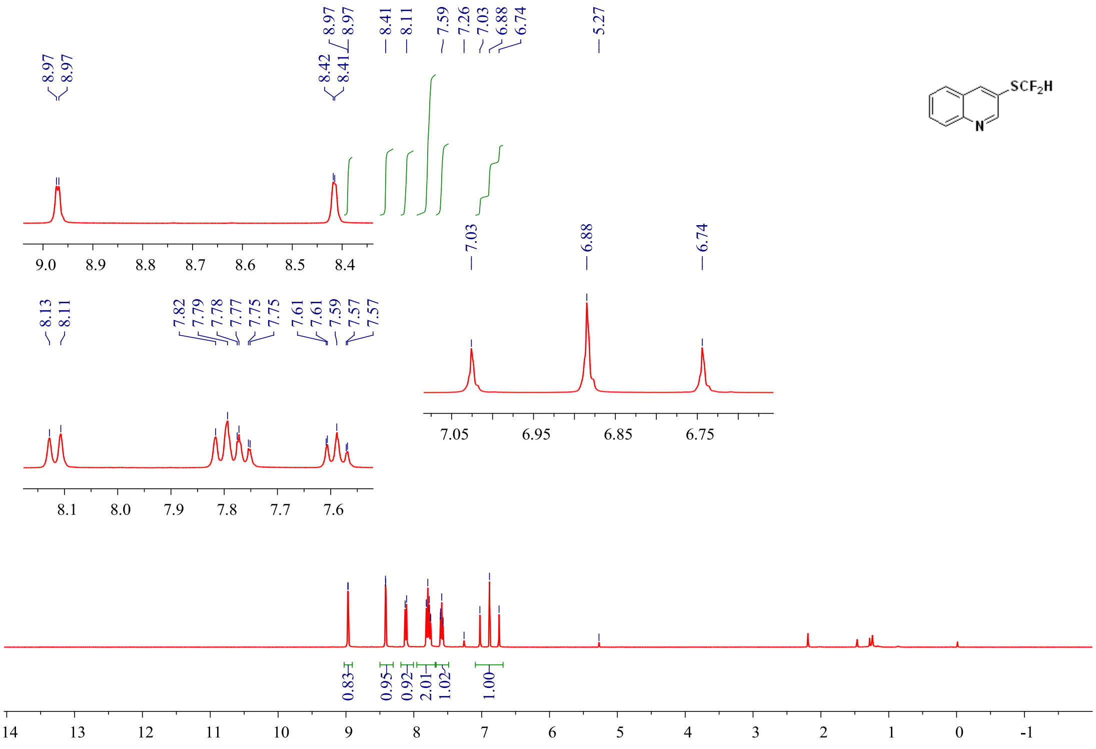

^{19}F NMR (376 MHz, $CDCl_3$) 3-((difluoromethyl)thio)quinolone 4r

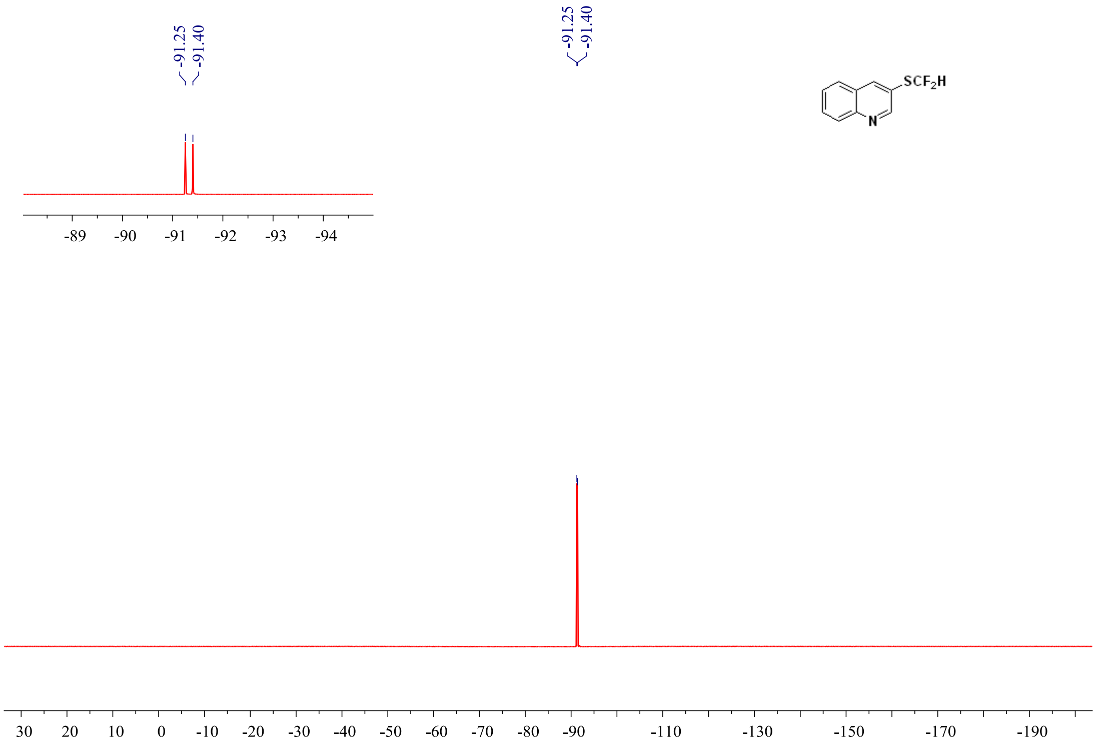

^{13}C NMR (101 MHz, $CDCl_3$) 3-((difluoromethyl)thio)quinolone 4r

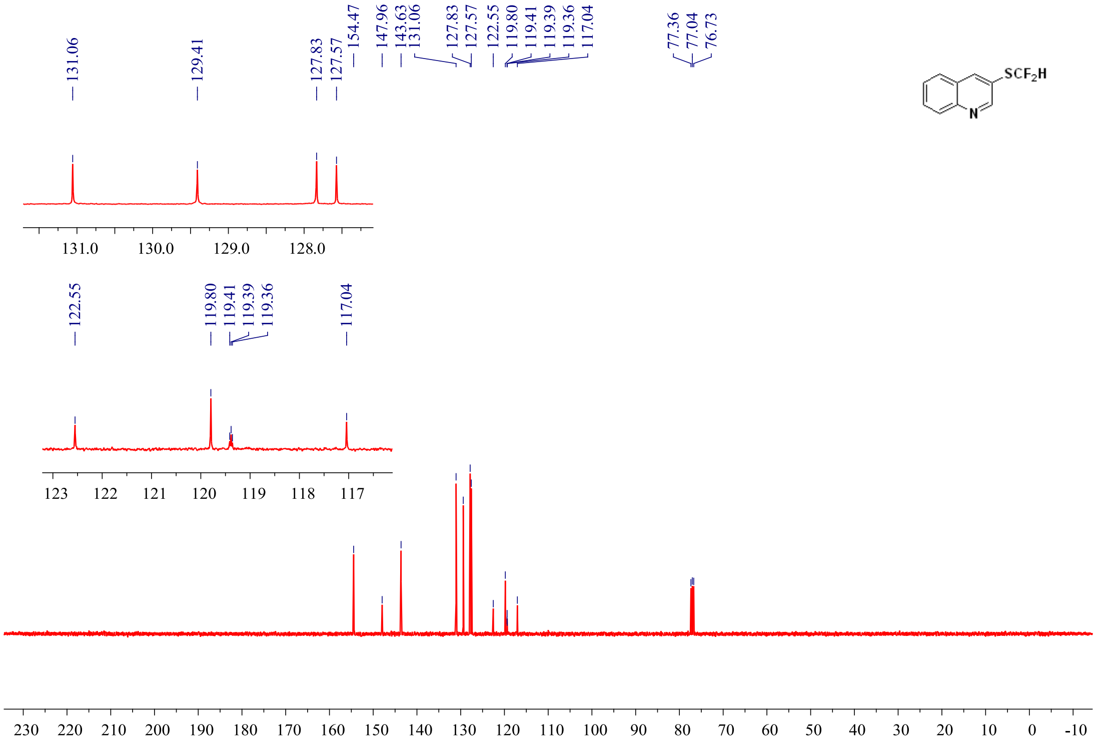

^{1}H NMR (400 MHz, CDCl$_3$) 6-((difluoromethyl)thio)quinolone 4s

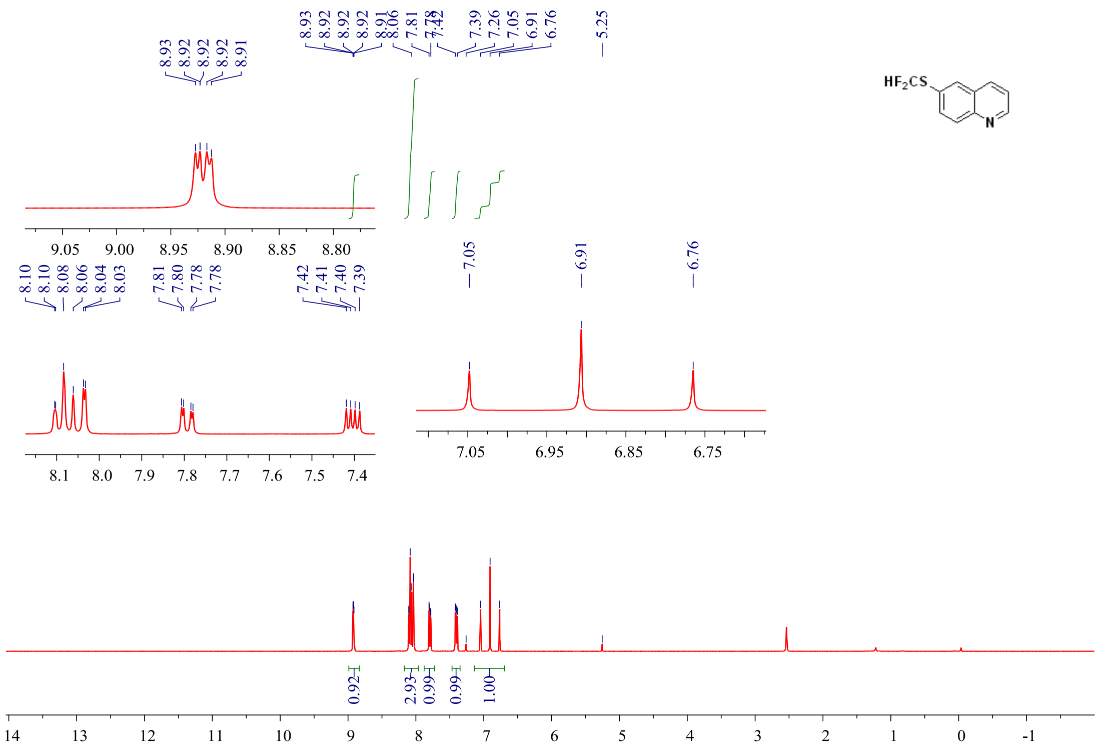

^{19}F NMR (376 MHz, CDCl$_3$) 6-((difluoromethyl)thio)quinolone 4s

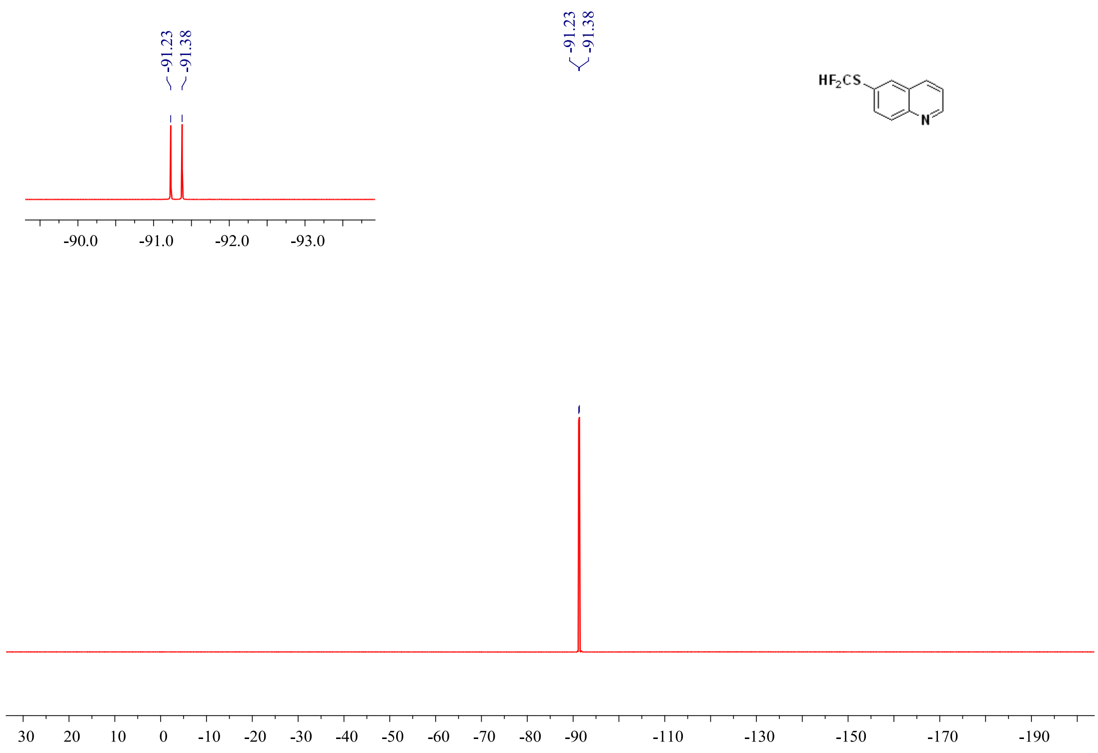

^{13}C NMR (101 MHz, $CDCl_3$) 6-((difluoromethyl)thio)quinolone 4s

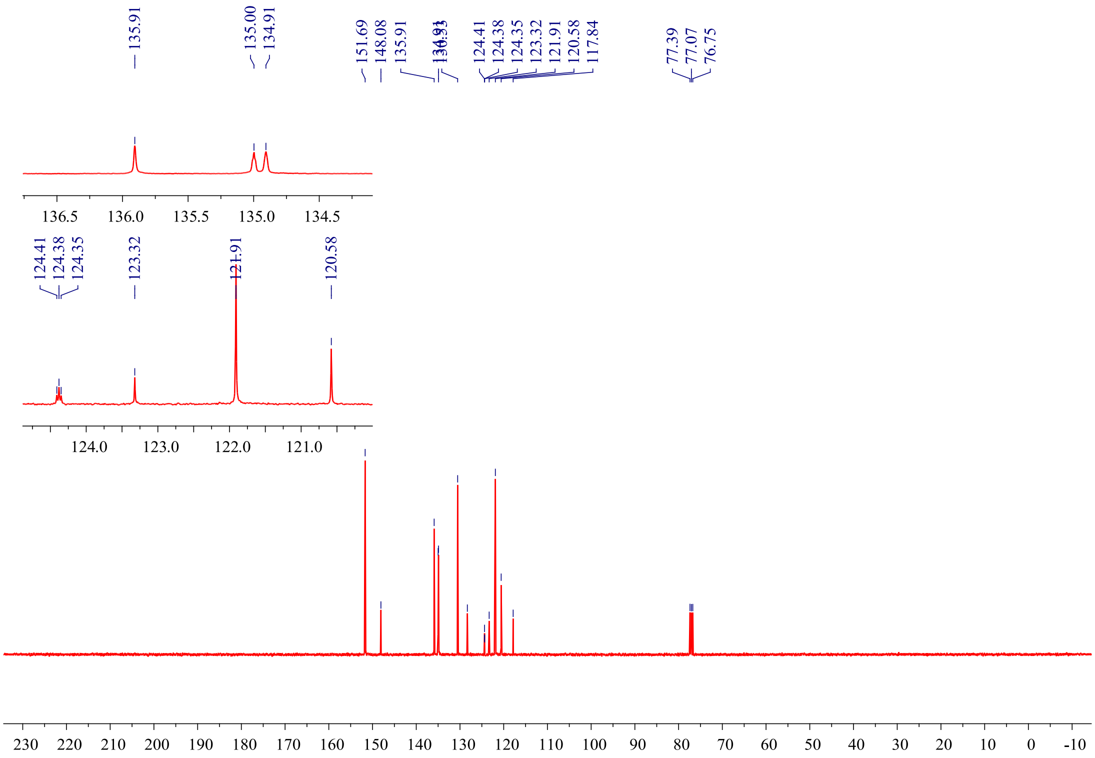

^{1}H NMR (400 MHz, $CDCl_3$) 7-chloro-4-((difluoromethyl)thio)quinolone 4t

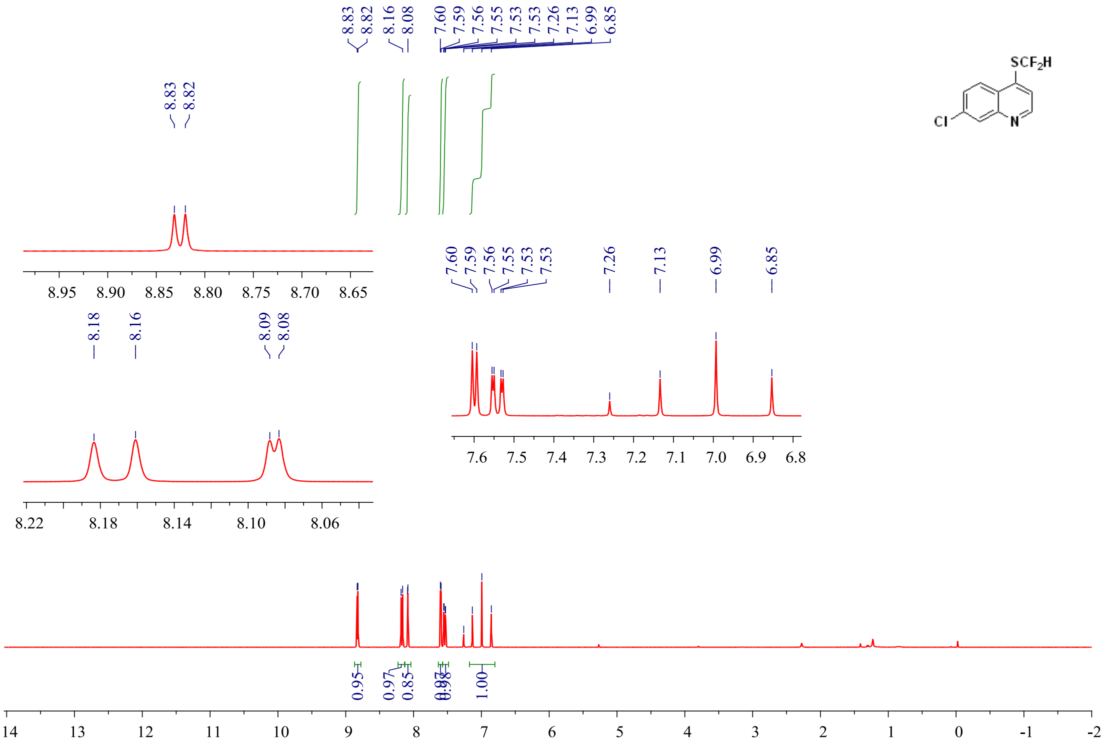

^{19}F NMR (376 MHz, $CDCl_3$) 7-chloro-4-((difluoromethyl)thio)quinolone 4t

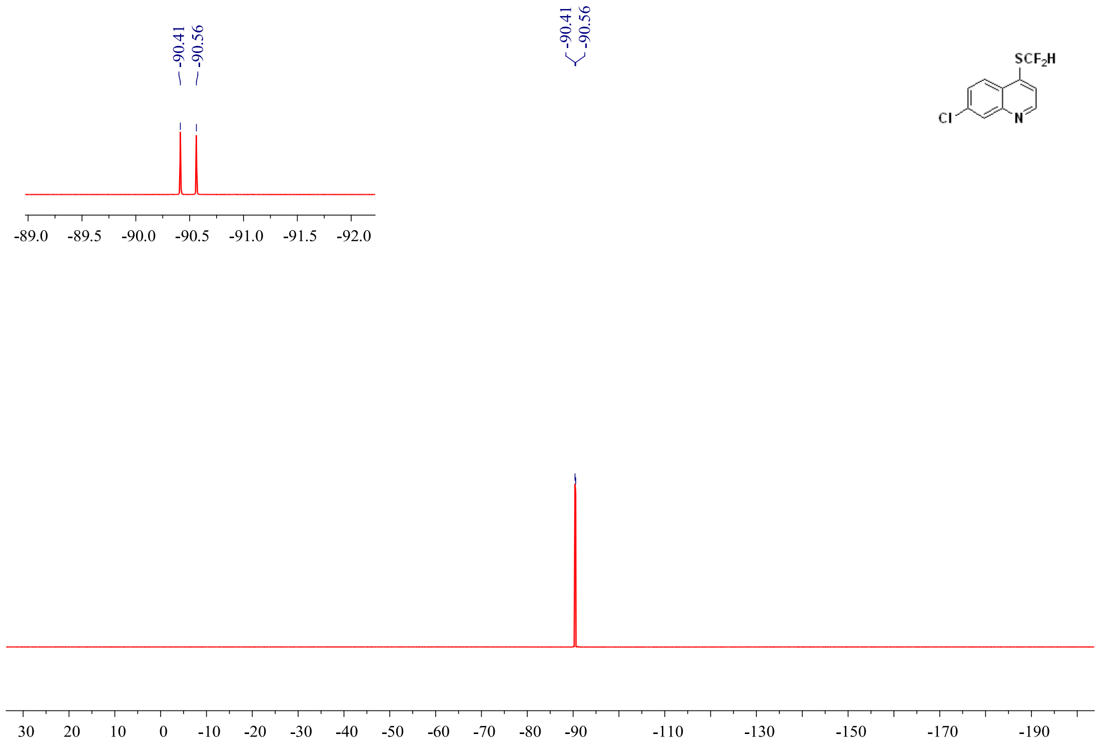

^{13}C NMR (101 MHz, $CDCl_3$) 7-chloro-4-((difluoromethyl)thio)quinolone 4t

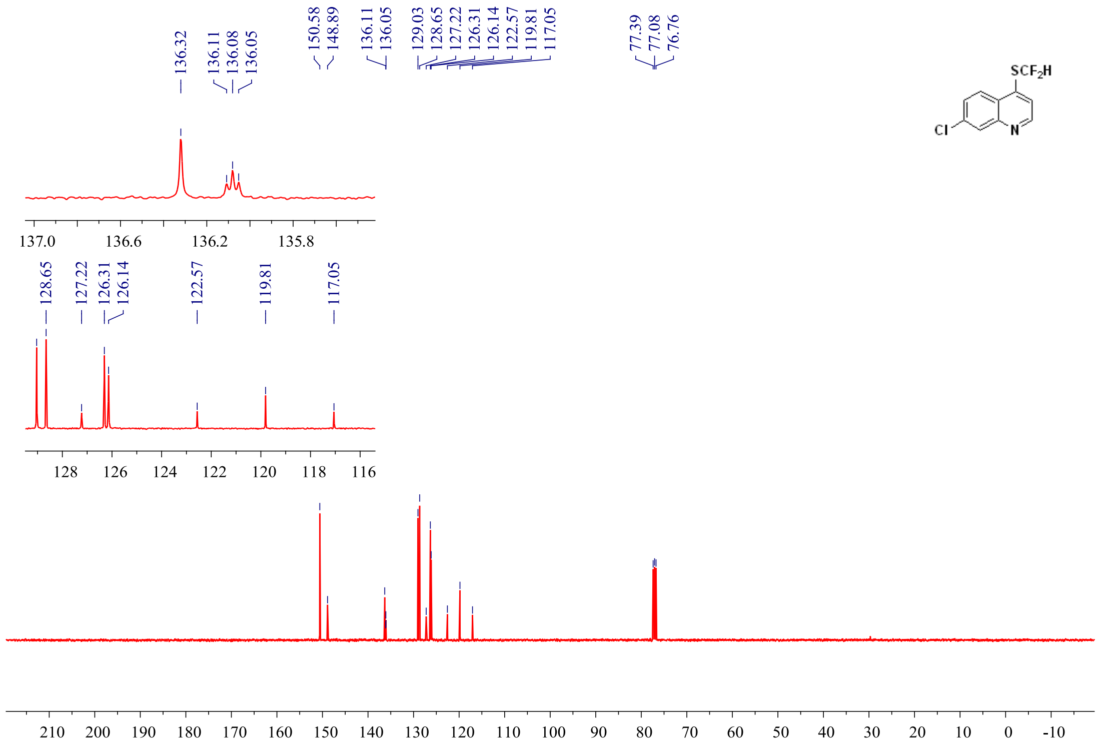

^{1}H NMR (400 MHz, $CDCl_3$) 8-(benzyloxy)-5,7-dichloro-3-((difluoromethyl)thio) quinolone 4u

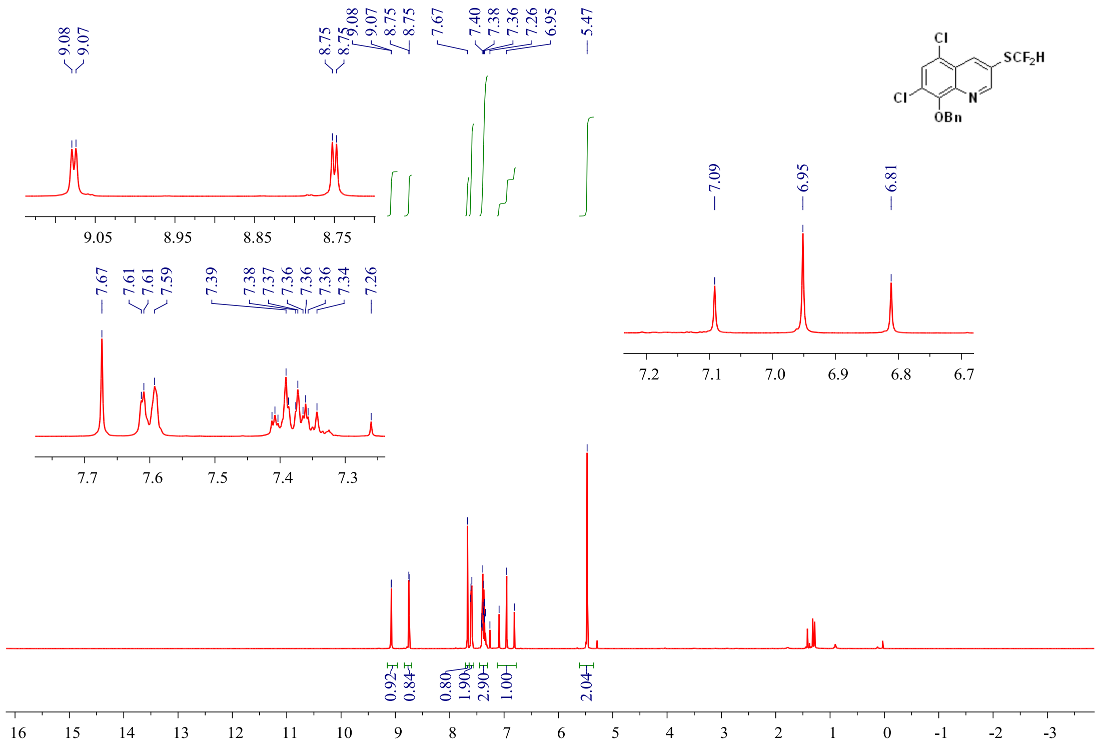

^{19}F NMR (376 MHz, $CDCl_3$) 8-(benzyloxy)-5,7-dichloro-3-((difluoromethyl)thio) quinolone 4u

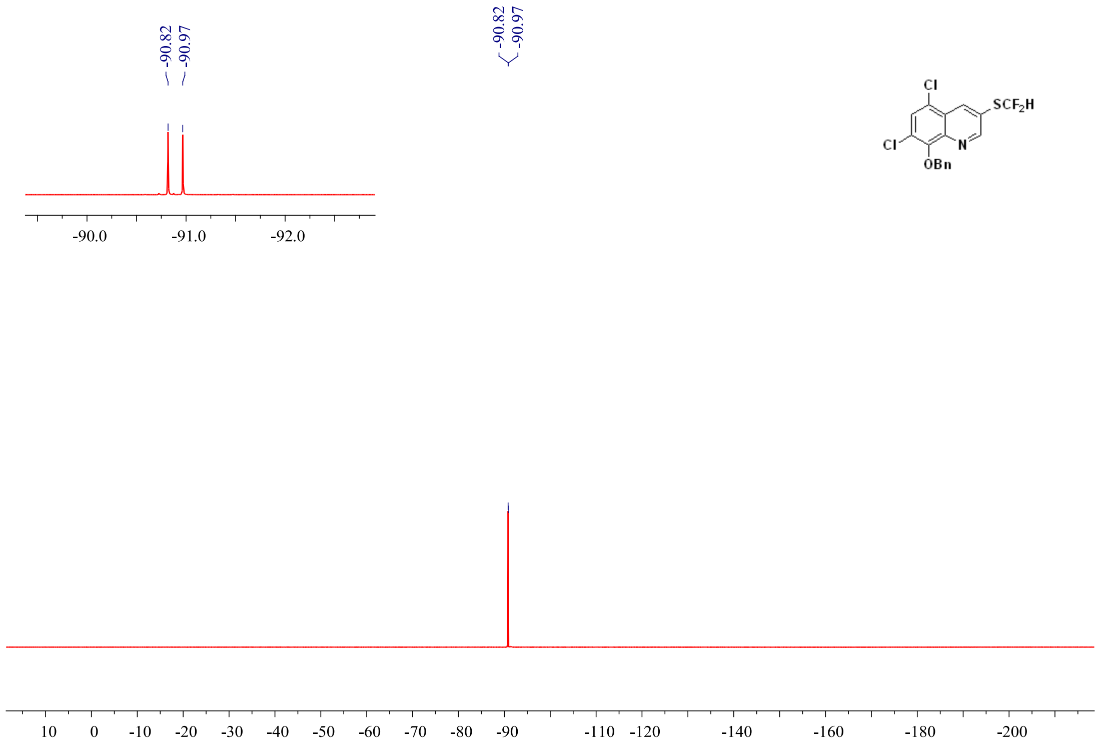

^{13}C NMR (101 MHz, $CDCl_3$) 8-(benzyloxy)-5,7-dichloro-3-((difluoromethyl)thio) quinolone 4u

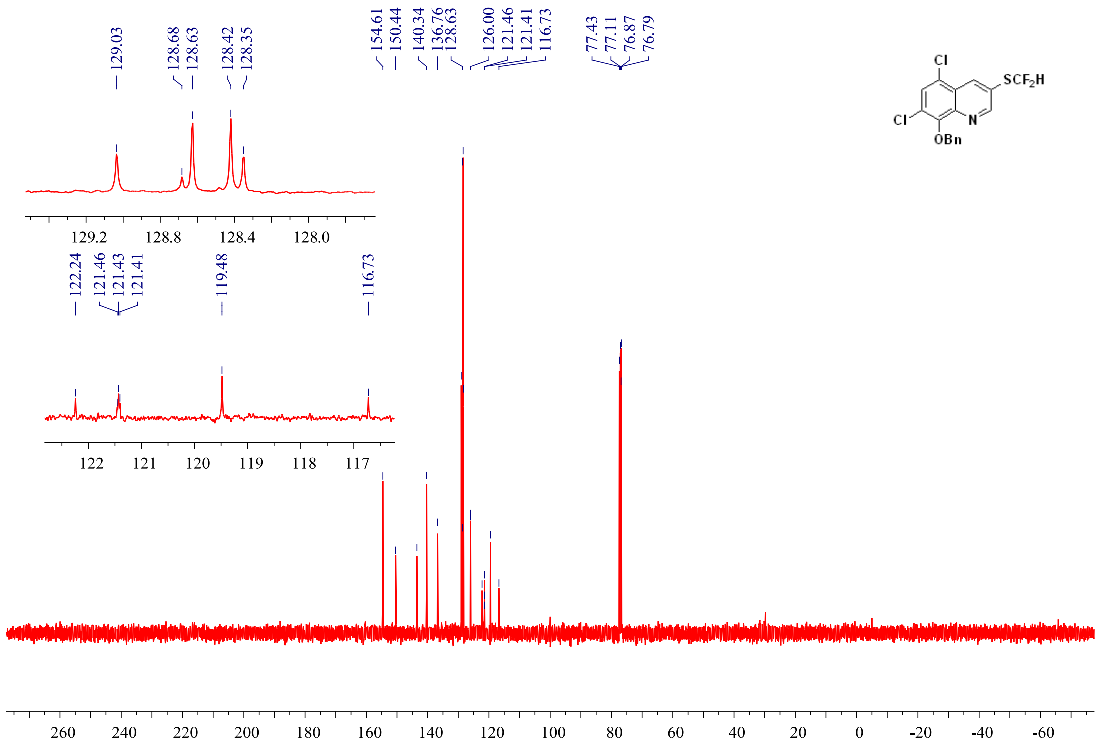

^{1}H NMR (400 MHz, $CDCl_3$) 1-((difluoromethyl)thio)isoquinoline 4v

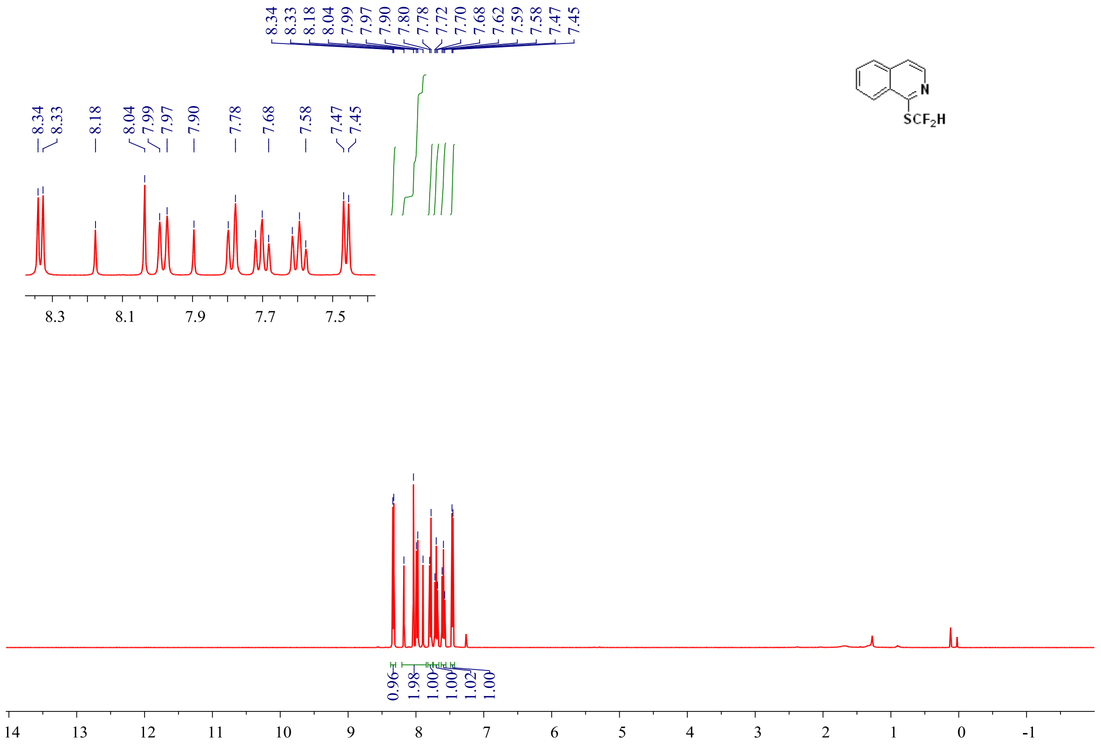

^{19}F NMR (376 MHz, $CDCl_3$) 1-((difluoromethyl)thio)isoquinoline 4v

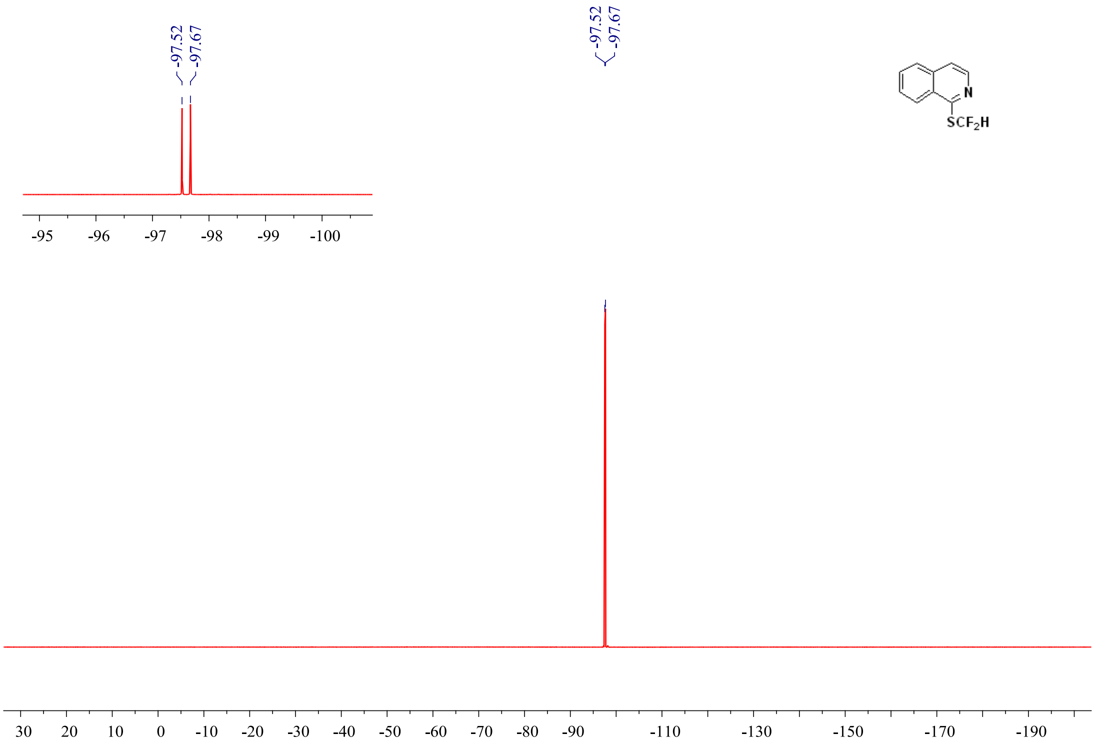

^{13}C NMR (101 MHz, $CDCl_3$) 1-((difluoromethyl)thio)isoquinoline 4v

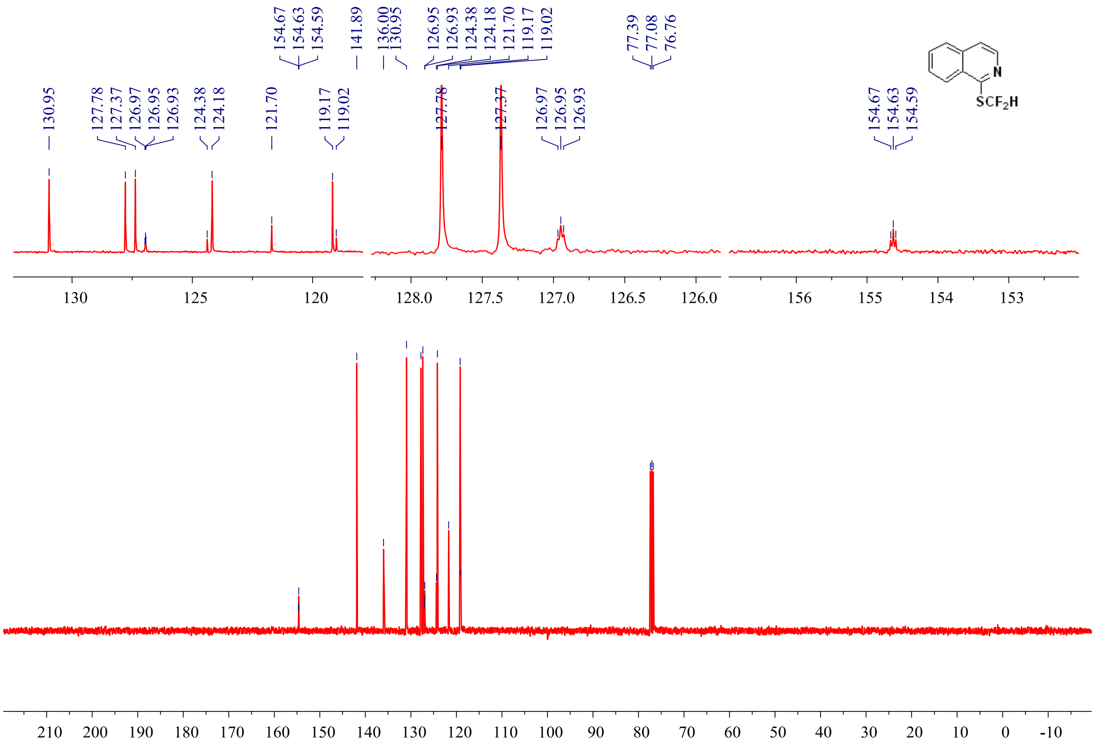

^{1}H NMR (400 MHz, CDCl$_3$) 5-((difluoromethyl)thio)-1-methyl-1*H*-indole 4w

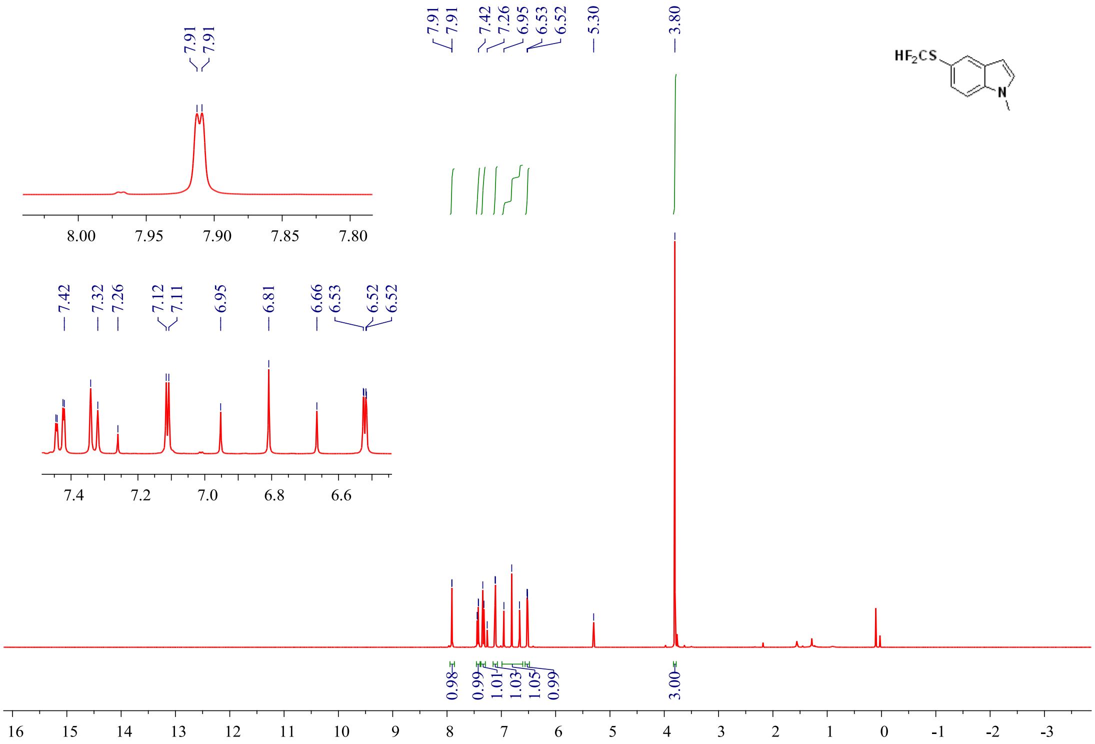

^{19}F NMR (376 MHz, CDCl$_3$) 5-((difluoromethyl)thio)-1-methyl-1*H*-indole 4w

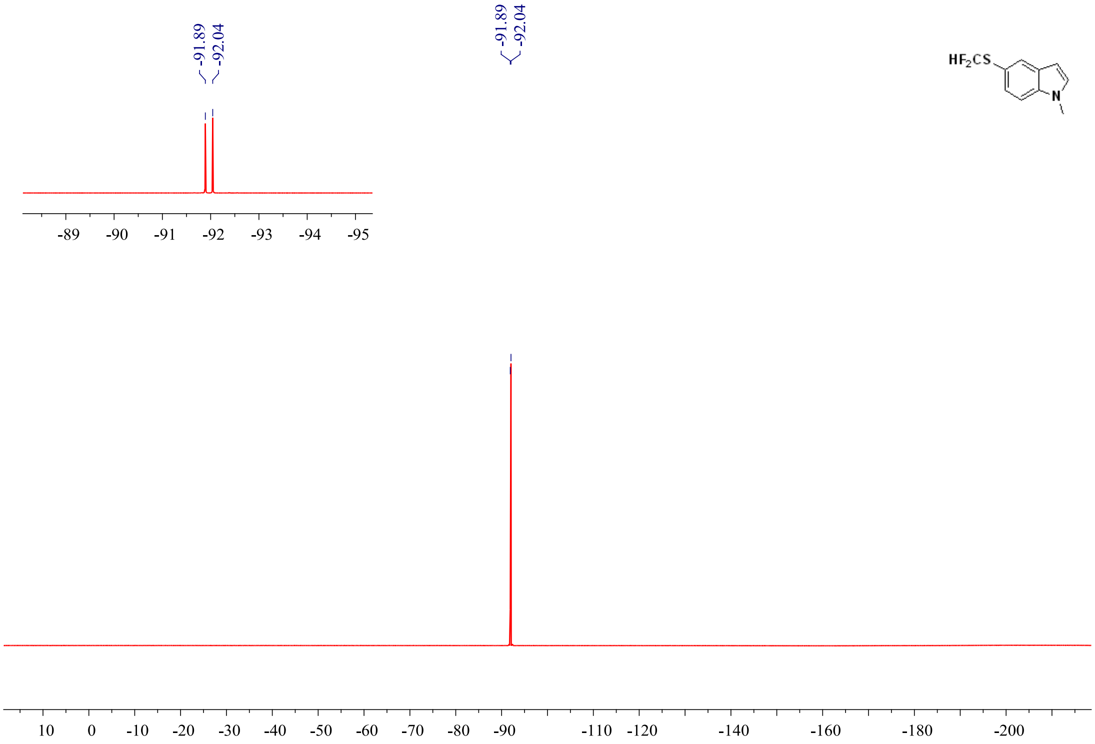

^{13}C NMR (101 MHz, $CDCl_3$) 5-((difluoromethyl)thio)-1-methyl-1*H*-indole 4w

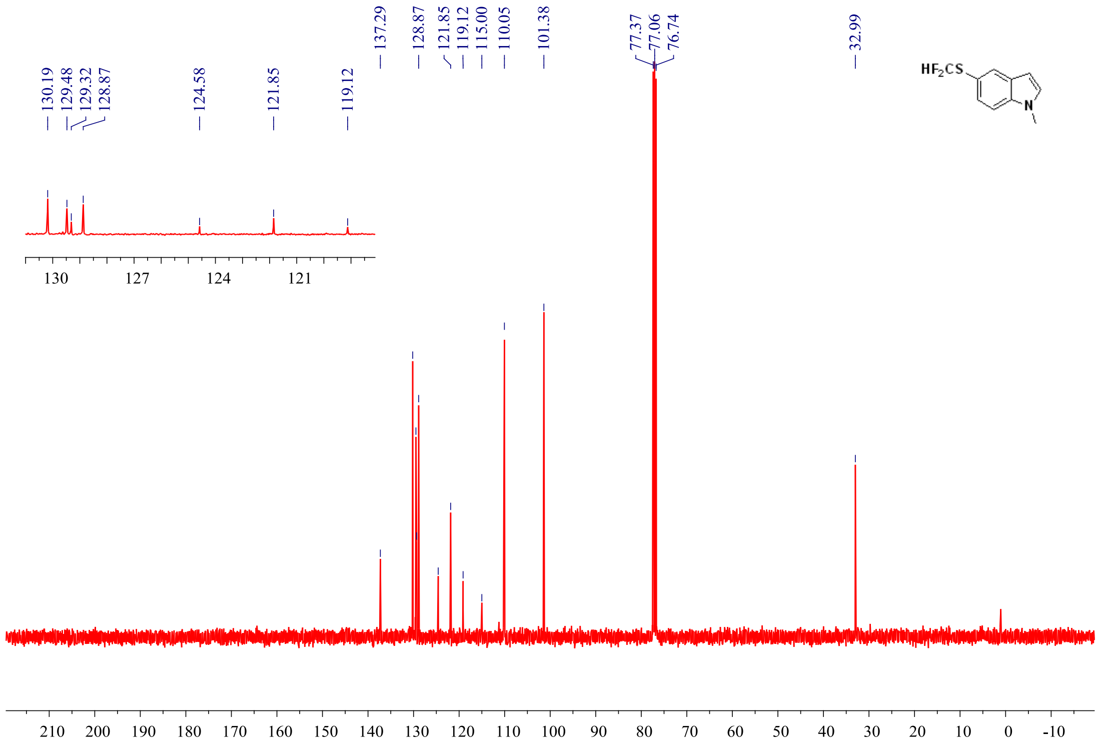

^{1}H NMR (400 MHz, $CDCl_3$) 3-((difluoromethyl)thio)-9-phenyl-9*H*-carbazole 4x

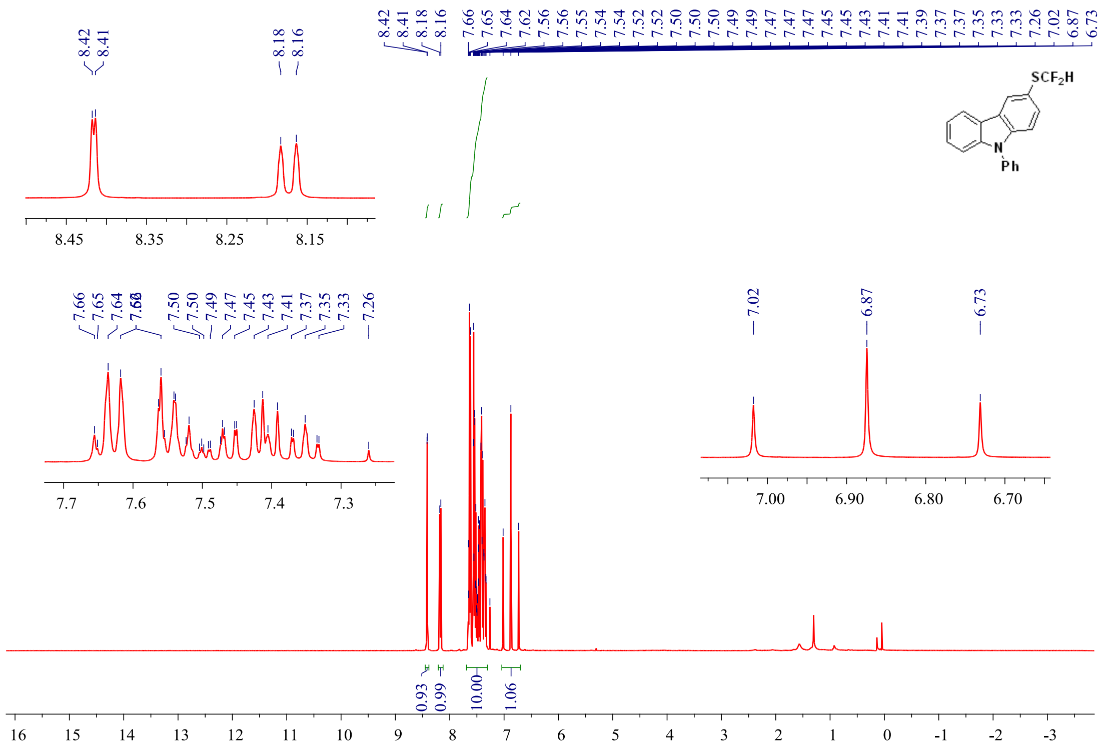

^{19}F NMR (376 MHz, CDCl$_3$) 3-((difluoromethyl)thio)-9-phenyl-9*H*-carbazole 4x

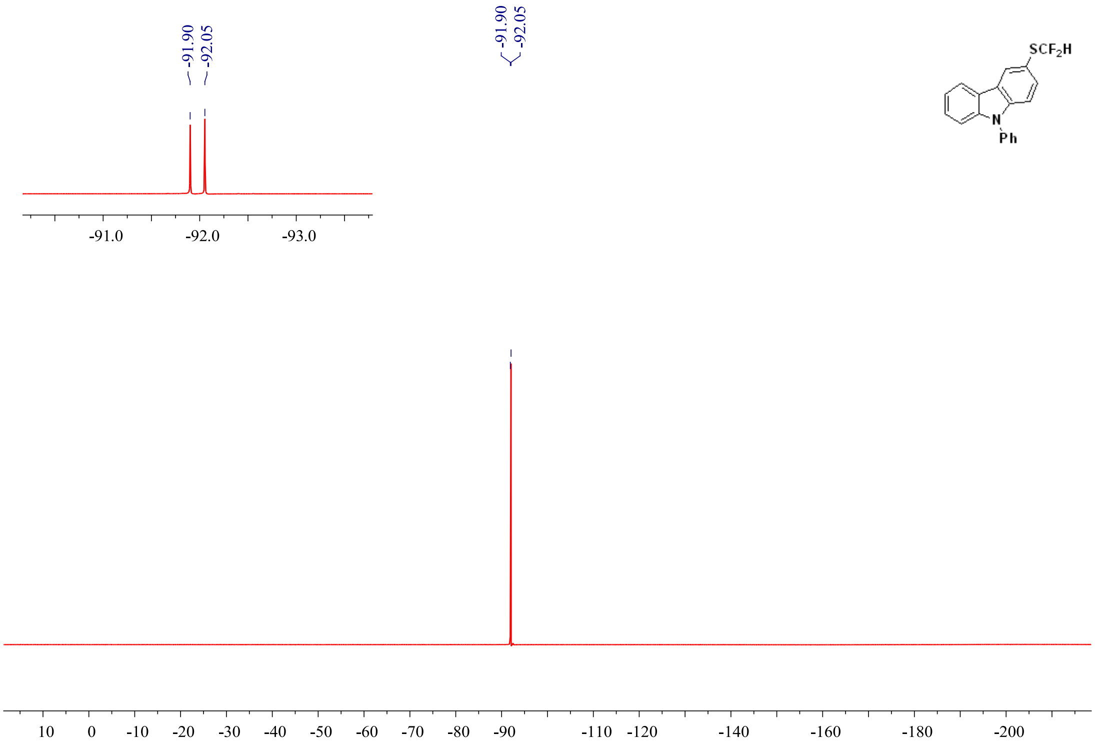

^{13}C NMR (101 MHz, CDCl$_3$) 3-((difluoromethyl)thio)-9-phenyl-9*H*-carbazole 4x

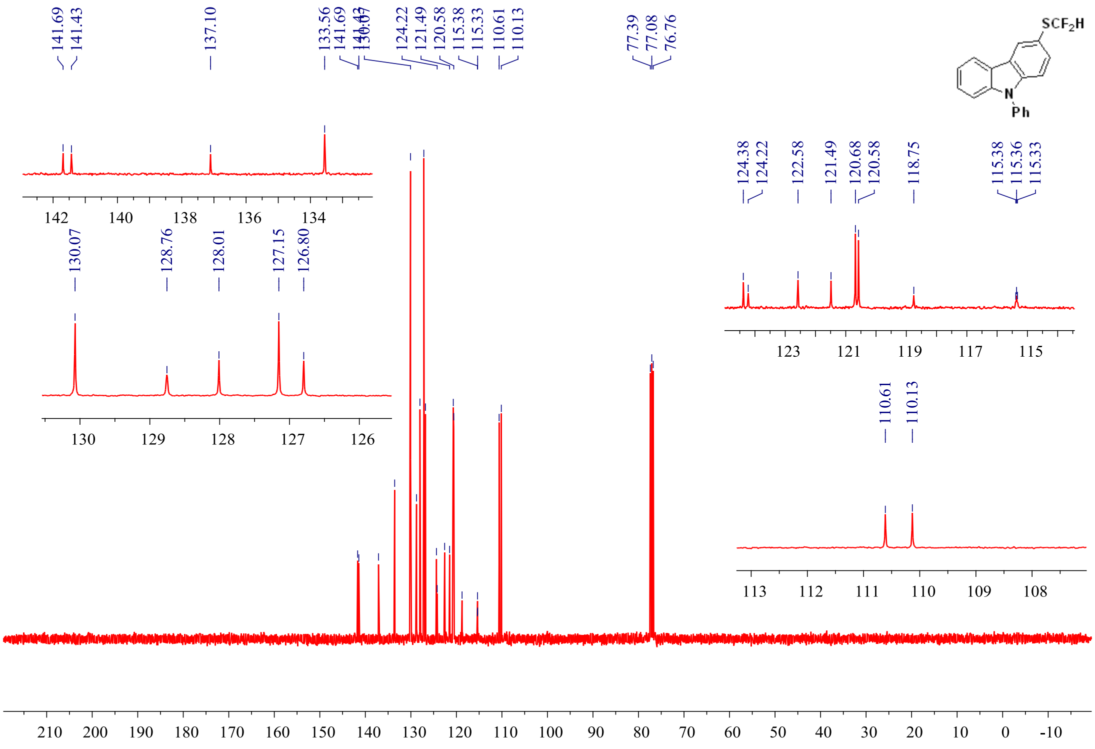

^{1}H NMR (400 MHz, $CDCl_3$) 5-((difluoromethyl)thio)benzofuran 4y

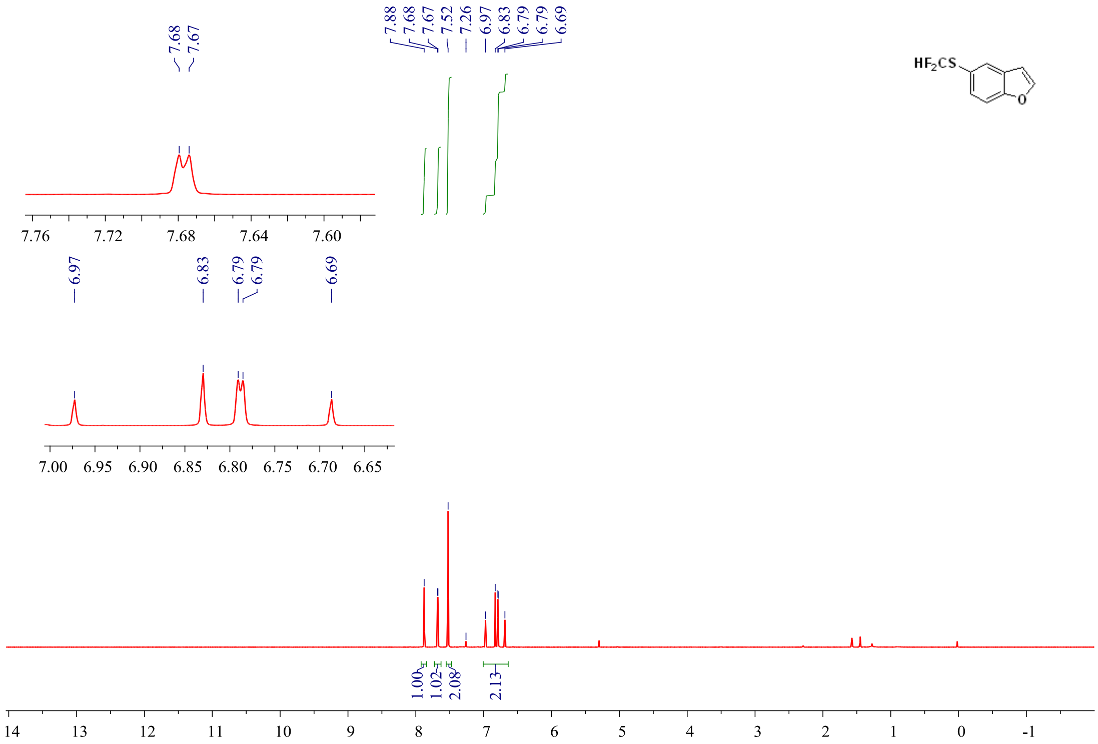

^{19}F NMR (376 MHz, $CDCl_3$) 5-((difluoromethyl)thio)benzofuran 4y

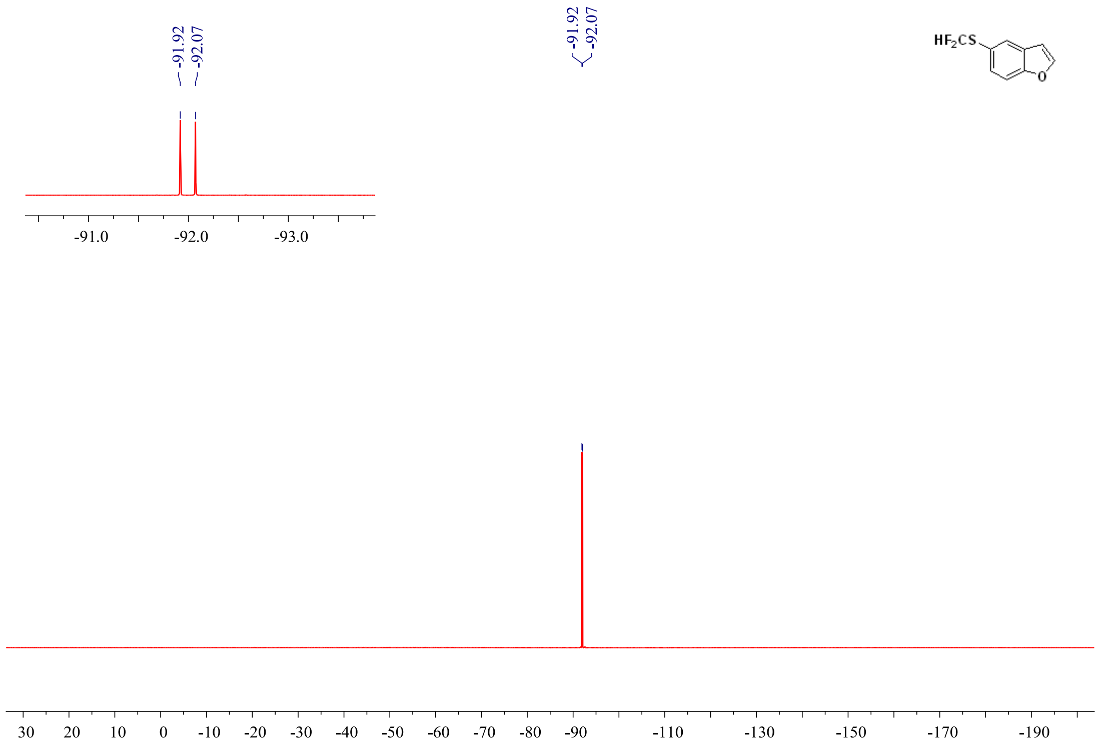

^{13}C NMR (101 MHz, $CDCl_3$) 5-((difluoromethyl)thio)benzofuran 4y

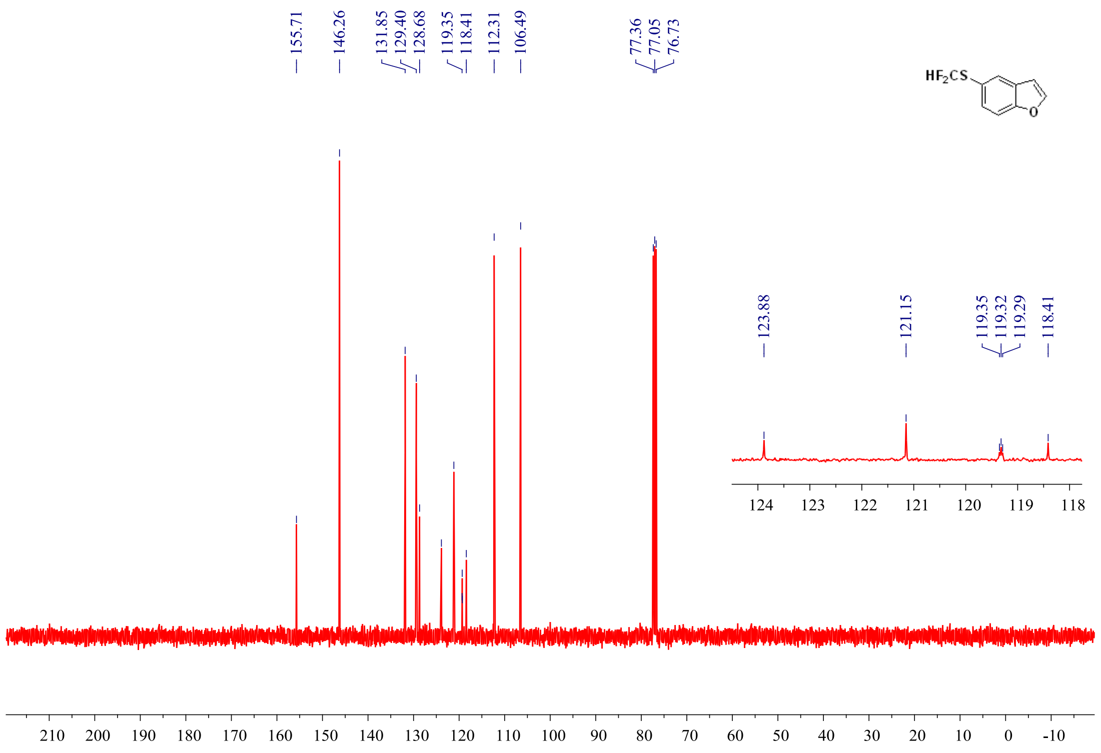

^{1}H NMR (400 MHz, $CDCl_3$) 1-(5-((difluoromethyl)thio) thiophen-2-yl)ethanone 4z

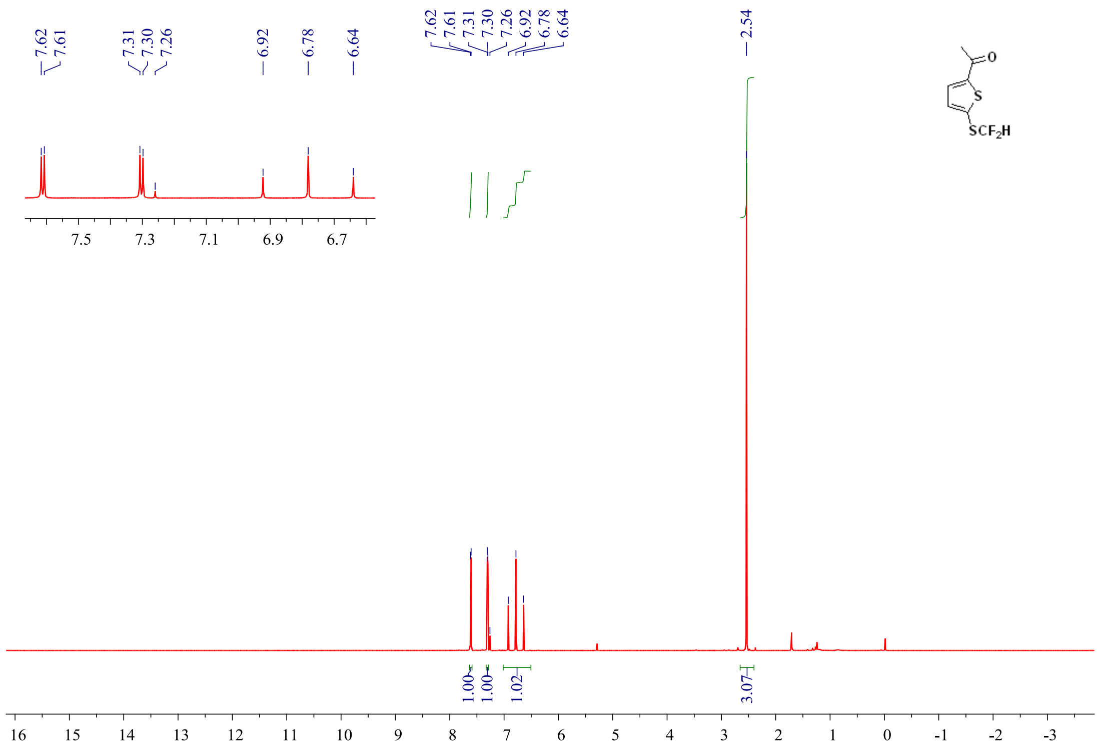

^{19}F NMR (376 MHz, $CDCl_3$) 1-(5-((difluoromethyl)thio) thiophen-2-yl)ethanone 4z

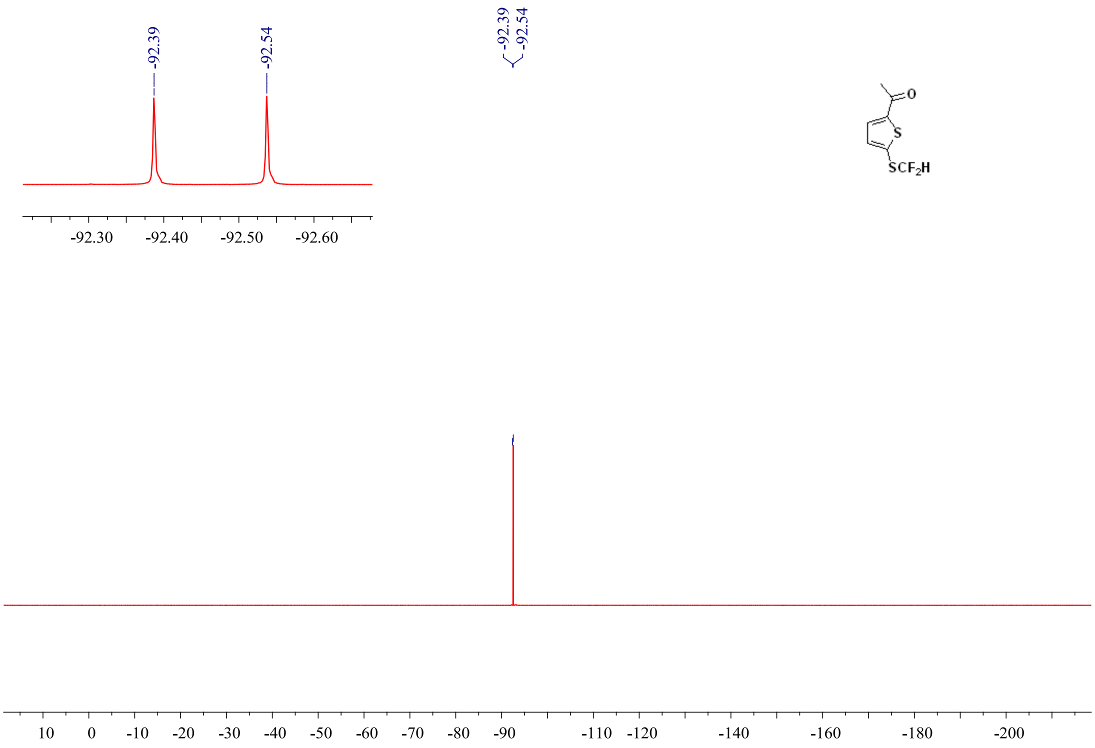

^{13}C NMR (101 MHz, $CDCl_3$) 1-(5-((difluoromethyl)thio) thiophen-2-yl)ethanone 4z

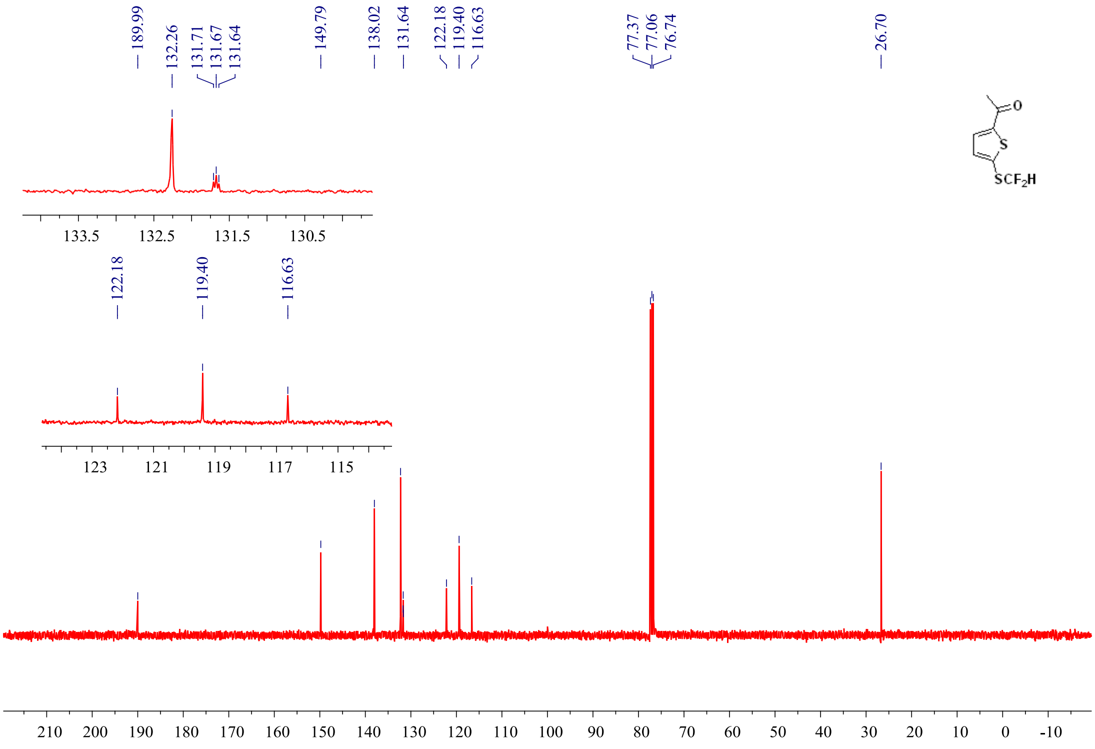

^{1}H NMR (400 MHz, $CDCl_3$) 4-((difluoromethyl)thio)dibenzo[b,d]thiophene 4aa

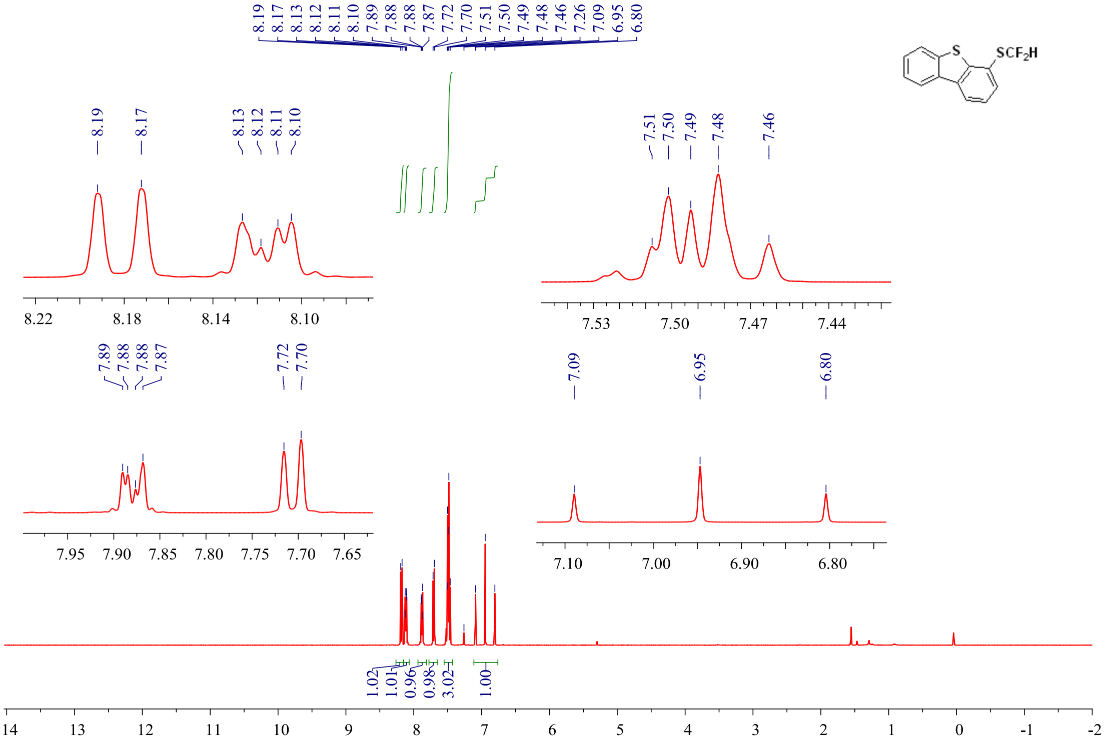

^{19}F NMR (376 MHz, $CDCl_3$) 4-((difluoromethyl)thio)dibenzo[b,d]thiophene 4aa

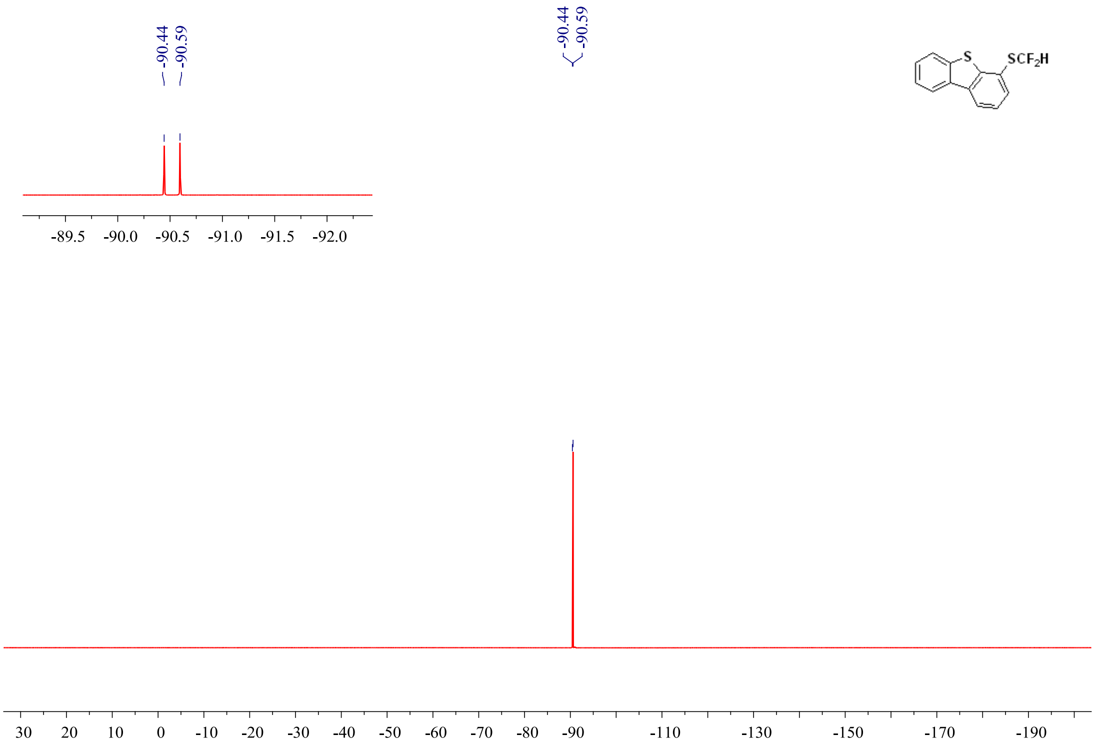

^{13}C NMR (101 MHz, $CDCl_3$) 4-((difluoromethyl)thio)dibenzo[b,d]thiophene 4aa

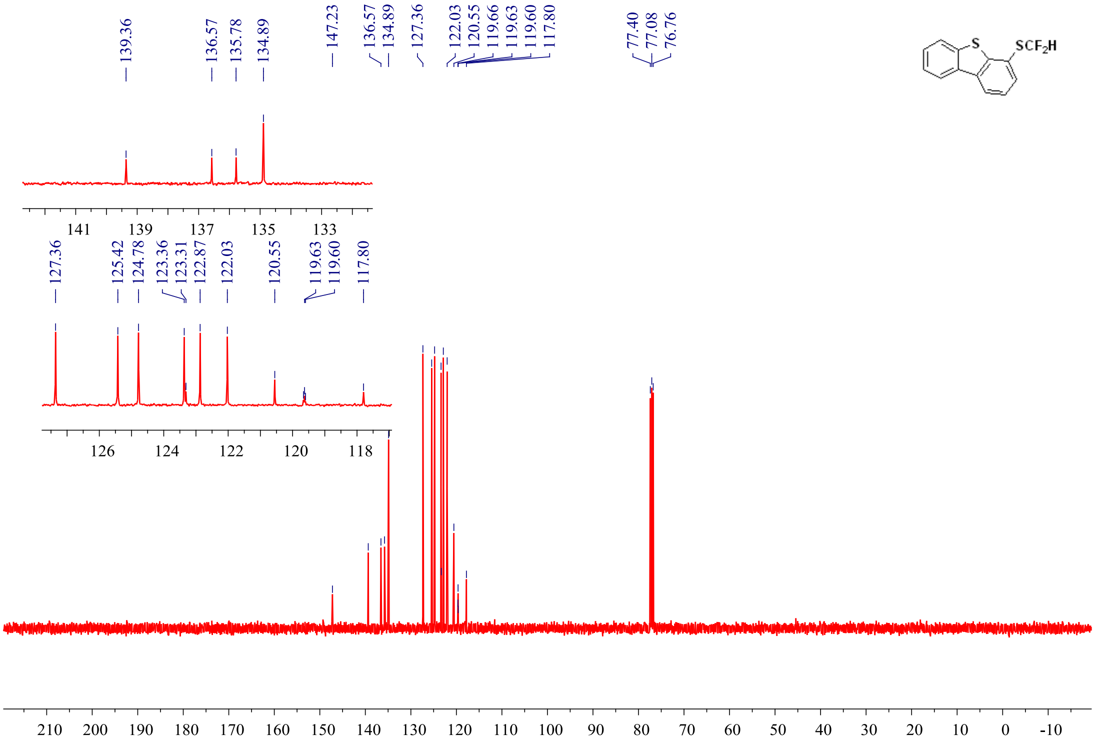

^{1}H NMR (400 MHz, $CDCl_3$) 2-chloro-5-((difluoromethyl)thio)pyrimidine 4ab

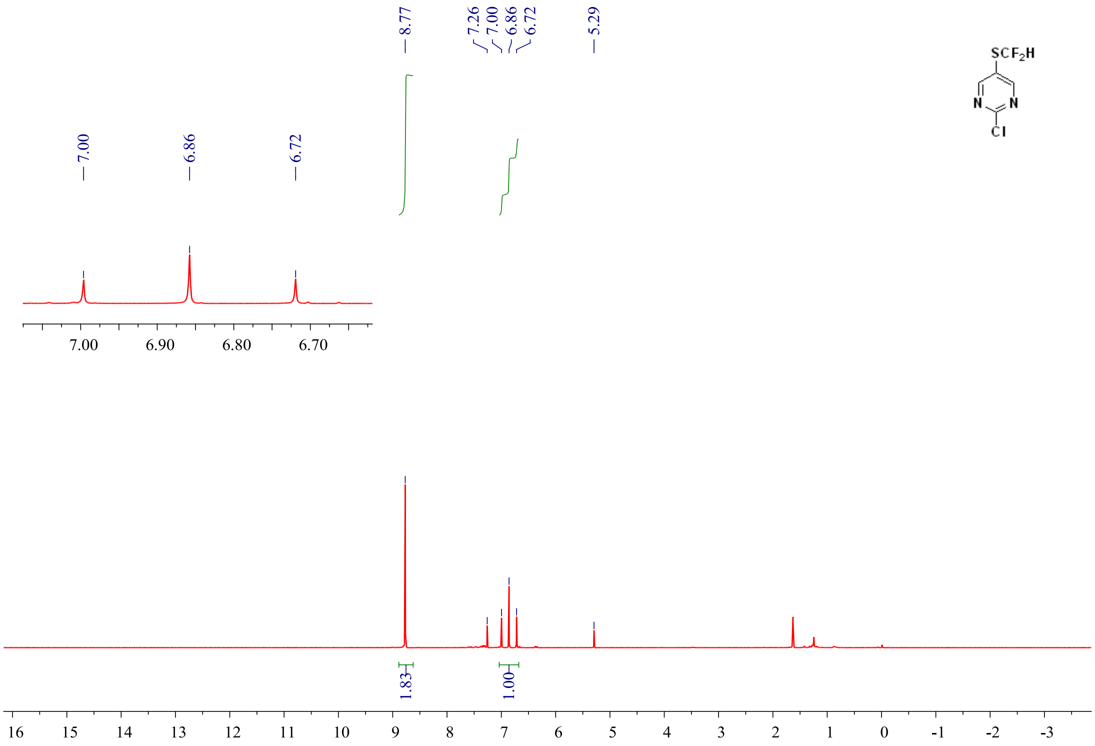

^{19}F NMR (376 MHz, $CDCl_3$) 2-chloro-5-((difluoromethyl)thio)pyrimidine 4ab

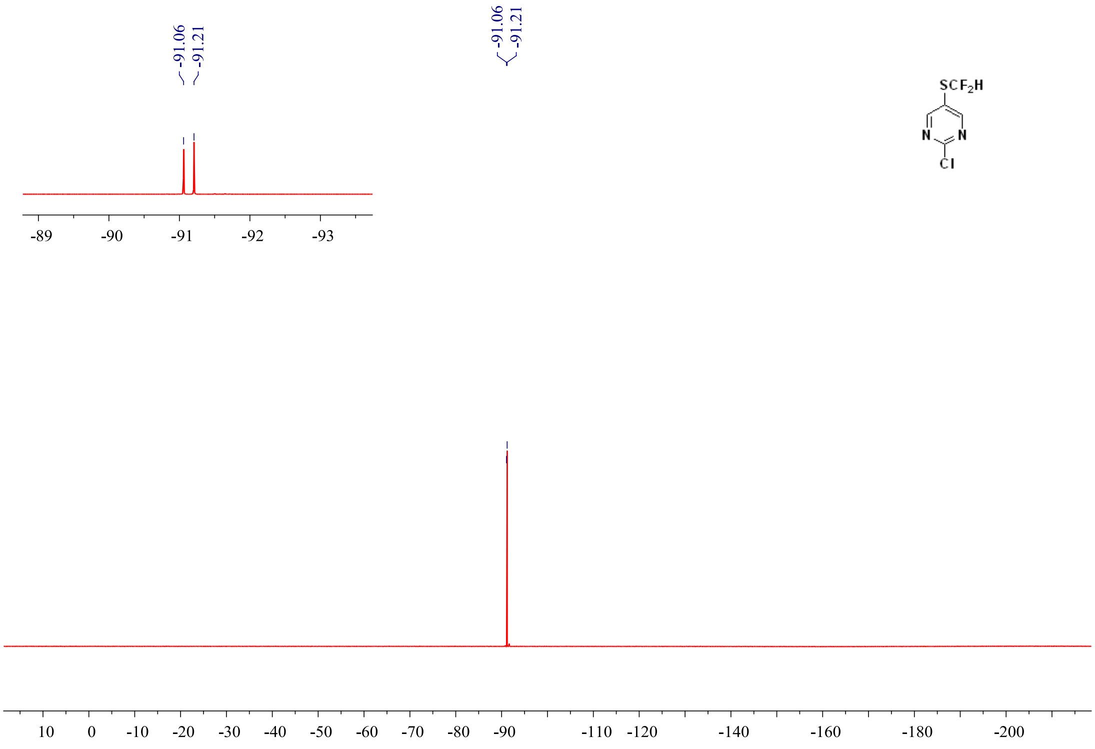

^{13}C NMR (101 MHz, $CDCl_3$) 2-chloro-5-((difluoromethyl)thio)pyrimidine 4ab

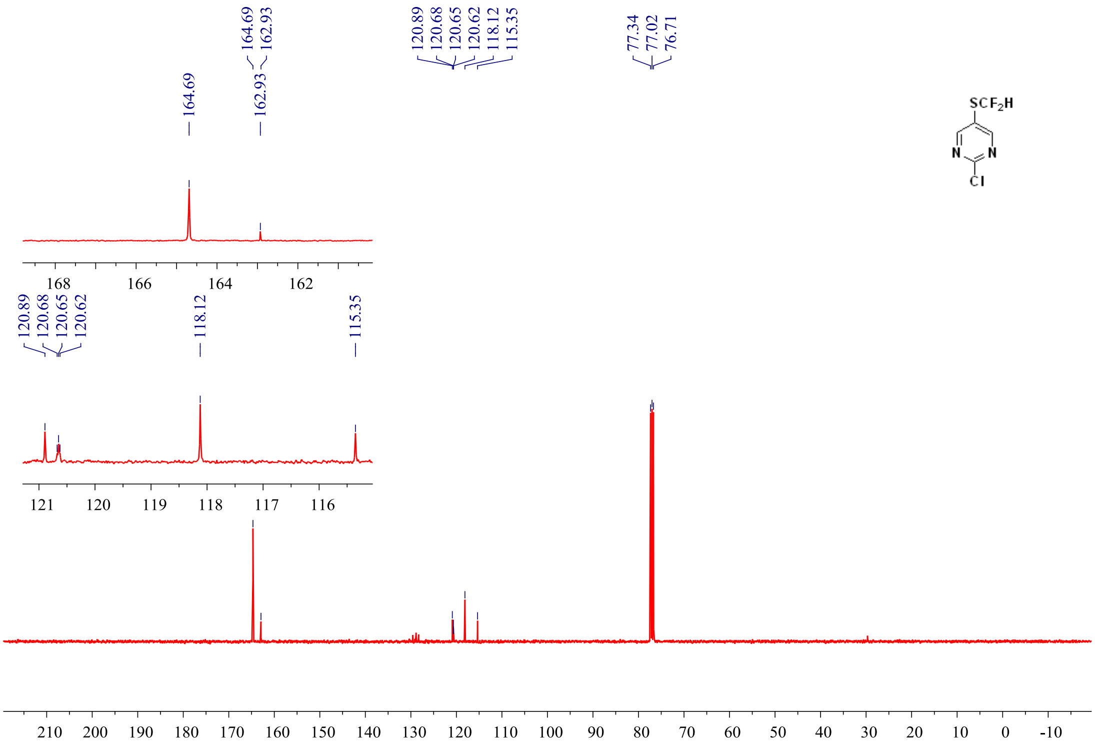

^{1}H NMR (400 MHz, $CDCl_3$) 5-bromo-2-((difluoromethyl)thio)pyrimidine 4ac

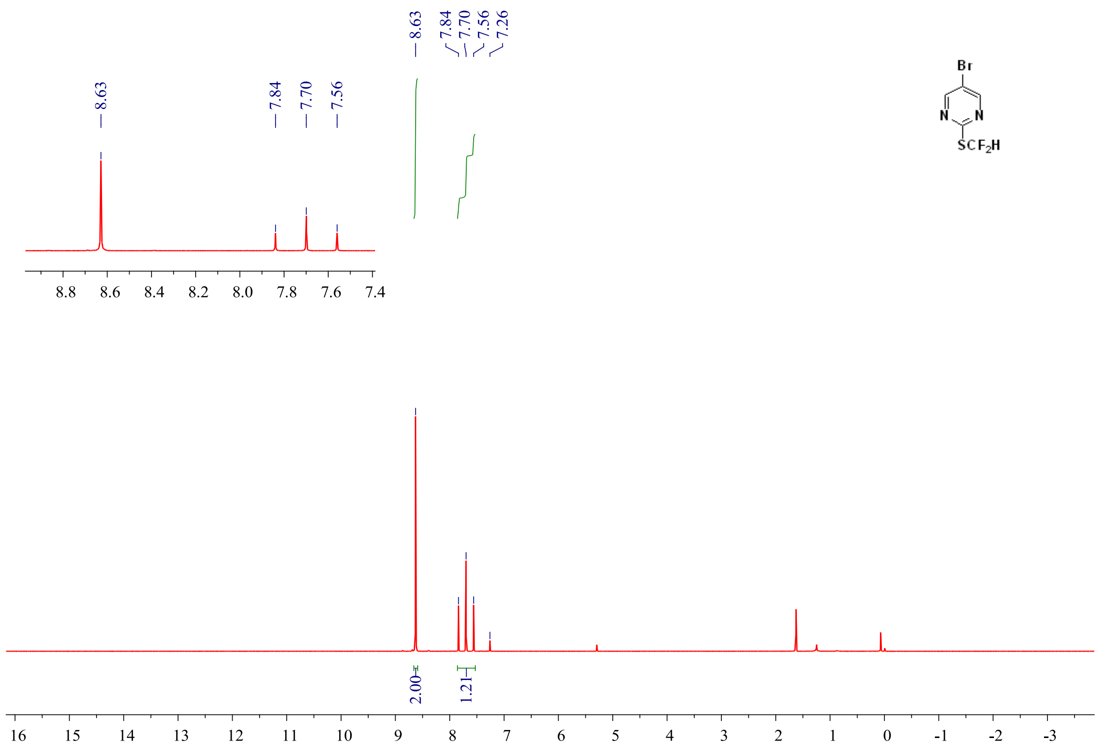

^{19}F NMR (376 MHz, $CDCl_3$) 5-bromo-2-((difluoromethyl)thio)pyrimidine 4ac

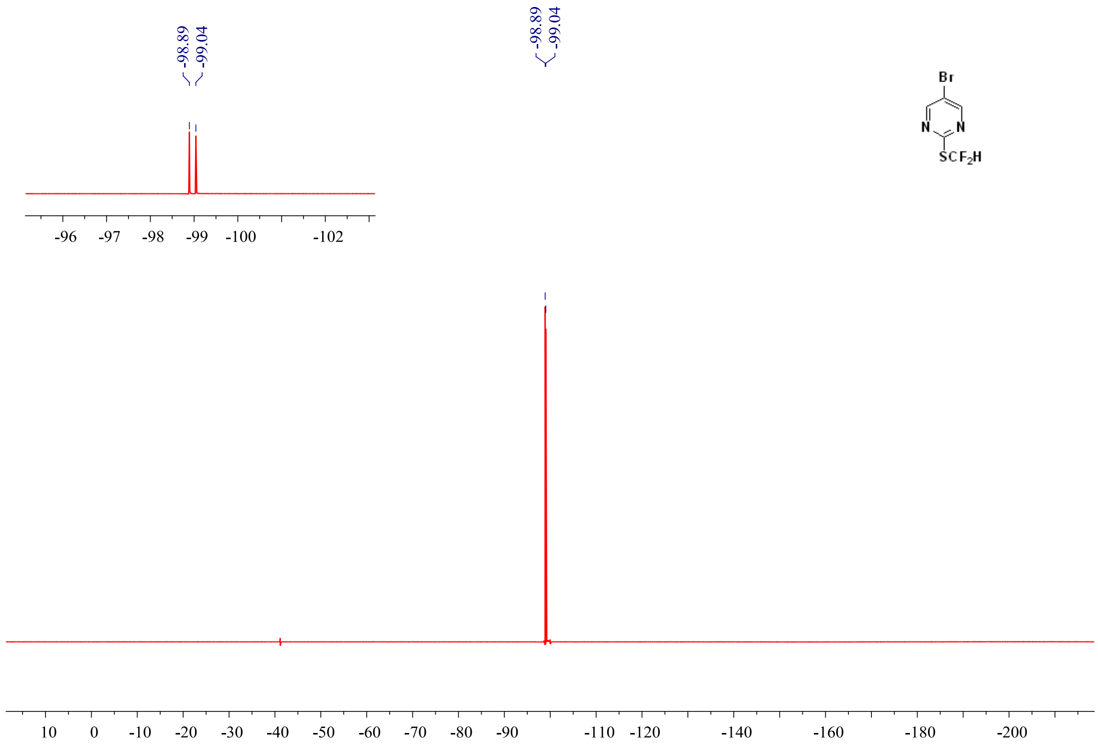

^{13}C NMR (101 MHz, $CDCl_3$) 5-bromo-2-((difluoromethyl)thio)pyrimidine 4ac

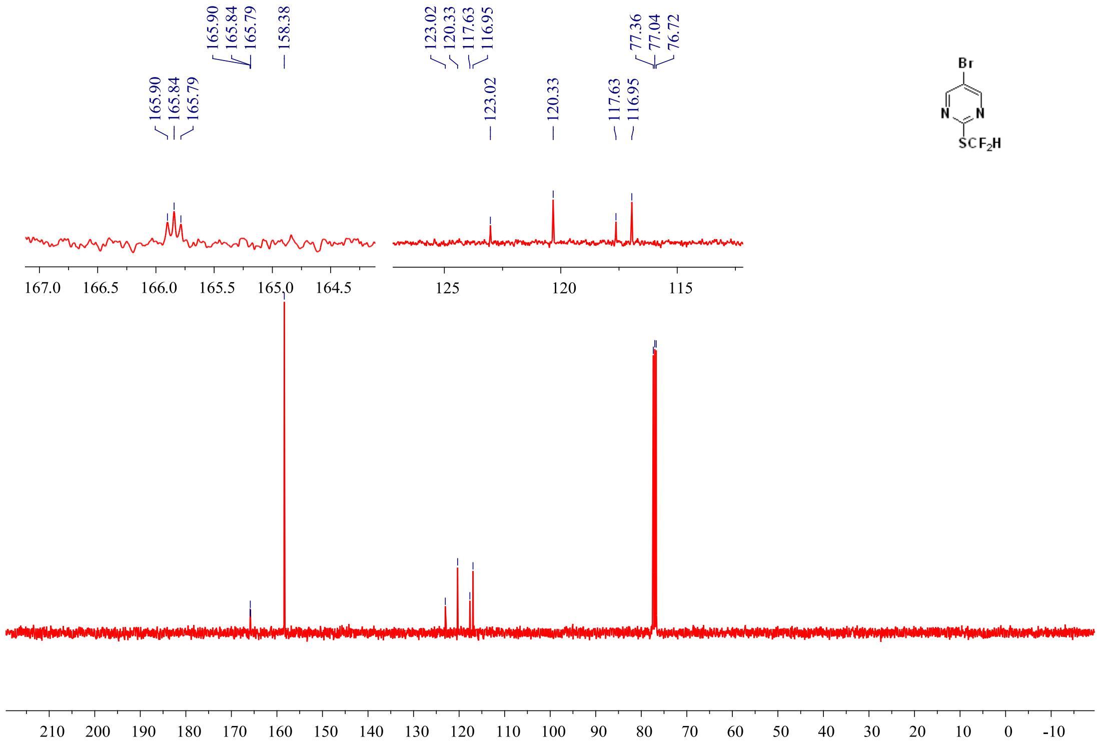

^{1}H NMR (400 MHz, $CDCl_3$) 5-chloro-2-((difluoromethyl)thio)pyrimidine 4ad

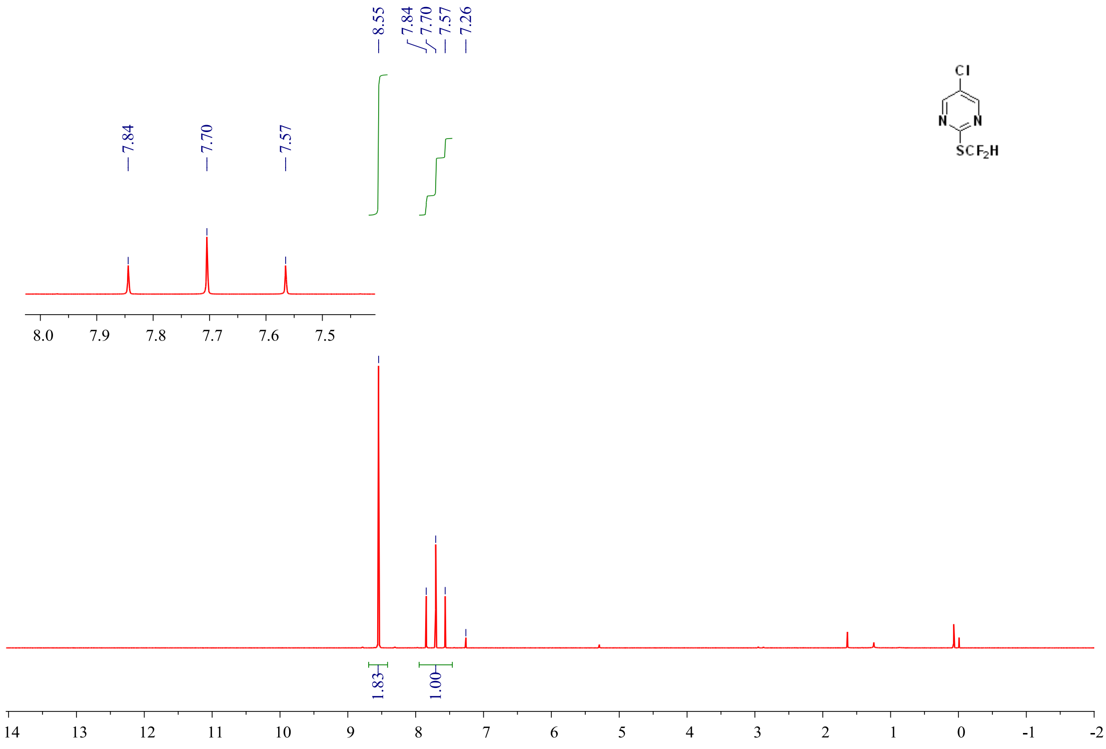

^{19}F NMR (376 MHz, $CDCl_3$) 5-chloro-2-((difluoromethyl)thio)pyrimidine 4ad

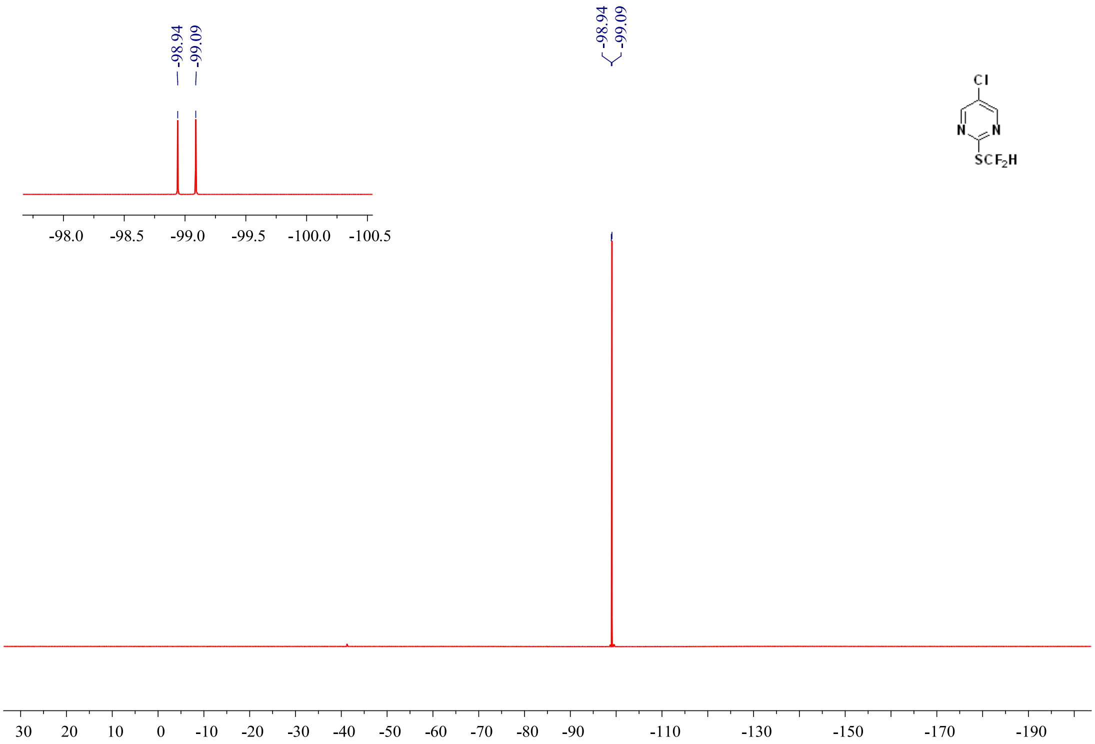

^{13}C NMR (101 MHz, $CDCl_3$) 5-chloro-2-((difluoromethyl)thio)pyrimidine 4ad

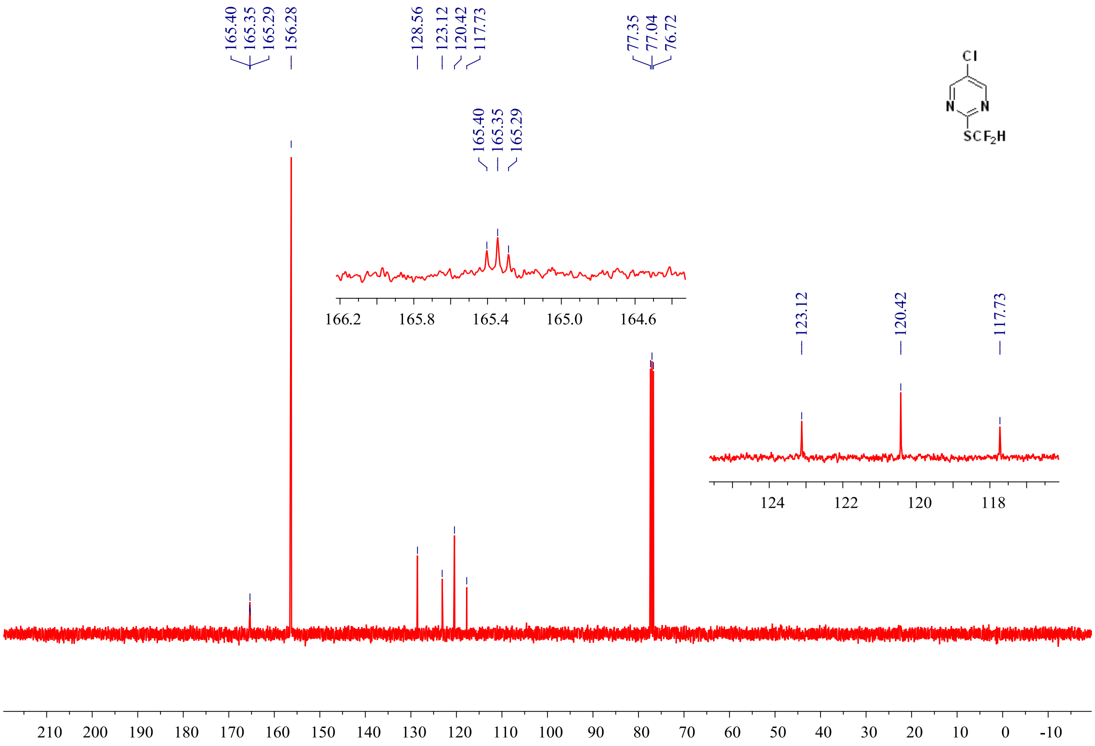

^{1}H NMR (400 MHz, CDCl$_3$) 2-((difluoromethyl)thio)pyrazine 4ae

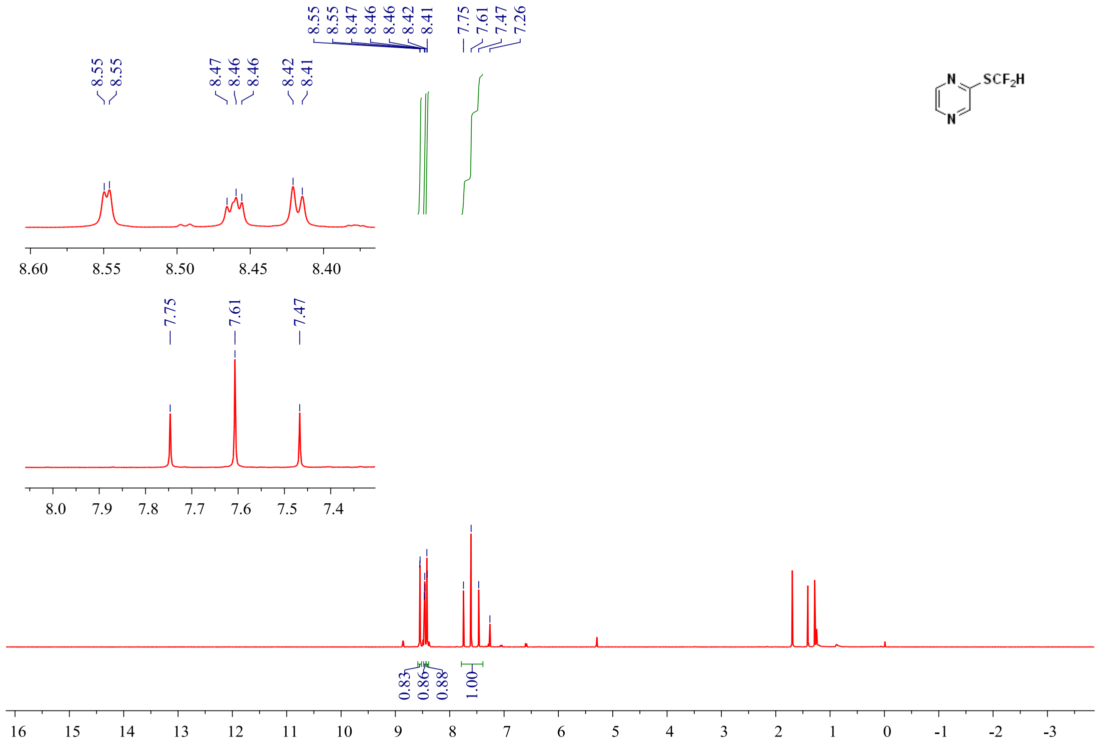

^{19}F NMR (376 MHz, CDCl$_3$) 2-((difluoromethyl)thio)pyrazine 4ae

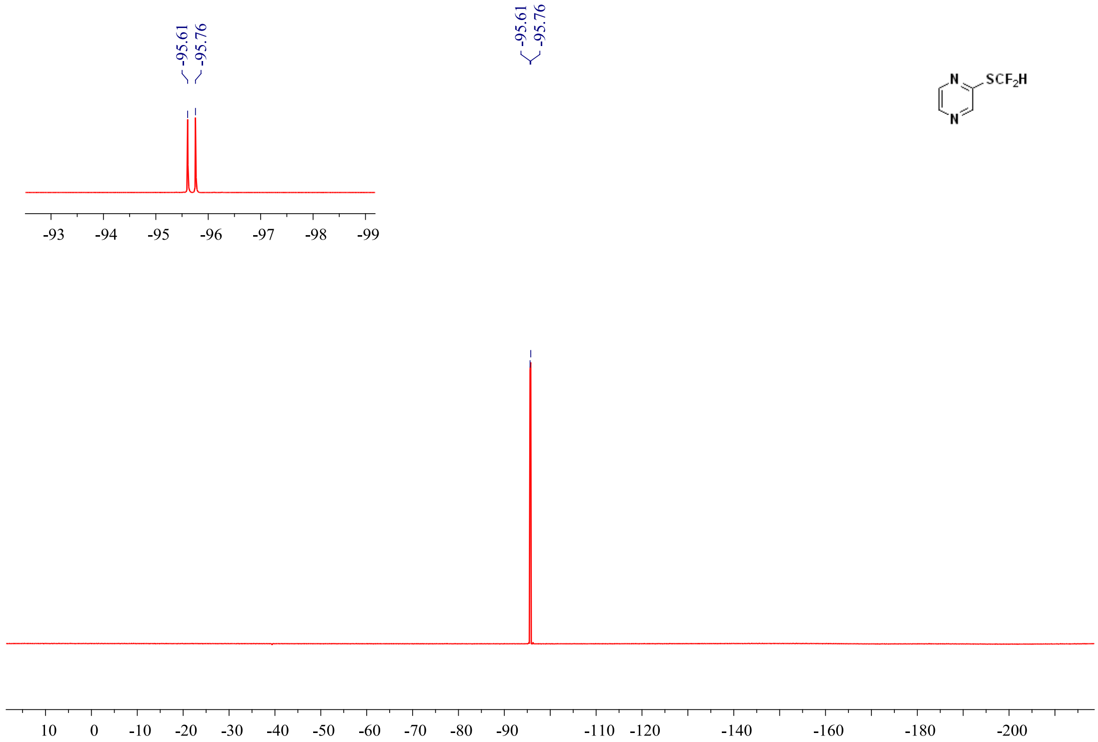

^{13}C NMR (101 MHz, $CDCl_3$) 2-((difluoromethyl)thio)pyrazine 4ae

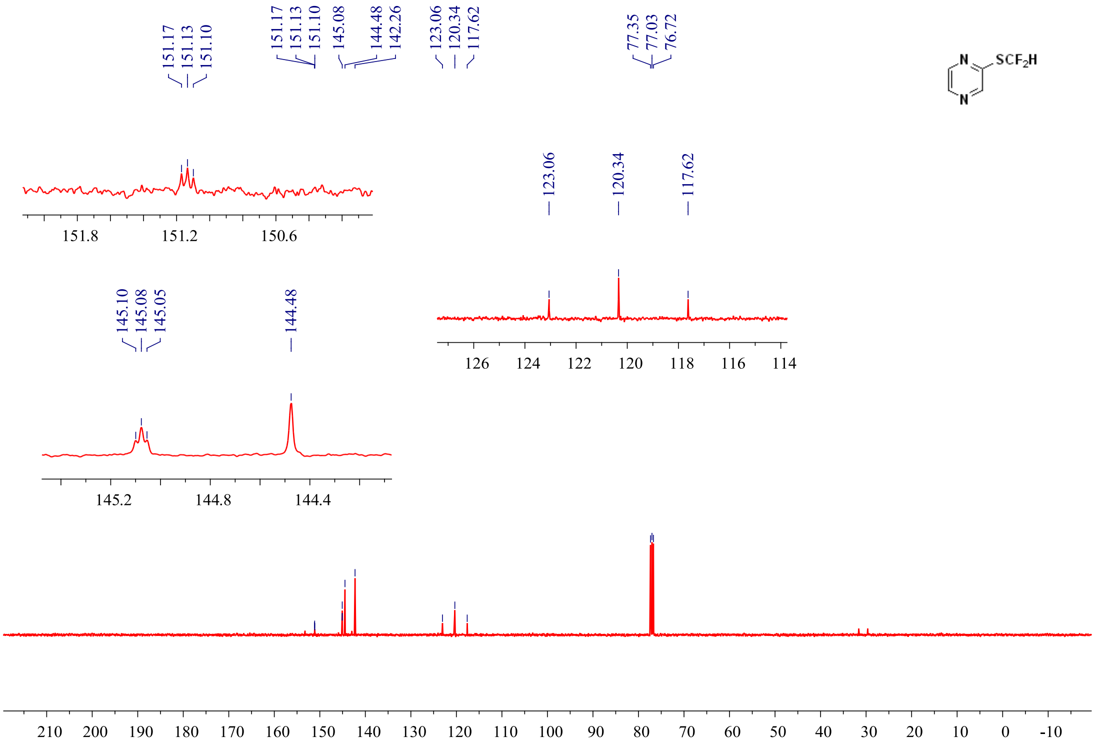

^{1}H NMR (400 MHz, $CDCl_3$) 2,5-bis((difluoromethyl)thio)pyrazine 4af

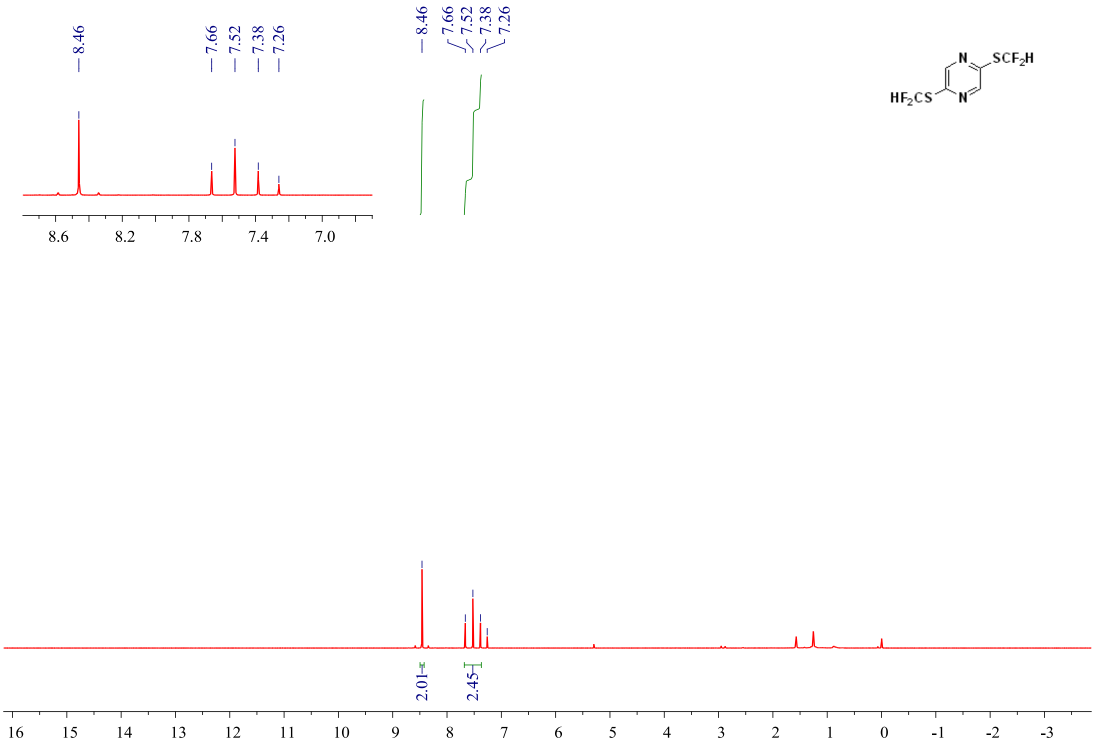

^{19}F NMR (376 MHz, $CDCl_3$) 2,5-bis((difluoromethyl)thio)pyrazine 4af

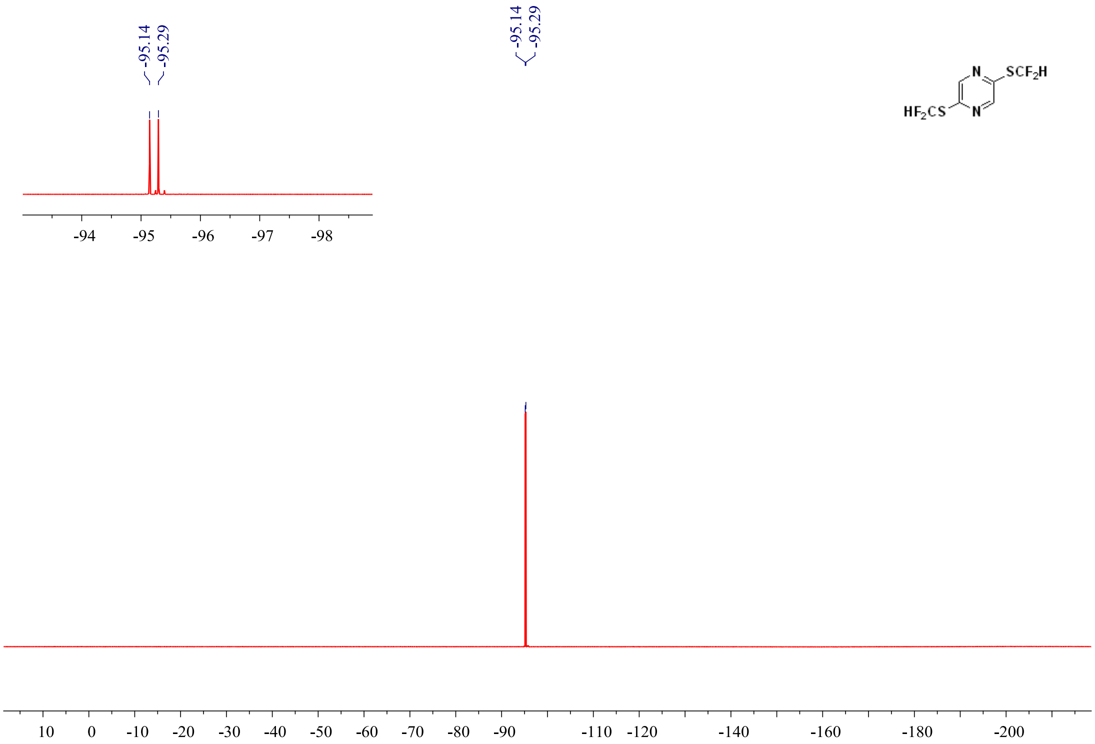

^{13}C NMR (101 MHz, $CDCl_3$) 2,5-bis((difluoromethyl)thio)pyrazine 4af

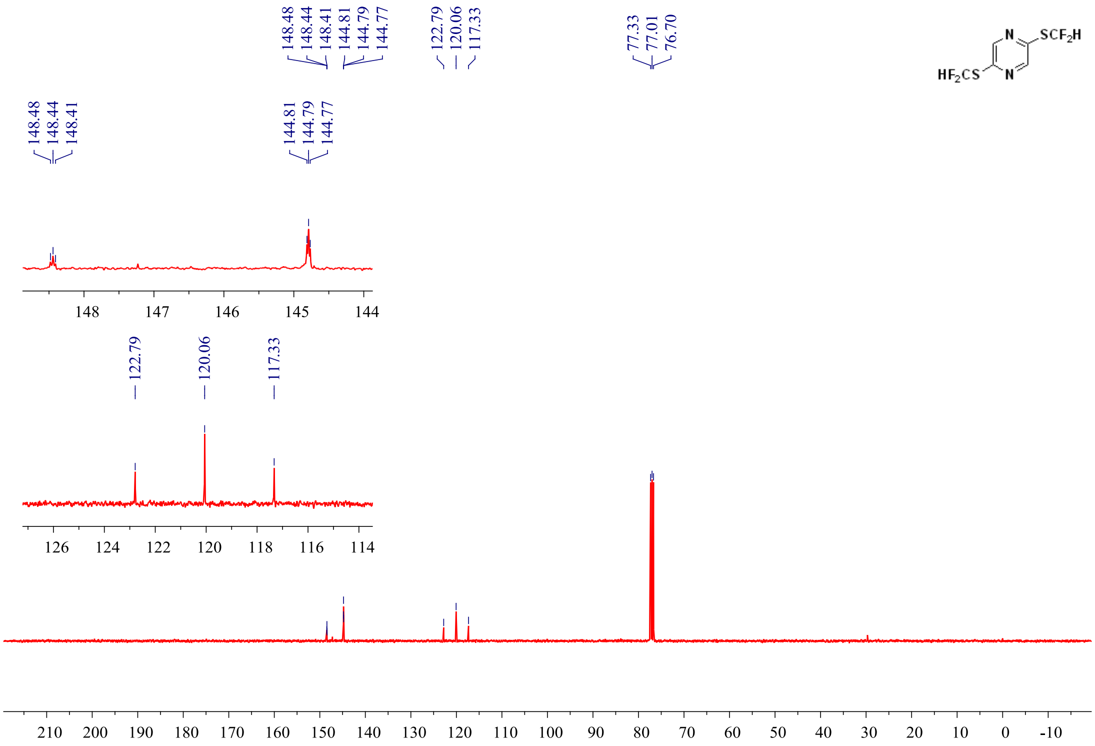

^{1}H NMR (400 MHz, $CDCl_3$) methyl 3-((difluoromethyl)thio)pyrazine-2-carboxylate 4ag

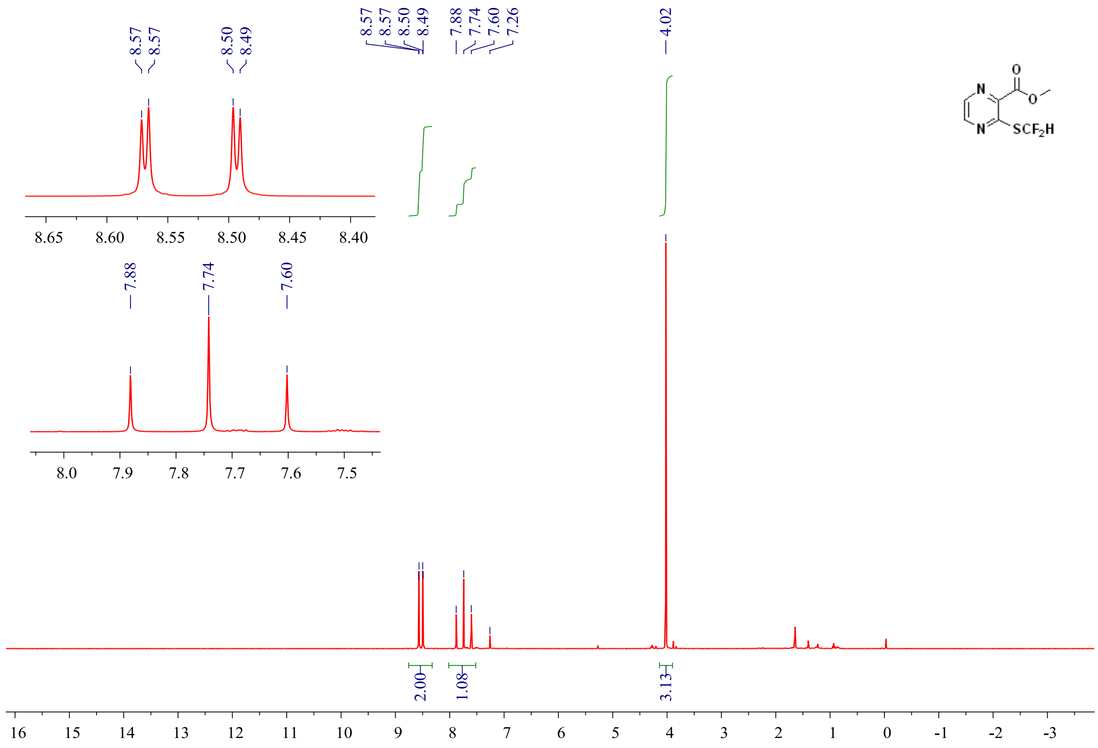

^{19}F NMR (376 MHz, $CDCl_3$) methyl 3-((difluoromethyl)thio)pyrazine-2-carboxylate 4ag

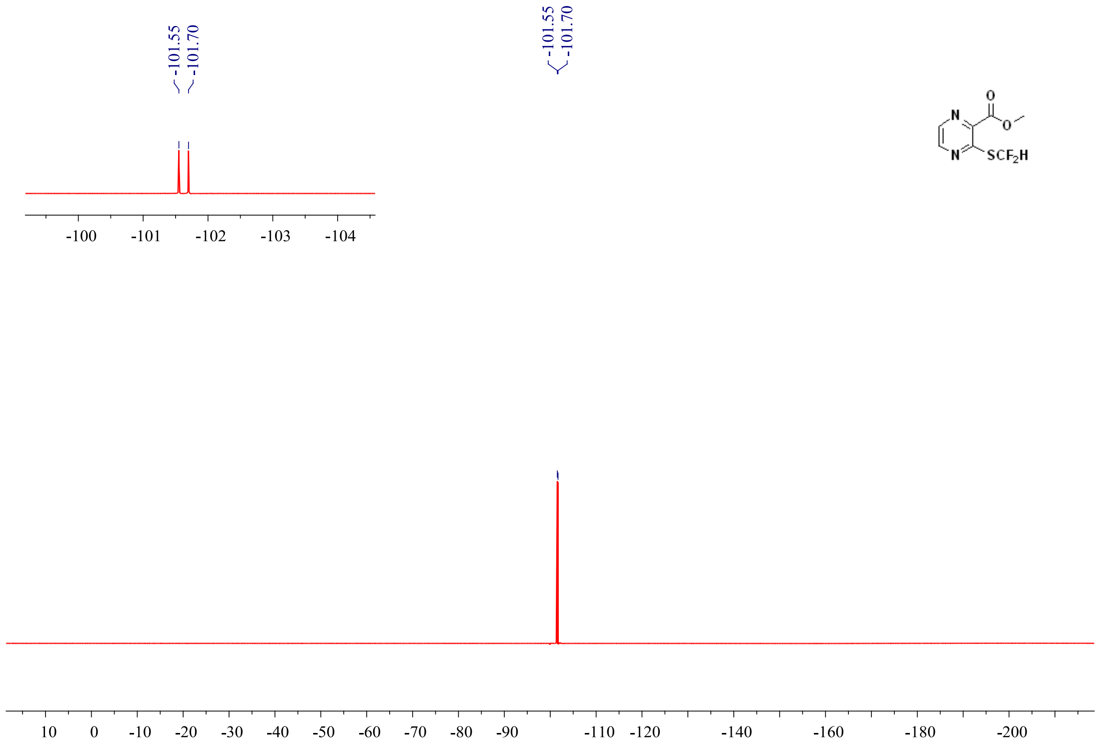

^{13}C NMR (101 MHz, CDCl$_3$) methyl 3-((difluoromethyl)thio)pyrazine-2-carboxylate 4ag

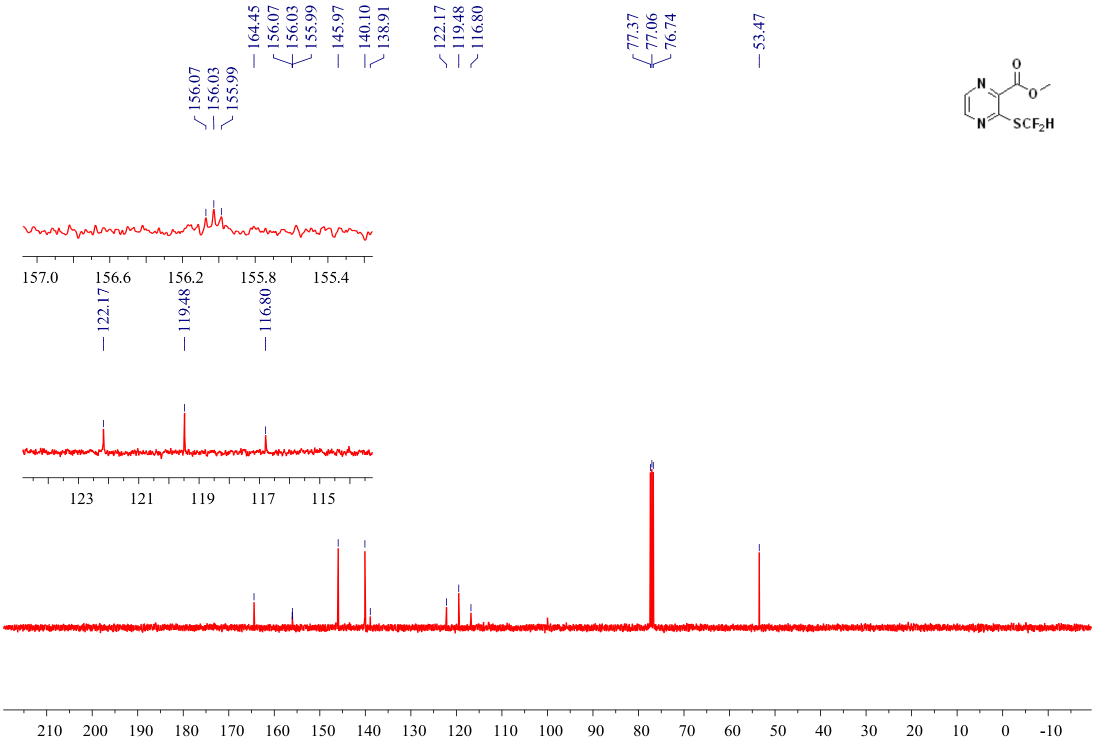

^{1}H NMR (400 MHz, CDCl$_3$) 4-chloro-6-((difluoromethyl)thio)quinazoline 4ah

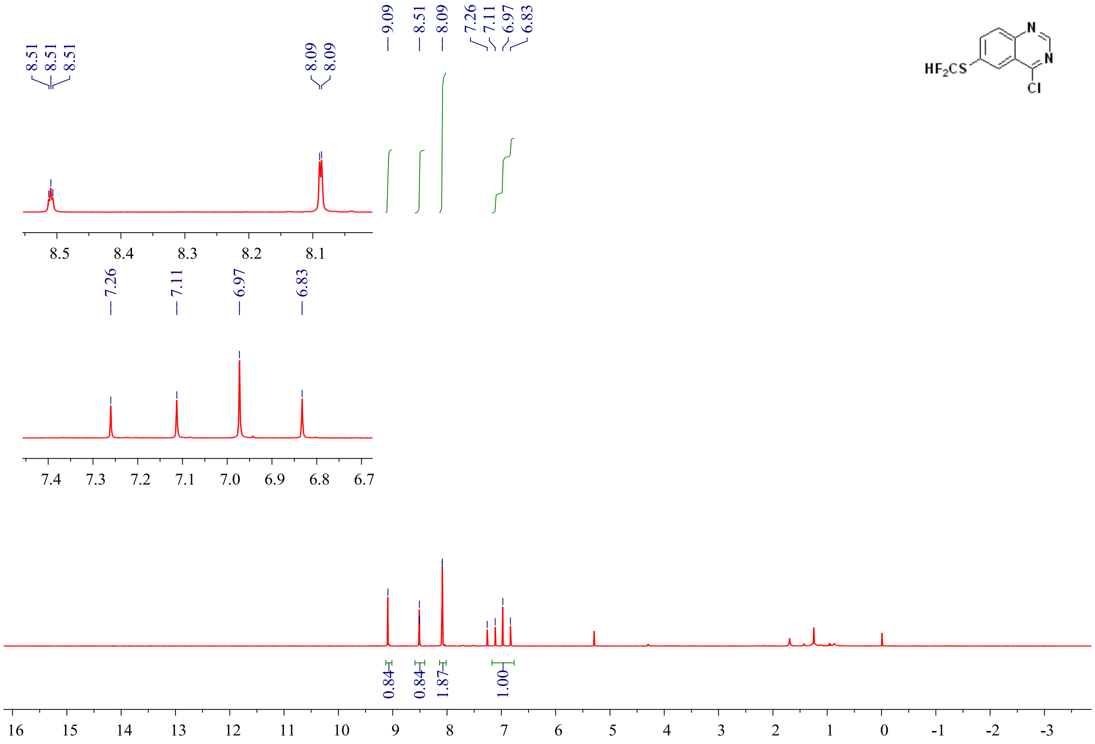

^{19}F NMR (376 MHz, $CDCl_3$) 4-chloro-6-((difluoromethyl)thio)quinazoline 4ah

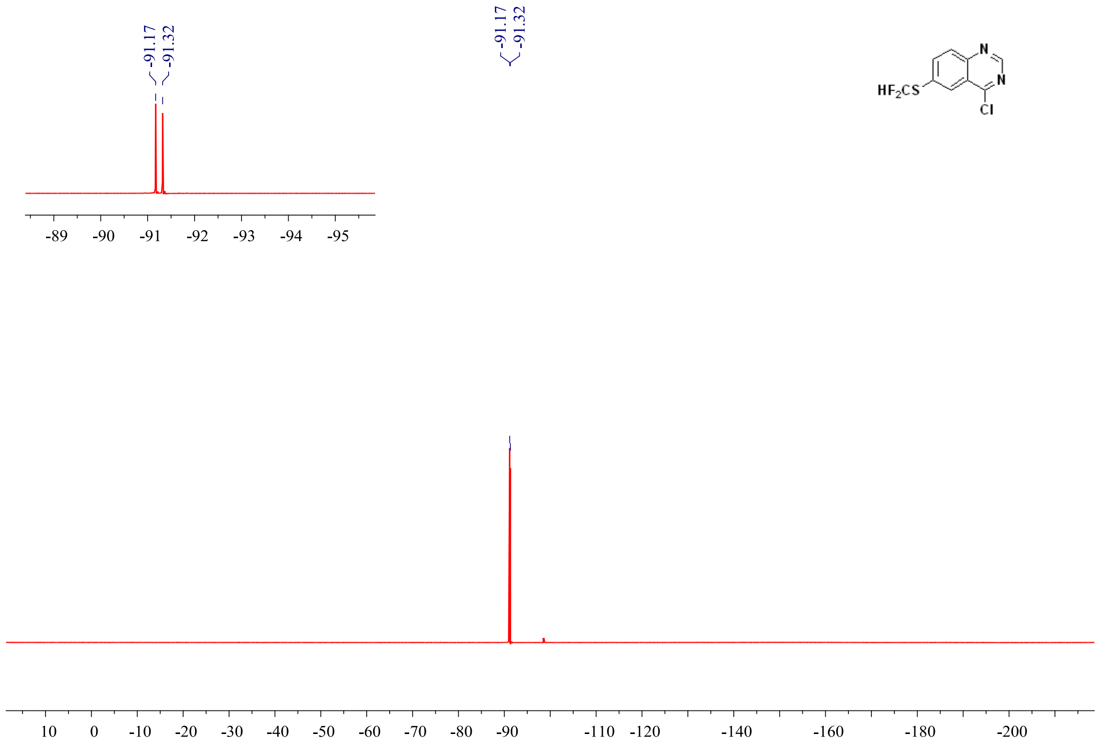

^{13}C NMR (101 MHz, $CDCl_3$) 4-chloro-6-((difluoromethyl)thio)quinazoline 4ah

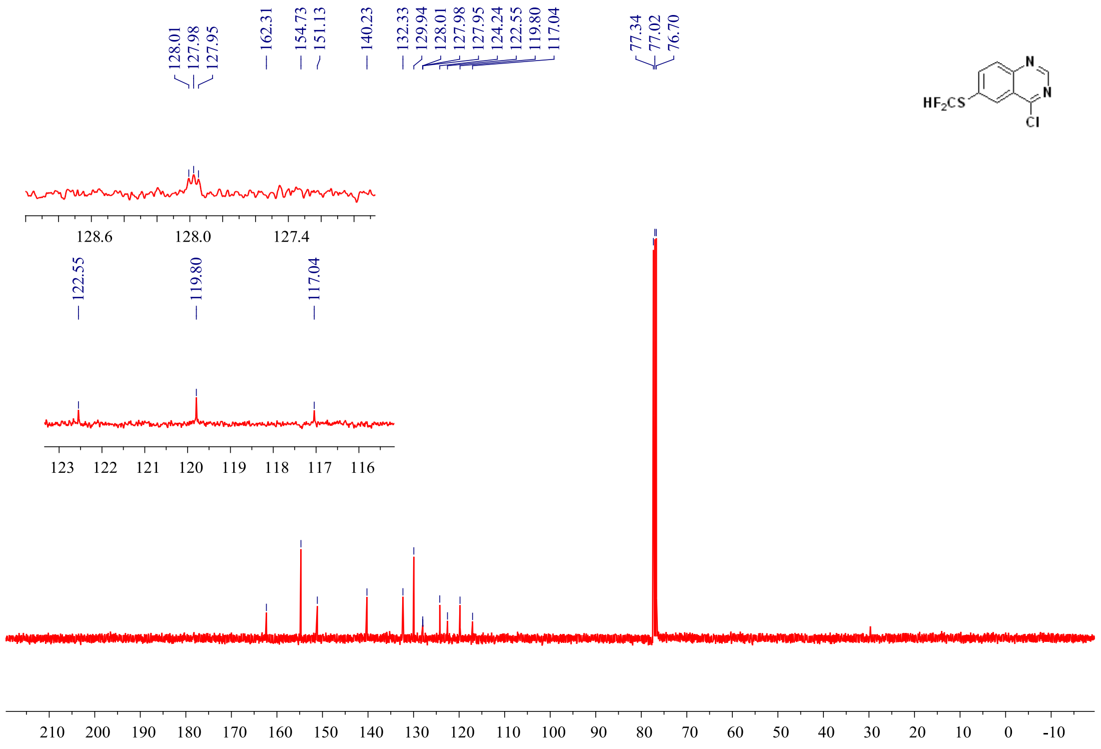

^{1}H NMR (400 MHz, $CDCl_3$) *tert*-butyl 5-((difluoromethyl)thio)-1*H*-indazole-1-carboxylate 4ai

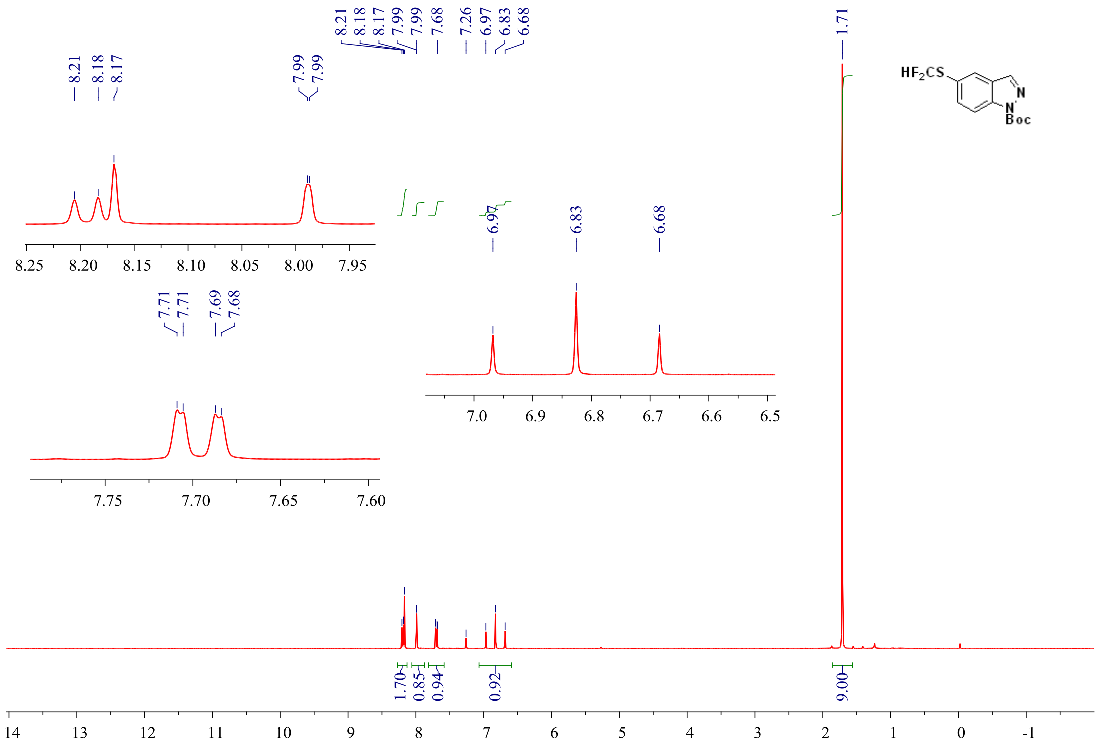

^{19}F NMR (376 MHz, $CDCl_3$) *tert*-butyl 5-((difluoromethyl)thio)-1*H*-indazole-1-carboxylate 4ai

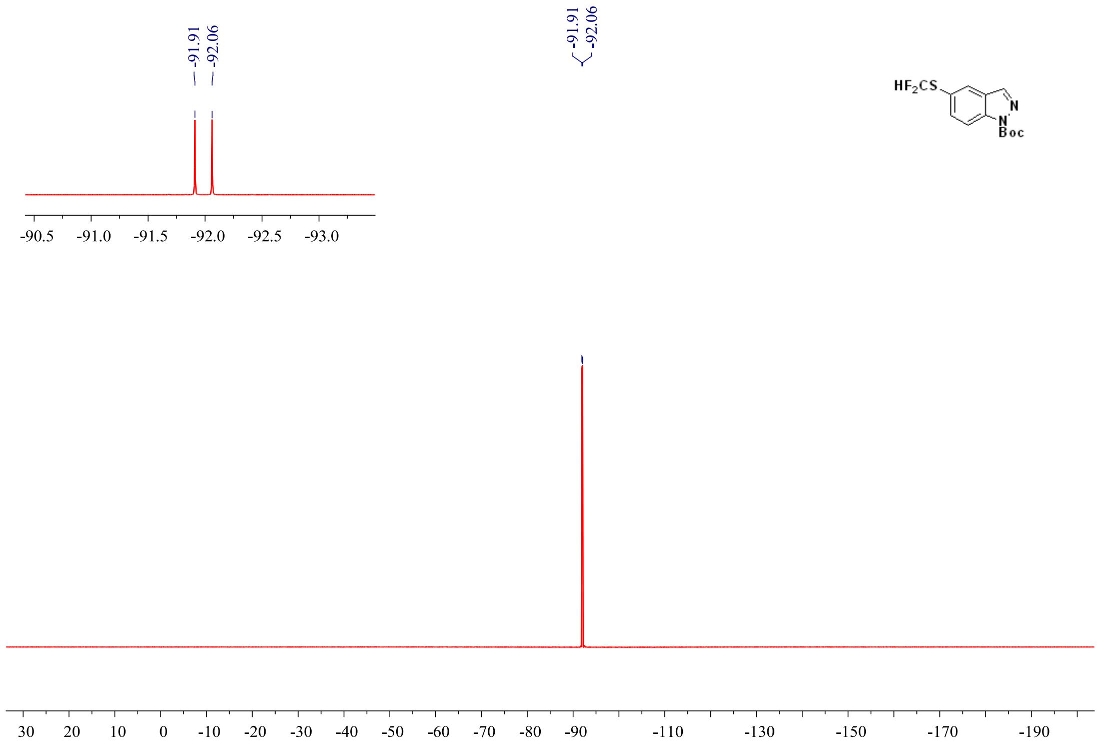

^{13}C NMR (101 MHz, $CDCl_3$) *tert*-butyl 5-((difluoromethyl)thio)-1*H*-indazole-1-carboxylate 4ai

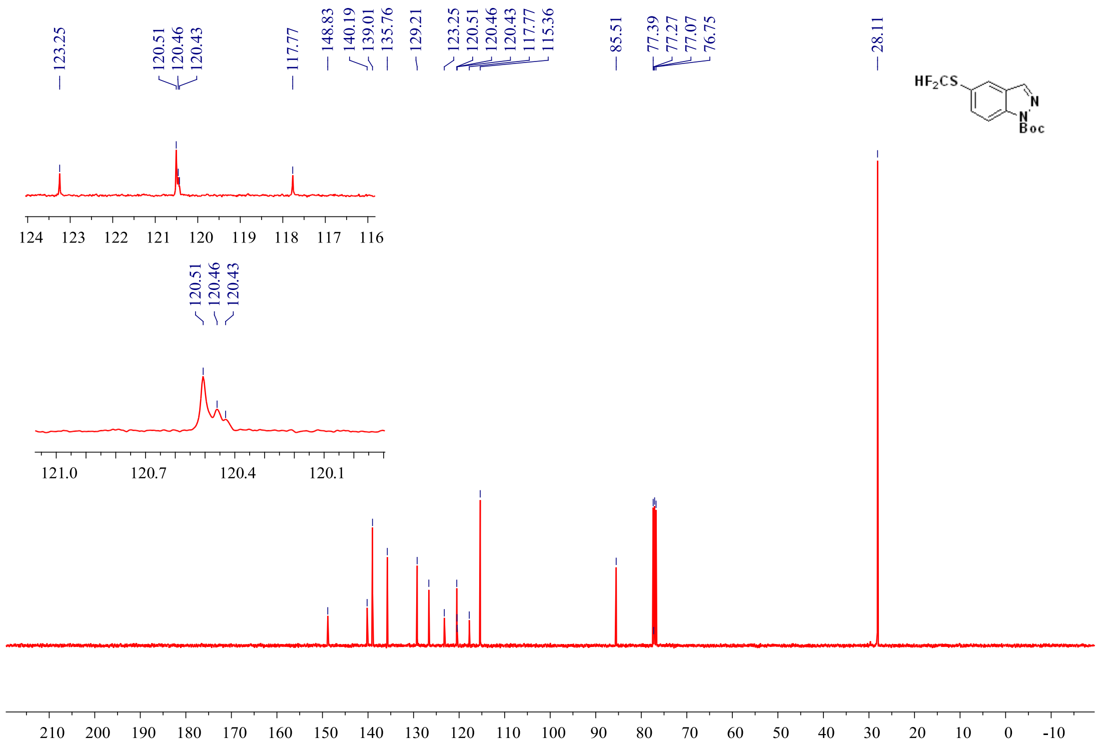

^{1}H NMR (400 MHz, $CDCl_3$) 7-chloro-2-((difluoromethyl)thio)thieno[3,2-b] pyridine-6-carbonitrile 4aj

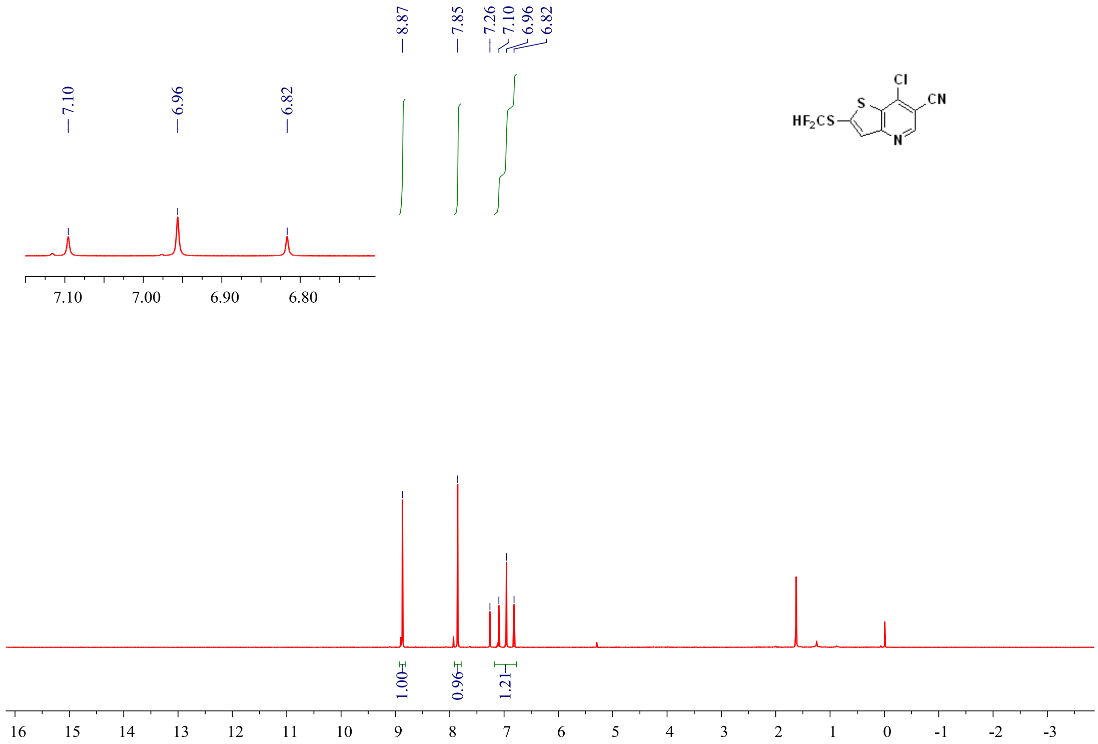

^{19}F NMR (376 MHz, CDCl$_3$) 7-chloro-2-((difluoromethyl)thio)thieno[3,2-b] pyridine-6-carbonitrile 4aj

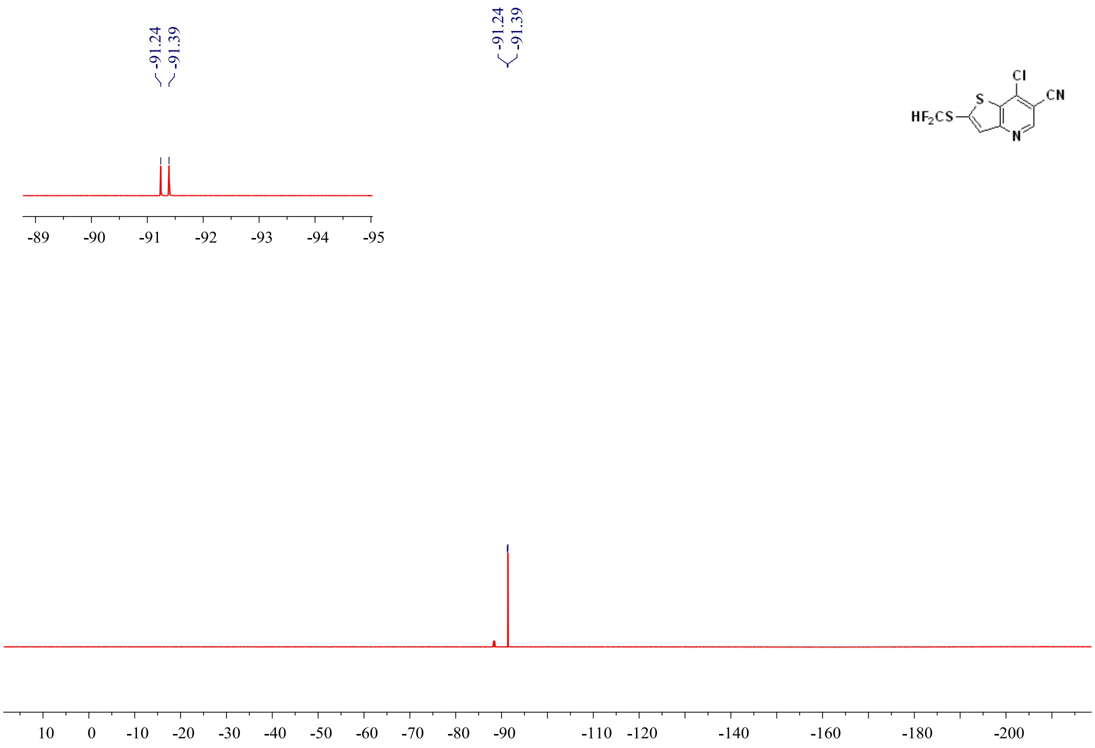

^{13}C NMR (101 MHz, CDCl$_3$) 7-chloro-2-((difluoromethyl)thio)thieno[3,2-b] pyridine-6-carbonitrile 4aj

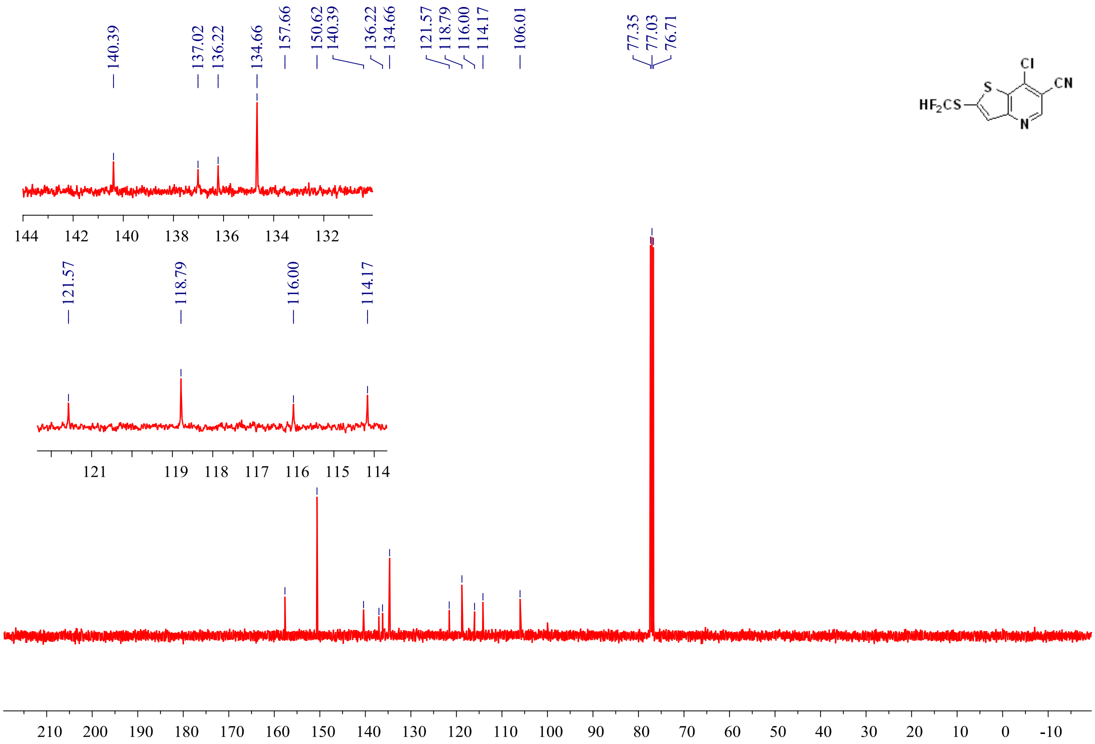

^{1}H NMR (400 MHz, $CDCl_3$) 6-((difluoromethyl)thio)benzo[d]thiazole 4ak

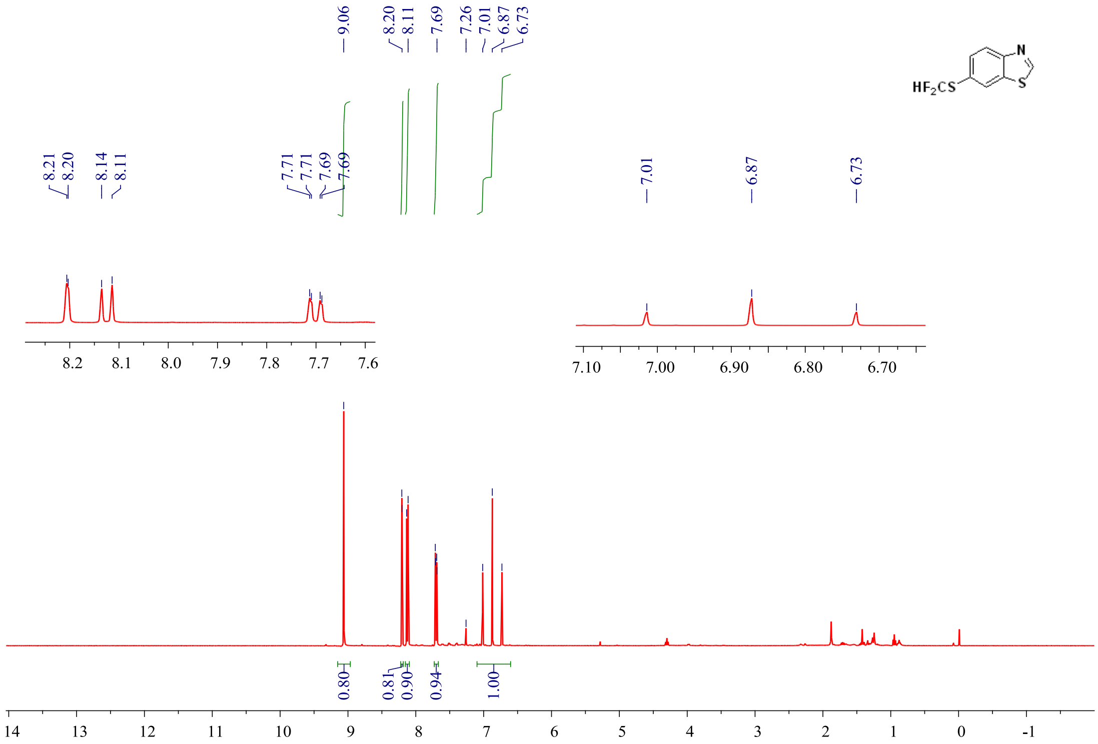

^{19}F NMR (376 MHz, $CDCl_3$) 6-((difluoromethyl)thio)benzo[d]thiazole 4ak

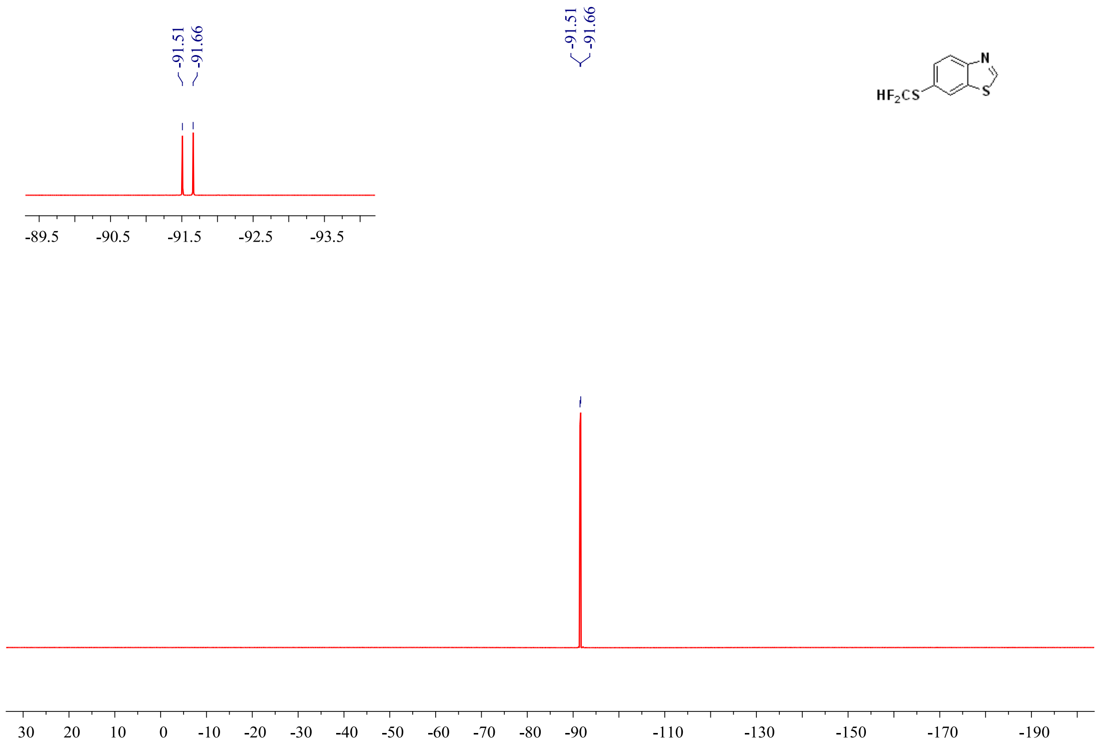

^{13}C NMR (101 MHz, $CDCl_3$) 6-((difluoromethyl)thio)benzo[d]thiazole 4ak

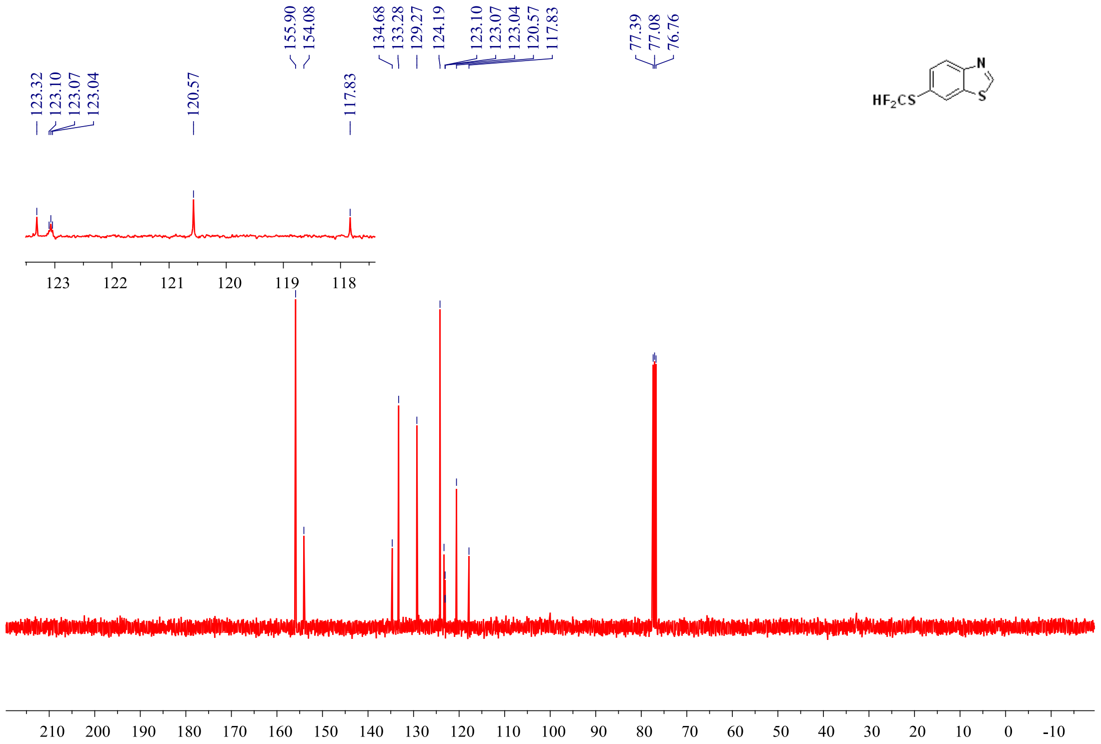

^{1}H NMR (400 MHz, $CDCl_3$) 6-((difluoromethyl)thio)nicotinonitrile 4al

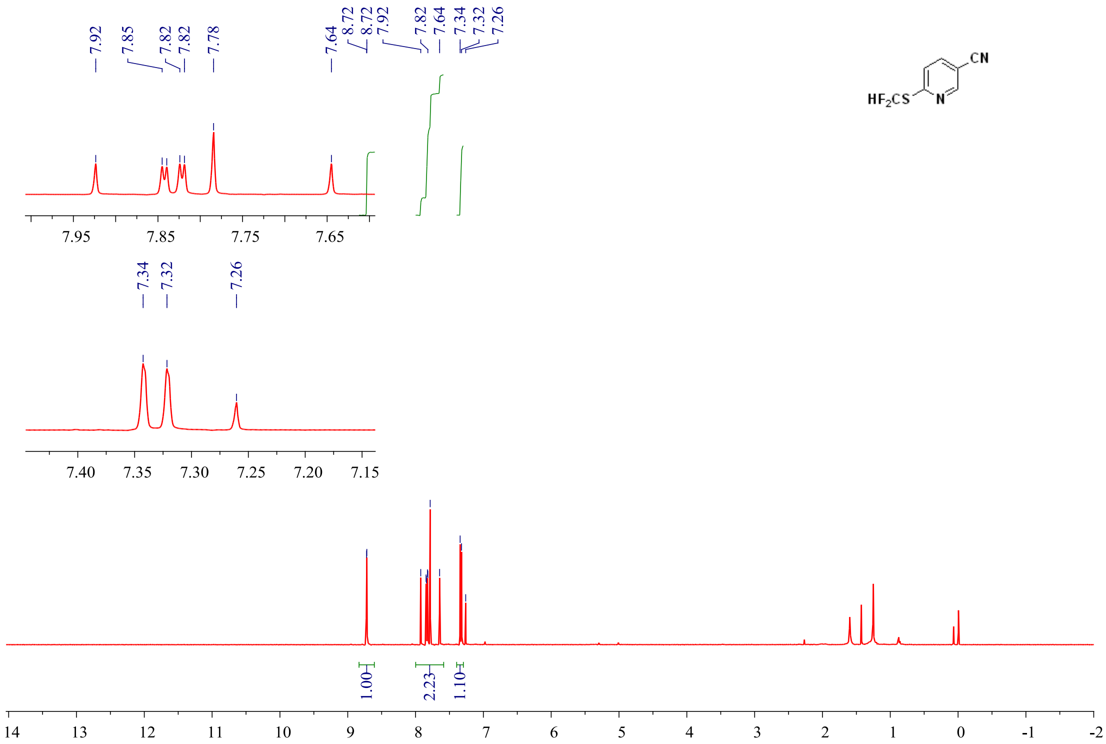

^{19}F NMR (376 MHz, $CDCl_3$) 6-((difluoromethyl)thio)nicotinonitrile 4al

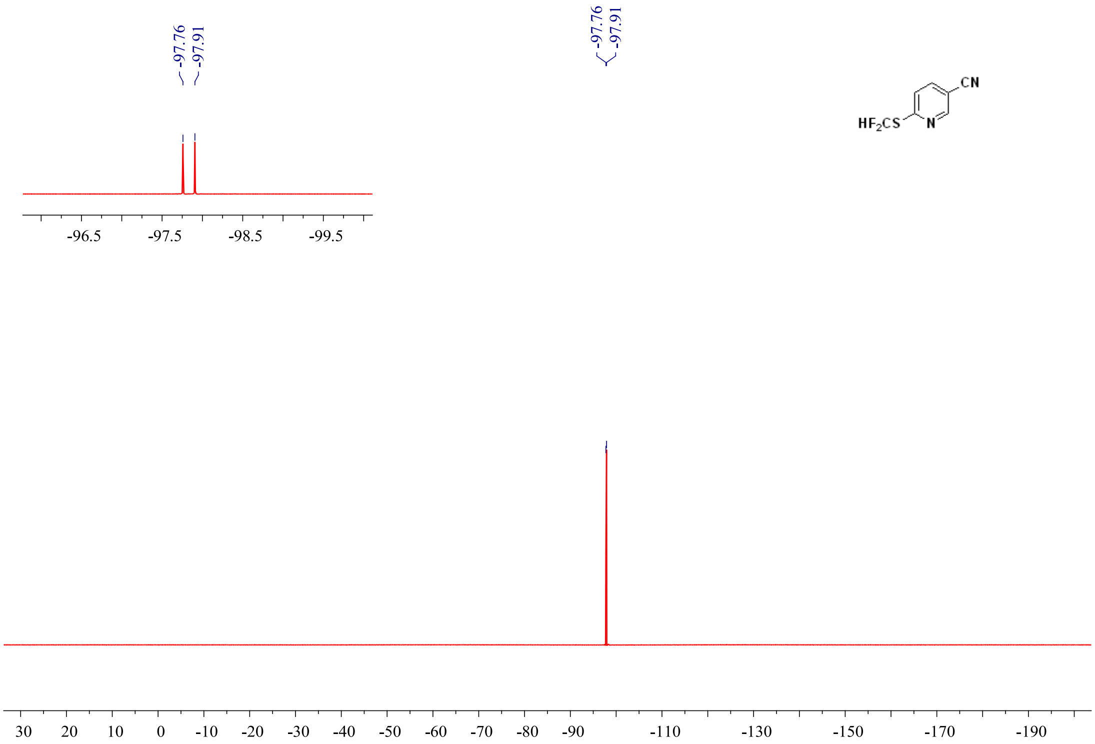

^{13}C NMR (101 MHz, $CDCl_3$) 6-((difluoromethyl)thio)nicotinonitrile 4al

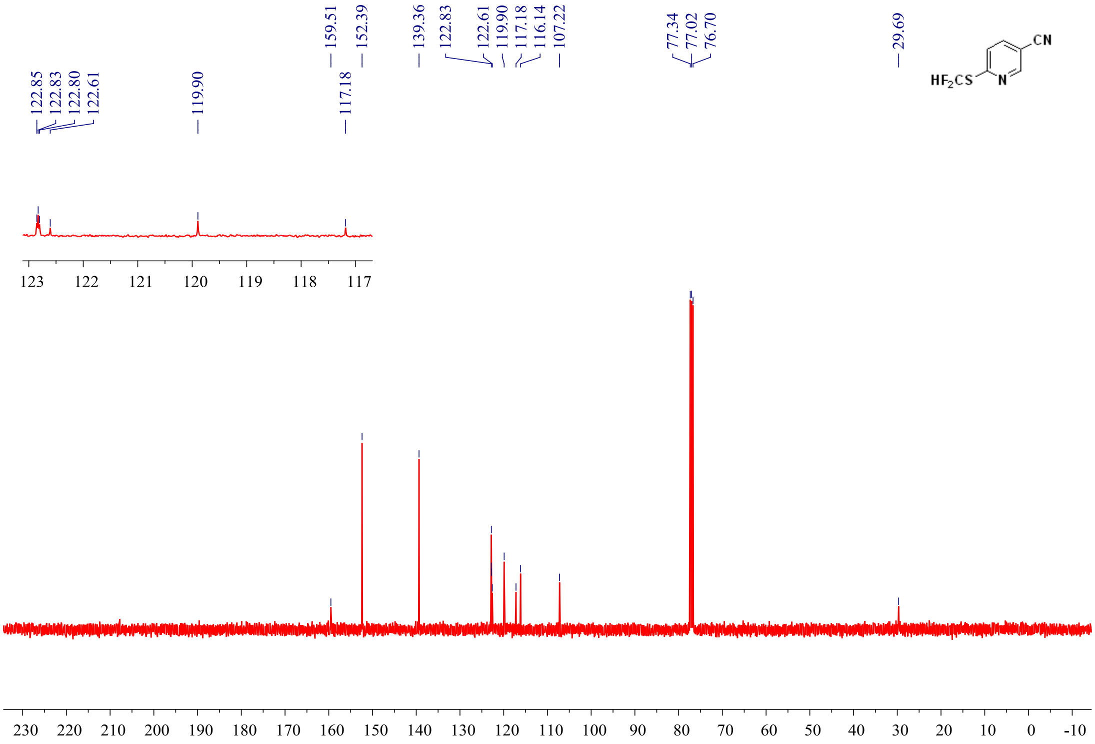

^{1}H NMR (400 MHz, $CDCl_3$) 3,5-dichloro-2-((difluoromethyl)thio)pyridine 4am

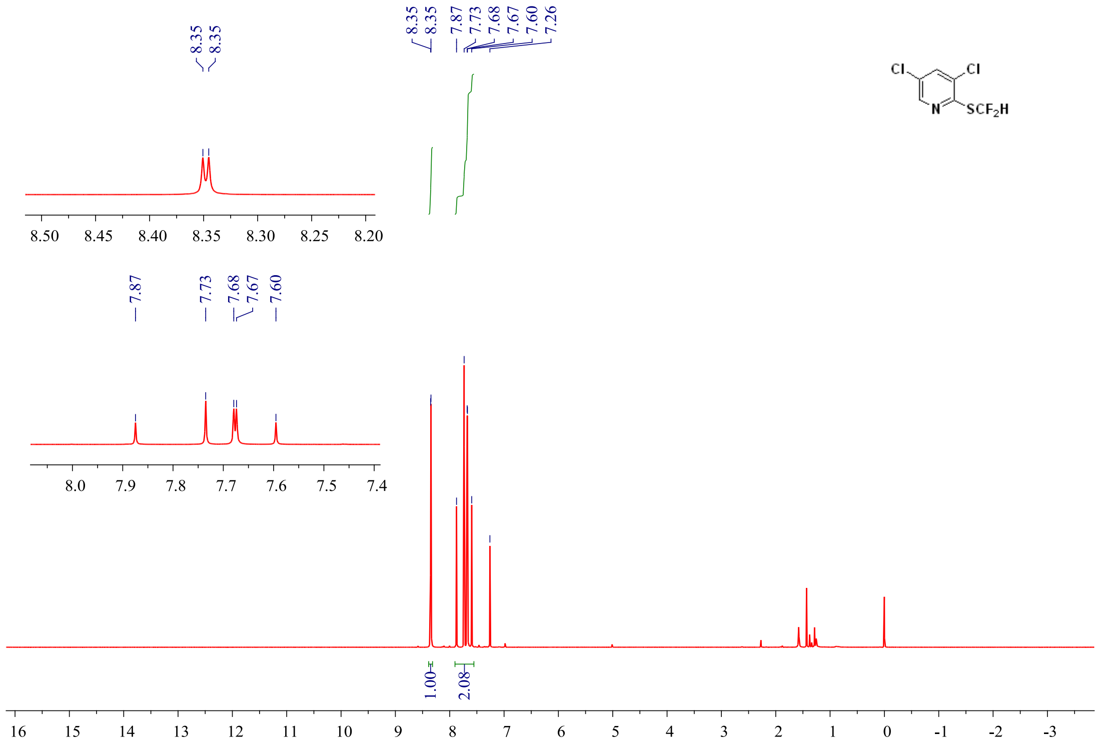

^{19}F NMR (376 MHz, $CDCl_3$) 3,5-dichloro-2-((difluoromethyl)thio)pyridine 4am

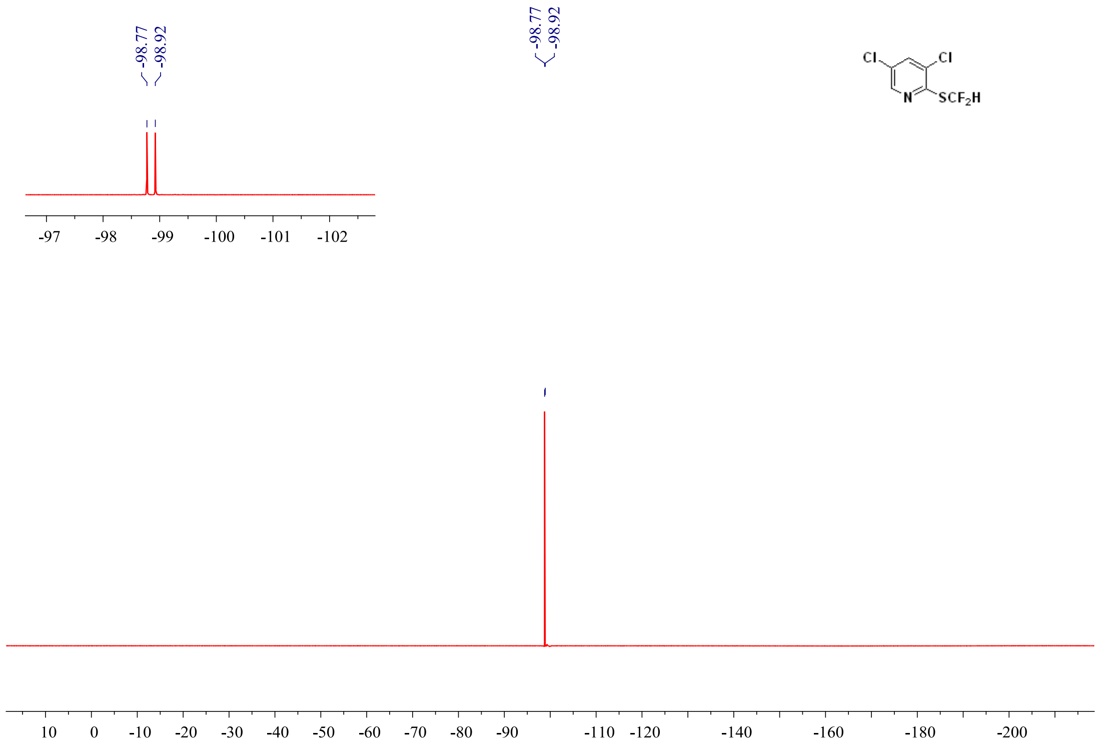

^{13}C NMR (101 MHz, CDCl$_3$) 3,5-dichloro-2-((difluoromethyl)thio)pyridine 4am

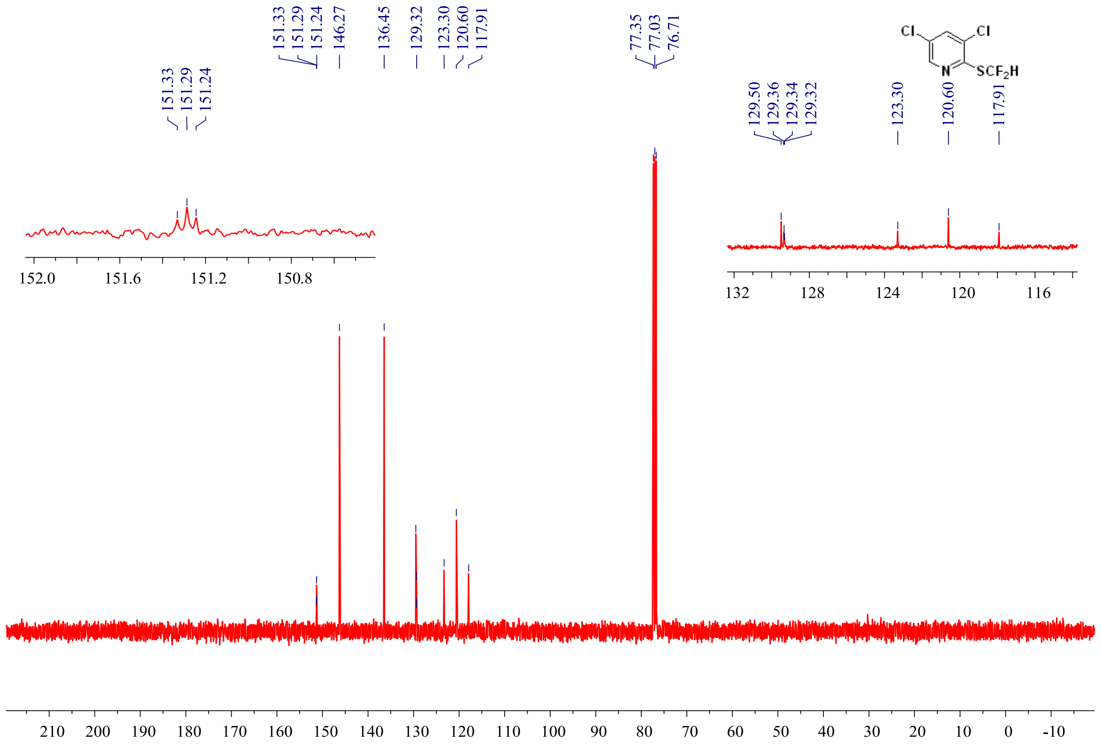

^{1}H NMR (400 MHz, CDCl$_3$) 1-(6-((difluoromethyl)thio)pyridin-3-yl) ethanone 4an

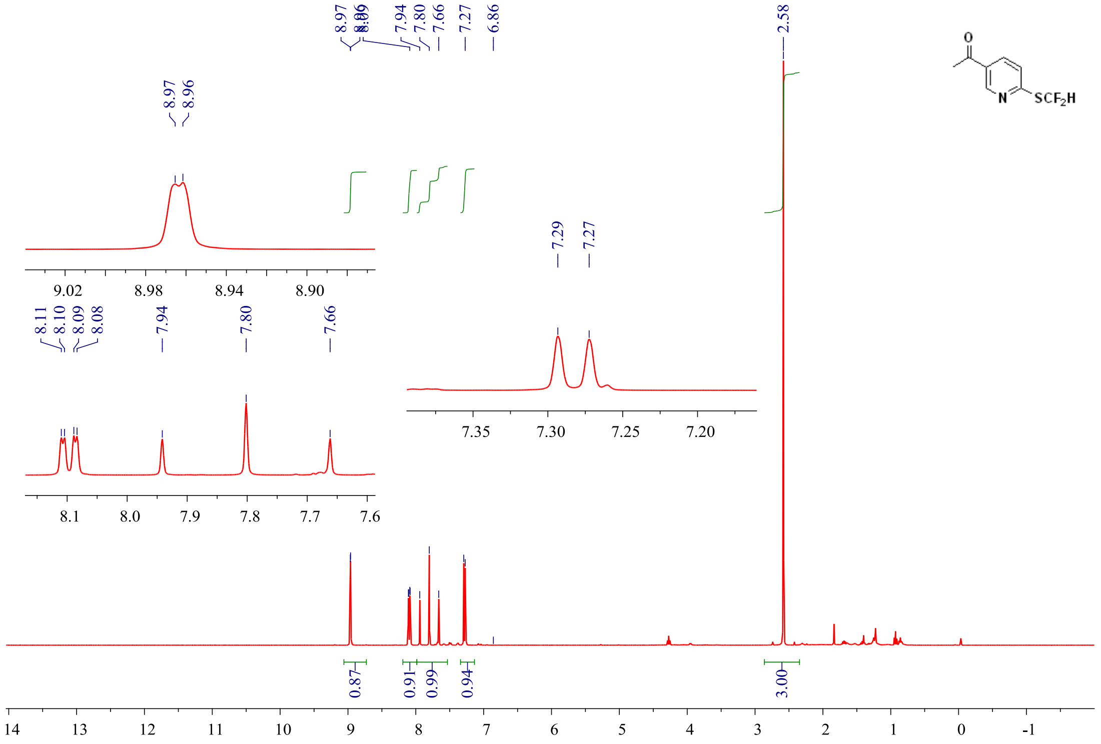

^{1}H NMR (400 MHz, $CDCl_3$) 1-(6-((difluoromethyl)thio)pyridin-3-yl) ethanone 4an

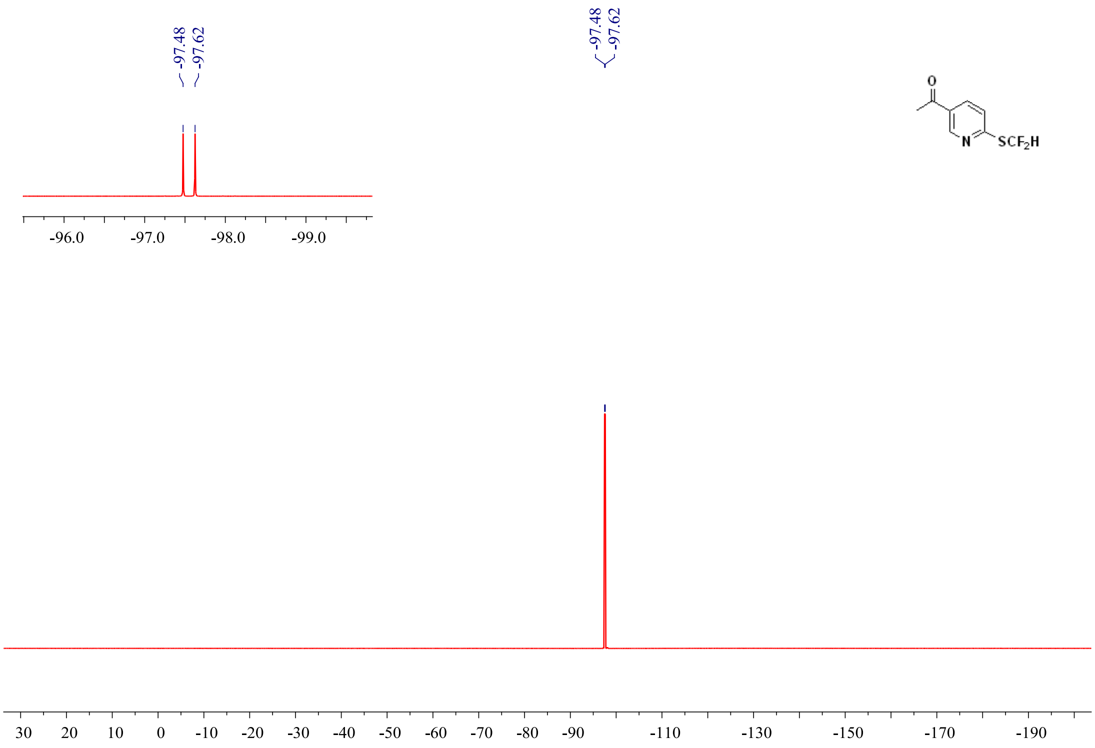

^{13}C NMR (101 MHz, $CDCl_3$) 1-(6-((difluoromethyl)thio)pyridin-3-yl) ethanone 4an

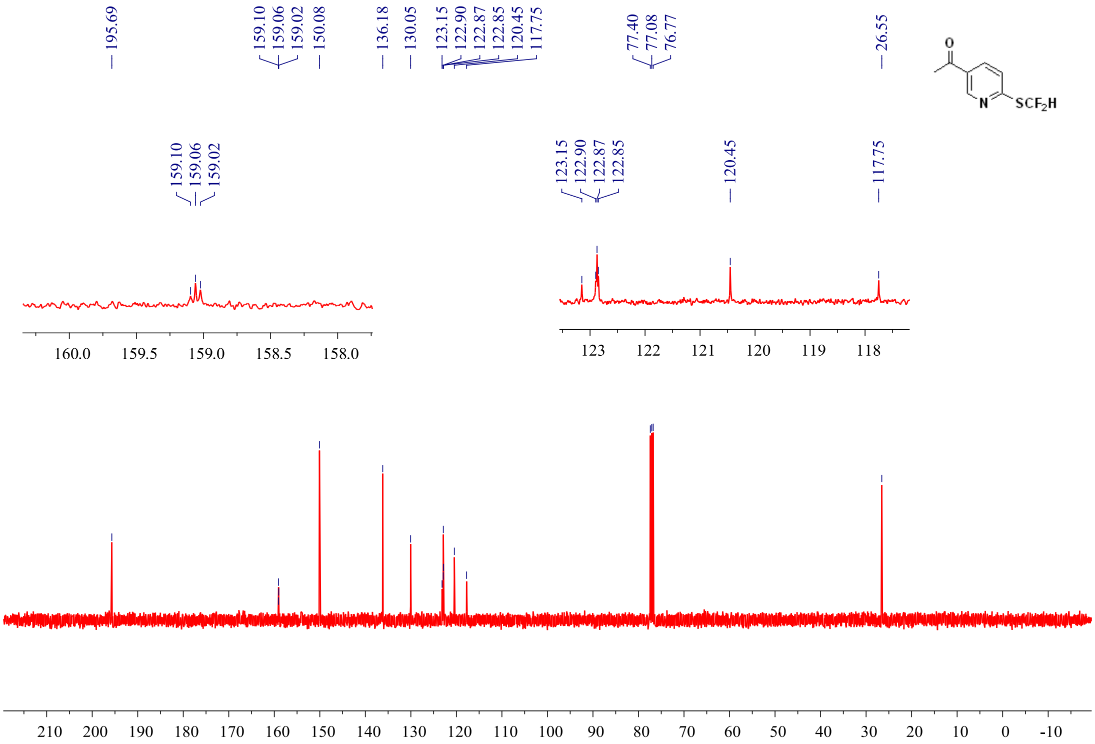

^{1}H NMR (400 MHz, $CDCl_3$) 4-bromo-2-((difluoromethyl)thio)pyridine 4ao

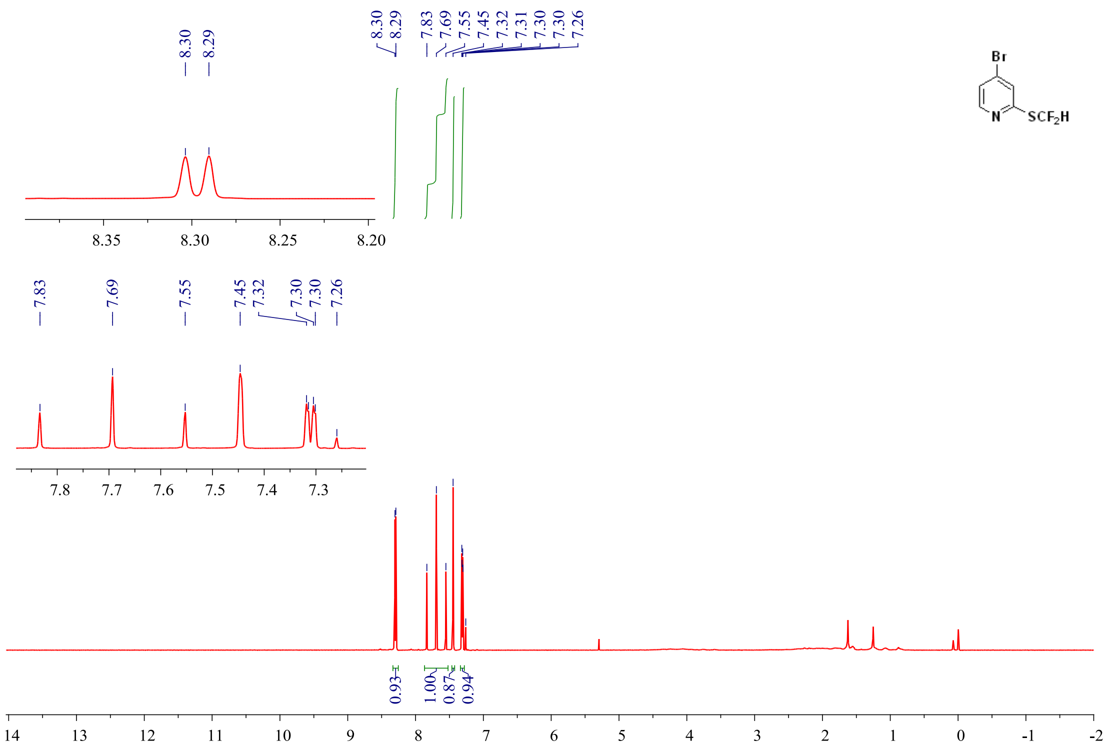

^{19}F NMR (376 MHz, $CDCl_3$) 4-bromo-2-((difluoromethyl)thio)pyridine 4ao

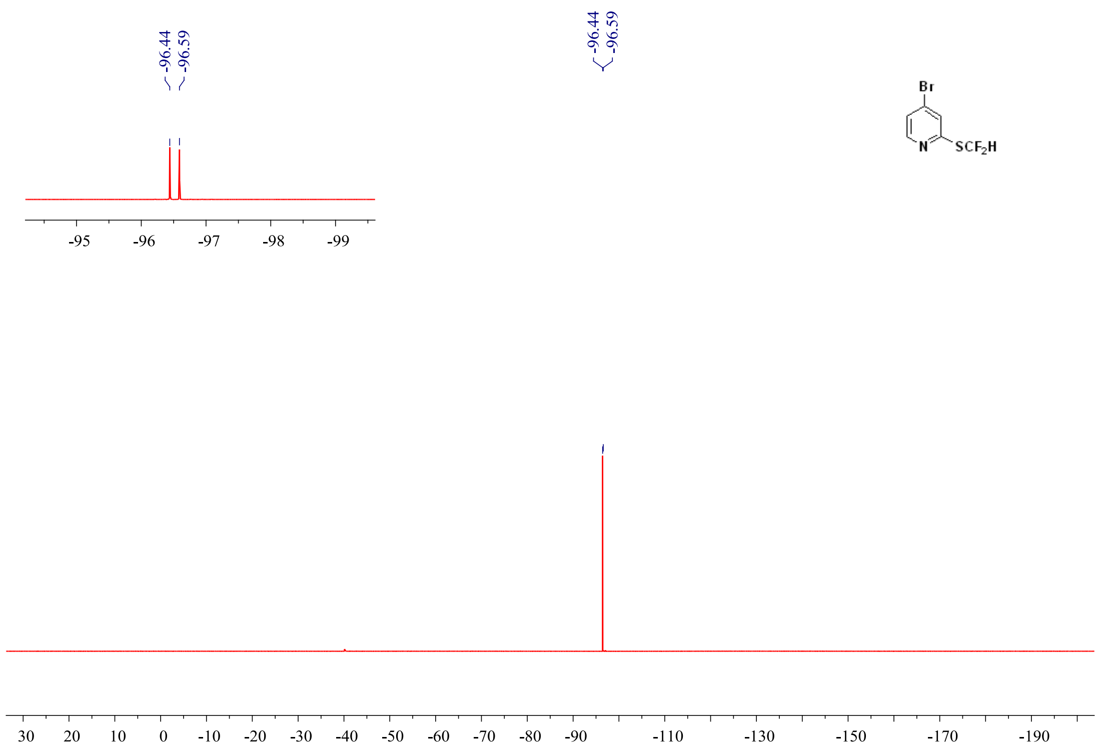

^{13}C NMR (101 MHz, CDCl$_3$) 4-bromo-2-((difluoromethyl)thio)pyridine 4ao

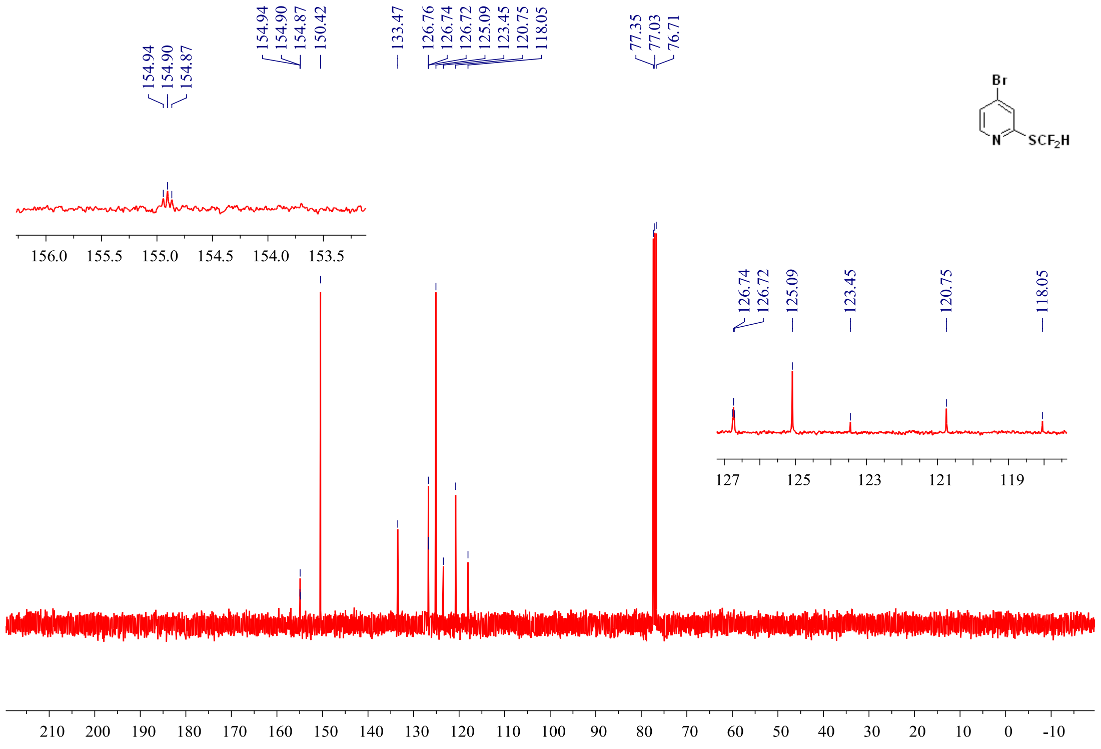

^{1}H NMR (400 MHz, CDCl$_3$) 2-((difluoromethyl)thio)quinolone 4ap

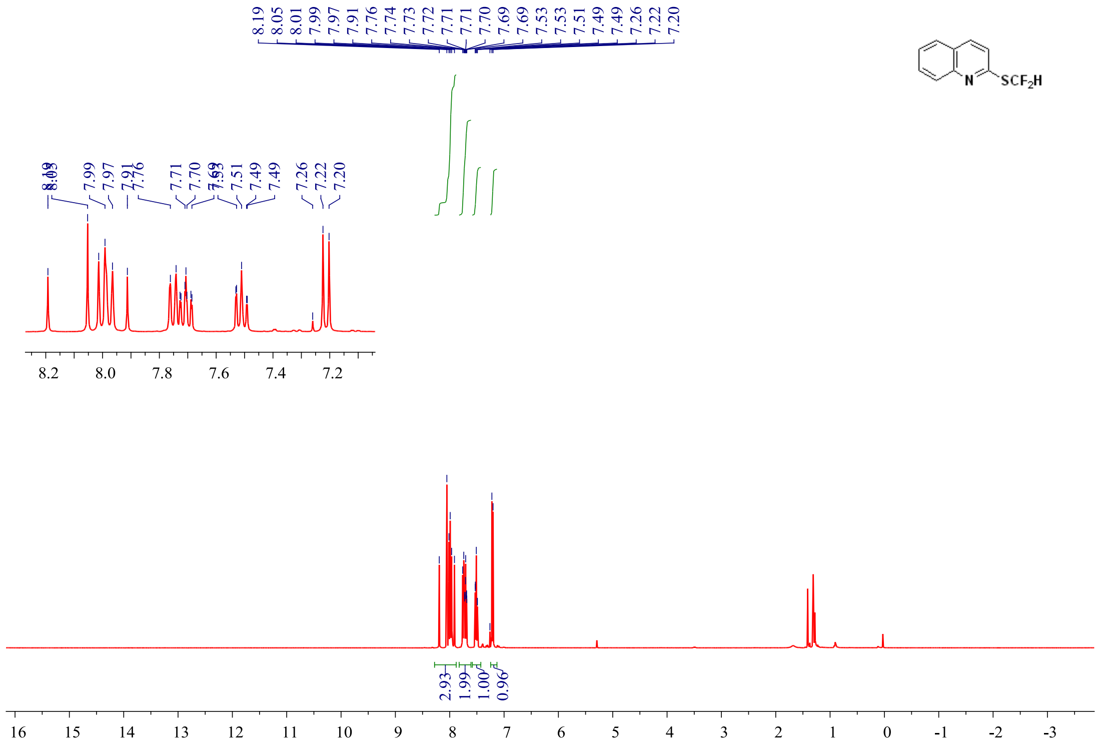

^{19}F NMR (376 MHz, $CDCl_3$) 2-((difluoromethyl)thio)quinolone 4ap

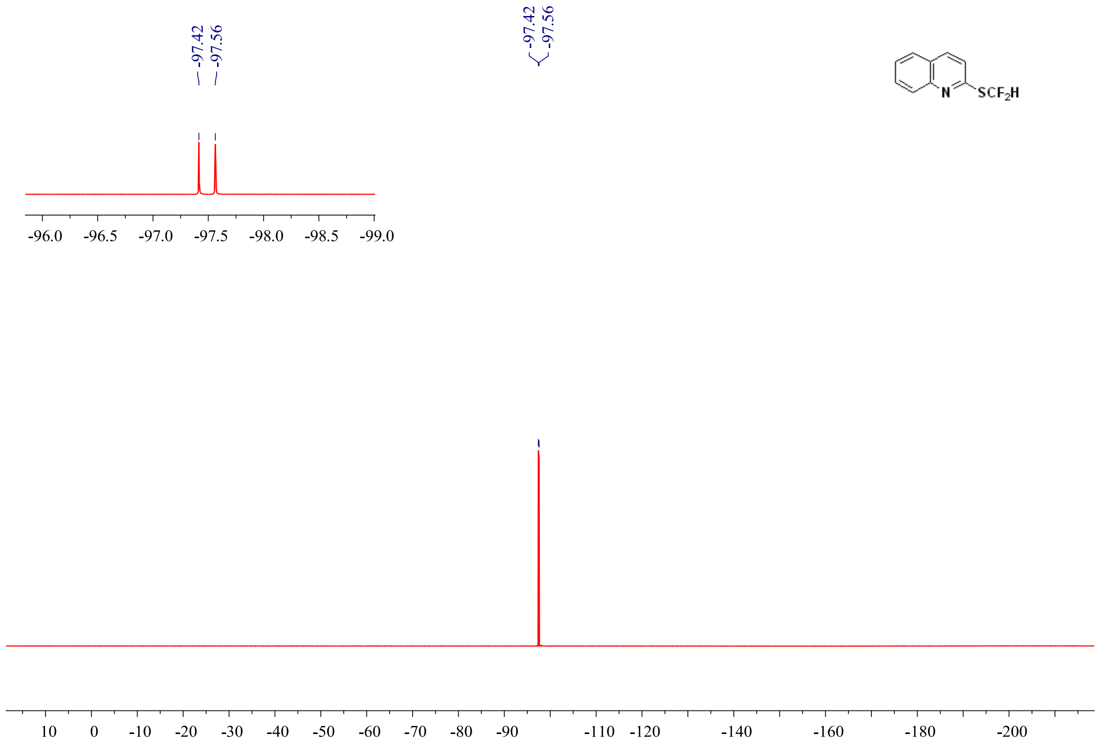

^{13}C NMR (101 MHz, $CDCl_3$) 2-((difluoromethyl)thio)quinolone 4ap

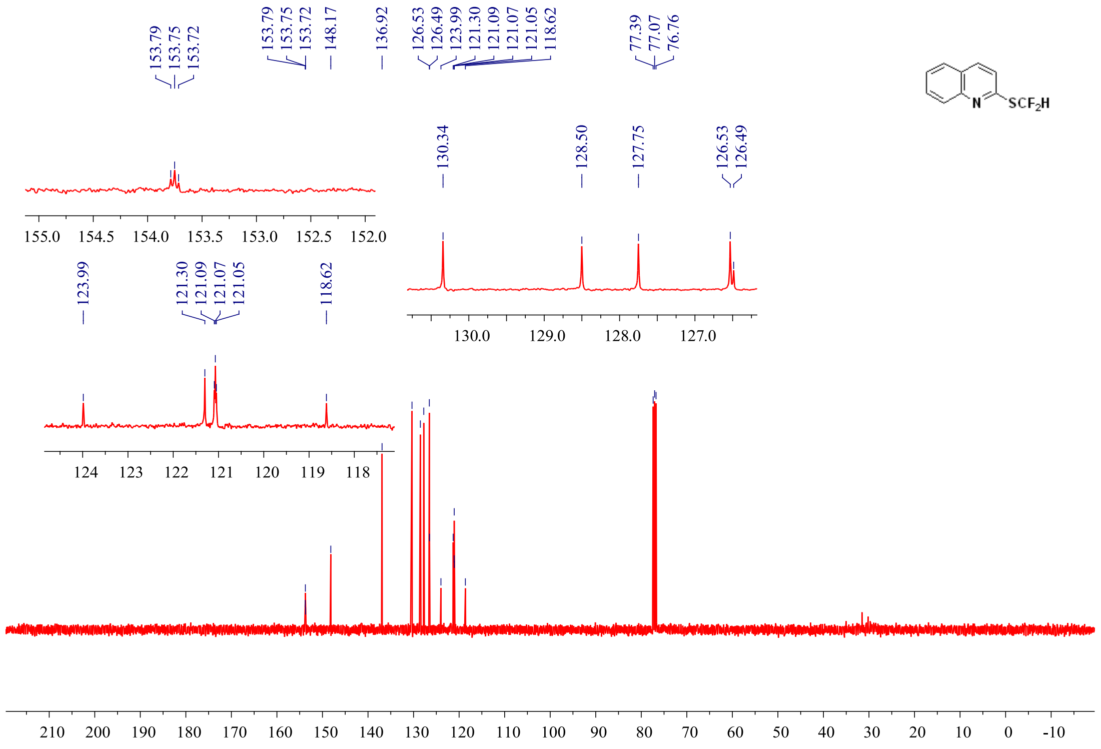

^{1}H NMR (400 MHz, $CDCl_3$) 6-((difluoromethyl)thio)pyrazine-2-carbonitrile 4aq

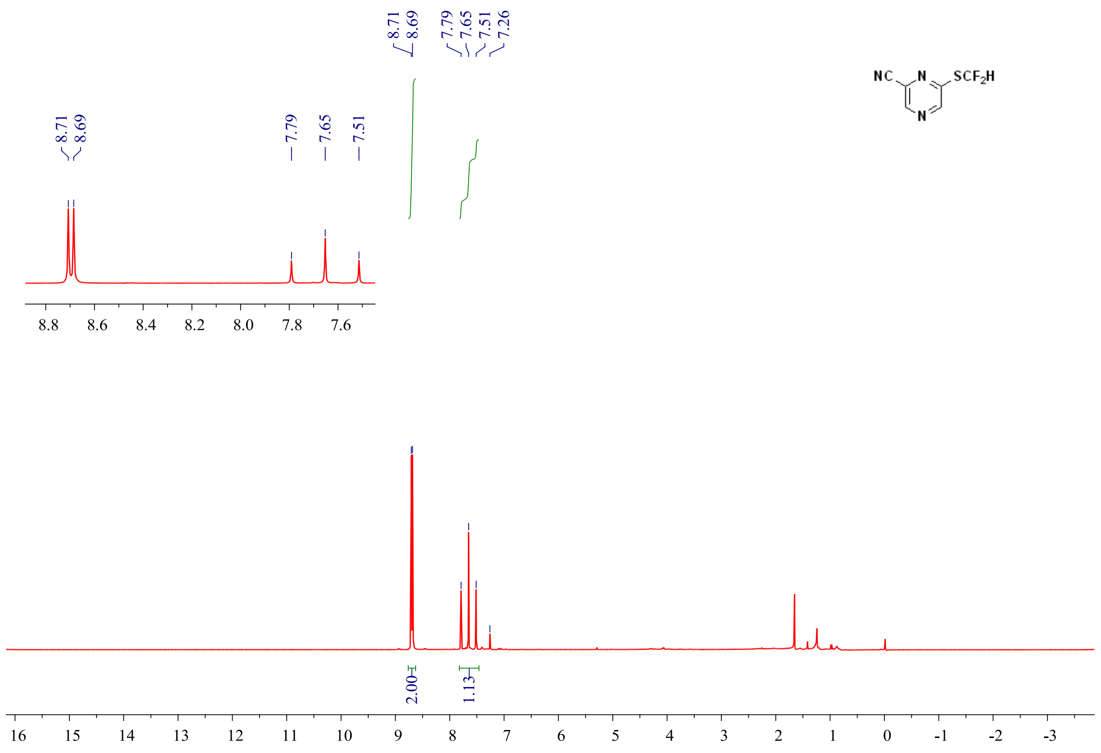

^{19}F NMR (376 MHz, $CDCl_3$) 6-((difluoromethyl)thio)pyrazine-2-carbonitrile 4aq

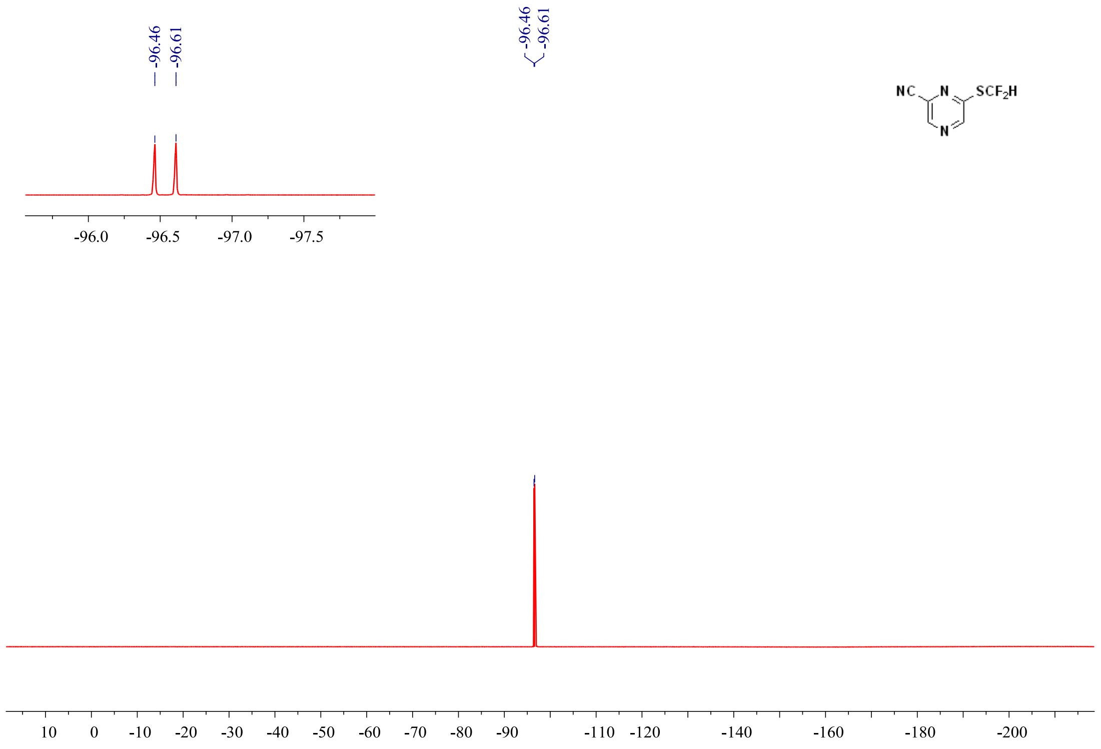

^{13}C NMR (101 MHz, $CDCl_3$) 6-((difluoromethyl)thio)pyrazine-2-carbonitrile 4aq

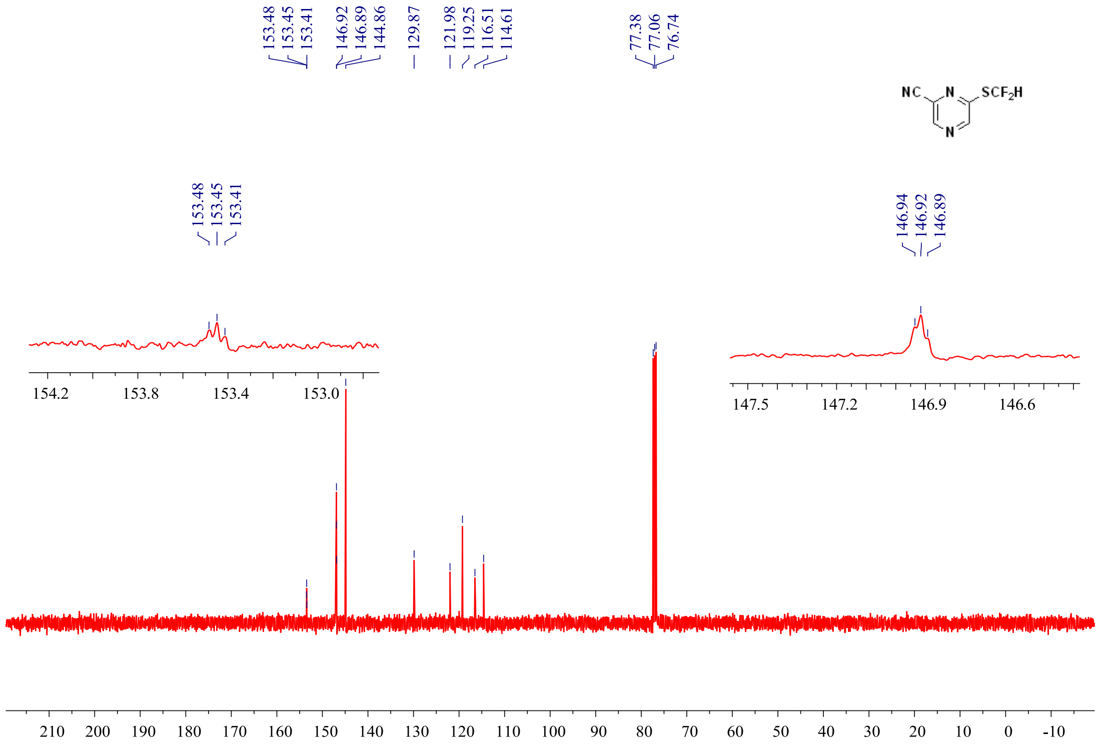

^{1}H NMR (400 MHz, $CDCl_3$) 2-chloro-5-((difluoromethyl)thio)pyrazine 4ar

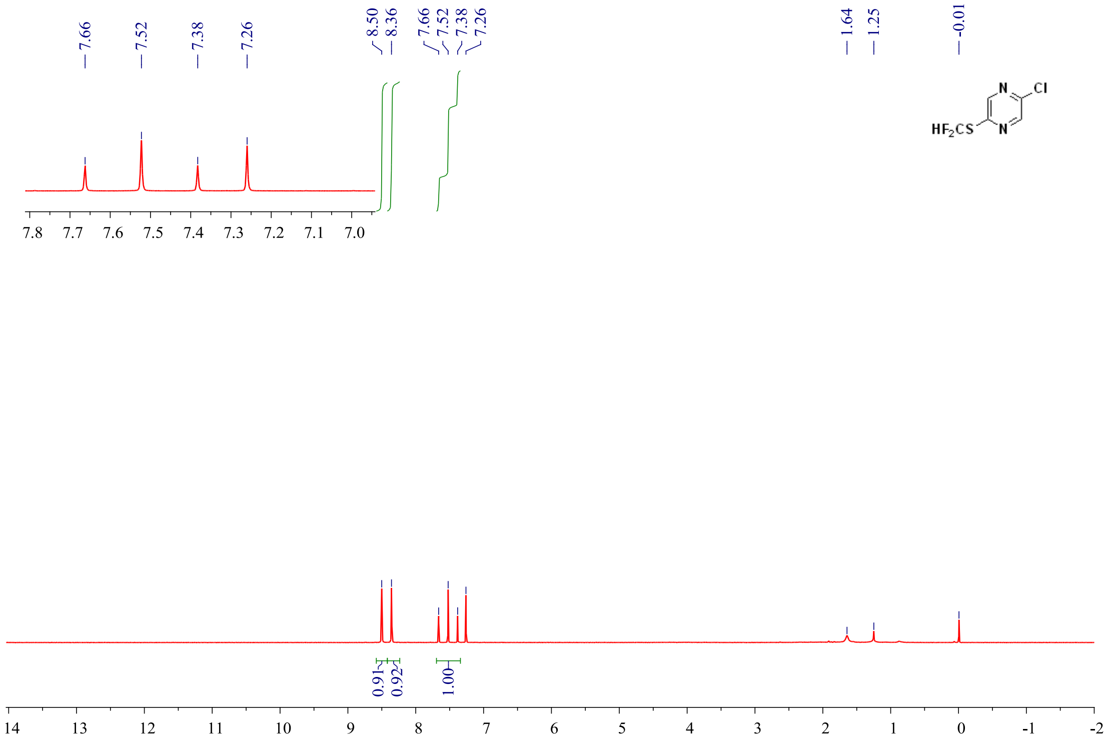

^{19}F NMR (376 MHz, $CDCl_3$) 2-chloro-5-((difluoromethyl)thio)pyrazine 4ar

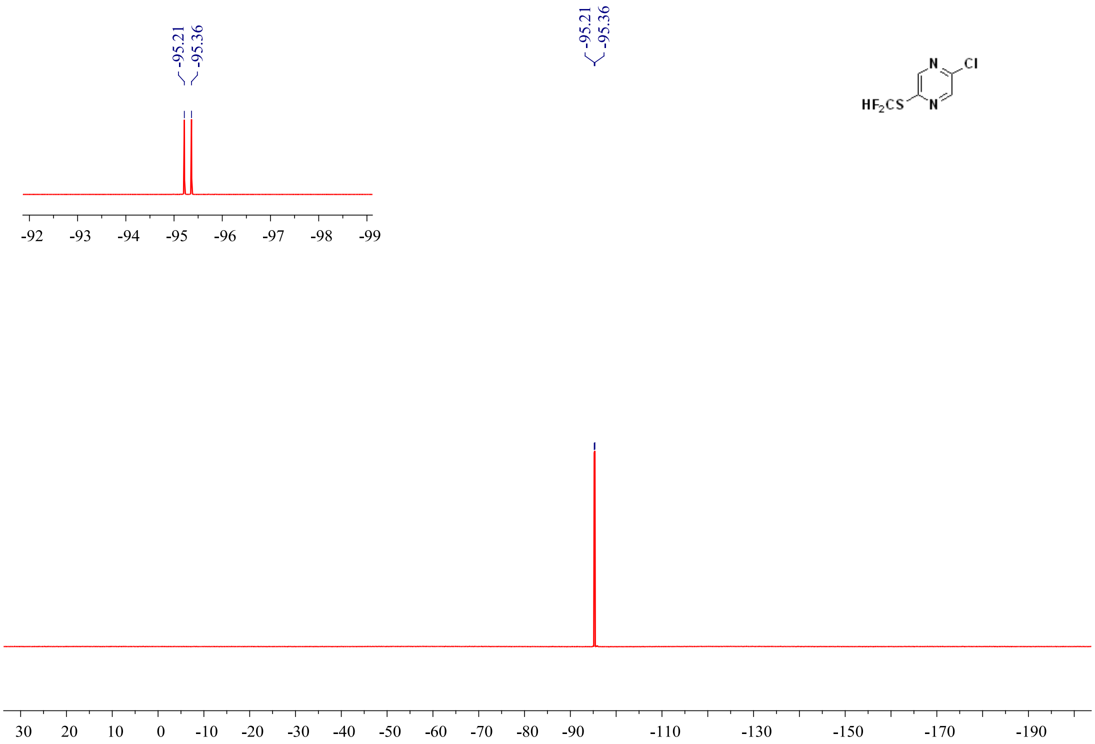

^{13}C NMR (101 MHz, $CDCl_3$) 2-chloro-5-((difluoromethyl)thio)pyrazine 4ar

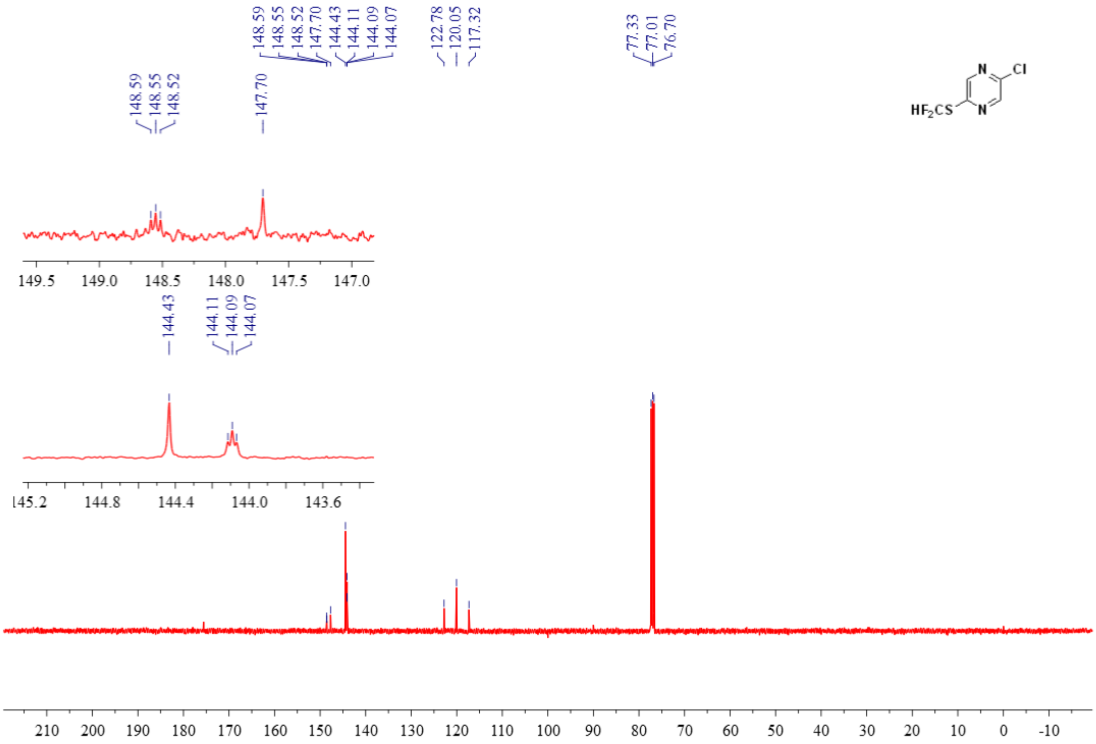

^{1}H NMR (400 MHz, $CDCl_3$) 2-((difluoromethyl)thio)-5-(methylthio)pyrazine 4as

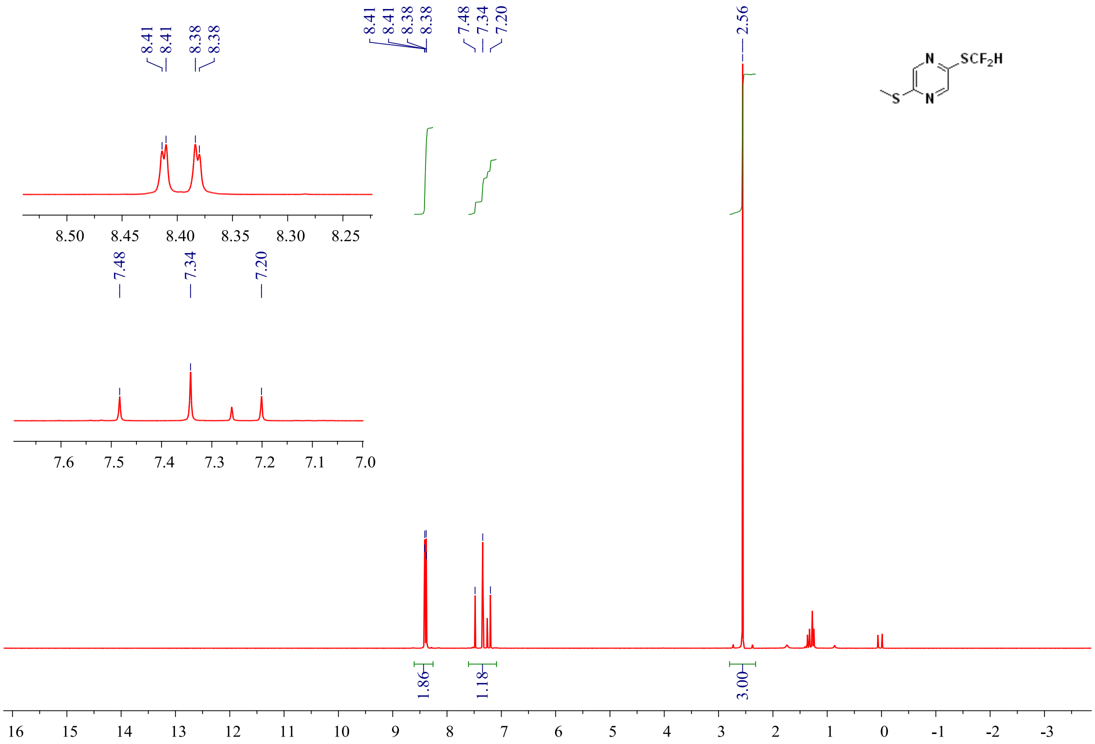

^{19}F NMR (376 MHz, $CDCl_3$) 2-((difluoromethyl)thio)-5-(methylthio)pyrazine 4as

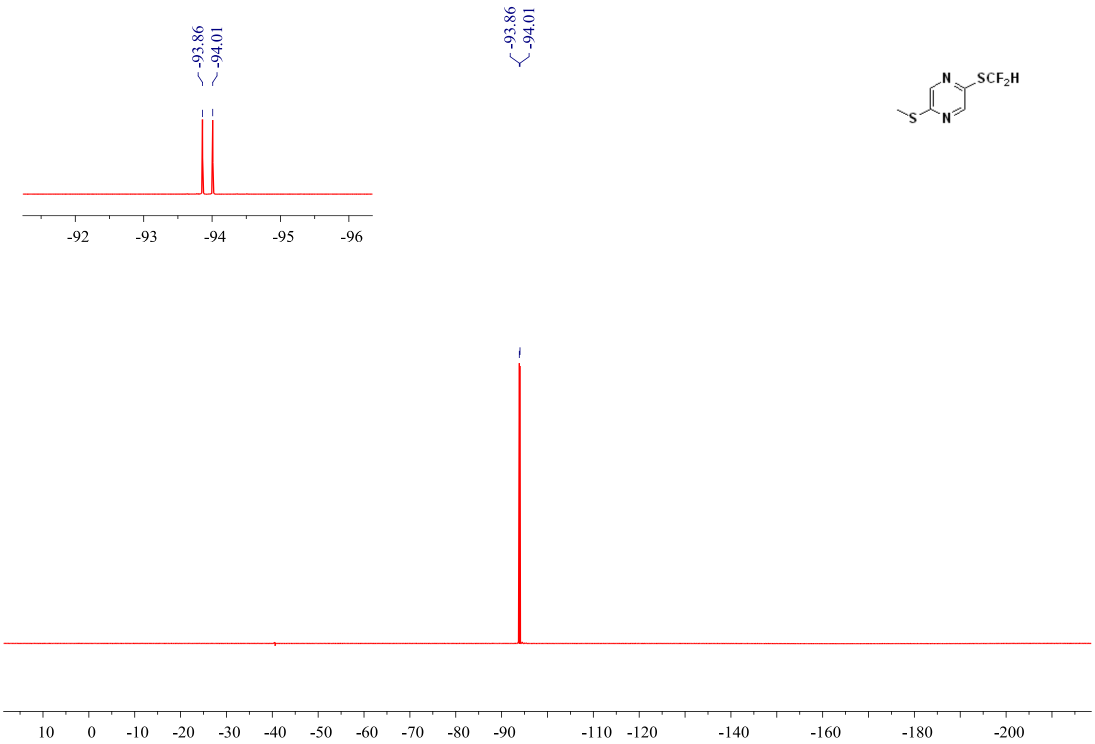

^{13}C NMR (101 MHz, $CDCl_3$) 2-((difluoromethyl)thio)-5-(methylthio)pyrazine 4as

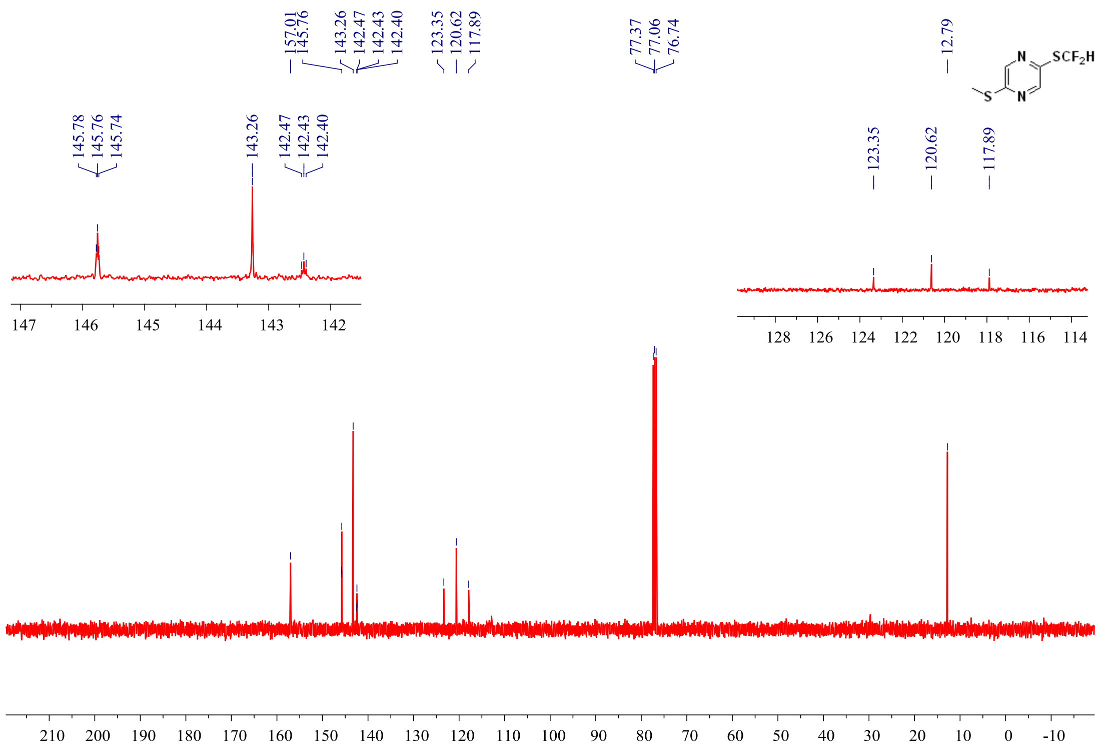

^{1}H NMR (400 MHz, $CDCl_3$) 2-((difluoromethyl)thio)quinoxaline 4at

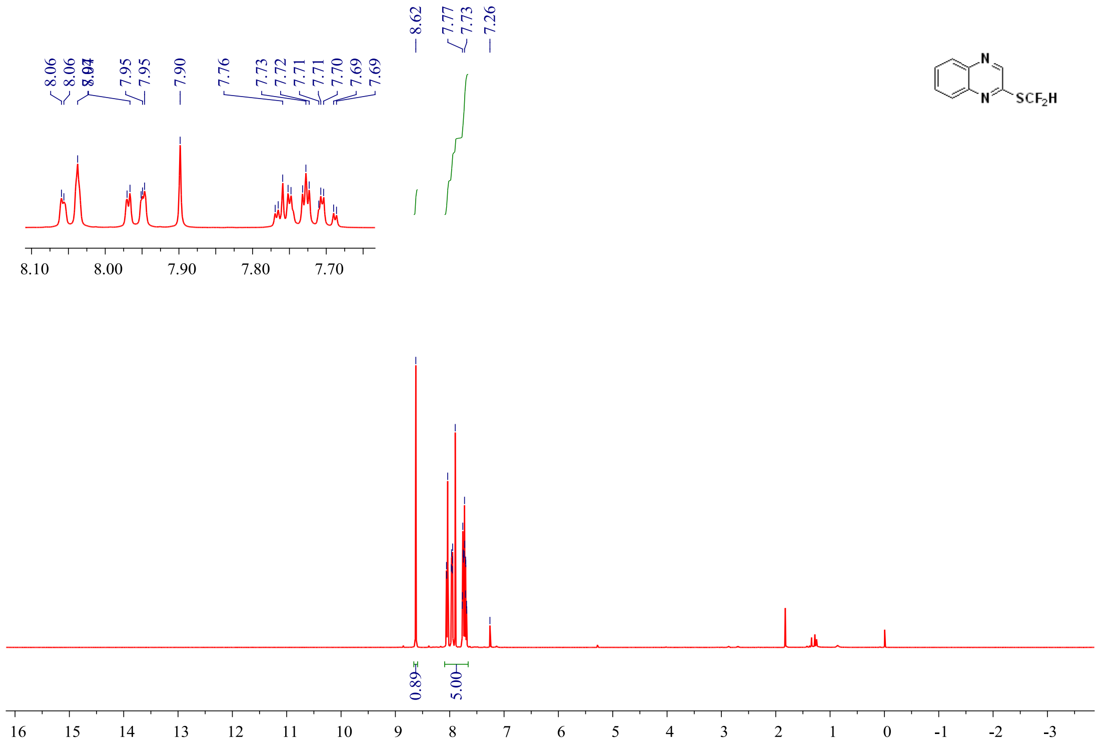

^{19}F NMR (376 MHz, $CDCl_3$) 2-((difluoromethyl)thio)quinoxaline 4at

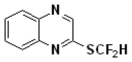

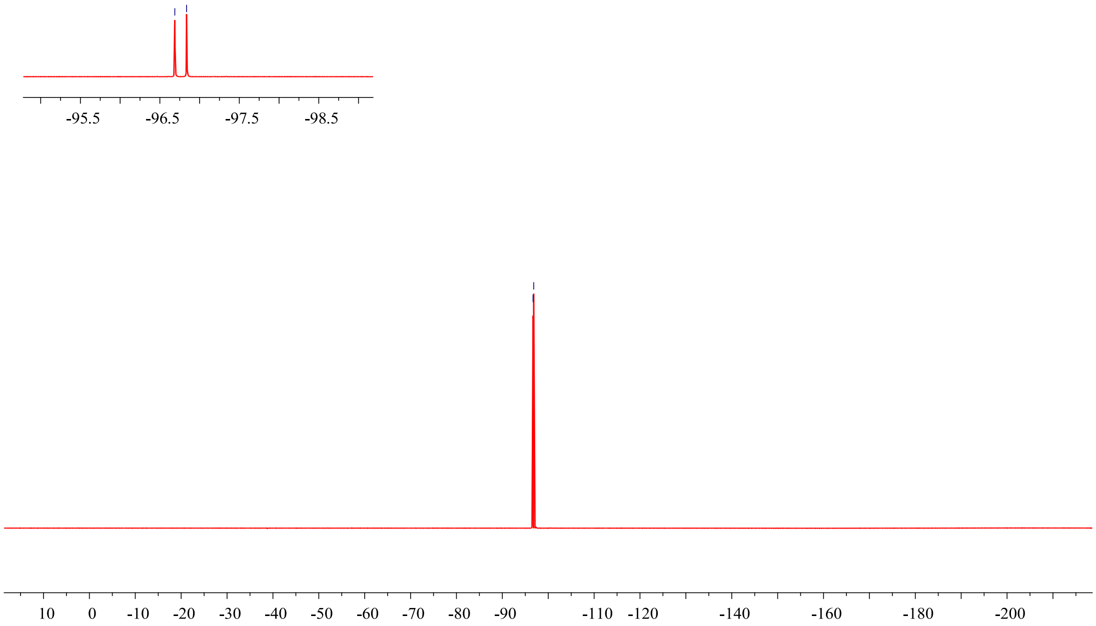

^{13}C NMR (101 MHz, $CDCl_3$) 2-((difluoromethyl)thio)quinoxaline 4at

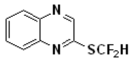

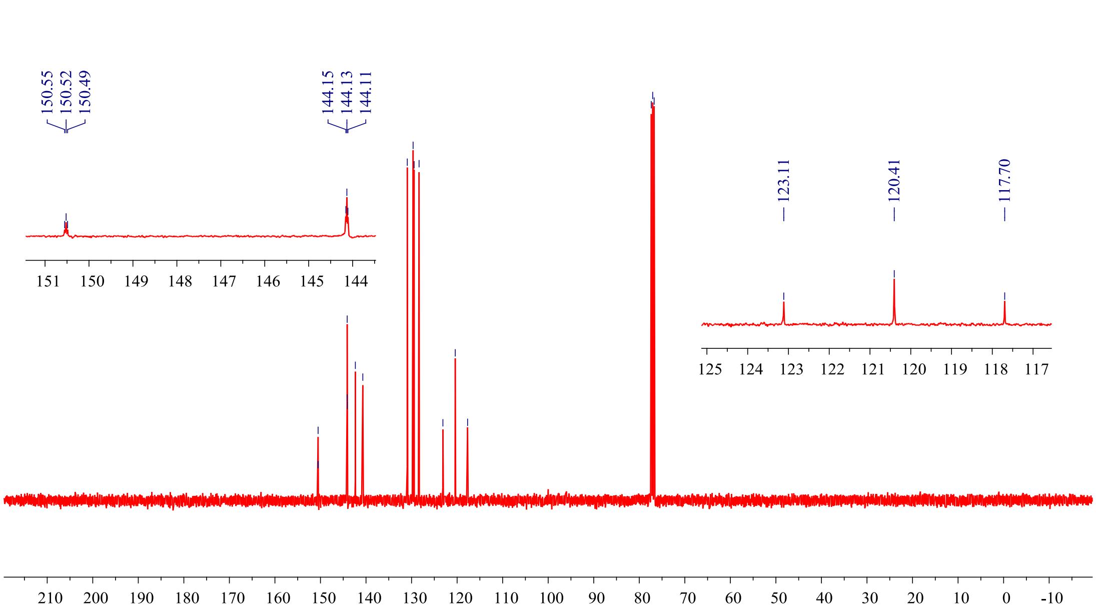

^{1}H NMR (400 MHz, $CDCl_3$) 6-((difluoromethyl)thio)-2,2'-bipyridine 4u

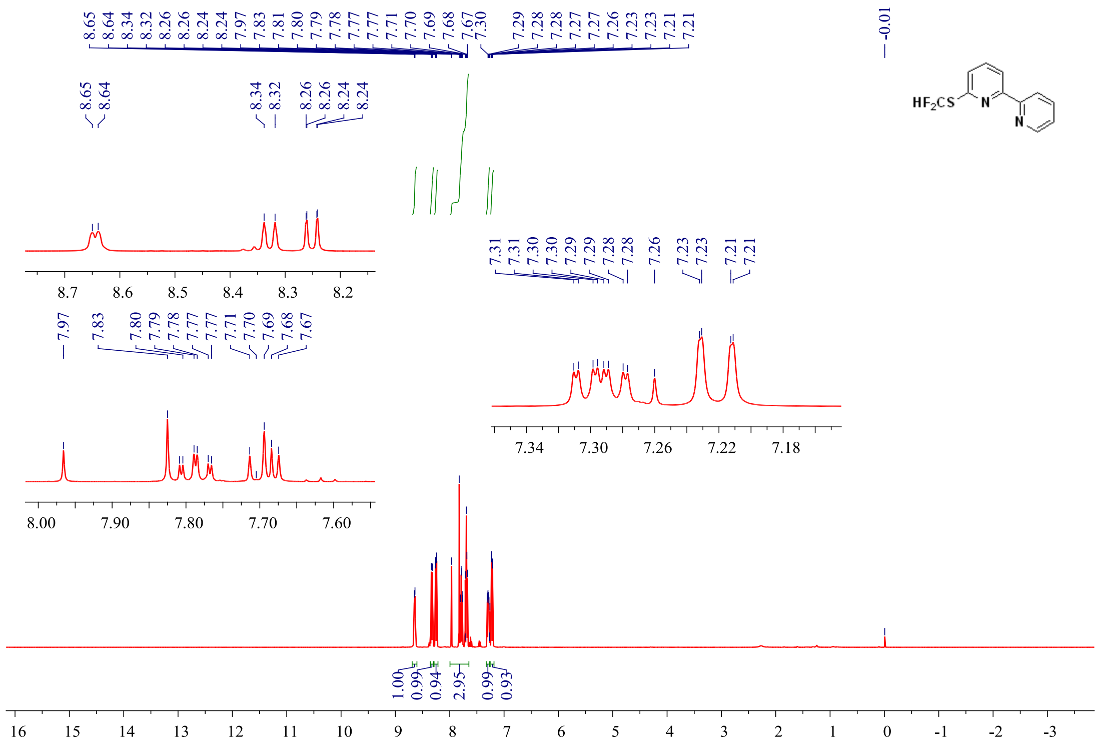

^{19}F NMR (376 MHz, $CDCl_3$) 6-((difluoromethyl)thio)-2,2'-bipyridine 4u

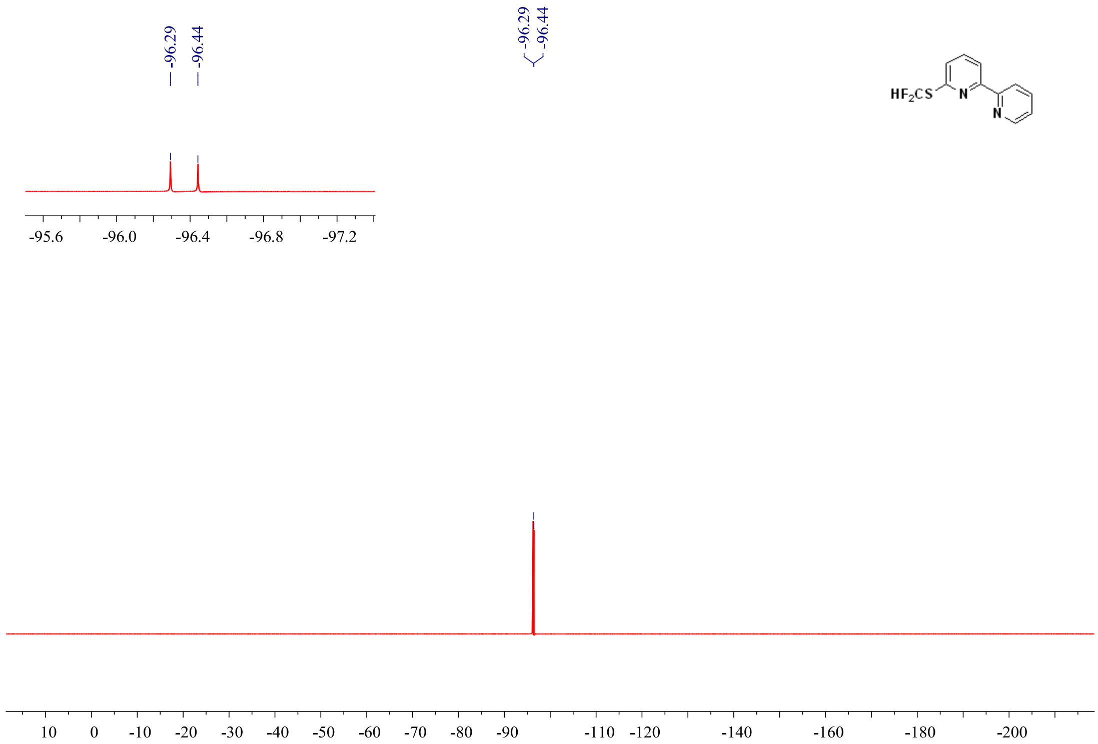

^{13}C NMR (101 MHz, $CDCl_3$) 6-((difluoromethyl)thio)-2,2'-bipyridine 4u

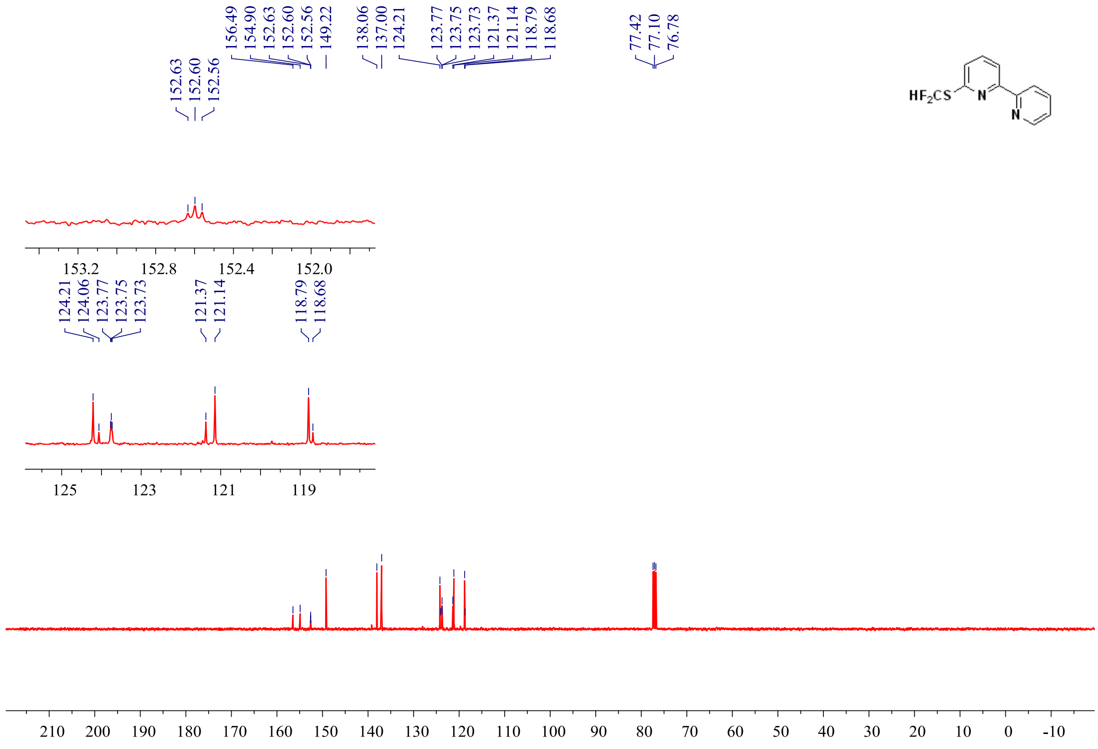

^{1}H NMR (400 MHz, $CDCl_3$) 5-bromo-2-((difluoromethyl)thio) -3-nitropyridine 4av

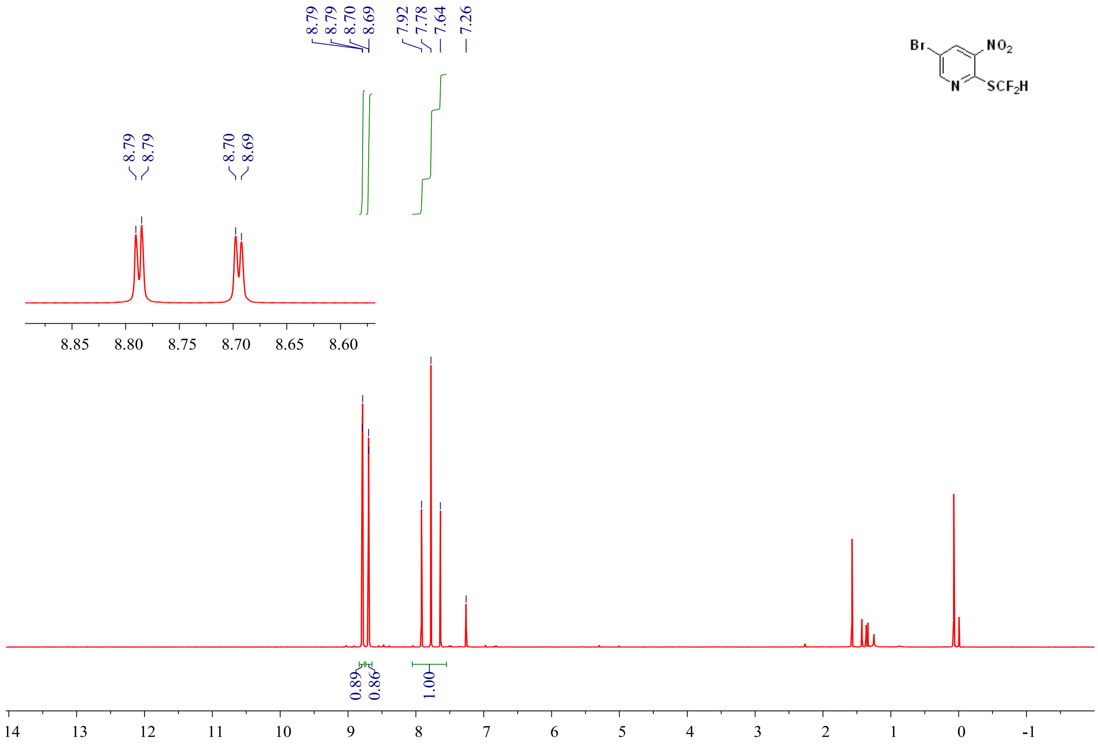

^{19}F NMR (376 MHz, $CDCl_3$) 5-bromo-2-((difluoromethyl)thio) -3-nitropyridine 4av

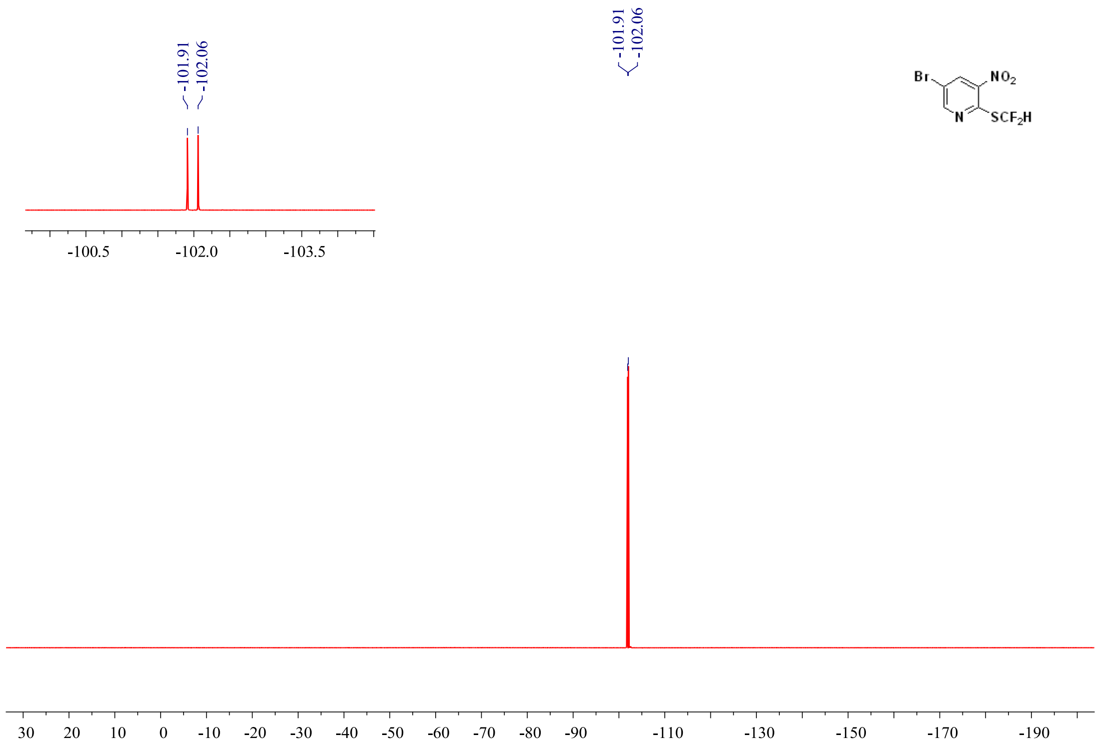

^{13}C NMR (101 MHz, $CDCl_3$) 5-bromo-2-((difluoromethyl)thio) -3-nitropyridine 4av

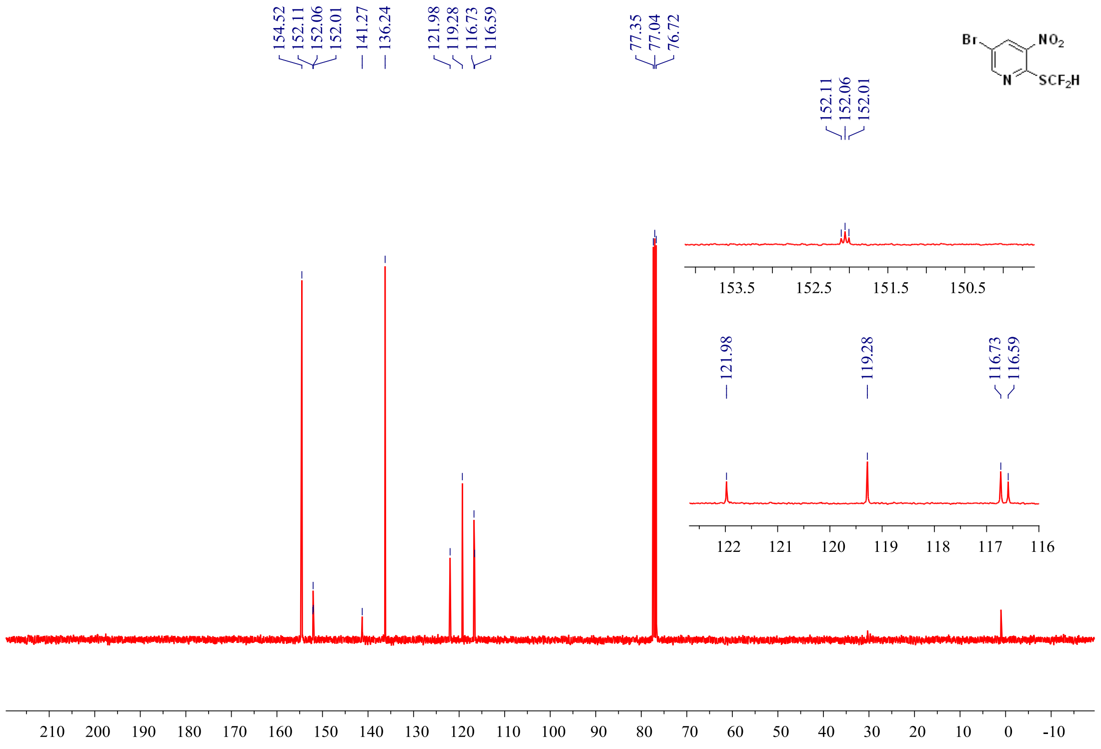

^{1}H NMR (400 MHz, $CDCl_3$) 7-bromo-1-((difluoromethyl)thio)isoquinoline 4aw

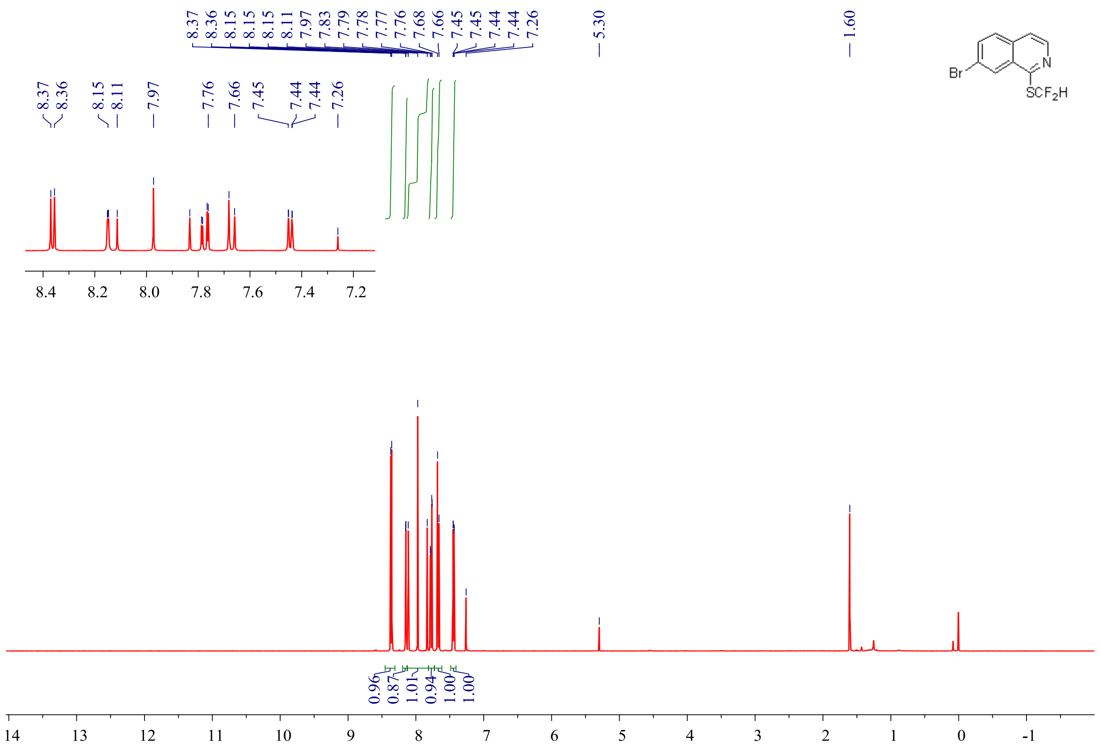

^{19}F NMR (376 MHz, $CDCl_3$) 7-bromo-1-((difluoromethyl)thio)isoquinoline 4aw

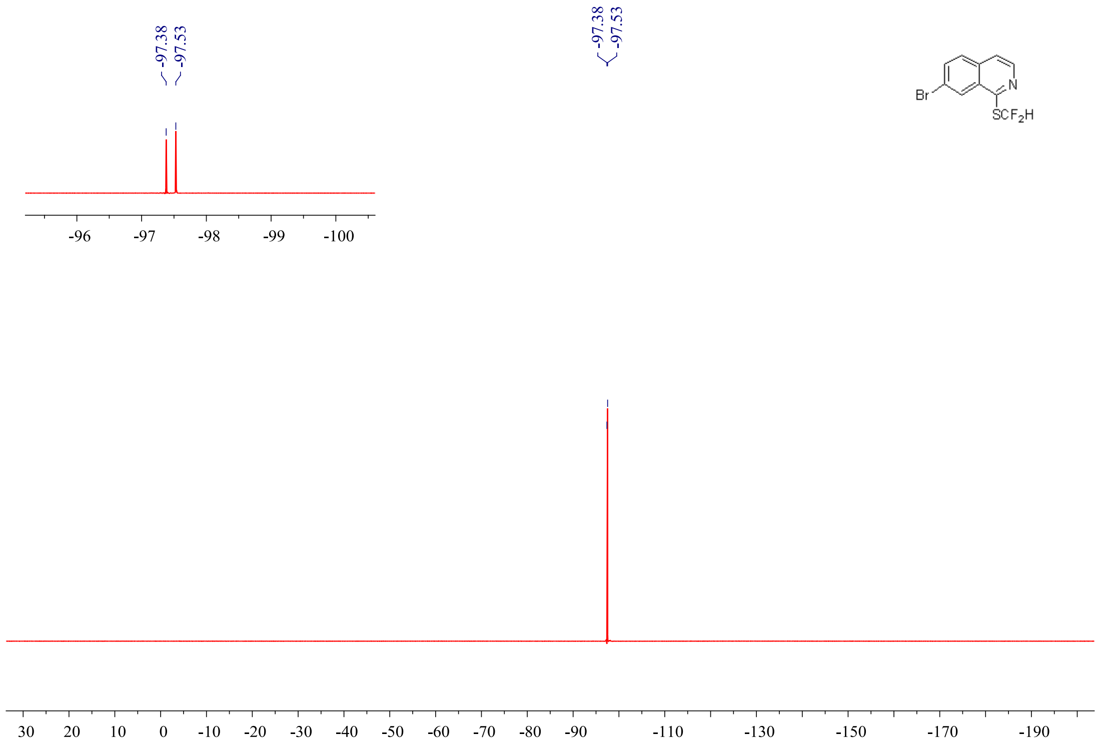

^{13}C NMR (101 MHz, $CDCl_3$) 7-bromo-1-((difluoromethyl)thio)isoquinoline 4aw

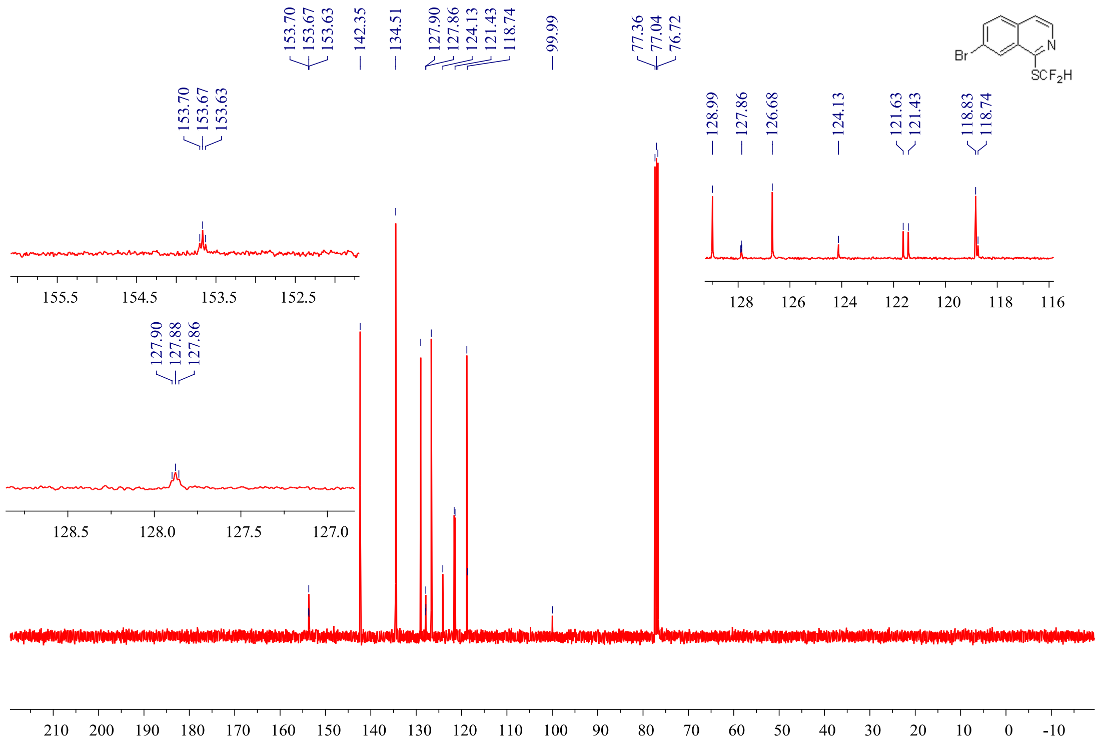

^{1}H NMR (400 MHz, $CDCl_3$) [1,1'-biphenyl]-4-yl(difluoromethyl)sulfane 5a

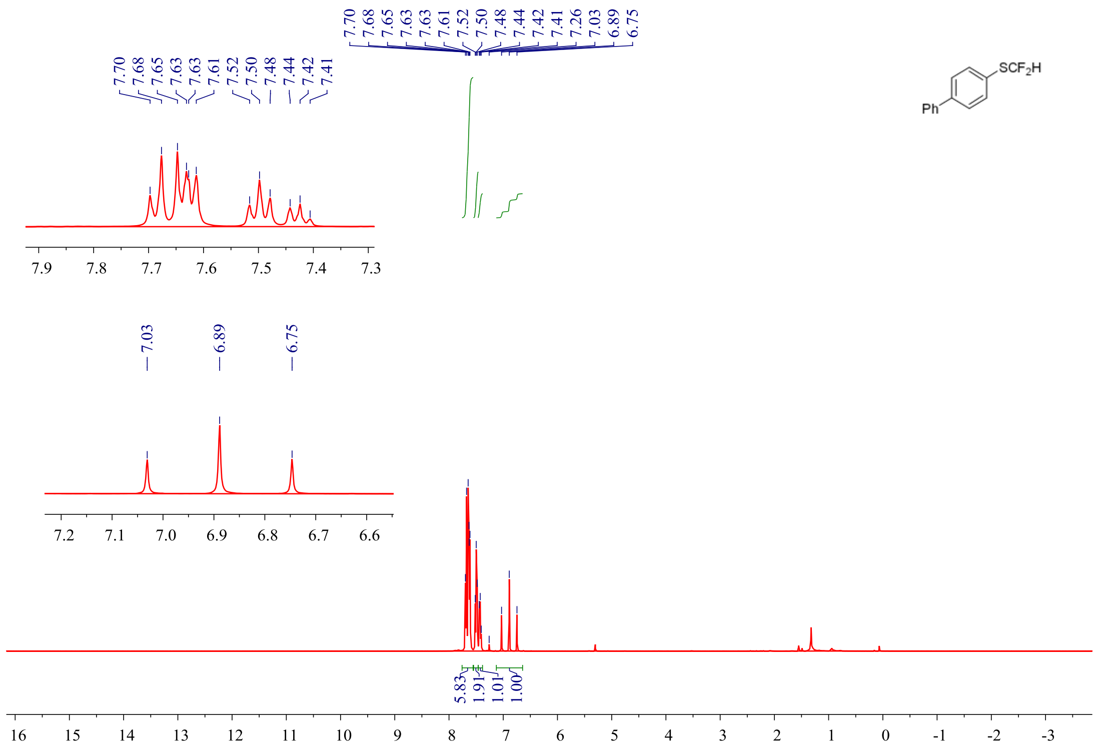

^{19}F NMR (376 MHz, $CDCl_3$) [1,1'-biphenyl]-4-yl(difluoromethyl)sulfane 5a

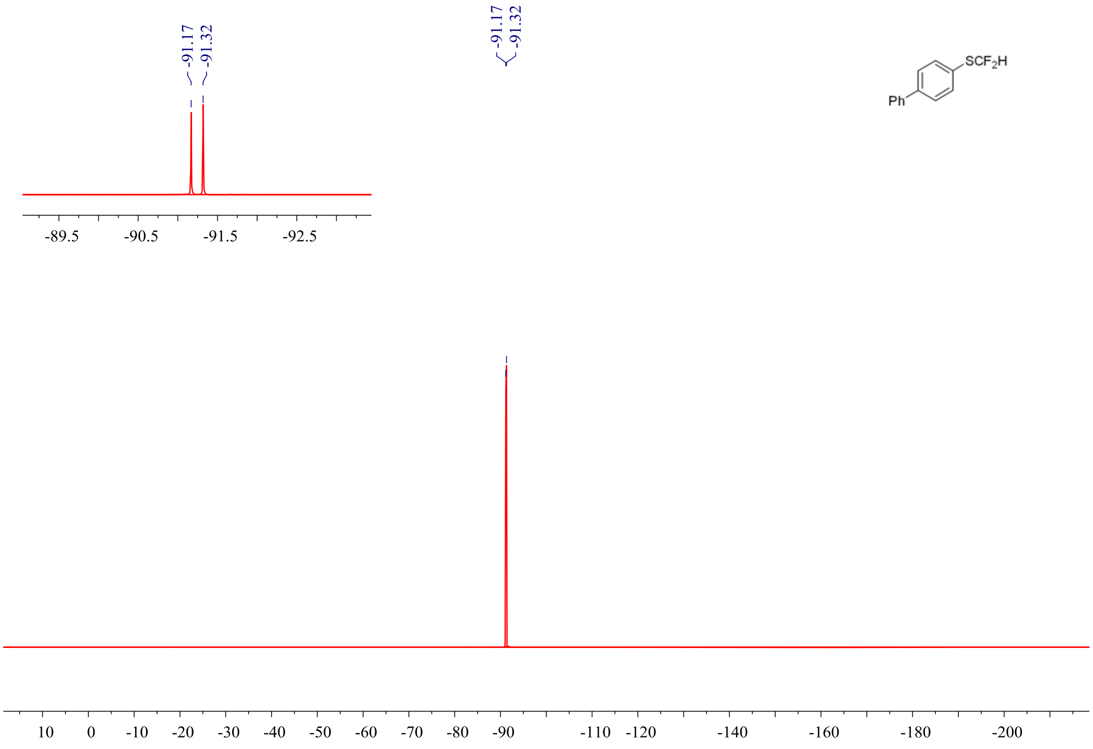

^{13}C NMR (101 MHz, $CDCl_3$) [1,1'-biphenyl]-4-yl(difluoromethyl)sulfane 5a

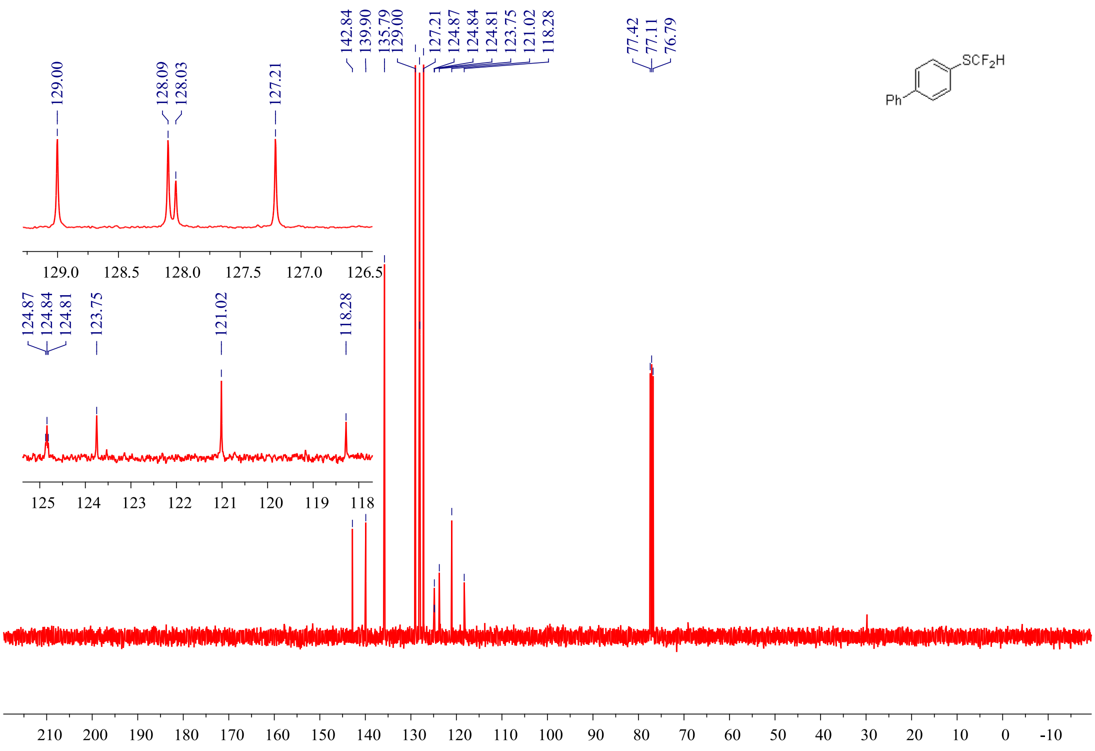

^{1}H NMR (400 MHz, $CDCl_3$) (4-(*tert*-butyl)phenyl)(difluoromethyl)sulfane 5b

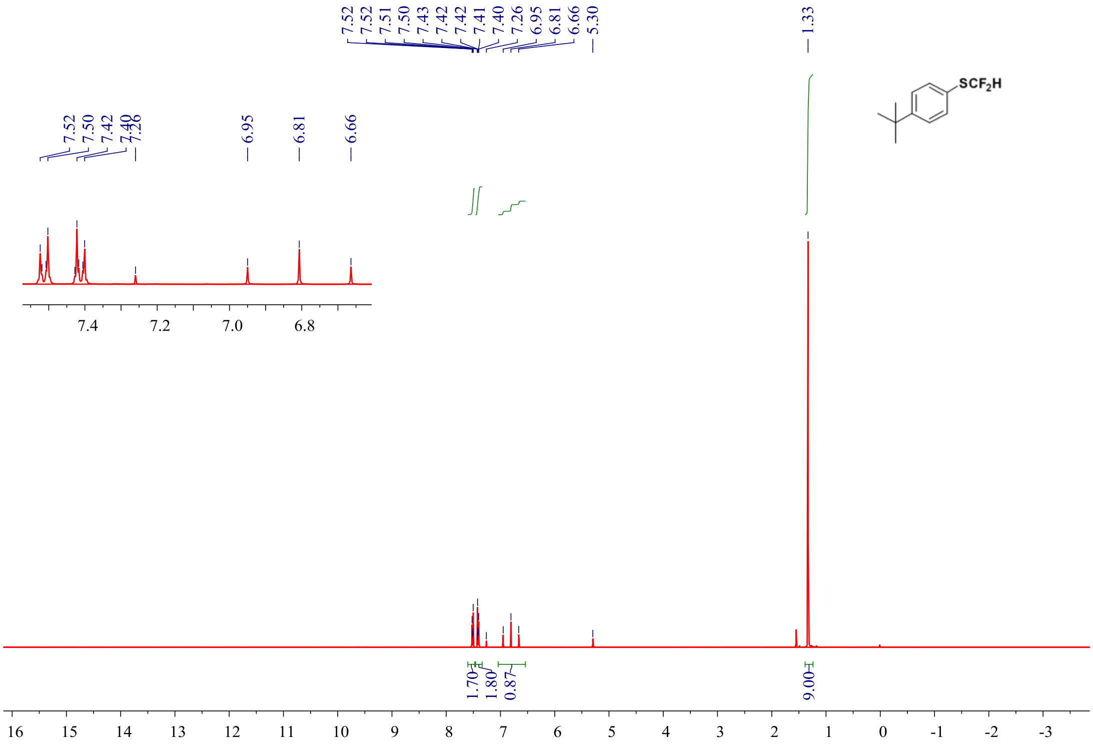

^{19}F NMR (376 MHz, $CDCl_3$) (4-(*tert*-butyl)phenyl)(difluoromethyl)sulfane 5b

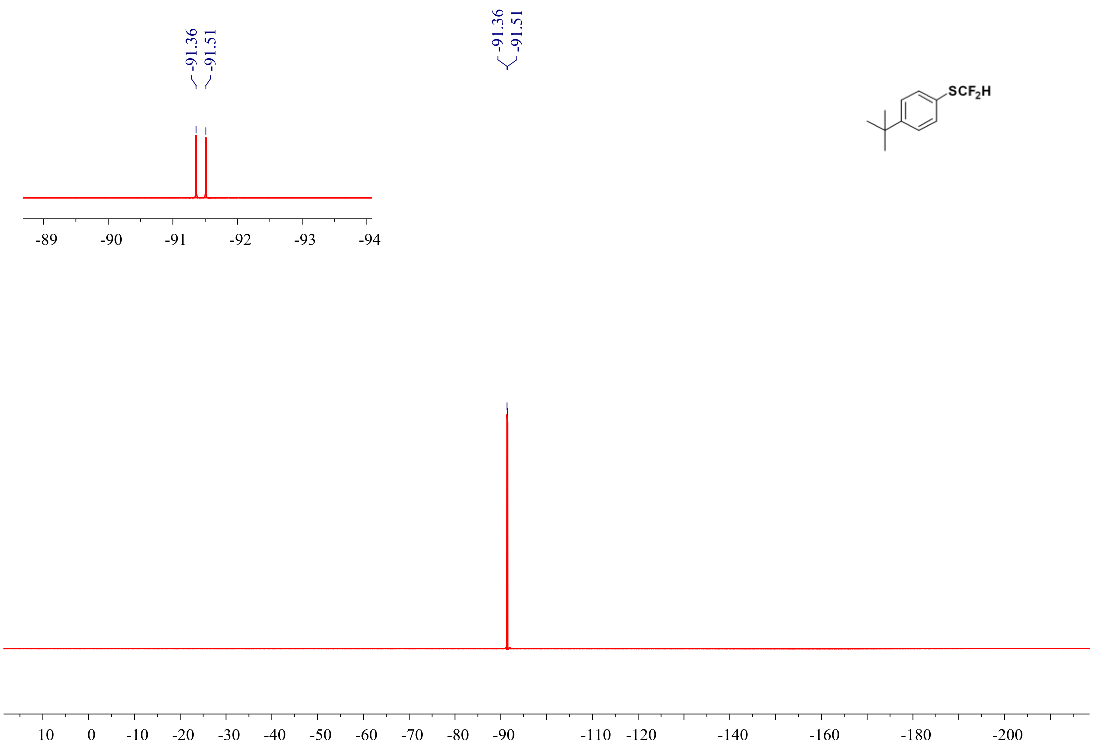

^{13}C NMR (101 MHz, $CDCl_3$) (4-(*tert*-butyl)phenyl)(difluoromethyl)sulfane 5b

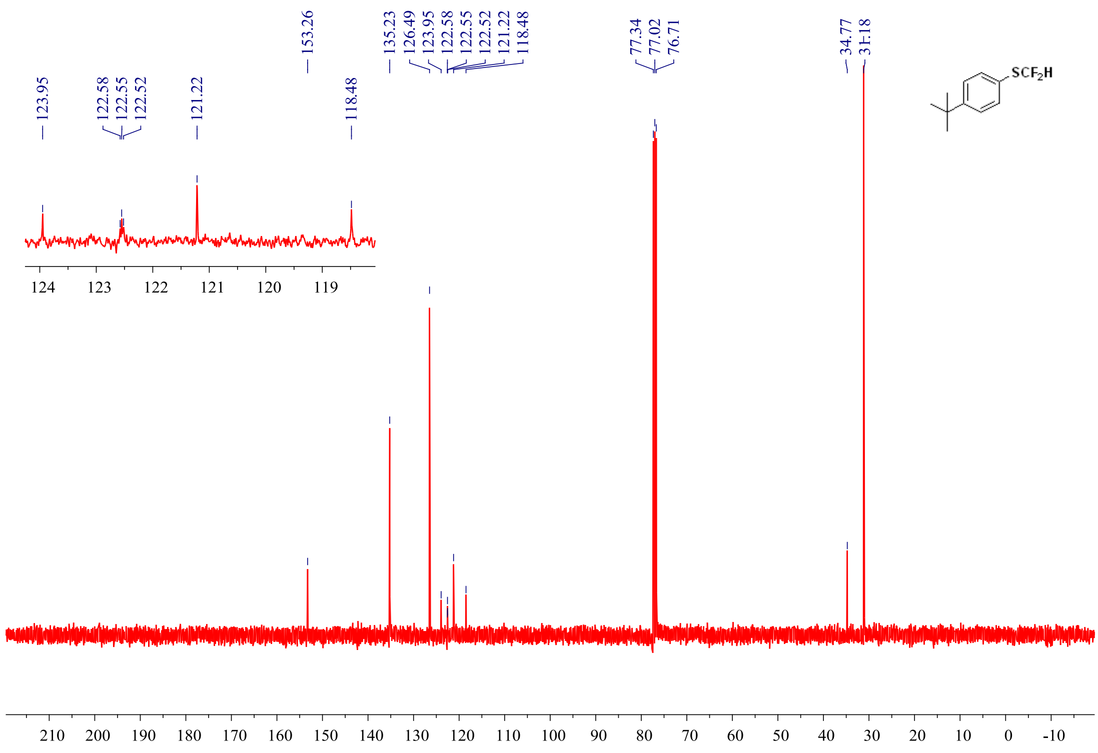

^{1}H NMR (400 MHz, $CDCl_3$) [1,1'-biphenyl]-2-yl(difluoromethyl)sulfane 5c

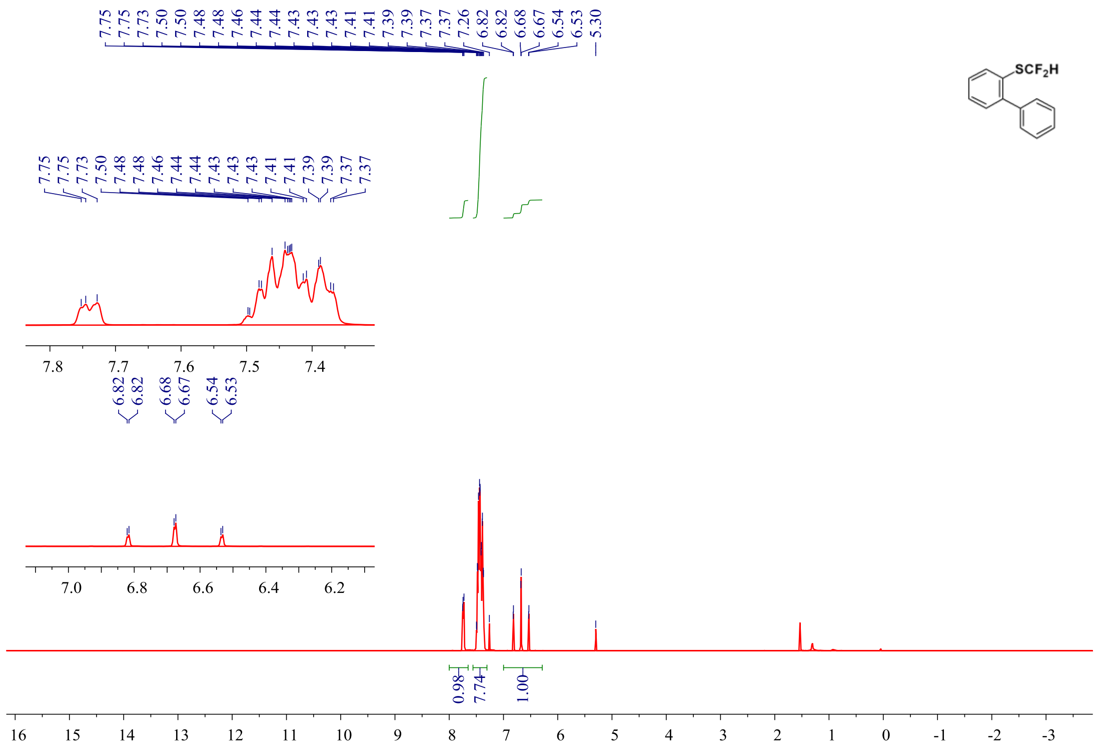

^{19}F NMR (376 MHz, $CDCl_3$) [1,1'-biphenyl]-2-yl(difluoromethyl)sulfane 5c

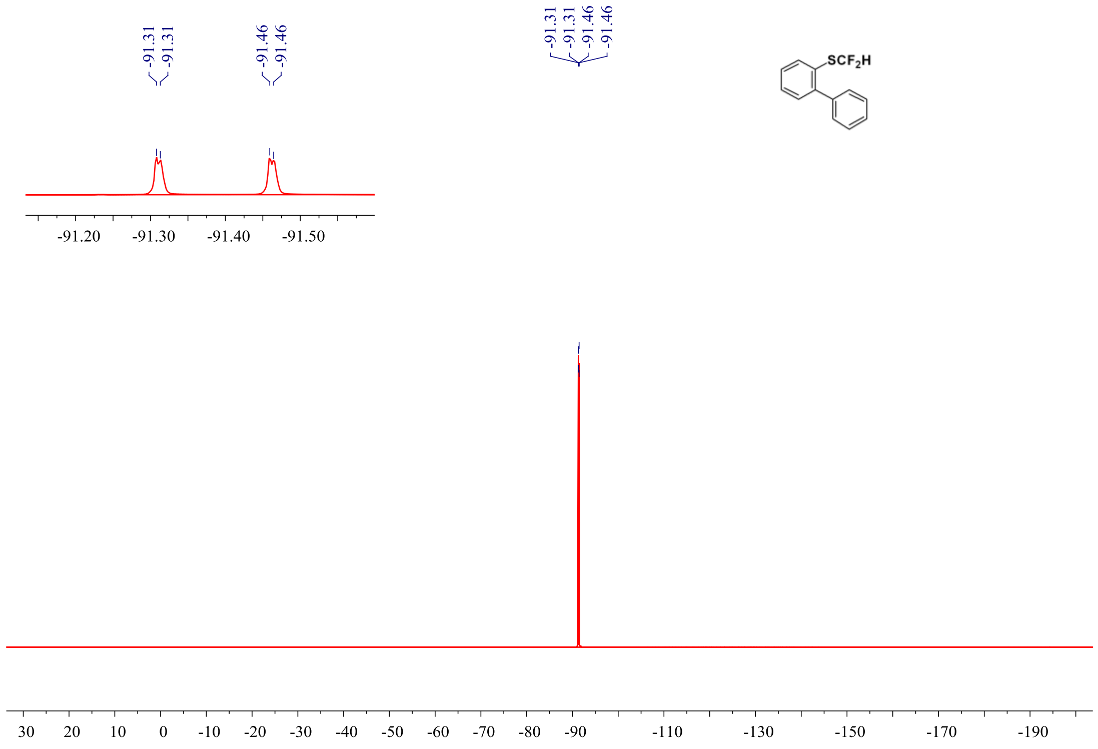

^{13}C NMR (101 MHz, $CDCl_3$) [1,1'-biphenyl]-2-yl(difluoromethyl)sulfane 5c

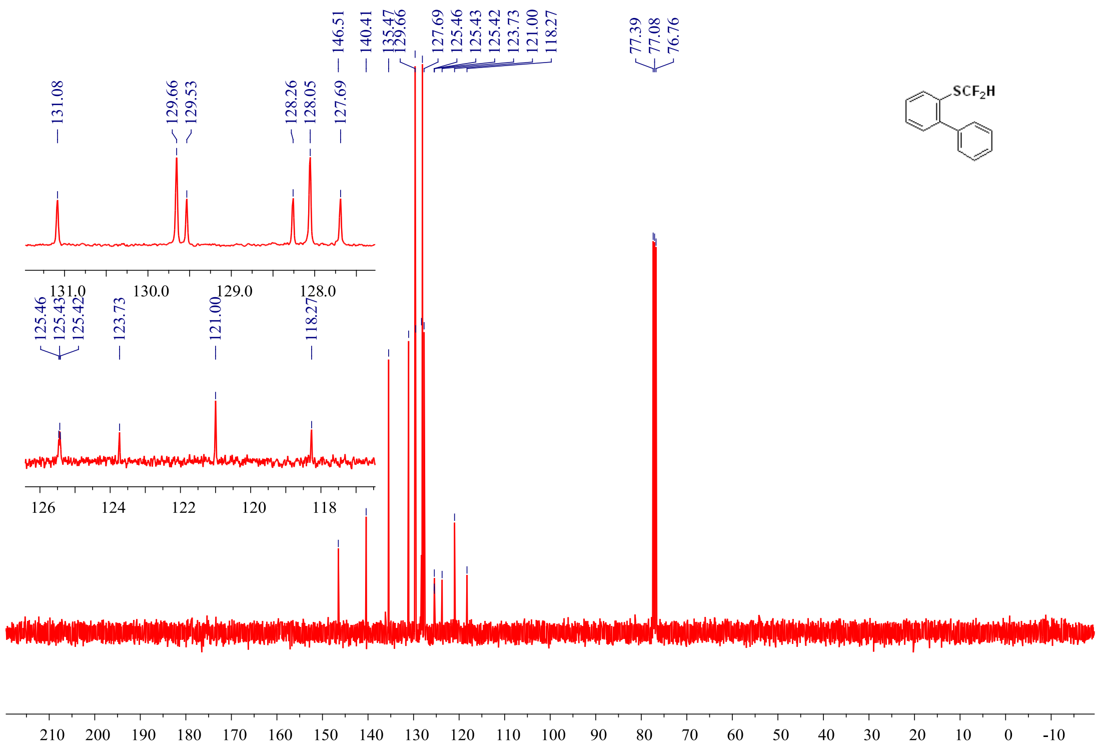

^{1}H NMR (400 MHz, $CDCl_3$) (difluoromethyl)(3,4,5-trimethoxyphenyl)sulfane 5d

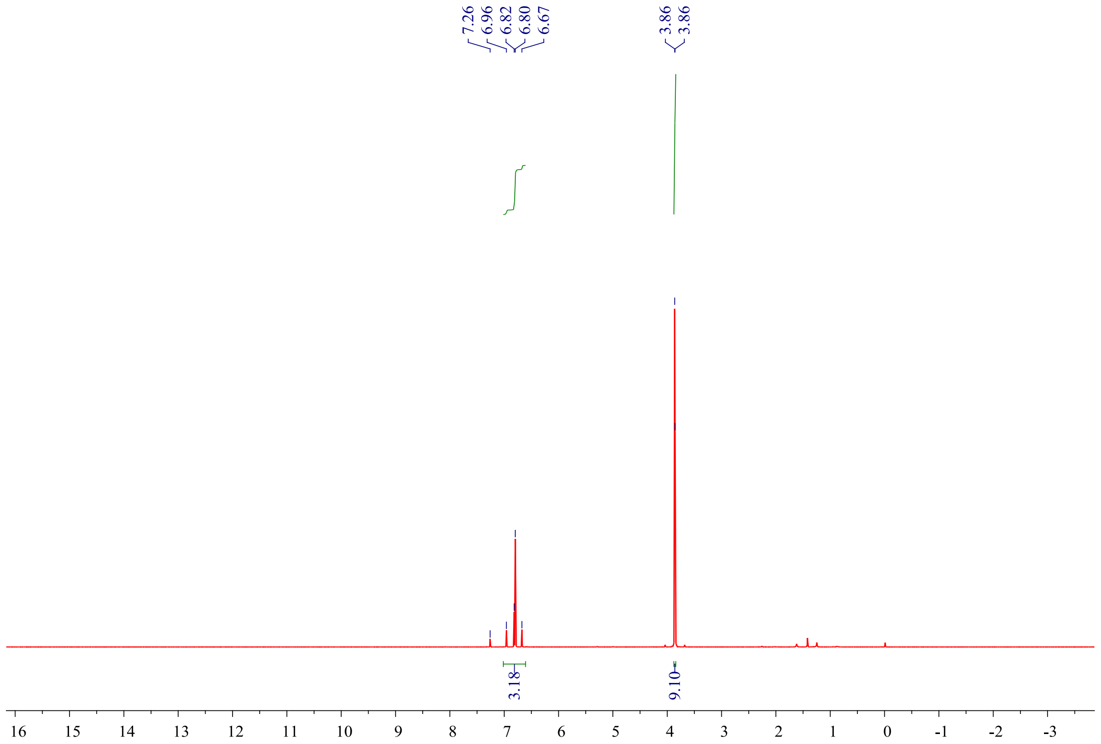

^{19}F NMR (376 MHz, $CDCl_3$) (difluoromethyl)(3,4,5-trimethoxyphenyl)sulfane 5d

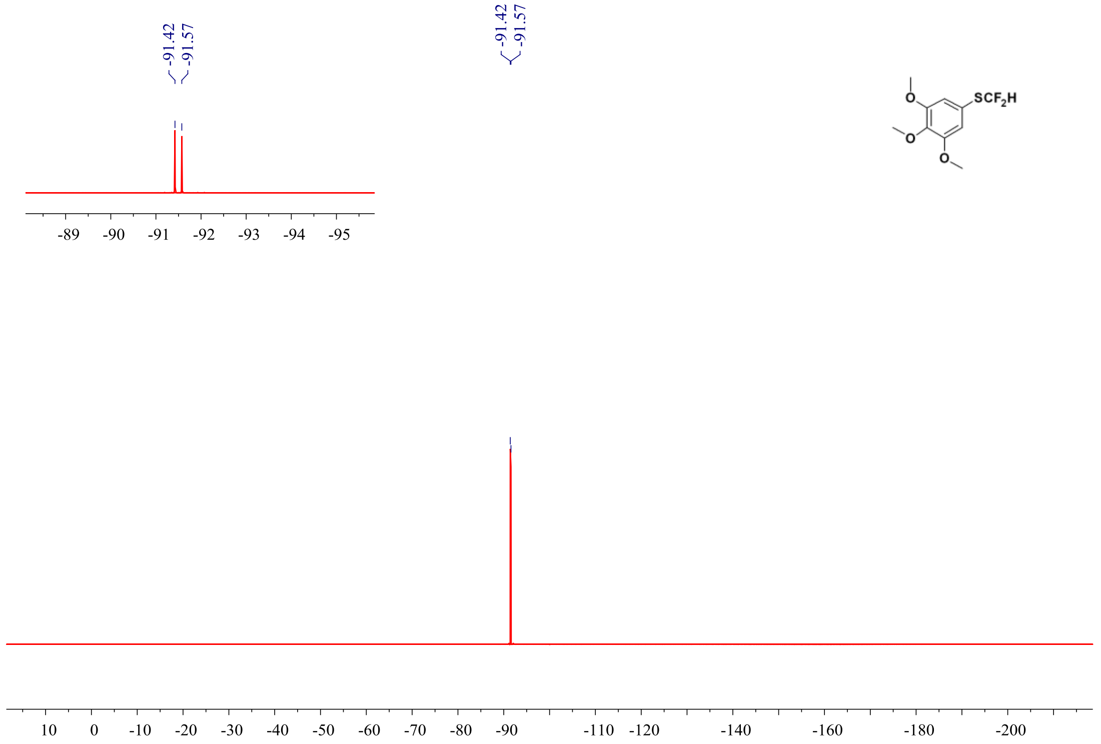

^{13}C NMR (101 MHz, $CDCl_3$) (difluoromethyl)(3,4,5-trimethoxyphenyl)sulfane 5d

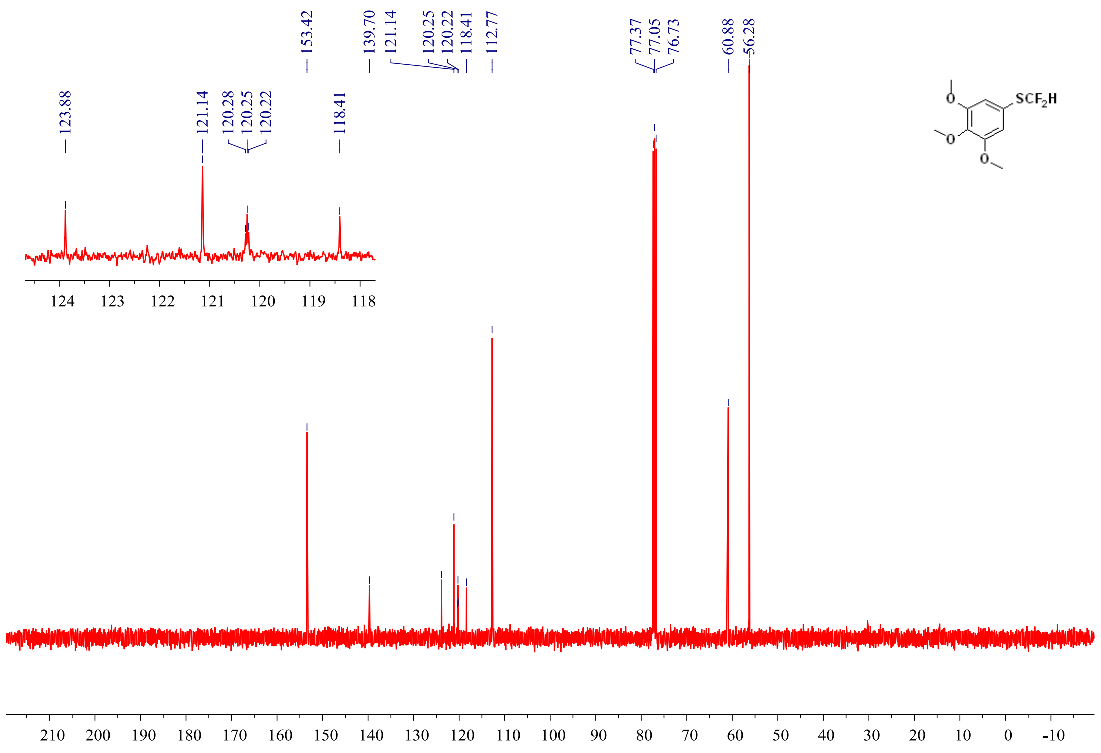

^{1}H NMR (400 MHz, $CDCl_3$) (difluoromethyl)(naphthalen-1-yl)sulfane 5e

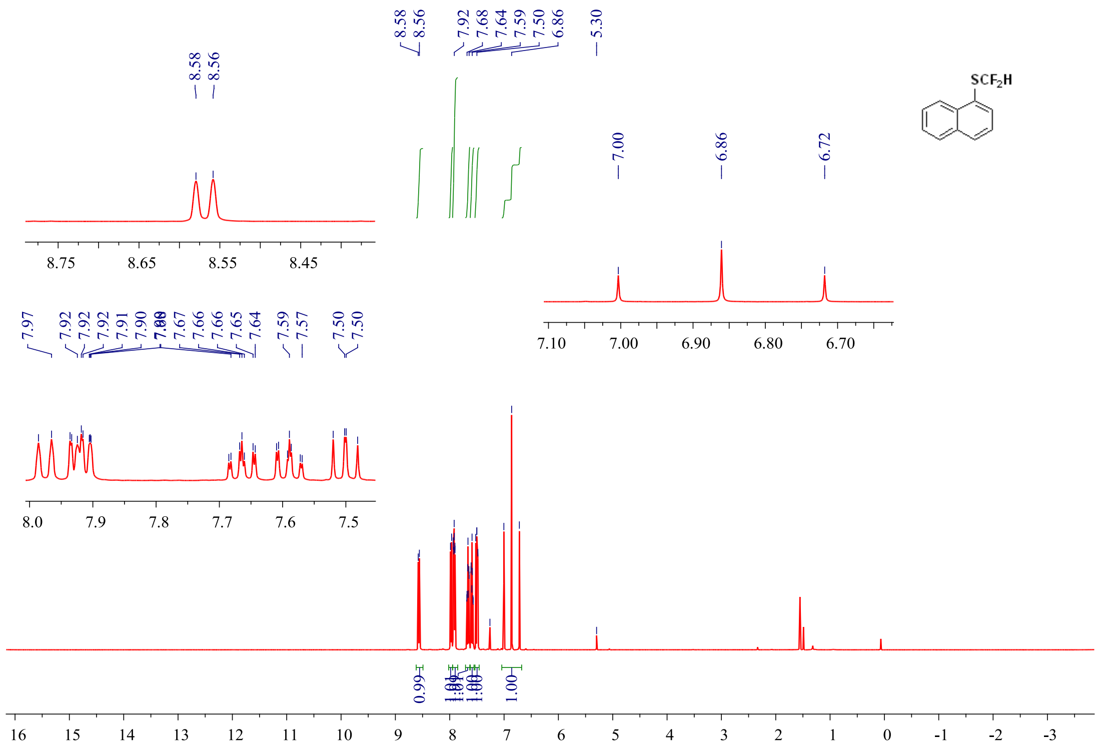

^{19}F NMR (376 MHz, $CDCl_3$) (difluoromethyl)(naphthalen-1-yl)sulfane 5e

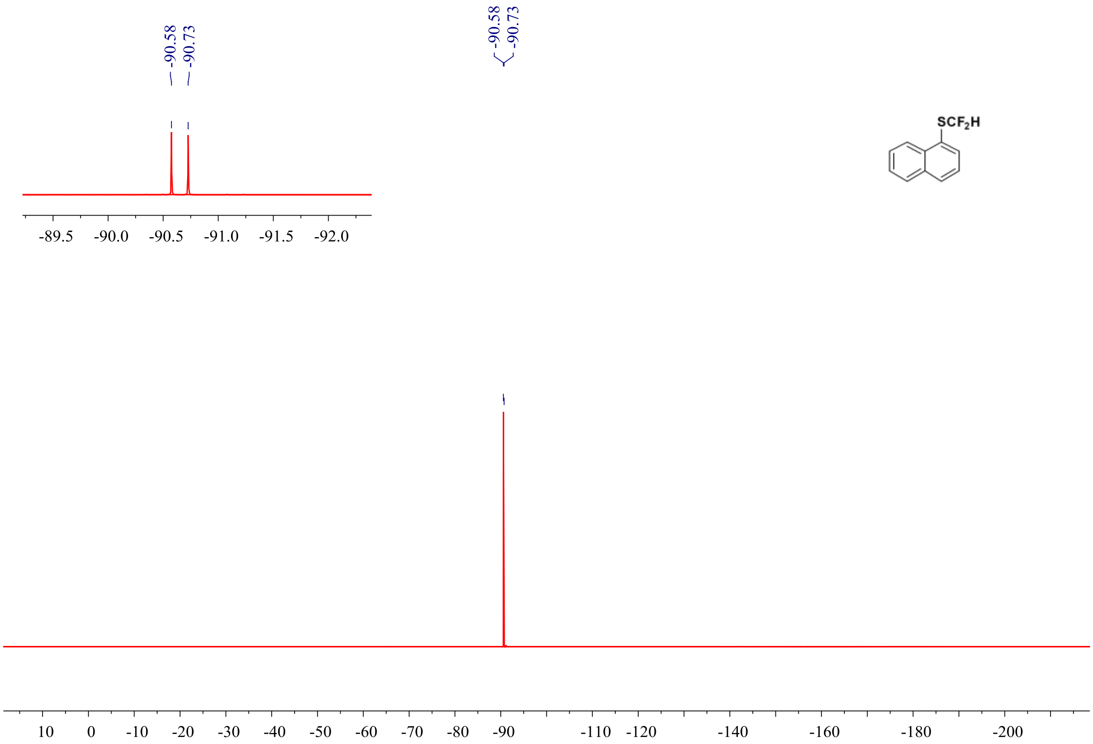

^{13}C NMR (101 MHz, $CDCl_3$) (difluoromethyl)(naphthalen-1-yl)sulfane 5e

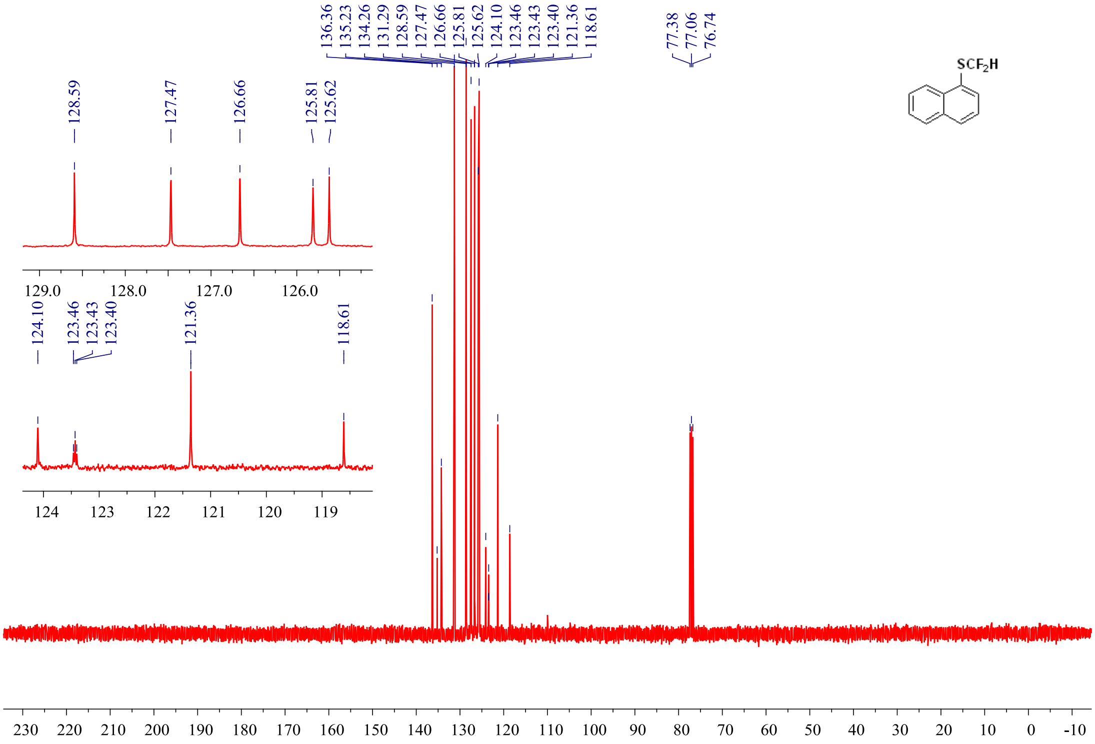

^{1}H NMR (400 MHz, $CDCl_3$) (difluoromethyl)(9*H*-fluoren-2-yl)sulfane 5f

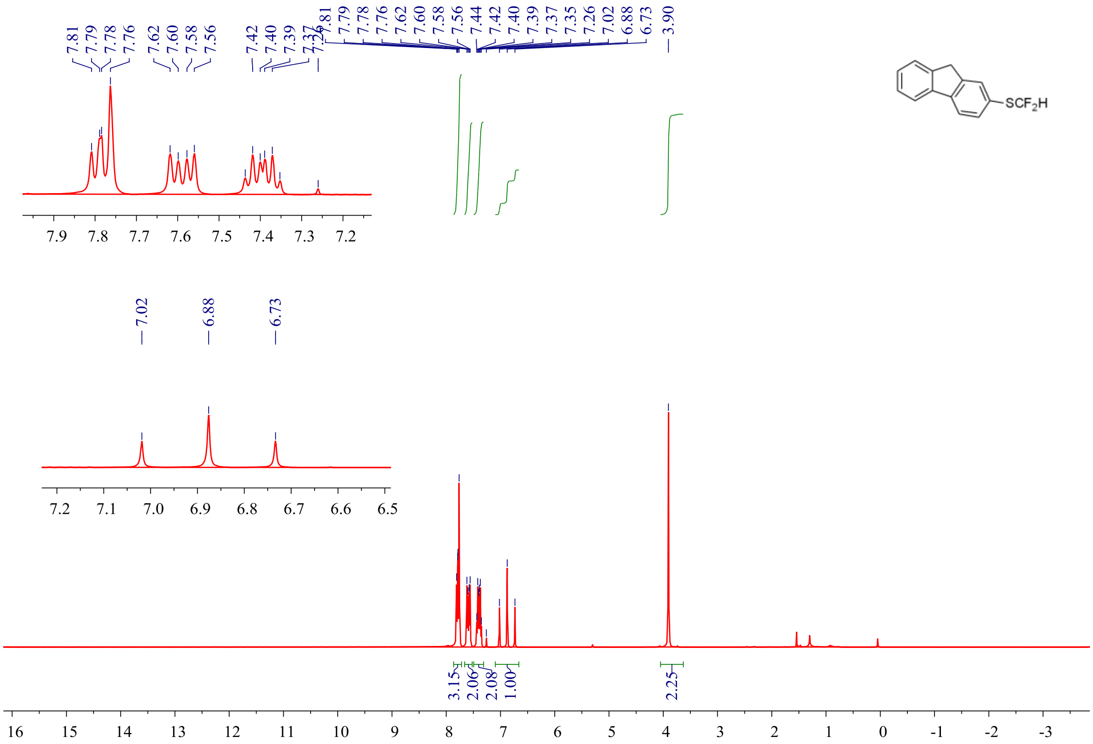

^{19}F NMR (376 MHz, $CDCl_3$) (difluoromethyl)(9*H*-fluoren-2-yl)sulfane 5f

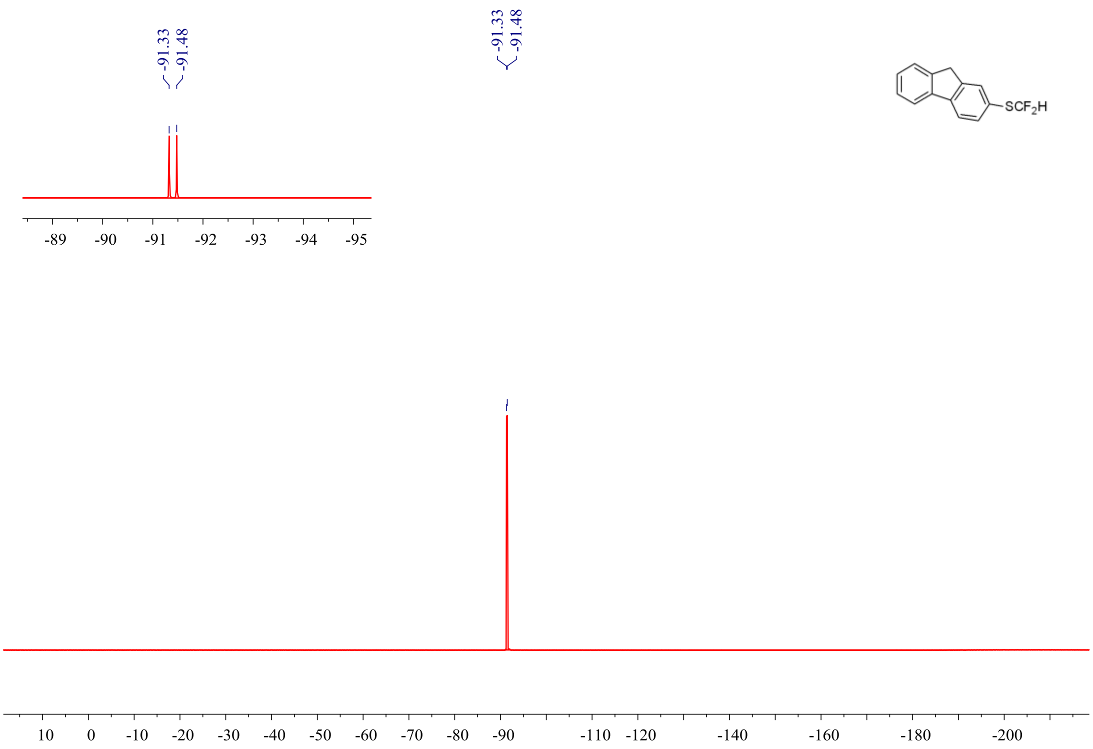

^{13}C NMR (101 MHz, $CDCl_3$) (difluoromethyl)(9*H*-fluoren-2-yl)sulfane 5f

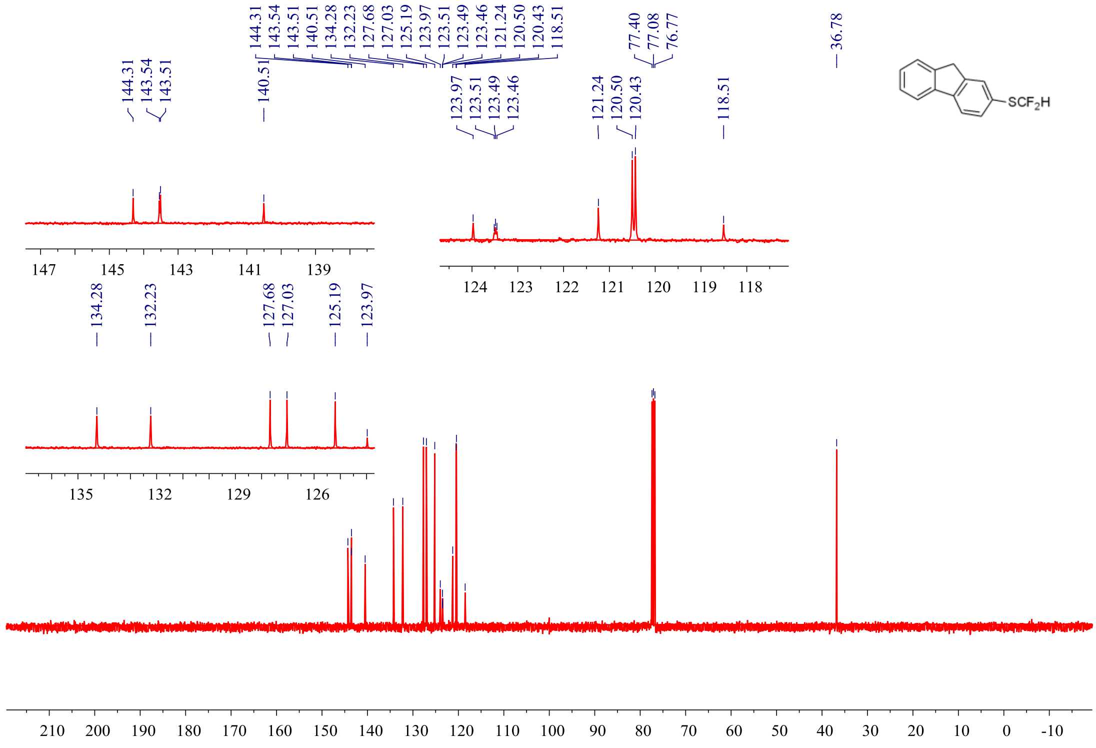

^{1}H NMR (400 MHz, $CDCl_3$) (Difluoromethyl)(phenanthren-9-yl)sulfane 5g

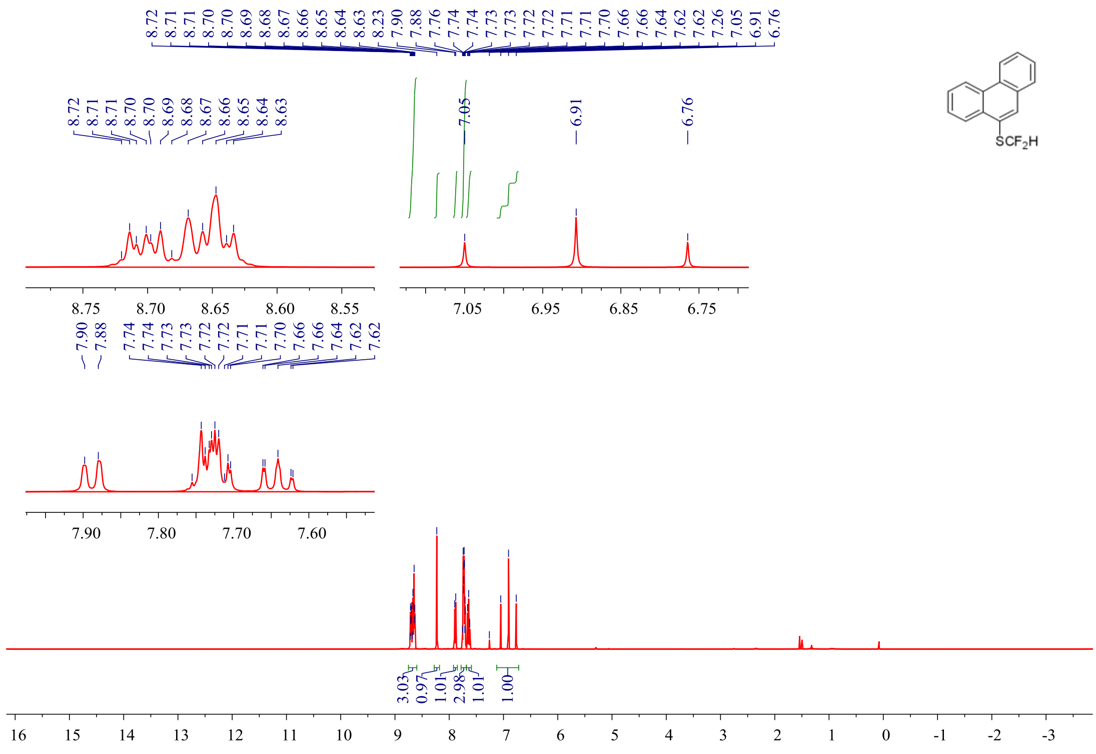

^{19}F NMR (376 MHz, $CDCl_3$) (difluoromethyl)(phenanthren-9-yl)sulfane 5g

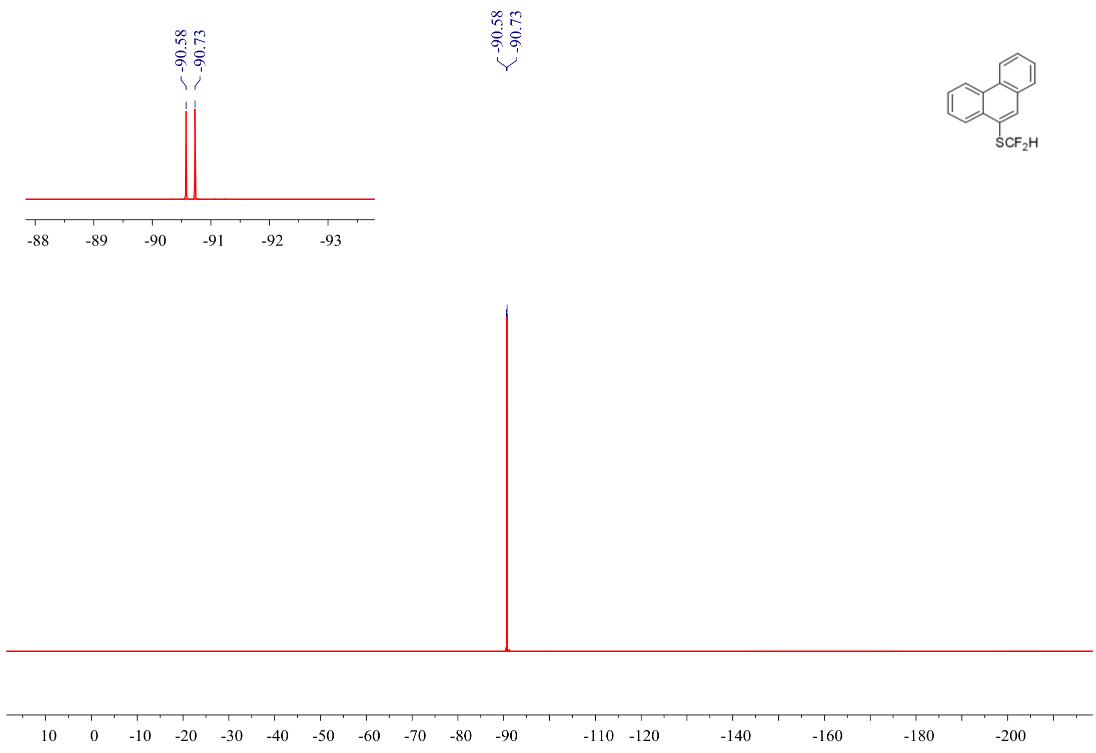

^{13}C NMR (101 MHz, $CDCl_3$) (difluoromethyl)(phenanthren-9-yl)sulfane 5g

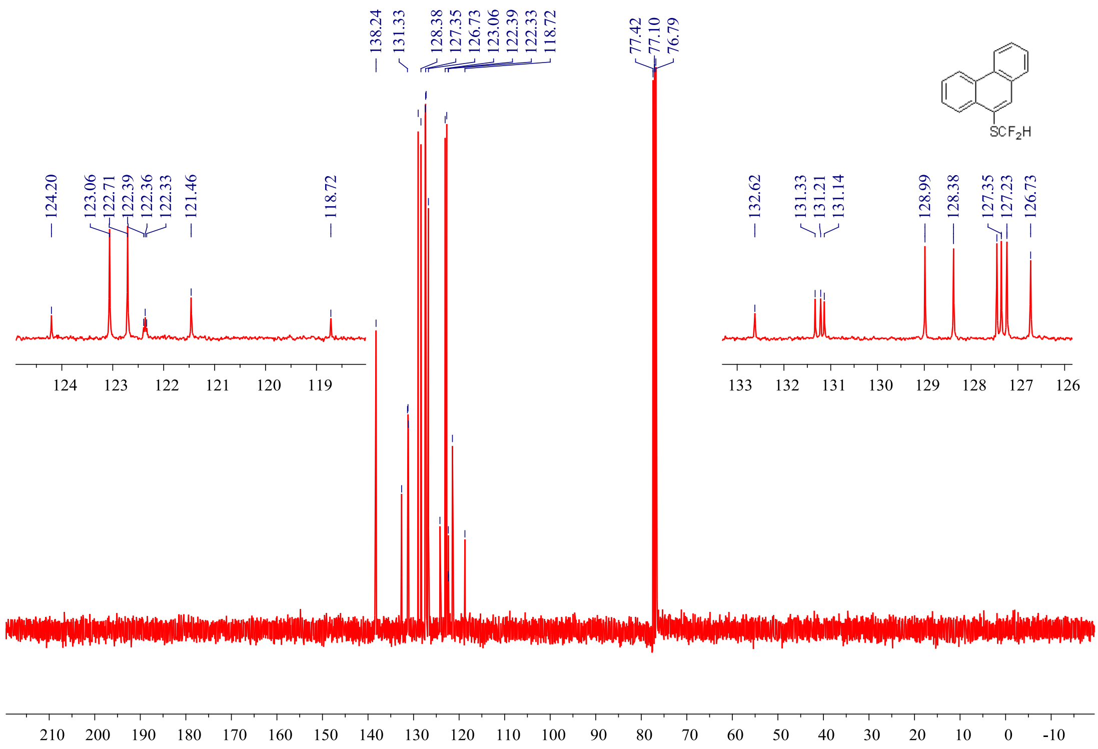

^{1}H NMR (400 MHz, $CDCl_3$) (3-(benzyloxy)phenyl)(difluoromethyl)sulfane 5h

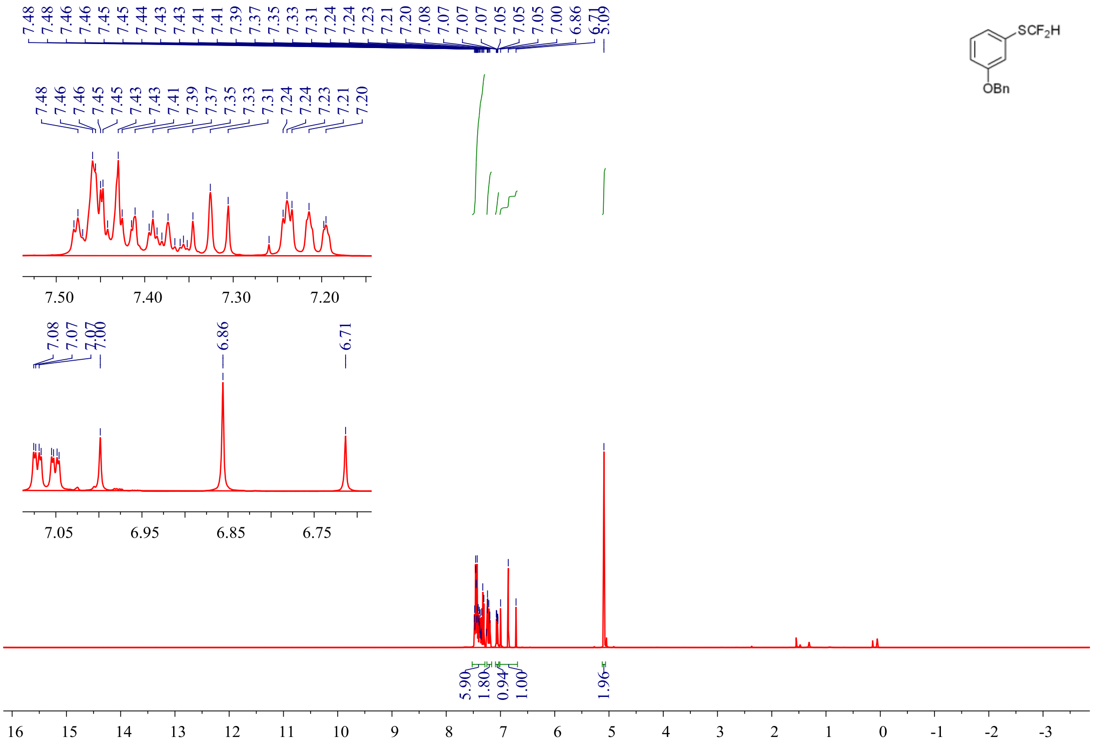

^{19}F NMR (376 MHz, $CDCl_3$) (3-(benzyloxy)phenyl)(difluoromethyl)sulfane 5h

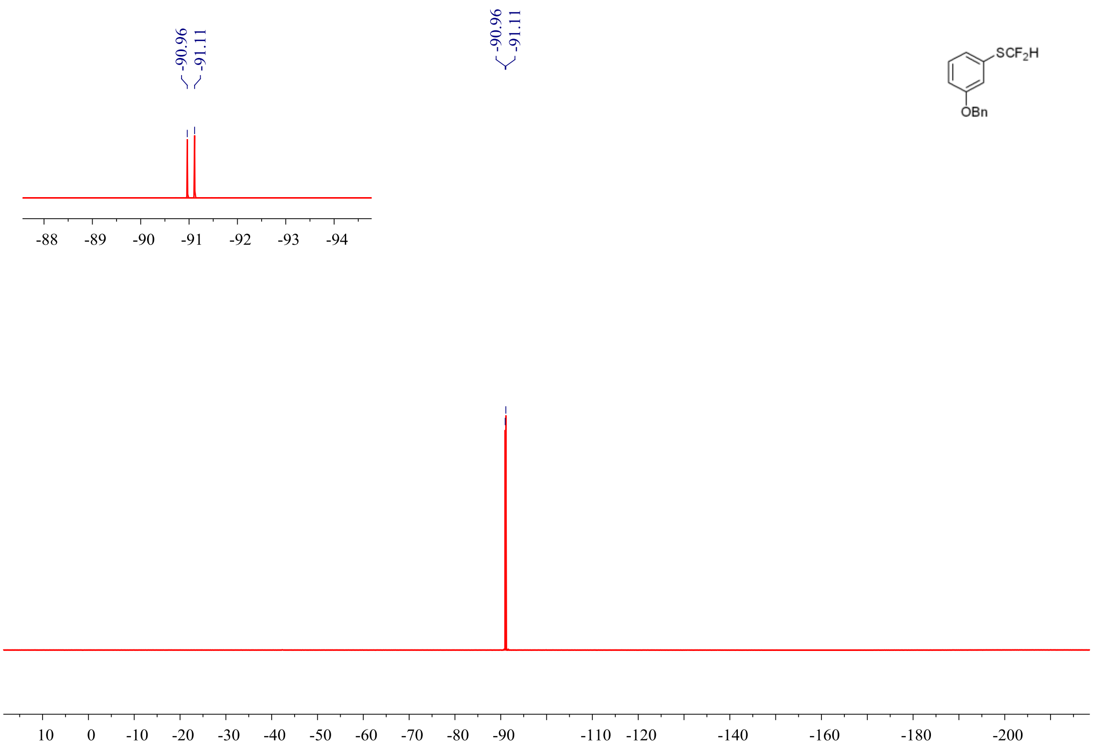

^{13}C NMR (101 MHz, $CDCl_3$ (3-(benzyloxy)phenyl)(difluoromethyl)sulfane 5h

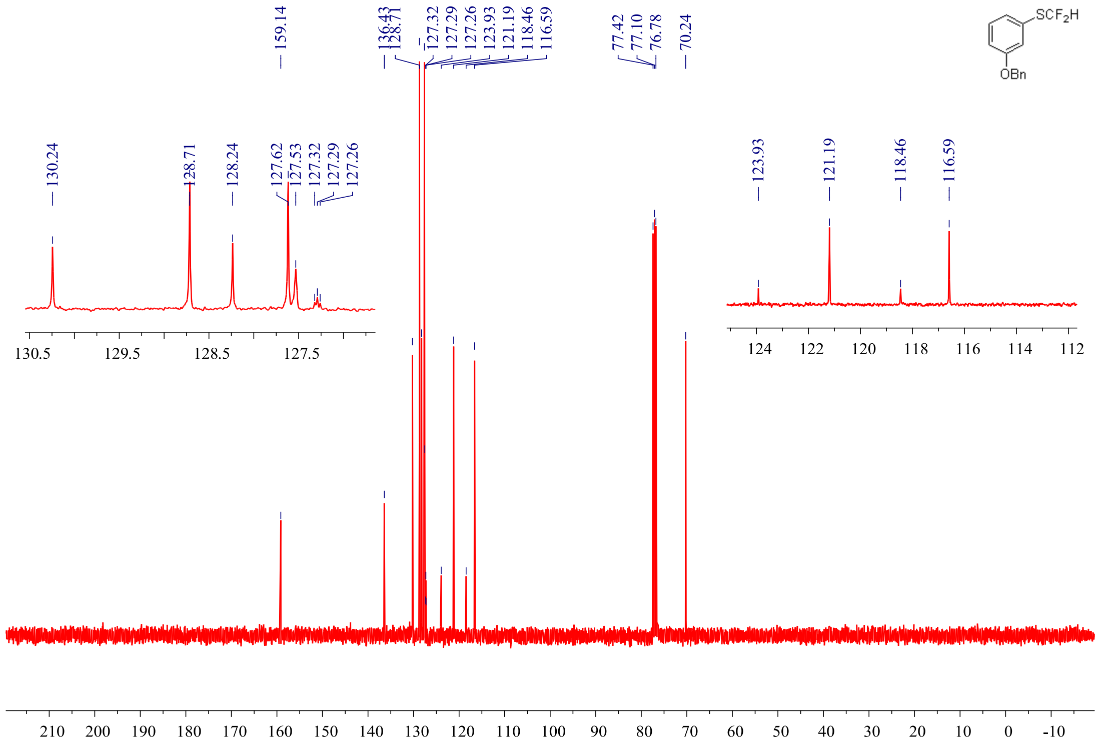

^{1}H NMR (400 MHz, $CDCl_3$) (difluoromethyl)(4-nitrophenyl)sulfane 5i

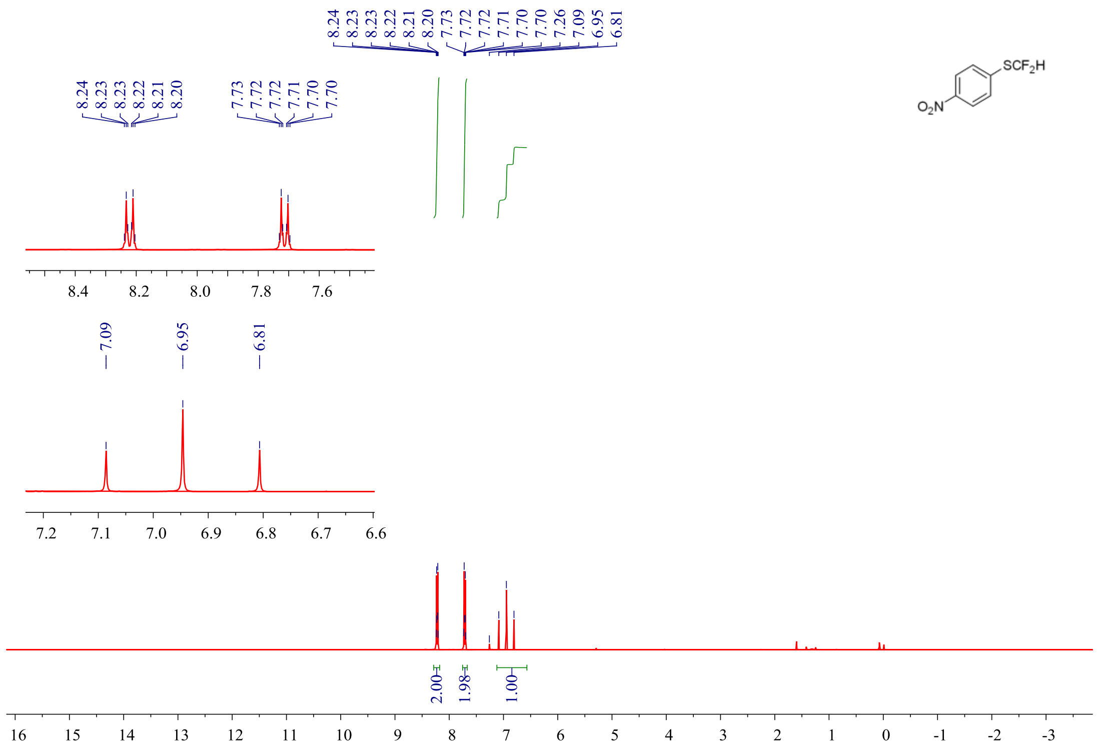

^{19}F NMR (376 MHz, $CDCl_3$) (difluoromethyl)(4-nitrophenyl)sulfane 5i

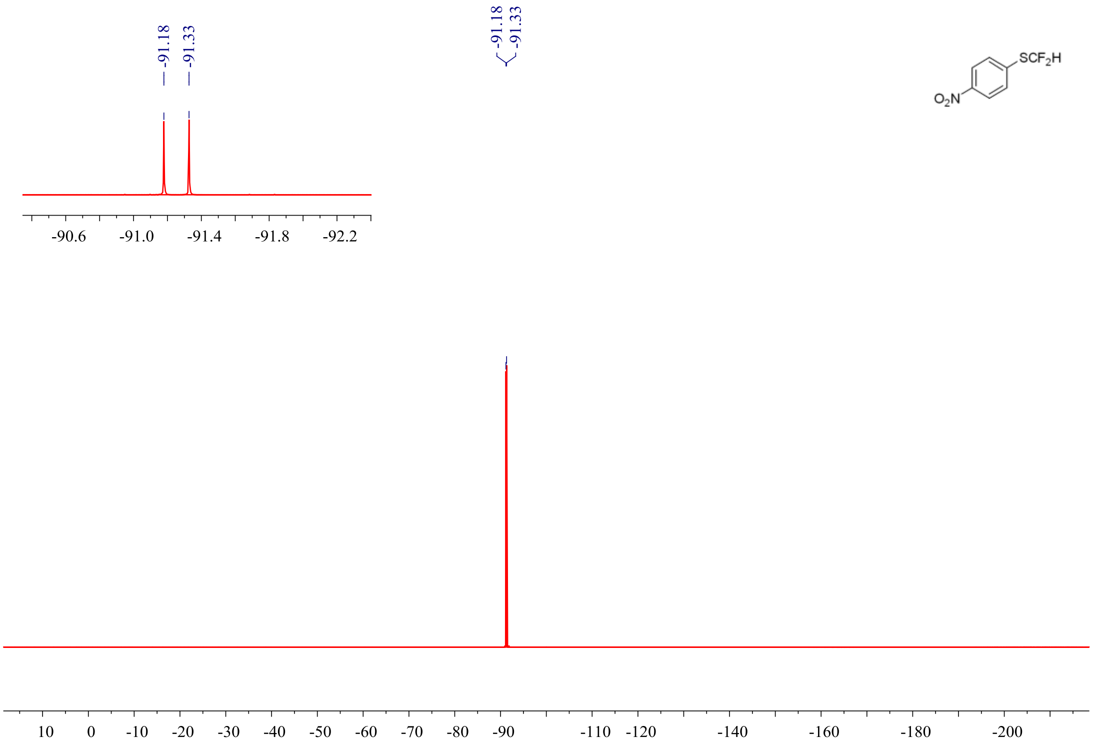

^{13}C NMR (101 MHz, $CDCl_3$) (difluoromethyl)(4-nitrophenyl)sulfane 5i

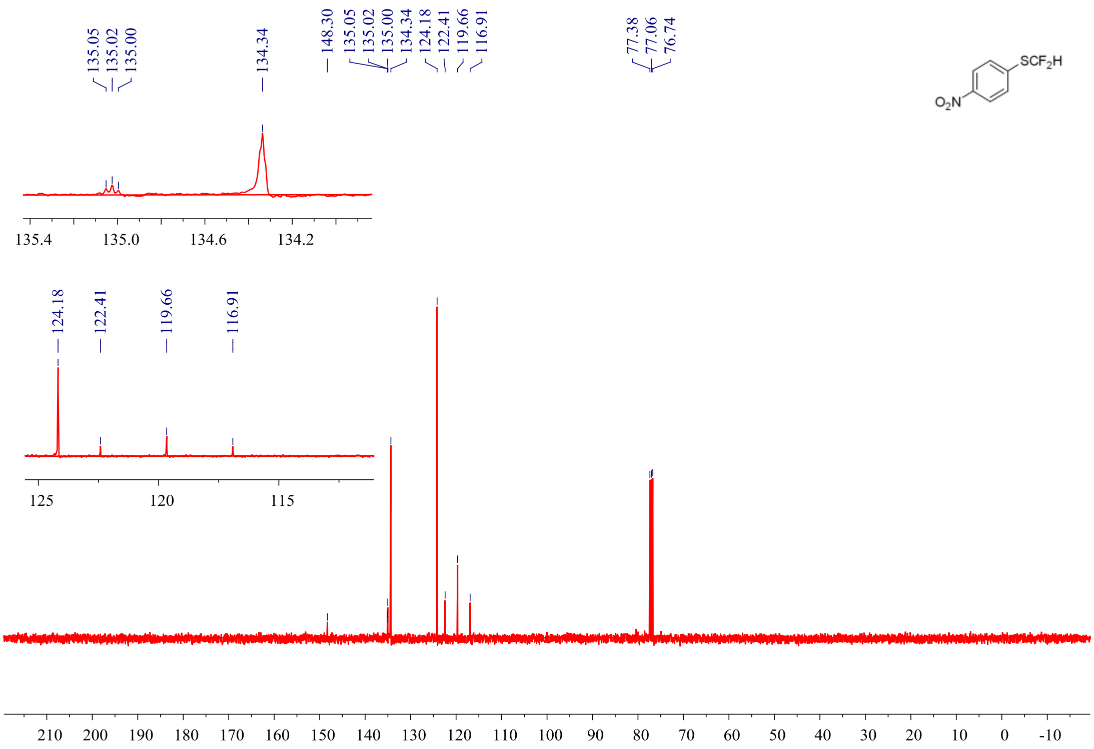

^{1}H NMR (400 MHz, $CDCl_3$) (difluoromethyl)(2-nitrophenyl)sulfane 5j

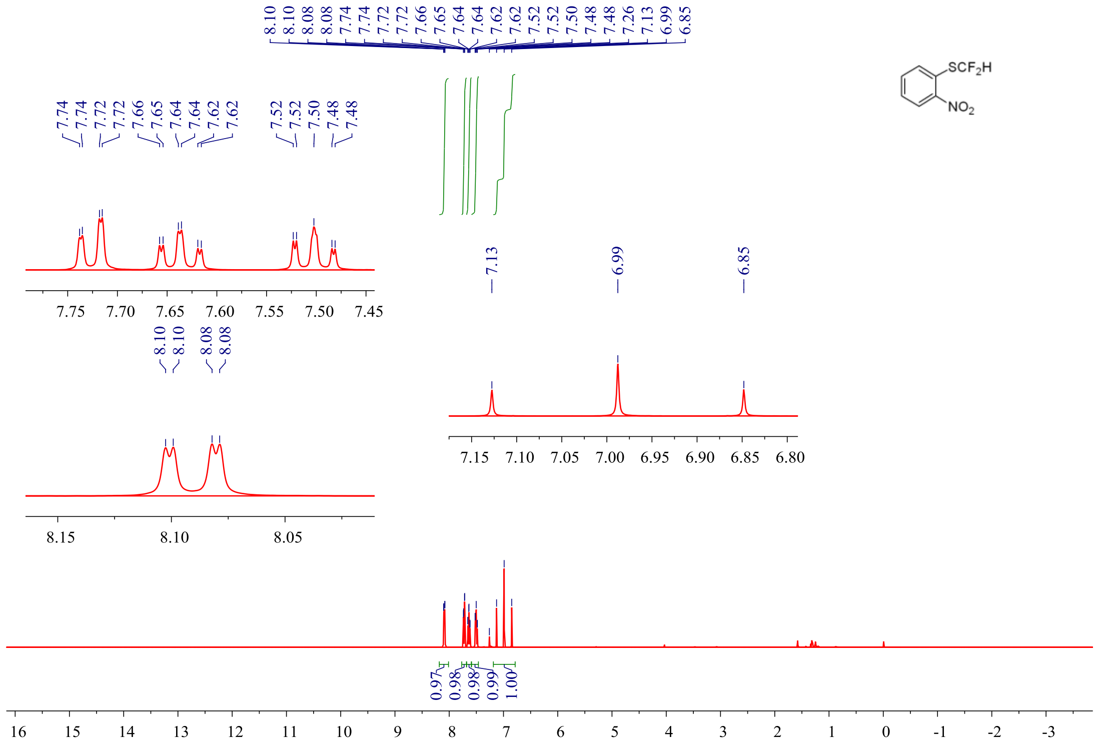

^{19}F NMR (376 MHz, $CDCl_3$) (difluoromethyl)(2-nitrophenyl)sulfane 5j

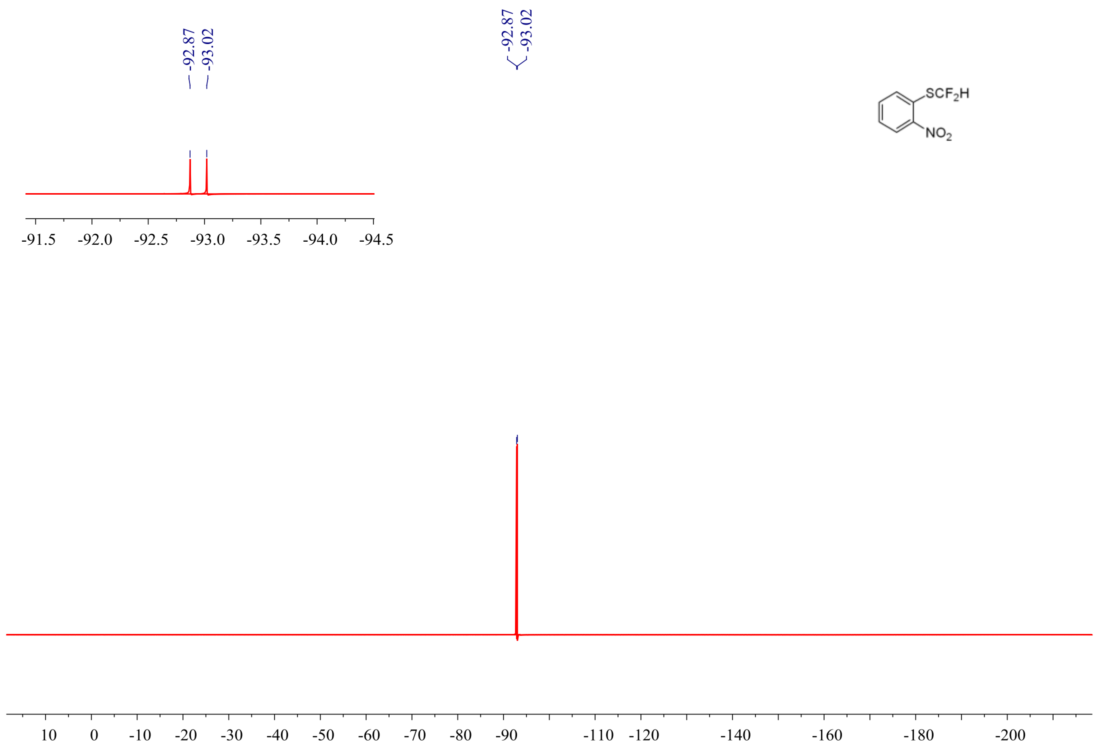

^{13}C NMR (101 MHz, $CDCl_3$) (difluoromethyl)(2-nitrophenyl)sulfane 5j

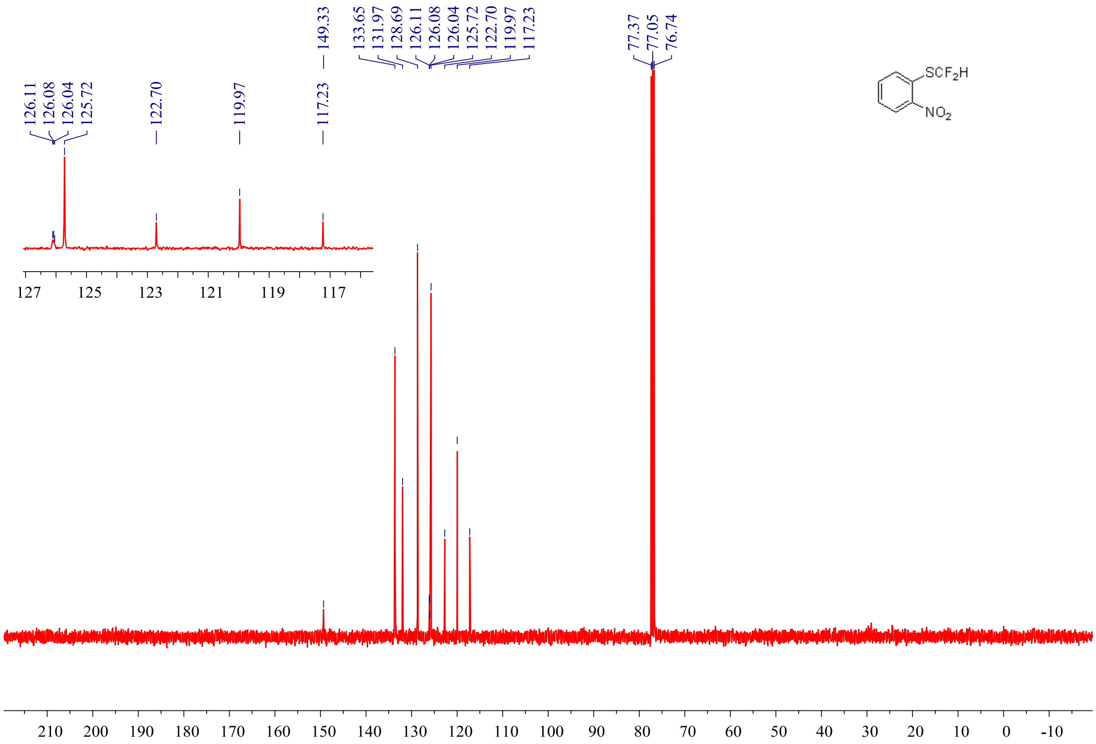

^{1}H NMR (400 MHz, $CDCl_3$) (difluoromethyl)(3-fluoro-5-nitrophenyl)sulfane 5k

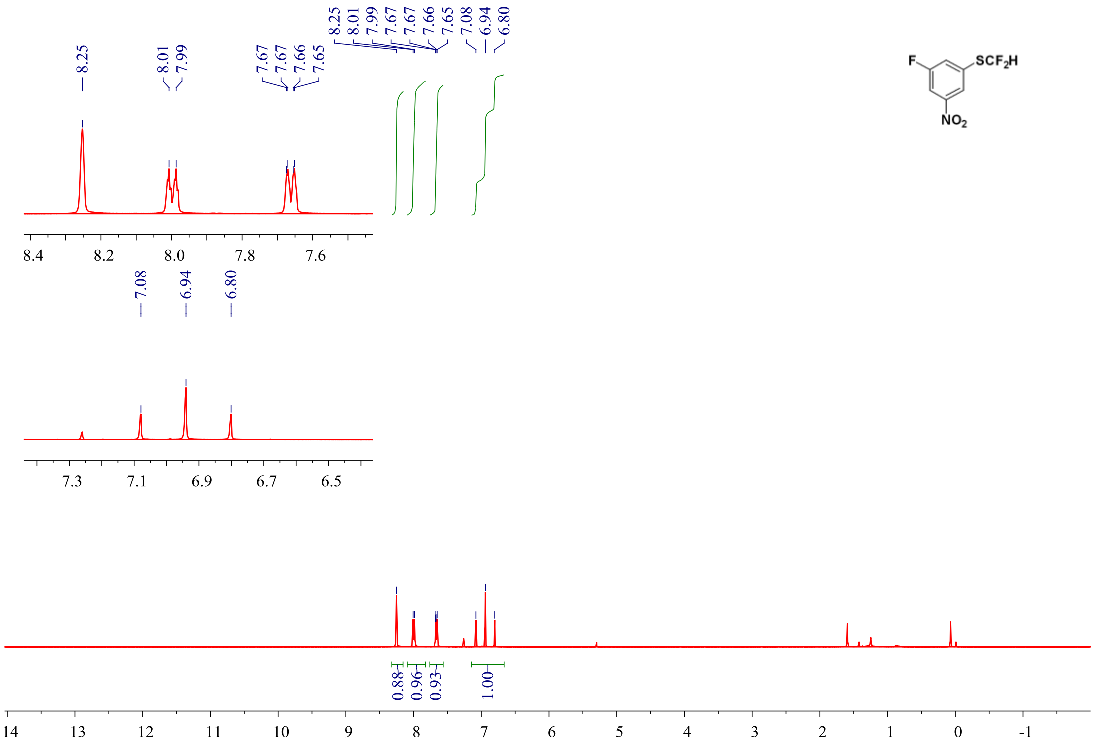

^{19}F NMR (376 MHz, $CDCl_3$) (difluoromethyl)(3-fluoro-5-nitrophenyl)sulfane 5k

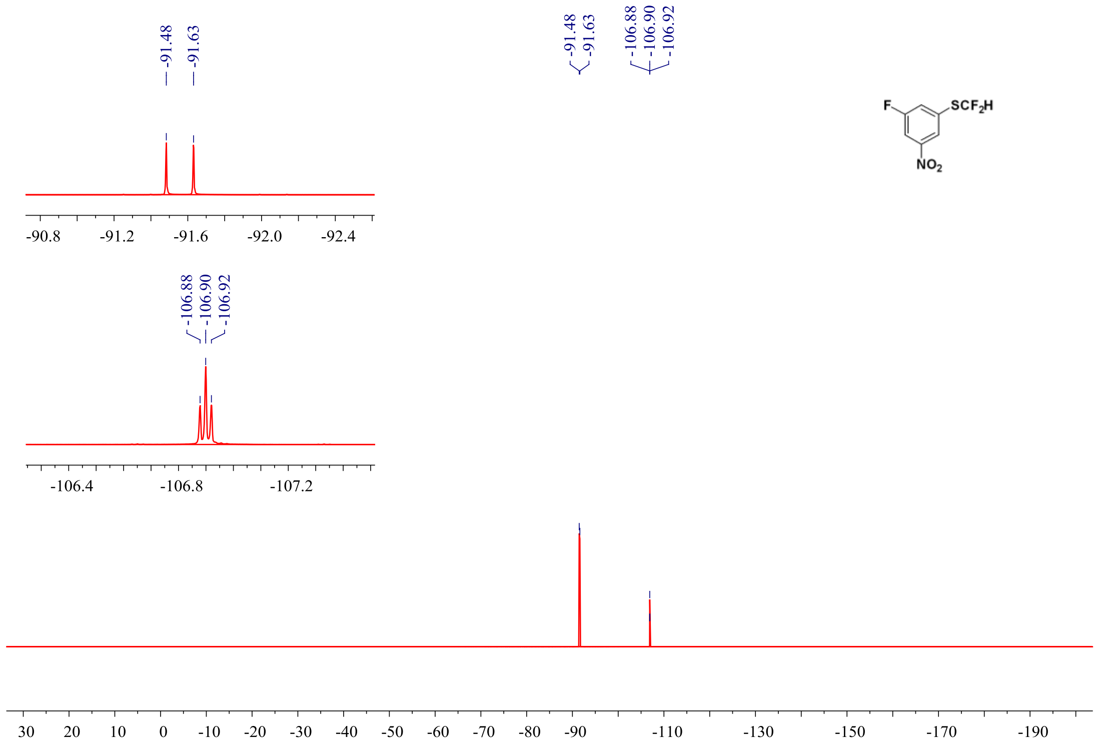

^{13}C NMR (101 MHz, $CDCl_3$) (difluoromethyl)(3-fluoro-5-nitrophenyl)sulfane 5k

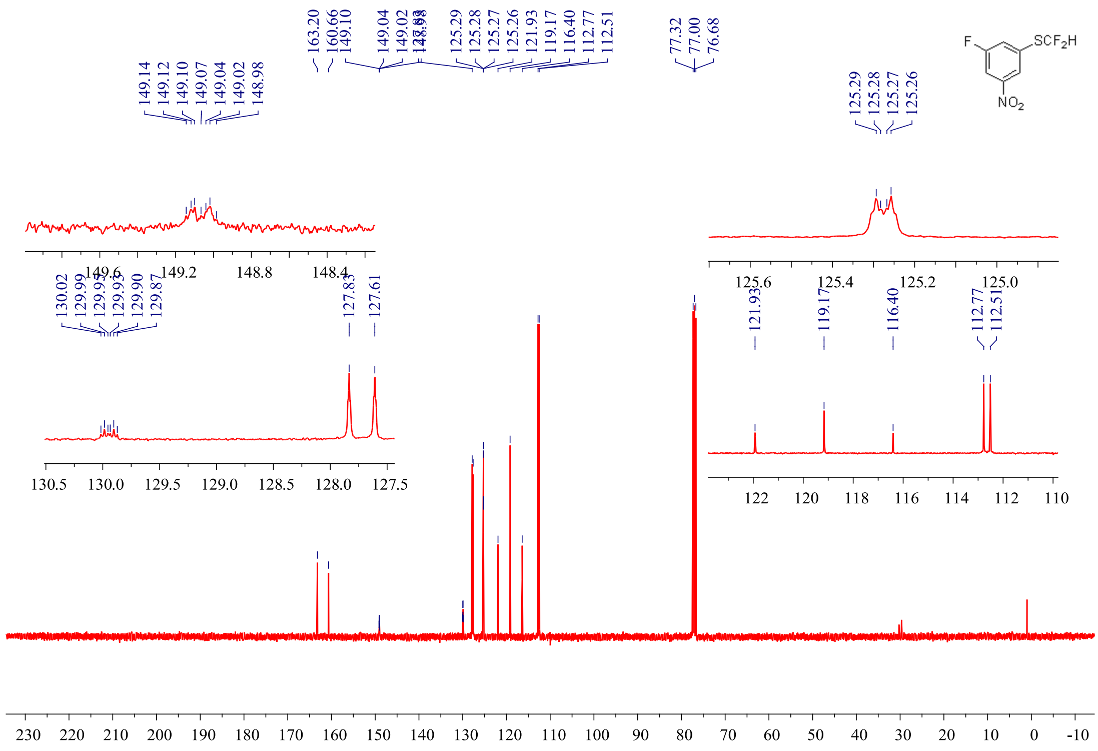

^{1}H NMR (400 MHz, $CDCl_3$) (3,4-dichlorophenyl)(difluoromethyl)sulfane 5l

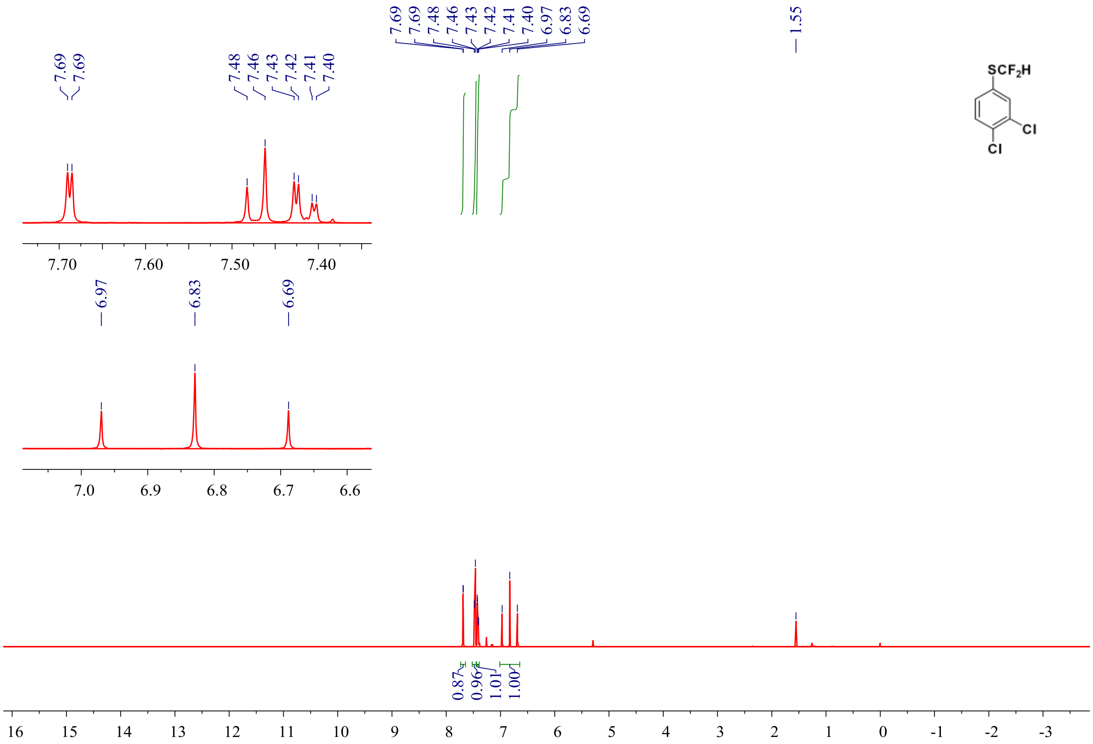

^{19}F NMR (376 MHz, $CDCl_3$) (3,4-dichlorophenyl)(difluoromethyl)sulfane 5l

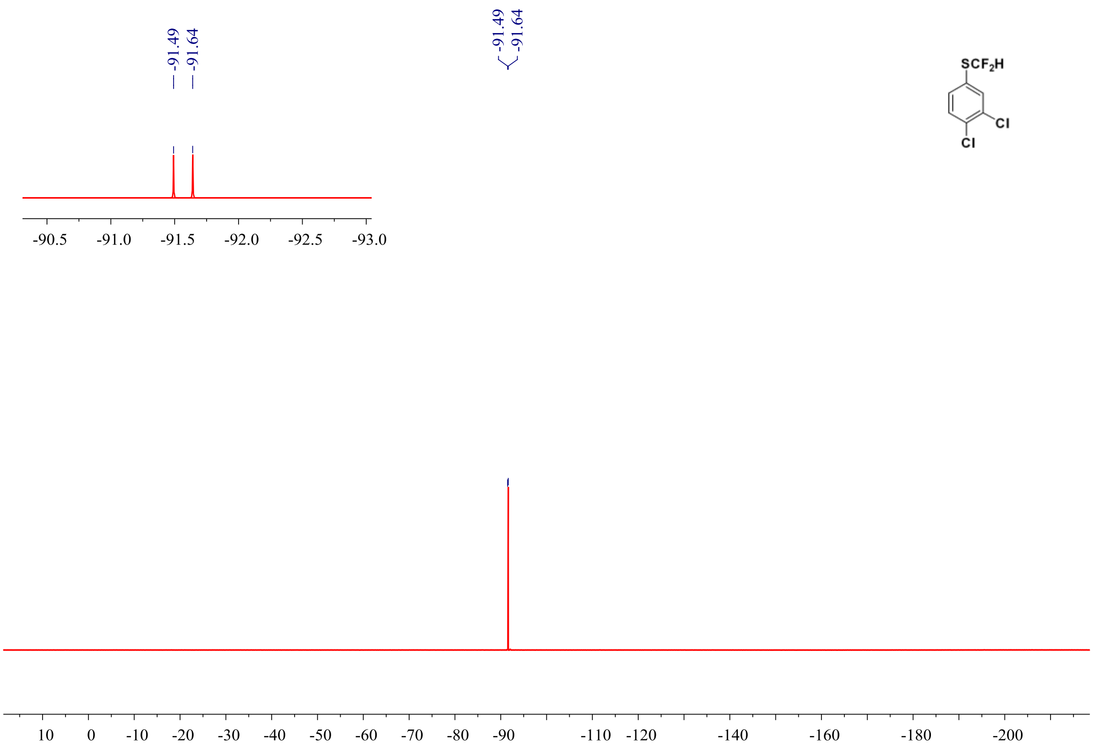

^{13}C NMR (101 MHz, $CDCl_3$) (3,4-dichlorophenyl)(difluoromethyl)sulfane 5l

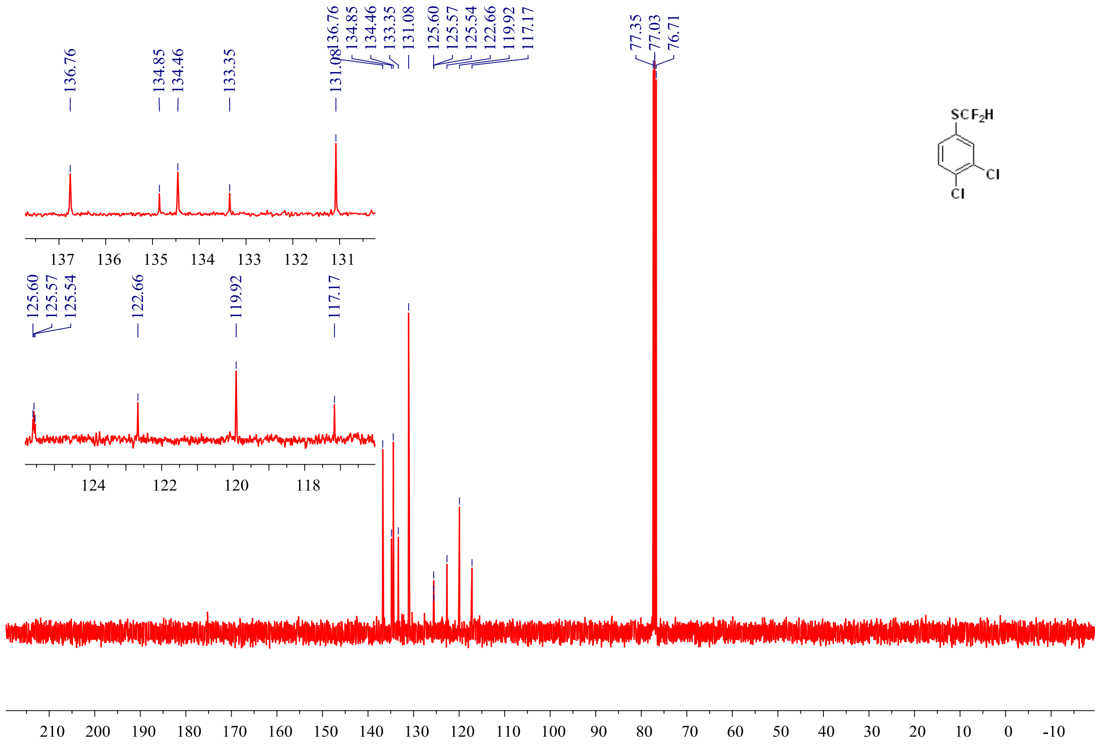

^{1}H NMR (400 MHz, $CDCl_3$) (3-bromophenyl)(difluoromethyl)sulfane 5m

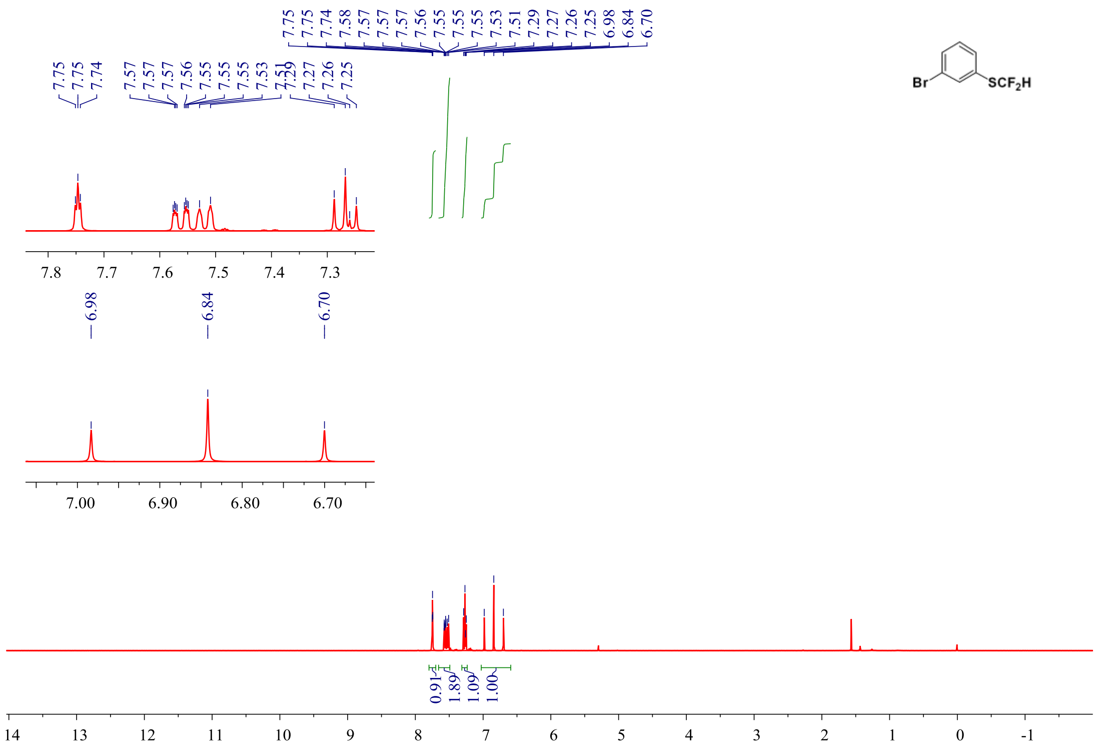

^{19}F NMR (376 MHz, $CDCl_3$) (3-bromophenyl)(difluoromethyl)sulfane 5m

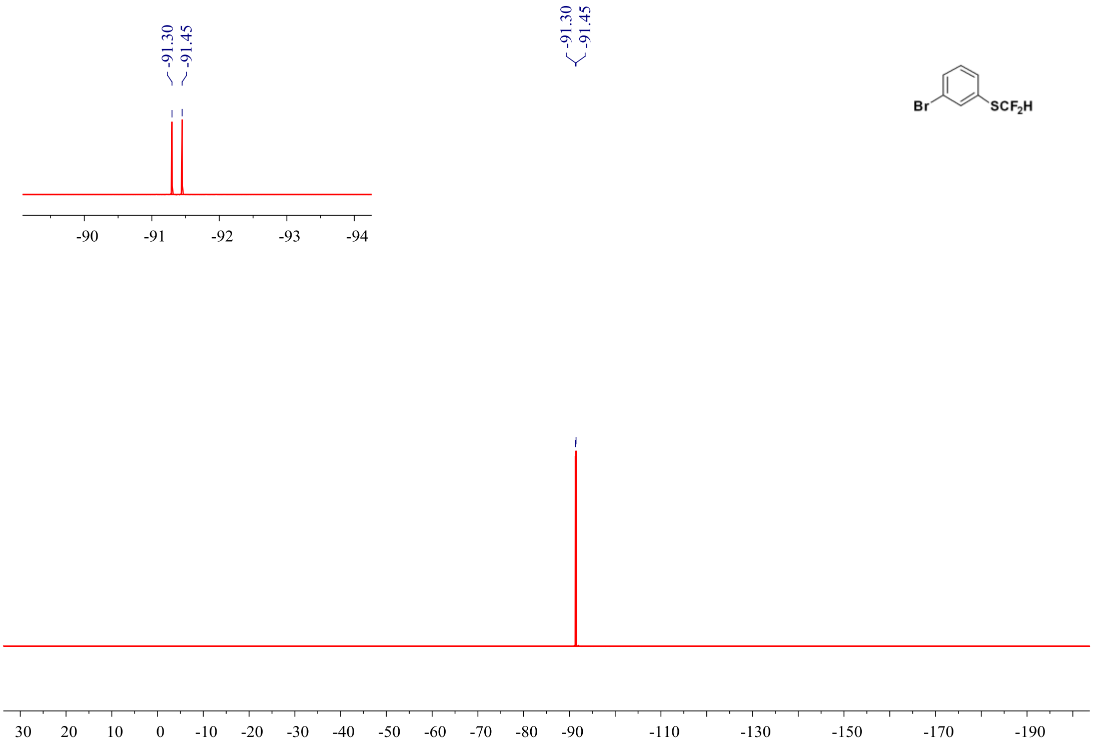

^{13}C NMR (101 MHz, $CDCl_3$) (3-bromophenyl)(difluoromethyl)sulfane 5m

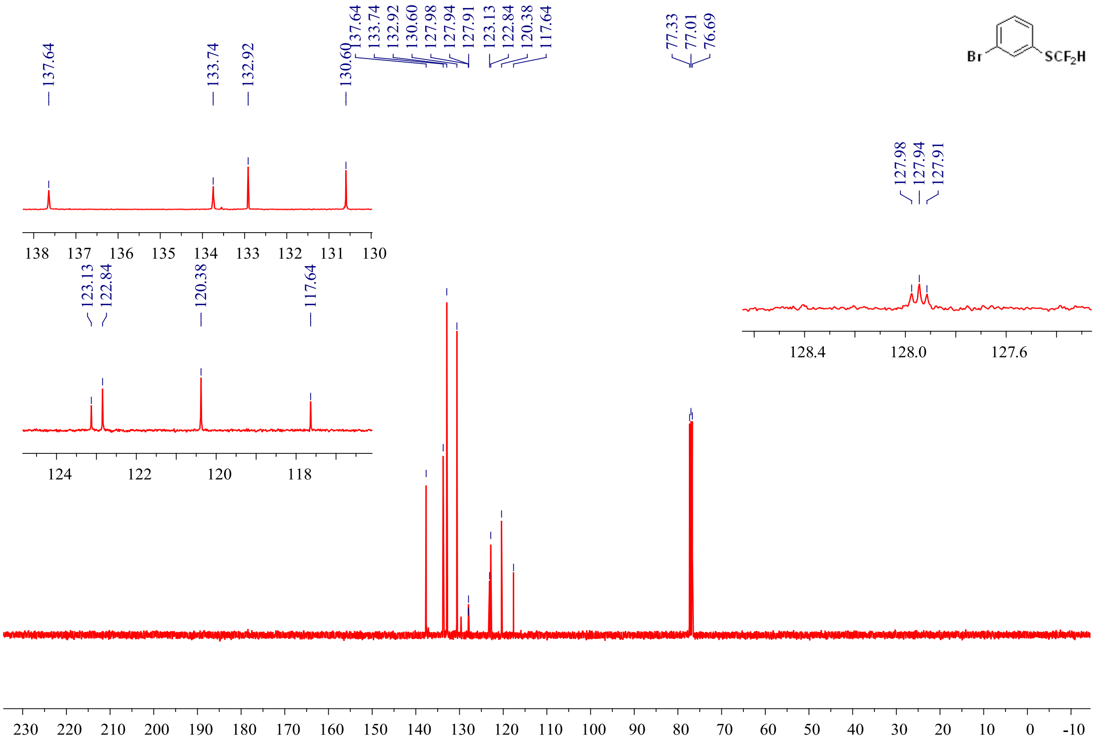

^{1}H NMR (400 MHz, $CDCl_3$) (4-bromophenyl)(difluoromethyl)sulfane 5n

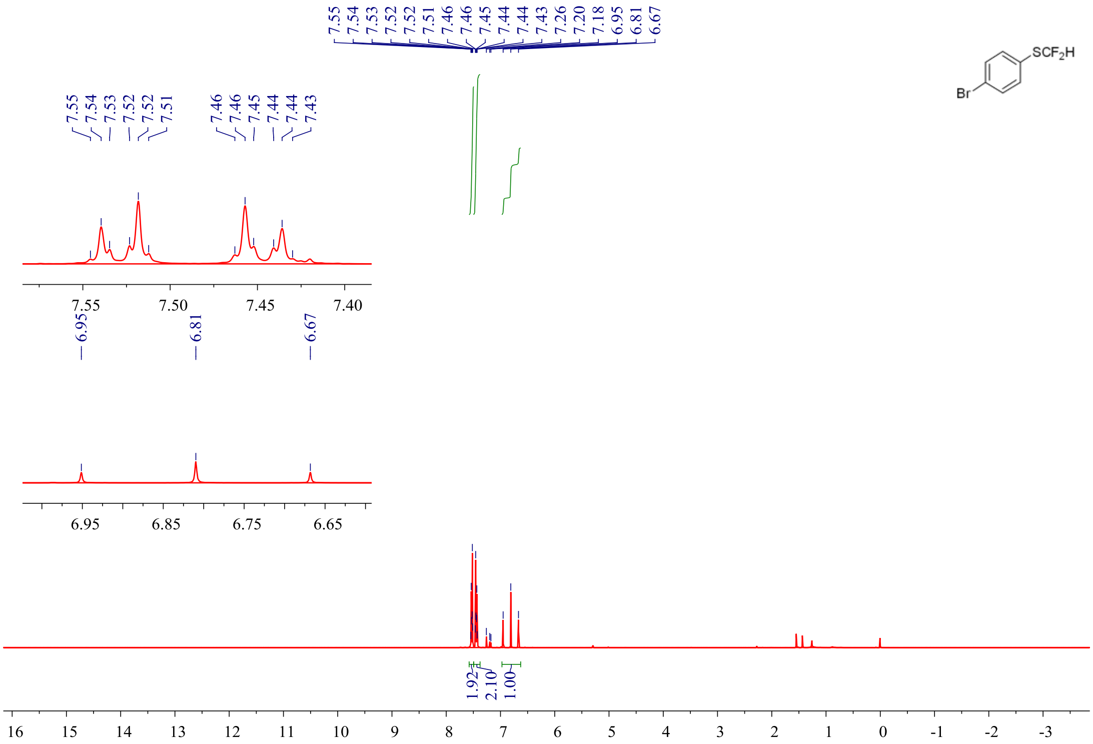

^{19}F NMR (376 MHz, $CDCl_3$) (4-bromophenyl)(difluoromethyl)sulfane 5n

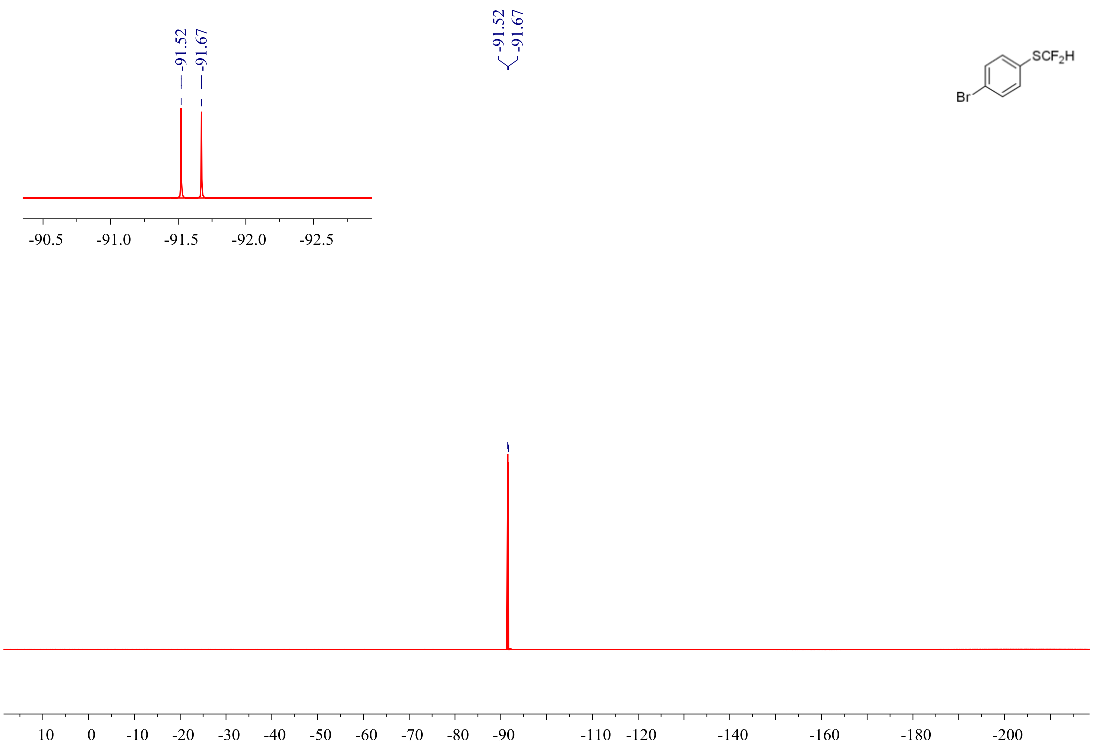

^{13}C NMR (101 MHz, $CDCl_3$) (4-bromophenyl)(difluoromethyl)sulfane 5n

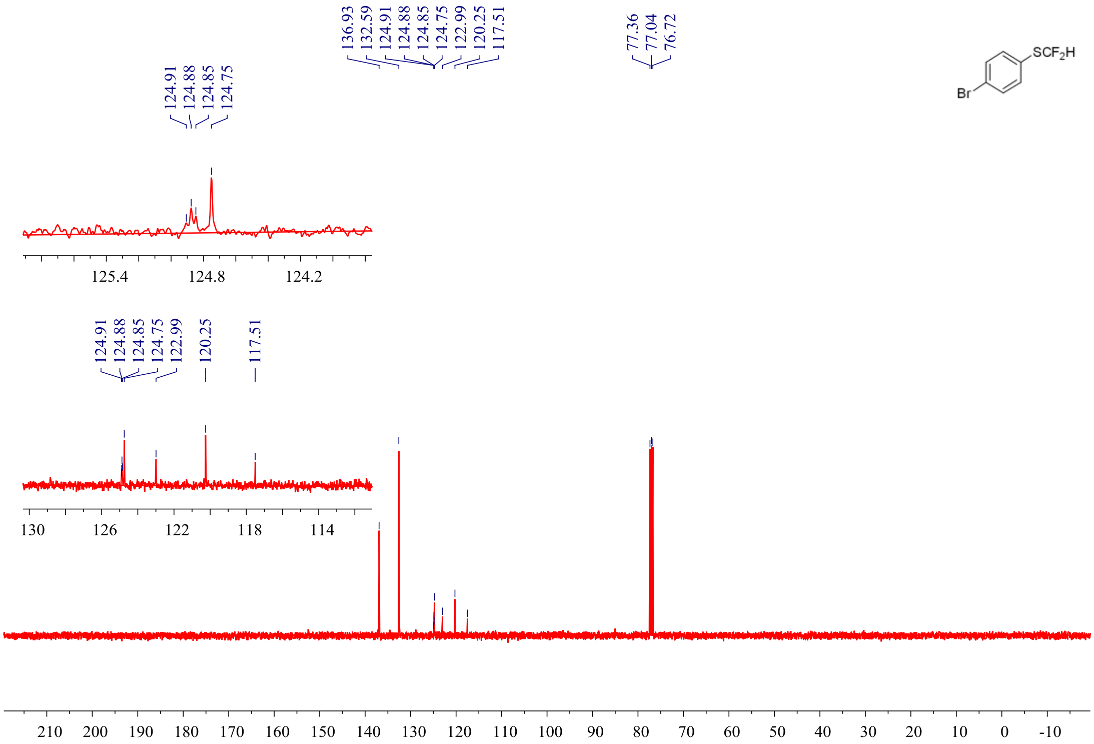

^{1}H NMR (400 MHz, $CDCl_3$) 1-(4-((difluoromethyl)thio)phenyl)ethanone 5o

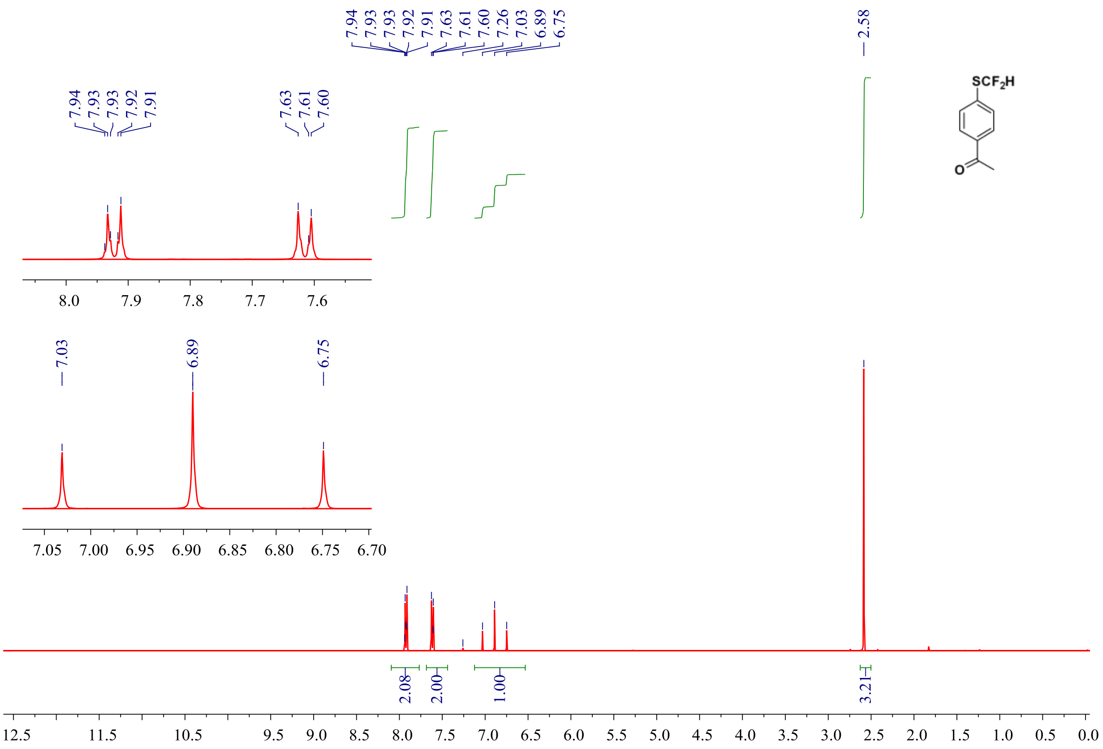

^{19}F NMR (376 MHz, $CDCl_3$) 1-(4-((difluoromethyl)thio)phenyl)ethanone 5o

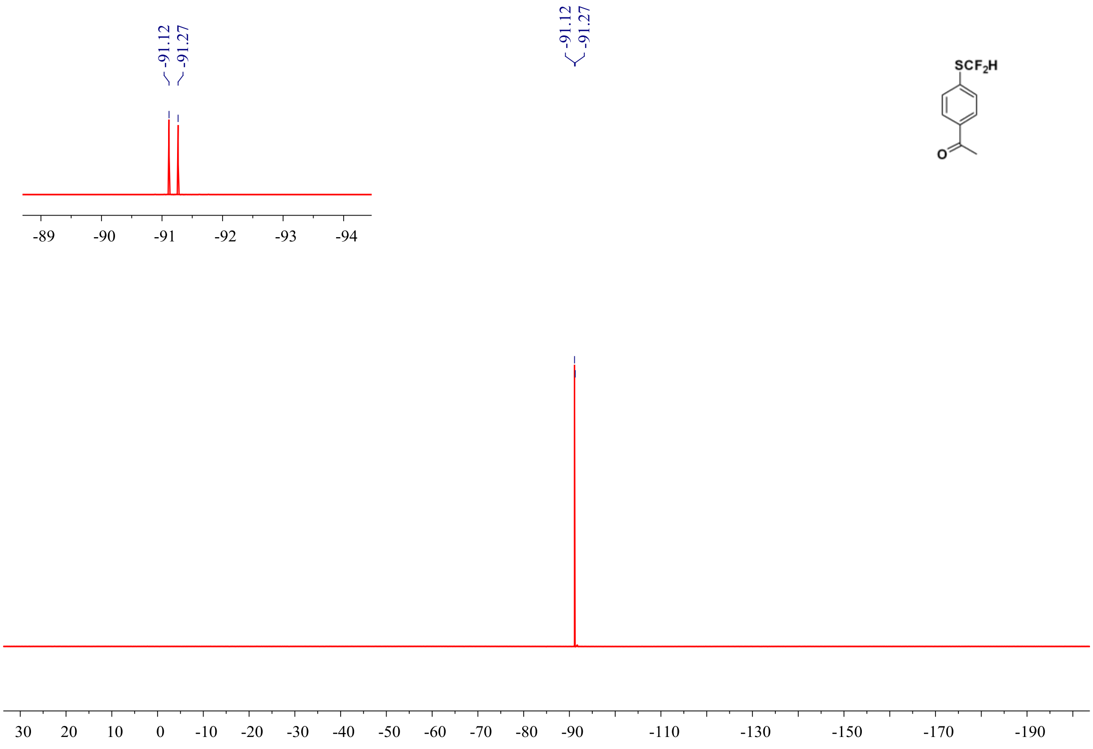

^{13}C NMR (101 MHz, $CDCl_3$) 1-(4-((difluoromethyl)thio)phenyl)ethanone 5o

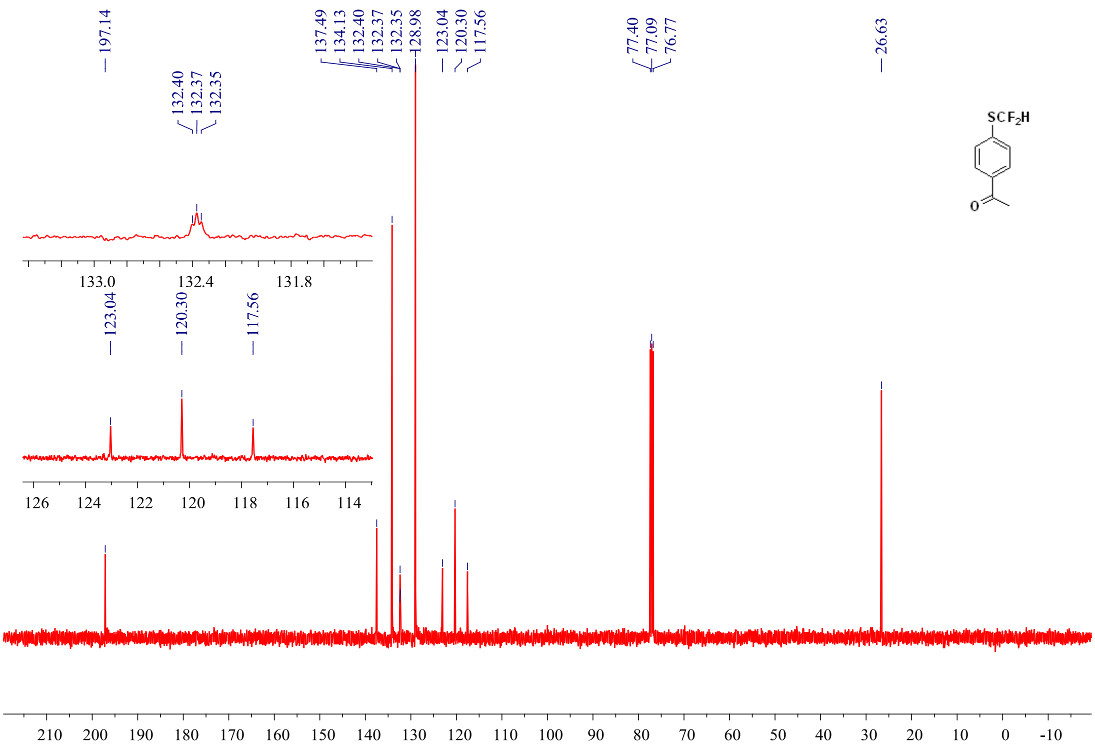

^{1}H NMR (400 MHz, $CDCl_3$) Methyl 2-((difluoromethyl)thio)benzoate 5p

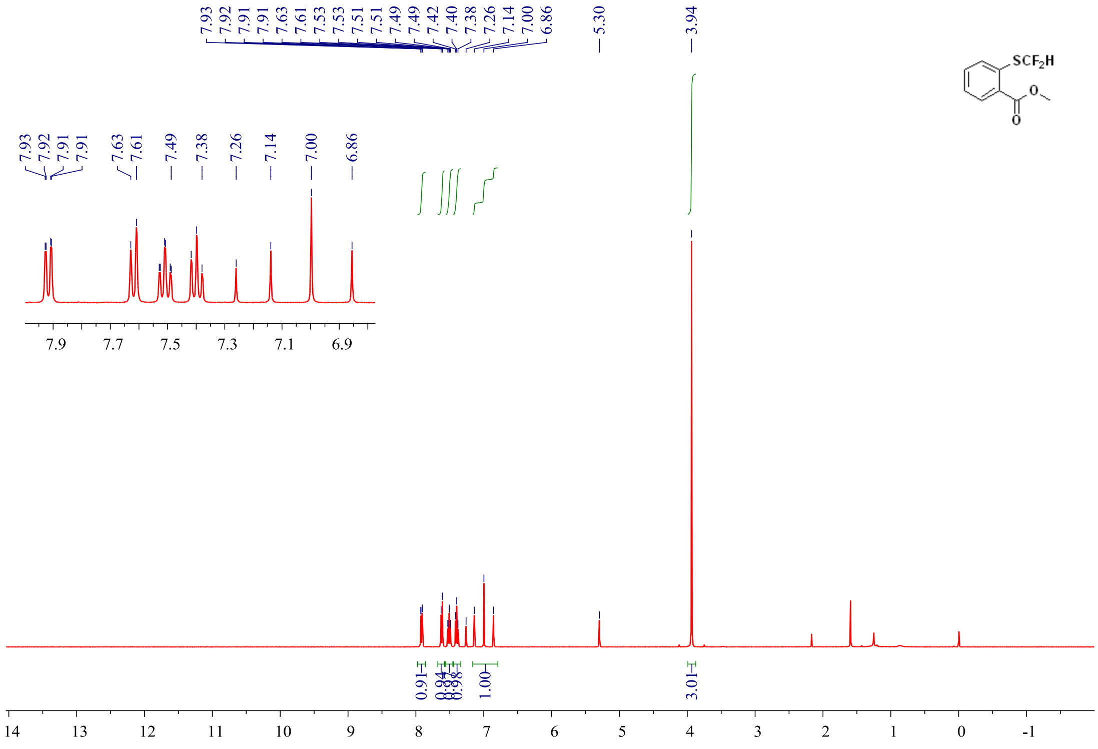

^{19}F NMR (376 MHz, $CDCl_3$) Methyl 2-((difluoromethyl)thio)benzoate 5p

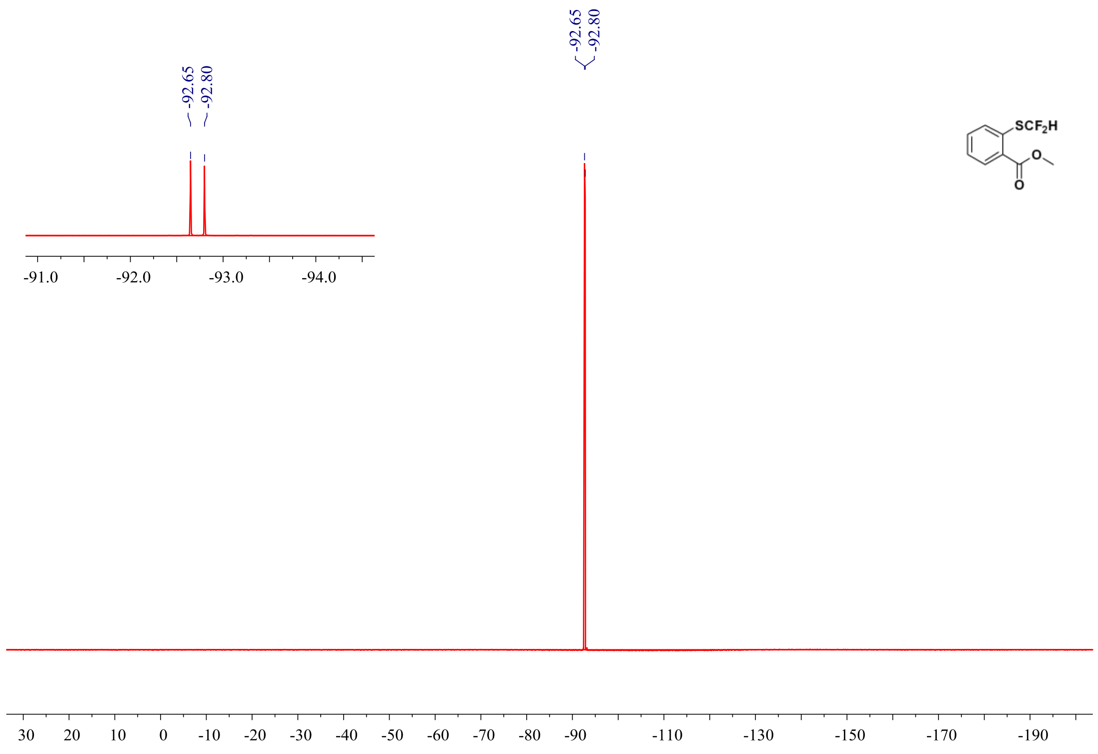

^{13}C NMR (101 MHz, $CDCl_3$) Methyl 2-((difluoromethyl)thio)benzoate 5p

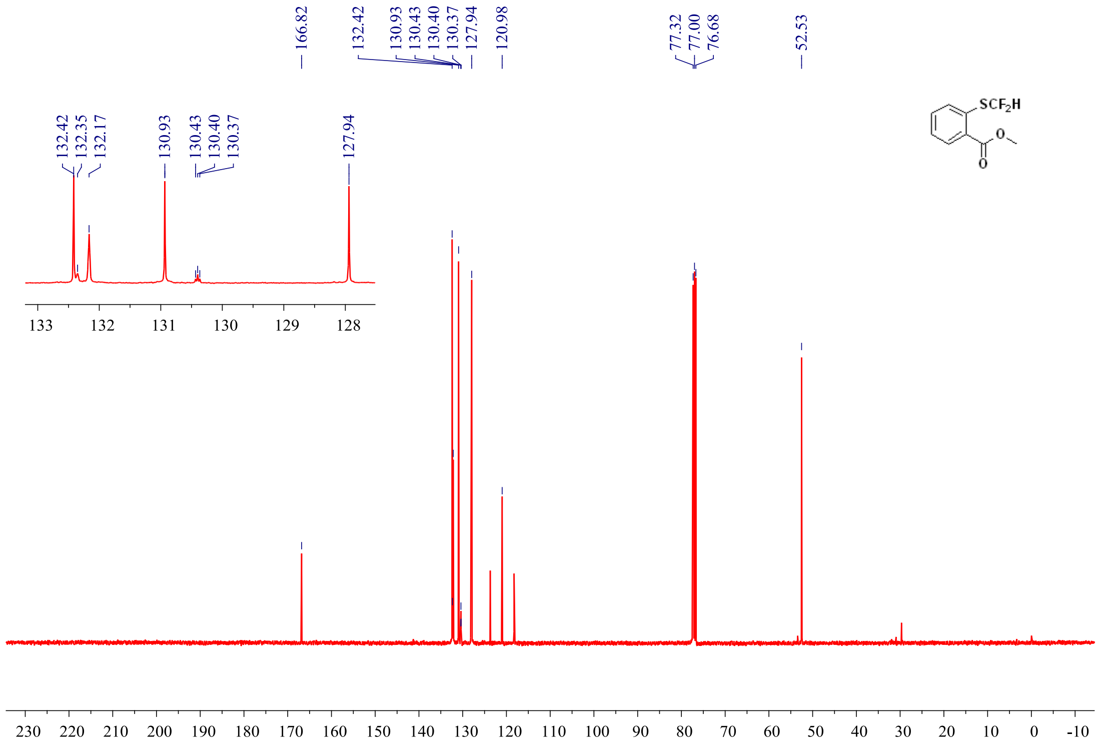

^{1}H NMR (400 MHz, $CDCl_3$) Methyl 4-((difluoromethyl)thio)benzoate 5q

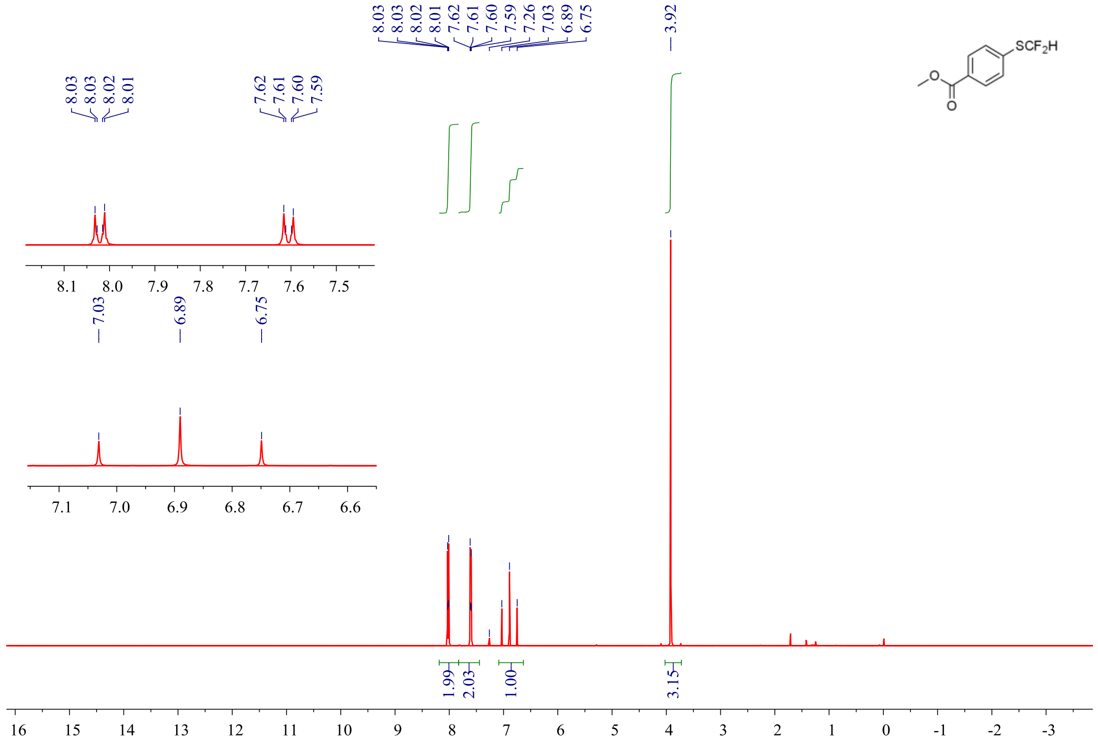

^{19}F NMR (376 MHz, $CDCl_3$) Methyl 4-((difluoromethyl)thio)benzoate 5q

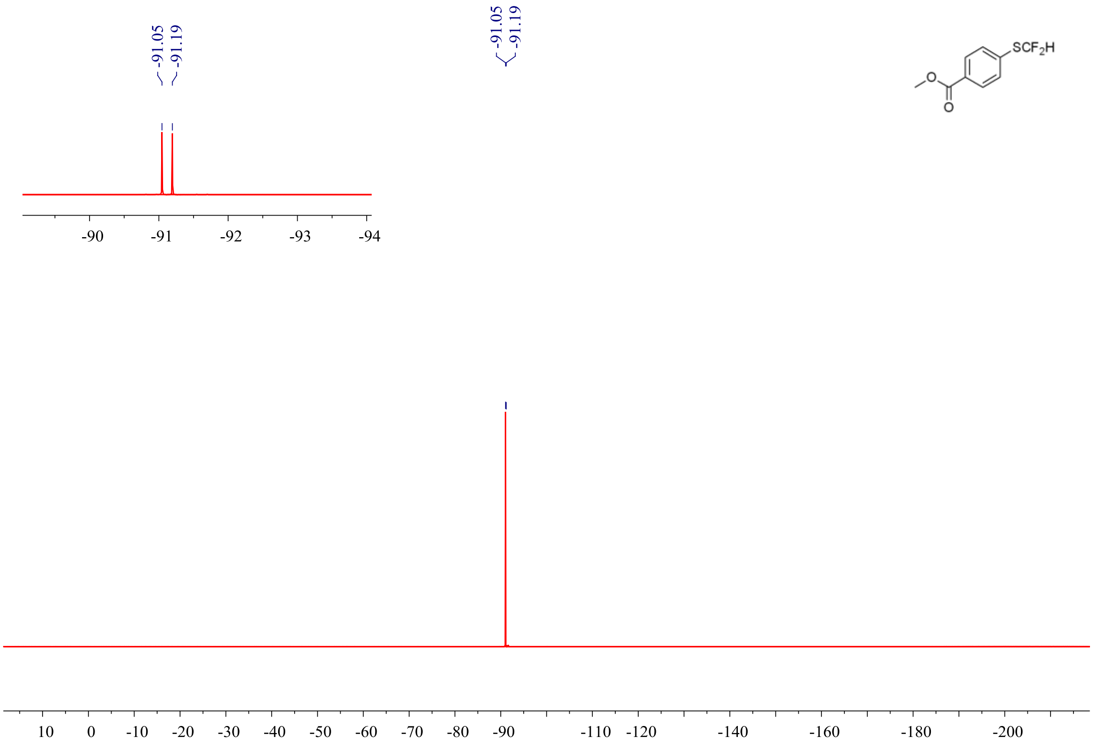

^{13}C NMR (101 MHz, $CDCl_3$) Methyl 4-((difluoromethyl)thio)benzoate 5q

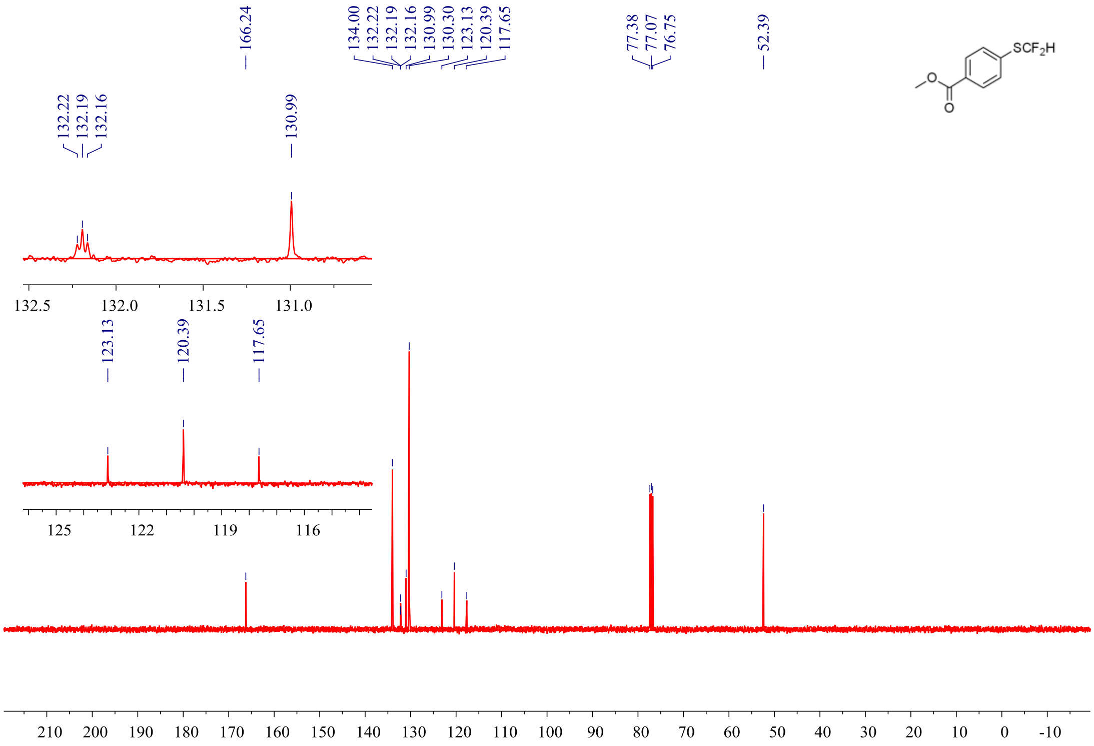

^{1}H NMR (400 MHz, $CDCl_3$) 4-((difluoromethyl)thio)benzonitrile 5r

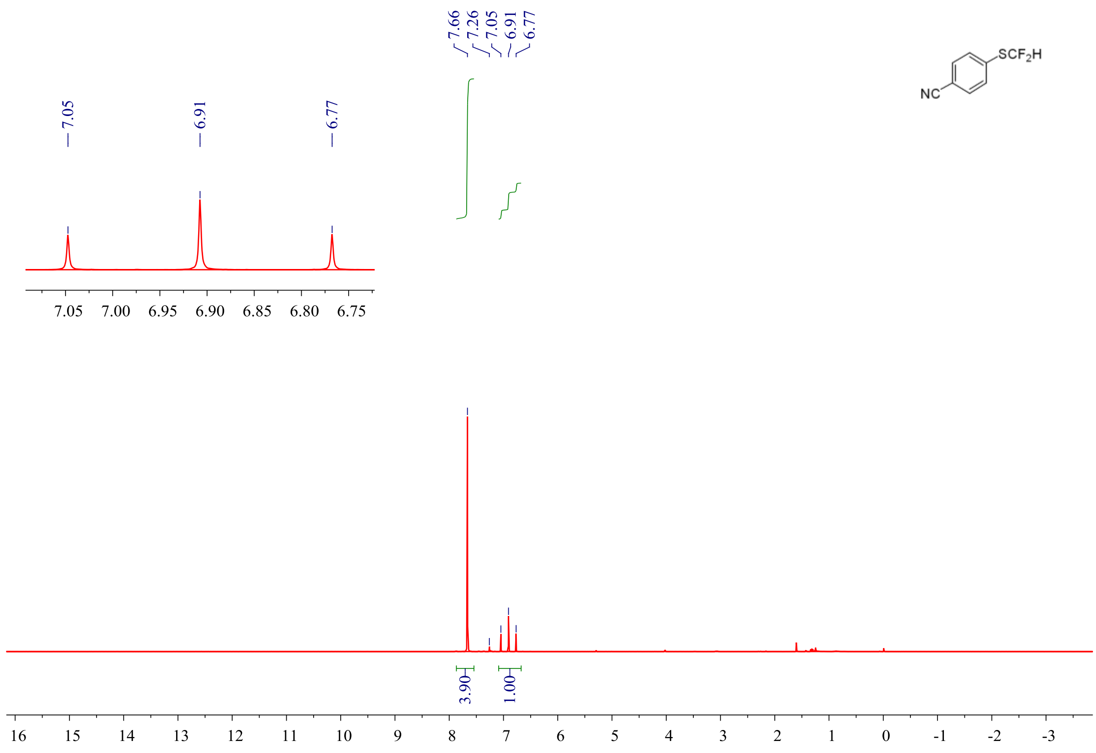

^{19}F NMR (376 MHz, $CDCl_3$) 4-((difluoromethyl)thio)benzonitrile 5r

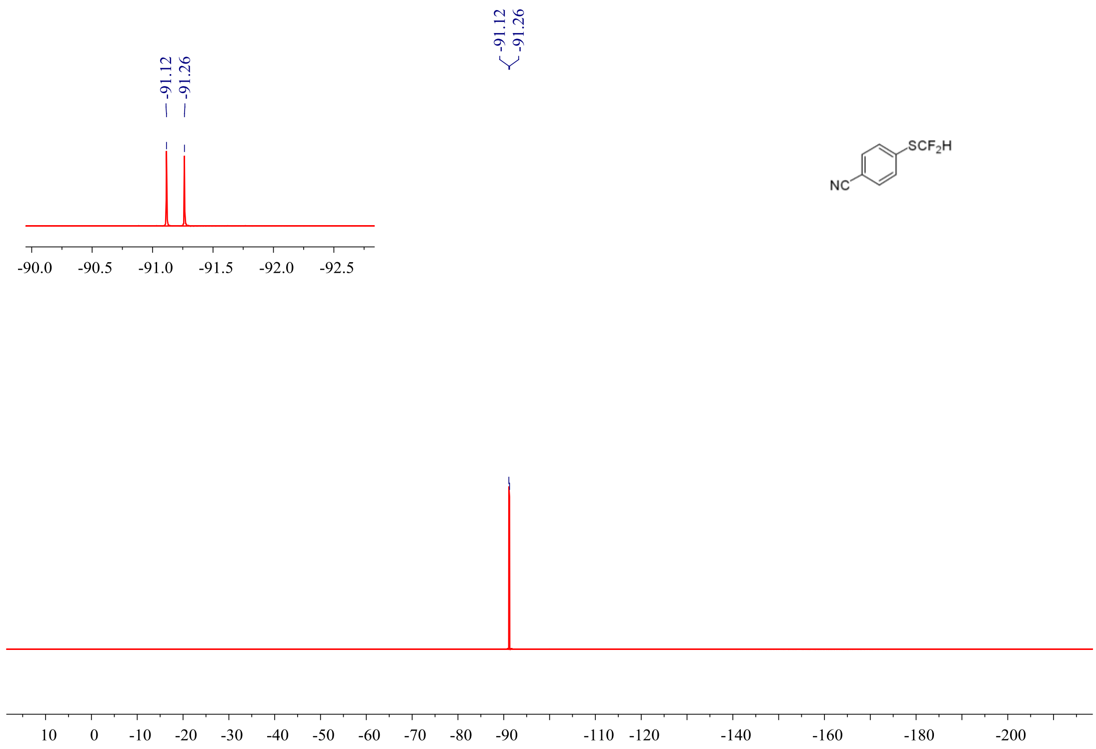

^{13}C NMR (101 MHz, $CDCl_3$) 4-((difluoromethyl)thio)benzonitrile 5r

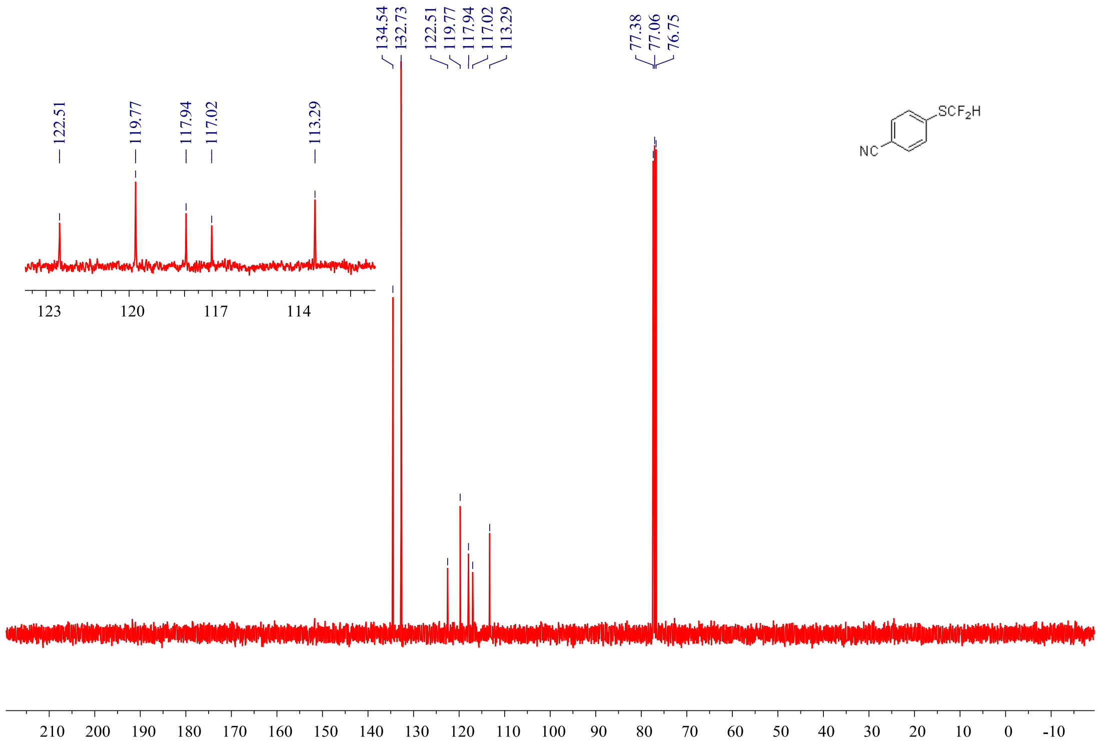

^{1}H NMR (400 MHz, $CDCl_3$) 4-((difluoromethyl)thio)-1-isobutyl-1*H*-imidazo[4,5-c]quinolone 6

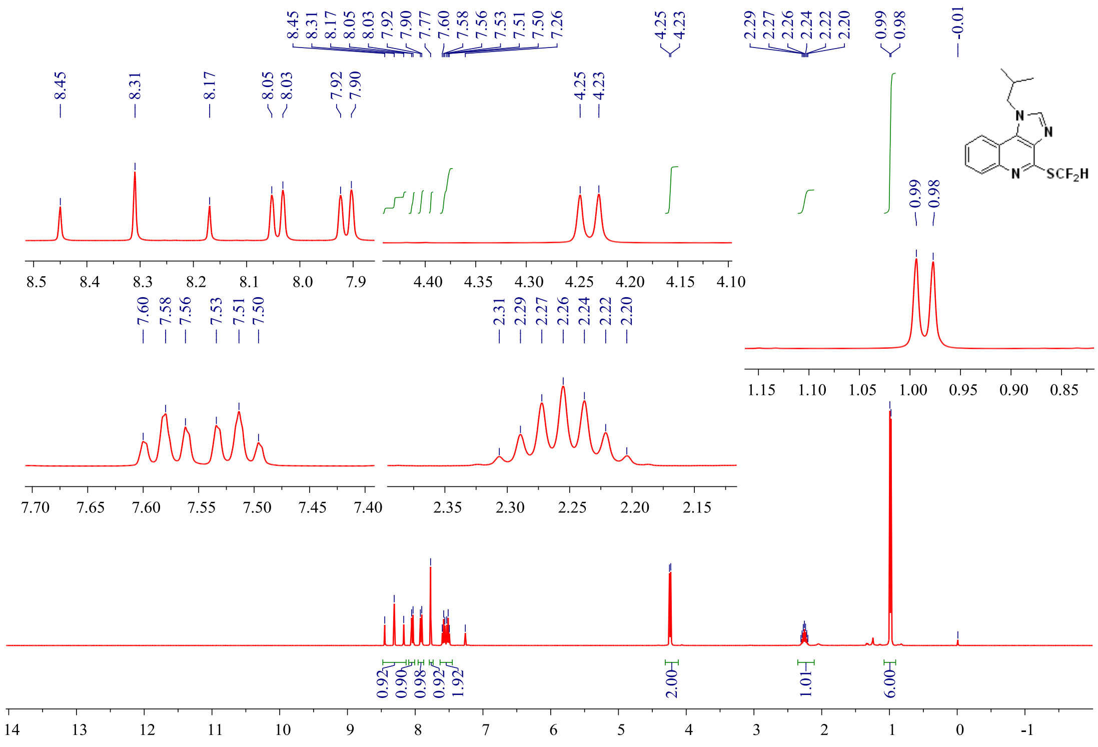

^{19}F NMR (376 MHz, $CDCl_3$) 4-((difluoromethyl)thio)-1-isobutyl-1*H*-imidazo[4,5-c]quinolone 6

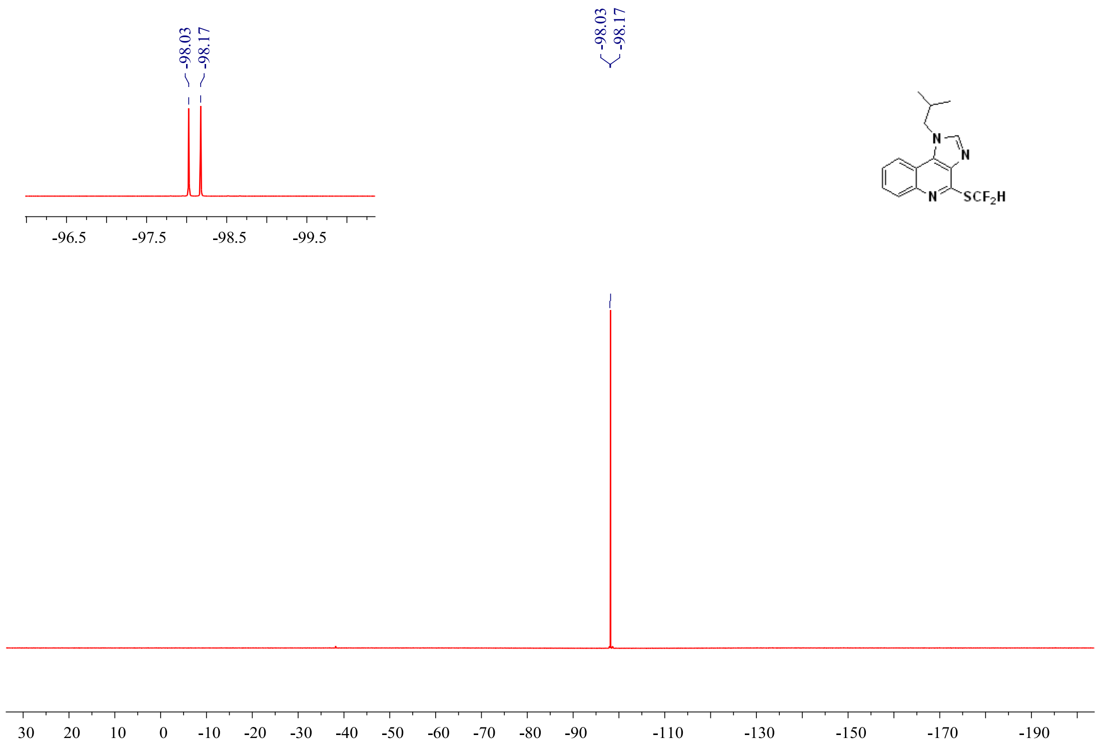

^{13}C NMR (101 MHz, $CDCl_3$) 4-((difluoromethyl)thio)-1-isobutyl-1*H*-imidazo[4,5-c]quinolone 6

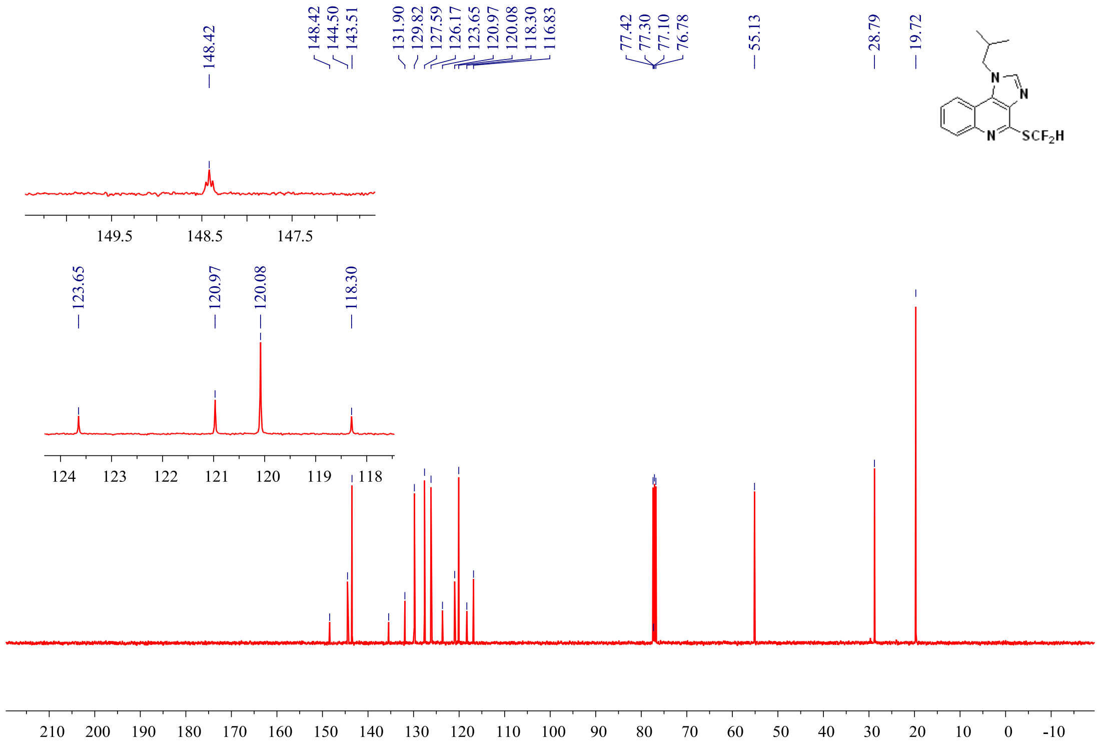

^{1}H NMR (400 MHz, $CDCl_3$) heptan-2-yl 2-((5-chloro-3-((difluoromethyl)thio) quinolin-8-yl)oxy)acetate 7

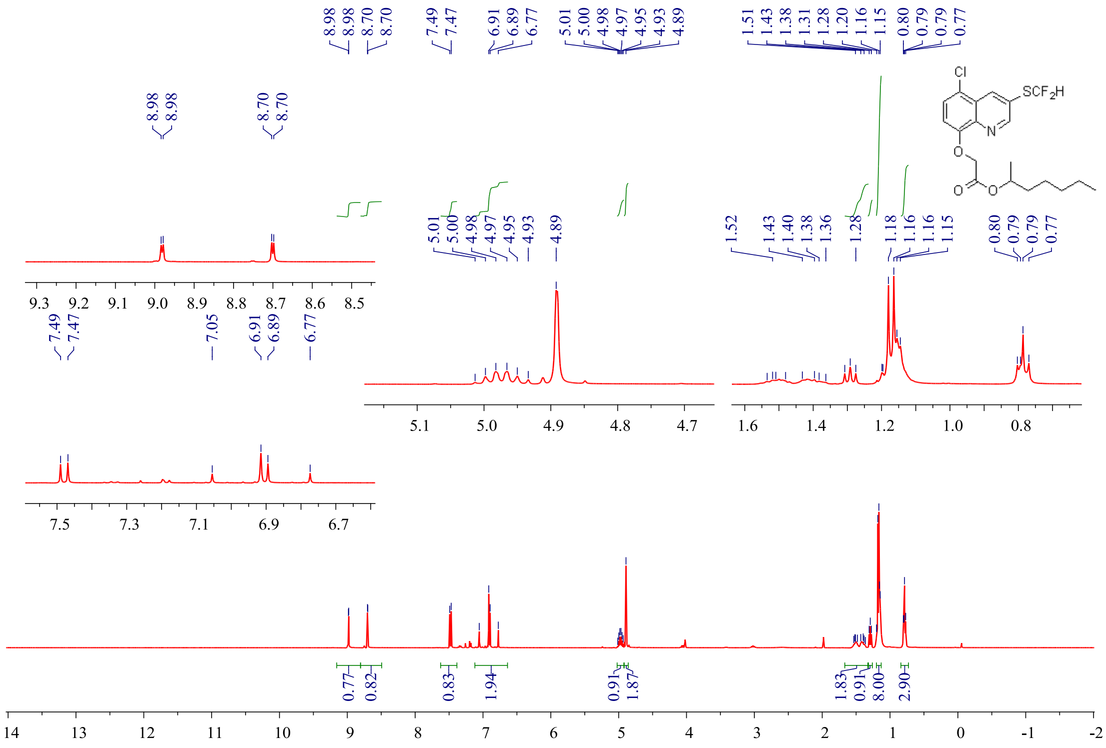

^{19}F NMR (376 MHz, $CDCl_3$) heptan-2-yl 2-((5-chloro-3-((difluoromethyl)thio) quinolin-8-yl)oxy)acetate 7

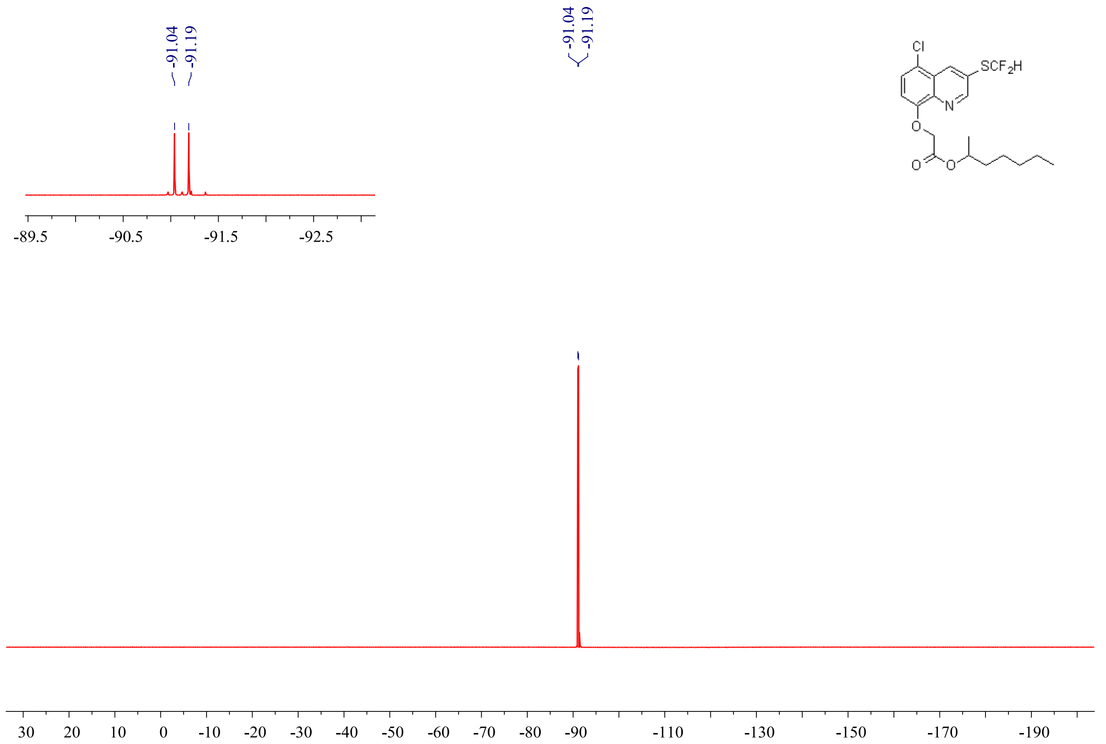

^{13}C NMR (101 MHz, $CDCl_3$) heptan-2-yl 2-((5-chloro-3-((difluoromethyl)thio) quinolin-8-yl)oxy)acetate 7

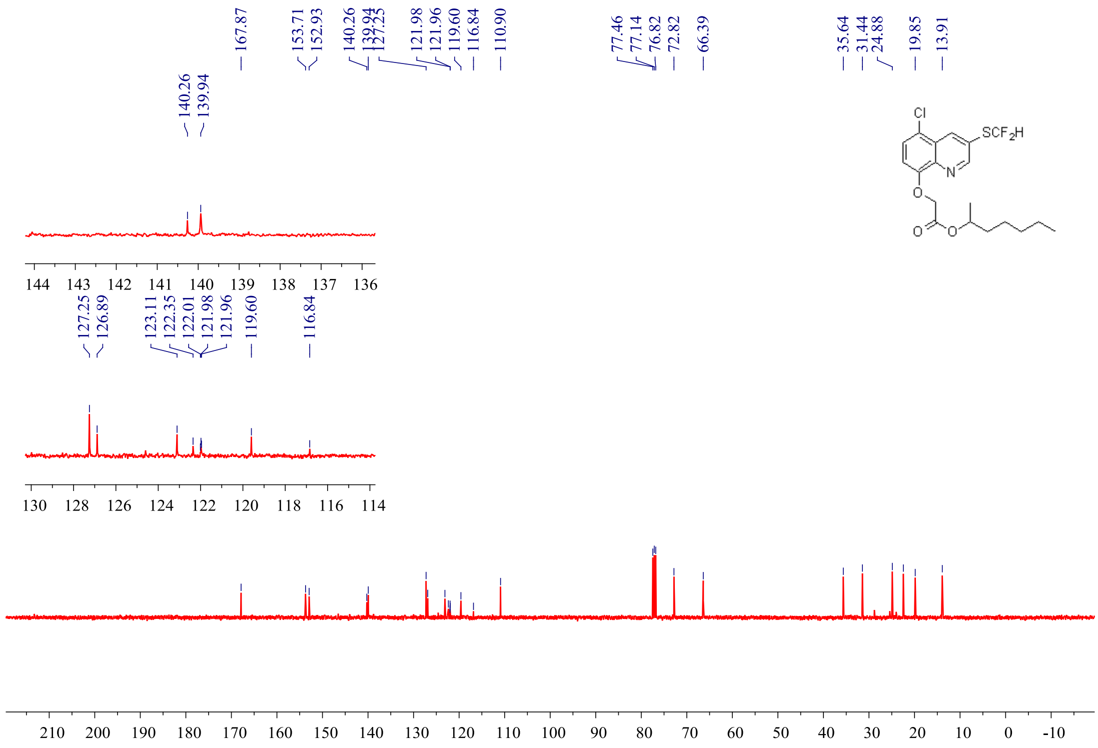

X-ray structure of [(Xantphos)Pd(3-py)(Br)]

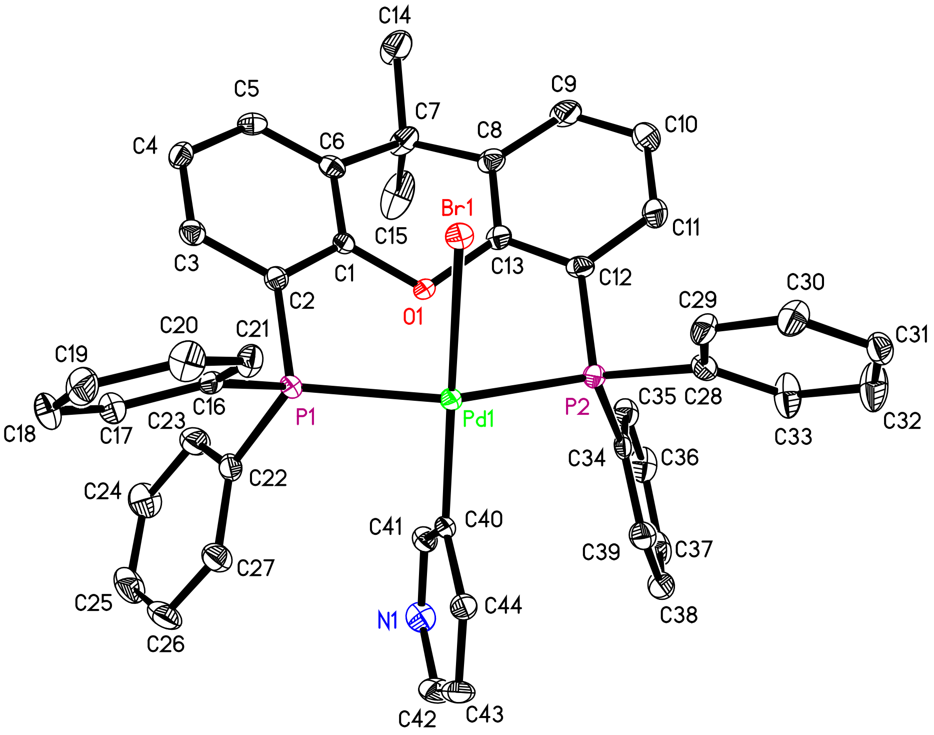

Figure S1. X-ray structure of [(Xantphos)Pd(3-py)(Br)].

Table S1. Crystal data and structure refinement for [(Xantphos)Pd(3-py)(Br)].

Identification code	mo_dm15772_0m	
Empirical formula	C44 H36 Br N O P2 Pd	
Formula weight	842.99	
Temperature	130 K	
Wavelength	0.71073 Å	
Crystal system	Triclinic	
Space group	P -1	
Unit cell dimensions	a = 11.120(2) Å	α= 69.218(4) °.
	b = 12.074(3) Å	β= 72.309(4) °.
	c = 15.487(4) Å	γ = 70.811(4) °.
Volume	1794.2(7) $Å^3$	
Z	2	
Density (calculated)	1.560 Mg/m^3	
Absorption coefficient	1.757 mm^{-1}	
F(000)	852	
Crystal size	0.15 x 0.1 x 0.08 mm^3	
Theta range for data collection	1.862 to 27.714 °.	
Index ranges	-14<=h<=12, -14<=k<=15, -20<=l<=20	
Reflections collected	14978	
Independent reflections	8389 [R(int) = 0.0553]	
Completeness to theta = 26.000 °	100.0 %	
Absorption correction	Semi-empirical from equivalents	
Max. and min. transmission	0.7456 and 0.4851	
Refinement method	Full-matrix least-squares on F^2	
Data / restraints / parameters	8389 / 0 / 453	
Goodness-of-fit on F^2	0.972	
Final R indices [I>2sigma(I)]	R1 = 0.0544, wR2 = 0.1207	
R indices (all data)	R1 = 0.0928, wR2 = 0.1383	
Extinction coefficient	n/a	
Largest diff. peak and hole	1.368 and -1.253 e.$Å^{-3}$	

Table S2. Atomic coordinates (x 10^4) and equivalent isotropic displacement parameters ($Å^2$x 10^3) for mo_dm15772_0m. U(eq) is defined as one third of the trace of the orthogonalized U^{ij} tensor.

	x	y	z	U(eq)
Pd(1)	4779(1)	1184(1)	3119(1)	19(1)
Br(1)	3435(1)	47(1)	2818(1)	27(1)
P(1)	6583(1)	387(1)	2111(1)	21(1)
P(2)	2956(1)	2736(1)	3373(1)	21(1)
O(1)	4426(3)	2597(3)	1387(2)	20(1)
N(1)	7079(4)	3428(4)	3087(4)	33(1)
C(1)	5056(4)	1902(5)	749(4)	21(1)
C(2)	6066(5)	882(5)	985(4)	22(1)
C(3)	6715(5)	237(5)	321(4)	25(1)
C(4)	6353(5)	582(5)	-529(4)	27(1)
C(5)	5314(5)	1591(5)	-728(4)	24(1)
C(6)	4664(5)	2271(5)	-103(4)	23(1)
C(7)	3601(6)	3429(5)	-306(4)	33(1)
C(8)	2620(5)	3507(5)	620(4)	28(1)
C(9)	1298(6)	4017(6)	700(5)	43(2)
C(10)	481(6)	4086(6)	1565(5)	47(2)
C(11)	980(5)	3677(5)	2362(4)	34(1)
C(12)	2316(5)	3185(5)	2315(4)	25(1)
C(13)	3110(5)	3105(4)	1434(4)	24(1)
C(14)	2956(7)	3463(7)	-1058(5)	62(2)
C(15)	4189(8)	4532(6)	-655(5)	62(2)
C(16)	7253(5)	-1265(5)	2395(4)	24(1)
C(17)	8543(5)	-1782(5)	2026(4)	33(1)
C(18)	9028(6)	-3040(6)	2314(5)	39(2)
C(19)	8250(6)	-3790(5)	2952(4)	37(1)
C(20)	6994(6)	-3274(5)	3321(4)	37(1)
C(21)	6501(5)	-2024(5)	3051(4)	27(1)
C(22)	8053(5)	926(5)	1771(4)	26(1)
C(23)	8487(5)	1597(6)	869(4)	39(2)
C(24)	9700(6)	1872(7)	628(5)	46(2)
C(25)	10444(6)	1468(6)	1300(5)	46(2)
C(26)	9986(6)	817(6)	2205(5)	41(2)
C(27)	8796(5)	554(5)	2450(4)	33(1)

C(28)	1572(5)	2477(5)	4359(4)	26(1)
C(29)	1319(5)	1340(5)	4673(4)	30(1)
C(30)	272(6)	1106(6)	5405(4)	35(1)
C(31)	-507(5)	1983(5)	5839(4)	31(1)
C(32)	-247(6)	3105(6)	5532(5)	50(2)
C(33)	788(6)	3363(5)	4792(5)	42(2)
C(34)	3185(4)	4174(5)	3359(4)	23(1)
C(35)	2922(5)	5232(5)	2651(4)	30(1)
C(36)	3147(6)	6300(6)	2649(4)	38(1)
C(37)	3605(5)	6312(6)	3371(5)	38(2)
C(38)	3828(5)	5267(6)	4105(5)	39(2)
C(39)	3622(5)	4197(5)	4103(4)	32(1)
C(40)	5863(4)	1922(5)	3483(4)	21(1)
C(41)	6355(5)	2909(5)	2874(4)	25(1)
C(42)	7340(6)	2938(5)	3944(5)	36(1)
C(43)	6895(5)	1974(5)	4601(4)	33(1)
C(44)	6138(5)	1449(5)	4373(4)	24(1)

Table S3. Bond lengths [Å] and angles [°] for mo_dm15772_0m.

Pd(1)-Br(1)	2.5605(7)
Pd(1)-P(1)	2.2982(14)
Pd(1)-P(2)	2.3059(14)
Pd(1)-C(40)	2.010(5)
P(1)-C(2)	1.835(5)
P(1)-C(16)	1.824(5)
P(1)-C(22)	1.818(5)
P(2)-C(12)	1.820(5)
P(2)-C(28)	1.830(5)
P(2)-C(34)	1.827(5)
O(1)-C(1)	1.398(6)
O(1)-C(13)	1.379(6)
N(1)-C(41)	1.337(7)
N(1)-C(42)	1.326(7)
C(1)-C(2)	1.390(7)
C(1)-C(6)	1.391(7)
C(2)-C(3)	1.391(7)
C(3)-H(3)	0.9500
C(3)-C(4)	1.375(7)
C(4)-H(4)	0.9500
C(4)-C(5)	1.392(7)
C(5)-H(5)	0.9500
C(5)-C(6)	1.372(7)
C(6)-C(7)	1.509(7)
C(7)-C(8)	1.528(8)
C(7)-C(14)	1.526(8)
C(7)-C(15)	1.530(9)
C(8)-C(9)	1.379(8)
C(8)-C(13)	1.390(7)
C(9)-H(9)	0.9500
C(9)-C(10)	1.384(9)
C(10)-H(10)	0.9500
C(10)-C(11)	1.372(8)
C(11)-H(11)	0.9500
C(11)-C(12)	1.396(7)
C(12)-C(13)	1.398(7)

C(14)-H(14A)	0.9800
C(14)-H(14B)	0.9800
C(14)-H(14C)	0.9800
C(15)-H(15A)	0.9800
C(15)-H(15B)	0.9800
C(15)-H(15C)	0.9800
C(16)-C(17)	1.393(7)
C(16)-C(21)	1.377(7)
C(17)-H(17)	0.9500
C(17)-C(18)	1.387(8)
C(18)-H(18)	0.9500
C(18)-C(19)	1.380(9)
C(19)-H(19)	0.9500
C(19)-C(20)	1.364(8)
C(20)-H(20)	0.9500
C(20)-C(21)	1.378(8)
C(21)-H(21)	0.9500
C(22)-C(23)	1.373(8)
C(22)-C(27)	1.392(7)
C(23)-H(23)	0.9500
C(23)-C(24)	1.403(8)
C(24)-H(24)	0.9500
C(24)-C(25)	1.380(9)
C(25)-H(25)	0.9500
C(25)-C(26)	1.374(9)
C(26)-H(26)	0.9500
C(26)-C(27)	1.372(8)
C(27)-H(27)	0.9500
C(28)-C(29)	1.377(8)
C(28)-C(33)	1.376(7)
C(29)-H(29)	0.9500
C(29)-C(30)	1.381(8)
C(30)-H(30)	0.9500
C(30)-C(31)	1.367(8)
C(31)-H(31)	0.9500
C(31)-C(32)	1.365(8)
C(32)-H(32)	0.9500
C(32)-C(33)	1.385(8)

C(33)-H(33)	0.9500
C(34)-C(35)	1.369(8)
C(34)-C(39)	1.392(7)
C(35)-H(35)	0.9500
C(35)-C(36)	1.391(8)
C(36)-H(36)	0.9500
C(36)-C(37)	1.369(9)
C(37)-H(37)	0.9500
C(37)-C(38)	1.376(9)
C(38)-H(38)	0.9500
C(38)-C(39)	1.386(8)
C(39)-H(39)	0.9500
C(40)-C(41)	1.389(7)
C(40)-C(44)	1.379(7)
C(41)-H(41)	0.9500
C(42)-H(42)	0.9500
C(42)-C(43)	1.366(8)
C(43)-H(43)	0.9500
C(43)-C(44)	1.388(7)
C(44)-H(44)	0.9500
P(1)-Pd(1)-Br(1)	90.38(4)
P(1)-Pd(1)-P(2)	149.77(5)
P(2)-Pd(1)-Br(1)	91.24(4)
C(40)-Pd(1)-Br(1)	173.67(15)
C(40)-Pd(1)-P(1)	90.35(14)
C(40)-Pd(1)-P(2)	91.30(14)
C(2)-P(1)-Pd(1)	104.33(16)
C(16)-P(1)-Pd(1)	119.20(18)
C(16)-P(1)-C(2)	107.6(2)
C(22)-P(1)-Pd(1)	120.31(18)
C(22)-P(1)-C(2)	102.5(2)
C(22)-P(1)-C(16)	101.3(2)
C(12)-P(2)-Pd(1)	102.70(17)
C(12)-P(2)-C(28)	105.0(2)
C(12)-P(2)-C(34)	102.2(2)
C(28)-P(2)-Pd(1)	122.11(18)
C(34)-P(2)-Pd(1)	118.37(16)

C(34)-P(2)-C(28)	103.9(2)
C(13)-O(1)-C(1)	116.1(4)
C(42)-N(1)-C(41)	116.2(5)
C(2)-C(1)-O(1)	118.5(4)
C(2)-C(1)-C(6)	122.7(5)
C(6)-C(1)-O(1)	118.7(4)
C(1)-C(2)-P(1)	122.9(4)
C(1)-C(2)-C(3)	117.4(5)
C(3)-C(2)-P(1)	119.7(4)
C(2)-C(3)-H(3)	119.4
C(4)-C(3)-C(2)	121.2(5)
C(4)-C(3)-H(3)	119.4
C(3)-C(4)-H(4)	120.2
C(3)-C(4)-C(5)	119.6(5)
C(5)-C(4)-H(4)	120.2
C(4)-C(5)-H(5)	119.3
C(6)-C(5)-C(4)	121.3(5)
C(6)-C(5)-H(5)	119.3
C(1)-C(6)-C(7)	118.1(5)
C(5)-C(6)-C(1)	117.7(5)
C(5)-C(6)-C(7)	124.0(5)
C(6)-C(7)-C(8)	108.3(4)
C(6)-C(7)-C(14)	111.1(5)
C(6)-C(7)-C(15)	109.2(5)
C(8)-C(7)-C(15)	107.3(5)
C(14)-C(7)-C(8)	111.8(5)
C(14)-C(7)-C(15)	109.0(6)
C(9)-C(8)-C(7)	125.0(5)
C(9)-C(8)-C(13)	117.6(5)
C(13)-C(8)-C(7)	117.3(5)
C(8)-C(9)-H(9)	119.3
C(8)-C(9)-C(10)	121.3(5)
C(10)-C(9)-H(9)	119.3
C(9)-C(10)-H(10)	119.9
C(11)-C(10)-C(9)	120.3(5)
C(11)-C(10)-H(10)	119.9
C(10)-C(11)-H(11)	119.7
C(10)-C(11)-C(12)	120.7(6)

C(12)-C(11)-H(11)	119.7
C(11)-C(12)-P(2)	119.4(4)
C(11)-C(12)-C(13)	117.6(5)
C(13)-C(12)-P(2)	122.9(4)
O(1)-C(13)-C(8)	119.6(5)
O(1)-C(13)-C(12)	117.8(4)
C(8)-C(13)-C(12)	122.6(5)
C(7)-C(14)-H(14A)	109.5
C(7)-C(14)-H(14B)	109.5
C(7)-C(14)-H(14C)	109.5
H(14A)-C(14)-H(14B)	109.5
H(14A)-C(14)-H(14C)	109.5
H(14B)-C(14)-H(14C)	109.5
C(7)-C(15)-H(15A)	109.5
C(7)-C(15)-H(15B)	109.5
C(7)-C(15)-H(15C)	109.5
H(15A)-C(15)-H(15B)	109.5
H(15A)-C(15)-H(15C)	109.5
H(15B)-C(15)-H(15C)	109.5
C(17)-C(16)-P(1)	121.8(4)
C(21)-C(16)-P(1)	119.4(4)
C(21)-C(16)-C(17)	118.6(5)
C(16)-C(17)-H(17)	120.3
C(18)-C(17)-C(16)	119.5(6)
C(18)-C(17)-H(17)	120.3
C(17)-C(18)-H(18)	119.4
C(19)-C(18)-C(17)	121.1(5)
C(19)-C(18)-H(18)	119.4
C(18)-C(19)-H(19)	120.5
C(20)-C(19)-C(18)	119.0(5)
C(20)-C(19)-H(19)	120.5
C(19)-C(20)-H(20)	119.7
C(19)-C(20)-C(21)	120.6(6)
C(21)-C(20)-H(20)	119.7
C(16)-C(21)-C(20)	121.2(5)
C(16)-C(21)-H(21)	119.4
C(20)-C(21)-H(21)	119.4
C(23)-C(22)-P(1)	122.6(4)

C(23)-C(22)-C(27)	120.0(5)
C(27)-C(22)-P(1)	117.2(4)
C(22)-C(23)-H(23)	120.1
C(22)-C(23)-C(24)	119.7(6)
C(24)-C(23)-H(23)	120.1
C(23)-C(24)-H(24)	120.2
C(25)-C(24)-C(23)	119.7(6)
C(25)-C(24)-H(24)	120.2
C(24)-C(25)-H(25)	120.0
C(26)-C(25)-C(24)	120.0(6)
C(26)-C(25)-H(25)	120.0
C(25)-C(26)-H(26)	119.6
C(27)-C(26)-C(25)	120.8(6)
C(27)-C(26)-H(26)	119.6
C(22)-C(27)-H(27)	120.1
C(26)-C(27)-C(22)	119.8(6)
C(26)-C(27)-H(27)	120.1
C(29)-C(28)-P(2)	117.8(4)
C(33)-C(28)-P(2)	122.8(4)
C(33)-C(28)-C(29)	119.4(5)
C(28)-C(29)-H(29)	120.0
C(28)-C(29)-C(30)	119.9(5)
C(30)-C(29)-H(29)	120.0
C(29)-C(30)-H(30)	119.6
C(31)-C(30)-C(29)	120.9(5)
C(31)-C(30)-H(30)	119.6
C(30)-C(31)-H(31)	120.4
C(32)-C(31)-C(30)	119.1(5)
C(32)-C(31)-H(31)	120.4
C(31)-C(32)-H(32)	119.6
C(31)-C(32)-C(33)	120.9(6)
C(33)-C(32)-H(32)	119.6
C(28)-C(33)-C(32)	119.8(6)
C(28)-C(33)-H(33)	120.1
C(32)-C(33)-H(33)	120.1
C(35)-C(34)-P(2)	122.4(4)
C(35)-C(34)-C(39)	118.9(5)
C(39)-C(34)-P(2)	118.7(4)

C(34)-C(35)-H(35)	119.6
C(34)-C(35)-C(36)	120.7(5)
C(36)-C(35)-H(35)	119.6
C(35)-C(36)-H(36)	119.9
C(37)-C(36)-C(35)	120.2(6)
C(37)-C(36)-H(36)	119.9
C(36)-C(37)-H(37)	120.1
C(36)-C(37)-C(38)	119.7(5)
C(38)-C(37)-H(37)	120.1
C(37)-C(38)-H(38)	119.9
C(37)-C(38)-C(39)	120.3(6)
C(39)-C(38)-H(38)	119.9
C(34)-C(39)-H(39)	119.9
C(38)-C(39)-C(34)	120.2(6)
C(38)-C(39)-H(39)	119.9
C(41)-C(40)-Pd(1)	122.8(4)
C(44)-C(40)-Pd(1)	119.9(4)
C(44)-C(40)-C(41)	117.4(5)
N(1)-C(41)-C(40)	124.7(5)
N(1)-C(41)-H(41)	117.6
C(40)-C(41)-H(41)	117.6
N(1)-C(42)-H(42)	118.1
N(1)-C(42)-C(43)	123.8(5)
C(43)-C(42)-H(42)	118.1
C(42)-C(43)-H(43)	120.3
C(42)-C(43)-C(44)	119.4(5)
C(44)-C(43)-H(43)	120.3
C(40)-C(44)-C(43)	118.5(5)
C(40)-C(44)-H(44)	120.8
C(43)-C(44)-H(44)	120.8

Symmetry transformations used to generate equivalent atoms:

Table S4. Anisotropic displacement parameters ($Å^2$x 10^3) for mo_dm15772_0m. The anisotropic displacement factor exponent takes the form: $-2\pi^2[h^2 a^{*2}U^{11} + ... + 2 h k a^* b^* U^{12}]$

	U^{11}	U^{22}	U^{33}	U^{23}	U^{13}	U^{12}
Pd(1)	17(1)	19(1)	21(1)	-8(1)	-5(1)	-2(1)
Br(1)	25(1)	29(1)	30(1)	-12(1)	-6(1)	-7(1)
P(1)	16(1)	22(1)	23(1)	-8(1)	-6(1)	-2(1)
P(2)	20(1)	19(1)	23(1)	-9(1)	-5(1)	-2(1)
O(1)	20(2)	22(2)	20(2)	-10(2)	-5(1)	-4(1)
N(1)	33(3)	28(3)	38(3)	-8(2)	-7(2)	-10(2)
C(1)	16(2)	25(3)	24(3)	-11(2)	-2(2)	-8(2)
C(2)	20(2)	27(3)	21(3)	-8(2)	-1(2)	-9(2)
C(3)	22(3)	29(3)	24(3)	-6(2)	-3(2)	-9(2)
C(4)	28(3)	29(3)	25(3)	-12(2)	0(2)	-9(2)
C(5)	31(3)	23(3)	23(3)	-2(2)	-9(2)	-14(2)
C(6)	21(2)	18(3)	29(3)	-2(2)	-9(2)	-7(2)
C(7)	50(4)	21(3)	24(3)	-5(2)	-20(3)	6(3)
C(8)	34(3)	22(3)	31(3)	-10(2)	-14(2)	-1(2)
C(9)	43(4)	46(4)	45(4)	-24(3)	-32(3)	17(3)
C(10)	28(3)	57(4)	58(4)	-32(4)	-24(3)	18(3)
C(11)	27(3)	34(3)	40(3)	-19(3)	-10(3)	6(2)
C(12)	28(3)	19(3)	30(3)	-8(2)	-16(2)	0(2)
C(13)	28(3)	13(2)	31(3)	-9(2)	-14(2)	5(2)
C(14)	59(5)	75(5)	54(5)	-40(4)	-39(4)	30(4)
C(15)	94(6)	25(3)	41(4)	-3(3)	4(4)	-6(4)
C(16)	24(3)	26(3)	25(3)	-13(2)	-11(2)	1(2)
C(17)	25(3)	37(3)	34(3)	-15(3)	-7(2)	-1(3)
C(18)	24(3)	37(4)	51(4)	-20(3)	-13(3)	9(3)
C(19)	42(4)	25(3)	39(3)	-11(3)	-16(3)	7(3)
C(20)	44(4)	26(3)	32(3)	-2(3)	-6(3)	-9(3)
C(21)	29(3)	23(3)	23(3)	-7(2)	-4(2)	-1(2)
C(22)	20(3)	27(3)	34(3)	-14(2)	-2(2)	-5(2)
C(23)	29(3)	56(4)	34(3)	-15(3)	-5(2)	-13(3)
C(24)	33(3)	64(5)	41(4)	-13(3)	8(3)	-29(3)
C(25)	23(3)	50(4)	64(5)	-18(4)	-2(3)	-14(3)
C(26)	27(3)	45(4)	59(4)	-15(3)	-20(3)	-8(3)
C(27)	26(3)	38(3)	34(3)	-9(3)	-7(2)	-9(3)

C(28)	21(3)	25(3)	33(3)	-10(2)	-7(2)	-4(2)
C(29)	33(3)	29(3)	29(3)	-16(2)	-2(2)	-5(2)
C(30)	43(3)	33(3)	33(3)	-14(3)	4(3)	-20(3)
C(31)	23(3)	35(3)	35(3)	-14(3)	1(2)	-10(2)
C(32)	47(4)	30(3)	66(5)	-26(3)	18(3)	-14(3)
C(33)	38(3)	22(3)	56(4)	-15(3)	12(3)	-10(3)
C(34)	14(2)	23(3)	30(3)	-15(2)	2(2)	-2(2)
C(35)	27(3)	28(3)	33(3)	-9(3)	0(2)	-11(2)
C(36)	40(3)	29(3)	40(4)	-6(3)	5(3)	-17(3)
C(37)	23(3)	35(3)	60(4)	-26(3)	7(3)	-13(3)
C(38)	29(3)	43(4)	59(4)	-36(3)	-8(3)	-5(3)
C(39)	28(3)	32(3)	38(3)	-15(3)	-10(2)	0(2)
C(40)	15(2)	23(3)	24(3)	-11(2)	-7(2)	2(2)
C(41)	22(3)	24(3)	28(3)	-7(2)	-5(2)	-6(2)
C(42)	37(3)	36(3)	49(4)	-16(3)	-16(3)	-17(3)
C(43)	34(3)	37(3)	35(3)	-12(3)	-18(3)	-7(3)
C(44)	25(3)	24(3)	27(3)	-9(2)	-5(2)	-7(2)

Table S5. Hydrogen coordinates (x 10^4) and isotropic displacement parameters ($Å^2$x 10^3) for mo_dm15772_0m.

	x	y	z	U(eq)
H(3)	7421	-455	457	30
H(4)	6808	135	-977	32
H(5)	5050	1813	-1308	29
H(9)	942	4328	151	52
H(10)	-430	4417	1606	56
H(11)	412	3731	2952	41
H(14A)	3625	3326	-1617	94
H(14B)	2348	4263	-1233	94
H(14C)	2480	2822	-807	94
H(15A)	4649	4490	-191	93
H(15B)	3492	5287	-735	93
H(15C)	4802	4527	-1262	93
H(17)	9086	-1277	1580	39
H(18)	9911	-3392	2069	46
H(19)	8583	-4652	3131	44
H(20)	6454	-3780	3769	44
H(21)	5628	-1680	3321	32
H(23)	7969	1874	410	46
H(24)	10007	2334	5	55
H(25)	11272	1641	1138	55
H(26)	10499	546	2666	49
H(27)	8481	118	3081	39
H(29)	1865	718	4388	36
H(30)	90	326	5610	42
H(31)	-1220	1813	6347	37
H(32)	-784	3718	5831	60
H(33)	957	4149	4583	51
H(35)	2582	5237	2156	36
H(36)	2982	7023	2146	46
H(37)	3768	7040	3366	45
H(38)	4126	5278	4615	47
H(39)	3778	3478	4611	39

H(41)	6164	3240	2261	30
H(42)	7867	3277	4110	43
H(43)	7102	1666	5208	39
H(44)	5817	779	4819	29

Table S6. Torsion angles [°] for mo_dm15772_0m.

Pd(1)-P(1)-C(2)-C(1)	23.9(5)
Pd(1)-P(1)-C(2)-C(3)	-157.3(4)
Pd(1)-P(1)-C(16)-C(17)	-157.6(4)
Pd(1)-P(1)-C(16)-C(21)	17.1(5)
Pd(1)-P(1)-C(22)-C(23)	-111.5(5)
Pd(1)-P(1)-C(22)-C(27)	73.2(5)
Pd(1)-P(2)-C(12)-C(11)	151.5(4)
Pd(1)-P(2)-C(12)-C(13)	-31.1(5)
Pd(1)-P(2)-C(28)-C(29)	-30.2(5)
Pd(1)-P(2)-C(28)-C(33)	149.7(4)
Pd(1)-P(2)-C(34)-C(35)	110.2(4)
Pd(1)-P(2)-C(34)-C(39)	-70.9(4)
Pd(1)-C(40)-C(41)-N(1)	180.0(4)
Pd(1)-C(40)-C(44)-C(43)	179.6(4)
P(1)-C(2)-C(3)-C(4)	179.9(4)
P(1)-C(16)-C(17)-C(18)	175.7(4)
P(1)-C(16)-C(21)-C(20)	-176.7(4)
P(1)-C(22)-C(23)-C(24)	-173.1(5)
P(1)-C(22)-C(27)-C(26)	172.8(5)
P(2)-C(12)-C(13)-O(1)	3.8(7)
P(2)-C(12)-C(13)-C(8)	-176.6(4)
P(2)-C(28)-C(29)-C(30)	-178.9(4)
P(2)-C(28)-C(33)-C(32)	179.8(5)
P(2)-C(34)-C(35)-C(36)	-178.0(4)
P(2)-C(34)-C(39)-C(38)	178.8(4)
O(1)-C(1)-C(2)-P(1)	1.5(6)
O(1)-C(1)-C(2)-C(3)	-177.3(4)
O(1)-C(1)-C(6)-C(5)	178.6(4)
O(1)-C(1)-C(6)-C(7)	1.8(7)
N(1)-C(42)-C(43)-C(44)	0.9(9)
C(1)-O(1)-C(13)-C(8)	-35.0(7)
C(1)-O(1)-C(13)-C(12)	144.6(5)
C(1)-C(2)-C(3)-C(4)	-1.2(7)
C(1)-C(6)-C(7)-C(8)	-36.4(7)
C(1)-C(6)-C(7)-C(14)	-159.6(5)
C(1)-C(6)-C(7)-C(15)	80.1(6)

C(2)-P(1)-C(16)-C(17)	84.1(5)
C(2)-P(1)-C(16)-C(21)	-101.2(4)
C(2)-P(1)-C(22)-C(23)	3.5(5)
C(2)-P(1)-C(22)-C(27)	-171.7(4)
C(2)-C(1)-C(6)-C(5)	-0.2(7)
C(2)-C(1)-C(6)-C(7)	-177.0(5)
C(2)-C(3)-C(4)-C(5)	-0.4(8)
C(3)-C(4)-C(5)-C(6)	1.8(8)
C(4)-C(5)-C(6)-C(1)	-1.5(7)
C(4)-C(5)-C(6)-C(7)	175.1(5)
C(5)-C(6)-C(7)-C(8)	147.0(5)
C(5)-C(6)-C(7)-C(14)	23.9(8)
C(5)-C(6)-C(7)-C(15)	-96.4(6)
C(6)-C(1)-C(2)-P(1)	-179.7(4)
C(6)-C(1)-C(2)-C(3)	1.6(7)
C(6)-C(7)-C(8)-C(9)	-147.0(6)
C(6)-C(7)-C(8)-C(13)	37.4(7)
C(7)-C(8)-C(9)-C(10)	-177.9(6)
C(7)-C(8)-C(13)-O(1)	-3.6(7)
C(7)-C(8)-C(13)-C(12)	176.9(5)
C(8)-C(9)-C(10)-C(11)	2.0(11)
C(9)-C(8)-C(13)-O(1)	-179.5(5)
C(9)-C(8)-C(13)-C(12)	0.9(8)
C(9)-C(10)-C(11)-C(12)	-0.2(10)
C(10)-C(11)-C(12)-P(2)	176.3(5)
C(10)-C(11)-C(12)-C(13)	-1.2(9)
C(11)-C(12)-C(13)-O(1)	-178.8(5)
C(11)-C(12)-C(13)-C(8)	0.8(8)
C(12)-P(2)-C(28)-C(29)	85.8(4)
C(12)-P(2)-C(28)-C(33)	-94.4(5)
C(12)-P(2)-C(34)-C(35)	-1.6(5)
C(12)-P(2)-C(34)-C(39)	177.3(4)
C(13)-O(1)-C(1)-C(2)	-145.1(5)
C(13)-O(1)-C(1)-C(6)	36.0(6)
C(13)-C(8)-C(9)-C(10)	-2.3(10)
C(14)-C(7)-C(8)-C(9)	-24.2(8)
C(14)-C(7)-C(8)-C(13)	160.1(5)
C(15)-C(7)-C(8)-C(9)	95.2(7)

C(15)-C(7)-C(8)-C(13)	-80.4(6)
C(16)-P(1)-C(2)-C(1)	151.4(4)
C(16)-P(1)-C(2)-C(3)	-29.8(5)
C(16)-P(1)-C(22)-C(23)	114.6(5)
C(16)-P(1)-C(22)-C(27)	-60.6(5)
C(16)-C(17)-C(18)-C(19)	0.9(9)
C(17)-C(16)-C(21)-C(20)	-1.8(8)
C(17)-C(18)-C(19)-C(20)	-1.9(9)
C(18)-C(19)-C(20)-C(21)	1.1(9)
C(19)-C(20)-C(21)-C(16)	0.8(9)
C(21)-C(16)-C(17)-C(18)	1.0(8)
C(22)-P(1)-C(2)-C(1)	-102.3(4)
C(22)-P(1)-C(2)-C(3)	76.5(4)
C(22)-P(1)-C(16)-C(17)	-23.0(5)
C(22)-P(1)-C(16)-C(21)	151.7(4)
C(22)-C(23)-C(24)-C(25)	-0.2(10)
C(23)-C(22)-C(27)-C(26)	-2.6(9)
C(23)-C(24)-C(25)-C(26)	-1.0(10)
C(24)-C(25)-C(26)-C(27)	0.4(10)
C(25)-C(26)-C(27)-C(22)	1.4(10)
C(27)-C(22)-C(23)-C(24)	2.0(9)
C(28)-P(2)-C(12)-C(11)	22.9(5)
C(28)-P(2)-C(12)-C(13)	-159.8(4)
C(28)-P(2)-C(34)-C(35)	-110.7(4)
C(28)-P(2)-C(34)-C(39)	68.2(4)
C(28)-C(29)-C(30)-C(31)	-1.4(9)
C(29)-C(28)-C(33)-C(32)	-0.4(9)
C(29)-C(30)-C(31)-C(32)	0.7(9)
C(30)-C(31)-C(32)-C(33)	0.1(10)
C(31)-C(32)-C(33)-C(28)	-0.3(11)
C(33)-C(28)-C(29)-C(30)	1.2(8)
C(34)-P(2)-C(12)-C(11)	-85.3(5)
C(34)-P(2)-C(12)-C(13)	92.0(5)
C(34)-P(2)-C(28)-C(29)	-167.3(4)
C(34)-P(2)-C(28)-C(33)	12.5(5)
C(34)-C(35)-C(36)-C(37)	-1.7(8)
C(35)-C(34)-C(39)-C(38)	-2.3(8)
C(35)-C(36)-C(37)-C(38)	-0.7(9)

C(36)-C(37)-C(38)-C(39)	1.6(9)
C(37)-C(38)-C(39)-C(34)	-0.1(8)
C(39)-C(34)-C(35)-C(36)	3.1(8)
C(41)-N(1)-C(42)-C(43)	-1.2(9)
C(41)-C(40)-C(44)-C(43)	-0.7(7)
C(42)-N(1)-C(41)-C(40)	0.6(8)
C(42)-C(43)-C(44)-C(40)	0.1(8)
C(44)-C(40)-C(41)-N(1)	0.3(8)

Symmetry transformations used to generate equivalent atoms:
